# GSK3β regulates epithelial-mesenchymal transition and cancer stem cell properties in triple-negative breast cancer

**DOI:** 10.1186/s13058-019-1125-0

**Published:** 2019-03-07

**Authors:** Geraldine Vidhya Vijay, Na Zhao, Petra Den Hollander, Mike J. Toneff, Robiya Joseph, Mika Pietila, Joseph H. Taube, Tapasree R. Sarkar, Esmeralda Ramirez-Pena, Steven J. Werden, Maryam Shariati, Ruli Gao, Mary Sobieski, Clifford C. Stephan, Nathalie Sphyris, Noayuki Miura, Peter Davies, Jeffrey T. Chang, Rama Soundararajan, Jeffrey M. Rosen, Sendurai A. Mani

**Affiliations:** 1 Department of Translational Molecular Pathology, UT MD Anderson Cancer Center, Houston, TX USA; 2 Turku Centre for Biotechnology, University of Turku, Tykistökatu 6, 20520 Turku, Finland; 3 Department of Biology, Baylor University, Waco, TX USA; 4 Center for Statistical Bioinformatics, Texas A&M University, College Station, TX USA; 5 Department of Genetics, The University of Texas MD Anderson Cancer Center, Houston, TX 77030 USA; 6 Center for Translational Cancer Research, Texas A&M Health Science Center, Institute of Biosciences and Technology, Houston, TX USA; 7 Department of Biochemistry, Hamamatsu University School of Medicine, Hamamatsu, Japan; 8 Department of Integrative Biology and Pharmacology, School of Medicine, School of Biomedical Informatics, UT Health Sciences Center at Houston, Houston, TX USA; 9 Center for Clinical and Translational Sciences, The University of Texas Health Science Center at Houston, Houston, TX USA; 10 Department of Molecular and Cellular Biology, Baylor College of Medicine, Houston, TX USA; 11 Metastasis Research Center, The University of Texas MD Anderson Cancer Center, Houston, TX USA

**Keywords:** Triple-negative breast cancer (TNBC), Epithelial-mesenchymal transition (EMT), Cancer stem cells (CSCs), Glycogen synthase kinase β (GSK3β), Wnt signaling

## Abstract

**Background:**

Triple-negative breast cancers (TNBCs), which lack receptors for estrogen, progesterone, and amplification of epidermal growth factor receptor 2, are highly aggressive. Consequently, patients diagnosed with TNBCs have reduced overall and disease-free survival rates compared to patients with other subtypes of breast cancer. TNBCs are characterized by the presence of cancer cells with mesenchymal properties, indicating that the epithelial to mesenchymal transition (EMT) plays a major role in the progression of this disease. The EMT program has also been implicated in chemoresistance, tumor recurrence, and induction of cancer stem cell (CSC) properties. Currently, there are no targeted therapies for TNBC, and hence, it is critical to identify the novel targets to treat TNBC.

**Methods:**

A library of compounds was screened for their ability to inhibit EMT in cells with mesenchymal phenotype as assessed using the previously described Z-cad reporters. Of the several drugs tested, GSK3β inhibitors were identified as EMT inhibitors. The effects of GSK3β inhibitors on the properties of TNBC cells with a mesenchymal phenotype were assessed using qRT-PCR, flow cytometry, western blot, mammosphere, and migration and cell viability assays. Publicly available datasets also were analyzed to examine if the expression of GSK3β correlates with the overall survival of breast cancer patients.

**Results:**

We identified a GSK3β inhibitor, BIO, in a drug screen as one of the most potent inhibitors of EMT. BIO and two other GSK3β inhibitors, TWS119 and LiCl, also decreased the expression of mesenchymal markers in several different cell lines with a mesenchymal phenotype. Further, inhibition of GSK3β reduced EMT-related migratory properties of cells with mesenchymal properties. To determine if GSK3β inhibitors target mesenchymal-like cells by affecting the CSC population, we employed mammosphere assays and profiled the stem cell-related cell surface marker CD44+/24− in cells after exposure to GSK3β inhibitors. We found that GSK3β inhibitors indeed decreased the CSC properties of cell types with mesenchymal properties. We treated cells with epithelial and mesenchymal properties with GSK3β inhibitors and found that GSK3β inhibitors selectively kill cells with mesenchymal attributes while sparing cells with epithelial properties. We analyzed patient data to identify genes predictive of poor clinical outcome that could serve as novel therapeutic targets for TNBC. The Wnt signaling pathway is critical to EMT, but among the various factors known to be involved in Wnt signaling, only the higher expression of GSK3β correlated with poorer overall patient survival.

**Conclusions:**

Taken together, our data demonstrate that GSK3β is a potential target for TNBCs and suggest that GSK3β inhibitors could serve as selective inhibitors of EMT and CSC properties for the treatment of a subset of aggressive TNBC. GSK3β inhibitors should be tested for use in combination with standard-of-care drugs in preclinical TNBC models.

**Electronic supplementary material:**

The online version of this article (10.1186/s13058-019-1125-0) contains supplementary material, which is available to authorized users.

## Background

Breast cancer is a leading cause of cancer-related death among women [1, 2]. In the USA, one in eight women will be diagnosed with breast cancer in their lifetime [1, 3]. Unlike patients with tumors that express the estrogen receptor (ER), progesterone receptor (PR), or human epidermal growth factor receptor 2 (HER2), who have chemotherapy and targeted therapy options, patients with triple-negative breast cancers (TNBCs), that lack the expression of ER, PR, and HER2, have limited treatment options. In general, TNBCs are highly aggressive, have a worse prognosis compared to other breast cancer subtypes [4**–**6], and recur at a very high rate [5, 6]. Thus far, no unifying characteristic of these breast cancers has been pinpointed to facilitate targeted treatment. Therefore, it is vital to identify the targets that enable TNBCs to thrive and progress and to design means of targeting these factors to treat these tumors [4].

One of the known characteristics of TNBCs is the predominance of cells with mesenchymal attributes; these cells have undergone epithelial-mesenchymal transition (EMT) and are characterized by the presence of poorly differentiated cancer cells [6–8]. EMT is a dynamic process necessary during embryonic development [9, 10], wound healing, and tumor progression during adulthood [9]. At the molecular level, EMT alters the adhesion ability, polarity, and differentiation characteristics of epithelial cells and renders them more migratory and invasive [9–11]. Induction of EMT leads to an increase in the expression of markers such as fibronectin and vimentin and a decrease in the expression of epithelial markers such as E-cadherin [9, 10]. Several transcription factors have been shown to be potent inducers of EMT including Snail, Twist, and Zeb1 [9, 10]. Ligands such as TGFβ and Wnt have also been shown to be key regulators of EMT [9, 10]. The induction of EMT by any of these factors enhances the metastatic potential of the cancer cells [11]. EMT has been demonstrated to generate cells that are less differentiated and give rise to cancer stem cells (CSCs) [11, 12]. CSCs have self-renewal potential and are capable of giving rise to new cancer stem cells or differentiated daughter cells [13, 14]. Thus, these cells can lead to different clonal populations that result in intratumoral heterogeneity [15–17]. Intratumoral heterogeneity results in the emergence of chemoresistance and subsequent tumor recurrence [16–19]. Therefore, targeting CSCs could be an important means for treatment of EMT- and CSC-rich TNBCs.

A number of signaling pathways have been shown to be responsible for inducing and maintaining CSC properties including the Wnt, Notch, and TGFβ1 pathways [20–26]. Signaling pathways involve multiple molecules, and critical signaling nodes must be identified in order to effectively inhibit a pathway. Among these signaling pathways, Wnt signaling has been shown to play a pivotal role during embryo development [27, 28]. In this study, we aimed to identify small molecule inhibitors with potential as novel therapeutic agents due to their ability to inhibit EMT and to discover signaling molecules that are critical for the maintenance of the EMT and CSC properties in TNBC.

## Methods

### Cell lines

Immortalized human mammary epithelial cells (HMLE), HMLE transduced with EMT transcription factor Snail (HMLE Snail), and Twist (HMLE Twist) and HMLE transformed with V12 H-Ras (HMLER) and overexpressing Snail transcription factor (HMLER Snail) were a generous gift from the Weinberg Lab and were grown in HMLE media, made by mixing MEGM (Lonza) and DMEM/F12 50:50 (Corning) and bovine pituitary extract (BPE) (Lonza), insulin (Sigma), hydrocortisone (Sigma), penicillin, and streptomycin (Gibco/Life Technologies), and were added to the media. SUM159, MCF7, MDA-MB-231, and HEK293T were previously purchased either from ATCC or the MDACC Characterized Cell Line Core (CCLC). Mesenchymal basal-like cells, SUM159, were cultured in Ham’s F12 medium (Corning) containing additional fetal bovine serum (FBS) (Sigma), hydrocortisone, insulin, penicillin, and streptomycin. Epithelial MCF7 cells were cultured in DMEM/F12 media containing 10% FBS, penicillin, and streptomycin. HEK293T cells and p53 null, claudin-low mouse mammary tumor-derived T11 cells [29] were grown in DMEM (Corning) with 10% FBS and were used for transfections. MDA-MB-231 reporter cells [29] that were used for the compound screen were cultured in DMEM media with 10% Tet-approved FBS. Mouse embryonic fibroblasts (MEFs) derived from wild-type and GSK3β knockout mice were grown in DMEM medium with 10% FBS. The cell lines used in this study were validated by STR DNA fingerprinting using the Promega 16 High Sensitivity STR Kit (Catalog # DC2100). The STR profiles were compared to online search databases (DSMZ/ATCC/JCRB/RIKEN) of 2455 known profiles, along with the MDACC CCLC database of 2556 known profiles. The STR profiles matched known DNA fingerprints. Mycoplasma testing was performed for all cell lines used in the lab using MycoAlert kit, Lonza. Experiments were conducted only with cell lines testing negative for mycoplasma. shRNAs to GSK3β in pGIPZ that were purchased from the MD Anderson shRNA core were used to silence GSK3β in HMLE Snail, HMLE Twist, and SUM159 cells. pMIG was modified to express RFP and luciferase to generate pMIRL, which was used to label HMLER Snail cells.

### EMT-MET screen

The goal of the screen was to identify the inhibitors that are capable of inhibiting EMT. Therefore, this particular Selleckchem drug library (Additional file 1: Data S1), which consists of several FDA-approved kinase inhibitors as well as non-FDA-approved novel compounds, was selected. In order to test the efficacy of the selected Selleckchem panel of drugs in inhibiting EMT and promoting MET, we used three different concentrations (0.1 μM, 1 μM, and 10 μM) of each of the drugs included in this panel. These concentrations were chosen such that at least one of the selected concentrations would be within the toxicity limit.

**Fig. 1:**
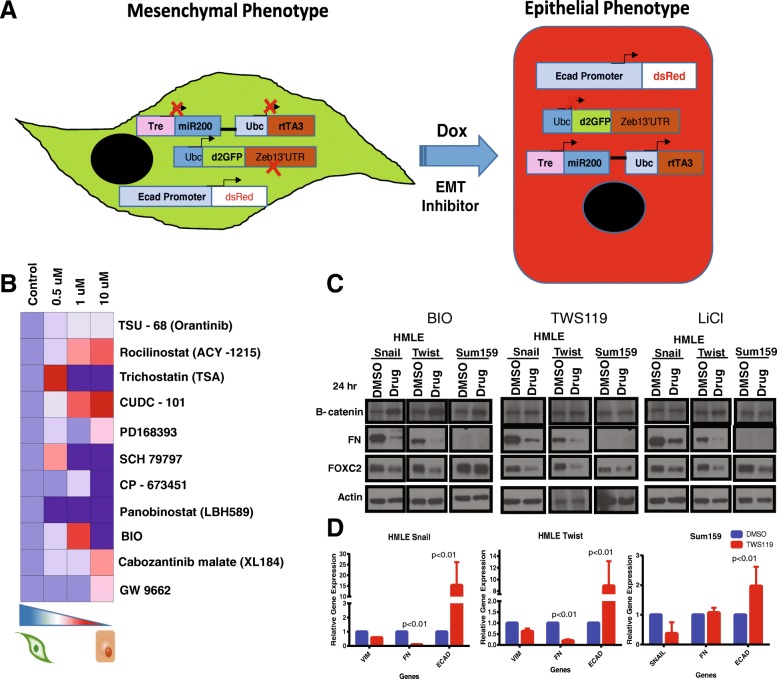
GSK3β inhibitors are one of the few drugs identified in this screen that are capable of inhibiting EMT. **a** Schematic of reporter system in MDA MB 231 reporter cells that were used to screen a panel of small-molecule drugs. In the assay, cells that have a mesenchymal-like phenotype express GFP (green), and those with epithelial cells express RFP (red). **b** The drugs shortlisted in the screen were validated using FACS. MDA-MB-231 cells were treated with three concentrations of all the three drugs (BIO, TWS119, and LiCl), and the proportion of red (epithelial cells) and green (mesenchymal cells) cells were plotted (Additional file 3: Figure S1) and summarized using a heatmap showing the changes in the proportions of epithelial cells and mesenchymal cells upon treatment with indicated inhibitor. **c** Western blot of extracts of HMLE-Snail, HMLE-Twist, and SUM159 cells treated with indicated inhibitors or DMSO and stained for fibronectin (FN), FOXC2, and β-catenin. β-Actin was used as loading control. **d** Expression of mesenchymal and epithelial markers such as vimentin (*VIM*), fibronectin (*FN*), and *E-cadherin* (*ECAD*) were tested in HMLE-Snail, HMLE-Twist, and Sum159 cells treated with TWS119 or DMSO

MDA-MB-231 cells stably transduced with the Z-cad reporters [29] were employed to perform the screen. In this reporter cell line, a destabilized *GFP* has been cloned in front of the *Zeb1* 3′UTR making the mesenchymal-like MDA MB 231 cells green in color. Additionally, these cells also express RFP under the control of the *E-cadherin* promoter and miR-200c-miR-141 cluster under the control of a *doxycycline* (*DOX*)-inducible promoter. Exposure of these cells to DOX induces the expression of miR200, which inhibits the expression of GFP and induces epithelial differentiation. As a result, expression driven by the *E-cadherin* promoter is activated, and, consequently, cells acquire a red color (Fig. 1a) [29]. The reporter cells were plated, and the cells were treated with a library of about 1300 small molecules purchased from Sellekchem; these compounds included inhibitors of kinases, receptor tyrosine kinases, and epigenetic modulators. After 5 days of treatment, the proportion of red to green fluorescence in each well was calculated (Additional file 2: Data S2). The drugs that were able to elicit at least a 1.5-fold increase in the red fluorescence were selected for further analysis.

### Western blot

Western blot assays were used to determine the expression of protein related to the mesenchymal phenotype. Proteins were extracted from the cells using RIPA buffer (Sigma) with kinase inhibitor (Complete from Roche) and phosphatase inhibitor (PhosphoStop from Roche). The concentrations were quantified using BIORad Bradford assay. Fifty micrograms of protein was then loaded for SDS-PAGE. After electrophoresis of the isolated proteins on SDS-PAGE gels, they were transferred to nitrocellulose membranes which were probed with different antibodies of interest and chemiluminescence was used to detect the expression of the proteins, β-actin (Santa Cruz), GSK3β (Cell Signaling), FOXC2 (Miura, Hamamatsu University, Japan), fibronectin (BD Biosciences), and β-catenin (BD Biosciences).

### qRT-PCR

qRT-PCR was performed to evaluate the relative expression of epithelial and mesenchymal markers following treatment with the GSK3β inhibitors. The cells to be analyzed by qRT-PCR were harvested and lysed using Trizol (Life Technologies). Qiagen RNA extraction kit was used to extract RNA from these cells. The RNA was quantified using Nanodrop (Thermoscientific). One thousand nanograms of RNA was used for cDNA synthesis using cDNA synthesis kit (Applied Biosystems). The cDNA generated was used for qRT-PCR analysis. Plates with 96- or 348-well formats were used for this analysis, and the Vii7 system from Applied Biosystems was used to perform this analysis. SyBr green (Applied Biosystems) was used as the detection agent. The CT values generated were used to calculate the fold change in the expression of the gene of interest. The primers used are as follows:

**Table:** 

Primer	Direction	Sequence
E-cadherin	Forward	TGCCCAGAAAATGAAAAAGG
	Reverse	GTGTATGTGGCAATGCGTTC
Vimentin	Forward	GAGAACTTTGCCGTTGAAGC
	Reverse	TCCAGCAGCTTCCTGTAGGT
Snail	Forward	ACCCCACATCCTTCTCACTG
	Reverse	TACAAAAACCCACGCAGACA
Fibronectin	Forward	CAGTGGGAGACCTCGAGAAG
	Reverse	GTCCCTCGGAACATCAGAAA

### Wound healing assay

The wound healing assay was employed to assess the migratory potential of the mesenchymal-like cells. Cells were plated and grown to confluence. Once the cells were confluent, a scratch was made. The scratches were imaged and quantified using a Zeiss microscope. The scratches were treated with either DMSO or a GSK3β inhibitor, and the scratch was imaged and quantified after 9 h. Following this, the scratches were fixed for immunofluorescence studies.

### Immunofluorescence

The immunofluorescence assay was performed to assess the expression of FOXC2 at the wound site. The cells were fixed using 2% paraformaldehyde. The paraformaldehyde was removed, and the cells were washed three times with PBS. The cells were then permeabilized using 10% Tween for 15 min. The Tween was removed by washing the cells three times with PBS. The cells were then treated with glycine for 20 min after which the cells were washed with PBS again. The cells were then stained with the primary antibody overnight. Following the overnight incubation, the cells were washed with PBS and the cells were then stained with the secondary antibody for 4 h. The excess of secondary antibodies was removed by washing the cells three times with PBS. The nuclei of the cells were stained with DAPI, the cells were washed with water, and the cover slip was mounted using Vectashield Mounting Media (DAKO) and sealed to prevent drying. The stained slides were then imaged using Axiom fluorescent microscope.

### Mammosphere assay

Sphere assays were used as a surrogate for measuring the stemness of mesenchymal-like cells. Cells were harvested by trypsinization, and the cells were counted with trypan blue to ensure that the only live cells were plated for the mammosphere assay. One thousand cells were plated into each well of the low attachment 96-well plate in 100 μl of the mammosphere media. The mammosphere media is MEGM media with 1% methylcellulose. EGF (10 ng/ml), FGF (20 ng/ml), and heparin (4 μg/ml) were added to aliquots before feeding the spheres. The spheres were allowed to grow for 10 days after which the spheres with a diameter greater than 100 μm were counted. For drug treatment, the drug was added to the media every time the media was refreshed every 2 days.

### FACS analysis

FACS assay was utilized to determine if there was a change in the expression of cell surface antigens CD24 and CD44. Cells to be used for this analysis were harvested and counted using trypan blue. 5 × 10^5^ cells were used for this analysis. The cells to be analyzed were suspended in FACs buffer (PBS with 2% FBS). CD24 conjugated with PE (BD Biosciences) and CD44 conjugated with APC (BD Biosciences) were incubated with the cells for 30 min following which the cells were thoroughly washed with the FACS buffer. The cells were analyzed using BD Accuri.

### MTT assay

The MTT assays were performed to evaluate the IC50 for each of the cell types for each of the drugs. The 96-plate format was used for this assay. HMLE Snail, HMLE Twist, and SUM159 cells were trypsinized, and viable cells were counted using trypan blue. One thousand cells in 100 μl of media were plated in each of the wells. The cells were allowed to attach, and the following day, the cells were treated with a range of concentrations for each of the drugs. Following the treatment, the MTT reagent (CellTiter 96® AQ_ueous_ One Solution Cell Proliferation Assay from Promega) was added to each of the wells, and the absorbance at 490 nm was evaluated and viability was calculated. Based on this data, the IC50 was calculated using GraphPad Prism (Additional file 3: Figure S1).

### Statistical method

All the experiments were repeated at least three times. All the graphs are represented as mean ± sd, and the *p* values (significance) were calculated using Student’s unpaired two-tailed *t* test. *p* < 0.05 were considered significant.

## Results

### BIO, a 6-bromo derivative of indirubin that inhibits GSK3β activity blocks EMT

TNBCs are characterized by the presence of cells that have undergone EMT. To identify small molecules that could selectively inhibit the proliferation of cells with mesenchymal or stem cell properties, we used MDA MB 231-Z-cad sensor cells expressing EMT reporters (Fig. 1a) [29]. Drugs that induced a change greater than 1.5-fold in the green to red fluorescence ratio compared to untreated cells were further analyzed for effects on Z-cad reporter cells by fluorescently activated cell sorting (FACS) analysis (Table 1). To validate the screen results, the Z-cad reporter cells were plated in 24-well plates and treated with the select compounds at several concentrations, and the cells were sorted for red and green cells following the treatment. Of the 11 drugs that were selected from the initial screen, only 2, CUDC-101 and BIO, were able to induce the expression of RFP in these cells (Fig. 1b, Additional file 4: Figure S2). CUDC-101 is known to target HDAC1, EGFR, and HER2; BIO is known to target GSK3β [30, 31]. Since TNBCs are HER2 negative, and many drugs targeting HDACs are under development, we chose to focus on the GSK3β inhibitor BIO [23, 30, 32, 33]. BIO is a 6-bromo derivative of indirubin, which is an active component of a traditional Chinese medicine used to treat leukemia [34]. BIO is a specific inhibitor of GSK3β kinase activity [34].

**Table 1:** List of the drugs that were most effective in inhibiting EMT in the mesenchymal MDA-MB 231 reporter cells and their reported targets

	Drug	Target
1	TSU-68 (orantinib)	VEGFR, PDGFR, FGFR
2	Rocilinostat (ACY-1215)	HDAC
3	Trichostatin A (TSA)	HDAC
4	CUDC-101	HDAC, HER2, EGFR
5	PD168393	EGFR
6	SCH79797	Par1 antagonist
7	CP-673451	PDGFR
8	Panobinostat (LBH589)	HDAC
9	BIO	GSK3
10	Cabozantinib malate	VEGFR, Axl
11	GW9662	PPAR

### Inhibition of GSK3β decreases mesenchymal properties and inhibits migration

Data from this screen suggested that GSK3β might function as a positive regulator of EMT and may provide an opportunity to target GSK3β. To ensure that this was not specific to BIO, we examined the effects of BIO and two other GSK3β inhibitors, LiCl and TWS119, on EMT. For this analysis, we used a mesenchymal-like TNBC cell line (SUM159) and two ER and PR breast cell lines induced to have mesenchymal characteristics by overexpression of either Snail or Twist (HMLE-Snail and HMLE-Twist lines, respectively) [35]. LiCl has been used in the clinic for more than 60 years, but only for the past decade has it been known to inhibit GSK3β function [36]. The lithium ion competes with magnesium ions that are required for kinase activity [37, 38]. TWS119, a 4,6-disubstituted pyrazolopyrimidine, was identified as a small molecule capable of inducing differentiation of mouse embryonic stem cells that was later discovered to be an inhibitor of GSK3β [39]. TWS119 binds to GSK3β as shown by affinity chromatography, western blot, and surface plasmon resonance [39].

To evaluate the effects of these compounds on EMT and stem cell properties, we harvested RNA and protein from cells treated with the GSK3β inhibitors (BIO 1 μM, TWS119 2 μM, and LiCl 20 mM) and analyzed the expression of epithelial and mesenchymal markers. Following treatment with GSK3β inhibitors, there was a significant decrease in the expression of mesenchymal marker fibronectin in two out of three cell lines tested. Since fibronectin is not expressed in SUM159, we did not observe any change in its expression in these cells (Fig. 1c). We previously demonstrated that FOXC2 is induced following EMT, independent of the EMT inducing signals [40, 41]. Exposure of these mesenchymal-like cells to GSK3β inhibitors also reduced FOXC2 protein expression (Fig. 1c). At the transcript level, we found that the *E-cadherin* expression was increased by treatment with TWS119 (Fig. 1d).

**Fig. 2:**
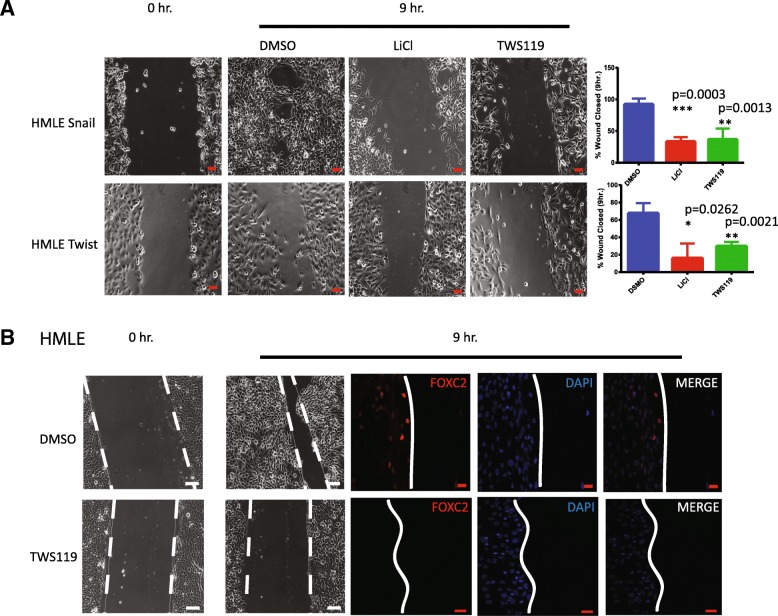
GSK3β inhibitors decrease the migratory properties of cells with mesenchymal phenotype. **a** HMLE-Snail, HMLE-Twist, and SUM159 cultures treated with indicated GSK3β inhibitors or DMSO (control) were wounded. After 9 h, the percentage wound closure was determined. Left: representative images of the wound region. Scale bars 100 μm. Right: plots of percentage wound closure in each cell type (*n* = 3, *p* values were calculated using Student’s unpaired two-tailed *t* test). **b** Cells were stained for FOXC2 (red). The nuclei were labeled with DAPI (blue)

To investigate the effect of the GSK3β inhibitors on migratory properties, we exposed HMLE-Snail and HMLE-Twist to GSK3β inhibitors and assessed the migration using the scratch assay. We observed significantly less wound closure after 9 h in cells treated with GSK3β inhibitors than in control cultures of cells (Fig. 2a). Induction of EMT at the migratory edge of the tumor is a well-known phenomenon [9]. We observed that FOXC2 is upregulated at the migratory front of a wound and that this increase is essential for migration [42]. Therefore, we performed immunofluorescence staining of the wounds in inhibitor-treated and control cultures. In HMLE treated with TWS119, FOXC2 expression was not increased at the wound edge, indicating the absence of induction of EMT (Fig. 2b).

### Inhibition of GSK3β reduces stem cell properties

Cells that have undergone EMT are known to acquire stem cell properties and have enhanced tumor-initiating properties [12, 43]. CSCs and cells that have undergone EMT are also known to be resistant to chemotherapies [19]. The ability to form spheres has been used as a surrogate assay for stem cell properties. We tested the ability of the drugs shortlisted in the screen to inhibit the sphere-forming potential of the MDA MB 231 reporter cells. BIO, the GSK3β inhibitor that was shown to inhibit EMT, was also among the drugs that significantly inhibited the formation of mammospheres by the MDA MB 231 reporter cells (Additional file 5: Figure S3).

**Fig. 3:**
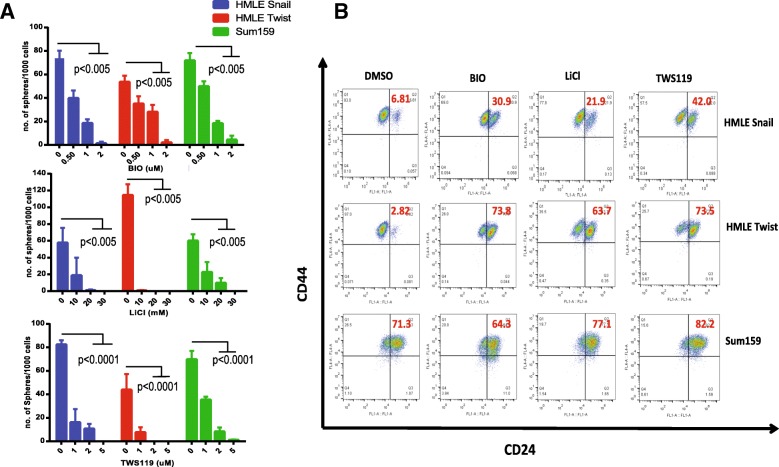
GSK3β inhibitors decrease the cancer stem cells properties of cells with a mesenchymal phenotype. **a** HMLE-Snail, HMLE-Twist, and Sum159 cells were grown in ultra-low attachment plates in mammosphere media for 10 days in the presence of LiCl or TWS119. The number of mammospheres was counted and graphed (*n* = 3, *p* values were calculated using Student’s unpaired two-tailed *t* test). **b** HMLE-Snail, HMLE-Twist, and SUM159 cells were treated with BIO, TWS119, or LiCl and assessed for the presence of CD44 and CD24 by flow cytometry. Treating mesenchymal cells with GSK3β inhibitors increases the expression of CD24 indicating that the cells are more differentiated following treatment

To confirm that multiple GSK3β inhibitors and not BIO (1 μM) alone are capable of inhibiting mammosphere formation, TWS119 (2 μM) and LiCl (20 mM) were also tested to assess their ability to inhibit the CSC population of the mesenchymal-like cell lines. The cells were plated for mammosphere assays and were treated with three different concentrations of the GSK3β inhibitors. All three drugs inhibited sphere formation of the three mesenchymal-like cell lines indicating that the GSK3β inhibitors are capable of inhibiting the stem-like population from forming mammospheres (Fig. 3a). In addition, it was also evident that 24 h pre-treatment with at least two of the three drugs resulted in a significant decrease in the sphere forming ability of HMLE-Snail, HMLE-Twist, and Sum159 cells without significantly affecting their proliferation (Additional file 6: Figure S4A). A simultaneous growth curve generated for these cells showed that pre-treatment with TWS119 and LiCl did not affect the proliferation of these cells (Additional file 6: Figure S4B).

To confirm the involvement of GSK3β in sphere formation, we depleted cells of GSK3β by treatment with shRNA to and found that while HMLE-Snail cells, HMLE-Twist cells, and Sum159 cells transduced with the control vector were capable of forming on an average of 91, 95, and 66 spheres per 1000 cells, respectively, the cells transduced with the shRNAs were only able to form an average of 55, 80, and 30 spheres per 1000 cells, respectively (Additional file 6: Figure S4C). Mouse embryonic fibroblasts (MEFs) isolated from *GSK3β*-null mice formed about 5 times fewer spheres than did the wild-type MEFs (Additional file 6: Figure S4D).

We also performed FACS analysis to evaluate the expression of stem cell-associated cell surface markers. The expression of differentiation-associated CD24 significantly increased in HMLE-Snail, HMLE-Twist, and SUM159 upon treatment with GSK3β inhibitors (BIO 1 μM, TWS119 2 μM, and LiCl 20 mM) relative to cells treated with DMSO (Fig. 3b and Additional file 6: Figure S4E and F). For example, following treatment with GSK3β inhibitors, the percentage of CD24-positive cells increased from about 6% to about 20–40% in HMLE-Snail cells, and in the case of HMLE-Twist cells from 3% to about 70%.

### Mesenchymal-like cells are more susceptible to GSK3β inhibitors than epithelial cells

**Fig. 4:**
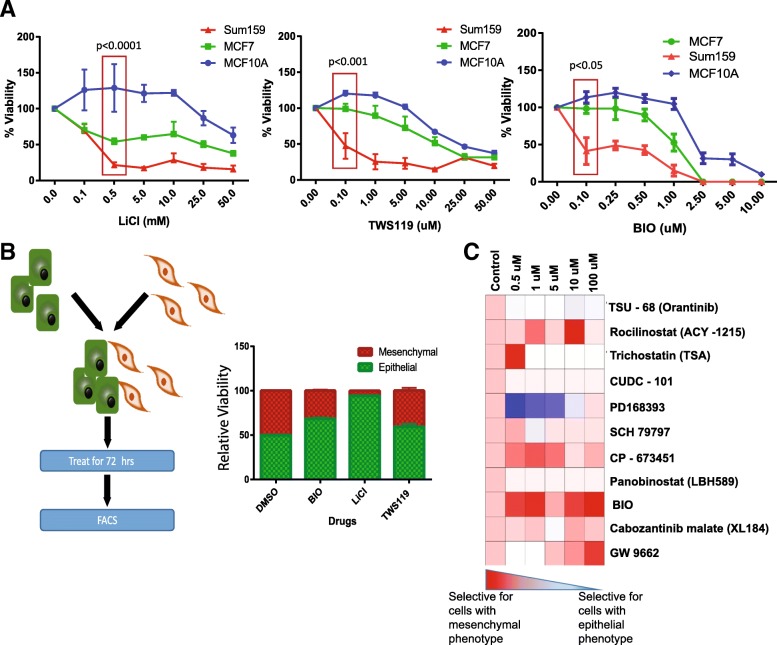
Mesenchymal-like cells are more sensitive to GSK3β inhibitors as compared to the epithelial cells. **a** MCF10A, a normal breast cell line, and MCF7 epithelial cells were more resistant to GSK3β inhibitors as compared to SUM159, a mesenchymal cell line. **b** A co-culture experiment was performed by mixing an equal proportion of green HMLER epithelial cells and red HMLER-Snail mesenchymal-like cells and treating them with GSK3β inhibitors. Following treatment with GSK3β inhibitors, the percentage of epithelial cells increased as compared to the percentage of mesenchymal cells. **c** HMLE and HMLE-Snail cells were treated with a dose range of the tested inhibitors, and viability was assessed by MTT assay and the heatmap summarizes the differential selectivity screen

To test if GSK3β inhibitors inhibit the growth of mesenchymal-like cells more efficiently than the growth of epithelial cancer cells, we exposed three representative cell lines to GSK3β inhibitors and evaluated viability using the MTT assay. These cell lines were MCF10A, representative of normal mammary glands; MCF7, a surrogate for the ER^+^/PR^+^ breast cancer cells; and Sum159, a TNBC cell line. The GSB3β inhibitors had a greater impact on the viability of the Sum159 cells than on the viability of the epithelial MCF7 or MCF10A cells (Fig. 4a). The IC50 for TWS119 in MCF10A and MCF7 cells was about 10 times higher than that for Sum159 cells, and similar observations were made for BIO and LiCl. To determine if this selectivity is unique to GSK3β inhibitors, we treated HMLE control cells and HMLE-Snail cells with a range of concentrations of all the 11 drugs identified in the initial screen for EMT inhibitors screen. We found that only GSK3β inhibitor BIO was capable of inhibiting EMT and CSC properties (Fig. 1b, Additional files 4 and 5: Figures S2 and S3). In addition, BIO selectively inhibits mesenchymal cells (Fig. 4b, Additional file 7: Figure S5).

In previous experiments, the epithelial and mesenchymal-like cell lines were analyzed separately. In a tumor, epithelial- and mesenchymal-like cells are found together. To test if GSK3β inhibitors can differentiate between cells with epithelial and mesenchymal phenotypes within the same culture system, we co-cultured epithelial HMLER cells that constitutively express GFP with the mesenchymal-like HMLER-Snail cells that express RFP. Equal numbers of these two cell types were mixed, cultured for 72 h, and then treated with BIO, TWS119, or LiCl. After 72 h, the proportion of RFP- to GFP-expressing cells was assessed using FACS. We observed an increase in the percentage of epithelial cells and a decrease in the proportion of mesenchymal-like cells with all GSK3β inhibitors tested (Fig. 4b). Additionally, HMLE-vector (epithelial) and HMLE-Snail (mesenchymal) cells were treated with 3 different concentrations of the 11 drugs that were selected from the screen. The viability of both cells following treatment was quantified using a MTT assay. The ratio of the viability of HMLE-vector cells to that of the HMLE-Snail cells was calculated and presented in the heatmap (Fig. 4c). Red represents more mesenchymal cell killing, and blue shows more epithelial cell killing. BIO was one of the drugs that scored as red indicating that it has a selective inhibitory effect on cells with mesenchymal properties as compared to the cells with an epithelial phenotype.

### *GSK3β* is overexpressed in tumors and correlates with poor survival of breast cancer patients

**Fig. 5:**
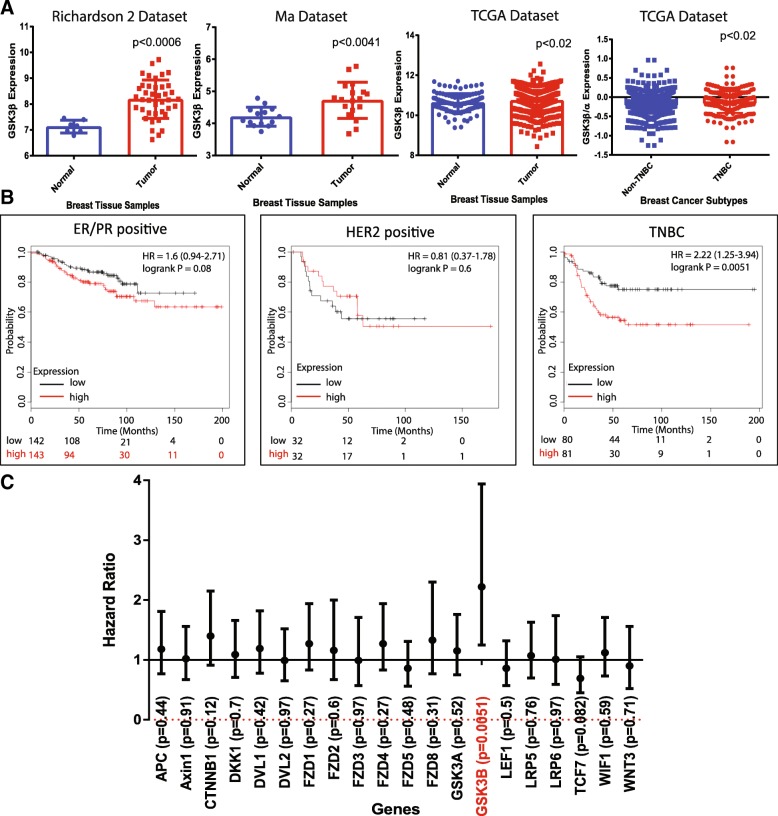
GSK3β is significantly upregulated in breast cancer. **a** GSK3β is significantly upregulated at the transcript levels in breast cancer tissues as compared to normal tissues in the Ma, Richardson, and TCGA datasets. **b** The KM plots generated using KMPlotter showed that higher expression of GSK3β correlates with worse survival only in TNBCs but not in the case of ER/PR-positive and HER2-positive breast cancers. GSK3β is the only signaling molecule in the Wnt signaling pathway that has a high hazard ratio and a significant *p* value. **c** Hazard ratios and the *p* values for several of the major players of the Wnt signaling pathway were generated and plotted for TNBC patients

To examine the clinical relevance of GSK3β, we analyzed Oncomine data and found *GSK3β* is significantly overexpressed in breast tumors in comparison with normal breast tissue in multiple datasets (Ma dataset [44], Richardson dataset 2 [45], and TCGA [46]). Of note are the cohorts described by Ma and Richardson in which GSK3β is significantly upregulated in tumor tissue as compared to the normal mammary gland (Fig. 5a) [47].

To examine the relation between GSK3β and patient survival, we analyzed patient survival using KmPlotter [48]. We also calculated hazard ratios to compare the survival of different groups of patients at a particular point in time. This analysis showed that *GSK3β* overexpression correlates with poor prognosis in patients diagnosed with TNBC (Fig. 5b). GSK3β was the only member of the Wnt pathway that was correlated with TNBC patient prognosis as demonstrated in Fig. 5c and Additional file 8: Figure S6. The correlation between *GSK3β* overexpression and survival was not significant in the case of patients with ER^+^/PR^+^ or HER2^+^ breast cancers. Additionally, using TCGA RPPA data, we also found that GSK3 expression is highly upregulated in TNBC cancers as compared to the non-TNBC tumors (Additional file 9: Figure S7).

## Discussion

TNBCs are aggressive breast cancers, and patients with TNBC have poor prognosis than those with other breast cancer subtypes. The presence of a high proportion of cells with mesenchymal and CSC characteristics results in a high metastatic potential. TNBCs have gene signatures similar to that of the cells that have undergone EMT [49]. EMT and CSC properties also underlie therapy resistance and tumor relapse. Some TNBCs respond to chemotherapy only to recur in a more aggressive and resistant form. Therefore, it is essential to discover the means of targeting the unique features that serve to drive TNBC tumor progression.

By the analysis of patient data, we found that high levels of expression of GSK3β correlated with poorer overall TNBC patient survival. GSK3β is a multifaceted kinase that is a key regulator of a number of cellular processes. GSK3β is a serine-threonine kinase that was originally discovered for its role in phosphorylating and inhibiting glycogen synthase [50]. However, GSK3β has been shown to play a central role in several cellular processes and participate in multiple different pathways [51, 52]. It is studied extensively due to its role in the Wnt/β-catenin signaling pathway.

GSK3β has been implicated in several diseases including neurological disorders and cancers [50, 51]. LiCl, a GSK3β inhibitor, has been used in the clinic for the treatment of psychiatric disorders for several decades [53]. Multiple roles have been suggested for GSK3β in different cancers, and its importance has been controversial [50]. We observed that GSK3β is upregulated in breast cancers versus normal mammary cells and that higher expression of GSK3β correlates with worse overall survival in TNBC patients. Inhibition of GSK3β results in a decrease in the expression of markers of the mesenchymal phenotype indicating its ability to impede the process of EMT. In addition to the increase of expression of mesenchymal markers as an indicator of EMT in cells, functional assays such as the wound healing assay have often been used to demonstrate the enhancement of the migratory potential of the cells which contribute to the highly aggressive and metastatic nature of the cells that have undergone EMT. GSK3β inhibitors also decrease the migratory potential of the cells that have a mesenchymal phenotype. However, GSK3β inhibitors have been previously demonstrated to have an inhibitory effect on the migratory properties of cells and several molecular mechanisms, such as their effect on Rho, ROCK, and Rac, which are known to regulate migration [54]. Data from this study suggest, in addition to the other mechanisms that might be at play, inhibition of EMT contributes to the detrimental effect of GSK3β inhibitors on the migratory properties of mesenchymal-like cells.

We and others have shown that the induction of EMT promotes stem-like properties of the cells, making them less differentiated and bestowing them with increased self-renewal potential [12, 43]. Our study shows that inhibition of GSK3β not only decreases the mesenchymal properties of the cells that have undergone EMT but significantly reduces the associated CSC properties. While exposing these mesenchyme-like breast cancer cells to GSK3β inhibitors increased CD24 expression, we did not see any change in the CD44 expression. We also observed a dramatic inhibition of sphere formation, suggesting that the GSK3β inhibitors target stem cell properties. This finding suggests that GSK3β inhibitors should be tested for efficacy in the treatment of TNBCs which have a high proportion of CSCs. Treatment with GSK3β inhibitors could prevent the emergence of chemotherapy resistance and tumor recurrence. Also, our data demonstrated that the GSK3β inhibitors are selective inhibitors of cells with mesenchymal and stem cell properties. Of note, not all mesenchyme-like cells have the same sensitivity to GSK3β inhibitors. T11 cells, a claudin-low mouse mammary tumor-derived primary line, only responded to BIO and TWS119 at high concentrations (Additional file 10: Figure S8). The current standard-of-care drugs are capable of eliminating the bulk of differentiated tumors. It is the stem-like cells with mesenchymal properties that are elusive. In our studies, we observed that GSK3β inhibitors are capable of inhibiting cells with mesenchymal and stem cell phenotypes. These findings suggest that using GSK3β inhibitors in combination or sequentially with the standard-of-care drugs might help in inhibiting both the differentiated and stem-like population of the tumors.

Our findings for the first time indicate that the inhibition of GSK3β negatively affects the cells with mesenchymal phenotype as compared to their epithelial counterparts. Currently, there is a paucity of methods of inhibiting cells with a mesenchymal phenotype. On their own, GSK3β inhibitors appear to have limited toxicity. In fact, GSK3β inhibitors such as LiCl have been safely used for the treatment of neuronal disorders, and hence, its effects and side effects have been well studied [55]. In the context of the Wnt signaling pathway, GSK3β is a negative regulator, and therefore, inhibition of GSK3β would mimic the activation of the Wnt signaling pathway. This often leads to the speculation that inhibition of GSK3β could stimulate tumorigenesis by promoting the CSCs. However, studies have shown that there is no association between the chronic use of LiCl and the occurrence of tumors in patients [56, 57].

Additionally, we emphasize the fact that GSK3β is a multifaceted kinase which is involved in several signaling pathways in addition to the Wnt signaling pathway. Activation of Wnt signaling is often correlated with the presence of stem cell properties which in turn indicates a poor prognosis, chemotherapy resistance, and consequent relapse of the disease [22, 58]. GSK3β is considered to be a tumor suppressor due to its ability to inhibit the Wnt-β-catenin pathway. In contrast, we observed that inhibition of GSK3β inhibited stem cell attributes instead of activating stem cell properties. We believe this conundrum is due to the multifaceted nature of GSK3β. For example, GSK3β is known to contribute to cell cycle, cell metabolism, differentiation, apoptosis, etc. Therefore, although this ubiquitous and promiscuous kinase is constitutively expressed, its function is heavily regulated in the cells. One of the primary modes of regulation is the substrate specificity and availability [59]. For example, GSK3β binds and phosphorylates primed substrates which are previously phosphorylated by other kinases. The Ser9 residue of GSK3β when phosphorylated binds to the site in which the primed phosphorylated residue on GSK3β substrate binds and inactivates the kinase. However, as the concentration of the primed substrate increases, the phosphorylated Ser9 residue of GSK3β is competitively displaced rendering the kinase active to phosphorylate the primed kinase [59, 60]. Secondly, GSK3β is present in different subcellular compartments such as the mitochondria, nucleus, and cytoplasm, and there is a constant flux between these compartments [59]. For example, the GSK3β associated with the destruction complex of the Wnt signaling pathway is sequestered in endosomes, and therefore, changes in this compartment can occur independently of GSK3β function in the nucleus or the cytoplasm [61]. This compartmentalization of GSK3β allows it to function in multiple different pathways. Thus, the effect of GSK3β inhibition on a cell is dependent on the cumulative effect of this inhibition on all the different cellular signaling pathways regulated by GSK3β. Therefore, its role in cell biology and thereby in tumor progression is entirely dependent on the cell context, and it is essential to study GSK3β as a central player regulating the fate of the cell rather than merely as a regulator of the Wnt signaling pathway.

Finally, GSK3β expression is increased in breast cancer correlating with a worse prognosis for TNBC patients. Another study based on immunohistochemistry done on TMAs of about 1600 patients supports this observation [62]. A clear correlation was demonstrated between the upregulation of GSK3β and worse survival rates for patients [62]. This suggests that the analysis of GSK3β expression may serve as a novel biomarker for predicting poor clinical outcome. Based on our findings, we propose that GSK3β inhibitors are promising candidates for combining with standard-of-care chemotherapy for the treatment of a subset of TNBCs, in particular for claudin-low subtypes to prevent their progression, emergence of chemoresistance, and tumor recurrence. This should be further validated by in vivo studies using patient-derived xenograft and genetically engineered preclinical mouse models.

## Conclusion

Triple-negative breast cancers are one of the most aggressive breast cancers with a high proportion of cells with mesenchymal and stem cell properties that currently lack targeted therapies. To treat patients with TNBCs, it is essential to identify druggable targets. Chemotherapeutic drugs are highly efficient causing tumor attrition but are unable to eliminate cancer stem cells which are predominant in TNBCs. Therefore, identification of targets that can be inhibited to reduce EMT and the associated stem cell properties may improve the prognosis of TNBC patients. We identified GSK3β as one such target that is highly upregulated in breast cancer patients, and this upregulation correlates with poor prognosis. GSK3β inhibitors were one of the few small molecule inhibitors that were capable of inhibiting EMT. Additionally, we also observed that inhibition of GSK3β inhibits EMT and CSC properties and migratory properties and is capable of selectively inhibiting cells with mesenchymal properties thereby serving as an ideal target to target in TNBCs.

## Additional files

Additional file 1: Data S1. List of drugs from Selleckchem used in the high-throughput screen; chemical data and their arrangement in the plates used for the screen are listed. (XLSX 446 kb)

Additional file 2: Data S2. Summary of results from the high-throughput screen and the detailed red/green readout for each drug tested in the screen. (PDF 3424 kb)

Additional file 3: **Figure S1.** The IC50 of the 3 drugs were calculated for the 3 mesenchymal-like cell lines used in this study. (PDF 122 kb)

Additional file 4: **Figure S2.** The drugs that were selected from the screen were validated using FACS. The MDA MB 231 reporter cells were treated with all the 11 drugs selected from the screen. Following treatment, the percentage of cells fluorescing red and green were assessed using flow cytometry and plotted for each of the three concentrations to generate the graphs. (PDF 160 kb)

Additional file 5: **Figure S3.** GSK3β inhibitor, BIO, is one of the drugs that is capable of inhibiting the sphere forming ability of mesenchymal MDA-MB-231 cells. The drugs that were selected from the screen were used to treat the mammosphere assay. MDA MB 231 reporter cells were grown in ultra-low attachments plates in mammosphere media for 10 days. The number of mammospheres was counted and graphed, and BIO was one of the drugs that decreased the sphere forming ability of the reporter MDA MB 231 cells. The heatmap summarizes the mammosphere data showing that BIO is one of the drugs that decreases the sphere-forming ability of the MDA MB 231 reporter cells. (PDF 141 kb)

Additional file 6: **Figure S4.** Genetic suppression of GSK3β expression decreases the sphere-forming potential of mesenchymal-like cells. (A) Cells with mesenchymal properties were treated with the 3 GSK3β inhibitors for 24 h. Following the treatment, the cells were plated for mammosphere assays and (B) a growth curve was generated to ensure that decrease in proliferation is not the reason for the decreased sphere forming ability of these cells. (C) Knockdown of GSK3β decreases the mammosphere forming capability of the mesenchymal cells. HMLE Snail, HMLE Twist, and Sum159 cells were stably transfected with GSK3β shRNA and grown in ultra-low attachments plates in mammosphere media for 10 days. (D). Mouse embryonic fibroblasts (MEFs) in which GSK3β were knocked out were grown in ultra-low attachment plates in mammosphere media for 10 days. Mouse embryonic fibroblasts (MEFs) in which GSK3β was knocked out were grown for 4 days, and growth was assessed on days 2, 3, and 4. Knocking out of GSK3β in MEFs reduces the sphere forming potential of the MEFs. The cells with mesenchymal properties were treated with 3 GSK3β inhibitors and the change in the CD24/44 profile of these cells following treatment was quantified and represented as a (E) table and (F) bar graph. (PDF 153 kb)

Additional file 7: **Figure S5.** HMLE-vector and HMLE-Snail cells were treated with a dose range of the tested inhibitors, and viability was assessed by MTT assay. Of the drugs that were shortlisted from the screen, BIO was one of the drugs that could selectively inhibit HMLE-Snail cells with mesenchymal phenotype more efficiently as compared to HMLE-vector cells with epithelial phenotype. (PDF 133 kb)

Additional file 8: **Figure S6. **KmPlots were generated for several major players of the Wnt signaling pathway using the KmPlotter. Of all the different players, GSK3β was the only gene, the upregulation of which significantly correlated with worse survival in TNBCs. (PDF 322 kb)

Additional file 9: **Figure S7. **TCGA RPPA data was mined to compare the expression of GSK3β in TNBCs and other types of breast cancer. The analysis of these data revealed a significant increase in the expression of GSK3 in TNBCs as compared to the other types of breast cancer. (PDF 139 kb)

Additional file 10: **Figure S8.** Claudin-low T11 cells were grown in ultra-low attachment plates in mammosphere media for 10 days in the presence of 3 GSK3β inhibitors. The numbers of mammospheres were counted and graphed (*n* = 3, *p* values were calculated using Student’s unpaired two-tailed *t* test). (PDF 94 kb)

## Abbreviations

APC: Allophycocyanin
cDNA: Complementary deoxyribonucleic acid
CSC: Cancer stem cells
EMT: Epithelial-mesenchymal transition
ER: Estrogen receptor
FACS: Fluorescence-activated cell sorting
GFP: Green fluorescent protein
GSK3β: Glycogen synthase kinase 3 beta
HER2: Human epidermal growth factor receptor 2
HMLE: Human mammary epithelial cells
HMLER: Human mammary epithelial cells with V12 H-Ras
KM plot: Kaplan-Meier plot
LiCl: Lithium chloride
PAGE: Polyacrylamide gel electrophoresis
PE: Phycoerythrin
PR: Progesterone receptor
qRT-PCR: Quantitative reverse transcription polymerase chain reaction
RFP: Red fluorescent protein
RIPA buffer: Radio-immunoprecipitation buffer
TNBC: Triple-negative breast cancer
