# Supplementary material for: GSK3β regulates epithelial-mesenchymal transition and cancer stem cell properties in triple-negative breast cancer
Source: Breast Cancer Res. 2019 Mar 7;21:37. doi: 10.1186/s13058-019-1125-0 (PMC6407242; doi:10.1186/s13058-019-1125-0)
Supplement: Supplementary file 2 — Data S2. Summary of results from the high-throughput screen and the detailed red/green readout for each drug tested in the screen. (PDF 3424 kb) [file 13058_2019_1125_MOESM2_ESM.pdf]

Selleck 01 (Replicate A)

Percent Green Cells	Percent Red Cells
10 uM 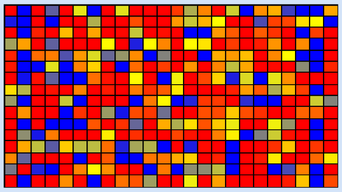	10 uM 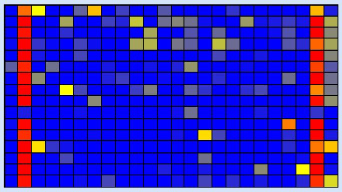
1 uM 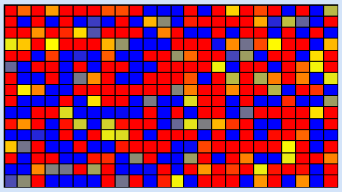	1 uM 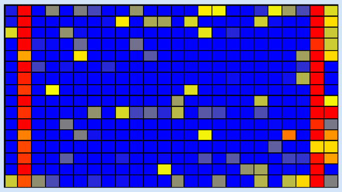
0.1 uM 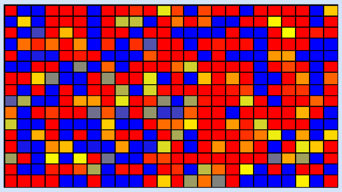	0.1 uM 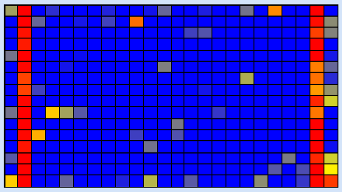

Selleck 01 (Replicate B)

Percent Green Cells	Percent Red Cells
10 uM 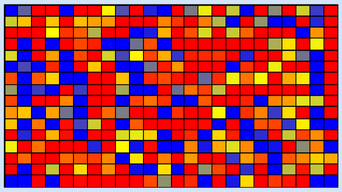	10 uM 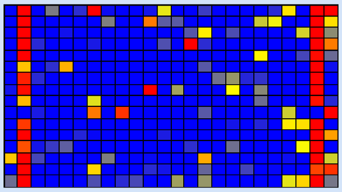
1 uM 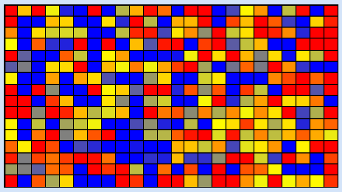	1 uM 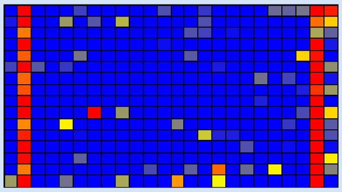
0.1 uM 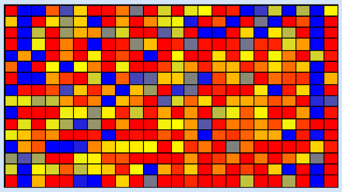	0.1 uM 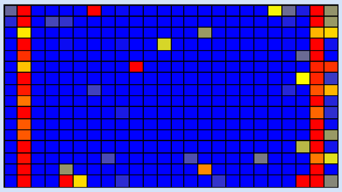

Selleck 02 (Replicate A)

Percent Green Cells	Percent Red Cells
10 uM 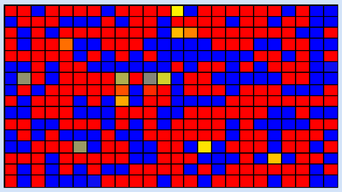	10 uM 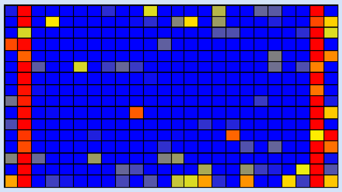
1 uM 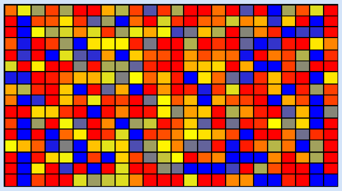	1 uM 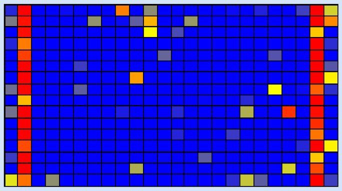
0.1 uM 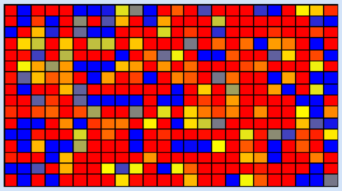	0.1 uM 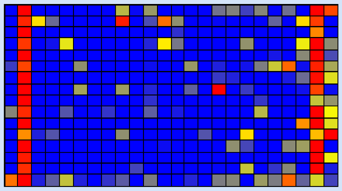

Selleck 02 (Replicate B)

Percent Green Cells	Percent Red Cells
10 uM 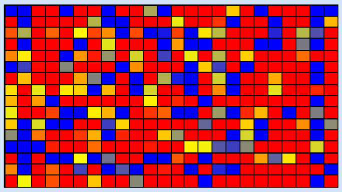	10 uM 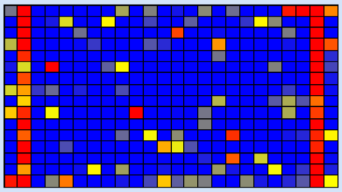
1 uM 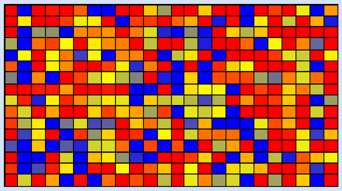	1 uM 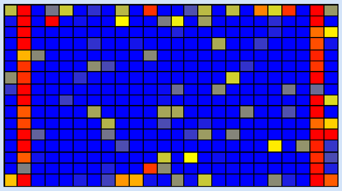
0.1 uM 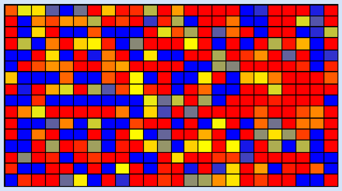	0.1 uM 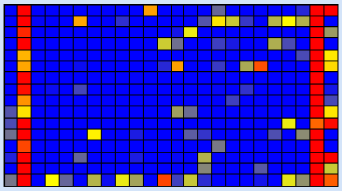

Selleck 03 (Replicate A)

Selleck 03 (Replicate B)

Percent Green Cells | Percent Red Cells

10 uM
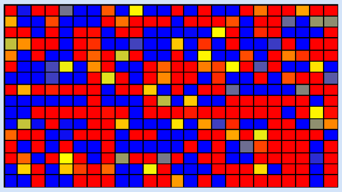
10 uM
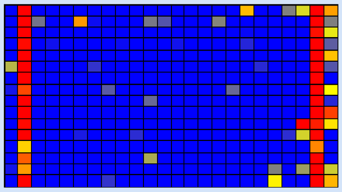

1 uM
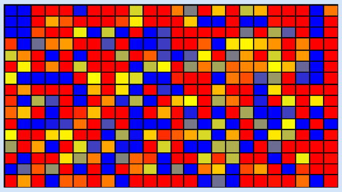
1 uM
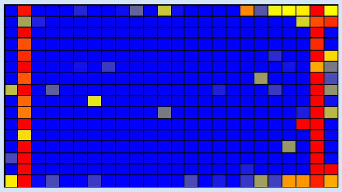

0.1 uM
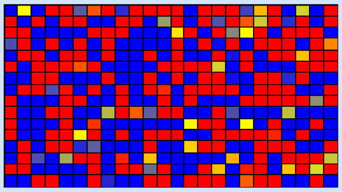
0.1 uM
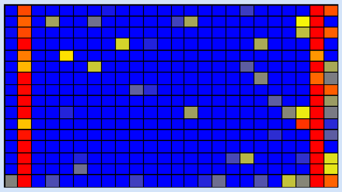

Percent Green Cells | Percent Red Cells

10 uM
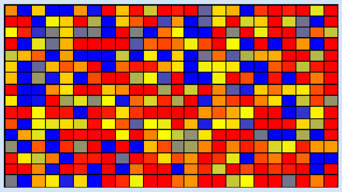
10 uM
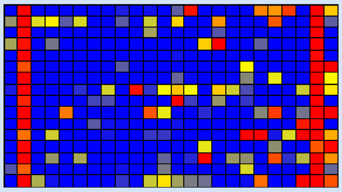

1 uM
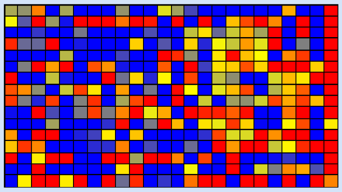
1 uM
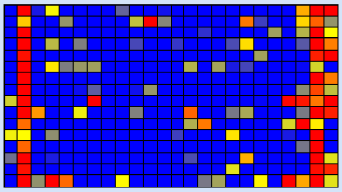

0.1 uM
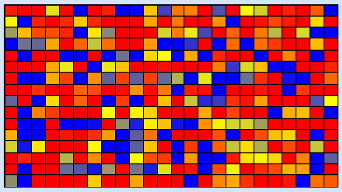
0.1 uM
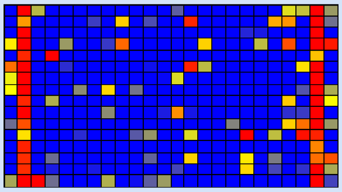

Selleck 04 (Replicate A)

Percent Green Cells | Percent Red Cells

10 uM
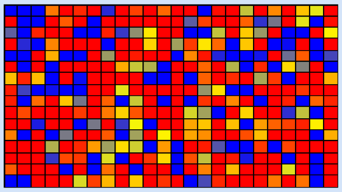
10 uM
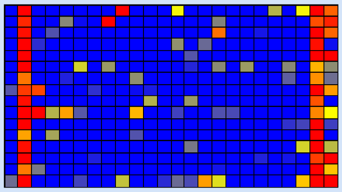

1 uM
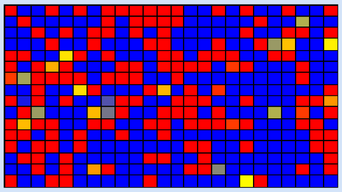
1 uM
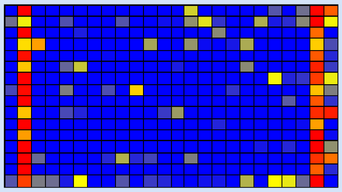

0.1 uM
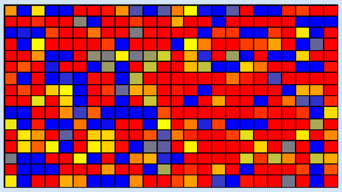
0.1 uM
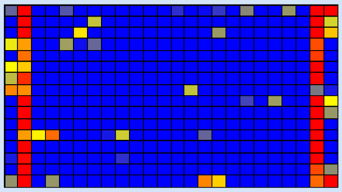

Selleck 04 (Replicate B)

Percent Green Cells | Percent Red Cells

10 uM
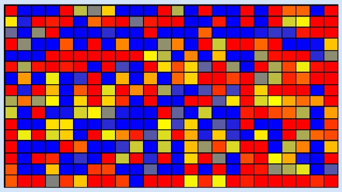
10 uM
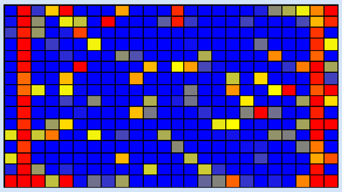

1 uM
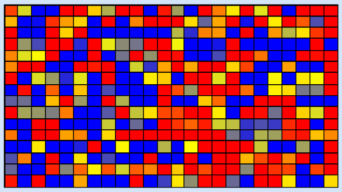
1 uM
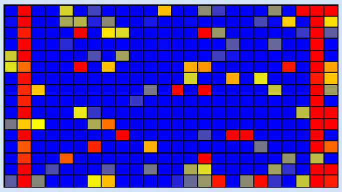

0.1 uM
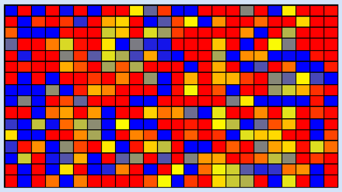
0.1 uM
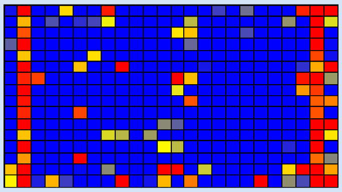

Compound	Normalized Values (A and B Sets)	Responses

(+)-JQ1

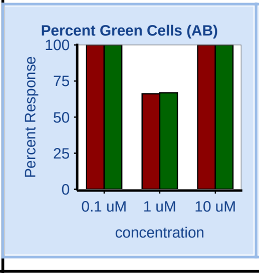

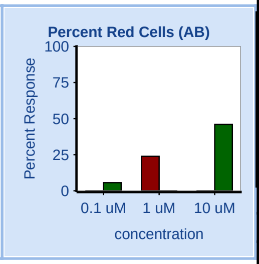

Average Response

Dose	Green Signal	Actual Pct Green Cells	Normalized Pct Green Cells	Red Signal	Actual Pct Red Cells	Normalized Pct Red Cells
0.1 uM	105.55	0.91	121.74	92.28	0.09	-15.20
1 uM	104.84	0.81	66.52	105.09	0.14	5.37
10 uM	110.68	0.91	121.48	110.60	0.16	22.19

Compound	Normalized Values (A and B Sets)	Responses

(+)-Matrine

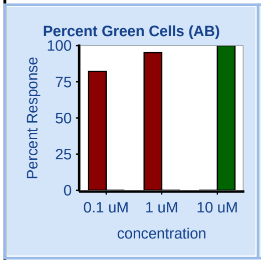

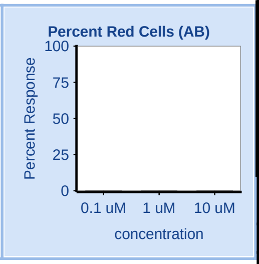

Average Response

Dose	Green Signal	Actual Pct Green Cells	Normalized Pct Green Cells	Red Signal	Actual Pct Red Cells	Normalized Pct Red Cells
0.1 uM	64.09	0.55	-282.88	70.34	0.00	-53.50
1 uM	92.16	0.81	46.80	85.27	0.06	-31.22
10 uM	93.24	0.89	2.15	67.27	0.01	-52.43

Compound	Normalized Values (A and B Sets)	Responses

(+)-Usniacin

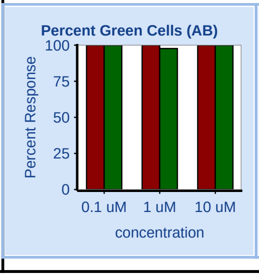

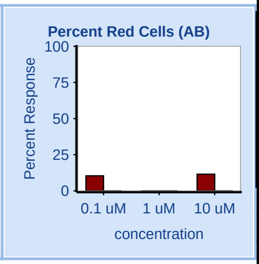

Average Response

Dose	Green Signal	Actual Pct Green Cells	Normalized Pct Green Cells	Red Signal	Actual Pct Red Cells	Normalized Pct Red Cells
0.1 uM	109.91	0.94	150.76	107.19	0.12	2.66
1 uM	113.16	0.93	116.02	84.71	0.06	-29.83
10 uM	111.48	0.94	241.09	99.31	0.10	-12.35

Compound	Normalized Values (A and B Sets)	Responses

(-)-Blebbistatin

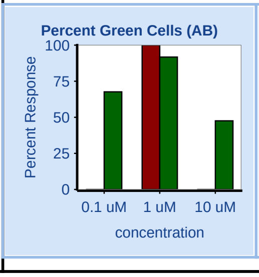

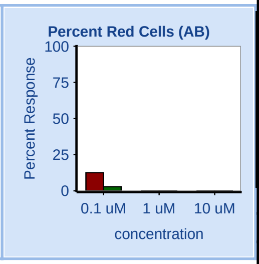

Average Response

Dose	Green Signal	Actual Pct Green Cells	Normalized Pct Green Cells	Red Signal	Actual Pct Red Cells	Normalized Pct Red Cells
0.1 uM	97.79	0.85	-11.32	97.68	0.14	7.69
1 uM	99.85	0.89	123.67	61.42	0.03	-51.94
10 uM	91.91	0.77	-13.51	89.46	0.08	-20.87

Compound	Normalized Values (A and B Sets)	Responses

(-)-Parthenolide

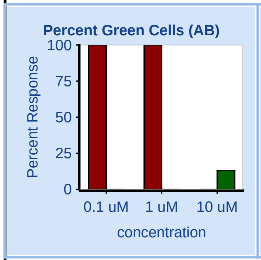

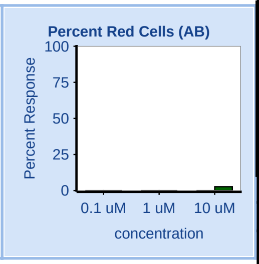

Average Response

Dose	Green Signal	Actual Pct Green Cells	Normalized Pct Green Cells	Red Signal	Actual Pct Red Cells	Normalized Pct Red Cells
0.1 uM	84.30	0.74	-74.80	86.93	0.06	-27.69
1 uM	96.57	0.82	53.34	79.48	0.03	-40.97
10 uM	88.96	0.79	-607.00	95.49	0.10	-9.31

Compound	Normalized Values (A and B Sets)	Responses

1,4PBITU duhydrobromide

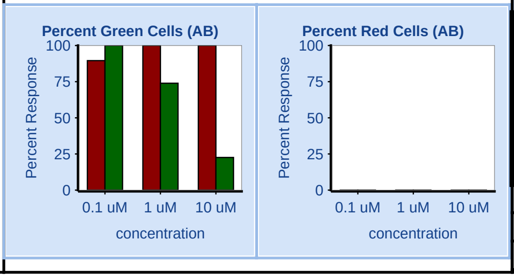

Average Response

Dose	Green Signal	Actual Pct Green Cells	Normalized Pct Green Cells	Red Signal	Actual Pct Red Cells	Normalized Pct Red Cells
0.1 uM	96.25	0.89	96.49	84.28	0.05	-41.84
1 uM	102.49	0.91	266.19	55.38	0.01	-42.85
10 uM	107.11	0.90	86.46	64.82	0.02	-40.96

Compound	Normalized Values (A and B Sets)	Responses

1-Hexadecanol

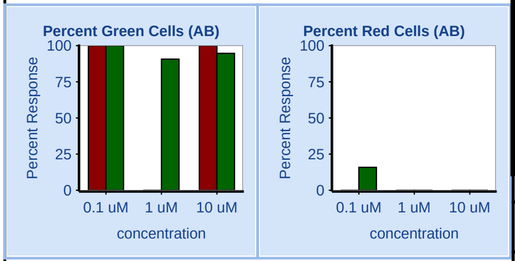

Average Response

Dose	Green Signal	Actual Pct Green Cells	Normalized Pct Green Cells	Red Signal	Actual Pct Red Cells	Normalized Pct Red Cells
0.1 uM	100.31	0.90	108.70	94.72	0.09	-22.39
1 uM	90.32	0.81	-443.80	89.90	0.08	-9.33
10 uM	108.15	0.93	115.10	89.40	0.09	-10.78

Compound	Normalized Values (A and B Sets)	Responses

2-Methoxyestradiol (2-MeOE2)

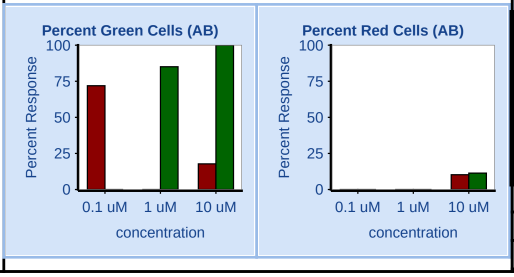

Average Response

Dose	Green Signal	Actual Pct Green Cells	Normalized Pct Green Cells	Red Signal	Actual Pct Red Cells	Normalized Pct Red Cells
0.1 uM	74.87	0.61	-71.04	69.72	0.02	-63.87
1 uM	96.86	0.79	-64.96	89.72	0.06	-29.55
10 uM	115.96	0.84	84.01	120.78	0.15	10.81

Compound	Normalized Values (A and B Sets)	Responses

2-Thiouracil

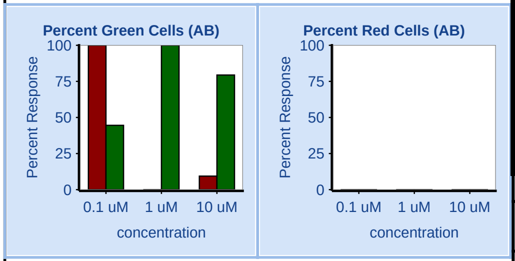

Average Response

Dose	Green Signal	Actual Pct Green Cells	Normalized Pct Green Cells	Red Signal	Actual Pct Red Cells	Normalized Pct Red Cells
0.1 uM	95.54	0.86	73.42	80.10	0.03	-57.81
1 uM	95.11	0.87	-222.75	71.31	0.03	-38.30
10 uM	92.38	0.86	44.40	81.59	0.06	-26.38

Compound	Normalized Values (A and B Sets)	Responses

3-Deazaneplanocin A (DZNeP)

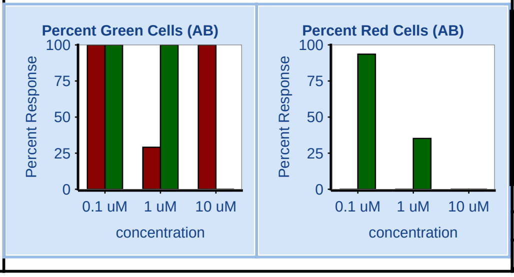

Average Response

Dose	Green Signal	Actual Pct Green Cells	Normalized Pct Green Cells	Red Signal	Actual Pct Red Cells	Normalized Pct Red Cells
0.1 uM	111.18	0.94	215.79	112.43	0.20	38.42
1 uM	105.58	0.82	75.30	96.59	0.12	-6.57
10 uM	90.94	0.72	43.36	84.92	0.07	-27.71

Compound | Normalized Values (A and B Sets) | Responses

3-Indolebutyric acid (IBA)

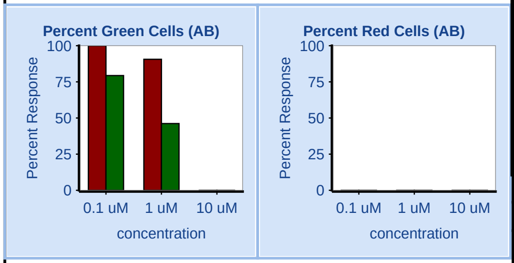

Average Response

Dose	Green Signal	Actual Pct Green Cells	Normalized Pct Green Cells	Red Signal	Actual Pct Red Cells	Normalized Pct Red Cells
0.1 uM	104.94	0.91	113.83	76.11	0.02	-45.01
1 uM	95.98	0.84	68.38	63.75	0.01	-52.13
10 uM	85.49	0.80	-478.46	99.98	0.08	-19.77

Compound | Normalized Values (A and B Sets) | Responses

3-Methyladenine

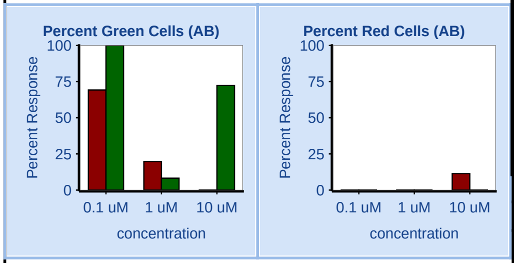

Average Response

Dose	Green Signal	Actual Pct Green Cells	Normalized Pct Green Cells	Red Signal	Actual Pct Red Cells	Normalized Pct Red Cells
0.1 uM	98.83	0.89	100.69	77.14	0.04	-37.52
1 uM	88.52	0.73	14.00	66.06	0.02	-48.26
10 uM	86.43	0.79	-728.27	92.32	0.08	-20.28

Compound | Normalized Values (A and B Sets) | Responses

4-Phenylbutyrate

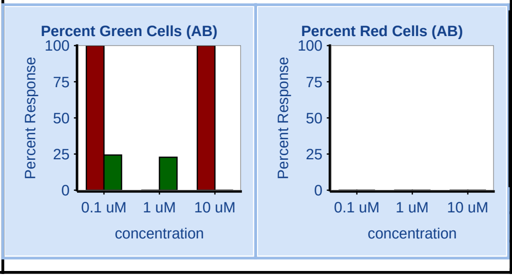

Average Response

Dose	Green Signal	Actual Pct Green Cells	Normalized Pct Green Cells	Red Signal	Actual Pct Red Cells	Normalized Pct Red Cells
0.1 uM	100.99	0.88	92.81	82.11	0.03	-56.66
1 uM	95.14	0.85	10.67	66.88	0.02	-39.64
10 uM	95.56	0.87	55.38	72.55	0.03	-37.22

Compound | Normalized Values (A and B Sets) | Responses

5-hydroxymethyl Tolterodine (PNU 200577, 5-HMT, 5-HM)

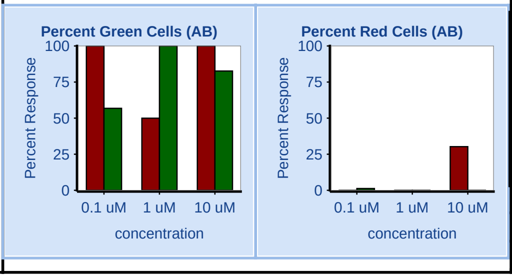

Average Response

Dose	Green Signal	Actual Pct Green Cells	Normalized Pct Green Cells	Red Signal	Actual Pct Red Cells	Normalized Pct Red Cells
0.1 uM	102.98	0.91	107.32	96.74	0.10	-8.78
1 uM	97.93	0.84	75.07	82.78	0.03	-40.31
10 uM	103.51	0.93	375.93	96.88	0.10	-9.94

Compound | Normalized Values (A and B Sets) | Responses

5-hydroxytryptophan (5-HTP)

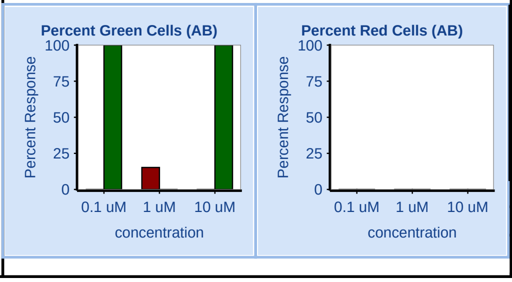

Average Response

Dose	Green Signal	Actual Pct Green Cells	Normalized Pct Green Cells	Red Signal	Actual Pct Red Cells	Normalized Pct Red Cells
0.1 uM	84.25	0.76	-23.35	88.90	0.06	-24.83
1 uM	83.10	0.71	3.71	81.15	0.03	-41.97
10 uM	92.16	0.88		82.92	0.03	-42.08

Compound | Normalized Values (A and B Sets) | Responses

			-169.23			

Compound | Normalized Values (A and B Sets) | Responses

10-Deacetylbaccatin-III

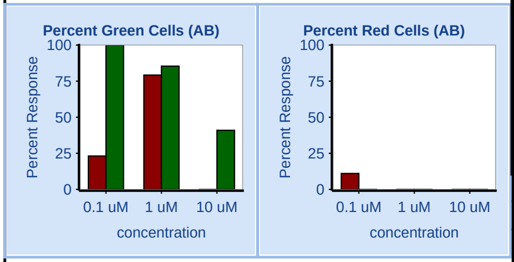

Average Response

Dose	Green Signal	Actual Pct Green Cells	Normalized Pct Green Cells	Red Signal	Actual Pct Red Cells	Normalized Pct Red Cells
0.1 uM	96.25	0.86	64.63	92.14	0.08	-20.34
1 uM	103.73	0.86	82.31	84.14	0.05	-35.75
10 uM	88.81	0.84	-248.65	91.59	0.02	-47.13

Compound | Normalized Values (A and B Sets) | Responses

17-AAG (Tanespimycin)

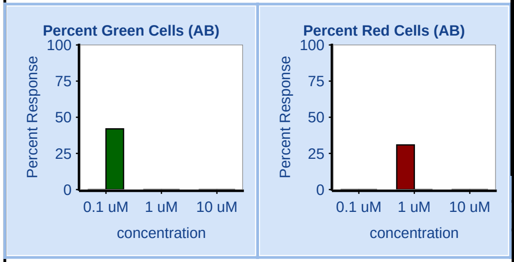

Average Response

Dose	Green Signal	Actual Pct Green Cells	Normalized Pct Green Cells	Red Signal	Actual Pct Red Cells	Normalized Pct Red Cells
0.1 uM	89.10	0.80	-0.41	84.63	0.05	-49.57
1 uM	91.91	0.73	-50.55	109.19	0.14	5.40
10 uM	70.59	0.51	-152.95	102.62	0.09	-13.00

Compound | Normalized Values (A and B Sets) | Responses

17-DMAG (Alvespimycin) HCl

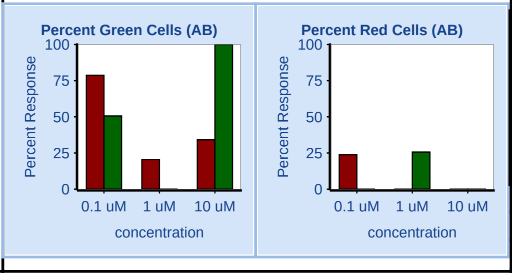

Average Response

Dose	Green Signal	Actual Pct Green Cells	Normalized Pct Green Cells	Red Signal	Actual Pct Red Cells	Normalized Pct Red Cells
0.1 uM	106.60	0.85	64.71	99.10	0.11	-20.45
1 uM	96.48	0.75	-17.31	112.03	0.15	10.77
10 uM	96.37	0.82	68.41	77.23	0.03	-39.00

Compound | Normalized Values (A and B Sets) | Responses

20-Hydroxyecdysone

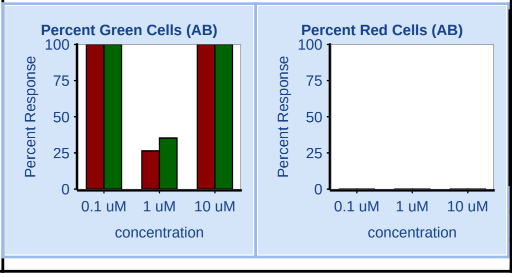

Average Response

Dose	Green Signal	Actual Pct Green Cells	Normalized Pct Green Cells	Red Signal	Actual Pct Red Cells	Normalized Pct Red Cells
0.1 uM	107.67	0.93	136.54	90.20	0.07	-20.60
1 uM	88.60	0.76	30.86	83.71	0.03	-40.64
10 uM	105.27	0.94	238.11	102.36	0.08	-21.42

Compound | Normalized Values (A and B Sets) | Responses

A-205804

Average Response

Dose	Green Signal			Red Signal	Actual Pct Red Cells	

Compound | Normalized Values (A and B Sets) | Responses

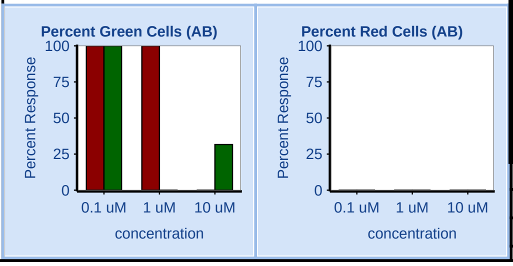

		Actual Pct Green Cells	Normalized Pct Green Cells			Normalized Pct Red Cells
0.1 uM	94.69	0.91	130.36	70.60	0.03	-50.84
1 uM	95.45	0.81	59.69	68.28	0.02	-56.22
10 uM	83.93	0.73	-45.71	70.41	0.02	-54.38

Compound | Normalized Values (A and B Sets) | Responses

A-674563

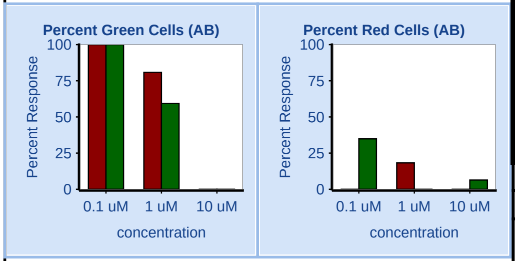

Average Response

Dose	Green Signal	Actual Pct Green Cells	Normalized Pct Green Cells	Red Signal	Actual Pct Red Cells	Normalized Pct Red Cells
0.1 uM	106.62	0.91	119.00	87.60	0.10	-4.60
1 uM	106.08	0.84	70.10	99.30	0.12	-1.84
10 uM	76.84	0.52	-2501.47	82.96	0.10	-12.94

Compound | Normalized Values (A and B Sets) | Responses

A-803467

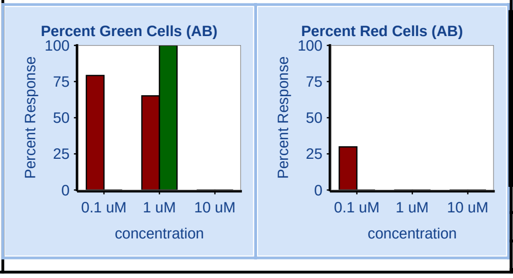

Average Response

Dose	Green Signal	Actual Pct Green Cells	Normalized Pct Green Cells	Red Signal	Actual Pct Red Cells	Normalized Pct Red Cells
0.1 uM	88.04	0.79	-10.30	92.68	0.10	-9.44
1 uM	104.82	0.88	97.08	84.56	0.05	-33.49
10 uM	84.90	0.82	-85.38	94.46	0.07	-25.58

Compound | Normalized Values (A and B Sets) | Responses

A-966492

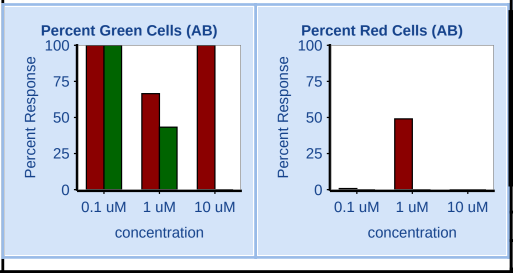

Average Response

Dose	Green Signal	Actual Pct Green Cells	Normalized Pct Green Cells	Red Signal	Actual Pct Red Cells	Normalized Pct Red Cells
0.1 uM	98.61	0.91	113.93	84.15	0.06	-27.23
1 uM	93.97	0.81	54.89	107.49	0.14	4.60
10 uM	93.82	0.83	28.17	96.12	0.07	-24.35

Compound | Normalized Values (A and B Sets) | Responses

A66

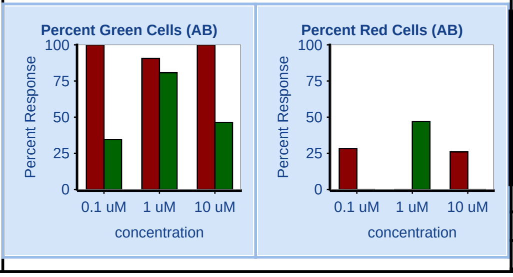

Average Response

Dose	Green Signal	Actual Pct Green Cells	Normalized Pct Green Cells	Red Signal	Actual Pct Red Cells	Normalized Pct Red Cells
0.1 uM	103.14	0.90	99.54	90.75	0.11	-8.14
1 uM	100.54	0.87	85.63	102.36	0.13	1.75
10 uM	98.47	0.90	228.90	104.75	0.11	-4.08

Compound | Normalized Values (A and B Sets) | Responses

A922500

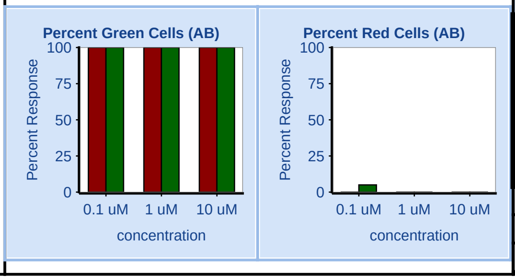

Average Response						
Dose	Green Signal	Actual Pct Green Cells	Normalized Pct Green Cells	Red Signal	Actual Pct Red Cells	Normalized Pct Red Cells
0.1 uM	113.68	0.96	166.06	92.37	0.09	-12.83
1 uM	110.04	0.92	114.39	77.73	0.04	-38.53
10 uM	108.15	0.94	326.92	84.35	0.04	-41.20

Compound | Normalized Values (A and B Sets) | Responses

Abiraterone Acetate

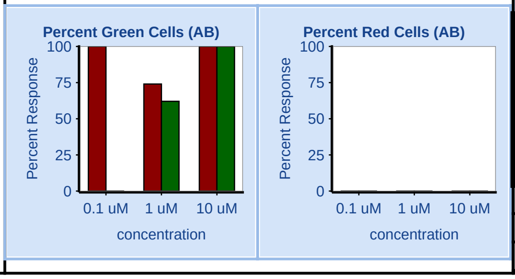

Average Response						
Dose	Green Signal	Actual Pct Green Cells	Normalized Pct Green Cells	Red Signal	Actual Pct Red Cells	Normalized Pct Red Cells
0.1 uM	92.72	0.84	40.09	77.88	0.03	-42.90
1 uM	100.31	0.83	68.07	99.51	0.10	-13.53
10 uM	104.34	0.93	302.50	99.70	0.08	-18.46

Compound | Normalized Values (A and B Sets) | Responses

ABT-199 (GDC-0199)

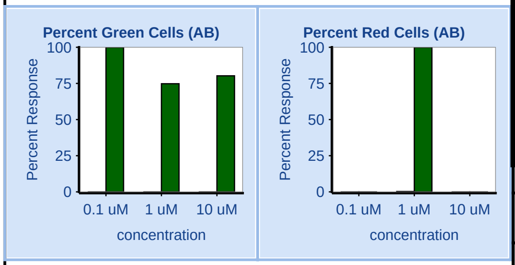

Average Response						
Dose	Green Signal	Actual Pct Green Cells	Normalized Pct Green Cells	Red Signal	Actual Pct Red Cells	Normalized Pct Red Cells
0.1 uM	96.37	0.85	56.49	92.52	0.05	-42.17
1 uM	91.34	0.83	-271.66	111.84	0.19	54.40
10 uM	78.34	0.64	-173.76	80.97	0.05	-28.82

Compound | Normalized Values (A and B Sets) | Responses

ABT-751 (E7010)

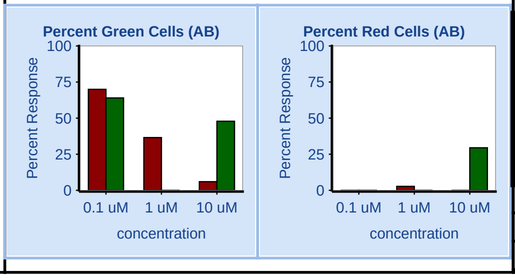

Average Response						
Dose	Green Signal	Actual Pct Green Cells	Normalized Pct Green Cells	Red Signal	Actual Pct Red Cells	Normalized Pct Red Cells
0.1 uM	97.60	0.86	66.97	82.10	0.03	-55.93
1 uM	103.79	0.79	12.99	104.53	0.10	-9.93
10 uM	107.19	0.76	26.91	108.71	0.15	13.59

Compound | Normalized Values (A and B Sets) | Responses

Acadesine

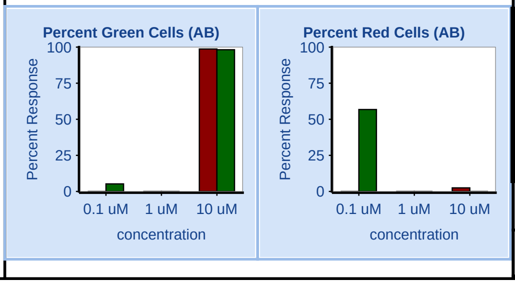

Average Response						
Dose	Green Signal	Actual Pct Green Cells	Normalized Pct Green Cells	Red Signal	Actual Pct Red Cells	Normalized Pct Red Cells
0.1 uM	74.80	0.63	-192.83	109.49	0.14	7.64
1 uM	73.99	0.59		92.84	0.06	-29.15

Compound	Normalized Values (A and B Sets)	Responses

			-193.47			
10 uM	103.15	0.87	98.45	115.00	0.11	-9.39

Compound	Normalized Values (A and B Sets)	Responses

Acesulfame Potassium

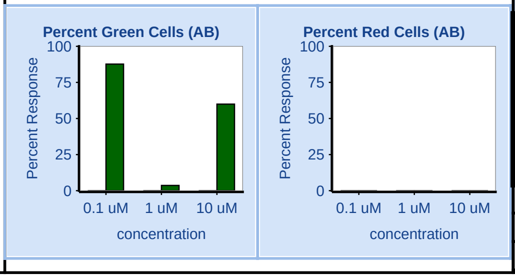

Average Response

Dose	Green Signal	Actual Pct Green Cells	Normalized Pct Green Cells	Red Signal	Actual Pct Red Cells	Normalized Pct Red Cells
0.1 uM	92.72	0.85	-67.41	64.72	0.02	-54.43
1 uM	84.61	0.72	-1.36	66.82	0.01	-60.77
10 uM	90.15	0.76	-30.53	81.01	0.04	-45.40

Compound	Normalized Values (A and B Sets)	Responses

Aclidinium Bromide

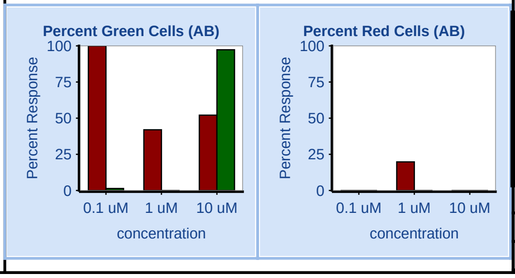

Average Response

Dose	Green Signal	Actual Pct Green Cells	Normalized Pct Green Cells	Red Signal	Actual Pct Red Cells	Normalized Pct Red Cells
0.1 uM	89.59	0.84	117.19	85.10	0.07	-27.30
1 uM	86.17	0.73	5.50	96.39	0.10	-14.66
10 uM	95.73	0.86	74.70	93.13	0.07	-26.95

Compound	Normalized Values (A and B Sets)	Responses

Adapalene

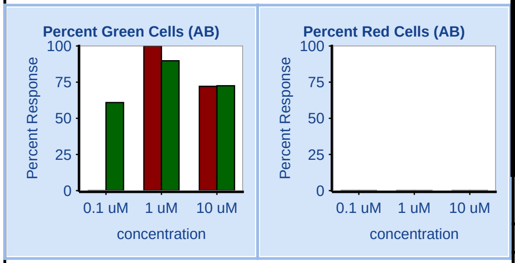

Average Response

Dose	Green Signal	Actual Pct Green Cells	Normalized Pct Green Cells	Red Signal	Actual Pct Red Cells	Normalized Pct Red Cells
0.1 uM	75.48	0.60	-272.51	82.58	0.03	-56.79
1 uM	104.68	0.91	130.48	102.14	0.07	-24.81
10 uM	102.06	0.84	72.33	76.82	0.02	-43.36

Compound	Normalized Values (A and B Sets)	Responses

Adefovir Dipivoxil

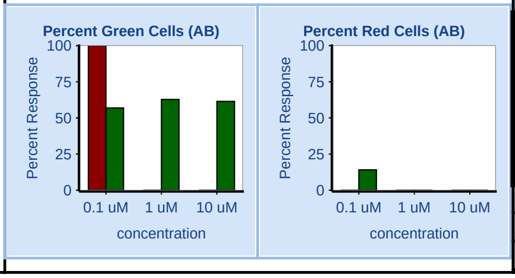

Average Response

Dose	Green Signal	Actual Pct Green Cells	Normalized Pct Green Cells	Red Signal	Actual Pct Red Cells	Normalized Pct Red Cells
0.1 uM	104.57	0.89	105.97	98.09	0.09	-17.42
1 uM	98.20	0.83	23.39	96.77	0.08	-20.22
10 uM	90.56	0.71	-0.69	107.27	0.10	-9.30

Compound	Normalized Values (A and B Sets)	Responses

Adenine HCl

Average Response

Dose	Green Signal			Red Signal	Actual Pct Red Cells	

Compound	Normalized Values (A and B Sets)	Responses

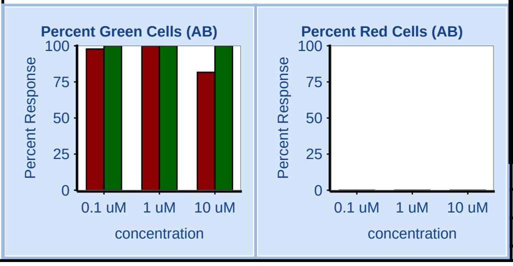

		Actual Pct Green Cells	Normalized Pct Green Cells			Normalized Pct Red Cells
0.1 uM	103.84	0.91	103.08	82.97	0.04	-52.66
1 uM	125.58	0.97	181.74	100.66	0.09	-17.67
10 uM	102.73	0.86	91.34	105.59	0.07	-23.60

Compound	Normalized Values (A and B Sets)	Responses

ADL5859 HCl

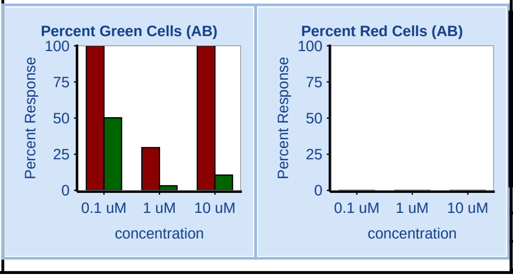

Average Response						
Dose	Green Signal	Actual Pct Green Cells	Normalized Pct Green Cells	Red Signal	Actual Pct Red Cells	Normalized Pct Red Cells
0.1 uM	96.14	0.88	100.30	68.39	0.02	-60.22
1 uM	88.93	0.80	16.39	84.53	0.03	-44.60
10 uM	89.49	0.83	60.17	96.59	0.03	-39.03

Compound	Normalized Values (A and B Sets)	Responses

Adrenalone HCl

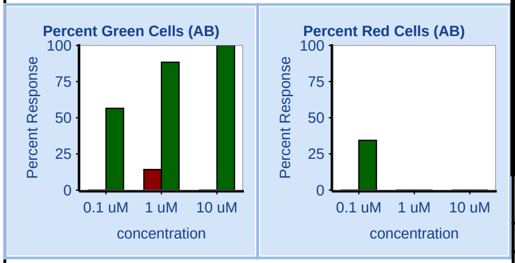

Average Response						
Dose	Green Signal	Actual Pct Green Cells	Normalized Pct Green Cells	Red Signal	Actual Pct Red Cells	Normalized Pct Red Cells
0.1 uM	81.80	0.80	-199.25	98.95	0.11	-7.31
1 uM	93.27	0.79	51.29	99.73	0.08	-26.66
10 uM	92.50	0.85	42.41	79.55	0.06	-34.07

Compound	Normalized Values (A and B Sets)	Responses

ADX-47273

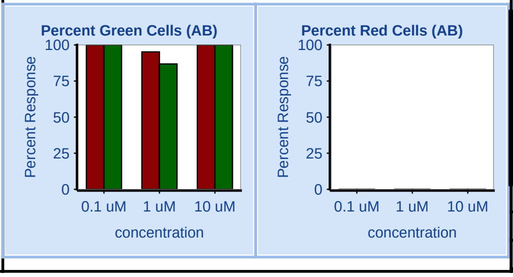

Average Response						
Dose	Green Signal	Actual Pct Green Cells	Normalized Pct Green Cells	Red Signal	Actual Pct Red Cells	Normalized Pct Red Cells
0.1 uM	112.07	0.94	153.69	82.26	0.06	-29.48
1 uM	105.86	0.88	91.03	86.76	0.07	-23.05
10 uM	107.31	0.94	321.84	94.93	0.07	-23.50

Compound	Normalized Values (A and B Sets)	Responses

AEE788 (NVP-AEE788)

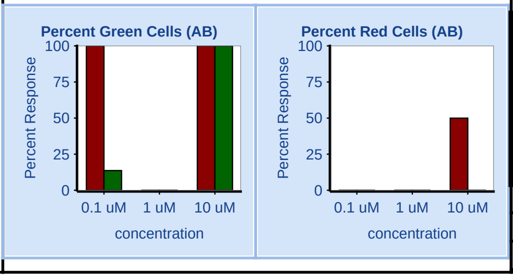

Average Response						
Dose	Green Signal	Actual Pct Green Cells	Normalized Pct Green Cells	Red Signal	Actual Pct Red Cells	Normalized Pct Red Cells
0.1 uM	100.36	0.86	99.26	100.81	0.09	-26.15
1 uM	88.64	0.76	-30.09	101.50	0.08	-19.55
10 uM	122.34	0.92	130.83	139.40	0.17	13.79

Compound | Normalized Values (A and B Sets) | Responses

Afatinib (BIBW2992)

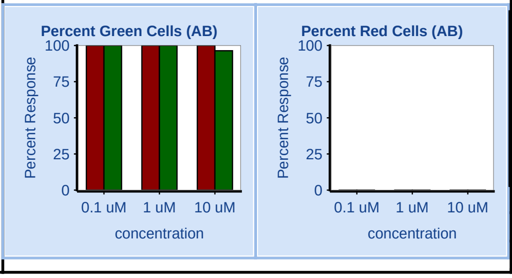

Average Response

Dose	Green Signal	Actual Pct Green Cells	Normalized Pct Green Cells	Red Signal	Actual Pct Red Cells	Normalized Pct Red Cells
0.1 uM	106.03	0.95	158.47	77.74	0.02	-60.88
1 uM	108.50	0.96	251.53	75.64	0.03	-35.06
10 uM	110.04	0.95	133.80	90.02	0.07	-19.26

Compound | Normalized Values (A and B Sets) | Responses

AG-18

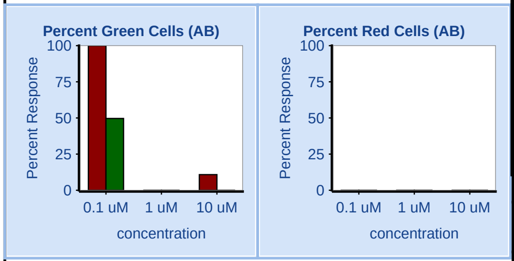

Average Response

Dose	Green Signal	Actual Pct Green Cells	Normalized Pct Green Cells	Red Signal	Actual Pct Red Cells	Normalized Pct Red Cells
0.1 uM	99.52	0.89	102.20	55.48	0.01	-72.21
1 uM	85.39	0.79	-246.11	80.31	0.04	-28.70
10 uM	79.98	0.72	-85.63	61.29	0.02	-43.27

Compound | Normalized Values (A and B Sets) | Responses

AG-490 (Tyrphostin B42)

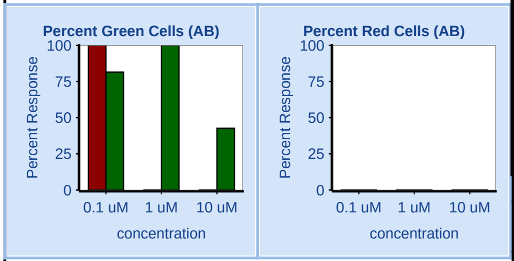

Average Response

Dose	Green Signal	Actual Pct Green Cells	Normalized Pct Green Cells	Red Signal	Actual Pct Red Cells	Normalized Pct Red Cells
0.1 uM	94.19	0.89	99.48	75.89	0.01	-66.76
1 uM	93.99	0.85	-292.22	66.66	0.02	-40.44
10 uM	88.15	0.84	20.63	71.19	0.03	-37.40

Compound | Normalized Values (A and B Sets) | Responses

AG-14361

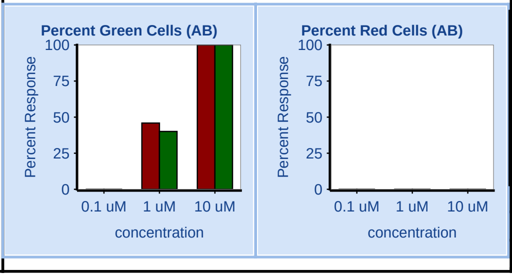

Average Response

Dose	Green Signal	Actual Pct Green Cells	Normalized Pct Green Cells	Red Signal	Actual Pct Red Cells	Normalized Pct Red Cells
0.1 uM	80.97	0.77	-30.28	77.41	0.04	-35.82
1 uM	92.24	0.78	42.99	69.52	0.02	-48.26
10 uM	99.89	0.93	165.28	98.68	0.08	-20.24

Compound | Normalized Values (A and B Sets) | Responses

AGI-5198

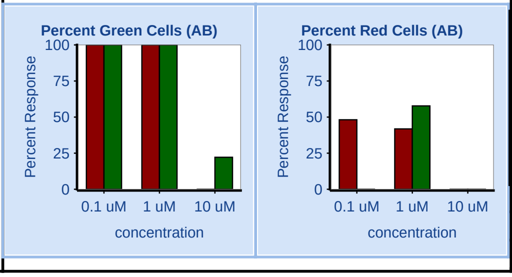

Average Response

Dose	Green Signal	Actual Pct Green Cells	Normalized Pct Green Cells	Red Signal	Actual Pct Red Cells	Normalized Pct Red Cells
0.1 uM	118.52	0.97	246.43	104.30	0.15	9.62
1 uM	109.15	0.89	123.60	124.58	0.23	49.79
10 uM	89.70	0.74	-27.51	86.47	0.06	-32.20

Compound	Normalized Values (A and B Sets)	Responses
**Agomelatine**	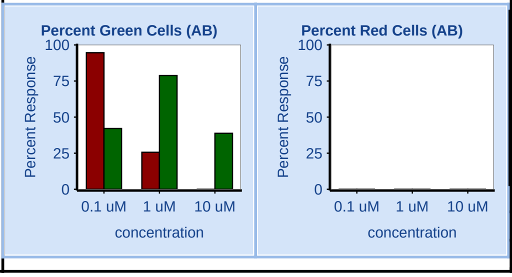	see table below

Average Response

Dose	Green Signal	Actual Pct Green Cells	Normalized Pct Green Cells	Red Signal	Actual Pct Red Cells	Normalized Pct Red Cells
0.1 uM	95.63	0.85	68.36	75.70	0.02	-61.48
1 uM	96.94	0.86	52.23	85.25	0.06	-31.14
10 uM	86.65	0.75	16.20	106.76	0.07	-23.21

Compound	Normalized Values (A and B Sets)	Responses
**Albendazole Oxide**	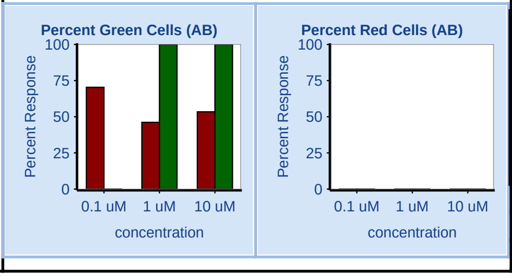	see table below

Average Response

Dose	Green Signal	Actual Pct Green Cells	Normalized Pct Green Cells	Red Signal	Actual Pct Red Cells	Normalized Pct Red Cells
0.1 uM	92.92	0.79	25.17	85.90	0.05	-50.04
1 uM	112.45	0.91	89.61	84.90	0.04	-41.12
10 uM	103.42	0.86	91.23	101.48	0.07	-25.96

Compound	Normalized Values (A and B Sets)	Responses
**Alectinib (CH5424802)**	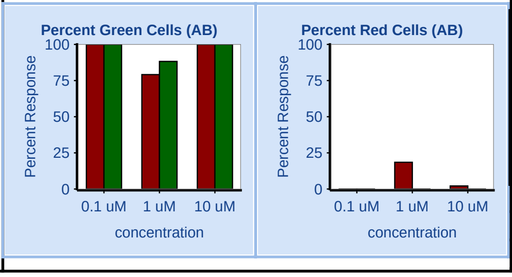	see table below

Average Response

Dose	Green Signal	Actual Pct Green Cells	Normalized Pct Green Cells	Red Signal	Actual Pct Red Cells	Normalized Pct Red Cells
0.1 uM	106.93	0.93	140.64	80.74	0.05	-32.46
1 uM	100.67	0.86	83.69	106.59	0.12	-0.35
10 uM	103.78	0.94	267.06	92.05	0.07	-22.34

Compound	Normalized Values (A and B Sets)	Responses
**Alibendol**	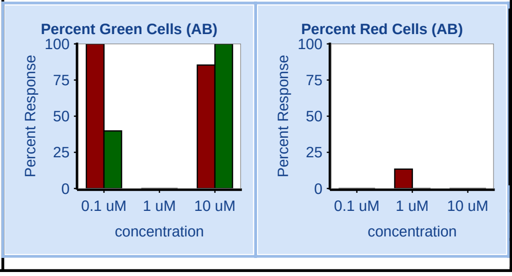	see table below

Average Response

Dose	Green Signal	Actual Pct Green Cells	Normalized Pct Green Cells	Red Signal	Actual Pct Red Cells	Normalized Pct Red Cells
0.1 uM	99.73	0.87	88.21	84.97	0.04	-48.74
1 uM	90.50	0.74	-33.75	89.84	0.08	-22.79
10 uM	107.54	0.88	100.92	104.14	0.06	-26.87

Compound	Normalized Values (A and B Sets)	Responses
**Alisertib (MLN8237)**	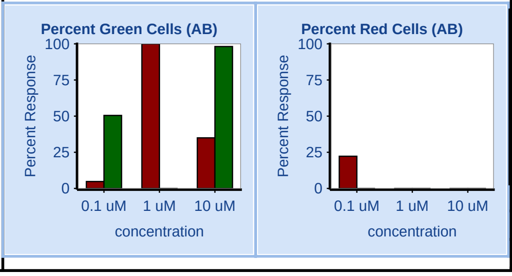	see table below

Average Response

Dose	Green Signal	Actual Pct Green Cells	Normalized Pct Green Cells	Red Signal	Actual Pct Red Cells	Normalized Pct Red Cells
0.1 uM	89.61	0.82	27.64	104.94	0.11	-23.33
1 uM	89.14	0.71	15.42	94.82	0.07	-24.57
10 uM	96.38	0.82	66.56	92.61	0.04	-38.91

Compound	Normalized Values (A and B Sets)	Responses

Aliskiren Hemifumarate

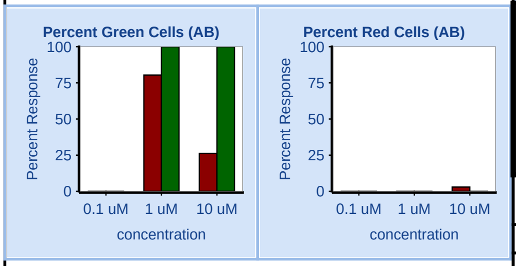

Average Response						
Dose	Green Signal	Actual Pct Green Cells	Normalized Pct Green Cells	Red Signal	Actual Pct Red Cells	Normalized Pct Red Cells
0.1 uM	71.89	0.61	-175.70	63.96	0.01	-49.12
1 uM	101.82	0.87	90.86	75.88	0.02	-44.89
10 uM	94.97	0.90	73.34	103.75	0.09	-13.63

Compound	Normalized Values (A and B Sets)	Responses

Allopurinol

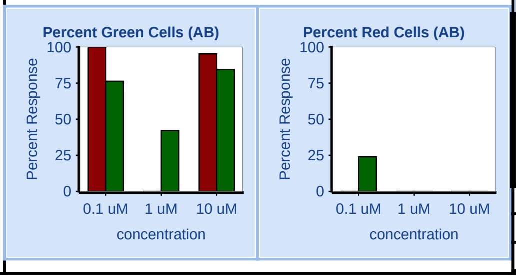

Average Response						
Dose	Green Signal	Actual Pct Green Cells	Normalized Pct Green Cells	Red Signal	Actual Pct Red Cells	Normalized Pct Red Cells
0.1 uM	105.94	0.90	108.55	105.88	0.11	-6.91
1 uM	93.92	0.80	-3.02	101.68	0.08	-20.79
10 uM	109.01	0.86	89.86	113.35	0.11	-4.67

Compound	Normalized Values (A and B Sets)	Responses

Allopurinol Sodium

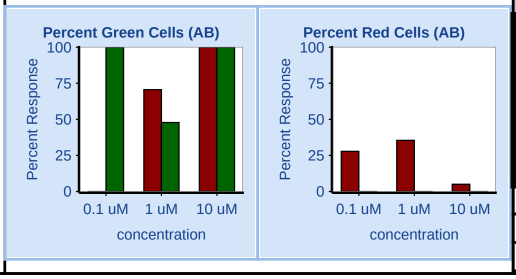

Average Response						
Dose	Green Signal	Actual Pct Green Cells	Normalized Pct Green Cells	Red Signal	Actual Pct Red Cells	Normalized Pct Red Cells
0.1 uM	106.16	0.87	48.54	115.62	0.16	9.37
1 uM	100.46	0.85	59.19	112.54	0.15	9.09
10 uM	110.97	0.90	113.14	102.93	0.09	-16.86

Compound	Normalized Values (A and B Sets)	Responses

Allylthiourea

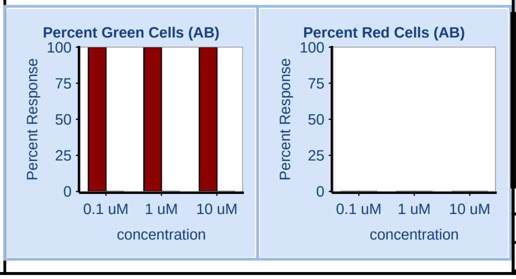

Average Response						
Dose	Green Signal	Actual Pct Green Cells	Normalized Pct Green Cells	Red Signal	Actual Pct Red Cells	Normalized Pct Red Cells
0.1 uM	86.10	0.81	44.42	60.99	0.01	-57.62
1 uM	90.82	0.80	52.26	75.97	0.02	-56.12
10 uM	68.54	0.50	-59.62	78.17	0.03	-49.51

Compound	Normalized Values (A and B Sets)	Responses

Almotriptan Malate

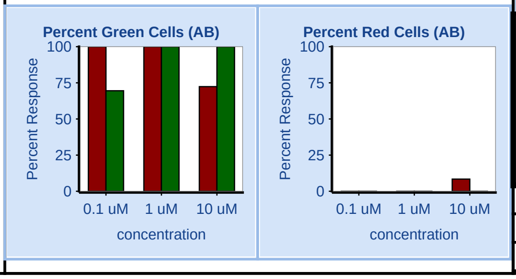

Average Response						
Dose	Green Signal	Actual Pct Green Cells	Normalized Pct Green Cells	Red Signal	Actual Pct Red Cells	Normalized Pct Red Cells
0.1 uM	108.02	0.90	116.16	91.02	0.06	-44.49
1 uM	119.64	0.94	154.52	92.35	0.06	-30.77
10 uM	108.21	0.87	98.01	108.71	0.10	-11.52

Compound | Normalized Values (A and B Sets) | Responses

Aloe-emodin

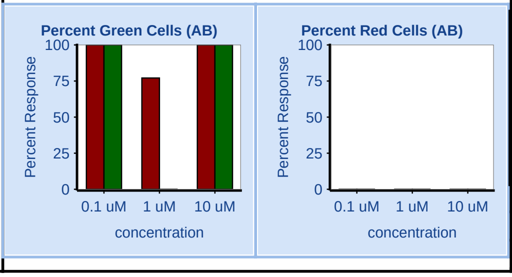

Average Response

Dose	Green Signal	Actual Pct Green Cells	Normalized Pct Green Cells	Red Signal	Actual Pct Red Cells	Normalized Pct Red Cells
0.1 uM	103.20	0.92	126.82	70.57	0.02	-43.86
1 uM	94.18	0.75	15.76	73.66	0.03	-42.80
10 uM	101.44	0.93	192.05	87.24	0.04	-40.93

Compound | Normalized Values (A and B Sets) | Responses

Alogliptin

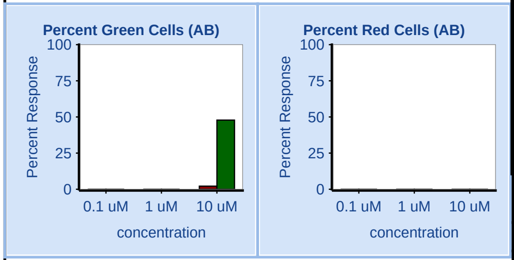

Average Response

Dose	Green Signal	Actual Pct Green Cells	Normalized Pct Green Cells	Red Signal	Actual Pct Red Cells	Normalized Pct Red Cells
0.1 uM	75.70	0.69	-313.08	61.60	0.01	-58.80
1 uM	80.21	0.67	-39.59	70.13	0.01	-61.54
10 uM	88.00	0.79	24.98	79.21	0.04	-44.70

Compound | Normalized Values (A and B Sets) | Responses

Aloin

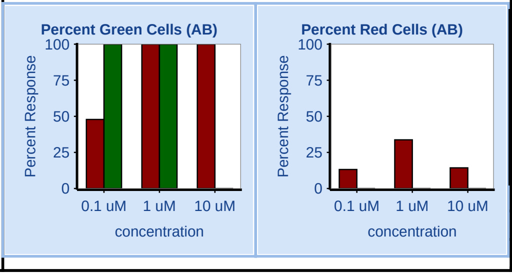

Average Response

Dose	Green Signal	Actual Pct Green Cells	Normalized Pct Green Cells	Red Signal	Actual Pct Red Cells	Normalized Pct Red Cells
0.1 uM	96.05	0.88	84.37	88.28	0.08	-18.08
1 uM	111.12	0.92	115.10	95.38	0.11	-6.69
10 uM	84.67	0.84	43.90	92.90	0.09	-16.97

Compound | Normalized Values (A and B Sets) | Responses

Aloperine

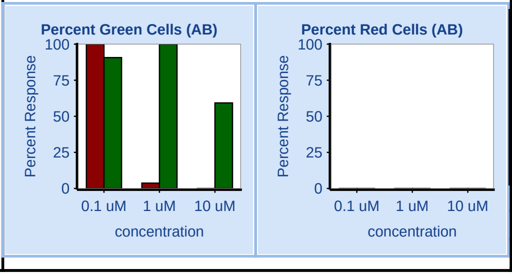

Average Response

Dose	Green Signal	Actual Pct Green Cells	Normalized Pct Green Cells	Red Signal	Actual Pct Red Cells	Normalized Pct Red Cells
0.1 uM	100.02	0.90	101.90	79.95	0.04	-35.14
1 uM	92.12	0.79	53.84	84.15	0.05	-33.77
10 uM	90.58	0.87	-69.29	69.92	0.01	-52.21

Compound | Normalized Values (A and B Sets) | Responses

Altrenogest

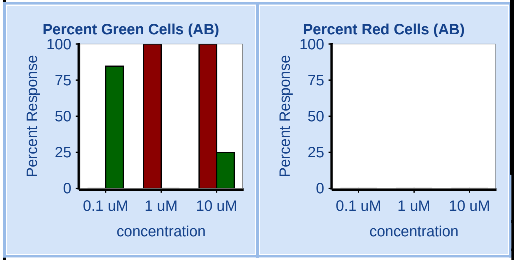

Average Response

Dose	Green Signal	Actual Pct Green Cells	Normalized Pct Green Cells	Red Signal	Actual Pct Red Cells	Normalized Pct Red Cells
0.1 uM	86.71	0.81	-226.41	67.18	0.02	-56.19
1 uM	84.50	0.70	-24.61	70.68	0.02	-57.56
10 uM	94.98	0.83	111.59	78.03	0.03	-47.92

Compound	Normalized Values (A and B Sets)	Responses

AM1241

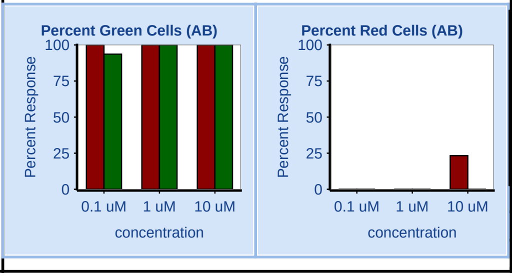

Average Response

Dose	Green Signal	Actual Pct Green Cells	Normalized Pct Green Cells	Red Signal	Actual Pct Red Cells	Normalized Pct Red Cells
0.1 uM	106.52	0.92	124.16	99.78	0.08	-33.96
1 uM	111.43	0.95	146.90	102.47	0.09	-17.30
10 uM	107.12	0.89	110.57	124.06	0.14	1.56

Compound	Normalized Values (A and B Sets)	Responses

Ambrisentan

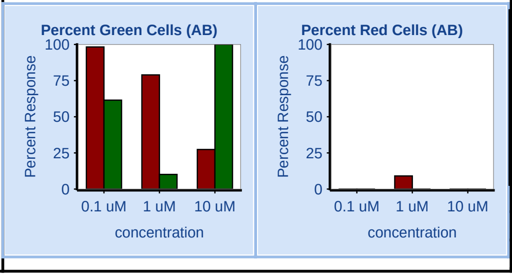

Average Response

Dose	Green Signal	Actual Pct Green Cells	Normalized Pct Green Cells	Red Signal	Actual Pct Red Cells	Normalized Pct Red Cells
0.1 uM	102.13	0.87	79.90	93.53	0.06	-40.74
1 uM	100.67	0.82	44.55	109.95	0.12	-3.84
10 uM	101.40	0.82	66.52	82.92	0.06	-27.58

Compound	Normalized Values (A and B Sets)	Responses

Amfenac Sodium Monohydrate

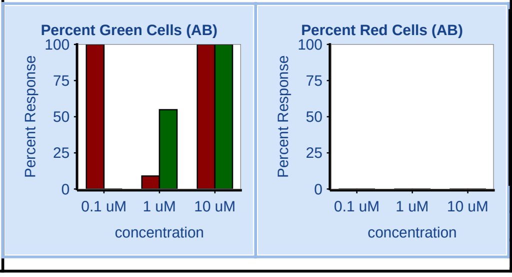

Average Response

Dose	Green Signal	Actual Pct Green Cells	Normalized Pct Green Cells	Red Signal	Actual Pct Red Cells	Normalized Pct Red Cells
0.1 uM	89.98	0.81	81.31	80.12	0.04	-43.29
1 uM	89.12	0.76	31.94	89.31	0.08	-29.85
10 uM	116.16	0.96	159.90	103.49	0.05	-37.22

Compound	Normalized Values (A and B Sets)	Responses

AMG-458

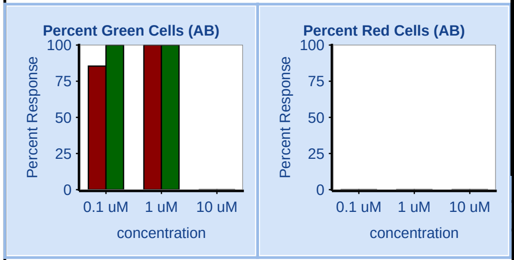

Average Response

Dose	Green Signal	Actual Pct Green Cells	Normalized Pct Green Cells	Red Signal	Actual Pct Red Cells	Normalized Pct Red Cells
0.1 uM	95.28	0.90	108.81	86.64	0.06	-24.97
1 uM	109.26	0.92	114.96	81.47	0.04	-37.16
10 uM	82.52	0.82	-205.97	87.28	0.05	-37.08

Compound	Normalized Values (A and B Sets)	Responses

AMG-517

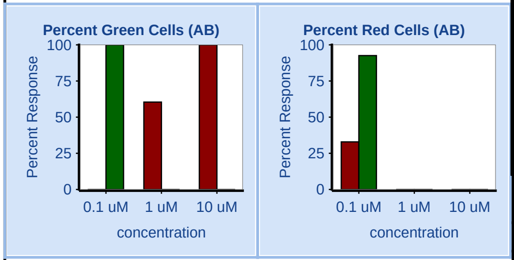

Average Response

Dose	Green Signal	Actual Pct Green Cells	Normalized Pct Green Cells	Red Signal	Actual Pct Red Cells	Normalized Pct Red Cells
0.1 uM	98.32	0.86	-206.32	125.40	0.25	62.73
1 uM	86.11	0.71	-14.51	79.28	0.05	-41.71
10 uM	94.53	0.77	65.33	93.43	0.08	-18.62

Compound | Normalized Values (A and B Sets) | Responses

AMG-900

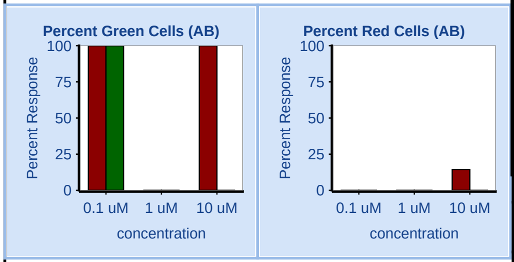

Average Response						
Dose	Green Signal	Actual Pct Green Cells	Normalized Pct Green Cells	Red Signal	Actual Pct Red Cells	Normalized Pct Red Cells
0.1 uM	95.93	0.91	115.07	76.62	0.04	-48.93
1 uM	77.83	0.67	-653.14	78.90	0.05	-22.09
10 uM	91.10	0.84	33.93	95.97	0.11	-3.62

Compound | Normalized Values (A and B Sets) | Responses

Aminothiazole

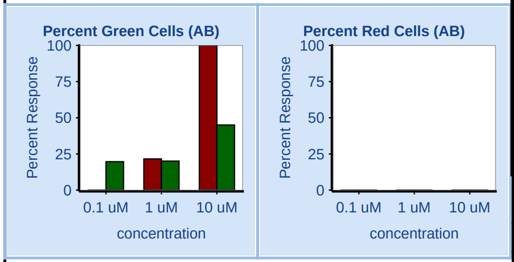

Average Response						
Dose	Green Signal	Actual Pct Green Cells	Normalized Pct Green Cells	Red Signal	Actual Pct Red Cells	Normalized Pct Red Cells
0.1 uM	81.54	0.80	-139.01	72.22	0.02	-52.81
1 uM	92.11	0.75	20.80	95.35	0.09	-19.77
10 uM	98.52	0.83	82.10	89.71	0.07	-28.42

Compound | Normalized Values (A and B Sets) | Responses

Ammonium Glycyrrhizinate

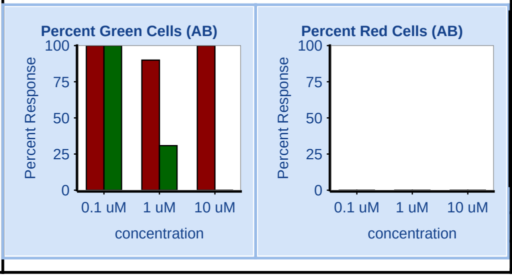

Average Response						
Dose	Green Signal	Actual Pct Green Cells	Normalized Pct Green Cells	Red Signal	Actual Pct Red Cells	Normalized Pct Red Cells
0.1 uM	101.65	0.94	143.86	96.85	0.10	-6.49
1 uM	97.40	0.83	60.38	86.44	0.06	-28.65
10 uM	89.15	0.88	163.39	88.82	0.05	-32.59

Compound | Normalized Values (A and B Sets) | Responses

Amorolfine HCl

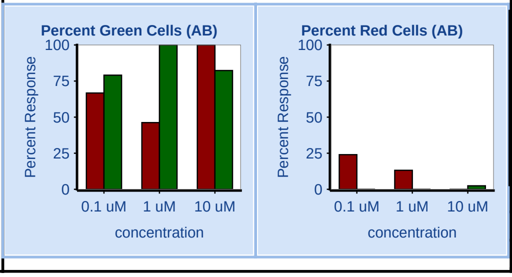

Average Response						
Dose	Green Signal	Actual Pct Green Cells	Normalized Pct Green Cells	Red Signal	Actual Pct Red Cells	Normalized Pct Red Cells
0.1 uM	98.38	0.87	72.90	113.04	0.14	-5.79
1 uM	110.73	0.90	85.39	110.35	0.13	1.30
10 uM	106.10	0.87	91.42	114.03	0.11	-3.13

Compound | Normalized Values (A and B Sets) | Responses

Amoxicillin Sodium

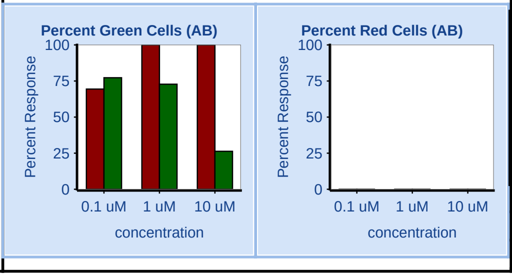

Average Response						
Dose	Green Signal	Actual Pct Green Cells	Normalized Pct Green Cells	Red Signal	Actual Pct Red Cells	Normalized Pct Red Cells
0.1 uM	96.54	0.87	73.34	75.08	0.03	-38.74
1 uM	104.20	0.88	90.88	107.60	0.11	-6.74
10 uM	98.60	0.89	149.85	83.82	0.03	-43.44

Compound | Normalized Values (A and B Sets) | Responses

Amphotericin B

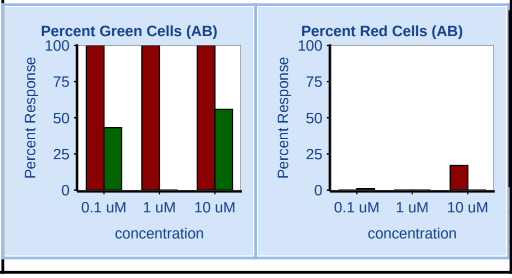

Average Response						
Dose	Green Signal	Actual Pct Green Cells	Normalized Pct Green Cells	Red Signal	Actual Pct Red Cells	Normalized Pct Red Cells
0.1 uM	101.82	0.86	76.61	102.13	0.10	-17.23
1 uM	100.02	0.82	73.31	102.66	0.09	-17.34
10 uM	108.10	0.87	92.58	116.82	0.11	-9.13

Compound | Normalized Values (A and B Sets) | Responses

Amprenavir

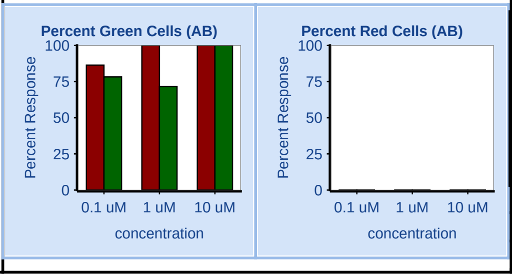

Average Response						
Dose	Green Signal	Actual Pct Green Cells	Normalized Pct Green Cells	Red Signal	Actual Pct Red Cells	Normalized Pct Red Cells
0.1 uM	99.88	0.88	82.38	96.98	0.08	-32.56
1 uM	108.01	0.89	112.09	108.14	0.10	-10.46
10 uM	112.32	0.90	115.66	86.62	0.04	-37.75

Compound | Normalized Values (A and B Sets) | Responses

Amuvatinib (MP-470)

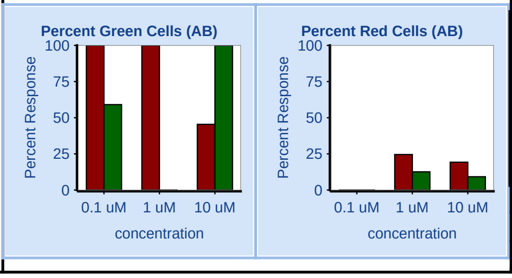

Average Response						
Dose	Green Signal	Actual Pct Green Cells	Normalized Pct Green Cells	Red Signal	Actual Pct Red Cells	Normalized Pct Red Cells
0.1 uM	93.44	0.88	91.80	91.65	0.05	-47.26
1 uM	105.61	0.82	67.32	119.34	0.17	18.57
10 uM	99.11	0.84	78.35	124.07	0.16	14.21

Compound | Normalized Values (A and B Sets) | Responses

Anacetrapib (MK-0859)

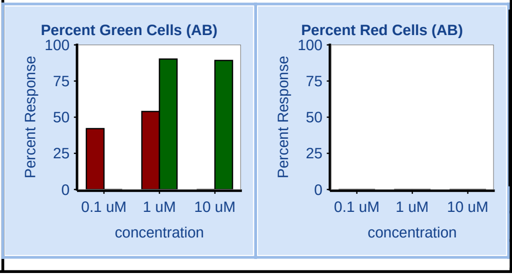

Average Response						
Dose	Green Signal	Actual Pct Green Cells	Normalized Pct Green Cells	Red Signal	Actual Pct Red Cells	Normalized Pct Red Cells
0.1 uM	88.06	0.82	17.58	77.70	0.03	-41.18
1 uM	101.02	0.83	72.07	92.53	0.07	-25.70
10 uM	94.41	0.89	16.91	82.19	0.04	-37.32

Compound | Normalized Values (A and B Sets) | Responses

Anagrelide

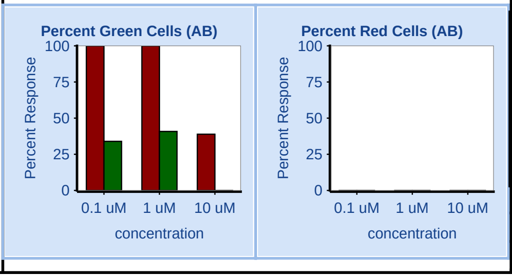

Average Response						
Dose	Green Signal	Actual Pct Green Cells	Normalized Pct Green Cells	Red Signal	Actual Pct Red Cells	Normalized Pct Red Cells
0.1 uM	97.40	0.88	99.72	71.74	0.02	-65.03
1 uM	97.71	0.88	179.93	80.83	0.05	-21.57
10 uM	88.63	0.80	-10.66	83.47	0.07	-18.45

Compound | Normalized Values (A and B Sets) | Responses

Anagrelide HCl

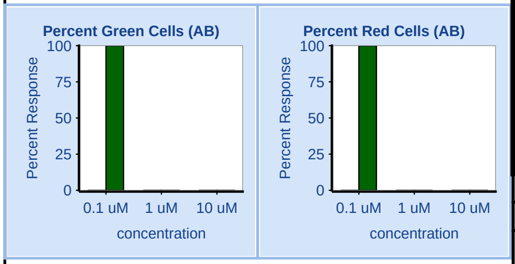

Average Response

Dose	Green Signal	Actual Pct Green Cells	Normalized Pct Green Cells	Red Signal	Actual Pct Red Cells	Normalized Pct Red Cells
0.1 uM	92.21	0.88	-49.49	95.43	0.16	18.72
1 uM	77.83	0.63	-64.99	81.86	0.06	-37.78
10 uM	78.78	0.63	-158.82	63.36	0.01	-57.98

Compound | Normalized Values (A and B Sets) | Responses

Andarine

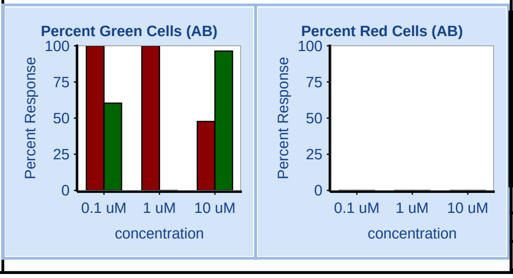

Average Response

Dose	Green Signal	Actual Pct Green Cells	Normalized Pct Green Cells	Red Signal	Actual Pct Red Cells	Normalized Pct Red Cells
0.1 uM	92.78	0.87	80.53	74.96	0.02	-63.76
1 uM	93.32	0.74	21.36	96.82	0.06	-31.57
10 uM	99.63	0.83	72.03	98.98	0.07	-25.72

Compound | Normalized Values (A and B Sets) | Responses

Andrographolide

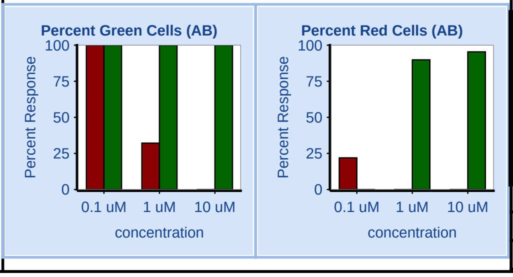

Average Response

Dose	Green Signal	Actual Pct Green Cells	Normalized Pct Green Cells	Red Signal	Actual Pct Red Cells	Normalized Pct Red Cells
0.1 uM	113.56	0.94	151.63	103.47	0.13	6.76
1 uM	103.63	0.82	68.42	116.14	0.22	41.72
10 uM	112.04	0.90	-88.66	112.03	0.20	34.14

Compound | Normalized Values (A and B Sets) | Responses

Anisotropine Methylbromide

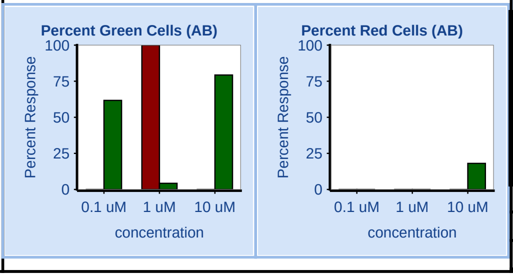

Average Response

Dose	Green Signal	Actual Pct Green Cells	Normalized Pct Green Cells	Red Signal	Actual Pct Red Cells	Normalized Pct Red Cells
0.1 uM	92.31	0.86	17.53	74.98	0.04	-43.14
1 uM	95.59	0.80	54.18	91.77	0.09	-23.18
10 uM	92.31	0.76	-63.94	97.32	0.10	-9.05

Compound | Normalized Values (A and B Sets) | Responses

AP26113

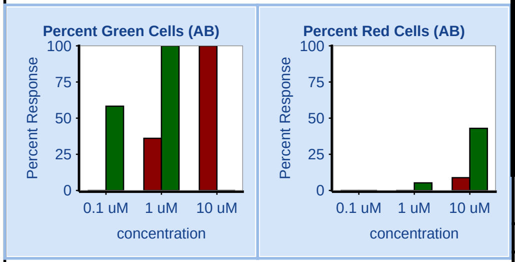

Average Response

Dose	Green Signal	Actual Pct Green Cells	Normalized Pct Green Cells	Red Signal	Actual Pct Red Cells	Normalized Pct Red Cells
0.1 uM	82.96	0.74	-493.79	73.48	0.04	-42.88
1 uM	106.80	0.85	100.01	97.94	0.10	-17.87
10 uM	96.79	0.73	42.23	101.67	0.17	25.91

Compound	Normalized Values (A and B Sets)	Responses

Apatinib

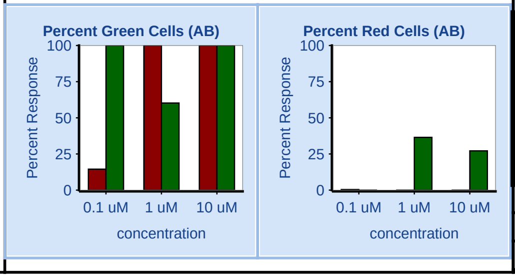

Average Response

Dose	Green Signal	Actual Pct Green Cells	Normalized Pct Green Cells	Red Signal	Actual Pct Red Cells	Normalized Pct Red Cells
0.1 uM	96.29	0.88	86.54	93.00	0.09	-14.45
1 uM	108.38	0.87	82.45	106.82	0.15	11.59
10 uM	103.12	0.96	428.00	112.12	0.14	6.77

Compound	Normalized Values (A and B Sets)	Responses

Apixaban

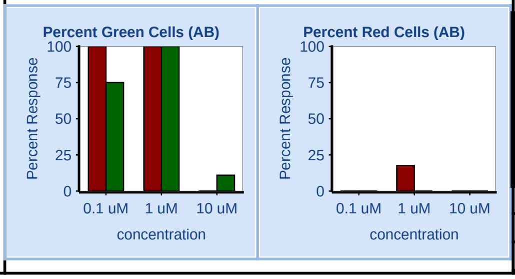

Average Response

Dose	Green Signal	Actual Pct Green Cells	Normalized Pct Green Cells	Red Signal	Actual Pct Red Cells	Normalized Pct Red Cells
0.1 uM	100.23	0.91	116.95	99.17	0.07	-37.92
1 uM	108.60	0.92	116.20	101.21	0.10	-10.83
10 uM	82.57	0.67	-33.48	100.61	0.07	-25.34

Compound	Normalized Values (A and B Sets)	Responses

Apocynin

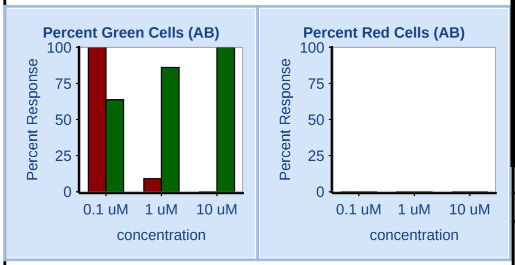

Average Response

Dose	Green Signal	Actual Pct Green Cells	Normalized Pct Green Cells	Red Signal	Actual Pct Red Cells	Normalized Pct Red Cells
0.1 uM	98.95	0.89	94.95	97.03	0.10	-8.63
1 uM	96.71	0.78	47.58	74.63	0.03	-43.94
10 uM	85.91	0.68	-1670.69	79.32	0.03	-43.08

Compound	Normalized Values (A and B Sets)	Responses

Apogossypolone (ApoG2)

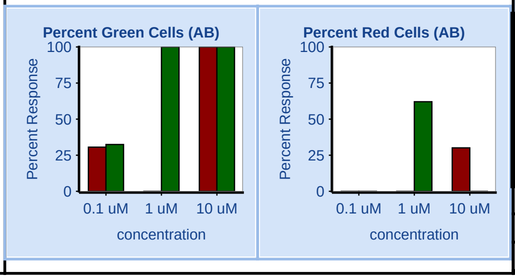

Average Response

Dose	Green Signal	Actual Pct Green Cells	Normalized Pct Green Cells	Red Signal	Actual Pct Red Cells	Normalized Pct Red Cells
0.1 uM	88.33	0.81	31.52	88.76	0.06	-38.37
1 uM	97.89	0.89	-40.78	90.60	0.11	17.82
10 uM	103.45	0.95	128.94	103.09	0.14	13.27

Compound	Normalized Values (A and B Sets)	Responses

Apoptosis Activator 2

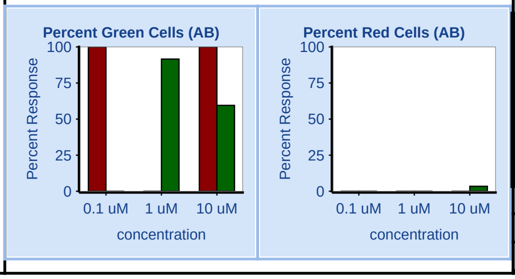

Average Response

Dose	Green Signal	Actual Pct Green Cells	Normalized Pct Green Cells	Red Signal	Actual Pct Red Cells	Normalized Pct Red Cells
0.1 uM	92.02	0.84	181.48	83.46	0.04	-45.93
1 uM	90.10	0.77	41.99	70.59	0.02	-55.74
10 uM	95.11	0.84	83.41	89.06	0.08	-24.03

Compound	Normalized Values (A and B Sets)	Responses

Apremilast (CC-10004)

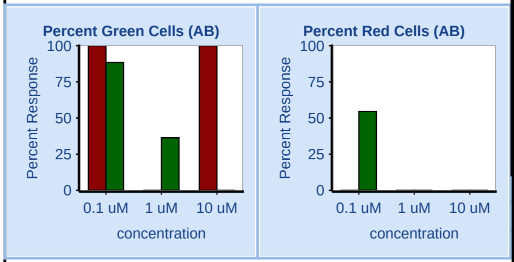

Average Response

Dose	Green Signal	Actual Pct Green Cells	Normalized Pct Green Cells	Red Signal	Actual Pct Red Cells	Normalized Pct Red Cells
0.1 uM	94.69	0.88	94.21	95.62	0.11	-6.43
1 uM	91.35	0.85	-35.72	63.99	0.01	-41.22
10 uM	75.20	0.67	-123.87	88.13	0.06	-25.79

Compound	Normalized Values (A and B Sets)	Responses

Aprepitant

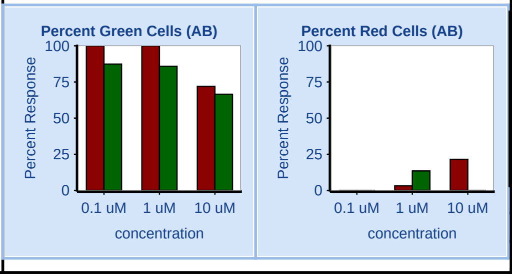

Average Response

Dose	Green Signal	Actual Pct Green Cells	Normalized Pct Green Cells	Red Signal	Actual Pct Red Cells	Normalized Pct Red Cells
0.1 uM	97.98	0.90	100.20	98.22	0.07	-36.55
1 uM	112.28	0.89	97.12	117.04	0.14	8.32
10 uM	100.95	0.83	69.27	115.51	0.11	-8.70

Compound	Normalized Values (A and B Sets)	Responses

AR-42

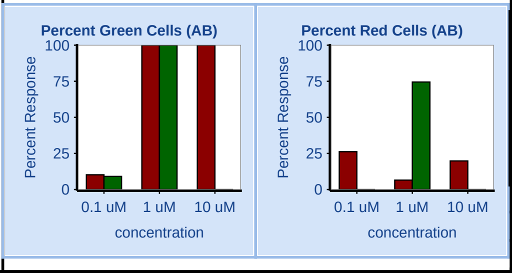

Average Response

Dose	Green Signal	Actual Pct Green Cells	Normalized Pct Green Cells	Red Signal	Actual Pct Red Cells	Normalized Pct Red Cells
0.1 uM	91.09	0.81	9.64	101.96	0.13	3.98
1 uM	118.02	0.92	113.30	144.62	0.22	40.50
10 uM	116.49	0.86	345.20	113.94	0.11	-4.41

Compound	Normalized Values (A and B Sets)	Responses

AR-A 014418

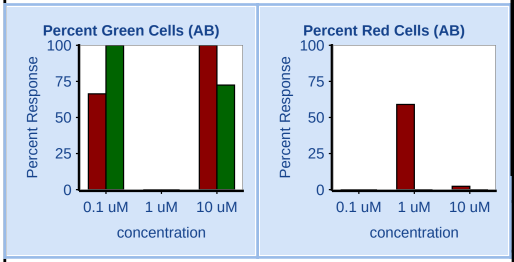

Average Response

Dose	Green Signal	Actual Pct Green Cells	Normalized Pct Green Cells	Red Signal	Actual Pct Red Cells	Normalized Pct Red Cells
0.1 uM	99.66	0.89	98.18	88.66	0.06	-34.82
1 uM	74.98	0.66	-661.64	101.50	0.17	14.56
10 uM	94.55	0.90	87.22	83.91	0.08	-14.14

Compound	Normalized Values (A and B Sets)	Responses

Arbidol HCl

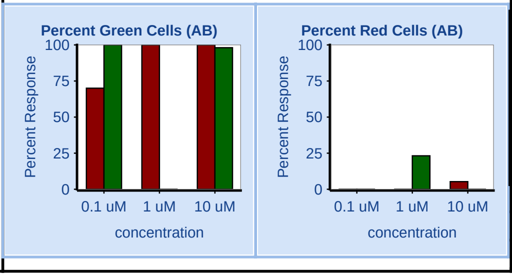

Average Response

Dose	Green Signal	Actual Pct Green Cells	Normalized Pct Green Cells	Red Signal	Actual Pct Red Cells	Normalized Pct Red Cells
0.1 uM	100.85	0.90	108.08	89.57	0.06	-35.82
1 uM	96.11	0.87	215.64	87.64	0.08	-0.82
10 uM	99.98	0.94	118.29	91.42	0.12	2.27

Compound	Normalized Values (A and B Sets)	Responses

ARN-509

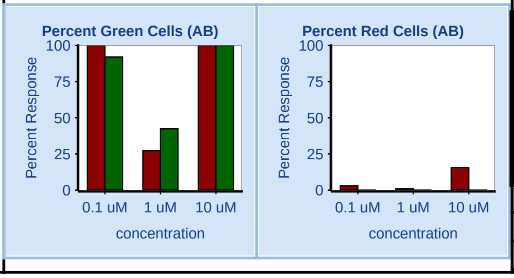

Average Response						
Dose	Green Signal	Actual Pct Green Cells	Normalized Pct Green Cells	Red Signal	Actual Pct Red Cells	Normalized Pct Red Cells
0.1 uM	104.64	0.92	124.00	101.51	0.12	0.16
1 uM	93.95	0.77	34.81	104.46	0.11	-6.46
10 uM	105.18	0.92	231.72	95.89	0.09	-15.19

Compound	Normalized Values (A and B Sets)	Responses

Asenapine

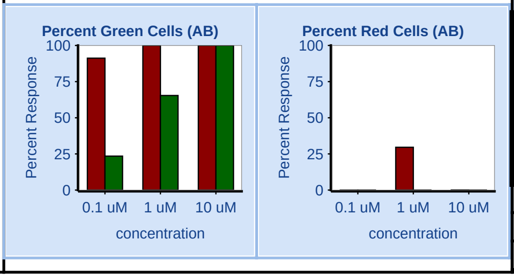

Average Response						
Dose	Green Signal	Actual Pct Green Cells	Normalized Pct Green Cells	Red Signal	Actual Pct Red Cells	Normalized Pct Red Cells
0.1 uM	94.22	0.83	57.36	98.46	0.11	-14.73
1 uM	104.55	0.89	116.62	114.02	0.14	4.18
10 uM	100.70	0.93	134.87	109.84	0.07	-24.89

Compound	Normalized Values (A and B Sets)	Responses

Asiatic Acid

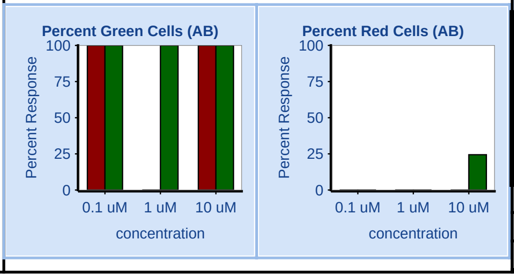

Average Response						
Dose	Green Signal	Actual Pct Green Cells	Normalized Pct Green Cells	Red Signal	Actual Pct Red Cells	Normalized Pct Red Cells
0.1 uM	106.14	0.92	121.25	78.25	0.04	-36.10
1 uM	93.90	0.78	49.35	84.03	0.07	-24.98
10 uM	101.50	0.93	321.49	100.35	0.10	-10.26

Compound	Normalized Values (A and B Sets)	Responses

AST-1306

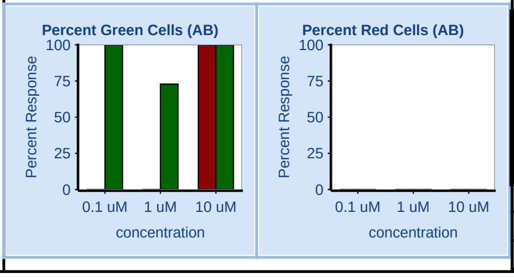

Average Response						
Dose	Green Signal	Actual Pct Green Cells	Normalized Pct Green Cells	Red Signal	Actual Pct Red Cells	Normalized Pct Red Cells
0.1 uM	93.24	0.85	60.59	60.51	0.01	-50.75
1 uM	90.03	0.74	27.17	66.89	0.02	-48.87
10 uM	112.31	0.97	402.72	82.97	0.04	-37.74

Compound	Normalized Values (A and B Sets)	Responses

Astragaloside A

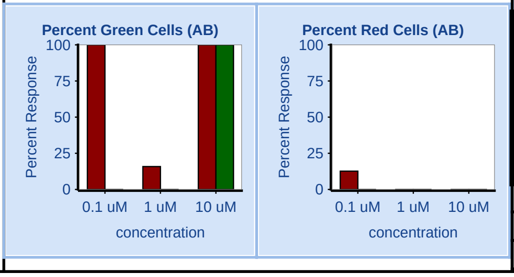

Average Response						
Dose	Green Signal	Actual Pct Green Cells	Normalized Pct Green Cells	Red Signal	Actual Pct Red Cells	Normalized Pct Red Cells
0.1 uM	99.79	0.83	21.32	94.88	0.12	-0.49
1 uM	82.61	0.71	1.40	85.96	0.06	-27.40
10 uM	106.07	0.93	324.51	94.98	0.09	-16.76

Compound | Normalized Values (A and B Sets) | Responses

AT7519

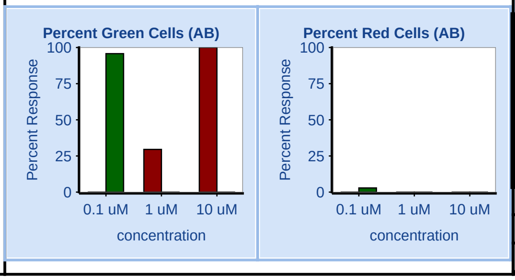

Average Response

Dose	Green Signal	Actual Pct Green Cells	Normalized Pct Green Cells	Red Signal	Actual Pct Red Cells	Normalized Pct Red Cells
0.1 uM	99.57	0.86	40.82	110.16	0.13	-4.17
1 uM	90.28	0.73	-26.95	96.61	0.07	-27.23
10 uM	92.33	0.73	-13.94	102.78	0.09	-11.86

Compound | Normalized Values (A and B Sets) | Responses

AT7867

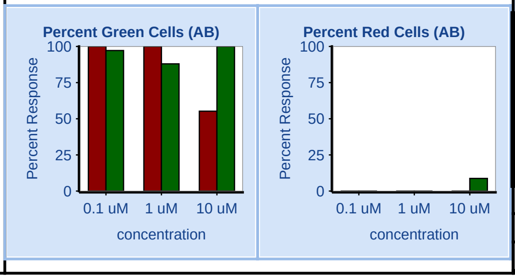

Average Response

Dose	Green Signal	Actual Pct Green Cells	Normalized Pct Green Cells	Red Signal	Actual Pct Red Cells	Normalized Pct Red Cells
0.1 uM	112.58	0.92	125.77	83.61	0.05	-49.86
1 uM	115.57	0.89	103.38	96.07	0.07	-25.17
10 uM	109.92	0.85	87.58	117.19	0.13	3.35

Compound | Normalized Values (A and B Sets) | Responses

AT9283

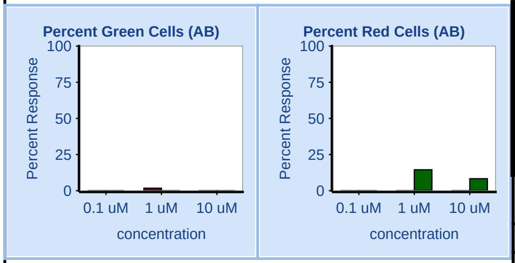

Average Response

Dose	Green Signal	Actual Pct Green Cells	Normalized Pct Green Cells	Red Signal	Actual Pct Red Cells	Normalized Pct Red Cells
0.1 uM	62.85	0.44	-428.51	83.51	0.06	-43.19
1 uM	92.65	0.78	-0.71	112.89	0.12	-3.71
10 uM	90.19	0.63	-64.60	103.17	0.10	-9.12

Compound | Normalized Values (A and B Sets) | Responses

AT13387

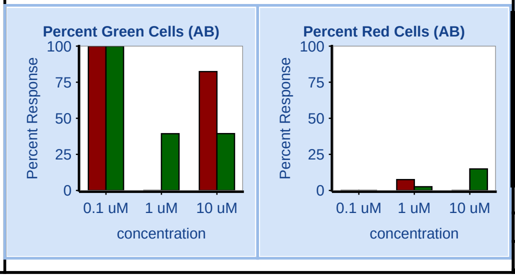

Average Response

Dose	Green Signal	Actual Pct Green Cells	Normalized Pct Green Cells	Red Signal	Actual Pct Red Cells	Normalized Pct Red Cells
0.1 uM	118.03	0.94	133.17	95.54	0.08	-31.62
1 uM	114.72	0.79	-17.73	107.47	0.14	5.07
10 uM	106.75	0.82	60.88	113.74	0.12	2.09

Compound | Normalized Values (A and B Sets) | Responses

Ataluren (PTC124)

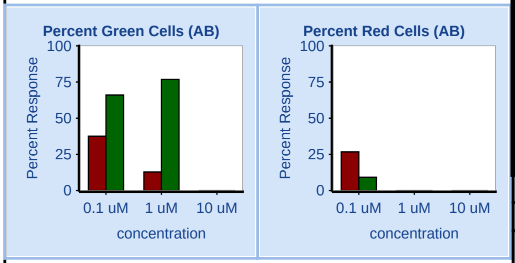

Average Response

Dose	Green Signal	Actual Pct Green Cells	Normalized Pct Green Cells	Red Signal	Actual Pct Red Cells	Normalized Pct Red Cells
0.1 uM	95.97	0.87	51.78	109.74	0.16	17.88
1 uM	92.94	0.78	44.80	80.05	0.05	-42.52
10 uM	76.13	0.59	-227.38	84.29	0.06	-31.45

Compound	Normalized Values (A and B Sets)	Responses

Atazanavir Sulfate

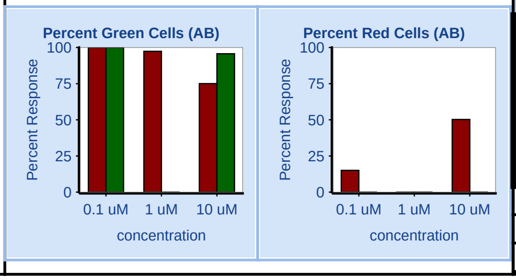

Average Response						
Dose	Green Signal	Actual Pct Green Cells	Normalized Pct Green Cells	Red Signal	Actual Pct Red Cells	Normalized Pct Red Cells
0.1 uM	108.38	0.91	108.24	111.59	0.14	0.12
1 uM	99.00	0.79	31.61	96.88	0.09	-17.80
10 uM	107.42	0.85	85.37	131.17	0.18	18.19

Compound	Normalized Values (A and B Sets)	Responses

Atorvastatin Calcium

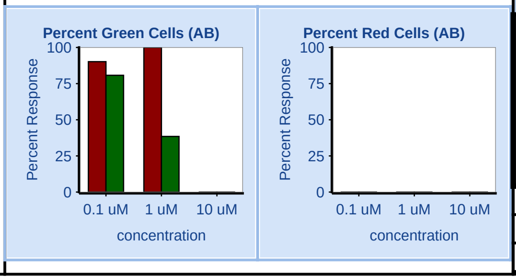

Average Response						
Dose	Green Signal	Actual Pct Green Cells	Normalized Pct Green Cells	Red Signal	Actual Pct Red Cells	Normalized Pct Red Cells
0.1 uM	101.28	0.88	85.49	85.69	0.04	-52.10
1 uM	99.92	0.85	69.91	93.07	0.05	-32.25
10 uM	89.70	0.62	-72.24	89.32	0.08	-16.88

Compound	Normalized Values (A and B Sets)	Responses

Atracurium Besylate

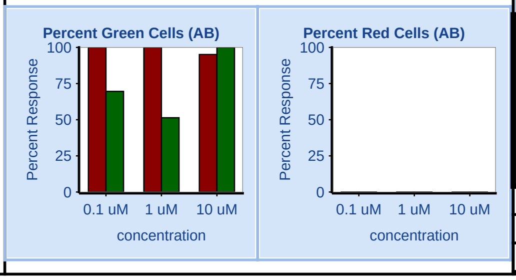

Average Response						
Dose	Green Signal	Actual Pct Green Cells	Normalized Pct Green Cells	Red Signal	Actual Pct Red Cells	Normalized Pct Red Cells
0.1 uM	107.75	0.91	131.70	75.08	0.03	-52.39
1 uM	111.04	0.89	124.74	93.49	0.06	-30.91
10 uM	107.24	0.87	97.84	106.37	0.08	-21.33

Compound	Normalized Values (A and B Sets)	Responses

Atropine

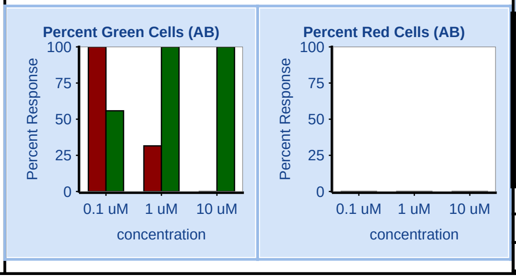

Average Response						
Dose	Green Signal	Actual Pct Green Cells	Normalized Pct Green Cells	Red Signal	Actual Pct Red Cells	Normalized Pct Red Cells
0.1 uM	93.97	0.89	91.85	85.45	0.06	-26.10
1 uM	99.11	0.84	80.76	92.37	0.08	-21.83
10 uM	91.41	0.90	37.23	102.75	0.08	-20.42

Compound	Normalized Values (A and B Sets)	Responses

Aurora A Inhibitor I

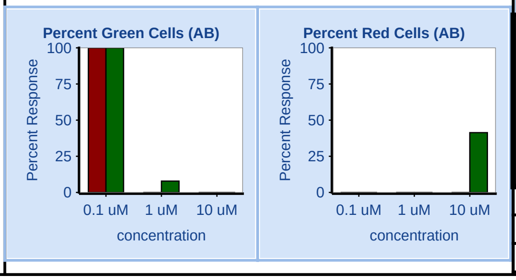

Average Response						
Dose	Green Signal	Actual Pct Green Cells	Normalized Pct Green Cells	Red Signal	Actual Pct Red Cells	Normalized Pct Red Cells
0.1 uM	117.30	0.95	137.35	101.85	0.09	-22.27
1 uM	93.47	0.78	-15.00	89.79	0.06	-32.27
10 uM	109.98	0.62	-68.57	110.38	0.16	16.52

Compound | Normalized Values (A and B Sets) | Responses

AUY922 (NVP-AUY922)

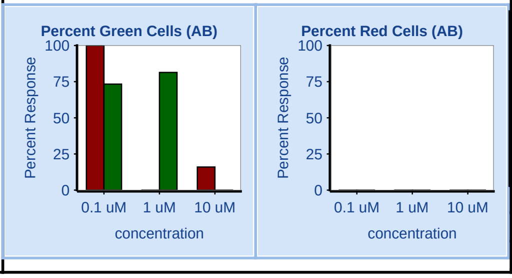

Average Response

Dose	Green Signal	Actual Pct Green Cells	Normalized Pct Green Cells	Red Signal	Actual Pct Red Cells	Normalized Pct Red Cells
0.1 uM	128.43	0.91	124.41	94.48	0.11	-15.86
1 uM	105.70	0.82	-12.54	106.68	0.12	-4.39
10 uM	94.35	0.73	-1.04	110.93	0.09	-14.32

Compound | Normalized Values (A and B Sets) | Responses

Avagacestat (BMS-708163)

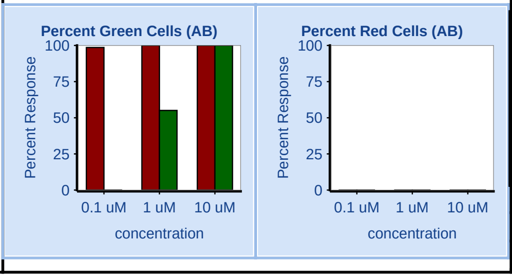

Average Response

Dose	Green Signal	Actual Pct Green Cells	Normalized Pct Green Cells	Red Signal	Actual Pct Red Cells	Normalized Pct Red Cells
0.1 uM	84.84	0.74	4.39	79.65	0.03	-57.93
1 uM	96.19	0.86	78.32	92.64	0.04	-37.87
10 uM	105.10	0.90	113.24	96.87	0.04	-36.85

Compound | Normalized Values (A and B Sets) | Responses

Avanafil

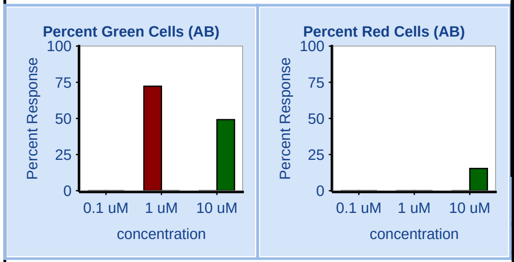

Average Response

Dose	Green Signal	Actual Pct Green Cells	Normalized Pct Green Cells	Red Signal	Actual Pct Red Cells	Normalized Pct Red Cells
0.1 uM	76.05	0.69	-148.77	59.56	0.01	-59.41
1 uM	87.72	0.75	17.74	62.71	0.01	-64.85
10 uM	85.49	0.78	0.46	75.08	0.07	-26.66

Compound | Normalized Values (A and B Sets) | Responses

Avasimibe

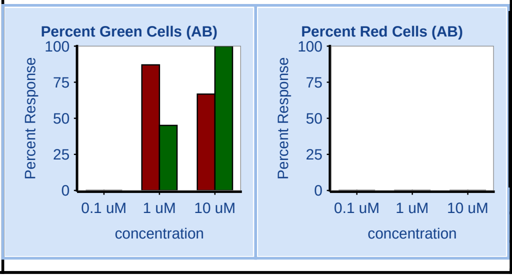

Average Response

Dose	Green Signal	Actual Pct Green Cells	Normalized Pct Green Cells	Red Signal	Actual Pct Red Cells	Normalized Pct Red Cells
0.1 uM	80.98	0.74	-51.83	68.31	0.02	-47.89
1 uM	99.62	0.84	66.05	74.12	0.03	-42.60
10 uM	95.28	0.91	98.78	93.68	0.07	-26.33

Compound | Normalized Values (A and B Sets) | Responses

AVL-292

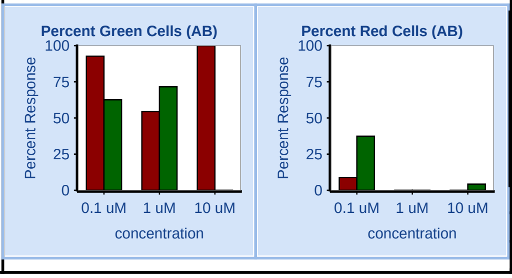

Average Response

Dose	Green Signal	Actual Pct Green Cells	Normalized Pct Green Cells	Red Signal	Actual Pct Red Cells	Normalized Pct Red Cells
0.1 uM	94.62	0.87	77.66	112.03	0.17	23.13
1 uM	95.19	0.81	62.97	95.14	0.09	-21.85
10 uM	80.07	0.68	46.44	91.74	0.07	-25.05

Compound | Normalized Values (A and B Sets) | Responses

AZ 23

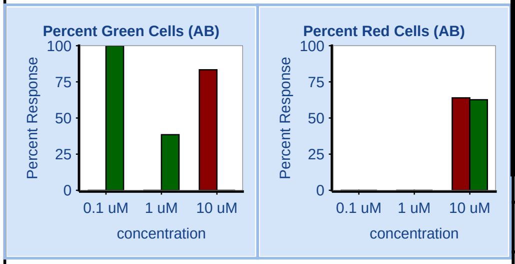

Average Response						
Dose	Green Signal	Actual Pct Green Cells	Normalized Pct Green Cells	Red Signal	Actual Pct Red Cells	Normalized Pct Red Cells
0.1 uM	90.49	0.81	25.28	88.24	0.06	-40.98
1 uM	83.23	0.78	-480.67	76.70	0.05	-31.70
10 uM	94.16	0.79	-18.50	119.24	0.26	63.28

Compound | Normalized Values (A and B Sets) | Responses

AZ 628

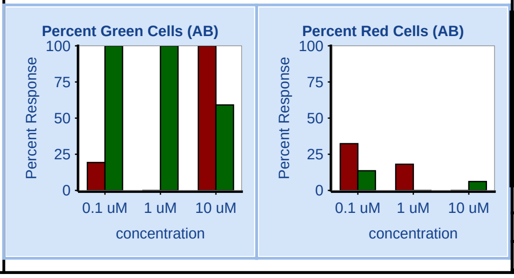

Average Response						
Dose	Green Signal	Actual Pct Green Cells	Normalized Pct Green Cells	Red Signal	Actual Pct Red Cells	Normalized Pct Red Cells
0.1 uM	91.28	0.86	66.51	107.41	0.17	22.96
1 uM	89.18	0.79	54.39	106.75	0.14	5.54
10 uM	105.68	0.89	96.34	87.30	0.10	-12.96

Compound | Normalized Values (A and B Sets) | Responses

AZ 960

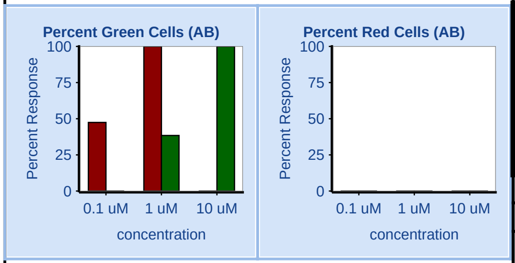

Average Response						
Dose	Green Signal	Actual Pct Green Cells	Normalized Pct Green Cells	Red Signal	Actual Pct Red Cells	Normalized Pct Red Cells
0.1 uM	83.33	0.80	2.32	66.93	0.01	-47.91
1 uM	108.90	0.87	82.51	81.22	0.05	-32.42
10 uM	96.99	0.80	-838.70	84.11	0.06	-30.02

Compound | Normalized Values (A and B Sets) | Responses

AZ 3146

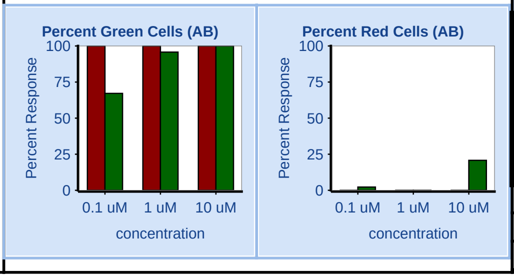

Average Response						
Dose	Green Signal	Actual Pct Green Cells	Normalized Pct Green Cells	Red Signal	Actual Pct Red Cells	Normalized Pct Red Cells
0.1 uM	100.73	0.90	106.47	85.80	0.07	-19.94
1 uM	104.30	0.91	105.15	82.44	0.04	-38.41
10 uM	110.36	0.94	261.31	92.40	0.09	-16.19

Compound | Normalized Values (A and B Sets) | Responses

AZ191

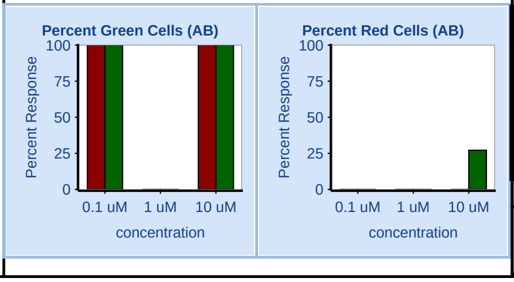

Average Response						
Dose	Green Signal	Actual Pct Green Cells	Normalized Pct Green Cells	Red Signal	Actual Pct Red Cells	Normalized Pct Red Cells
0.1 uM	98.46	0.90	112.23	69.20	0.02	-65.60
1 uM	68.97	0.55		60.69	0.01	-44.10

Compound | Normalized Values (A and B Sets) | Responses

			-1703.81			
10 uM	107.89	0.93	108.69	93.40	0.11	-4.63

Compound | Normalized Values (A and B Sets) | Responses

Azatadine dimaleate

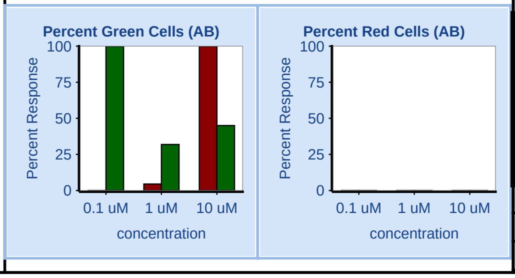

Average Response

Dose	Green Signal	Actual Pct Green Cells	Normalized Pct Green Cells	Red Signal	Actual Pct Red Cells	Normalized Pct Red Cells
0.1 uM	90.02	0.87	-10.04	63.06	0.01	-60.28
1 uM	84.79	0.74	18.19	76.35	0.03	-52.34
10 uM	92.78	0.84	102.23	68.10	0.02	-52.95

Compound | Normalized Values (A and B Sets) | Responses

Azathioprine

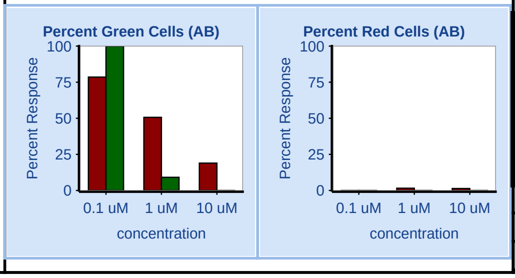

Average Response

Dose	Green Signal	Actual Pct Green Cells	Normalized Pct Green Cells	Red Signal	Actual Pct Red Cells	Normalized Pct Red Cells
0.1 uM	101.84	0.90	93.51	105.58	0.10	-21.50
1 uM	100.02	0.81	29.89	104.77	0.10	-13.57
10 uM	81.26	0.65	-56.30	99.01	0.07	-25.58

Compound | Normalized Values (A and B Sets) | Responses

AZD1080

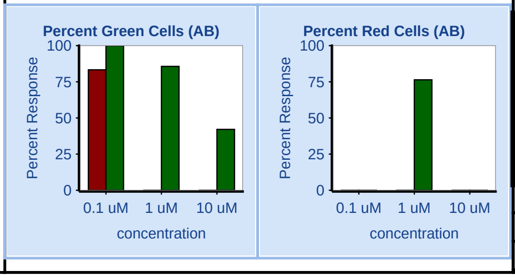

Average Response

Dose	Green Signal	Actual Pct Green Cells	Normalized Pct Green Cells	Red Signal	Actual Pct Red Cells	Normalized Pct Red Cells
0.1 uM	101.98	0.91	102.30	76.26	0.05	-39.84
1 uM	96.35	0.71	-3.09	108.58	0.18	24.03
10 uM	92.20	0.74	-46.13	85.16	0.06	-30.64

Compound | Normalized Values (A and B Sets) | Responses

AZD1480

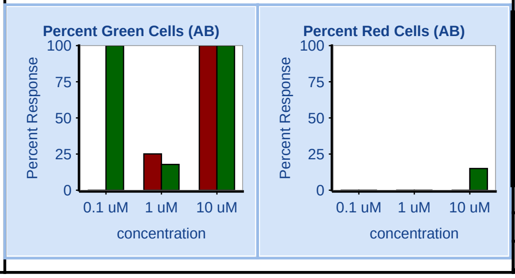

Average Response

Dose	Green Signal	Actual Pct Green Cells	Normalized Pct Green Cells	Red Signal	Actual Pct Red Cells	Normalized Pct Red Cells
0.1 uM	91.93	0.84	48.41	97.27	0.08	-17.81
1 uM	88.62	0.74	21.45	89.84	0.05	-34.95
10 uM	103.09	0.94	400.81	98.48	0.10	-12.84

Compound | Normalized Values (A and B Sets) | Responses

AZD1981

Average Response

Dose	Green Signal			Red Signal	Actual Pct Red Cells	

Compound	Normalized Values (A and B Sets)	Responses

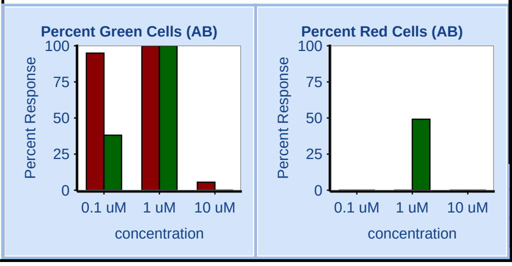

		Actual Pct Green Cells	Normalized Pct Green Cells			Normalized Pct Red Cells
0.1 uM	92.69	0.85	66.53	69.16	0.01	-69.55
1 uM	106.98	0.94	242.62	75.11	0.10	10.25
10 uM	83.14	0.80	-14.18	64.46	0.02	-41.32

Compound	Normalized Values (A and B Sets)	Responses

AZD2014

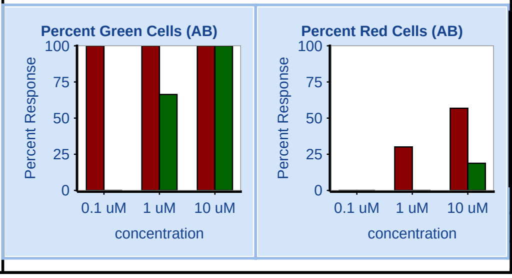

Average Response

Dose	Green Signal	Actual Pct Green Cells	Normalized Pct Green Cells	Red Signal	Actual Pct Red Cells	Normalized Pct Red Cells
0.1 uM	92.72	0.82	6.62	94.81	0.08	-15.13
1 uM	96.52	0.89	91.59	106.06	0.12	-2.08
10 uM	102.03	0.95	455.92	125.20	0.20	37.79

Compound	Normalized Values (A and B Sets)	Responses

AZD2461

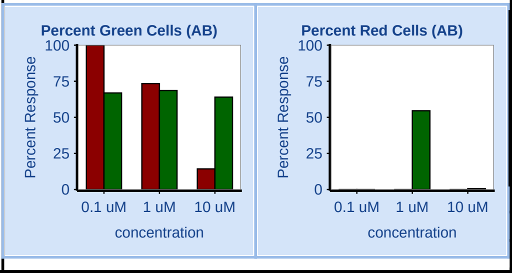

Average Response

Dose	Green Signal	Actual Pct Green Cells	Normalized Pct Green Cells	Red Signal	Actual Pct Red Cells	Normalized Pct Red Cells
0.1 uM	92.32	0.88	92.40	75.10	0.03	-47.63
1 uM	96.76	0.82	70.99	106.05	0.16	11.36
10 uM	90.87	0.81	39.16	100.71	0.10	-9.99

Compound	Normalized Values (A and B Sets)	Responses

AZD2858

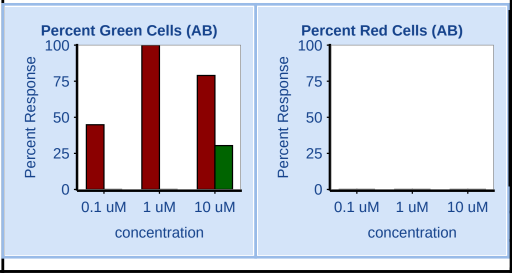

Average Response

Dose	Green Signal	Actual Pct Green Cells	Normalized Pct Green Cells	Red Signal	Actual Pct Red Cells	Normalized Pct Red Cells
0.1 uM	88.58	0.80	18.52	76.03	0.01	-66.87
1 uM	96.42	0.82	32.30	73.59	0.03	-36.33
10 uM	100.04	0.87	54.68	81.82	0.05	-26.98

Compound	Normalized Values (A and B Sets)	Responses

AZD3463

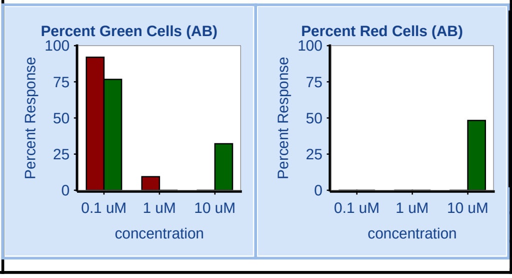

Average Response

Dose	Green Signal	Actual Pct Green Cells	Normalized Pct Green Cells	Red Signal	Actual Pct Red Cells	Normalized Pct Red Cells
0.1 uM	99.67	0.88	84.34	75.72	0.04	-41.63
1 uM	81.17	0.63	-68.75	94.41	0.09	-20.87
10 uM	98.10	0.72	-64.39	90.29	0.13	2.43

Compound | Normalized Values (A and B Sets) | Responses

AZD3514

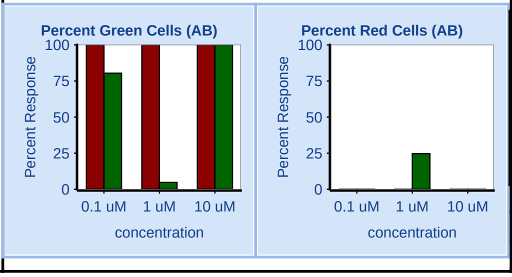

Average Response

Dose	Green Signal	Actual Pct Green Cells	Normalized Pct Green Cells	Red Signal	Actual Pct Red Cells	Normalized Pct Red Cells
0.1 uM	100.49	0.91	210.26	83.62	0.05	-40.37
1 uM	95.78	0.81	64.35	105.30	0.13	-4.30
10 uM	101.51	0.92	160.85	84.12	0.05	-37.76

Compound | Normalized Values (A and B Sets) | Responses

AZD4547

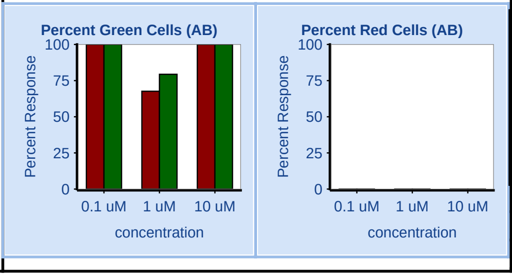

Average Response

Dose	Green Signal	Actual Pct Green Cells	Normalized Pct Green Cells	Red Signal	Actual Pct Red Cells	Normalized Pct Red Cells
0.1 uM	101.86	0.92	121.26	85.94	0.06	-27.44
1 uM	96.45	0.84	73.53	87.18	0.06	-29.56
10 uM	126.26	0.99	582.46	82.78	0.03	-45.40

Compound | Normalized Values (A and B Sets) | Responses

AZD5363

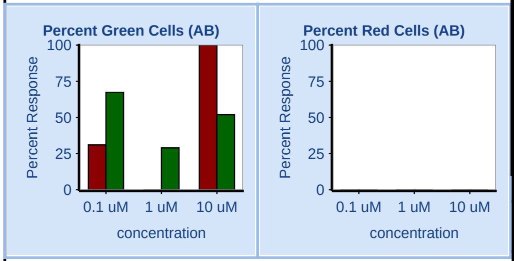

Average Response

Dose	Green Signal	Actual Pct Green Cells	Normalized Pct Green Cells	Red Signal	Actual Pct Red Cells	Normalized Pct Red Cells
0.1 uM	91.20	0.83	49.16	83.32	0.04	-46.87
1 uM	79.57	0.74	-703.67	70.92	0.01	-42.63
10 uM	94.27	0.90	89.68	67.83	0.03	-37.05

Compound | Normalized Values (A and B Sets) | Responses

AZD5438

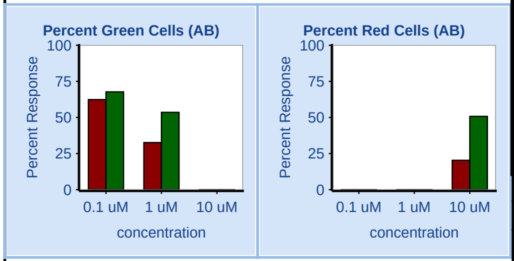

Average Response

Dose	Green Signal	Actual Pct Green Cells	Normalized Pct Green Cells	Red Signal	Actual Pct Red Cells	Normalized Pct Red Cells
0.1 uM	91.64	0.86	65.07	81.19	0.04	-34.86
1 uM	94.62	0.78	43.09	81.29	0.05	-34.03
10 uM	98.97	0.74	-803.96	112.34	0.20	35.54

Compound | Normalized Values (A and B Sets) | Responses

AZD6482

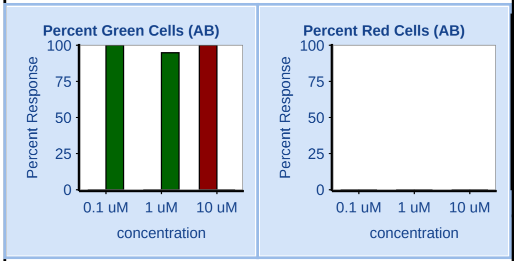

Average Response

Dose	Green Signal	Actual Pct Green Cells	Normalized Pct Green Cells	Red Signal	Actual Pct Red Cells	Normalized Pct Red Cells
0.1 uM	88.40	0.78	-12.61	74.20	0.02	-65.98
1 uM	93.78	0.85	-216.91	84.48	0.05	-27.68

Compound | Normalized Values (A and B Sets) | Responses

10 uM	89.61	0.87	60.56	85.21	0.05	-27.26

Compound | Normalized Values (A and B Sets) | Responses

AZD8055

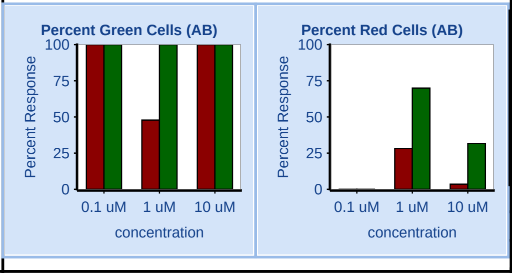

Average Response

Dose	Green Signal	Actual Pct Green Cells	Normalized Pct Green Cells	Red Signal	Actual Pct Red Cells	Normalized Pct Red Cells
0.1 uM	106.78	0.95	148.13	83.31	0.05	-45.83
1 uM	98.62	0.90	86.79	137.19	0.23	49.08
10 uM	115.39	0.92	131.83	126.57	0.16	17.53

Compound | Normalized Values (A and B Sets) | Responses

AZD8330

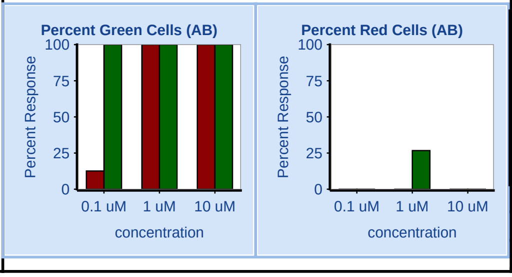

Average Response

Dose	Green Signal	Actual Pct Green Cells	Normalized Pct Green Cells	Red Signal	Actual Pct Red Cells	Normalized Pct Red Cells
0.1 uM	96.34	0.90	116.37	80.27	0.05	-29.29
1 uM	113.47	0.93	115.96	97.81	0.11	-7.63
10 uM	99.92	0.94	349.93	77.97	0.05	-36.61

Compound | Normalized Values (A and B Sets) | Responses

AZD8931 (Sapitinib)

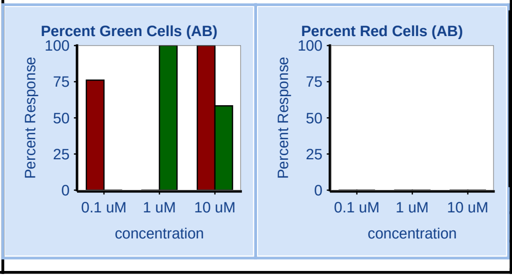

Average Response

Dose	Green Signal	Actual Pct Green Cells	Normalized Pct Green Cells	Red Signal	Actual Pct Red Cells	Normalized Pct Red Cells
0.1 uM	86.06	0.82	18.14	77.22	0.03	-42.19
1 uM	90.50	0.76	43.25	87.23	0.06	-30.25
10 uM	92.13	0.90	204.55	89.13	0.06	-30.01

Compound | Normalized Values (A and B Sets) | Responses

Azelnidipine

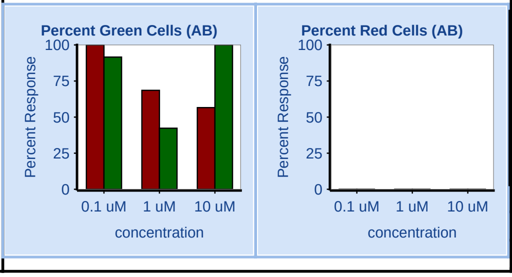

Average Response

Dose	Green Signal	Actual Pct Green Cells	Normalized Pct Green Cells	Red Signal	Actual Pct Red Cells	Normalized Pct Red Cells
0.1 uM	101.09	0.90	119.52	85.47	0.07	-30.34
1 uM	96.52	0.80	55.42	81.64	0.04	-44.44
10 uM	99.34	0.89	91.46	92.54	0.07	-24.68

Compound | Normalized Values (A and B Sets) | Responses

Azilsartan

Average Response

Dose	Green Signal	Actual Pct Green Cells	Normalized Pct Green Cells	Red Signal	Actual Pct Red Cells	Normalized Pct Red Cells

Compound	Normalized Values (A and B Sets)	Responses

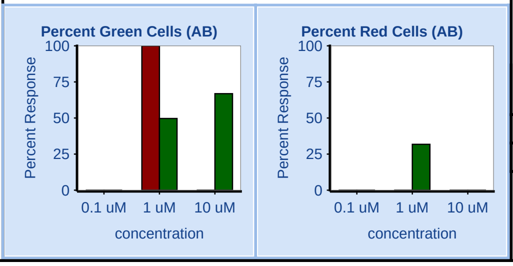

0.1 uM	85.73	0.77	-184.01	83.42	0.05	-37.16
1 uM	104.54	0.85	93.81	104.08	0.13	-2.37
10 uM	90.50	0.80	12.70	81.11	0.03	-46.92

Compound	Normalized Values (A and B Sets)	Responses

Azilsartan Medoxomil

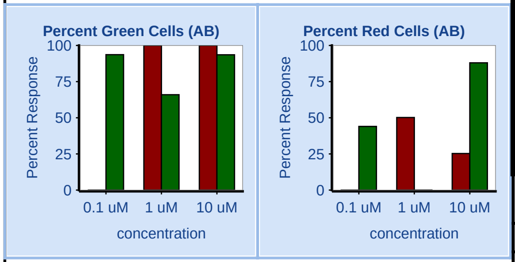

Average Response

Dose	Green Signal	Actual Pct Green Cells	Normalized Pct Green Cells	Red Signal	Actual Pct Red Cells	Normalized Pct Red Cells
0.1 uM	88.05	0.82	-214.56	85.17	0.11	-8.39
1 uM	106.67	0.87	104.69	114.50	0.17	23.90
10 uM	103.24	0.88	103.62	124.62	0.23	56.65

Compound	Normalized Values (A and B Sets)	Responses

Azithromycin

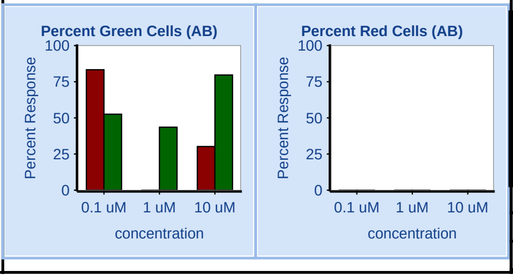

Average Response

Dose	Green Signal	Actual Pct Green Cells	Normalized Pct Green Cells	Red Signal	Actual Pct Red Cells	Normalized Pct Red Cells
0.1 uM	98.78	0.86	67.93	89.63	0.05	-47.58
1 uM	96.30	0.81	10.28	90.59	0.05	-33.38
10 uM	93.60	0.80	54.93	88.28	0.04	-36.67

Compound	Normalized Values (A and B Sets)	Responses

Azithromycin Dihydrate

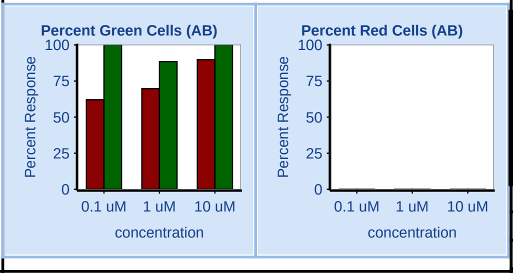

Average Response

Dose	Green Signal	Actual Pct Green Cells	Normalized Pct Green Cells	Red Signal	Actual Pct Red Cells	Normalized Pct Red Cells
0.1 uM	103.38	0.91	92.41	79.18	0.04	-41.38
1 uM	95.96	0.83	79.08	89.13	0.05	-38.64
10 uM	98.78	0.88	98.78	67.60	0.02	-56.00

Compound	Normalized Values (A and B Sets)	Responses

Azlocillin sodium salt

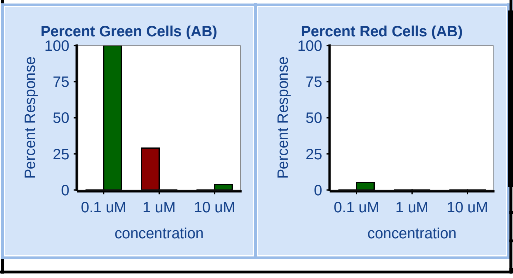

Average Response

Dose	Green Signal	Actual Pct Green Cells	Normalized Pct Green Cells	Red Signal	Actual Pct Red Cells	Normalized Pct Red Cells
0.1 uM	99.80	0.92	68.49	74.83	0.07	-28.01
1 uM	86.31	0.71	-7.50	66.66	0.02	-56.43
10 uM	85.68	0.74	-5.56	70.84	0.02	-52.06

Compound | Normalized Values (A and B Sets) | Responses

Azomycin

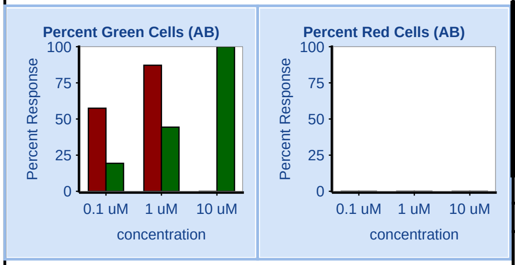

Average Response						
Dose	Green Signal	Actual Pct Green Cells	Normalized Pct Green Cells	Red Signal	Actual Pct Red Cells	Normalized Pct Red Cells
0.1 uM	87.28	0.84	38.42	84.75	0.04	-35.83
1 uM	96.39	0.84	65.78	99.45	0.10	-12.60
10 uM	95.13	0.87	-177.13	75.36	0.02	-48.07

Compound | Normalized Values (A and B Sets) | Responses

Bacitracin

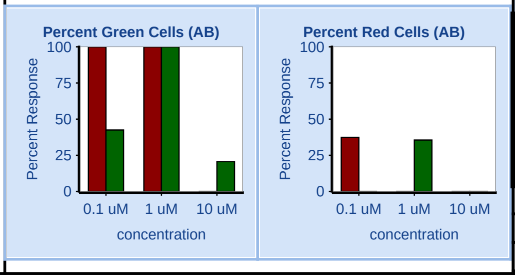

Average Response						
Dose	Green Signal	Actual Pct Green Cells	Normalized Pct Green Cells	Red Signal	Actual Pct Red Cells	Normalized Pct Red Cells
0.1 uM	96.89	0.87	123.78	108.43	0.16	14.28
1 uM	109.01	0.90	134.27	105.08	0.13	-0.40
10 uM	94.67	0.76	2.09	83.56	0.05	-35.36

Compound | Normalized Values (A and B Sets) | Responses

Bafetinib (INNO-406)

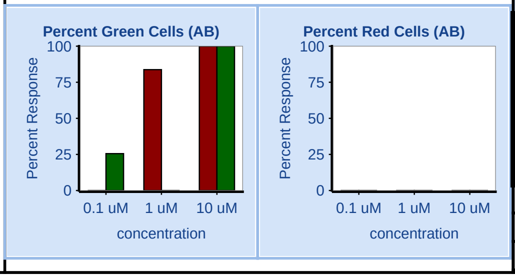

Average Response						
Dose	Green Signal	Actual Pct Green Cells	Normalized Pct Green Cells	Red Signal	Actual Pct Red Cells	Normalized Pct Red Cells
0.1 uM	87.37	0.80	8.98	88.45	0.06	-42.97
1 uM	89.54	0.80	35.14	84.08	0.05	-34.87
10 uM	99.35	0.92	126.62	123.41	0.08	-18.14

Compound | Normalized Values (A and B Sets) | Responses

Baicalin

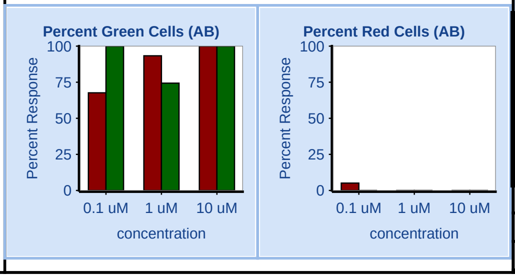

Average Response						
Dose	Green Signal	Actual Pct Green Cells	Normalized Pct Green Cells	Red Signal	Actual Pct Red Cells	Normalized Pct Red Cells
0.1 uM	97.31	0.89	94.81	93.28	0.09	-15.38
1 uM	103.95	0.87	83.84	80.66	0.04	-38.91
10 uM	110.07	0.95	330.42	90.39	0.06	-30.97

Compound | Normalized Values (A and B Sets) | Responses

Balofloxacin

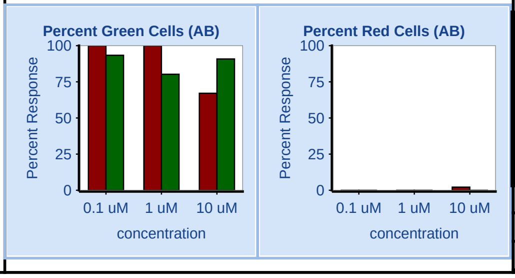

Average Response						
Dose	Green Signal	Actual Pct Green Cells	Normalized Pct Green Cells	Red Signal	Actual Pct Red Cells	Normalized Pct Red Cells
0.1 uM	107.38	0.91	116.62	94.73	0.05	-45.47
1 uM	105.31	0.88	92.01	90.68	0.04	-36.87
10 uM	100.03	0.84	78.97	119.58	0.12	-3.38

Compound	Normalized Values (A and B Sets)	Responses
**BAM7**	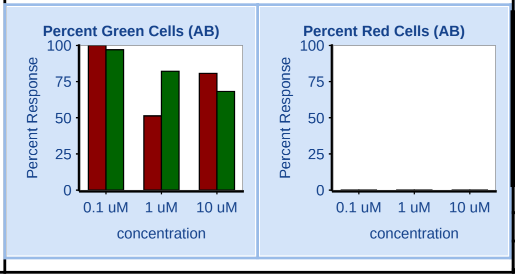	see table below

Average Response

Dose	Green Signal	Actual Pct Green Cells	Normalized Pct Green Cells	Red Signal	Actual Pct Red Cells	Normalized Pct Red Cells
0.1 uM	103.77	0.92	227.70	81.65	0.06	-31.81
1 uM	94.11	0.81	66.78	77.07	0.02	-57.75
10 uM	94.25	0.84	74.49	90.35	0.06	-31.25

Compound	Normalized Values (A and B Sets)	Responses
**Banoxantrone dihydrochloride**	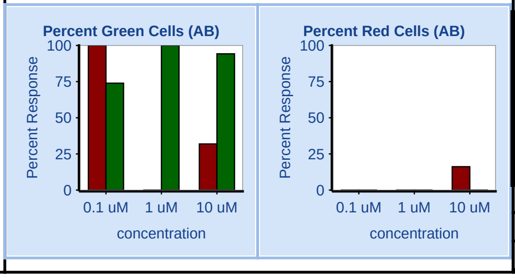	see table below

Average Response

Dose	Green Signal	Actual Pct Green Cells	Normalized Pct Green Cells	Red Signal	Actual Pct Red Cells	Normalized Pct Red Cells
0.1 uM	95.47	0.88	90.51	75.29	0.03	-55.23
1 uM	99.13	0.90	-21.66	72.01	0.03	-36.03
10 uM	101.18	0.88	63.07	92.34	0.11	-1.59

Compound	Normalized Values (A and B Sets)	Responses
**Barasertib (AZD1152-HQPA)**	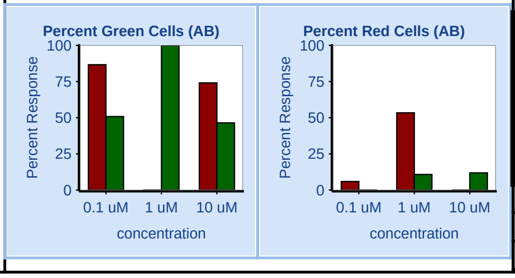	see table below

Average Response

Dose	Green Signal	Actual Pct Green Cells	Normalized Pct Green Cells	Red Signal	Actual Pct Red Cells	Normalized Pct Red Cells
0.1 uM	93.46	0.86	68.75	100.74	0.10	-27.22
1 uM	100.03	0.84	-1.59	129.19	0.20	32.09
10 uM	97.86	0.82	60.28	109.34	0.12	0.74

Compound	Normalized Values (A and B Sets)	Responses
**Bardoxolone Methyl**	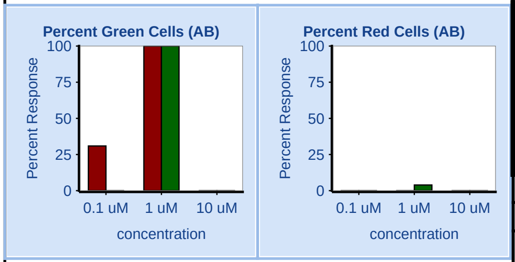	see table below

Average Response

Dose	Green Signal	Actual Pct Green Cells	Normalized Pct Green Cells	Red Signal	Actual Pct Red Cells	Normalized Pct Red Cells
0.1 uM	86.11	0.78	2.67	78.03	0.02	-60.76
1 uM	104.47	0.95	313.29	91.54	0.09	-3.13
10 uM	86.30	0.69	-117.16	85.38	0.06	-22.73

Compound	Normalized Values (A and B Sets)	Responses
**Batimastat (BB-94)**	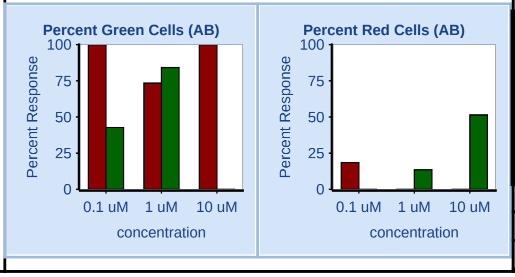	see table below

Average Response

Dose	Green Signal	Actual Pct Green Cells	Normalized Pct Green Cells	Red Signal	Actual Pct Red Cells	Normalized Pct Red Cells
0.1 uM	89.58	0.86	75.39	102.11	0.12	-1.74
1 uM	97.90	0.83	78.80	88.95	0.09	-21.52
10 uM	92.20	0.76	68.49	113.54	0.16	20.32

Compound	Normalized Values (A and B Sets)	Responses
**Bay 11-7085**	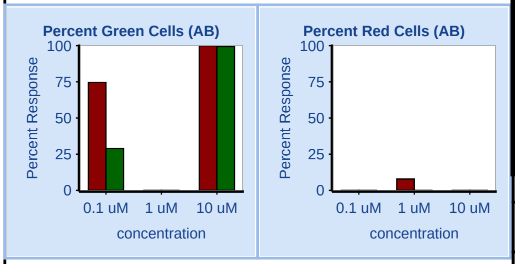	see table below

Average Response

Dose	Green Signal	Actual Pct Green Cells	Normalized Pct Green Cells	Red Signal	Actual Pct Red Cells	Normalized Pct Red Cells
0.1 uM	93.63	0.83	51.77	97.47	0.09	-21.47
1 uM	81.69	0.74	-618.21	91.13	0.09	-10.38
10 uM	106.91	0.93	108.79	81.95	0.07	-20.38

Compound	Normalized Values (A and B Sets)	Responses
**Bazedoxifene HCl**	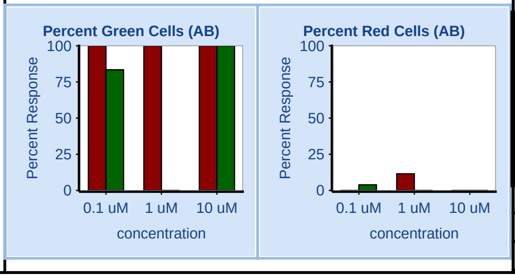	see table below

Average Response

Dose	Green Signal	Actual Pct Green Cells	Normalized Pct Green Cells	Red Signal	Actual Pct Red Cells	Normalized Pct Red Cells
0.1 uM	99.46	0.89	101.87	96.88	0.10	-15.17
1 uM	97.20	0.85	232.24	98.32	0.11	0.87
10 uM	105.73	0.96	143.09	62.85	0.01	-45.95

Compound	Normalized Values (A and B Sets)	Responses
**BD 1047**	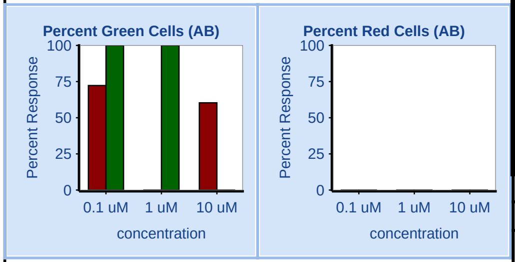	see table below

Average Response

Dose	Green Signal	Actual Pct Green Cells	Normalized Pct Green Cells	Red Signal	Actual Pct Red Cells	Normalized Pct Red Cells
0.1 uM	97.66	0.88	91.03	99.62	0.10	-10.10
1 uM	105.57	0.92	-35.70	84.76	0.07	-14.10
10 uM	73.23	0.63	-157.92	88.19	0.10	-6.28

Compound	Normalized Values (A and B Sets)	Responses
**Bekanamycin**	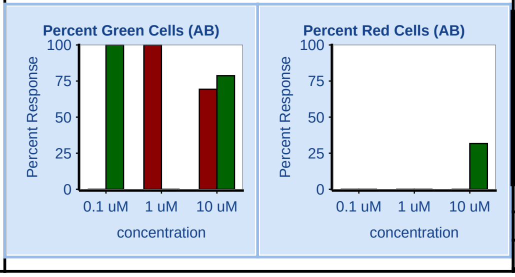	see table below

Average Response

Dose	Green Signal	Actual Pct Green Cells	Normalized Pct Green Cells	Red Signal	Actual Pct Red Cells	Normalized Pct Red Cells
0.1 uM	93.88	0.87	-68.03	90.69	0.10	-14.84
1 uM	85.40	0.67	-48.71	76.44	0.07	-33.44
10 uM	101.54	0.85	74.02	99.47	0.14	10.93

Compound	Normalized Values (A and B Sets)	Responses
**Belinostat (PXD101)**	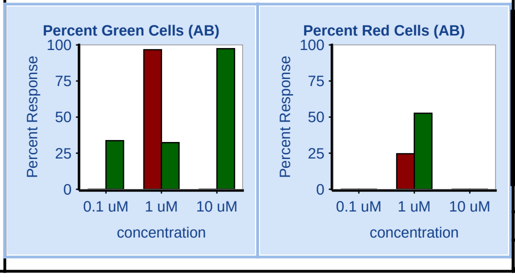	see table below

Average Response

Dose	Green Signal	Actual Pct Green Cells	Normalized Pct Green Cells	Red Signal	Actual Pct Red Cells	Normalized Pct Red Cells
0.1 uM	90.43	0.79	-11.87	90.74	0.05	-46.60
1 uM	104.37	0.84	64.49	146.84	0.21	38.63
10 uM	95.37	0.77	36.95	93.83	0.03	-41.96

Compound	Normalized Values (A and B Sets)	Responses

Benfotiamine

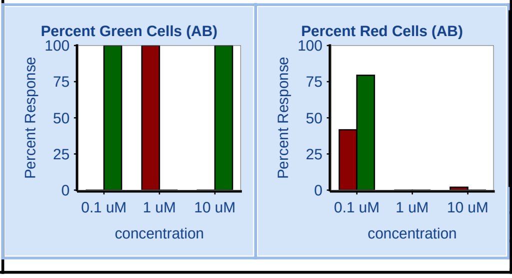

Average Response

Dose	Green Signal	Actual Pct Green Cells	Normalized Pct Green Cells	Red Signal	Actual Pct Red Cells	Normalized Pct Red Cells
0.1 uM	105.79	0.91	12.74	123.31	0.24	60.58
1 uM	89.79	0.72	-8.48	84.11	0.06	-35.15
10 uM	98.70	0.86	55.36	92.01	0.07	-23.79

Compound	Normalized Values (A and B Sets)	Responses

Benidipine HCl

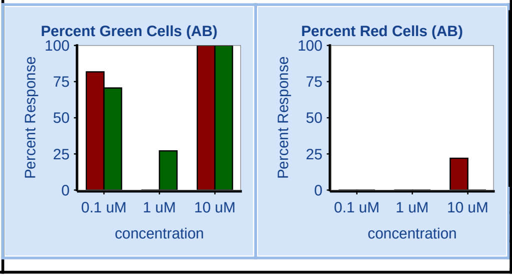

Average Response

Dose	Green Signal	Actual Pct Green Cells	Normalized Pct Green Cells	Red Signal	Actual Pct Red Cells	Normalized Pct Red Cells
0.1 uM	96.69	0.87	76.18	91.48	0.05	-46.19
1 uM	93.29	0.80	-2.97	90.69	0.04	-37.19
10 uM	110.88	0.92	125.31	124.71	0.14	1.15

Compound	Normalized Values (A and B Sets)	Responses

Bentiromide

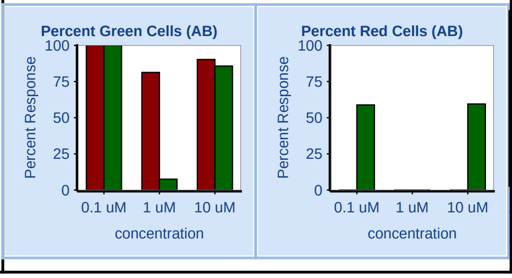

Average Response

Dose	Green Signal	Actual Pct Green Cells	Normalized Pct Green Cells	Red Signal	Actual Pct Red Cells	Normalized Pct Red Cells
0.1 uM	108.98	0.94	144.28	99.97	0.14	9.55
1 uM	94.95	0.78	44.42	73.67	0.03	-54.23
10 uM	104.36	0.86	88.02	110.22	0.16	18.04

Compound	Normalized Values (A and B Sets)	Responses

Bepotastine Besilate

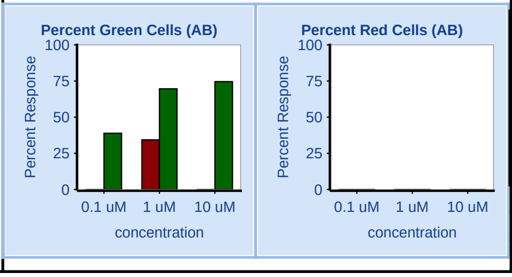

Average Response

Dose	Green Signal	Actual Pct Green Cells	Normalized Pct Green Cells	Red Signal	Actual Pct Red Cells	Normalized Pct Red Cells
0.1 uM	88.14	0.84	11.88	64.59	0.01	-58.92
1 uM	95.36	0.79	51.98	85.87	0.06	-38.35
10 uM	91.45	0.80	9.45	89.95	0.06	-30.57

Compound	Normalized Values (A and B Sets)	Responses

Bergenin

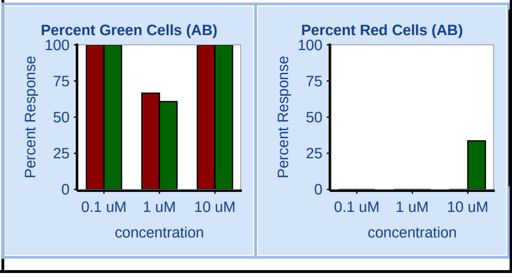

Average Response

Dose	Green Signal	Actual Pct Green Cells	Normalized Pct Green Cells	Red Signal	Actual Pct Red Cells	Normalized Pct Red Cells
0.1 uM	114.69	0.94	153.27	85.48	0.05	-29.89
1 uM	96.42	0.83	63.68	85.01	0.05	-34.20
10 uM	103.68	0.91	135.50	99.27	0.12	-3.48

Compound	Normalized Values (A and B Sets)	Responses

Betamethasone Dipropionate

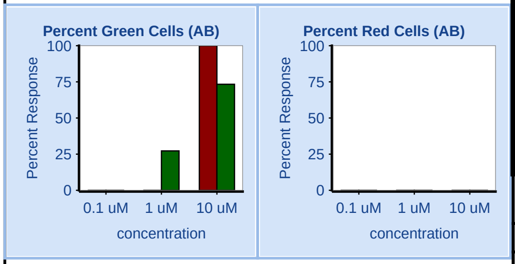

Average Response						
Dose	Green Signal	Actual Pct Green Cells	Normalized Pct Green Cells	Red Signal	Actual Pct Red Cells	Normalized Pct Red Cells
0.1 uM	63.37	0.43	-312.51	66.46	0.01	-67.93
1 uM	88.88	0.85	-26.36	74.56	0.02	-37.51
10 uM	99.22	0.92	98.44	67.74	0.02	-40.76

Compound	Normalized Values (A and B Sets)	Responses

Betamethasone Valerate

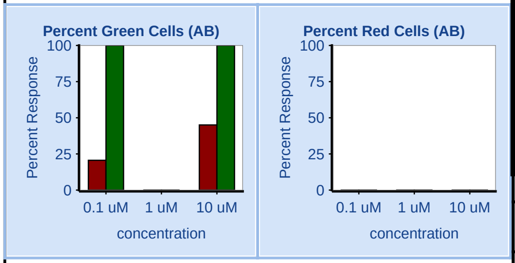

Average Response						
Dose	Green Signal	Actual Pct Green Cells	Normalized Pct Green Cells	Red Signal	Actual Pct Red Cells	Normalized Pct Red Cells
0.1 uM	107.07	0.88	86.18	75.72	0.03	-55.07
1 uM	81.30	0.76	-413.46	67.15	0.01	-42.96
10 uM	95.64	0.91	94.09	59.10	0.01	-46.53

Compound	Normalized Values (A and B Sets)	Responses

Betaxolol

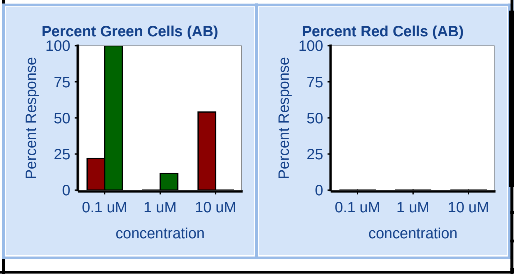

Average Response						
Dose	Green Signal	Actual Pct Green Cells	Normalized Pct Green Cells	Red Signal	Actual Pct Red Cells	Normalized Pct Red Cells
0.1 uM	108.65	0.90	77.24	91.36	0.05	-45.51
1 uM	97.58	0.79	3.50	103.70	0.10	-13.22
10 uM	92.42	0.76	16.89	109.70	0.09	-16.99

Compound	Normalized Values (A and B Sets)	Responses

Betulinic acid

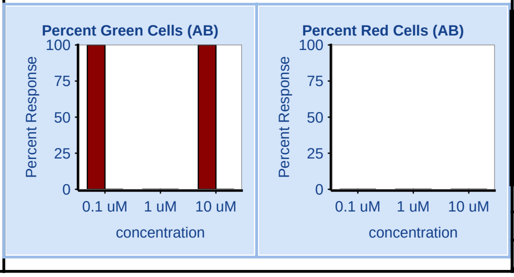

Average Response						
Dose	Green Signal	Actual Pct Green Cells	Normalized Pct Green Cells	Red Signal	Actual Pct Red Cells	Normalized Pct Red Cells
0.1 uM	85.22	0.80	105.28	81.10	0.03	-47.54
1 uM	80.75	0.65	-48.81	78.68	0.03	-50.72
10 uM	89.44	0.78	90.03	66.84	0.01	-61.16

Compound	Normalized Values (A and B Sets)	Responses

BGJ398 (NVP-BGJ398)

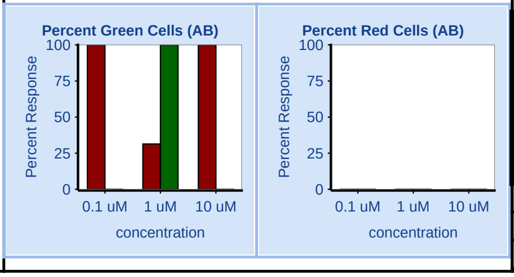

Average Response						
Dose	Green Signal	Actual Pct Green Cells	Normalized Pct Green Cells	Red Signal	Actual Pct Red Cells	Normalized Pct Red Cells
0.1 uM	92.86	0.83	22.35	80.79	0.05	-29.61
1 uM	94.98	0.82	68.87	103.73	0.10	-12.22
10 uM	91.11	0.87	185.02	73.79	0.02	-50.19

Compound | Normalized Values (A and B Sets) | Responses

BGT226 (NVP-BGT226)

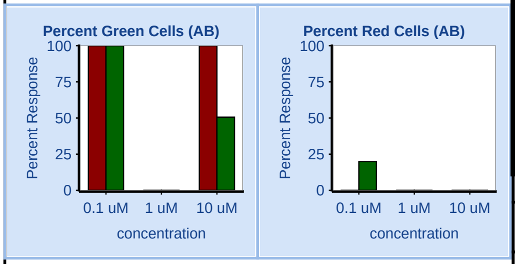

Average Response

Dose	Green Signal	Actual Pct Green Cells	Normalized Pct Green Cells	Red Signal	Actual Pct Red Cells	Normalized Pct Red Cells
0.1 uM	104.85	0.94	142.21	102.16	0.12	2.54
1 uM	62.47	0.38	-148.88	68.78	0.05	-34.74
10 uM	101.29	0.93	478.09	92.14	0.04	-37.81

Compound | Normalized Values (A and B Sets) | Responses

BI 78D3

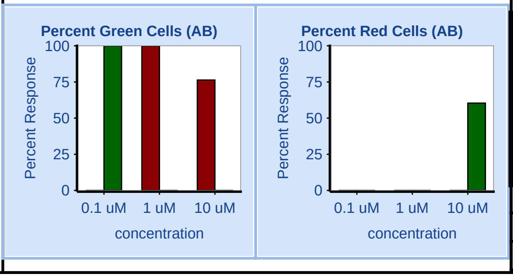

Average Response

Dose	Green Signal	Actual Pct Green Cells	Normalized Pct Green Cells	Red Signal	Actual Pct Red Cells	Normalized Pct Red Cells
0.1 uM	93.02	0.82	28.72	90.93	0.07	-31.81
1 uM	92.27	0.85	195.72	87.80	0.05	-20.48
10 uM	85.81	0.84	26.79	96.48	0.14	9.64

Compound | Normalized Values (A and B Sets) | Responses

BI-D1870

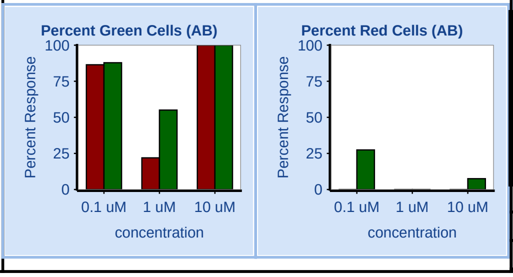

Average Response

Dose	Green Signal	Actual Pct Green Cells	Normalized Pct Green Cells	Red Signal	Actual Pct Red Cells	Normalized Pct Red Cells
0.1 uM	100.62	0.88	87.14	103.61	0.13	6.84
1 uM	88.66	0.77	38.50	75.83	0.02	-47.47
10 uM	123.08	0.99	569.62	108.08	0.12	-1.10

Compound | Normalized Values (A and B Sets) | Responses

BIBR 1532

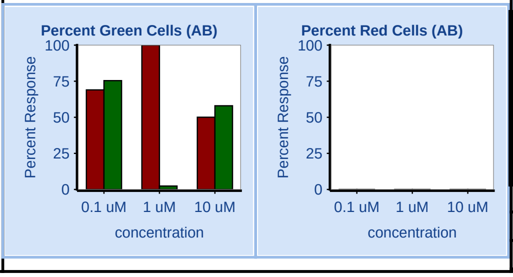

Average Response

Dose	Green Signal	Actual Pct Green Cells	Normalized Pct Green Cells	Red Signal	Actual Pct Red Cells	Normalized Pct Red Cells
0.1 uM	98.69	0.87	72.20	89.76	0.05	-48.47
1 uM	100.98	0.83	74.65	90.99	0.06	-28.63
10 uM	96.52	0.81	54.03	82.36	0.03	-39.16

Compound | Normalized Values (A and B Sets) | Responses

BIIB021

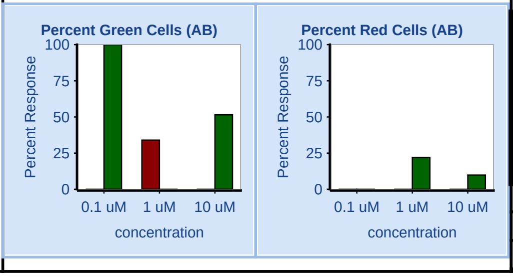

Average Response

Dose	Green Signal	Actual Pct Green Cells	Normalized Pct Green Cells	Red Signal	Actual Pct Red Cells	Normalized Pct Red Cells
0.1 uM	104.44	0.89	53.68	97.72	0.08	-25.37
1 uM	93.63	0.77	0.33	114.67	0.14	6.12
10 uM	87.76	0.63	-52.75	93.25	0.09	-10.32

Compound | Normalized Values (A and B Sets) | Responses

Bilobalide

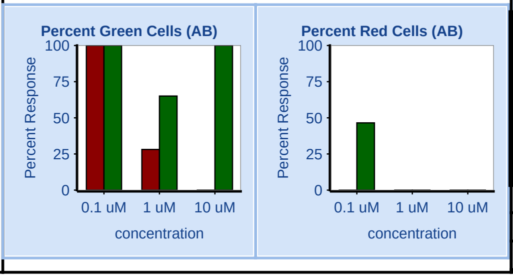

Average Response						
Dose	Green Signal	Actual Pct Green Cells	Normalized Pct Green Cells	Red Signal	Actual Pct Red Cells	Normalized Pct Red Cells
0.1 uM	109.00	0.95	154.58	107.35	0.15	17.36
1 uM	92.70	0.78	46.59	78.29	0.04	-39.81
10 uM	100.88	0.90	15.59	89.67	0.05	-32.37

Compound | Normalized Values (A and B Sets) | Responses

Bimatoprost

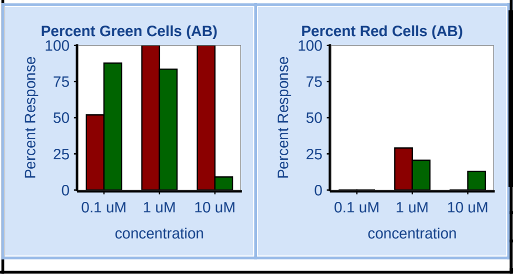

Average Response						
Dose	Green Signal	Actual Pct Green Cells	Normalized Pct Green Cells	Red Signal	Actual Pct Red Cells	Normalized Pct Red Cells
0.1 uM	98.68	0.88	69.93	75.61	0.02	-63.11
1 uM	115.72	0.91	138.83	119.43	0.18	24.87
10 uM	109.40	0.86	77.75	114.07	0.13	2.01

Compound | Normalized Values (A and B Sets) | Responses

Bindarit

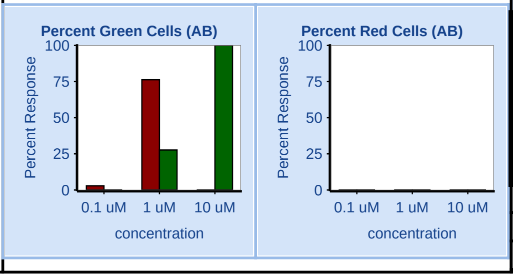

Average Response						
Dose	Green Signal	Actual Pct Green Cells	Normalized Pct Green Cells	Red Signal	Actual Pct Red Cells	Normalized Pct Red Cells
0.1 uM	79.86	0.70	-83.16	73.00	0.03	-50.71
1 uM	96.34	0.79	51.97	78.67	0.04	-47.28
10 uM	97.02	0.79	-39.20	72.38	0.03	-49.73

Compound | Normalized Values (A and B Sets) | Responses

BIO

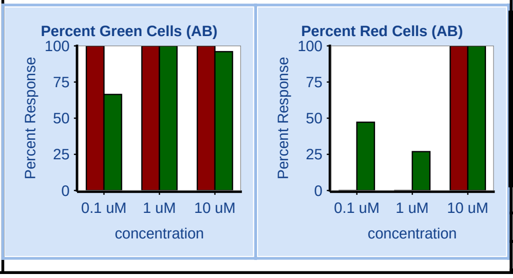

Average Response						
Dose	Green Signal	Actual Pct Green Cells	Normalized Pct Green Cells	Red Signal	Actual Pct Red Cells	Normalized Pct Red Cells
0.1 uM	100.40	0.88	89.10	105.20	0.13	4.07
1 uM	109.77	0.92	186.08	96.58	0.12	13.33
10 uM	130.76	0.94	124.68	391.58	0.92	355.29

Compound | Normalized Values (A and B Sets) | Responses

Biochanin A

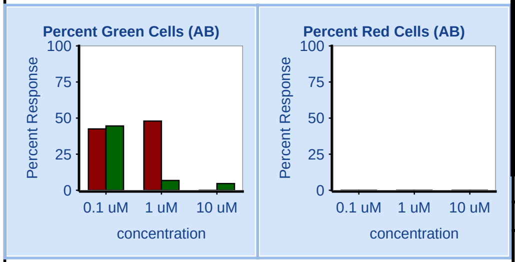

Average Response						
Dose	Green Signal	Actual Pct Green Cells	Normalized Pct Green Cells	Red Signal	Actual Pct Red Cells	Normalized Pct Red Cells
0.1 uM	90.80	0.84	43.53	67.91	0.01	-49.15
1 uM	88.24	0.76	27.37	66.19	0.02	-47.56
10 uM	88.31	0.83	-263.91	65.19	0.01	-51.87

Compound | Normalized Values (A and B Sets) | Responses

Biperiden HCl

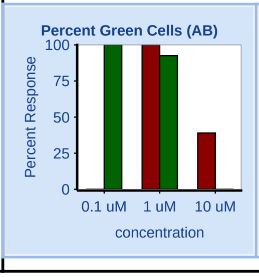

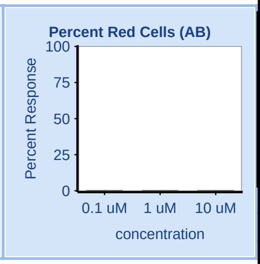

Average Response						
Dose	Green Signal	Actual Pct Green Cells	Normalized Pct Green Cells	Red Signal	Actual Pct Red Cells	Normalized Pct Red Cells
0.1 uM	88.75	0.80	-56.27	80.98	0.03	-58.11
1 uM	107.52	0.90	111.33	72.83	0.02	-50.24
10 uM	84.93	0.75	12.33	103.33	0.06	-30.26

Compound | Normalized Values (A and B Sets) | Responses

BIRB 796 (Doramapimod)

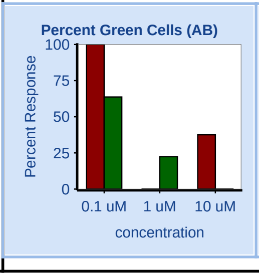

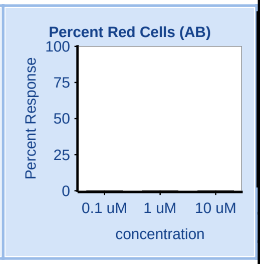

Average Response						
Dose	Green Signal	Actual Pct Green Cells	Normalized Pct Green Cells	Red Signal	Actual Pct Red Cells	Normalized Pct Red Cells
0.1 uM	97.26	0.89	106.66	89.15	0.04	-49.46
1 uM	88.60	0.76	-56.36	97.54	0.07	-25.69
10 uM	82.40	0.73	0.68	86.04	0.04	-38.65

Compound | Normalized Values (A and B Sets) | Responses

Birinapant

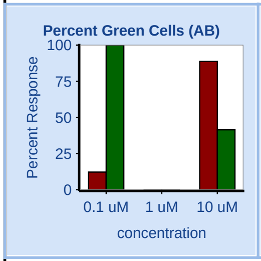

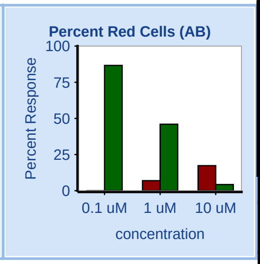

Average Response						
Dose	Green Signal	Actual Pct Green Cells	Normalized Pct Green Cells	Red Signal	Actual Pct Red Cells	Normalized Pct Red Cells
0.1 uM	102.79	0.85	58.20	94.79	0.13	4.25
1 uM	84.02	0.70	-171.60	102.47	0.15	26.47
10 uM	108.23	0.88	65.07	97.62	0.14	10.80

Compound | Normalized Values (A and B Sets) | Responses

BIX 01294

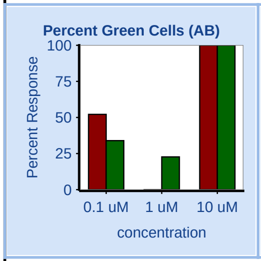

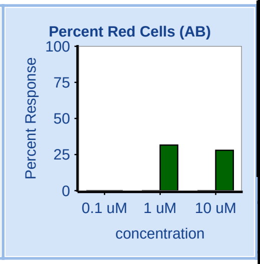

Average Response						
Dose	Green Signal	Actual Pct Green Cells	Normalized Pct Green Cells	Red Signal	Actual Pct Red Cells	Normalized Pct Red Cells
0.1 uM	87.34	0.82	43.05	71.19	0.02	-65.72
1 uM	88.11	0.82	-164.53	86.00	0.08	2.13
10 uM	102.65	0.97	143.80	99.16	0.11	-3.87

Compound | Normalized Values (A and B Sets) | Responses

BIX 02188

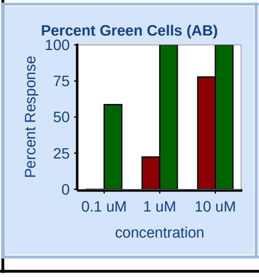

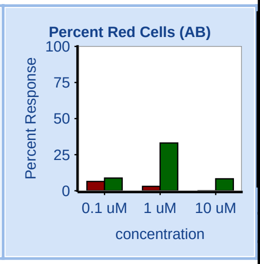

Average Response						
Dose	Green Signal	Actual Pct Green Cells	Normalized Pct Green Cells	Red Signal	Actual Pct Red Cells	Normalized Pct Red Cells
0.1 uM	92.71	0.82	22.78	113.69	0.15	7.63
1 uM	104.69	0.90	75.45	119.71	0.16	18.07
10 uM	105.04	0.87	96.56	114.14	0.12	0.56

Compound	Normalized Values (A and B Sets)	Responses

BIX 02189

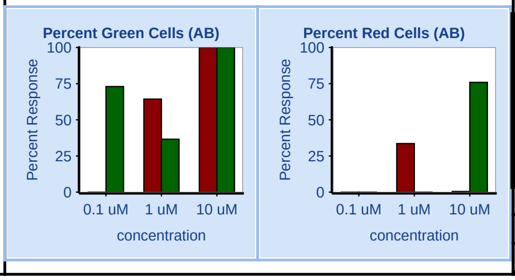

Average Response						
Dose	Green Signal	Actual Pct Green Cells	Normalized Pct Green Cells	Red Signal	Actual Pct Red Cells	Normalized Pct Red Cells
0.1 uM	89.99	0.77	-55.18	90.12	0.08	-31.44
1 uM	97.00	0.84	50.48	108.74	0.12	-2.03
10 uM	112.28	0.95	147.96	141.36	0.20	38.19

Compound	Normalized Values (A and B Sets)	Responses

BKM120 (NVP-BKM120, Buparlisib)

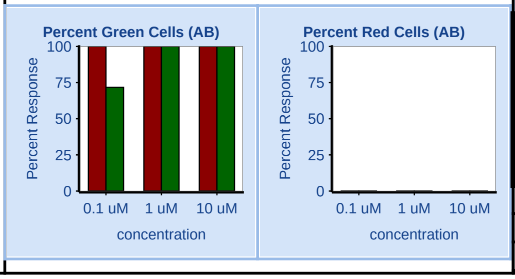

Average Response						
Dose	Green Signal	Actual Pct Green Cells	Normalized Pct Green Cells	Red Signal	Actual Pct Red Cells	Normalized Pct Red Cells
0.1 uM	96.67	0.88	88.54	76.93	0.03	-39.07
1 uM	99.35	0.90	104.31	75.25	0.02	-45.52
10 uM	112.49	0.96	356.44	92.93	0.09	-16.98

Compound	Normalized Values (A and B Sets)	Responses

Bleomycin Sulfate

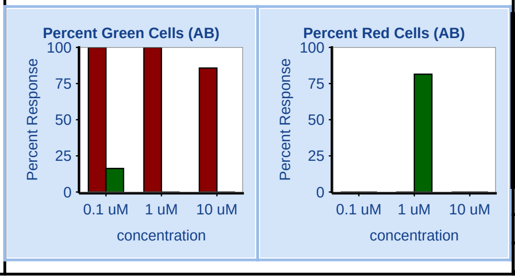

Average Response						
Dose	Green Signal	Actual Pct Green Cells	Normalized Pct Green Cells	Red Signal	Actual Pct Red Cells	Normalized Pct Red Cells
0.1 uM	93.02	0.86	75.54	83.37	0.03	-53.78
1 uM	88.22	0.79	8.65	101.39	0.14	31.25
10 uM	91.95	0.85	35.49	72.87	0.04	-33.32

Compound	Normalized Values (A and B Sets)	Responses

BM- 266

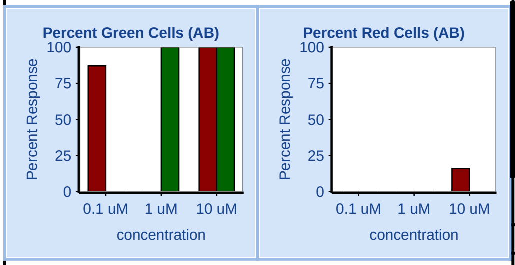

Average Response						
Dose	Green Signal	Actual Pct Green Cells	Normalized Pct Green Cells	Red Signal	Actual Pct Red Cells	Normalized Pct Red Cells
0.1 uM	77.83	0.63	-113.23	77.20	0.01	-66.62
1 uM	93.91	0.90	22.22	63.43	0.01	-44.05
10 uM	100.87	0.97	150.04	91.51	0.08	-13.29

Compound	Normalized Values (A and B Sets)	Responses

BML-190

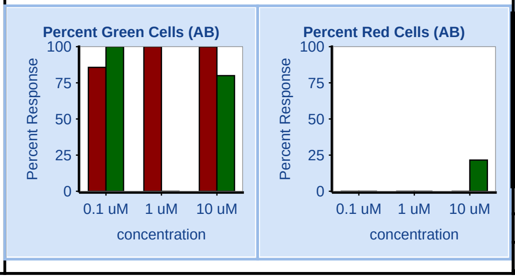

Average Response						
Dose	Green Signal	Actual Pct Green Cells	Normalized Pct Green Cells	Red Signal	Actual Pct Red Cells	Normalized Pct Red Cells
0.1 uM	107.00	0.91	120.62	88.38	0.04	-50.48
1 uM	88.90	0.79	74.40	77.62	0.04	-30.82
10 uM	102.55	0.91	89.95	87.27	0.11	-4.46

Compound | Normalized Values (A and B Sets) | Responses

BML-277

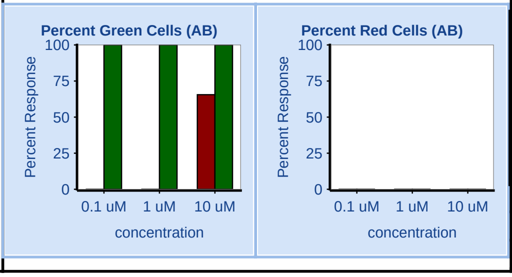

Average Response

Dose	Green Signal	Actual Pct Green Cells	Normalized Pct Green Cells	Red Signal	Actual Pct Red Cells	Normalized Pct Red Cells
0.1 uM	90.21	0.84	50.31	65.87	0.01	-71.08
1 uM	102.21	0.91	-59.31	63.90	0.01	-45.33
10 uM	92.00	0.91	93.00	64.45	0.04	-34.54

Compound | Normalized Values (A and B Sets) | Responses

BMN 673

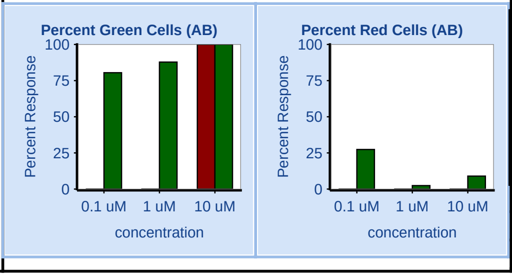

Average Response

Dose	Green Signal	Actual Pct Green Cells	Normalized Pct Green Cells	Red Signal	Actual Pct Red Cells	Normalized Pct Red Cells
0.1 uM	92.54	0.85	-50.77	93.87	0.11	-5.59
1 uM	84.92	0.69	-16.15	85.20	0.08	-27.84
10 uM	117.97	0.95	187.59	93.50	0.09	-14.12

Compound | Normalized Values (A and B Sets) | Responses

BMS-265246

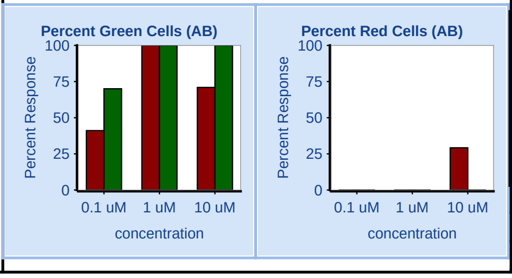

Average Response

Dose	Green Signal	Actual Pct Green Cells	Normalized Pct Green Cells	Red Signal	Actual Pct Red Cells	Normalized Pct Red Cells
0.1 uM	98.76	0.86	55.56	79.68	0.03	-53.25
1 uM	119.56	0.93	150.03	92.19	0.06	-29.47
10 uM	105.57	0.85	85.73	122.68	0.15	4.98

Compound | Normalized Values (A and B Sets) | Responses

BMS-345541

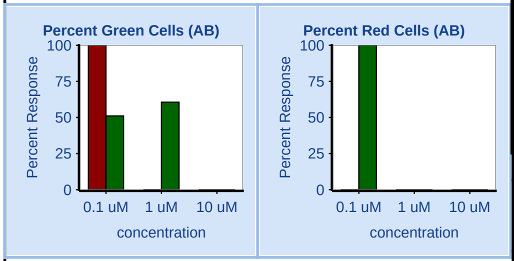

Average Response

Dose	Green Signal	Actual Pct Green Cells	Normalized Pct Green Cells	Red Signal	Actual Pct Red Cells	Normalized Pct Red Cells
0.1 uM	109.64	0.88	96.75	113.27	0.18	35.00
1 uM	81.15	0.66	-1340.69	62.95	0.01	-43.87
10 uM	65.97	0.51	-266.86	72.35	0.06	-24.73

Compound | Normalized Values (A and B Sets) | Responses

BMS-378806

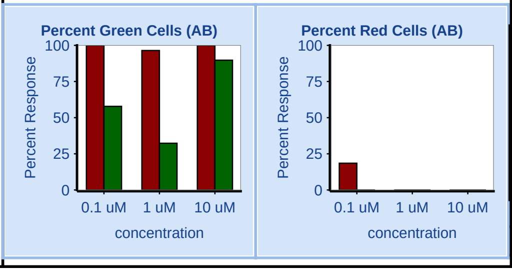

Average Response

Dose	Green Signal	Actual Pct Green Cells	Normalized Pct Green Cells	Red Signal	Actual Pct Red Cells	Normalized Pct Red Cells
0.1 uM	99.55	0.89	91.69	96.06	0.09	-13.39
1 uM	96.96	0.84	64.38	88.85	0.06	-28.18

Compound	Normalized Values (A and B Sets)	Responses

10 uM	101.08	0.90	99.69	86.70	0.07	-27.03

Compound	Normalized Values (A and B Sets)	Responses

BMS-536924

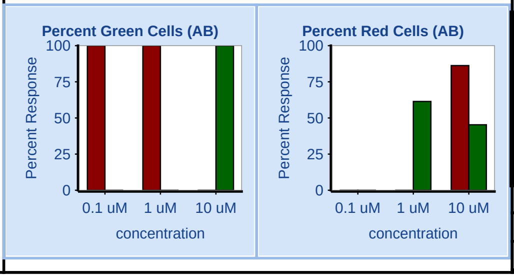

Average Response

Dose	Green Signal	Actual Pct Green Cells	Normalized Pct Green Cells	Red Signal	Actual Pct Red Cells	Normalized Pct Red Cells
0.1 uM	93.84	0.83	50.43	82.08	0.04	-52.89
1 uM	94.31	0.86	186.66	105.03	0.15	30.00
10 uM	99.12	0.87	44.20	122.86	0.26	65.76

Compound	Normalized Values (A and B Sets)	Responses

BMS-707035

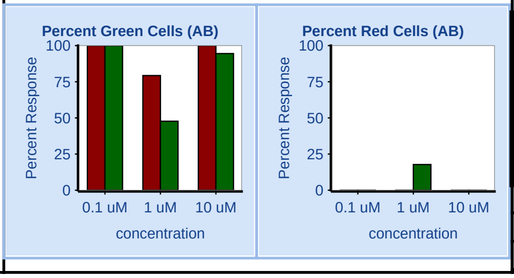

Average Response

Dose	Green Signal	Actual Pct Green Cells	Normalized Pct Green Cells	Red Signal	Actual Pct Red Cells	Normalized Pct Red Cells
0.1 uM	104.62	0.94	127.29	88.82	0.04	-49.33
1 uM	95.79	0.85	63.51	120.79	0.14	8.46
10 uM	105.34	0.89	106.86	96.08	0.05	-35.61

Compound	Normalized Values (A and B Sets)	Responses

BMS-754807

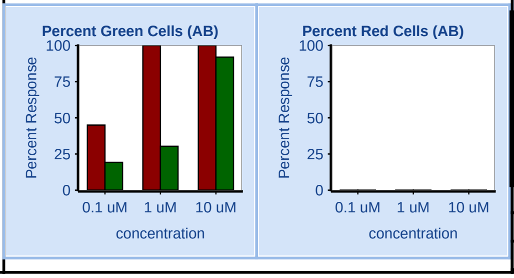

Average Response

Dose	Green Signal	Actual Pct Green Cells	Normalized Pct Green Cells	Red Signal	Actual Pct Red Cells	Normalized Pct Red Cells
0.1 uM	90.33	0.81	32.15	68.71	0.01	-66.74
1 uM	96.63	0.85	85.67	88.66	0.04	-37.99
10 uM	101.45	0.87	97.49	104.87	0.07	-25.05

Compound	Normalized Values (A and B Sets)	Responses

BMS-777607

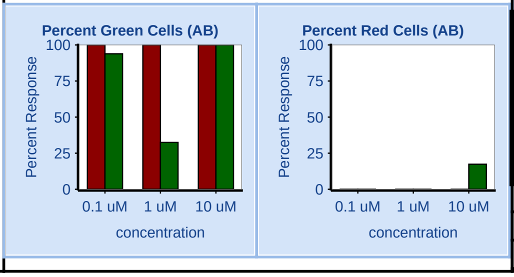

Average Response

Dose	Green Signal	Actual Pct Green Cells	Normalized Pct Green Cells	Red Signal	Actual Pct Red Cells	Normalized Pct Red Cells
0.1 uM	101.32	0.91	104.89	102.63	0.09	-21.23
1 uM	108.22	0.88	124.33	89.52	0.04	-40.63
10 uM	105.39	0.90	115.46	111.33	0.10	-7.89

Compound	Normalized Values (A and B Sets)	Responses

BMS-794833

Average Response

Dose	Green Signal	Actual Pct Green Cells	Normalized Pct Green Cells	Red Signal	Actual Pct Red Cells	Normalized Pct Red Cells

Compound	Normalized Values (A and B Sets)	Responses

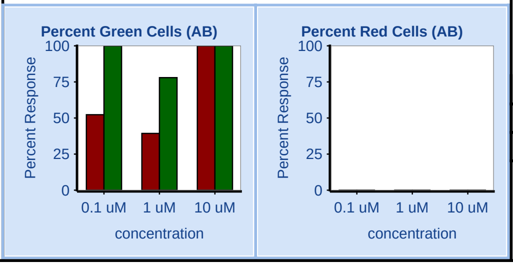

0.1 uM	100.52	0.91	122.59	67.86	0.01	-51.25
1 uM	94.44	0.81	58.65	84.33	0.05	-34.57
10 uM	95.09	0.94	217.12	81.98	0.03	-46.22

Compound	Normalized Values (A and B Sets)	Responses
**BMS-833923**		

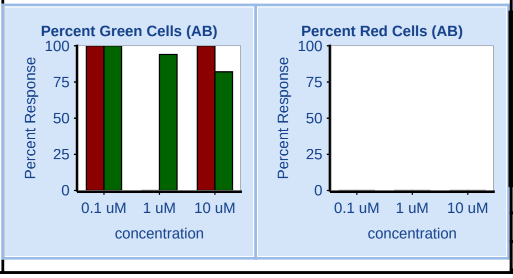

Average Response

Dose	Green Signal	Actual Pct Green Cells	Normalized Pct Green Cells	Red Signal	Actual Pct Red Cells	Normalized Pct Red Cells
0.1 uM	108.80	0.94	236.32	91.49	0.08	-25.28
1 uM	86.64	0.66	-34.44	72.57	0.05	-43.71
10 uM	98.95	0.86	91.43	83.56	0.04	-43.17

Compound	Normalized Values (A and B Sets)	Responses
**BMY 7378**		

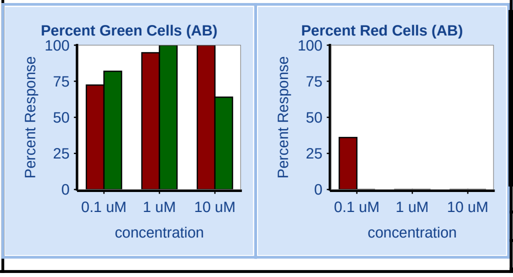

Average Response

Dose	Green Signal	Actual Pct Green Cells	Normalized Pct Green Cells	Red Signal	Actual Pct Red Cells	Normalized Pct Red Cells
0.1 uM	99.17	0.87	77.14	108.20	0.14	9.02
1 uM	104.60	0.89	98.53	74.98	0.02	-46.38
10 uM	109.41	0.93	417.56	87.62	0.05	-35.25

Compound	Normalized Values (A and B Sets)	Responses
**Bosentan Hydrate**		

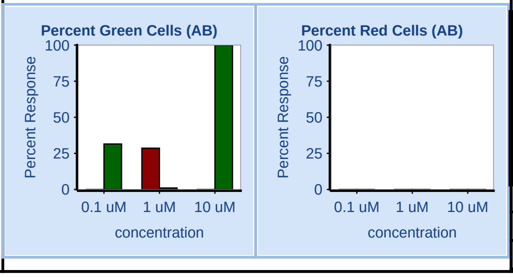

Average Response

Dose	Green Signal	Actual Pct Green Cells	Normalized Pct Green Cells	Red Signal	Actual Pct Red Cells	Normalized Pct Red Cells
0.1 uM	90.82	0.84	5.03	89.28	0.06	-32.02
1 uM	87.69	0.74	14.82	69.41	0.01	-60.79
10 uM	100.29	0.83	6.89	79.09	0.04	-40.22

Compound	Normalized Values (A and B Sets)	Responses
**Brimonidine Tartrate**		

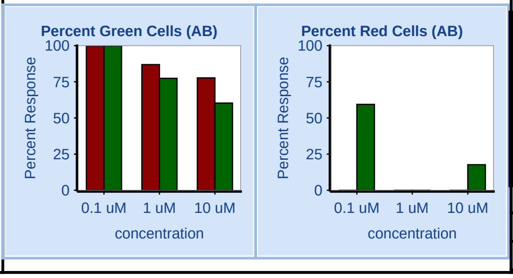

Average Response

Dose	Green Signal	Actual Pct Green Cells	Normalized Pct Green Cells	Red Signal	Actual Pct Red Cells	Normalized Pct Red Cells
0.1 uM	104.62	0.95	199.28	105.84	0.15	15.78
1 uM	100.19	0.83	82.16	94.80	0.09	-20.31
10 uM	100.32	0.83	69.01	102.11	0.12	0.93

Compound	Normalized Values (A and B Sets)	Responses

Brivanib Alaninate (BMS-582664)

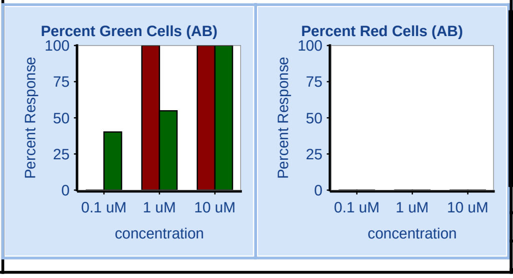

Average Response

Dose	Green Signal	Actual Pct Green Cells	Normalized Pct Green Cells	Red Signal	Actual Pct Red Cells	Normalized Pct Red Cells
0.1 uM	84.07	0.75	-65.63	82.46	0.03	-56.90
1 uM	97.95	0.87	95.95	86.54	0.04	-40.17
10 uM	107.34	0.91	119.35	95.21	0.03	-37.76

Compound	Normalized Values (A and B Sets)	Responses

BRL-15572

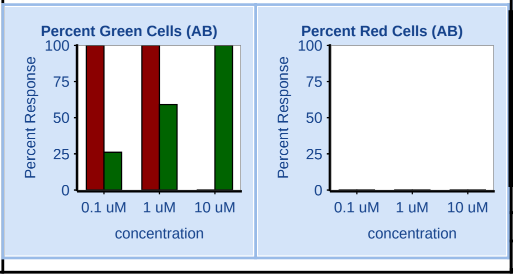

Average Response

Dose	Green Signal	Actual Pct Green Cells	Normalized Pct Green Cells	Red Signal	Actual Pct Red Cells	Normalized Pct Red Cells
0.1 uM	96.26	0.88	82.92	78.72	0.04	-36.28
1 uM	104.34	0.86	79.80	87.87	0.06	-29.18
10 uM	96.87	0.88	-38.58	90.01	0.06	-28.63

Compound	Normalized Values (A and B Sets)	Responses

BRL-54443

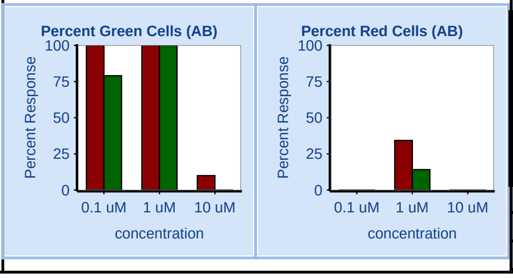

Average Response

Dose	Green Signal	Actual Pct Green Cells	Normalized Pct Green Cells	Red Signal	Actual Pct Red Cells	Normalized Pct Red Cells
0.1 uM	95.11	0.88	89.65	84.74	0.04	-51.75
1 uM	104.00	0.92	126.71	105.41	0.17	24.22
10 uM	91.53	0.81	2.69	87.65	0.09	-13.25

Compound	Normalized Values (A and B Sets)	Responses

Bromfenac Sodium

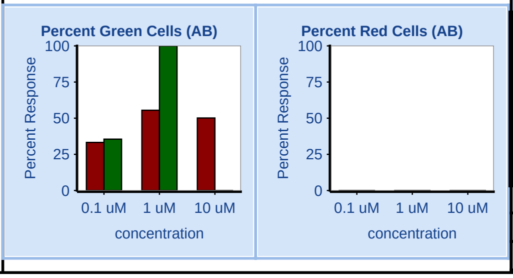

Average Response

Dose	Green Signal	Actual Pct Green Cells	Normalized Pct Green Cells	Red Signal	Actual Pct Red Cells	Normalized Pct Red Cells
0.1 uM	90.05	0.84	34.38	75.90	0.04	-42.80
1 uM	97.94	0.83	81.87	87.90	0.06	-35.14
10 uM	85.24	0.74	19.97	77.50	0.02	-50.67

Compound	Normalized Values (A and B Sets)	Responses

Bromocriptine Mesylate

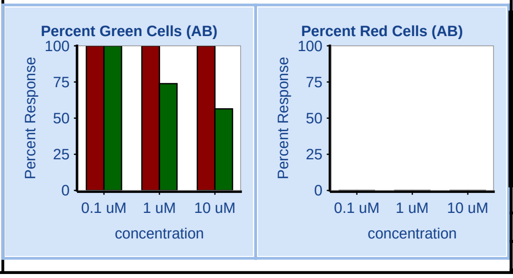

Average Response

Dose	Green Signal	Actual Pct Green Cells	Normalized Pct Green Cells	Red Signal	Actual Pct Red Cells	Normalized Pct Red Cells
0.1 uM	103.99	0.93	156.46	76.14	0.03	-46.80
1 uM	99.55	0.87	107.87	89.28	0.07	-31.98
10 uM	96.68	0.86	117.28	97.00	0.09	-17.49

Compound	Normalized Values (A and B Sets)	Responses

Bromosporine

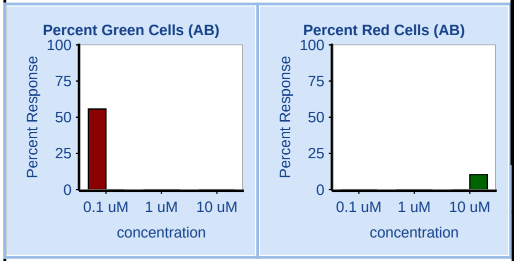

Average Response						
Dose	Green Signal	Actual Pct Green Cells	Normalized Pct Green Cells	Red Signal	Actual Pct Red Cells	Normalized Pct Red Cells
0.1 uM	65.35	0.50	-227.89	87.20	0.05	-39.74
1 uM	83.89	0.76	-266.87	86.24	0.07	-13.91
10 uM	92.88	0.74	-65.56	90.10	0.11	-2.01

Compound	Normalized Values (A and B Sets)	Responses

Brucine

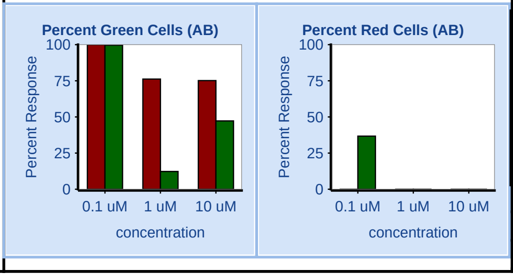

Average Response						
Dose	Green Signal	Actual Pct Green Cells	Normalized Pct Green Cells	Red Signal	Actual Pct Red Cells	Normalized Pct Red Cells
0.1 uM	105.26	0.91	143.51	94.63	0.11	-7.22
1 uM	94.26	0.78	44.24	68.40	0.03	-54.42
10 uM	94.55	0.81	61.20	78.01	0.03	-50.05

Compound	Normalized Values (A and B Sets)	Responses

BS-181 HCl

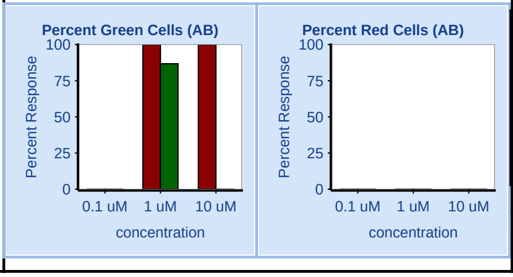

Average Response						
Dose	Green Signal	Actual Pct Green Cells	Normalized Pct Green Cells	Red Signal	Actual Pct Red Cells	Normalized Pct Red Cells
0.1 uM	80.40	0.67	-85.52	93.53	0.08	-28.19
1 uM	110.13	0.90	117.20	88.48	0.06	-31.36
10 uM	93.95	0.77	20.38	98.67	0.08	-19.30

Compound	Normalized Values (A and B Sets)	Responses

BTZ043 Racemate

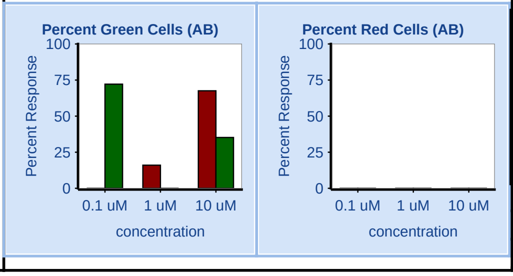

Average Response						
Dose	Green Signal	Actual Pct Green Cells	Normalized Pct Green Cells	Red Signal	Actual Pct Red Cells	Normalized Pct Red Cells
0.1 uM	87.86	0.78	-38.03	79.80	0.03	-56.42
1 uM	87.05	0.73	-30.70	93.01	0.05	-32.93
10 uM	92.44	0.81	51.39	84.15	0.02	-44.95

Compound	Normalized Values (A and B Sets)	Responses

Budesonide

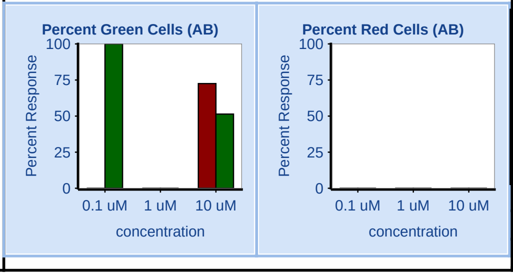

Average Response						
Dose	Green Signal	Actual Pct Green Cells	Normalized Pct Green Cells	Red Signal	Actual Pct Red Cells	Normalized Pct Red Cells
0.1 uM	90.30	0.85	17.43	65.79	0.01	-67.17
1 uM	86.90	0.74	-38.56	83.59	0.03	-42.25
10 uM	92.18	0.82	61.97	80.86	0.04	-37.05

Compound	Normalized Values (A and B Sets)	Responses

Butein

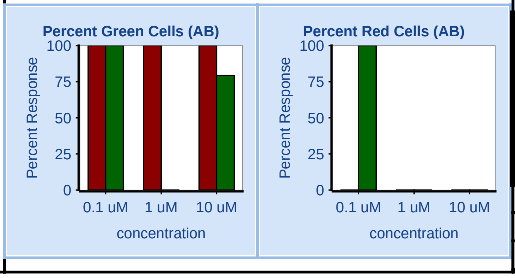

Average Response

Dose	Green Signal	Actual Pct Green Cells	Normalized Pct Green Cells	Red Signal	Actual Pct Red Cells	Normalized Pct Red Cells
0.1 uM	112.35	0.94	149.35	103.46	0.19	37.18
1 uM	96.15	0.84	134.64	71.71	0.03	-32.92
10 uM	102.25	0.91	95.69	85.53	0.07	-20.03

Compound	Normalized Values (A and B Sets)	Responses

BYL719

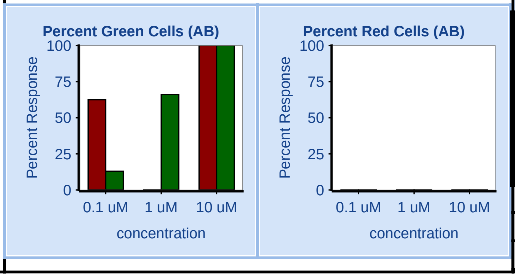

Average Response

Dose	Green Signal	Actual Pct Green Cells	Normalized Pct Green Cells	Red Signal	Actual Pct Red Cells	Normalized Pct Red Cells
0.1 uM	90.06	0.84	37.76	73.08	0.03	-43.20
1 uM	84.02	0.64	-15.90	77.09	0.04	-38.90
10 uM	101.33	0.94	329.19	85.95	0.03	-41.91

Compound	Normalized Values (A and B Sets)	Responses

C 646

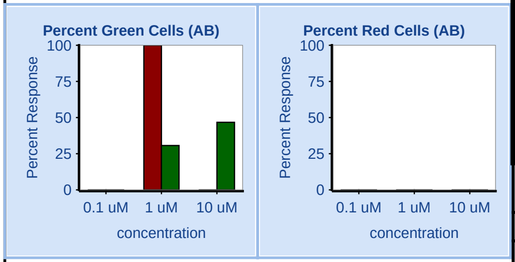

Average Response

Dose	Green Signal	Actual Pct Green Cells	Normalized Pct Green Cells	Red Signal	Actual Pct Red Cells	Normalized Pct Red Cells
0.1 uM	72.11	0.57	-190.00	68.20	0.01	-67.06
1 uM	107.03	0.89	222.25	74.14	0.03	-32.50
10 uM	74.30	0.63	-180.06	61.98	0.01	-47.88

Compound	Normalized Values (A and B Sets)	Responses

C646

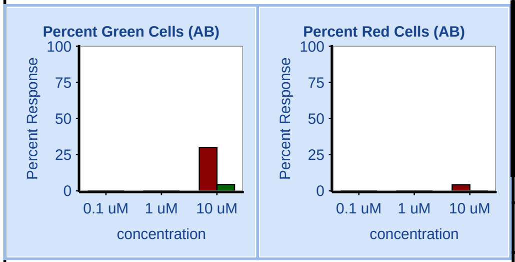

Average Response

Dose	Green Signal	Actual Pct Green Cells	Normalized Pct Green Cells	Red Signal	Actual Pct Red Cells	Normalized Pct Red Cells
0.1 uM	80.51	0.73	-41.82	88.73	0.05	-46.20
1 uM	85.40	0.77	-180.22	73.52	0.04	-31.98
10 uM	88.05	0.83	17.18	94.48	0.10	-5.78

Compound	Normalized Values (A and B Sets)	Responses

Cabozantinib (XL184, BMS-907351)

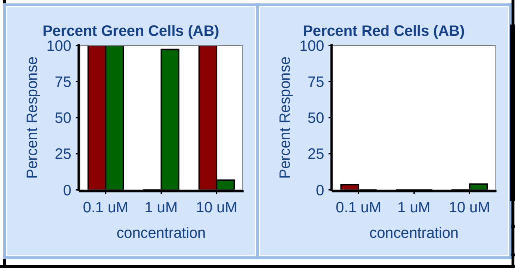

Average Response

Dose	Green Signal	Actual Pct Green Cells	Normalized Pct Green Cells	Red Signal	Actual Pct Red Cells	Normalized Pct Red Cells
0.1 uM	104.24	0.91	110.17	101.30	0.10	-26.27
1 uM	97.30	0.83	-1.26	89.19	0.05	-32.40

Compound	Normalized Values (A and B Sets)	Responses						
		10 uM	89.05	0.82	58.95	122.71	0.13	0.89

Compound	Normalized Values (A and B Sets)	Responses
**Cabozantinib malate (XL184)**	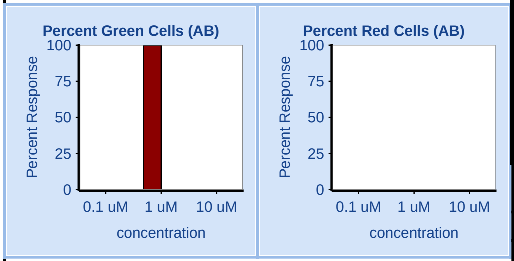	Average Response

Dose	Green Signal	Actual Pct Green Cells	Normalized Pct Green Cells	Red Signal	Actual Pct Red Cells	Normalized Pct Red Cells
0.1 uM	79.90	0.78	-132.14	60.00	0.01	-58.49
1 uM	84.64	0.69	-32.35	62.44	0.01	-62.47
10 uM	62.65	0.34	-572.16	66.25	0.01	-57.43

Compound	Normalized Values (A and B Sets)	Responses
**CAL-101 (Idelalisib, GS-1101)**	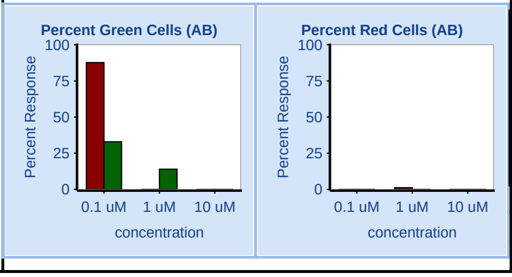	Average Response

Dose	Green Signal	Actual Pct Green Cells	Normalized Pct Green Cells	Red Signal	Actual Pct Red Cells	Normalized Pct Red Cells
0.1 uM	90.59	0.86	60.29	72.34	0.03	-42.81
1 uM	83.05	0.70	3.57	92.95	0.08	-22.49
10 uM	84.90	0.85	-61.50	86.07	0.03	-42.57

Compound	Normalized Values (A and B Sets)	Responses
**Calcifediol**	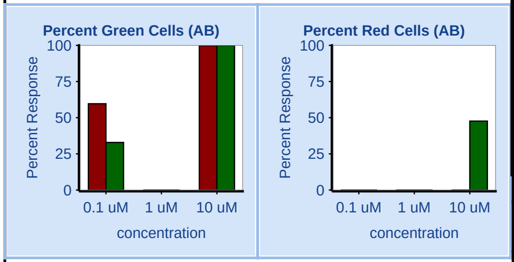	Average Response

Dose	Green Signal	Actual Pct Green Cells	Normalized Pct Green Cells	Red Signal	Actual Pct Red Cells	Normalized Pct Red Cells
0.1 uM	90.66	0.83	46.27	80.17	0.03	-58.66
1 uM	84.02	0.75	-367.81	68.67	0.02	-35.83
10 uM	103.37	0.93	108.62	84.34	0.12	0.70

Compound	Normalized Values (A and B Sets)	Responses
**Calcium Gluceptate**	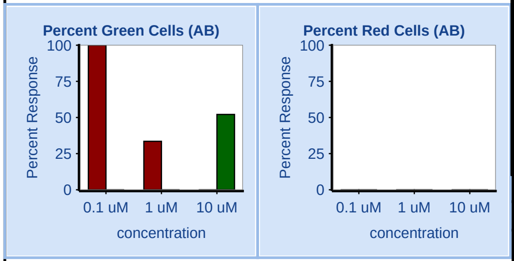	Average Response

Dose	Green Signal	Actual Pct Green Cells	Normalized Pct Green Cells	Red Signal	Actual Pct Red Cells	Normalized Pct Red Cells
0.1 uM	80.48	0.66	-37.76	76.69	0.03	-46.24
1 uM	86.98	0.69	-25.09	70.73	0.04	-49.55
10 uM	82.39	0.66	-180.68	84.03	0.06	-34.47

Compound	Normalized Values (A and B Sets)	Responses
**Camostat Mesilate**		Average Response

Dose				Red Signal		

Compound | Normalized Values (A and B Sets) | Responses

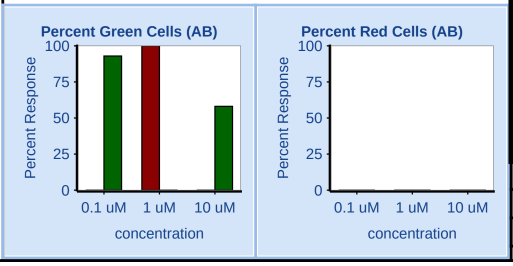

	Green Signal	Actual Pct Green Cells	Normalized Pct Green Cells		Actual Pct Red Cells	Normalized Pct Red Cells
0.1 uM	86.14	0.85	-94.97	73.44	0.01	-57.69
1 uM	89.21	0.80	54.09	66.25	0.01	-62.85
10 uM	87.96	0.77	-14.07	84.33	0.05	-35.43

Compound | Normalized Values (A and B Sets) | Responses

Canagliflozin

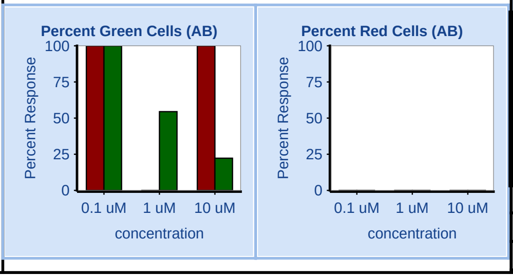

Average Response						
Dose	Green Signal	Actual Pct Green Cells	Normalized Pct Green Cells	Red Signal	Actual Pct Red Cells	Normalized Pct Red Cells
0.1 uM	102.18	0.93	135.87	88.57	0.06	-24.69
1 uM	86.96	0.73	22.83	81.12	0.03	-40.40
10 uM	95.10	0.91	363.42	86.35	0.04	-39.18

Compound | Normalized Values (A and B Sets) | Responses

Candesartan

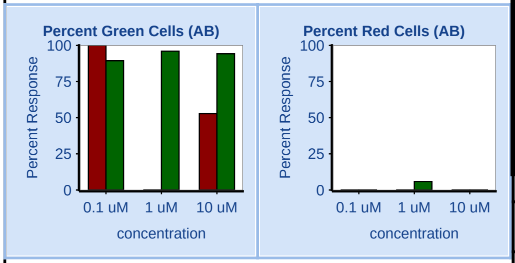

Average Response						
Dose	Green Signal	Actual Pct Green Cells	Normalized Pct Green Cells	Red Signal	Actual Pct Red Cells	Normalized Pct Red Cells
0.1 uM	104.90	0.92	125.17	85.66	0.05	-47.52
1 uM	91.24	0.71	-202.40	107.59	0.11	-6.74
10 uM	99.20	0.83	73.52	111.39	0.09	-12.11

Compound | Normalized Values (A and B Sets) | Responses

Capecitabine

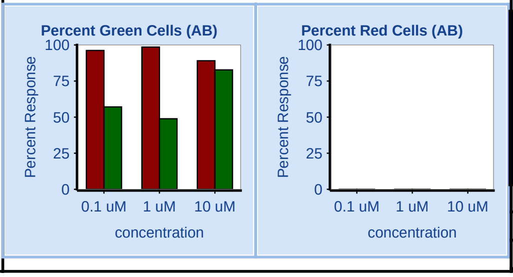

Average Response						
Dose	Green Signal	Actual Pct Green Cells	Normalized Pct Green Cells	Red Signal	Actual Pct Red Cells	Normalized Pct Red Cells
0.1 uM	96.67	0.87	76.63	72.93	0.02	-63.93
1 uM	100.08	0.86	73.71	85.60	0.03	-42.04
10 uM	101.82	0.86	85.89	105.64	0.07	-20.35

Compound | Normalized Values (A and B Sets) | Responses

Carbazochrome sodium sulfonate (AC-17)

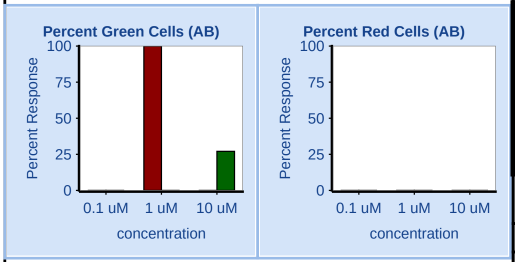

Average Response						
Dose	Green Signal	Actual Pct Green Cells	Normalized Pct Green Cells	Red Signal	Actual Pct Red Cells	Normalized Pct Red Cells
0.1 uM	68.51	0.60	-248.69	79.81	0.04	-40.86
1 uM	90.75	0.81	58.33	74.49	0.03	-50.63
10 uM	83.23	0.74	-31.24	66.24	0.01	-56.21

Compound | Normalized Values (A and B Sets) | Responses

Carbenoxolone Sodium

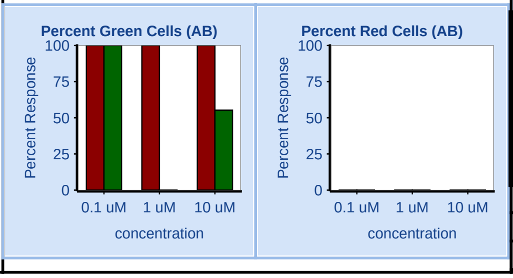

Average Response						
Dose	Green Signal	Actual Pct Green Cells	Normalized Pct Green Cells	Red Signal	Actual Pct Red Cells	Normalized Pct Red Cells
0.1 uM	111.39	0.94	217.05	81.06	0.05	-38.99
1 uM	88.47	0.74	4.76	91.39	0.06	-36.64
10 uM	100.43	0.85	104.94	93.76	0.07	-25.12

Compound | Normalized Values (A and B Sets) | Responses

Carboplatin

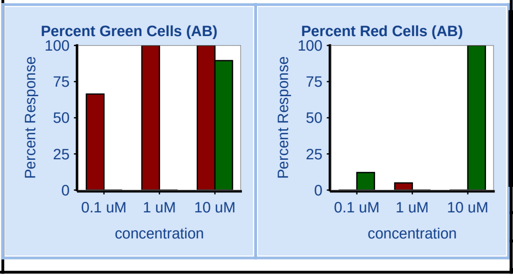

Average Response						
Dose	Green Signal	Actual Pct Green Cells	Normalized Pct Green Cells	Red Signal	Actual Pct Red Cells	Normalized Pct Red Cells
0.1 uM	88.83	0.80	20.46	94.06	0.10	-12.81
1 uM	86.31	0.75	110.59	92.79	0.10	-2.54
10 uM	97.24	0.92	97.59	106.51	0.22	45.21

Compound | Normalized Values (A and B Sets) | Responses

Caspofungin Acetate

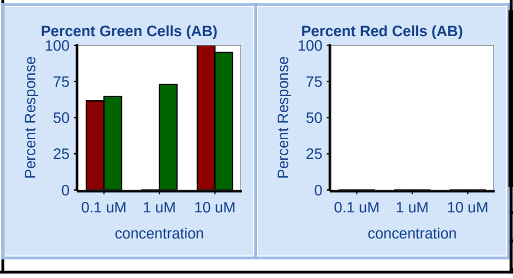

Average Response						
Dose	Green Signal	Actual Pct Green Cells	Normalized Pct Green Cells	Red Signal	Actual Pct Red Cells	Normalized Pct Red Cells
0.1 uM	98.04	0.87	63.21	91.58	0.10	-14.59
1 uM	84.56	0.68	-26.16	89.55	0.07	-29.27
10 uM	103.59	0.89	130.07	74.92	0.03	-50.33

Compound | Normalized Values (A and B Sets) | Responses

Cathepsin Inhibitor 1

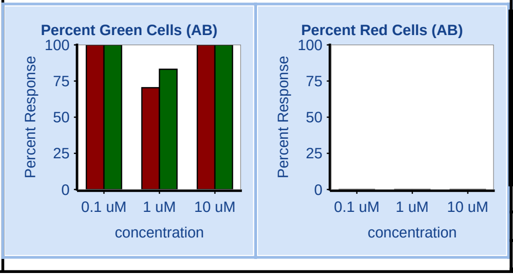

Average Response						
Dose	Green Signal	Actual Pct Green Cells	Normalized Pct Green Cells	Red Signal	Actual Pct Red Cells	Normalized Pct Red Cells
0.1 uM	111.58	0.94	144.45	92.44	0.08	-19.34
1 uM	103.67	0.85	76.77	83.66	0.05	-33.61
10 uM	104.76	0.93	287.09	94.15	0.06	-32.18

Compound | Normalized Values (A and B Sets) | Responses

CCG 50014

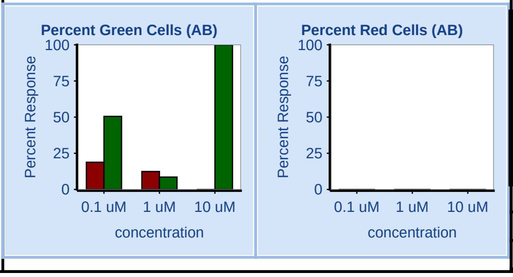

Average Response						
Dose	Green Signal	Actual Pct Green Cells	Normalized Pct Green Cells	Red Signal	Actual Pct Red Cells	Normalized Pct Red Cells
0.1 uM	88.54	0.85	34.67	81.15	0.04	-42.42
1 uM	87.55	0.73	10.48	63.42	0.01	-63.95
10 uM	88.30	0.77	-82.96	61.09	0.01	-59.96

Compound	Normalized Values (A and B Sets)	Responses

CCT 018159

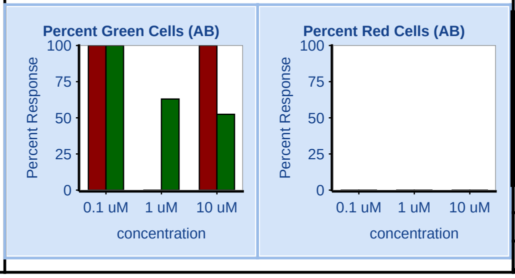

Average Response						
Dose	Green Signal	Actual Pct Green Cells	Normalized Pct Green Cells	Red Signal	Actual Pct Red Cells	Normalized Pct Red Cells
0.1 uM	99.33	0.91	118.60	75.31	0.02	-65.57
1 uM	91.68	0.87	-15.33	66.24	0.02	-40.76
10 uM	97.66	0.93	112.34	80.42	0.05	-29.24

Compound	Normalized Values (A and B Sets)	Responses

CCT128930

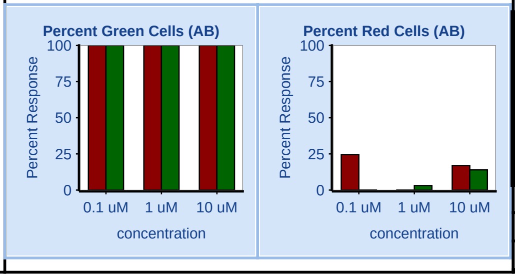

Average Response						
Dose	Green Signal	Actual Pct Green Cells	Normalized Pct Green Cells	Red Signal	Actual Pct Red Cells	Normalized Pct Red Cells
0.1 uM	114.88	0.96	164.43	102.91	0.14	9.68
1 uM	113.37	0.95	129.12	99.24	0.12	-3.50
10 uM	106.68	0.95	412.18	120.57	0.15	15.48

Compound	Normalized Values (A and B Sets)	Responses

CCT129202

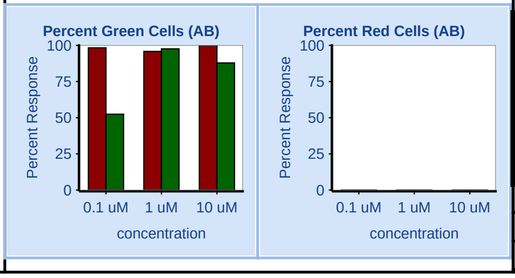

Average Response						
Dose	Green Signal	Actual Pct Green Cells	Normalized Pct Green Cells	Red Signal	Actual Pct Red Cells	Normalized Pct Red Cells
0.1 uM	101.41	0.86	75.38	86.40	0.06	-39.00
1 uM	117.00	0.89	96.74	76.14	0.04	-39.80
10 uM	108.20	0.87	95.50	104.94	0.09	-16.32

Compound	Normalized Values (A and B Sets)	Responses

CCT137690

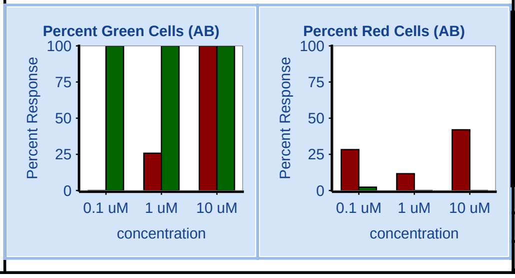

Average Response						
Dose	Green Signal	Actual Pct Green Cells	Normalized Pct Green Cells	Red Signal	Actual Pct Red Cells	Normalized Pct Red Cells
0.1 uM	95.36	0.83	45.02	111.06	0.15	15.28
1 uM	99.23	0.84	79.05	95.37	0.09	-14.78
10 uM	106.41	0.97	497.59	107.43	0.13	2.62

Compound	Normalized Values (A and B Sets)	Responses

CD 437

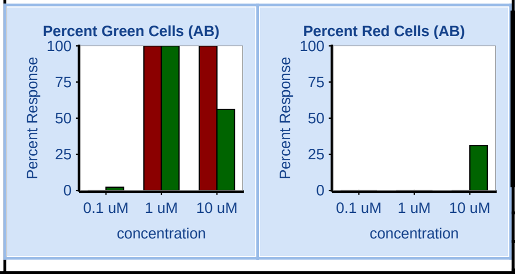

Average Response						
Dose	Green Signal	Actual Pct Green Cells	Normalized Pct Green Cells	Red Signal	Actual Pct Red Cells	Normalized Pct Red Cells
0.1 uM	81.73	0.73	-47.38	77.71	0.03	-53.52
1 uM	105.79	0.95	263.64	52.86	0.02	-38.71
10 uM	112.77	0.91	93.72	93.81	0.11	-1.46

Compound | Normalized Values (A and B Sets) | Responses

CDIBA

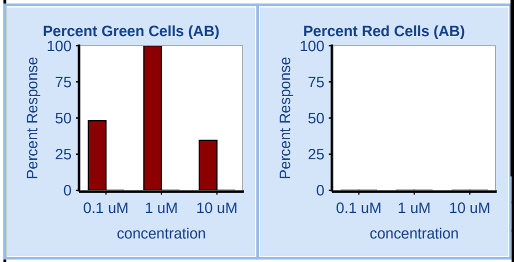

Average Response

Dose	Green Signal	Actual Pct Green Cells	Normalized Pct Green Cells	Red Signal	Actual Pct Red Cells	Normalized Pct Red Cells
0.1 uM	85.50	0.78	8.43	83.10	0.03	-57.77
1 uM	94.56	0.84	56.44	85.09	0.05	-19.21
10 uM	71.38	0.65	-146.15	65.64	0.01	-47.12

Compound | Normalized Values (A and B Sets) | Responses

Cefditoren Pivoxil

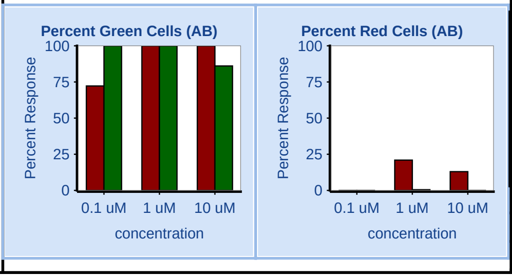

Average Response

Dose	Green Signal	Actual Pct Green Cells	Normalized Pct Green Cells	Red Signal	Actual Pct Red Cells	Normalized Pct Red Cells
0.1 uM	100.39	0.91	93.51	88.46	0.05	-46.85
1 uM	113.95	0.93	142.01	121.73	0.15	10.72
10 uM	110.43	0.90	109.89	114.35	0.12	-6.42

Compound | Normalized Values (A and B Sets) | Responses

Cefoperazone

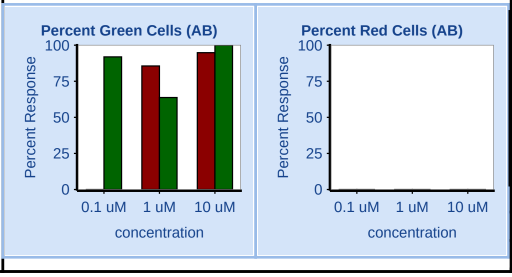

Average Response

Dose	Green Signal	Actual Pct Green Cells	Normalized Pct Green Cells	Red Signal	Actual Pct Red Cells	Normalized Pct Red Cells
0.1 uM	96.52	0.85	39.08	86.81	0.04	-49.46
1 uM	102.73	0.86	74.67	94.99	0.06	-30.22
10 uM	108.51	0.89	110.51	110.42	0.09	-14.94

Compound | Normalized Values (A and B Sets) | Responses

Cefoselis Sulfate

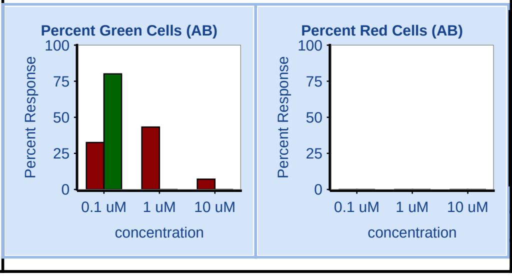

Average Response

Dose	Green Signal	Actual Pct Green Cells	Normalized Pct Green Cells	Red Signal	Actual Pct Red Cells	Normalized Pct Red Cells
0.1 uM	99.08	0.86	56.32	74.78	0.02	-63.90
1 uM	85.75	0.75	-9.92	90.34	0.05	-32.73
10 uM	85.39	0.73	-0.72	83.13	0.03	-39.68

Compound | Normalized Values (A and B Sets) | Responses

Ceftazidime Pentahydrate

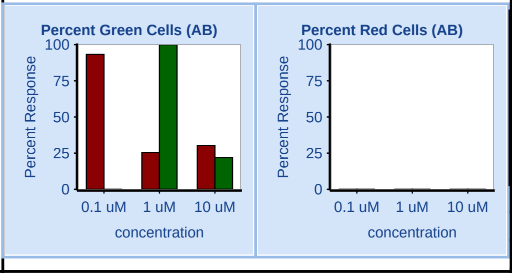

Average Response

Dose	Green Signal	Actual Pct Green Cells	Normalized Pct Green Cells	Red Signal	Actual Pct Red Cells	Normalized Pct Red Cells
0.1 uM	84.03	0.74	-14.02	79.15	0.05	-39.64
1 uM	100.31	0.83	83.52	83.61	0.05	-44.07
10 uM	88.14	0.77	26.04	78.73	0.03	-47.08

Compound	Normalized Values (A and B Sets)	Responses

Ceftiofur HCl

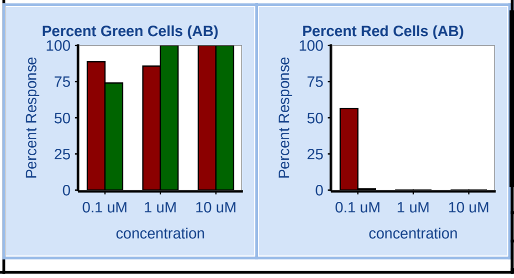

Average Response

Dose	Green Signal	Actual Pct Green Cells	Normalized Pct Green Cells	Red Signal	Actual Pct Red Cells	Normalized Pct Red Cells
0.1 uM	95.61	0.88	81.45	114.06	0.18	28.58
1 uM	105.11	0.89	98.13	83.63	0.05	-33.57
10 uM	111.94	0.95	368.52	82.71	0.04	-39.28

Compound	Normalized Values (A and B Sets)	Responses

CEP-18770 (Delanzomib)

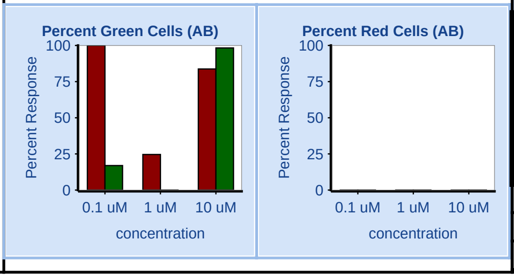

Average Response

Dose	Green Signal	Actual Pct Green Cells	Normalized Pct Green Cells	Red Signal	Actual Pct Red Cells	Normalized Pct Red Cells
0.1 uM	103.27	0.86	94.50	104.04	0.11	-12.41
1 uM	87.46	0.68	-58.02	98.00	0.08	-22.24
10 uM	97.30	0.86	91.03	98.48	0.06	-28.97

Compound	Normalized Values (A and B Sets)	Responses

CEP-32496

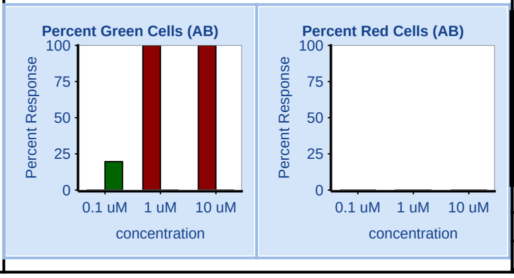

Average Response

Dose	Green Signal	Actual Pct Green Cells	Normalized Pct Green Cells	Red Signal	Actual Pct Red Cells	Normalized Pct Red Cells
0.1 uM	77.88	0.71	-67.67	84.92	0.04	-51.14
1 uM	85.04	0.75	46.13	74.88	0.04	-26.38
10 uM	90.89	0.79	-12.75	83.07	0.08	-15.65

Compound	Normalized Values (A and B Sets)	Responses

CEP-33779

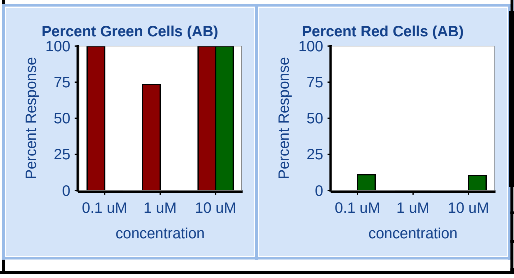

Average Response

Dose	Green Signal	Actual Pct Green Cells	Normalized Pct Green Cells	Red Signal	Actual Pct Red Cells	Normalized Pct Red Cells
0.1 uM	91.88	0.83	30.58	81.79	0.08	-15.87
1 uM	82.71	0.62	-63.13	81.64	0.05	-33.79
10 uM	113.63	0.95	266.57	102.30	0.11	-5.34

Compound	Normalized Values (A and B Sets)	Responses

Cephalomannine

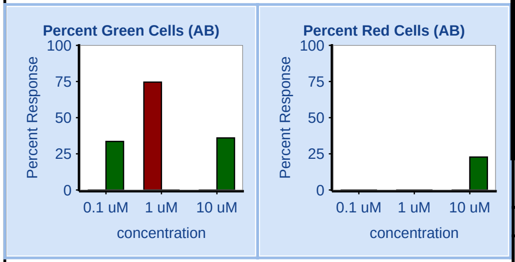

Average Response

Dose	Green Signal	Actual Pct Green Cells	Normalized Pct Green Cells	Red Signal	Actual Pct Red Cells	Normalized Pct Red Cells
0.1 uM	80.81	0.62	-167.93	73.46	0.07	-22.48
1 uM	99.08	0.78	36.18	85.60	0.07	-25.70
10 uM	83.14	0.69		89.92	0.11	-5.88

Compound	Normalized Values (A and B Sets)	Responses						
					-1442.28			

Compound	Normalized Values (A and B Sets)	Responses
**CFTRinh-172**	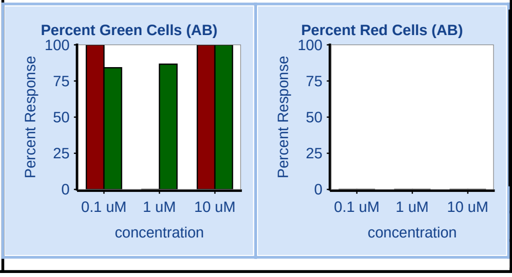	

Average Response

Dose	Green Signal	Actual Pct Green Cells	Normalized Pct Green Cells	Red Signal	Actual Pct Red Cells	Normalized Pct Red Cells
0.1 uM	104.33	0.90	171.26	85.22	0.07	-29.98
1 uM	93.84	0.76	30.06	78.61	0.04	-48.53
10 uM	111.95	0.94	168.00	86.55	0.04	-40.13

Compound	Normalized Values (A and B Sets)	Responses
**CGP 3466B**	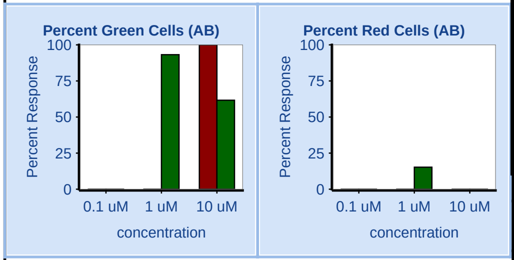	

Average Response

Dose	Green Signal	Actual Pct Green Cells	Normalized Pct Green Cells	Red Signal	Actual Pct Red Cells	Normalized Pct Red Cells
0.1 uM	78.84	0.72	-47.94	82.33	0.04	-52.82
1 uM	92.08	0.80	-537.14	86.12	0.07	-5.21
10 uM	94.62	0.93	117.43	74.02	0.02	-41.34

Compound	Normalized Values (A and B Sets)	Responses
**CGS 9343B**	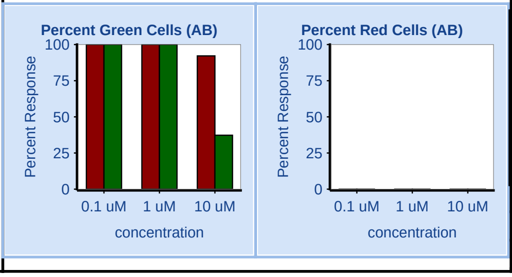	

Average Response

Dose	Green Signal	Actual Pct Green Cells	Normalized Pct Green Cells	Red Signal	Actual Pct Red Cells	Normalized Pct Red Cells
0.1 uM	105.91	0.95	157.65	87.78	0.05	-42.42
1 uM	110.01	0.95	343.76	86.10	0.06	-21.27
10 uM	90.76	0.88	64.73	81.57	0.04	-32.20

Compound	Normalized Values (A and B Sets)	Responses
**CGS 21680 HCl**	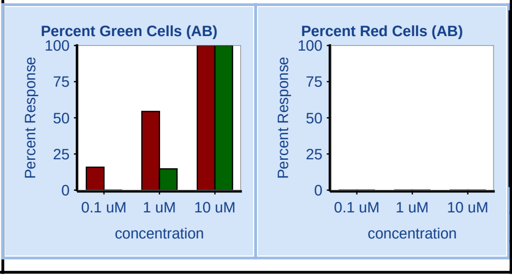	

Average Response

Dose	Green Signal	Actual Pct Green Cells	Normalized Pct Green Cells	Red Signal	Actual Pct Red Cells	Normalized Pct Red Cells
0.1 uM	87.06	0.80	0.87	72.79	0.02	-44.82
1 uM	92.36	0.77	34.54	68.90	0.03	-44.15
10 uM	95.13	0.93	303.09	85.37	0.05	-35.28

Compound	Normalized Values (A and B Sets)	Responses
**CH5132799**		

Average Response

Dose	Green Signal			Red Signal	Actual Pct Red Cells	

Compound	Normalized Values (A and B Sets)	Responses

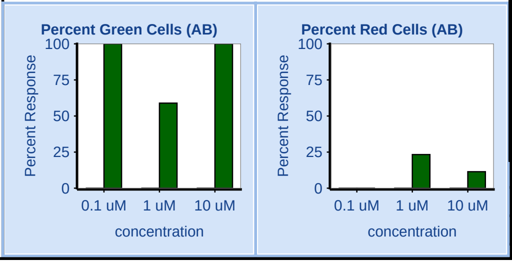

		Actual Pct Green Cells	Normalized Pct Green Cells			Normalized Pct Red Cells
0.1 uM	92.40	0.81	26.26	90.36	0.06	-26.61
1 uM	75.35	0.56	-53.39	103.17	0.13	2.99
10 uM	106.73	0.93	91.28	93.86	0.08	-20.24

Compound	Normalized Values (A and B Sets)	Responses

CHIR-124

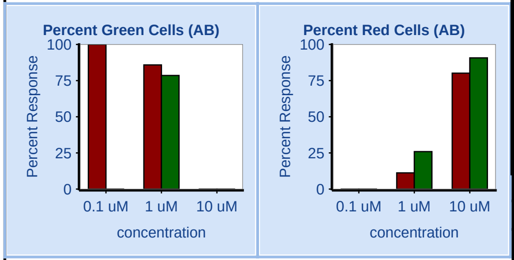

Average Response

Dose	Green Signal	Actual Pct Green Cells	Normalized Pct Green Cells	Red Signal	Actual Pct Red Cells	Normalized Pct Red Cells
0.1 uM	94.13	0.78	-34.87	95.46	0.08	-16.54
1 uM	116.57	0.86	82.21	113.26	0.17	18.56
10 uM	114.54	0.81	-163.36	133.06	0.30	85.46

Compound	Normalized Values (A and B Sets)	Responses

CHIR-99021 (CT99021) HCl

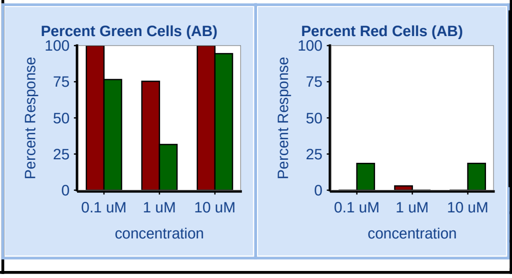

Average Response

Dose	Green Signal	Actual Pct Green Cells	Normalized Pct Green Cells	Red Signal	Actual Pct Red Cells	Normalized Pct Red Cells
0.1 uM	96.18	0.91	212.14	102.45	0.13	4.83
1 uM	95.64	0.80	53.47	97.63	0.10	-16.57
10 uM	121.81	0.90	143.01	88.62	0.09	-16.21

Compound	Normalized Values (A and B Sets)	Responses

Chlorprothixene

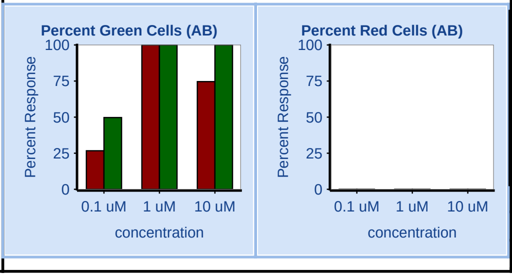

Average Response

Dose	Green Signal	Actual Pct Green Cells	Normalized Pct Green Cells	Red Signal	Actual Pct Red Cells	Normalized Pct Red Cells
0.1 uM	95.12	0.83	38.20	83.69	0.04	-54.59
1 uM	114.34	0.93	144.47	91.45	0.05	-35.74
10 uM	102.72	0.86	91.06	99.87	0.05	-32.67

Compound	Normalized Values (A and B Sets)	Responses

CI994 (Tacedinaline)

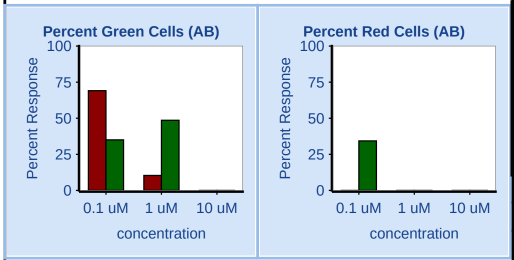

Average Response

Dose	Green Signal	Actual Pct Green Cells	Normalized Pct Green Cells	Red Signal	Actual Pct Red Cells	Normalized Pct Red Cells
0.1 uM	93.97	0.85	52.07	104.06	0.14	13.76
1 uM	87.15	0.75	29.49	74.69	0.03	-44.34
10 uM	82.97	0.68	-348.58	100.15	0.08	-19.04

Compound	Normalized Values (A and B Sets)	Responses

Ciclopirox

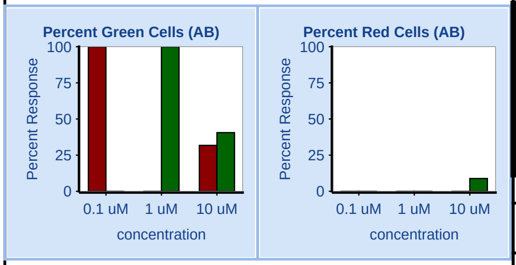

Average Response						
Dose	Green Signal	Actual Pct Green Cells	Normalized Pct Green Cells	Red Signal	Actual Pct Red Cells	Normalized Pct Red Cells
0.1 uM	91.16	0.80	29.03	75.19	0.03	-58.77
1 uM	90.70	0.84	-285.91	79.49	0.06	-18.93
10 uM	98.93	0.85	36.18	86.68	0.11	-4.94

Compound	Normalized Values (A and B Sets)	Responses

Cilazapril Monohydrate

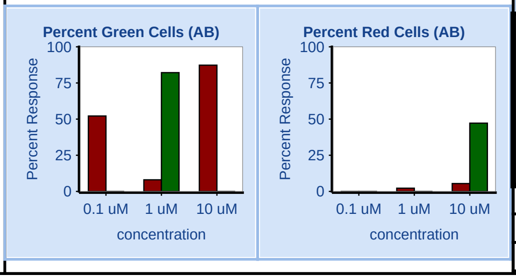

Average Response						
Dose	Green Signal	Actual Pct Green Cells	Normalized Pct Green Cells	Red Signal	Actual Pct Red Cells	Normalized Pct Red Cells
0.1 uM	93.05	0.79	22.16	100.51	0.10	-14.94
1 uM	99.04	0.85	45.08	99.03	0.09	-17.36
10 uM	90.35	0.76	17.54	128.63	0.18	26.32

Compound	Normalized Values (A and B Sets)	Responses

Cilengitide

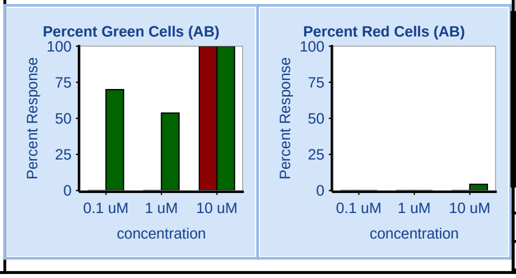

Average Response						
Dose	Green Signal	Actual Pct Green Cells	Normalized Pct Green Cells	Red Signal	Actual Pct Red Cells	Normalized Pct Red Cells
0.1 uM	96.76	0.85	-23.45	78.02	0.05	-39.61
1 uM	85.10	0.64	-54.47	88.04	0.06	-39.61
10 uM	112.52	0.90	137.63	97.08	0.10	-10.65

Compound	Normalized Values (A and B Sets)	Responses

Cilomilast

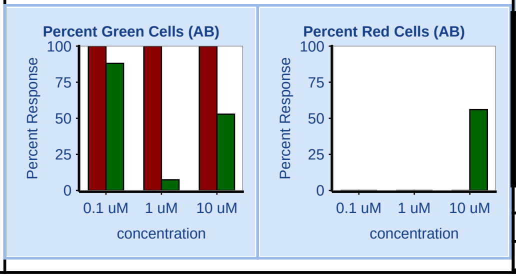

Average Response						
Dose	Green Signal	Actual Pct Green Cells	Normalized Pct Green Cells	Red Signal	Actual Pct Red Cells	Normalized Pct Red Cells
0.1 uM	109.12	0.92	122.62	96.43	0.06	-39.90
1 uM	102.60	0.83	61.80	95.37	0.08	-22.54
10 uM	96.67	0.85	79.38	124.08	0.14	13.22

Compound	Normalized Values (A and B Sets)	Responses

Cinacalcet HCl

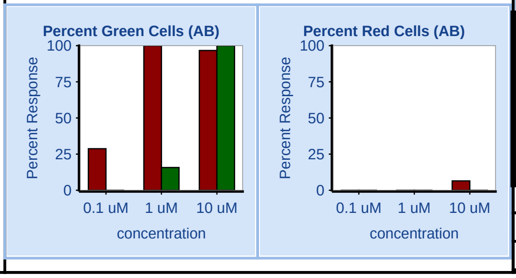

Average Response						
Dose	Green Signal	Actual Pct Green Cells	Normalized Pct Green Cells	Red Signal	Actual Pct Red Cells	Normalized Pct Red Cells
0.1 uM	81.29	0.71	-28.69	92.27	0.05	-49.38
1 uM	95.43	0.83	63.94	75.31	0.01	-51.19
10 uM	119.08	0.91	121.55	121.71	0.12	-2.80

Compound | Normalized Values (A and B Sets) | Responses

Cinchonidine

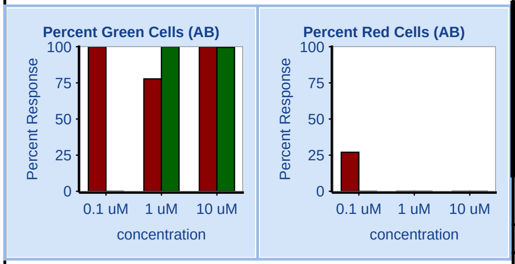

Average Response						
Dose	Green Signal	Actual Pct Green Cells	Normalized Pct Green Cells	Red Signal	Actual Pct Red Cells	Normalized Pct Red Cells
0.1 uM	82.02	0.69	-123.49	93.36	0.10	-9.10
1 uM	101.53	0.87	91.75	82.52	0.03	-40.46
10 uM	107.33	0.94	417.35	76.67	0.02	-48.05

Compound | Normalized Values (A and B Sets) | Responses

Cinchophen

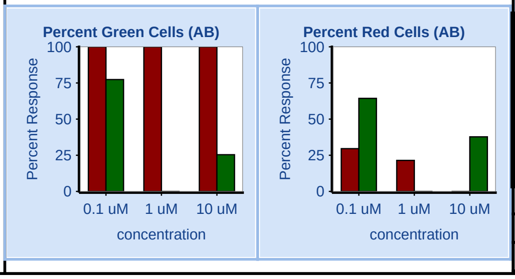

Average Response						
Dose	Green Signal	Actual Pct Green Cells	Normalized Pct Green Cells	Red Signal	Actual Pct Red Cells	Normalized Pct Red Cells
0.1 uM	100.65	0.90	109.13	117.43	0.19	46.96
1 uM	80.99	0.65	-23.98	84.96	0.09	-13.88
10 uM	94.45	0.88	64.15	92.56	0.13	3.61

Compound | Normalized Values (A and B Sets) | Responses

Cinepazide maleate

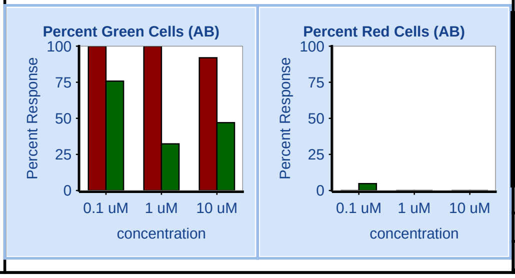

Average Response						
Dose	Green Signal	Actual Pct Green Cells	Normalized Pct Green Cells	Red Signal	Actual Pct Red Cells	Normalized Pct Red Cells
0.1 uM	105.52	0.91	201.00	82.21	0.08	-24.36
1 uM	99.17	0.83	80.69	75.13	0.03	-51.51
10 uM	96.49	0.82	69.50	73.97	0.02	-51.15

Compound | Normalized Values (A and B Sets) | Responses

Ciproxifan

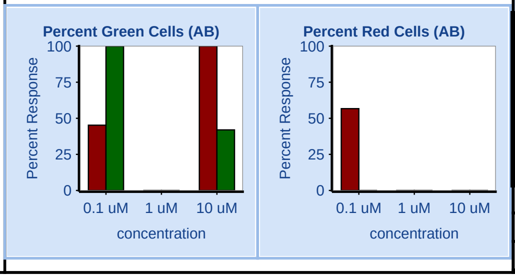

Average Response						
Dose	Green Signal	Actual Pct Green Cells	Normalized Pct Green Cells	Red Signal	Actual Pct Red Cells	Normalized Pct Red Cells
0.1 uM	103.96	0.88	87.89	99.02	0.14	8.67
1 uM	75.45	0.58	-71.28	83.67	0.05	-34.30
10 uM	94.23	0.89	163.72	100.89	0.07	-23.25

Compound | Normalized Values (A and B Sets) | Responses

Cisatracurium Besylate

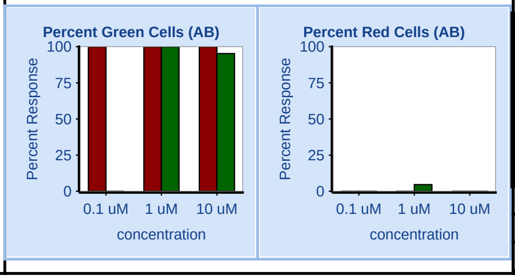

Average Response						
Dose	Green Signal	Actual Pct Green Cells	Normalized Pct Green Cells	Red Signal	Actual Pct Red Cells	Normalized Pct Red Cells
0.1 uM	95.41	0.85	67.92	60.56	0.00	-72.73
1 uM	105.95	0.94	196.32	95.64	0.09	-2.58
10 uM	107.85	0.94	119.77	87.28	0.08	-15.43

Compound | Normalized Values (A and B Sets) | Responses

Cisplatin

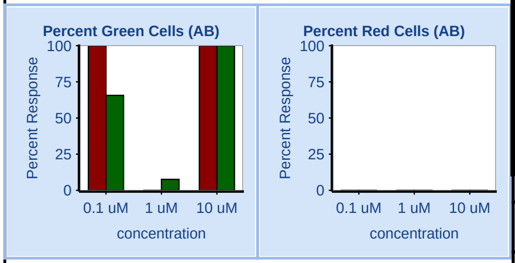

Average Response						
Dose	Green Signal	Actual Pct Green Cells	Normalized Pct Green Cells	Red Signal	Actual Pct Red Cells	Normalized Pct Red Cells
0.1 uM	102.65	0.88	92.64	75.19	0.02	-62.78
1 uM	82.84	0.69	-157.87	67.10	0.02	-48.58
10 uM	107.13	0.90	115.74	104.23	0.08	-19.45

Compound | Normalized Values (A and B Sets) | Responses

Clevidipine Butyrate

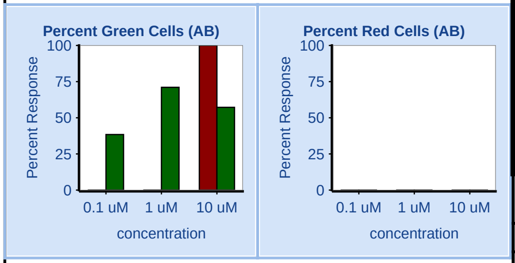

Average Response						
Dose	Green Signal	Actual Pct Green Cells	Normalized Pct Green Cells	Red Signal	Actual Pct Red Cells	Normalized Pct Red Cells
0.1 uM	74.85	0.59	-262.24	96.31	0.09	-26.18
1 uM	97.18	0.84	28.31	94.76	0.07	-25.92
10 uM	109.89	0.88	97.22	83.79	0.04	-35.50

Compound | Normalized Values (A and B Sets) | Responses

Clevudine

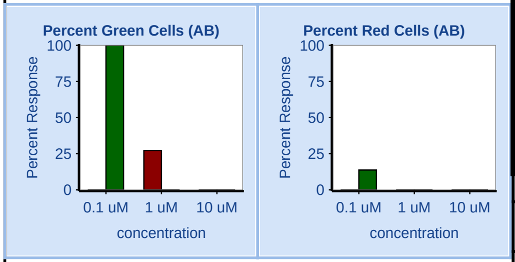

Average Response						
Dose	Green Signal	Actual Pct Green Cells	Normalized Pct Green Cells	Red Signal	Actual Pct Red Cells	Normalized Pct Red Cells
0.1 uM	92.32	0.88	-19.57	87.15	0.08	-22.41
1 uM	71.34	0.52	-156.69	77.21	0.04	-48.39
10 uM	74.79	0.58	-75.45	67.04	0.05	-39.42

Compound | Normalized Values (A and B Sets) | Responses

Climbazole

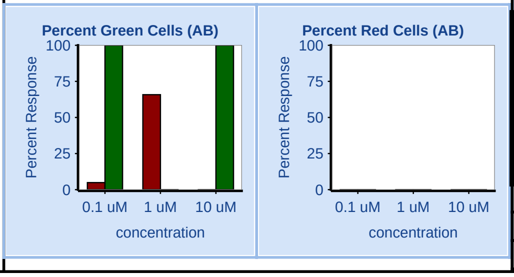

Average Response						
Dose	Green Signal	Actual Pct Green Cells	Normalized Pct Green Cells	Red Signal	Actual Pct Red Cells	Normalized Pct Red Cells
0.1 uM	94.26	0.85	61.66	76.25	0.02	-61.14
1 uM	90.32	0.84	27.06	78.27	0.03	-35.16
10 uM	96.67	0.88	59.41	82.74	0.05	-28.02

Compound | Normalized Values (A and B Sets) | Responses

Clinafoxacin HCl

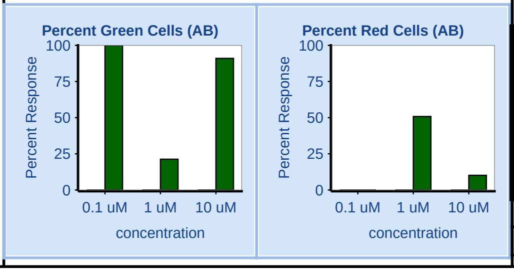

Average Response						
Dose	Green Signal	Actual Pct Green Cells	Normalized Pct Green Cells	Red Signal	Actual Pct Red Cells	Normalized Pct Red Cells
0.1 uM	101.70	0.91	36.97	93.56	0.10	-12.59
1 uM	88.90	0.73	6.64	112.39	0.17	19.86

Compound | Normalized Values (A and B Sets) | Responses

Dose	Green Signal	Actual Pct Green Cells	Normalized Pct Green Cells	Red Signal	Actual Pct Red Cells	Normalized Pct Red Cells
10 uM	92.61	0.83	41.91	104.49	0.12	0.61

Compound | Normalized Values (A and B Sets) | Responses

Clindamycin

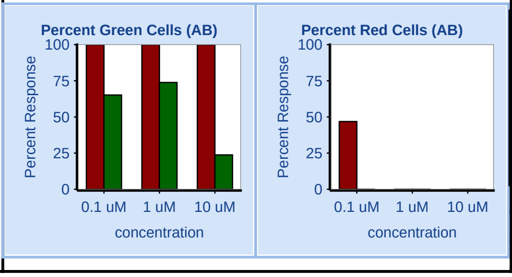

Average Response

Dose	Green Signal	Actual Pct Green Cells	Normalized Pct Green Cells	Red Signal	Actual Pct Red Cells	Normalized Pct Red Cells
0.1 uM	107.97	0.91	110.59	98.36	0.13	1.46
1 uM	104.10	0.90	97.15	76.83	0.02	-45.25
10 uM	98.22	0.89	171.06	85.68	0.04	-38.07

Compound | Normalized Values (A and B Sets) | Responses

Clindamycin HCl

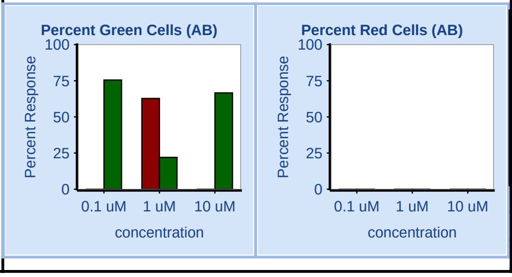

Average Response

Dose	Green Signal	Actual Pct Green Cells	Normalized Pct Green Cells	Red Signal	Actual Pct Red Cells	Normalized Pct Red Cells
0.1 uM	93.03	0.83	35.17	86.51	0.05	-28.29
1 uM	92.97	0.79	42.38	96.79	0.08	-19.76
10 uM	99.24	0.88	15.97	100.40	0.08	-17.83

Compound | Normalized Values (A and B Sets) | Responses

Clindamycin palmitate HCl

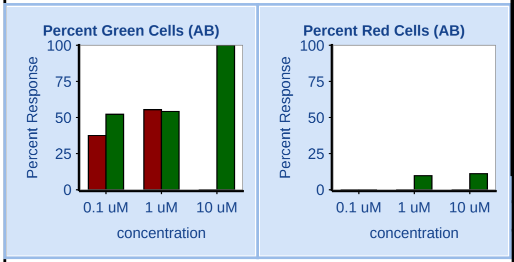

Average Response

Dose	Green Signal	Actual Pct Green Cells	Normalized Pct Green Cells	Red Signal	Actual Pct Red Cells	Normalized Pct Red Cells
0.1 uM	92.67	0.84	44.93	81.75	0.04	-34.50
1 uM	94.55	0.81	54.79	94.52	0.10	-12.63
10 uM	96.61	0.87	-272.53	96.63	0.09	-18.02

Compound | Normalized Values (A and B Sets) | Responses

Clinofibrate

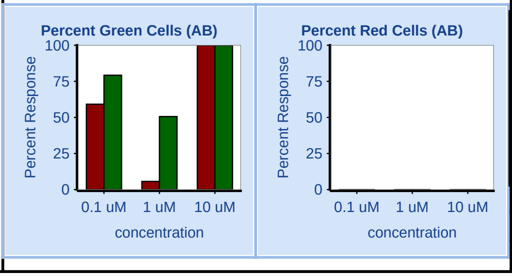

Average Response

Dose	Green Signal	Actual Pct Green Cells	Normalized Pct Green Cells	Red Signal	Actual Pct Red Cells	Normalized Pct Red Cells
0.1 uM	94.83	0.86	69.21	87.95	0.05	-32.04
1 uM	89.55	0.75	28.14	89.64	0.06	-28.30
10 uM	97.02	0.92	265.80	80.18	0.03	-46.16

Compound | Normalized Values (A and B Sets) | Responses

Clofarabine

Average Response

Dose	Green Signal	Actual Pct Green Cells	Normalized Pct Green Cells	Red Signal	Actual Pct Red Cells	Normalized Pct Red Cells

Compound	Normalized Values (A and B Sets)	Responses

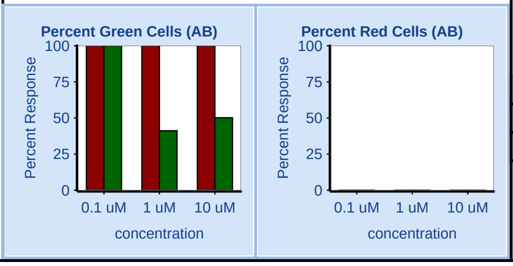

0.1 uM	107.83	0.93	113.87	76.07	0.02	-61.40
1 uM	117.08	0.86	92.62	92.20	0.05	-32.18
10 uM	113.61	0.88	95.61	95.05	0.08	-15.95

Compound	Normalized Values (A and B Sets)	Responses

Clorprenaline HCL

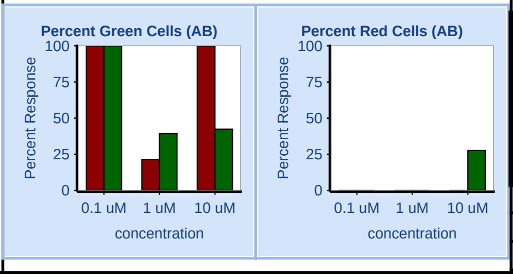

Average Response

Dose	Green Signal	Actual Pct Green Cells	Normalized Pct Green Cells	Red Signal	Actual Pct Red Cells	Normalized Pct Red Cells
0.1 uM	106.93	0.93	179.96	73.41	0.03	-45.60
1 uM	92.68	0.76	30.22	68.02	0.03	-55.07
10 uM	99.07	0.85	124.26	95.44	0.10	-10.89

Compound	Normalized Values (A and B Sets)	Responses

Closantel Sodium

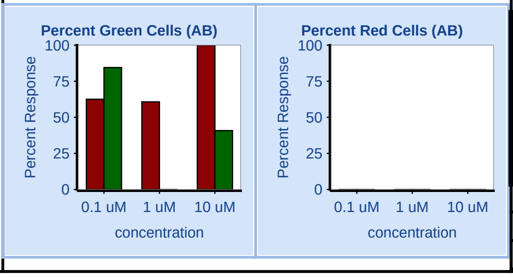

Average Response

Dose	Green Signal	Actual Pct Green Cells	Normalized Pct Green Cells	Red Signal	Actual Pct Red Cells	Normalized Pct Red Cells
0.1 uM	94.67	0.88	73.51	77.53	0.05	-36.62
1 uM	87.11	0.71	-11.99	78.73	0.03	-50.41
10 uM	100.31	0.85	117.31	89.85	0.05	-35.85

Compound	Normalized Values (A and B Sets)	Responses

Cloxacillin Sodium

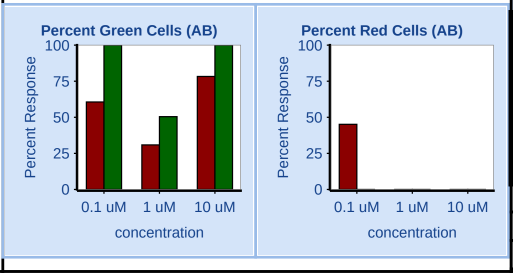

Average Response

Dose	Green Signal	Actual Pct Green Cells	Normalized Pct Green Cells	Red Signal	Actual Pct Red Cells	Normalized Pct Red Cells
0.1 uM	102.18	0.90	108.79	110.81	0.16	18.23
1 uM	91.51	0.78	40.65	82.70	0.05	-34.34
10 uM	107.41	0.93	144.86	84.30	0.04	-40.09

Compound	Normalized Values (A and B Sets)	Responses

CNX-2006

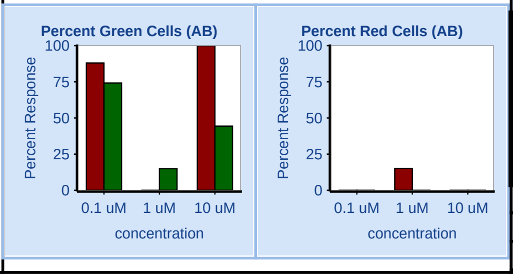

Average Response

Dose	Green Signal	Actual Pct Green Cells	Normalized Pct Green Cells	Red Signal	Actual Pct Red Cells	Normalized Pct Red Cells
0.1 uM	95.48	0.87	81.12	94.97	0.07	-35.45
1 uM	92.70	0.83	-71.66	100.70	0.11	1.66
10 uM	97.05	0.89	78.40	83.98	0.06	-25.29

Compound | Normalized Values (A and B Sets) | Responses

CO-1686 (AVL-301)

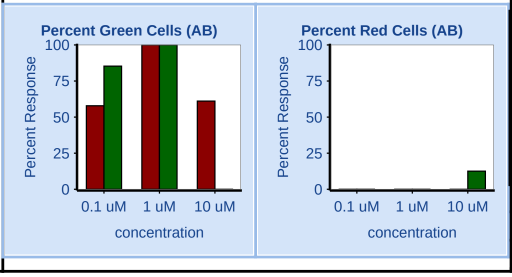

Average Response						
Dose	Green Signal	Actual Pct Green Cells	Normalized Pct Green Cells	Red Signal	Actual Pct Red Cells	Normalized Pct Red Cells
0.1 uM	91.70	0.86	71.59	74.78	0.04	-51.31
1 uM	102.43	0.92	182.42	66.09	0.01	-43.16
10 uM	89.60	0.82	7.38	79.31	0.09	-14.48

Compound | Normalized Values (A and B Sets) | Responses

Cobicistat (GS-9350)

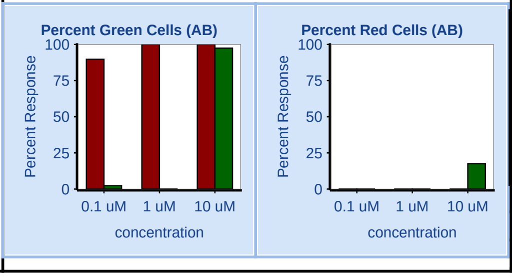

Average Response						
Dose	Green Signal	Actual Pct Green Cells	Normalized Pct Green Cells	Red Signal	Actual Pct Red Cells	Normalized Pct Red Cells
0.1 uM	92.28	0.83	46.08	78.98	0.06	-34.36
1 uM	93.23	0.79	49.53	68.31	0.01	-60.33
10 uM	95.41	0.88	105.49	75.63	0.08	-24.52

Compound | Normalized Values (A and B Sets) | Responses

Colistimethate Sodium

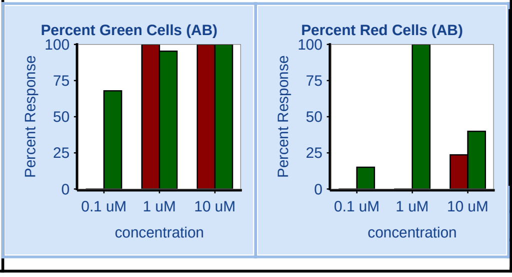

Average Response						
Dose	Green Signal	Actual Pct Green Cells	Normalized Pct Green Cells	Red Signal	Actual Pct Red Cells	Normalized Pct Red Cells
0.1 uM	89.89	0.86	33.72	99.37	0.11	-7.39
1 uM	104.93	0.88	117.78	112.02	0.20	33.47
10 uM	112.74	0.93	134.77	117.68	0.18	31.79

Compound | Normalized Values (A and B Sets) | Responses

Combretastatin A4

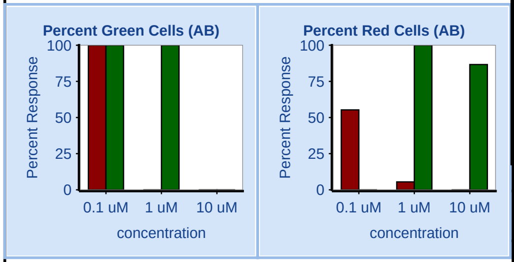

Average Response						
Dose	Green Signal	Actual Pct Green Cells	Normalized Pct Green Cells	Red Signal	Actual Pct Red Cells	Normalized Pct Red Cells
0.1 uM	110.79	0.92	127.77	105.19	0.15	19.90
1 uM	104.06	0.87	-247.98	117.19	0.23	77.55
10 uM	80.39	0.58	-229.20	99.86	0.19	31.58

Compound | Normalized Values (A and B Sets) | Responses

Conivaptan HCl

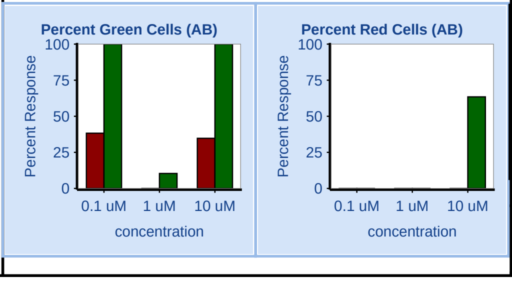

Average Response						
Dose	Green Signal	Actual Pct Green Cells	Normalized Pct Green Cells	Red Signal	Actual Pct Red Cells	Normalized Pct Red Cells
0.1 uM	97.20	0.86	72.39	86.21	0.05	-44.10
1 uM	74.00	0.60		70.89	0.04	-22.73

Compound	Normalized Values (A and B Sets)	Responses

			-1576.75			
10 uM	100.47	0.89	69.99	105.73	0.18	28.26

Compound	Normalized Values (A and B Sets)	Responses

Cortisone acetate

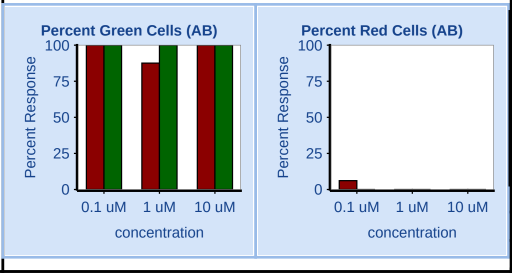

Average Response

Dose	Green Signal	Actual Pct Green Cells	Normalized Pct Green Cells	Red Signal	Actual Pct Red Cells	Normalized Pct Red Cells
0.1 uM	109.41	0.94	142.17	97.40	0.10	-8.20
1 uM	106.43	0.91	113.24	99.41	0.11	-5.98
10 uM	107.73	0.95	274.73	101.07	0.09	-13.78

Compound	Normalized Values (A and B Sets)	Responses

Costunolide

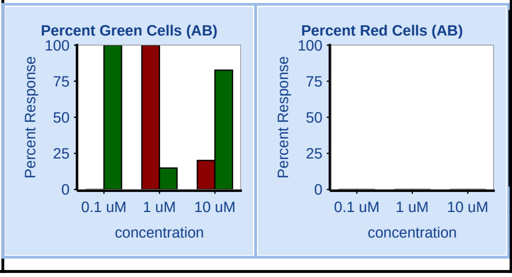

Average Response

Dose	Green Signal	Actual Pct Green Cells	Normalized Pct Green Cells	Red Signal	Actual Pct Red Cells	Normalized Pct Red Cells
0.1 uM	91.01	0.77	-71.30	84.99	0.05	-43.21
1 uM	97.95	0.86	99.39	88.79	0.03	-41.44
10 uM	98.55	0.80	51.37	93.34	0.05	-29.30

Compound	Normalized Values (A and B Sets)	Responses

CP-91149

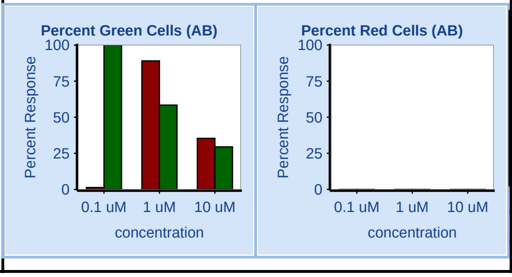

Average Response

Dose	Green Signal	Actual Pct Green Cells	Normalized Pct Green Cells	Red Signal	Actual Pct Red Cells	Normalized Pct Red Cells
0.1 uM	92.94	0.86	65.75	83.47	0.03	-55.45
1 uM	96.89	0.88	73.73	79.60	0.04	-31.83
10 uM	91.18	0.85	32.44	77.85	0.04	-31.37

Compound	Normalized Values (A and B Sets)	Responses

CP-673451

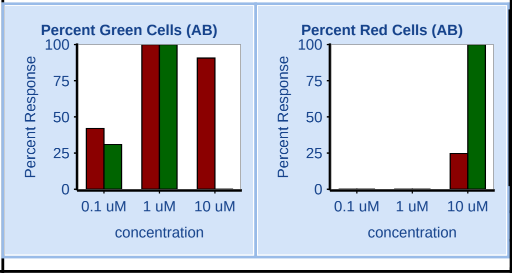

Average Response

Dose	Green Signal	Actual Pct Green Cells	Normalized Pct Green Cells	Red Signal	Actual Pct Red Cells	Normalized Pct Red Cells
0.1 uM	92.47	0.82	36.48	84.84	0.04	-52.58
1 uM	113.68	0.92	120.52	100.13	0.08	-20.61
10 uM	96.88	0.78	28.87	154.78	0.31	84.30

Compound	Normalized Values (A and B Sets)	Responses

CP-724714

Average Response

Dose	Green Signal			Red Signal	Actual Pct Red Cells	

Compound	Normalized Values (A and B Sets)	Responses

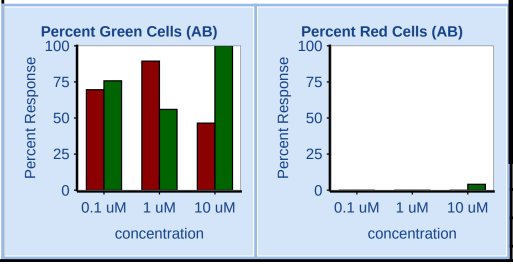

		Actual Pct Green Cells	Normalized Pct Green Cells			Normalized Pct Red Cells
0.1 uM	93.11	0.87	72.68	76.63	0.02	-63.08
1 uM	104.21	0.86	72.68	97.81	0.08	-19.02
10 uM	98.13	0.84	77.94	111.61	0.09	-10.23

Compound	Normalized Values (A and B Sets)	Responses

cPEPCK inhibitor

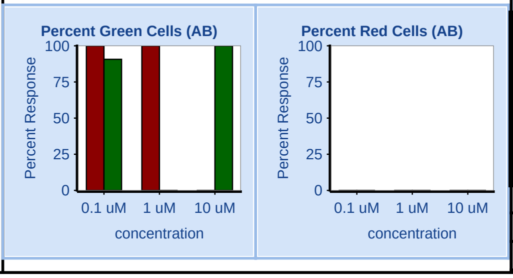

Average Response

Dose	Green Signal	Actual Pct Green Cells	Normalized Pct Green Cells	Red Signal	Actual Pct Red Cells	Normalized Pct Red Cells
0.1 uM	96.71	0.89	103.49	74.33	0.02	-63.46
1 uM	87.12	0.79	61.74	63.05	0.01	-43.56
10 uM	89.02	0.87	51.94	74.40	0.05	-28.48

Compound	Normalized Values (A and B Sets)	Responses

CPI-613

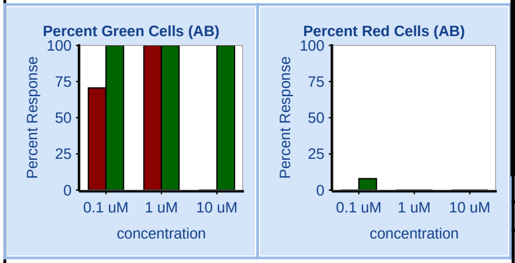

Average Response

Dose	Green Signal	Actual Pct Green Cells	Normalized Pct Green Cells	Red Signal	Actual Pct Red Cells	Normalized Pct Red Cells
0.1 uM	98.81	0.89	100.33	99.06	0.10	-6.89
1 uM	106.38	0.90	103.37	93.96	0.07	-24.20
10 uM	93.66	0.84	-524.73	107.42	0.10	-9.46

Compound	Normalized Values (A and B Sets)	Responses

Crenolanib (CP-868596)

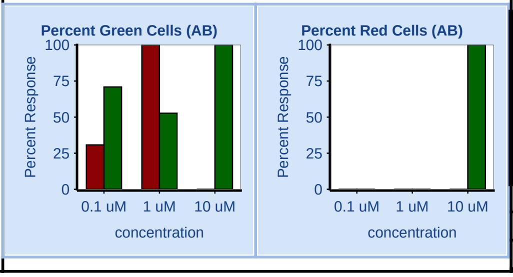

Average Response

Dose	Green Signal	Actual Pct Green Cells	Normalized Pct Green Cells	Red Signal	Actual Pct Red Cells	Normalized Pct Red Cells
0.1 uM	91.55	0.84	50.84	79.41	0.04	-38.38
1 uM	103.80	0.88	86.27	80.59	0.05	-31.96
10 uM	143.22	0.91	-44.15	129.92	0.25	61.15

Compound	Normalized Values (A and B Sets)	Responses

CTEP (RO4956371)

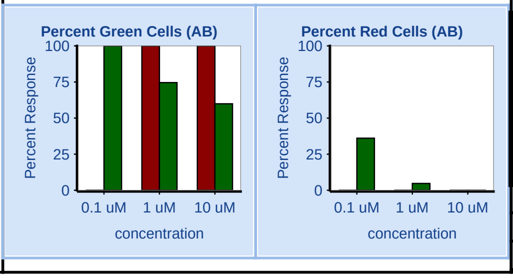

Average Response

Dose	Green Signal	Actual Pct Green Cells	Normalized Pct Green Cells	Red Signal	Actual Pct Red Cells	Normalized Pct Red Cells
0.1 uM	94.48	0.92	76.49	93.96	0.11	-5.54
1 uM	95.28	0.87	108.89	98.01	0.09	-19.83
10 uM	99.12	0.89	157.68	98.83	0.07	-23.69

Compound	Normalized Values (A and B Sets)	Responses

CUDC-101

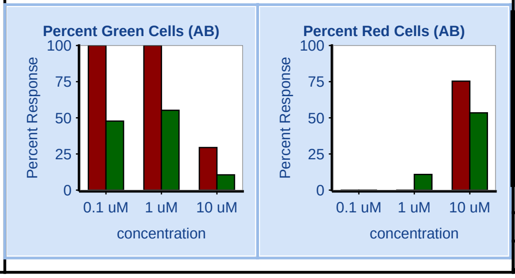

Average Response

Dose	Green Signal	Actual Pct Green Cells	Normalized Pct Green Cells	Red Signal	Actual Pct Red Cells	Normalized Pct Red Cells
0.1 uM	97.26	0.86	82.03	84.52	0.04	-49.92
1 uM	116.48	0.89	117.26	105.30	0.11	-8.56
10 uM	94.35	0.76	20.01	151.28	0.28	64.41

Compound	Normalized Values (A and B Sets)	Responses

CUDC-907

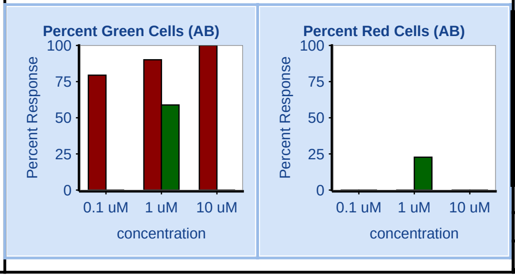

Average Response

Dose	Green Signal	Actual Pct Green Cells	Normalized Pct Green Cells	Red Signal	Actual Pct Red Cells	Normalized Pct Red Cells
0.1 uM	91.73	0.81	5.55	87.75	0.05	-28.63
1 uM	106.24	0.85	74.49	108.86	0.12	-2.04
10 uM	89.86	0.72	152.37	73.89	0.06	-30.55

Compound	Normalized Values (A and B Sets)	Responses

Curcumol

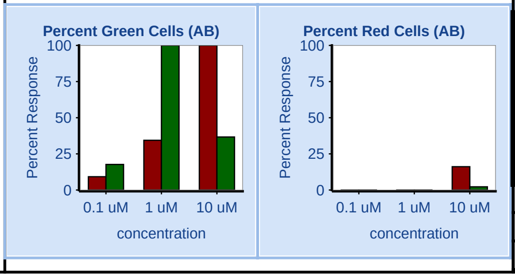

Average Response

Dose	Green Signal	Actual Pct Green Cells	Normalized Pct Green Cells	Red Signal	Actual Pct Red Cells	Normalized Pct Red Cells
0.1 uM	86.72	0.81	13.45	90.38	0.06	-26.77
1 uM	97.08	0.82	69.88	70.95	0.02	-48.99
10 uM	103.31	0.92	366.89	115.93	0.14	9.18

Compound	Normalized Values (A and B Sets)	Responses

CX-4945 (Silmitasertib)

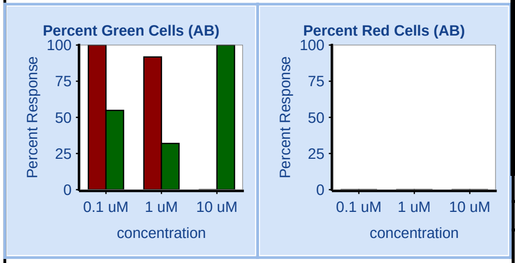

Average Response

Dose	Green Signal	Actual Pct Green Cells	Normalized Pct Green Cells	Red Signal	Actual Pct Red Cells	Normalized Pct Red Cells
0.1 uM	99.57	0.89	88.81	86.62	0.06	-27.01
1 uM	101.01	0.83	61.86	88.95	0.06	-30.13
10 uM	102.16	0.86	-403.24	78.29	0.03	-45.82

Compound	Normalized Values (A and B Sets)	Responses

CYC116

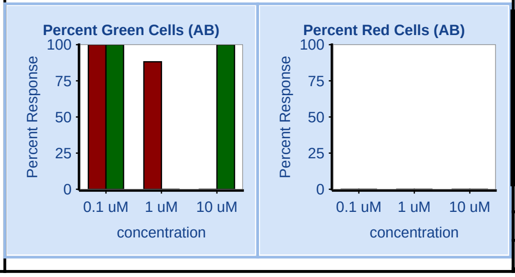

Average Response

Dose	Green Signal	Actual Pct Green Cells	Normalized Pct Green Cells	Red Signal	Actual Pct Red Cells	Normalized Pct Red Cells
0.1 uM	108.69	0.94	124.01	105.45	0.10	-18.05
1 uM	96.14	0.76	11.89	84.77	0.05	-33.70
10 uM	93.95	0.77	37.01	96.52	0.06	-28.81

Compound | Normalized Values (A and B Sets) | Responses

Cyclamic acid

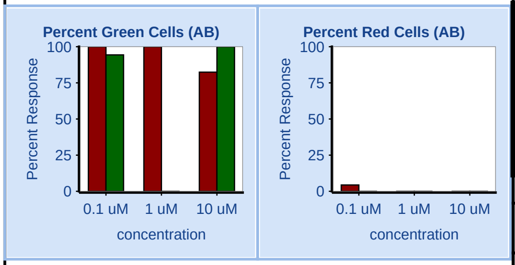

Average Response

Dose	Green Signal	Actual Pct Green Cells	Normalized Pct Green Cells	Red Signal	Actual Pct Red Cells	Normalized Pct Red Cells
0.1 uM	105.77	0.93	251.21	92.54	0.08	-23.35
1 uM	78.62	0.58	-116.44	83.19	0.03	-51.96
10 uM	99.67	0.87	92.37	86.05	0.05	-35.89

Compound | Normalized Values (A and B Sets) | Responses

Cyclandelate

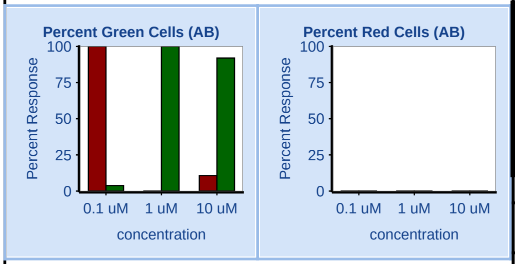

Average Response

Dose	Green Signal	Actual Pct Green Cells	Normalized Pct Green Cells	Red Signal	Actual Pct Red Cells	Normalized Pct Red Cells
0.1 uM	94.40	0.84	58.14	90.32	0.06	-40.42
1 uM	91.31	0.83	-410.50	82.92	0.04	-28.74
10 uM	92.52	0.87	51.39	68.57	0.03	-36.30

Compound | Normalized Values (A and B Sets) | Responses

Cyclizine 2HCl

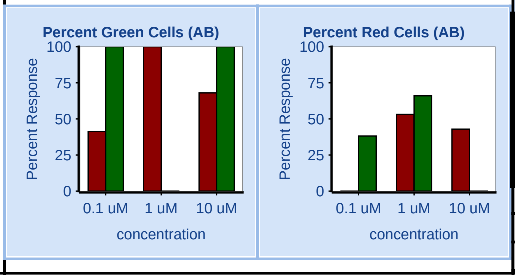

Average Response

Dose	Green Signal	Actual Pct Green Cells	Normalized Pct Green Cells	Red Signal	Actual Pct Red Cells	Normalized Pct Red Cells
0.1 uM	111.53	0.93	102.32	98.46	0.14	7.53
1 uM	92.94	0.73	2.27	122.28	0.25	59.61
10 uM	107.38	0.89	94.04	106.51	0.15	16.61

Compound | Normalized Values (A and B Sets) | Responses

Cyclocytidine HCl

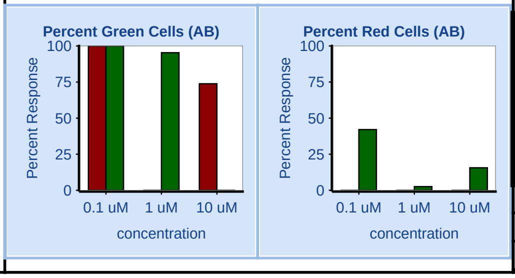

Average Response

Dose	Green Signal	Actual Pct Green Cells	Normalized Pct Green Cells	Red Signal	Actual Pct Red Cells	Normalized Pct Red Cells
0.1 uM	113.70	0.95	145.45	106.02	0.12	-0.74
1 uM	108.45	0.83	-0.98	107.86	0.12	-0.84
10 uM	101.82	0.75	11.55	117.47	0.13	5.21

Compound | Normalized Values (A and B Sets) | Responses

Cyclosporin A

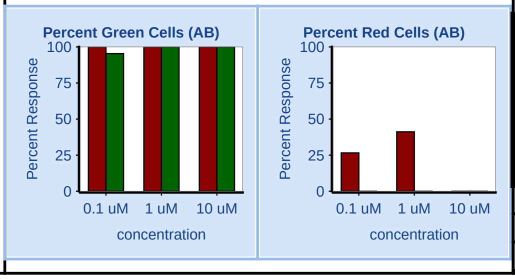

Average Response

Dose	Green Signal	Actual Pct Green Cells	Normalized Pct Green Cells	Red Signal	Actual Pct Red Cells	Normalized Pct Red Cells
0.1 uM	102.81	0.89	98.03	102.98	0.12	0.08
1 uM	112.53	0.91	112.35	104.24	0.14	3.97
10 uM	103.68	0.92	250.89	90.31	0.07	-23.40

Compound	Normalized Values (A and B Sets)	Responses

Cyclosporine

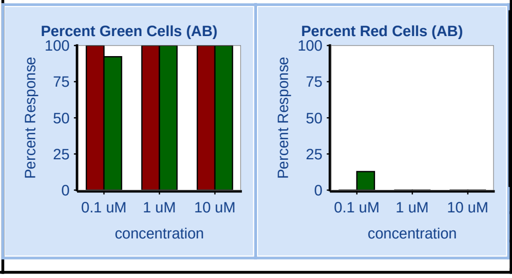

Average Response

Dose	Green Signal	Actual Pct Green Cells	Normalized Pct Green Cells	Red Signal	Actual Pct Red Cells	Normalized Pct Red Cells
0.1 uM	105.43	0.92	127.41	111.95	0.12	-5.64
1 uM	113.20	0.92	132.47	105.65	0.09	-15.35
10 uM	111.50	0.90	114.57	113.03	0.10	-10.99

Compound	Normalized Values (A and B Sets)	Responses

Cyproterone Acetate

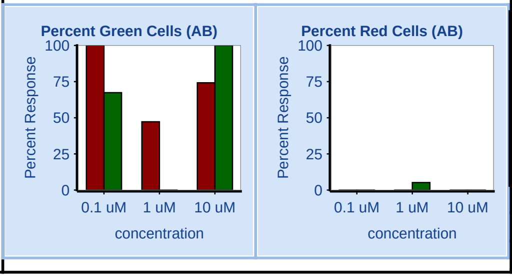

Average Response

Dose	Green Signal	Actual Pct Green Cells	Normalized Pct Green Cells	Red Signal	Actual Pct Red Cells	Normalized Pct Red Cells
0.1 uM	99.35	0.89	102.95	96.82	0.07	-41.37
1 uM	93.55	0.78	10.87	106.92	0.11	-8.79
10 uM	106.94	0.86	87.49	107.39	0.08	-21.29

Compound	Normalized Values (A and B Sets)	Responses

Cyromazine

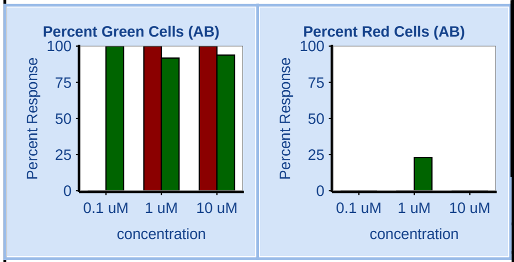

Average Response

Dose	Green Signal	Actual Pct Green Cells	Normalized Pct Green Cells	Red Signal	Actual Pct Red Cells	Normalized Pct Red Cells
0.1 uM	87.42	0.81	-323.81	86.29	0.07	-26.00
1 uM	105.82	0.86	101.39	84.46	0.09	-23.19
10 uM	107.21	0.88	114.27	79.10	0.04	-43.99

Compound	Normalized Values (A and B Sets)	Responses

CYT387

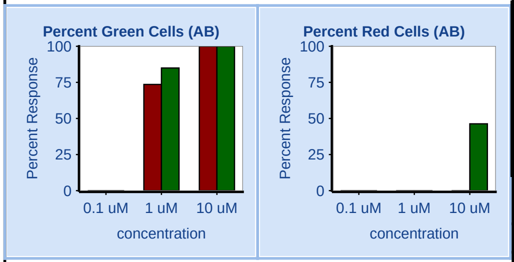

Average Response

Dose	Green Signal	Actual Pct Green Cells	Normalized Pct Green Cells	Red Signal	Actual Pct Red Cells	Normalized Pct Red Cells
0.1 uM	72.73	0.63	-176.03	79.10	0.03	-39.63
1 uM	99.01	0.85	79.28	75.09	0.02	-45.06
10 uM	121.95	0.95	451.11	112.73	0.14	9.35

Compound	Normalized Values (A and B Sets)	Responses

CYT997 (Lexibulin)

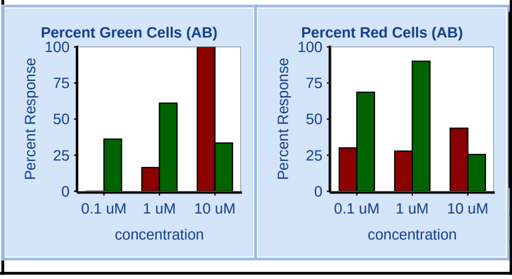

Average Response

Dose	Green Signal	Actual Pct Green Cells	Normalized Pct Green Cells	Red Signal	Actual Pct Red Cells	Normalized Pct Red Cells
0.1 uM	86.93	0.76	-35.26	109.55	0.22	49.32
1 uM	102.42	0.77	38.75	116.53	0.26	58.94
10 uM	105.24	0.89	156.48	109.12	0.19	34.57

Compound	Normalized Values (A and B Sets)	Responses

Cytidine

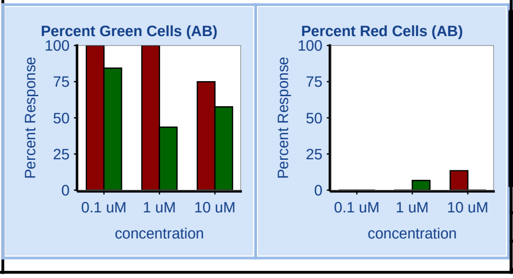

Average Response

Dose	Green Signal	Actual Pct Green Cells	Normalized Pct Green Cells	Red Signal	Actual Pct Red Cells	Normalized Pct Red Cells
0.1 uM	106.95	0.90	103.94	88.35	0.04	-52.86
1 uM	106.31	0.88	110.04	106.74	0.09	-14.24
10 uM	98.23	0.83	66.27	109.43	0.10	-16.46

Compound	Normalized Values (A and B Sets)	Responses

CZC24832

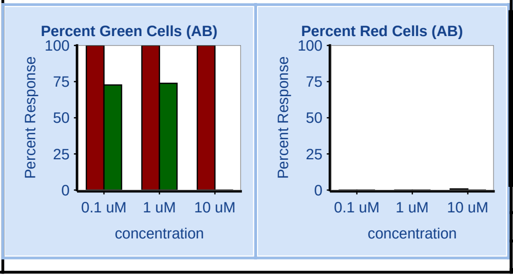

Average Response

Dose	Green Signal	Actual Pct Green Cells	Normalized Pct Green Cells	Red Signal	Actual Pct Red Cells	Normalized Pct Red Cells
0.1 uM	98.96	0.89	104.35	79.02	0.02	-61.64
1 uM	95.66	0.90	181.02	79.69	0.04	-31.55
10 uM	98.45	0.87	60.31	83.61	0.07	-20.00

Compound	Normalized Values (A and B Sets)	Responses

D-Mannitol

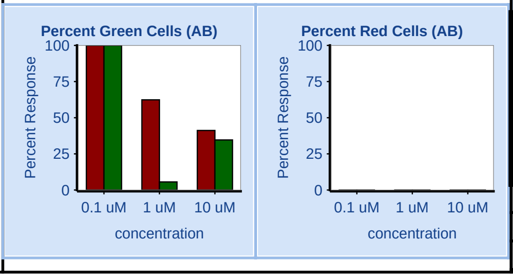

Average Response

Dose	Green Signal	Actual Pct Green Cells	Normalized Pct Green Cells	Red Signal	Actual Pct Red Cells	Normalized Pct Red Cells
0.1 uM	106.98	0.96	164.86	74.02	0.03	-38.07
1 uM	91.35	0.78	33.98	92.23	0.05	-33.12
10 uM	87.77	0.87	37.91	91.19	0.04	-37.53

Compound	Normalized Values (A and B Sets)	Responses

D-Pantothenic acid

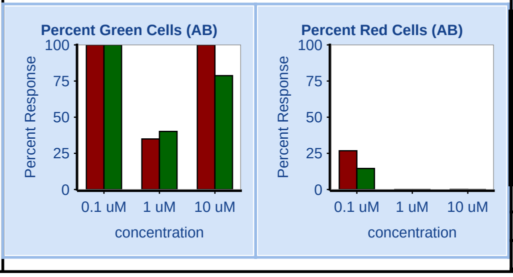

Average Response

Dose	Green Signal	Actual Pct Green Cells	Normalized Pct Green Cells	Red Signal	Actual Pct Red Cells	Normalized Pct Red Cells
0.1 uM	107.48	0.93	138.67	109.81	0.16	20.66
1 uM	93.03	0.77	37.55	73.39	0.03	-44.08
10 uM	102.22	0.91	241.52	95.62	0.08	-17.56

Compound	Normalized Values (A and B Sets)	Responses

Dabigatran Etexilate

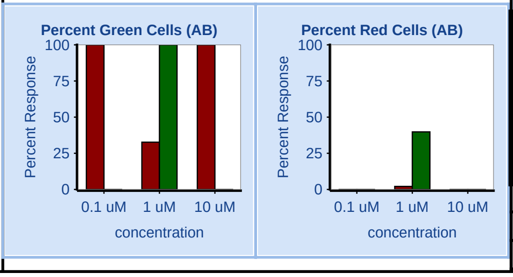

Average Response

Dose	Green Signal	Actual Pct Green Cells	Normalized Pct Green Cells	Red Signal	Actual Pct Red Cells	Normalized Pct Red Cells
0.1 uM	80.33	0.73	-86.01	82.59	0.05	-32.07
1 uM	99.39	0.84	82.10	110.84	0.17	20.94
10 uM	97.03	0.89	318.94	93.20	0.04	-37.74

Compound | Normalized Values (A and B Sets) | Responses

Dabrafenib (GSK2118436)

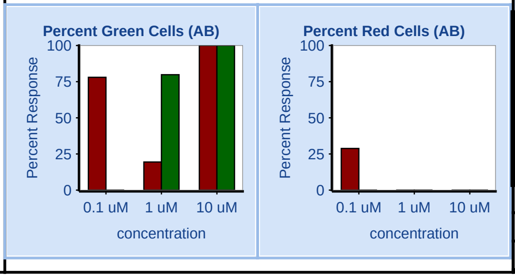

Average Response						
Dose	Green Signal	Actual Pct Green Cells	Normalized Pct Green Cells	Red Signal	Actual Pct Red Cells	Normalized Pct Red Cells
0.1 uM	89.09	0.81	6.45	105.92	0.12	1.45
1 uM	95.08	0.79	49.63	88.69	0.05	-31.47
10 uM	108.31	0.97	451.38	88.80	0.06	-29.88

Compound | Normalized Values (A and B Sets) | Responses

Daclatasvir (BMS-790052)

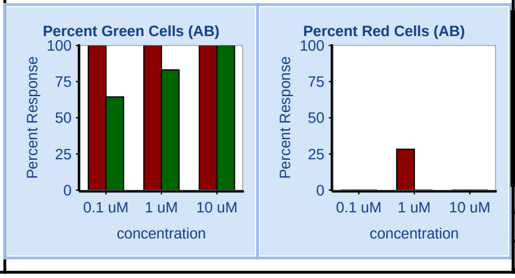

Average Response						
Dose	Green Signal	Actual Pct Green Cells	Normalized Pct Green Cells	Red Signal	Actual Pct Red Cells	Normalized Pct Red Cells
0.1 uM	100.07	0.88	89.88	103.51	0.11	-13.35
1 uM	112.63	0.91	139.91	105.47	0.12	-2.23
10 uM	110.83	0.95	146.93	111.65	0.09	-14.36

Compound | Normalized Values (A and B Sets) | Responses

Dacomitinib (PF299804, PF299)

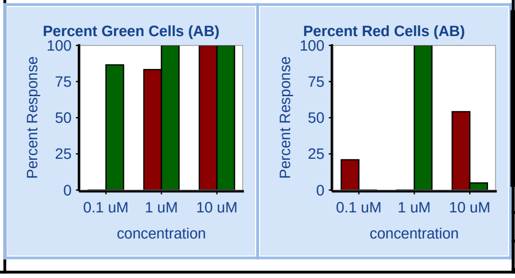

Average Response						
Dose	Green Signal	Actual Pct Green Cells	Normalized Pct Green Cells	Red Signal	Actual Pct Red Cells	Normalized Pct Red Cells
0.1 uM	91.23	0.81	18.10	104.97	0.13	7.28
1 uM	101.38	0.90	109.12	107.77	0.19	29.28
10 uM	116.71	0.98	541.98	121.71	0.18	29.53

Compound | Normalized Values (A and B Sets) | Responses

Dalcetrapib (JTT-705, RO4607381)

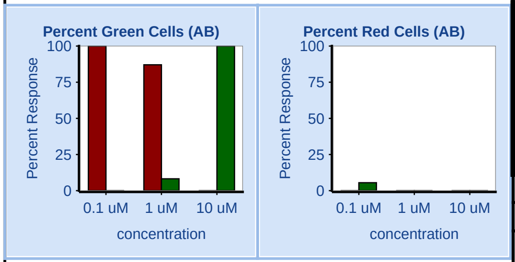

Average Response						
Dose	Green Signal	Actual Pct Green Cells	Normalized Pct Green Cells	Red Signal	Actual Pct Red Cells	Normalized Pct Red Cells
0.1 uM	87.98	0.82	17.81	89.88	0.09	-11.21
1 uM	92.44	0.81	47.63	82.19	0.04	-37.25
10 uM	93.15	0.87	-198.34	74.64	0.02	-46.56

Compound | Normalized Values (A and B Sets) | Responses

Danoprevir (ITMN-191)

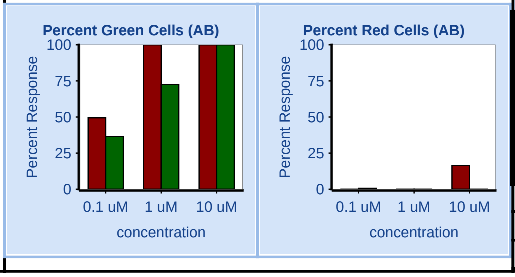

Average Response						
Dose	Green Signal	Actual Pct Green Cells	Normalized Pct Green Cells	Red Signal	Actual Pct Red Cells	Normalized Pct Red Cells
0.1 uM	95.87	0.83	43.00	114.12	0.13	-3.47
1 uM	106.56	0.88	94.00	82.46	0.04	-38.69
10 uM	108.73	0.92	127.27	118.26	0.11	-11.07

Compound | Normalized Values (A and B Sets) | Responses

Danusertib (PHA-739358)

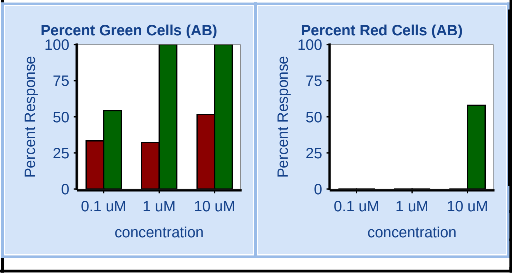

Average Response						
Dose	Green Signal	Actual Pct Green Cells	Normalized Pct Green Cells	Red Signal	Actual Pct Red Cells	Normalized Pct Red Cells
0.1 uM	88.82	0.84	43.83	85.96	0.04	-52.38
1 uM	95.56	0.89	72.97	84.56	0.06	-32.13
10 uM	109.15	0.84	79.57	125.79	0.16	19.62

Compound | Normalized Values (A and B Sets) | Responses

Dapagliflozin

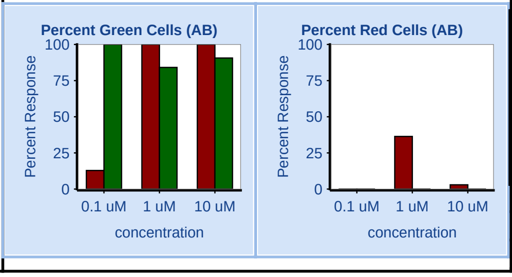

Average Response						
Dose	Green Signal	Actual Pct Green Cells	Normalized Pct Green Cells	Red Signal	Actual Pct Red Cells	Normalized Pct Red Cells
0.1 uM	104.23	0.90	71.23	85.00	0.04	-54.52
1 uM	113.89	0.91	129.21	115.79	0.14	8.08
10 uM	111.02	0.90	110.91	108.05	0.09	-19.24

Compound | Normalized Values (A and B Sets) | Responses

Dapivirine (TMC120)

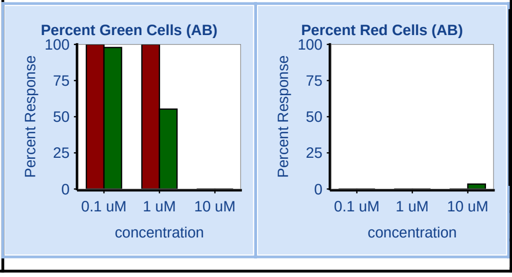

Average Response						
Dose	Green Signal	Actual Pct Green Cells	Normalized Pct Green Cells	Red Signal	Actual Pct Red Cells	Normalized Pct Red Cells
0.1 uM	92.17	0.91	166.76	94.23	0.07	-25.60
1 uM	98.84	0.85	90.04	72.49	0.03	-53.99
10 uM	75.76	0.65	-75.70	93.17	0.10	-12.27

Compound | Normalized Values (A and B Sets) | Responses

Dapoxetine HCl

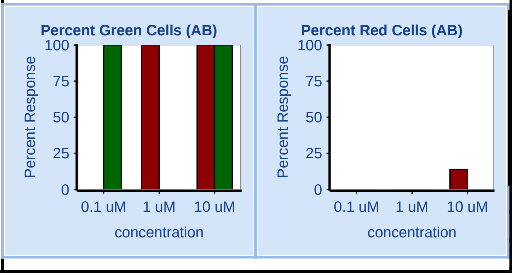

Average Response						
Dose	Green Signal	Actual Pct Green Cells	Normalized Pct Green Cells	Red Signal	Actual Pct Red Cells	Normalized Pct Red Cells
0.1 uM	93.32	0.79	-62.45	84.75	0.05	-45.40
1 uM	93.76	0.74	8.30	78.84	0.05	-33.02
10 uM	112.70	0.92	131.09	113.37	0.11	-9.45

Compound | Normalized Values (A and B Sets) | Responses

DAPT (GSI-IX)

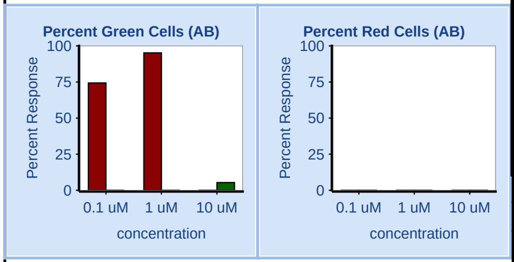

Average Response						
Dose	Green Signal	Actual Pct Green Cells	Normalized Pct Green Cells	Red Signal	Actual Pct Red Cells	Normalized Pct Red Cells
0.1 uM	87.61	0.82	14.19	72.40	0.02	-45.03
1 uM	88.21	0.78	32.77	79.99	0.02	-46.07
10 uM	88.39	0.82	-363.05	80.09	0.03	-42.27

Compound	Normalized Values (A and B Sets)	Responses
**Daptomycin**		

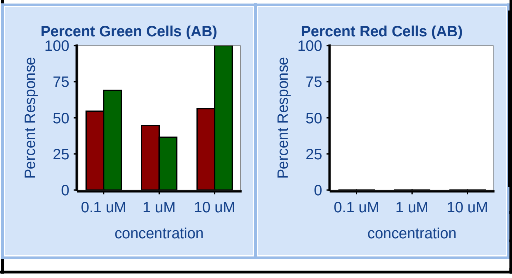

Average Response

Dose	Green Signal	Actual Pct Green Cells	Normalized Pct Green Cells	Red Signal	Actual Pct Red Cells	Normalized Pct Red Cells
0.1 uM	92.21	0.86	61.87	85.18	0.03	-57.81
1 uM	96.48	0.83	40.66	74.90	0.02	-48.17
10 uM	98.89	0.84	80.15	85.81	0.03	-39.66

Compound	Normalized Values (A and B Sets)	Responses
**Darunavir Ethanolate**		

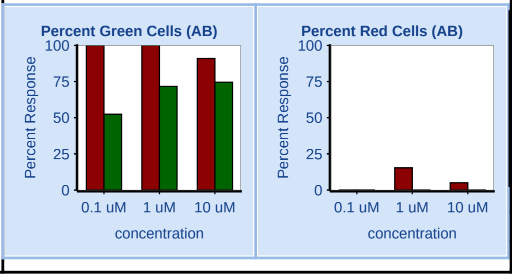

Average Response

Dose	Green Signal	Actual Pct Green Cells	Normalized Pct Green Cells	Red Signal	Actual Pct Red Cells	Normalized Pct Red Cells
0.1 uM	97.67	0.89	107.06	83.03	0.05	-43.03
1 uM	109.35	0.88	98.85	99.93	0.09	-15.52
10 uM	104.71	0.85	82.79	108.81	0.09	-18.52

Compound	Normalized Values (A and B Sets)	Responses
**Dasatinib**		

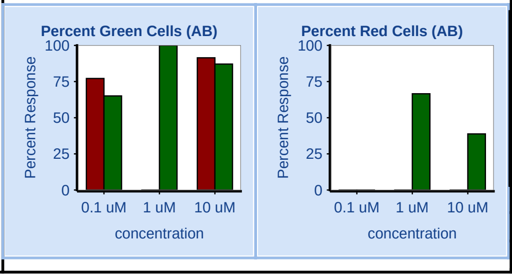

Average Response

Dose	Green Signal	Actual Pct Green Cells	Normalized Pct Green Cells	Red Signal	Actual Pct Red Cells	Normalized Pct Red Cells
0.1 uM	107.97	0.86	71.11	84.00	0.05	-43.65
1 uM	109.91	0.91	65.37	98.96	0.13	26.85
10 uM	104.64	0.91	89.28	83.92	0.12	1.81

Compound	Normalized Values (A and B Sets)	Responses
**DBeQ**		

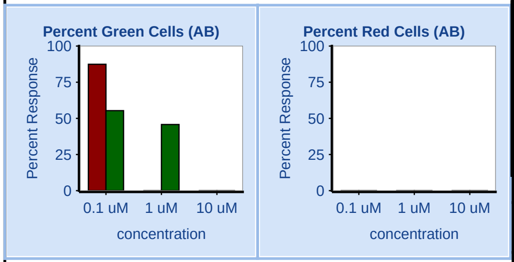

Average Response

Dose	Green Signal	Actual Pct Green Cells	Normalized Pct Green Cells	Red Signal	Actual Pct Red Cells	Normalized Pct Red Cells
0.1 uM	97.39	0.86	71.37	98.86	0.09	-16.26
1 uM	92.33	0.84	-137.77	66.65	0.01	-44.74
10 uM	79.44	0.72	-84.01	96.36	0.06	-26.19

Compound	Normalized Values (A and B Sets)	Responses
**DCC-2036 (Rebastinib)**		

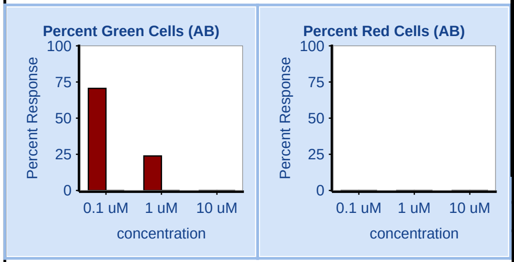

Average Response

Dose	Green Signal	Actual Pct Green Cells	Normalized Pct Green Cells	Red Signal	Actual Pct Red Cells	Normalized Pct Red Cells
0.1 uM	89.57	0.82	21.48	76.35	0.03	-39.89
1 uM	75.98	0.56	-92.36	93.88	0.10	-9.97
10 uM	89.29	0.80	-273.15	90.59	0.05	-33.39

Compound	Normalized Values (A and B Sets)	Responses

Decitabine

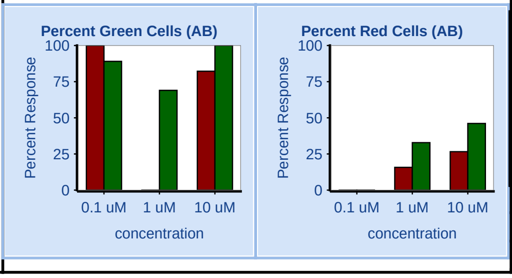

Average Response

Dose	Green Signal	Actual Pct Green Cells	Normalized Pct Green Cells	Red Signal	Actual Pct Red Cells	Normalized Pct Red Cells
0.1 uM	101.61	0.89	106.16	85.72	0.06	-41.29
1 uM	100.65	0.87	16.62	101.11	0.15	24.29
10 uM	109.99	0.92	96.34	104.78	0.20	36.34

Compound	Normalized Values (A and B Sets)	Responses

Deferasirox

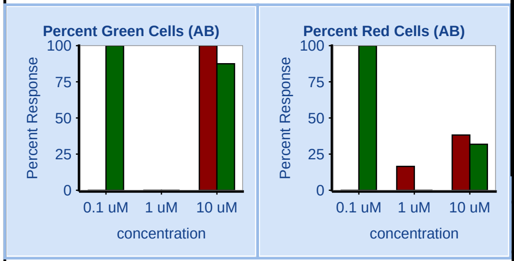

Average Response

Dose	Green Signal	Actual Pct Green Cells	Normalized Pct Green Cells	Red Signal	Actual Pct Red Cells	Normalized Pct Red Cells
0.1 uM	92.99	0.71	-77.39	111.55	0.19	41.03
1 uM	78.73	0.66	-930.64	91.84	0.12	2.85
10 uM	99.74	0.91	94.71	116.10	0.19	35.01

Compound	Normalized Values (A and B Sets)	Responses

Degrasyn (WP1130)

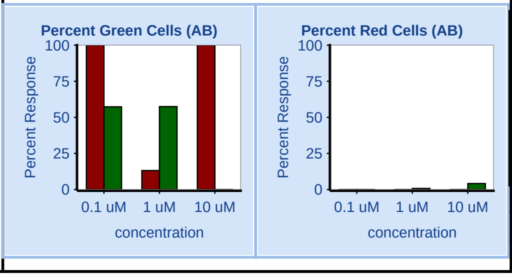

Average Response

Dose	Green Signal	Actual Pct Green Cells	Normalized Pct Green Cells	Red Signal	Actual Pct Red Cells	Normalized Pct Red Cells
0.1 uM	96.29	0.88	86.48	67.45	0.03	-43.32
1 uM	95.28	0.76	35.30	92.44	0.09	-16.37
10 uM	90.03	0.88	234.30	108.93	0.09	-18.06

Compound	Normalized Values (A and B Sets)	Responses

Deoxyarbutin

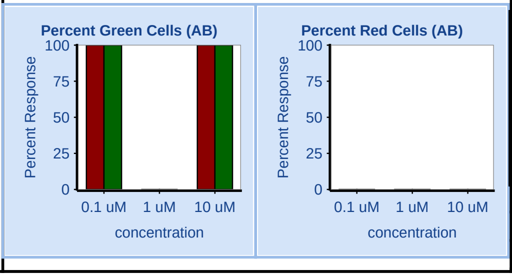

Average Response

Dose	Green Signal	Actual Pct Green Cells	Normalized Pct Green Cells	Red Signal	Actual Pct Red Cells	Normalized Pct Red Cells
0.1 uM	103.54	0.92	151.31	92.36	0.07	-30.31
1 uM	78.43	0.62	-73.84	83.48	0.04	-49.17
10 uM	109.28	0.93	156.30	92.72	0.07	-28.95

Compound	Normalized Values (A and B Sets)	Responses

Deoxycorticosterone acetate

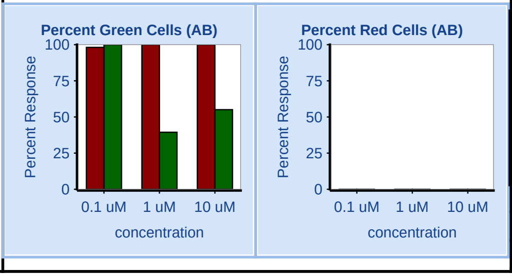

Average Response

Dose	Green Signal	Actual Pct Green Cells	Normalized Pct Green Cells	Red Signal	Actual Pct Red Cells	Normalized Pct Red Cells
0.1 uM	99.03	0.91	107.14	84.20	0.06	-30.95
1 uM	92.86	0.82	73.78	92.05	0.07	-33.28
10 uM	97.97	0.84	90.62	77.54	0.03	-47.21

Compound	Normalized Values (A and B Sets)	Responses

Desmethyl Erlotinib (CP-473420, OSI-774)

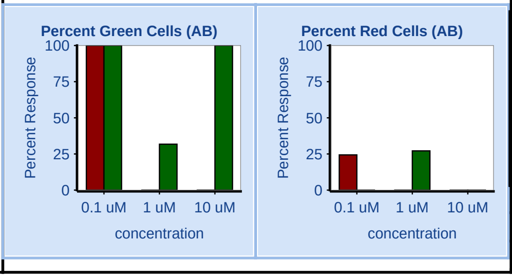

Average Response

Dose	Green Signal	Actual Pct Green Cells	Normalized Pct Green Cells	Red Signal	Actual Pct Red Cells	Normalized Pct Red Cells
0.1 uM	101.43	0.91	121.68	101.26	0.13	7.14
1 uM	77.85	0.64	-24.02	102.21	0.13	2.05
10 uM	92.84	0.89	-86.79	106.95	0.12	-0.24

Compound	Normalized Values (A and B Sets)	Responses

Desonide

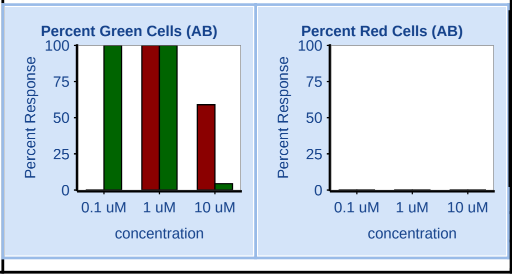

Average Response

Dose	Green Signal	Actual Pct Green Cells	Normalized Pct Green Cells	Red Signal	Actual Pct Red Cells	Normalized Pct Red Cells
0.1 uM	89.03	0.83	44.24	61.48	0.01	-70.96
1 uM	105.16	0.95	316.40	66.85	0.01	-43.33
10 uM	89.10	0.84	31.67	77.07	0.06	-25.49

Compound	Normalized Values (A and B Sets)	Responses

Desvenlafaxine

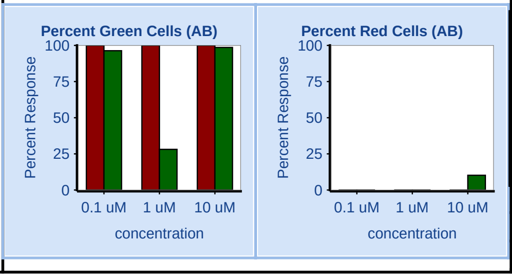

Average Response

Dose	Green Signal	Actual Pct Green Cells	Normalized Pct Green Cells	Red Signal	Actual Pct Red Cells	Normalized Pct Red Cells
0.1 uM	101.90	0.91	184.82	84.16	0.06	-32.03
1 uM	101.57	0.85	89.89	73.34	0.02	-57.32
10 uM	104.06	0.90	139.29	88.95	0.07	-25.21

Compound	Normalized Values (A and B Sets)	Responses

Desvenlafaxine Succinate

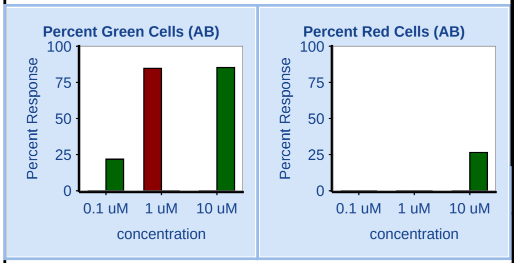

Average Response

Dose	Green Signal	Actual Pct Green Cells	Normalized Pct Green Cells	Red Signal	Actual Pct Red Cells	Normalized Pct Red Cells
0.1 uM	78.03	0.65	-794.59	71.08	0.03	-50.25
1 uM	79.24	0.57	-117.18	80.09	0.04	-45.10
10 uM	93.53	0.80	1.63	104.95	0.11	-6.22

Compound	Normalized Values (A and B Sets)	Responses

Detomidine HCl

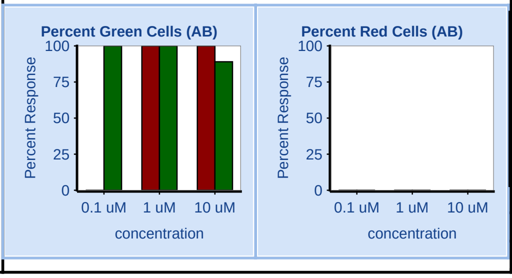

Average Response

Dose	Green Signal	Actual Pct Green Cells	Normalized Pct Green Cells	Red Signal	Actual Pct Red Cells	Normalized Pct Red Cells
0.1 uM	92.43	0.81	-22.42	93.16	0.08	-36.33
1 uM	107.04	0.92	119.83	66.17	0.02	-50.27
10 uM	108.03	0.87	96.21	78.17	0.05	-34.00

Compound	Normalized Values (A and B Sets)	Responses

Dexamethasone (DHAP)

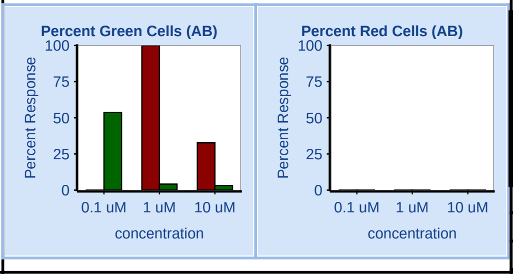

Average Response

Dose	Green Signal	Actual Pct Green Cells	Normalized Pct Green Cells	Red Signal	Actual Pct Red Cells	Normalized Pct Red Cells
0.1 uM	88.35	0.81	4.01	82.95	0.04	-51.62
1 uM	98.42	0.84	90.39	72.51	0.01	-52.00
10 uM	85.60	0.76	17.97	97.79	0.05	-30.33

Compound	Normalized Values (A and B Sets)	Responses

Dexlansoprazole

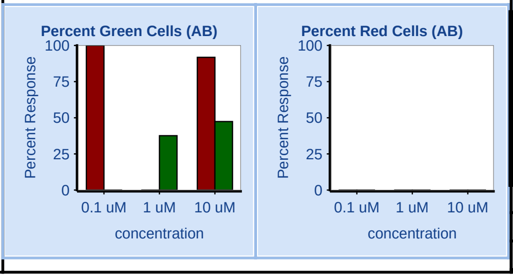

Average Response

Dose	Green Signal	Actual Pct Green Cells	Normalized Pct Green Cells	Red Signal	Actual Pct Red Cells	Normalized Pct Red Cells
0.1 uM	87.68	0.82	48.52	87.51	0.05	-37.20
1 uM	85.55	0.71	-2.12	73.28	0.02	-56.02
10 uM	95.72	0.82	69.60	74.91	0.02	-50.82

Compound	Normalized Values (A and B Sets)	Responses

Dexmedetomidine

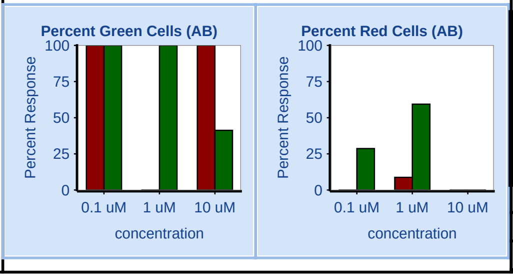

Average Response

Dose	Green Signal	Actual Pct Green Cells	Normalized Pct Green Cells	Red Signal	Actual Pct Red Cells	Normalized Pct Red Cells
0.1 uM	116.47	0.97	166.94	100.05	0.11	-9.58
1 uM	102.22	0.91	26.53	107.10	0.16	34.08
10 uM	102.49	0.89	79.55	71.74	0.06	-26.53

Compound	Normalized Values (A and B Sets)	Responses

Dexmedetomidine HCl

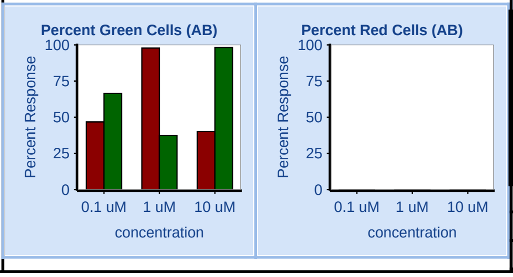

Average Response

Dose	Green Signal	Actual Pct Green Cells	Normalized Pct Green Cells	Red Signal	Actual Pct Red Cells	Normalized Pct Red Cells
0.1 uM	95.21	0.85	56.55	83.79	0.04	-50.30
1 uM	101.97	0.85	67.60	86.22	0.03	-42.94
10 uM	99.28	0.83	69.08	88.16	0.03	-40.70

Compound	Normalized Values (A and B Sets)	Responses

Dexrazoxane HCl (ICRF-187, ADR-529)

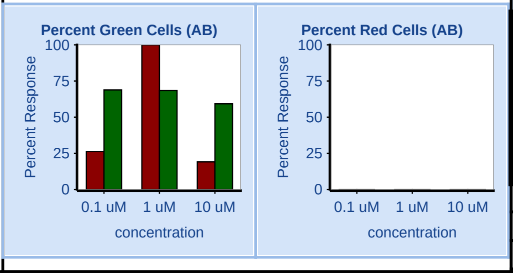

Average Response

Dose	Green Signal	Actual Pct Green Cells	Normalized Pct Green Cells	Red Signal	Actual Pct Red Cells	Normalized Pct Red Cells
0.1 uM	93.99	0.85	47.54	93.54	0.05	-46.92
1 uM	100.54	0.89	109.36	79.18	0.02	-48.18
10 uM	90.42	0.78	39.13	97.95	0.06	-31.89

Compound	Normalized Values (A and B Sets)	Responses

Dextrose

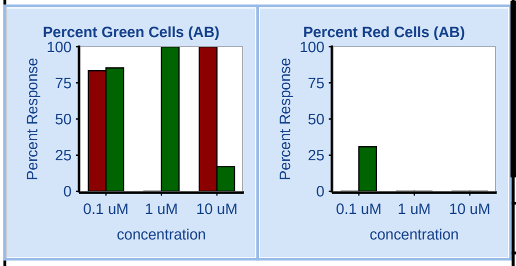

Average Response						
Dose	Green Signal	Actual Pct Green Cells	Normalized Pct Green Cells	Red Signal	Actual Pct Red Cells	Normalized Pct Red Cells
0.1 uM	95.15	0.87	84.36	93.93	0.09	-21.20
1 uM	93.09	0.82	-686.41	71.84	0.03	-37.68
10 uM	99.27	0.88	62.44	74.55	0.04	-35.38

Compound	Normalized Values (A and B Sets)	Responses

DFMO

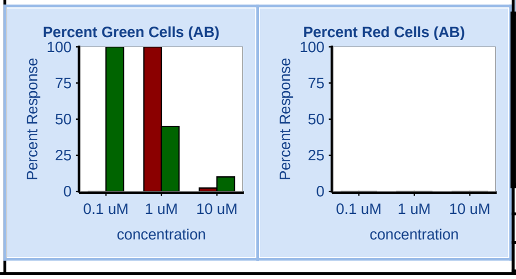

Average Response						
Dose	Green Signal	Actual Pct Green Cells	Normalized Pct Green Cells	Red Signal	Actual Pct Red Cells	Normalized Pct Red Cells
0.1 uM	93.83	0.84	48.44	72.88	0.01	-68.74
1 uM	101.49	0.90	283.92	62.47	0.01	-44.82
10 uM	86.22	0.82	6.15	73.40	0.05	-31.16

Compound	Normalized Values (A and B Sets)	Responses

Dichlorisone Acetate

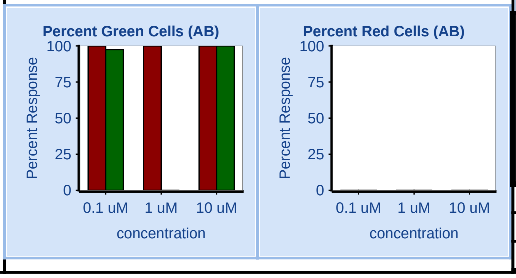

Average Response						
Dose	Green Signal	Actual Pct Green Cells	Normalized Pct Green Cells	Red Signal	Actual Pct Red Cells	Normalized Pct Red Cells
0.1 uM	96.47	0.91	146.16	86.74	0.06	-32.35
1 uM	94.53	0.80	50.25	87.76	0.05	-41.60
10 uM	100.86	0.90	118.92	80.11	0.04	-41.82

Compound	Normalized Values (A and B Sets)	Responses

Diclofenac Diethylamine

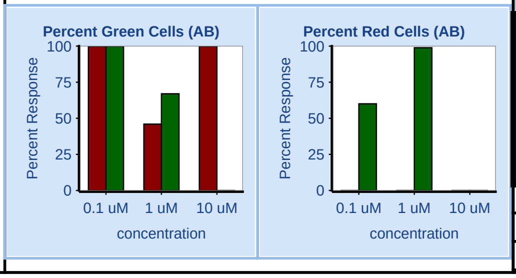

Average Response						
Dose	Green Signal	Actual Pct Green Cells	Normalized Pct Green Cells	Red Signal	Actual Pct Red Cells	Normalized Pct Red Cells
0.1 uM	112.84	0.93	138.02	98.70	0.14	7.86
1 uM	98.56	0.80	56.50	116.40	0.22	42.90
10 uM	86.54	0.69	29.61	79.68	0.03	-48.71

Compound	Normalized Values (A and B Sets)	Responses

Diclofenac Potassium

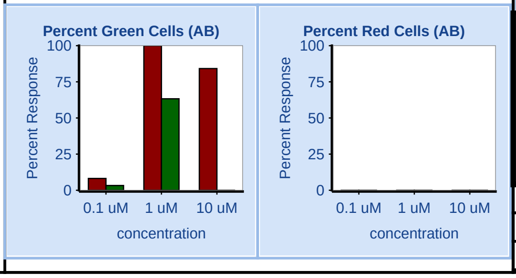

Average Response						
Dose	Green Signal	Actual Pct Green Cells	Normalized Pct Green Cells	Red Signal	Actual Pct Red Cells	Normalized Pct Red Cells
0.1 uM	90.64	0.82	5.74	81.81	0.06	-34.16
1 uM	102.41	0.85	95.51	86.17	0.06	-37.53
10 uM	90.97	0.74	31.55	95.98	0.08	-19.01

Compound	Normalized Values (A and B Sets)	Responses

Dicloxacillin Sodium

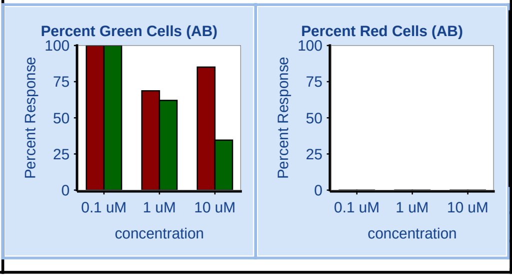

Average Response						
Dose	Green Signal	Actual Pct Green Cells	Normalized Pct Green Cells	Red Signal	Actual Pct Red Cells	Normalized Pct Red Cells
0.1 uM	108.32	0.97	276.13	89.82	0.07	-29.96
1 uM	95.95	0.81	65.37	87.59	0.07	-33.46
10 uM	91.84	0.80	59.83	75.64	0.02	-54.84

Compound	Normalized Values (A and B Sets)	Responses

Dienogest

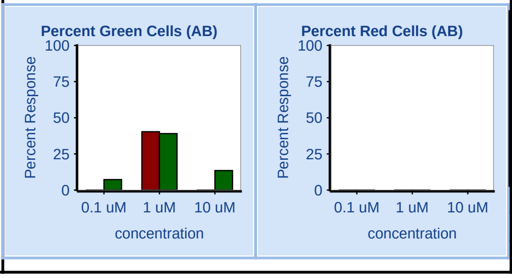

Average Response						
Dose	Green Signal	Actual Pct Green Cells	Normalized Pct Green Cells	Red Signal	Actual Pct Red Cells	Normalized Pct Red Cells
0.1 uM	81.02	0.72	-83.94	84.71	0.04	-51.45
1 uM	94.04	0.83	39.70	111.27	0.10	-11.91
10 uM	84.17	0.72	-0.30	95.06	0.04	-37.38

Compound	Normalized Values (A and B Sets)	Responses

Difluprednate

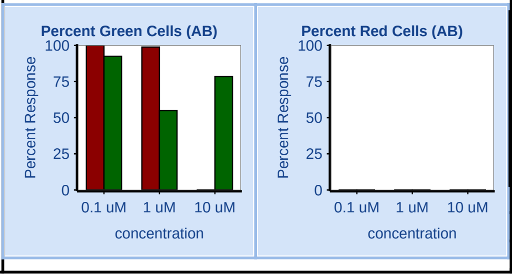

Average Response						
Dose	Green Signal	Actual Pct Green Cells	Normalized Pct Green Cells	Red Signal	Actual Pct Red Cells	Normalized Pct Red Cells
0.1 uM	93.46	0.91	160.74	77.32	0.03	-46.02
1 uM	93.46	0.83	76.89	67.35	0.01	-62.22
10 uM	87.07	0.81	26.39	60.66	0.01	-59.29

Compound	Normalized Values (A and B Sets)	Responses

Dihydroartemisinin (DHA)

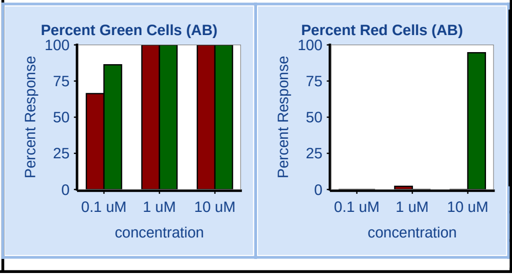

Average Response						
Dose	Green Signal	Actual Pct Green Cells	Normalized Pct Green Cells	Red Signal	Actual Pct Red Cells	Normalized Pct Red Cells
0.1 uM	90.85	0.86	76.27	86.54	0.05	-46.06
1 uM	113.76	0.94	195.88	84.93	0.07	-18.42
10 uM	104.82	0.93	112.20	105.38	0.20	34.65

Compound	Normalized Values (A and B Sets)	Responses

Dihydromyricetin

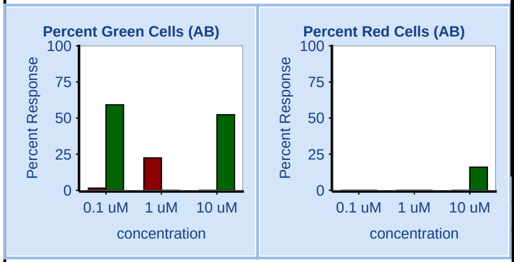

Average Response						
Dose	Green Signal	Actual Pct Green Cells	Normalized Pct Green Cells	Red Signal	Actual Pct Red Cells	Normalized Pct Red Cells
0.1 uM	87.48	0.82	30.34	95.45	0.07	-21.86
1 uM	83.69	0.70	-4.94	84.24	0.04	-37.81
10 uM	90.19	0.85	-160.89	109.01	0.12	-1.00

Compound	Normalized Values (A and B Sets)	Responses

Dimesna

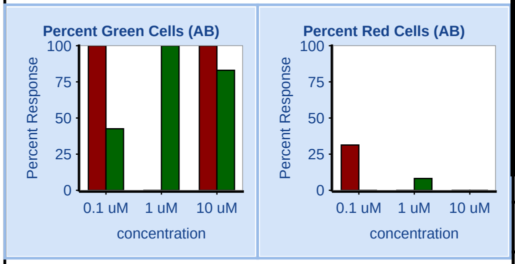

Average Response

Dose	Green Signal	Actual Pct Green Cells	Normalized Pct Green Cells	Red Signal	Actual Pct Red Cells	Normalized Pct Red Cells
0.1 uM	93.68	0.86	74.67	105.33	0.12	-0.27
1 uM	96.47	0.87	-189.27	89.08	0.08	-5.54
10 uM	105.49	0.93	113.99	80.22	0.05	-29.19

Compound	Normalized Values (A and B Sets)	Responses

Dimethyl Fumarate

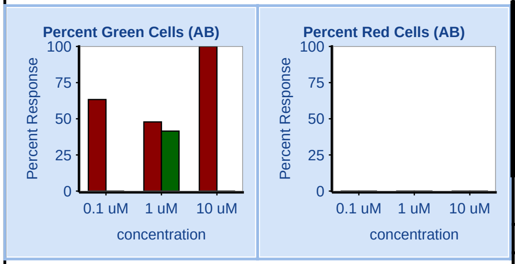

Average Response

Dose	Green Signal	Actual Pct Green Cells	Normalized Pct Green Cells	Red Signal	Actual Pct Red Cells	Normalized Pct Red Cells
0.1 uM	80.12	0.68	-138.51	73.26	0.03	-40.64
1 uM	90.17	0.79	44.65	85.61	0.06	-27.15
10 uM	96.89	0.86	47.62	94.18	0.07	-26.60

Compound	Normalized Values (A and B Sets)	Responses

Diminazene Aceturate

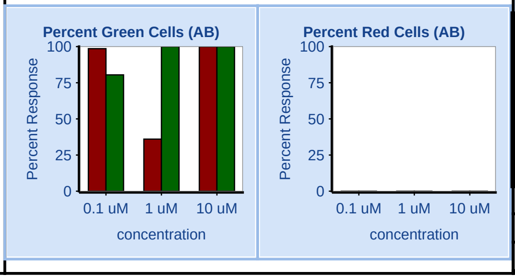

Average Response

Dose	Green Signal	Actual Pct Green Cells	Normalized Pct Green Cells	Red Signal	Actual Pct Red Cells	Normalized Pct Red Cells
0.1 uM	91.54	0.88	89.52	71.04	0.01	-59.44
1 uM	90.17	0.81	69.21	68.96	0.02	-59.64
10 uM	104.42	0.91	151.69	78.32	0.02	-50.53

Compound	Normalized Values (A and B Sets)	Responses

Dinaciclib (SCH727965)

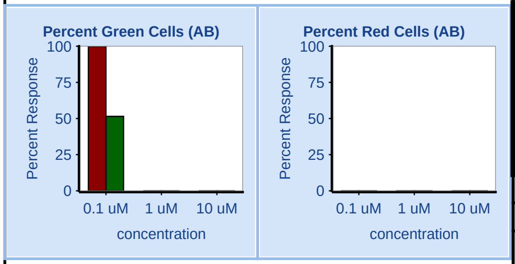

Average Response

Dose	Green Signal	Actual Pct Green Cells	Normalized Pct Green Cells	Red Signal	Actual Pct Red Cells	Normalized Pct Red Cells
0.1 uM	102.71	0.88	81.50	79.64	0.04	-35.99
1 uM	76.32	0.54	-85.13	71.07	0.04	-37.13
10 uM	91.90	0.80	-468.62	89.43	0.07	-24.00

Compound	Normalized Values (A and B Sets)	Responses

Dioscin

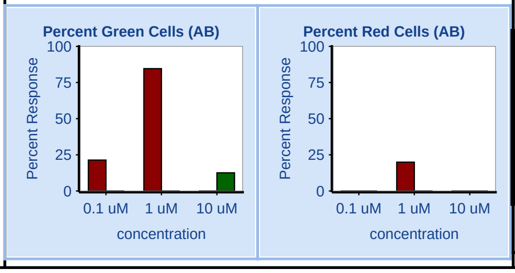

Average Response

Dose	Green Signal	Actual Pct Green Cells	Normalized Pct Green Cells	Red Signal	Actual Pct Red Cells	Normalized Pct Red Cells
0.1 uM	66.76	0.54	-290.71	65.58	0.01	-49.96

Compound	Normalized Values (A and B Sets)	Responses

1 uM	86.73	0.76	18.28	108.48	0.12	-2.17
10 uM	88.26	0.86	-61.45	70.34	0.02	-50.38

Compound	Normalized Values (A and B Sets)	Responses

Dirithromycin

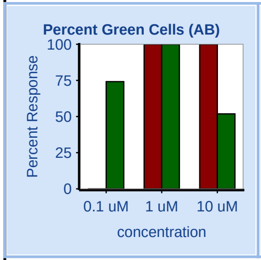

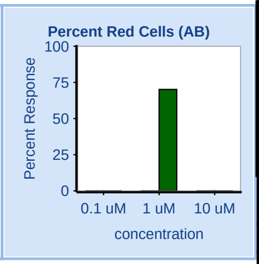

Average Response

Dose	Green Signal	Actual Pct Green Cells	Normalized Pct Green Cells	Red Signal	Actual Pct Red Cells	Normalized Pct Red Cells
0.1 uM	85.15	0.83	-131.22	72.90	0.03	-50.66
1 uM	101.70	0.87	106.59	111.77	0.17	17.20
10 uM	95.72	0.85	117.45	75.24	0.02	-53.67

Compound	Normalized Values (A and B Sets)	Responses

Divalproex Sodium

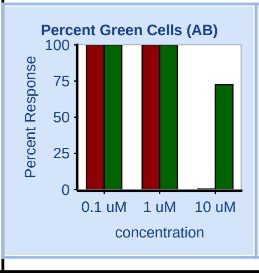

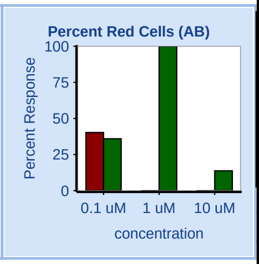

Average Response

Dose	Green Signal	Actual Pct Green Cells	Normalized Pct Green Cells	Red Signal	Actual Pct Red Cells	Normalized Pct Red Cells
0.1 uM	102.96	0.90	112.08	117.09	0.18	38.10
1 uM	104.69	0.92	158.83	113.76	0.24	93.14
10 uM	88.51	0.85	36.36	97.06	0.11	-1.06

Compound	Normalized Values (A and B Sets)	Responses

DMXAA (Vadimezan)

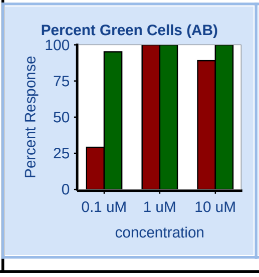

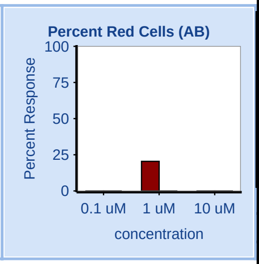

Average Response

Dose	Green Signal	Actual Pct Green Cells	Normalized Pct Green Cells	Red Signal	Actual Pct Red Cells	Normalized Pct Red Cells
0.1 uM	101.66	0.87	62.15	92.99	0.06	-39.35
1 uM	115.67	0.94	146.28	116.47	0.14	8.71
10 uM	101.05	0.88	102.96	105.93	0.07	-23.84

Compound	Normalized Values (A and B Sets)	Responses

Docetaxel

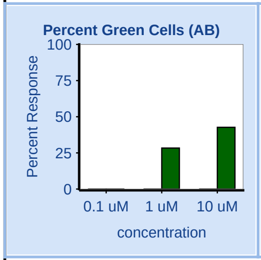

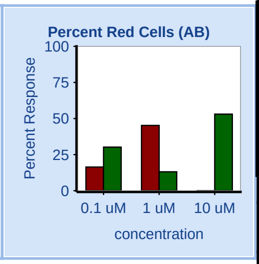

Average Response

Dose	Green Signal	Actual Pct Green Cells	Normalized Pct Green Cells	Red Signal	Actual Pct Red Cells	Normalized Pct Red Cells
0.1 uM	94.77	0.72	-46.52	115.02	0.18	23.31
1 uM	94.78	0.70	-158.81	117.09	0.19	29.15
10 uM	103.00	0.75	20.87	111.95	0.16	21.10

Compound	Normalized Values (A and B Sets)	Responses

Dolutegravir (GSK1349572)

Average Response

Dose	Green Signal			Red Signal	Actual Pct Red Cells	

Compound	Normalized Values (A and B Sets)	Responses

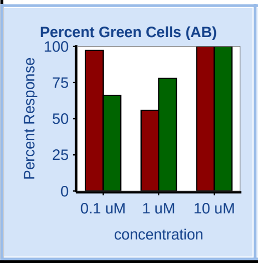

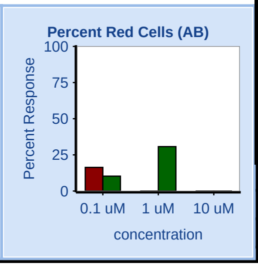

		Actual Pct Green Cells	Normalized Pct Green Cells			Normalized Pct Red Cells
0.1 uM	95.54	0.88	81.62	111.17	0.15	13.32
1 uM	100.57	0.83	66.89	98.69	0.11	-6.59
10 uM	102.90	0.92	200.36	76.54	0.02	-46.46

Compound	Normalized Values (A and B Sets)	Responses

Domiphen Bromide

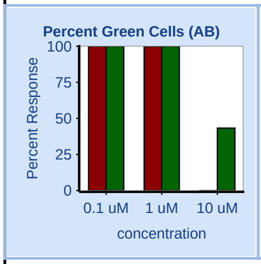

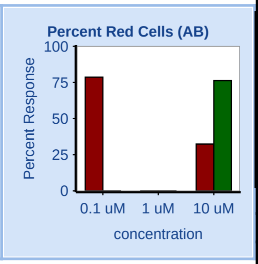

Average Response						
Dose	Green Signal	Actual Pct Green Cells	Normalized Pct Green Cells	Red Signal	Actual Pct Red Cells	Normalized Pct Red Cells
0.1 uM	104.18	0.93	139.77	103.58	0.13	8.26
1 uM	102.98	0.94	287.39	83.11	0.05	-16.98
10 uM	111.64	0.77	-44.07	114.61	0.24	54.34

Compound	Normalized Values (A and B Sets)	Responses

Doripenem Hydrate

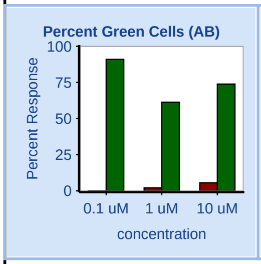

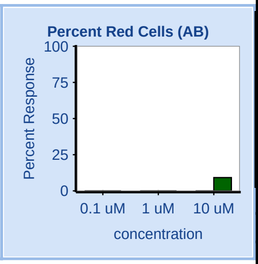

Average Response						
Dose	Green Signal	Actual Pct Green Cells	Normalized Pct Green Cells	Red Signal	Actual Pct Red Cells	Normalized Pct Red Cells
0.1 uM	91.52	0.80	-26.19	96.14	0.06	-41.23
1 uM	93.88	0.84	31.70	88.40	0.05	-35.04
10 uM	90.96	0.78	39.71	112.14	0.09	-9.54

Compound	Normalized Values (A and B Sets)	Responses

Dovitinib (TKI-258) Dilactic Acid

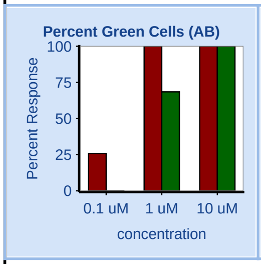

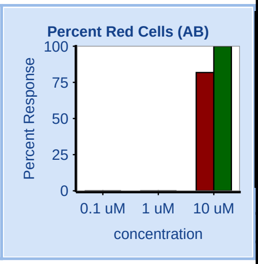

Average Response						
Dose	Green Signal	Actual Pct Green Cells	Normalized Pct Green Cells	Red Signal	Actual Pct Red Cells	Normalized Pct Red Cells
0.1 uM	87.25	0.79	-8.09	74.45	0.03	-41.64
1 uM	103.08	0.89	91.59	89.29	0.08	-22.00
10 uM	125.34	0.99	542.67	174.26	0.45	157.08

Compound	Normalized Values (A and B Sets)	Responses

Dovitinib (TKI-258, CHIR-258)

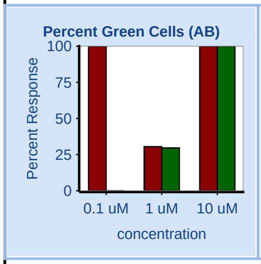

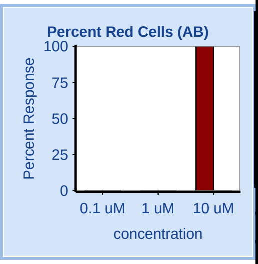

Average Response						
Dose	Green Signal	Actual Pct Green Cells	Normalized Pct Green Cells	Red Signal	Actual Pct Red Cells	Normalized Pct Red Cells
0.1 uM	85.41	0.82	41.39	74.73	0.02	-65.84
1 uM	89.68	0.86	30.08	66.44	0.02	-37.48
10 uM	100.64	0.94	120.94	113.85	0.21	46.71

Compound | Normalized Values (A and B Sets) | Responses

Doxapram HCl

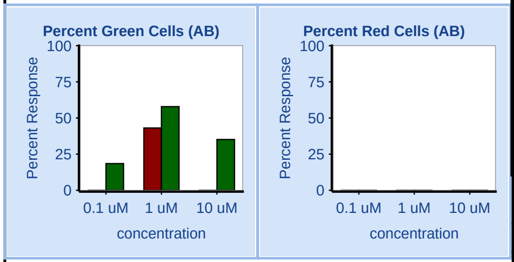

Average Response						
Dose	Green Signal	Actual Pct Green Cells	Normalized Pct Green Cells	Red Signal	Actual Pct Red Cells	Normalized Pct Red Cells
0.1 uM	81.25	0.80	-138.26	69.87	0.02	-52.74
1 uM	93.15	0.79	50.48	77.10	0.04	-46.39
10 uM	84.57	0.72	-77.85	86.18	0.04	-41.72

Compound | Normalized Values (A and B Sets) | Responses

Doxercalciferol

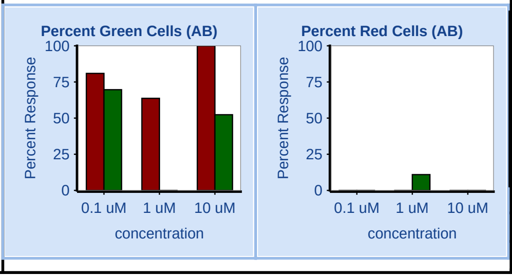

Average Response						
Dose	Green Signal	Actual Pct Green Cells	Normalized Pct Green Cells	Red Signal	Actual Pct Red Cells	Normalized Pct Red Cells
0.1 uM	91.34	0.86	75.29	69.40	0.01	-67.19
1 uM	87.85	0.77	-29.20	92.82	0.09	-1.59
10 uM	102.18	0.90	87.36	71.34	0.04	-34.62

Compound | Normalized Values (A and B Sets) | Responses

Doxifluridine

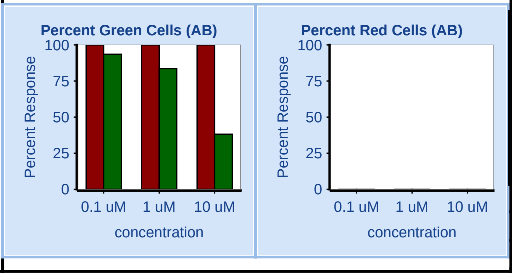

Average Response						
Dose	Green Signal	Actual Pct Green Cells	Normalized Pct Green Cells	Red Signal	Actual Pct Red Cells	Normalized Pct Red Cells
0.1 uM	106.32	0.92	117.44	90.08	0.07	-41.59
1 uM	110.87	0.90	122.88	96.33	0.08	-22.50
10 uM	107.00	0.88	92.65	112.22	0.10	-13.16

Compound | Normalized Values (A and B Sets) | Responses

Doxycycline Hyclate

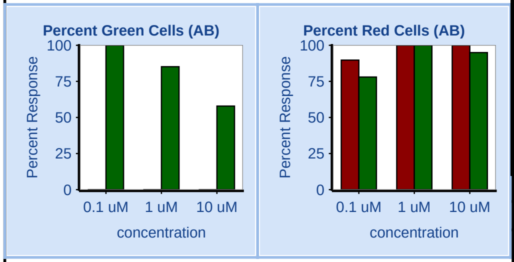

Average Response						
Dose	Green Signal	Actual Pct Green Cells	Normalized Pct Green Cells	Red Signal	Actual Pct Red Cells	Normalized Pct Red Cells
0.1 uM	96.97	0.89	16.95	177.26	0.29	83.89
1 uM	87.15	0.69	-14.35	183.80	0.34	107.72
10 uM	69.39	0.46	-496.17	172.70	0.31	100.68

Compound | Normalized Values (A and B Sets) | Responses

Dronedarone HCl

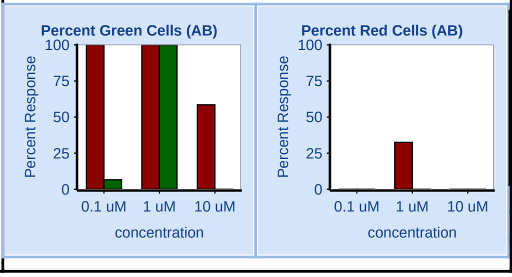

Average Response						
Dose	Green Signal	Actual Pct Green Cells	Normalized Pct Green Cells	Red Signal	Actual Pct Red Cells	Normalized Pct Red Cells
0.1 uM	95.11	0.86	75.50	86.57	0.06	-37.38
1 uM	109.41	0.94	295.55	92.25	0.11	-11.39
10 uM	87.25	0.82	10.73	74.48	0.04	-34.87

Compound | Normalized Values (A and B Sets) | Responses

Drospirenone

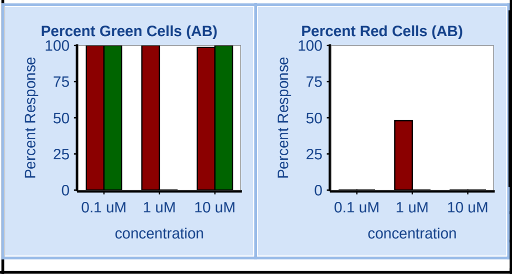

Average Response						
Dose	Green Signal	Actual Pct Green Cells	Normalized Pct Green Cells	Red Signal	Actual Pct Red Cells	Normalized Pct Red Cells
0.1 uM	114.70	0.95	146.03	104.35	0.11	-16.03
1 uM	106.15	0.83	81.82	110.94	0.15	11.47
10 uM	104.51	0.88	104.89	110.05	0.11	-6.25

Compound | Normalized Values (A and B Sets) | Responses

Droxinostat

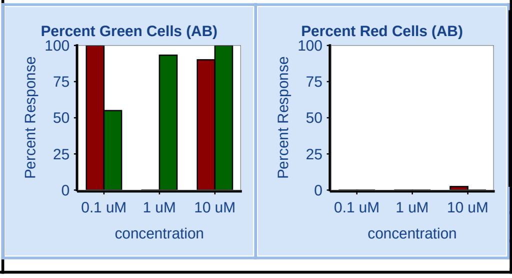

Average Response						
Dose	Green Signal	Actual Pct Green Cells	Normalized Pct Green Cells	Red Signal	Actual Pct Red Cells	Normalized Pct Red Cells
0.1 uM	103.56	0.89	110.14	104.14	0.09	-31.66
1 uM	97.09	0.84	14.76	82.77	0.02	-46.45
10 uM	114.10	0.90	116.43	116.34	0.12	-3.77

Compound | Normalized Values (A and B Sets) | Responses

Dutasteride

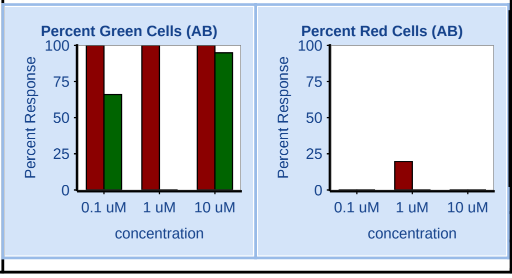

Average Response						
Dose	Green Signal	Actual Pct Green Cells	Normalized Pct Green Cells	Red Signal	Actual Pct Red Cells	Normalized Pct Red Cells
0.1 uM	100.54	0.90	109.66	77.88	0.02	-61.54
1 uM	101.20	0.86	255.57	93.44	0.09	-15.11
10 uM	103.48	0.94	124.06	77.59	0.03	-35.82

Compound | Normalized Values (A and B Sets) | Responses

Dynasore

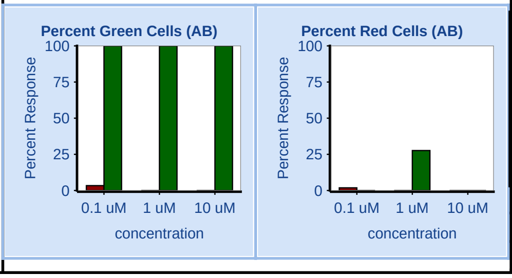

Average Response						
Dose	Green Signal	Actual Pct Green Cells	Normalized Pct Green Cells	Red Signal	Actual Pct Red Cells	Normalized Pct Red Cells
0.1 uM	101.27	0.89	96.73	99.31	0.11	-6.98
1 uM	95.79	0.88	-42.97	89.26	0.09	4.31
10 uM	90.64	0.86	36.49	74.59	0.06	-26.91

Compound | Normalized Values (A and B Sets) | Responses

Ebastine

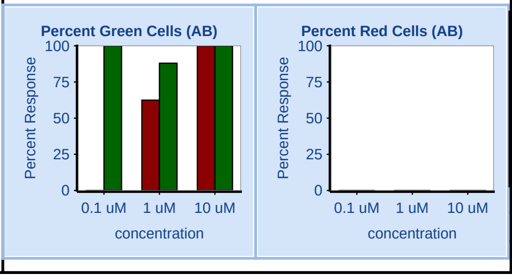

Average Response						
Dose	Green Signal	Actual Pct Green Cells	Normalized Pct Green Cells	Red Signal	Actual Pct Red Cells	Normalized Pct Red Cells
0.1 uM	102.06	0.91	63.42	96.41	0.08	-24.83
1 uM	100.05	0.82	75.22	81.94	0.05	-43.43
10 uM	101.37	0.90	113.08	75.27	0.02	-52.71

Compound	Normalized Values (A and B Sets)	Responses

EBPC

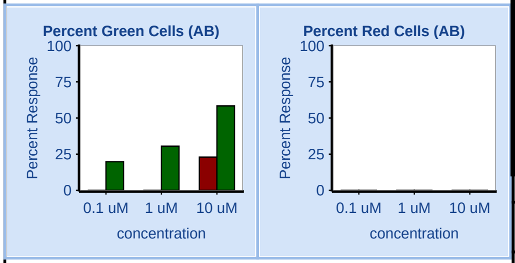

Average Response						
Dose	Green Signal	Actual Pct Green Cells	Normalized Pct Green Cells	Red Signal	Actual Pct Red Cells	Normalized Pct Red Cells
0.1 uM	88.08	0.78	3.45	68.36	0.01	-67.40
1 uM	87.27	0.79	-377.23	78.55	0.04	-34.01
10 uM	89.75	0.86	40.66	70.58	0.03	-39.01

Compound	Normalized Values (A and B Sets)	Responses

Edoxaban

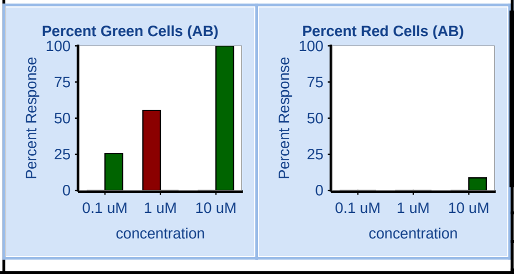

Average Response						
Dose	Green Signal	Actual Pct Green Cells	Normalized Pct Green Cells	Red Signal	Actual Pct Red Cells	Normalized Pct Red Cells
0.1 uM	87.73	0.79	12.13	69.03	0.01	-68.42
1 uM	93.04	0.83	13.16	66.96	0.01	-44.17
10 uM	90.24	0.86	35.75	87.71	0.08	-18.35

Compound	Normalized Values (A and B Sets)	Responses

Efaproxiral Sodium

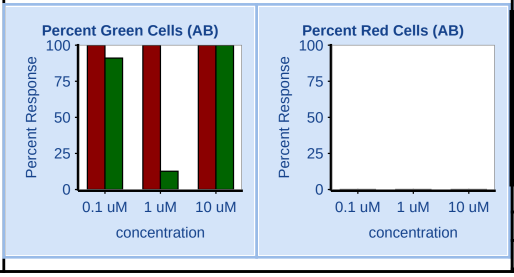

Average Response						
Dose	Green Signal	Actual Pct Green Cells	Normalized Pct Green Cells	Red Signal	Actual Pct Red Cells	Normalized Pct Red Cells
0.1 uM	93.82	0.90	119.54	75.12	0.02	-52.65
1 uM	93.56	0.80	56.72	75.54	0.04	-49.48
10 uM	106.32	0.92	153.26	71.81	0.02	-51.50

Compound	Normalized Values (A and B Sets)	Responses

EHop-016

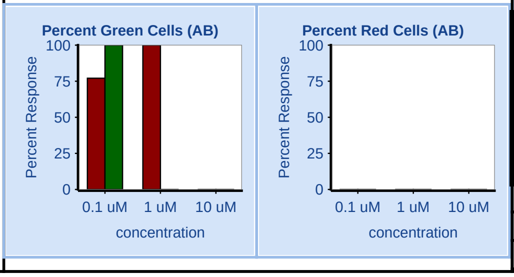

Average Response						
Dose	Green Signal	Actual Pct Green Cells	Normalized Pct Green Cells	Red Signal	Actual Pct Red Cells	Normalized Pct Red Cells
0.1 uM	95.67	0.88	92.17	66.35	0.01	-70.71
1 uM	94.51	0.83	116.33	77.45	0.03	-36.93
10 uM	79.47	0.72	-85.54	74.19	0.02	-40.35

Compound	Normalized Values (A and B Sets)	Responses

Elacridar

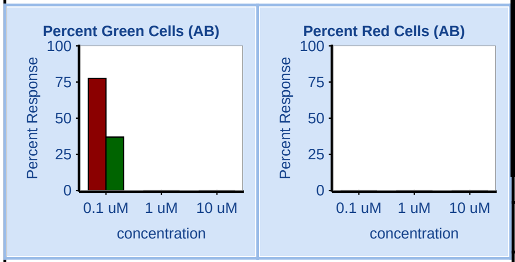

Average Response						
Dose	Green Signal	Actual Pct Green Cells	Normalized Pct Green Cells	Red Signal	Actual Pct Red Cells	Normalized Pct Red Cells
0.1 uM	87.55	0.84	57.20	80.72	0.03	-56.31
1 uM	84.39	0.78	-298.81	66.18	0.01	-41.90
10 uM	80.20	0.75	-53.13	61.67	0.01	-45.17

Compound	Normalized Values (A and B Sets)	Responses

Elaiophylin

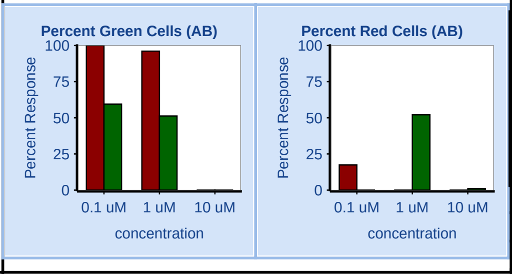

Average Response

Dose	Green Signal	Actual Pct Green Cells	Normalized Pct Green Cells	Red Signal	Actual Pct Red Cells	Normalized Pct Red Cells
0.1 uM	107.46	0.90	124.70	106.46	0.10	-25.09
1 uM	108.80	0.86	73.70	112.97	0.14	7.54
10 uM	86.76	0.65	-58.34	109.65	0.09	-13.05

Compound	Normalized Values (A and B Sets)	Responses

Eletriptan HBr

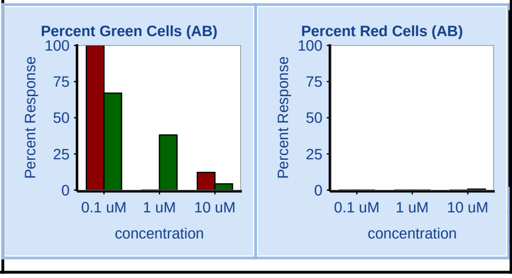

Average Response

Dose	Green Signal	Actual Pct Green Cells	Normalized Pct Green Cells	Red Signal	Actual Pct Red Cells	Normalized Pct Red Cells
0.1 uM	91.88	0.88	129.09	72.57	0.03	-48.65
1 uM	85.54	0.74	15.08	82.64	0.03	-50.04
10 uM	86.73	0.75	8.26	98.56	0.08	-20.87

Compound	Normalized Values (A and B Sets)	Responses

Eltrombopag

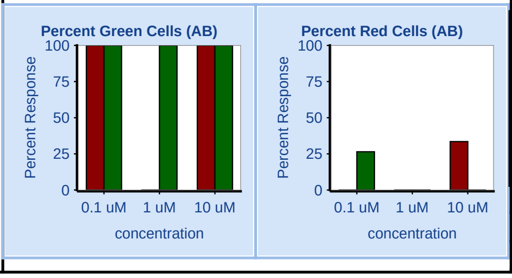

Average Response

Dose	Green Signal	Actual Pct Green Cells	Normalized Pct Green Cells	Red Signal	Actual Pct Red Cells	Normalized Pct Red Cells
0.1 uM	110.30	0.96	166.26	92.41	0.10	-5.80
1 uM	94.28	0.78	52.29	96.61	0.08	-20.11
10 uM	102.15	0.92	274.36	112.16	0.15	13.80

Compound	Normalized Values (A and B Sets)	Responses

Elvitegravir (GS-9137, JTK-303)

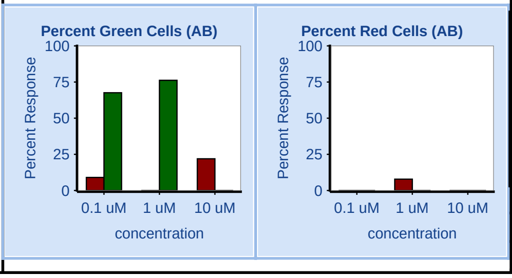

Average Response

Dose	Green Signal	Actual Pct Green Cells	Normalized Pct Green Cells	Red Signal	Actual Pct Red Cells	Normalized Pct Red Cells
0.1 uM	92.49	0.84	38.31	90.02	0.06	-45.44
1 uM	97.16	0.82	-5.92	96.98	0.08	-22.34
10 uM	82.62	0.69	-25.85	95.93	0.06	-26.47

Compound	Normalized Values (A and B Sets)	Responses

EMD 1214063

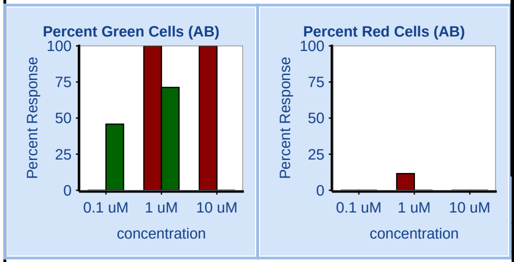

Average Response

Dose	Green Signal	Actual Pct Green Cells	Normalized Pct Green Cells	Red Signal	Actual Pct Red Cells	Normalized Pct Red Cells
0.1 uM	79.52	0.67	-742.84	64.78	0.02	-52.07
1 uM	108.62	0.89	123.73	108.10	0.14	4.22
10 uM	92.17	0.78	70.87	82.14	0.04	-41.65

Compound | Normalized Values (A and B Sets) | Responses

Emetine

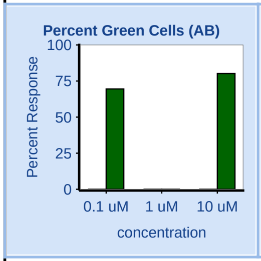

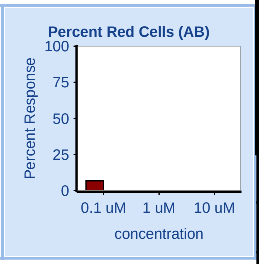

Average Response

Dose	Green Signal	Actual Pct Green Cells	Normalized Pct Green Cells	Red Signal	Actual Pct Red Cells	Normalized Pct Red Cells
0.1 uM	108.15	0.82	-165.76	94.56	0.13	0.84
1 uM	67.39	0.48	-185.19	73.59	0.06	-35.89
10 uM	82.42	0.66	-221.66	76.66	0.05	-38.87

Compound | Normalized Values (A and B Sets) | Responses

Empagliflozin (BI 10773)

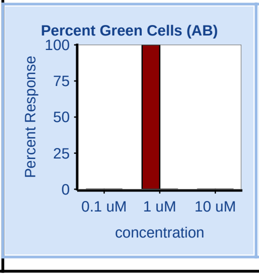

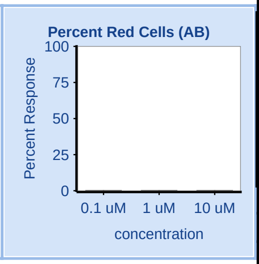

Average Response

Dose	Green Signal	Actual Pct Green Cells	Normalized Pct Green Cells	Red Signal	Actual Pct Red Cells	Normalized Pct Red Cells
0.1 uM	76.53	0.68	-89.44	75.02	0.02	-65.69
1 uM	83.96	0.67	54.80	85.84	0.05	-31.58
10 uM	81.17	0.78	-30.72	67.83	0.01	-44.83

Compound | Normalized Values (A and B Sets) | Responses

Emtricitabine

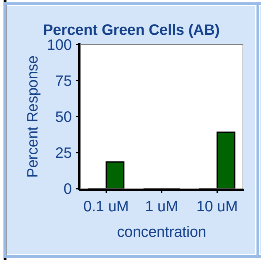

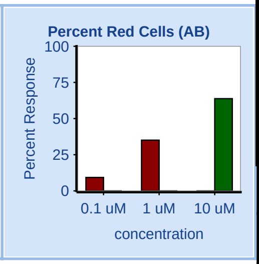

Average Response

Dose	Green Signal	Actual Pct Green Cells	Normalized Pct Green Cells	Red Signal	Actual Pct Red Cells	Normalized Pct Red Cells
0.1 uM	75.12	0.57	-202.28	95.22	0.09	-15.13
1 uM	85.80	0.76	-161.89	97.85	0.12	-2.90
10 uM	90.47	0.82	3.82	104.59	0.19	30.34

Compound | Normalized Values (A and B Sets) | Responses

Enalaprilat Dihydrate

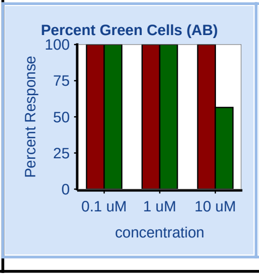

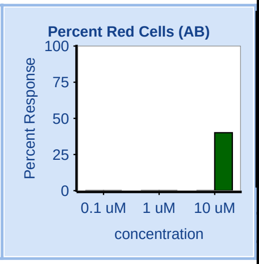

Average Response

Dose	Green Signal	Actual Pct Green Cells	Normalized Pct Green Cells	Red Signal	Actual Pct Red Cells	Normalized Pct Red Cells
0.1 uM	112.47	0.92	111.57	102.39	0.10	-17.25
1 uM	110.56	0.91	122.63	83.13	0.04	-40.01
10 uM	106.15	0.87	89.21	126.83	0.16	18.23

Compound | Normalized Values (A and B Sets) | Responses

ENMD-2076

Average Response

Dose	Green Signal	Actual Pct Green Cells	Normalized Pct Green Cells	Red Signal	Actual Pct Red Cells	Normalized Pct Red Cells
0.1 uM	89.98	0.73	5.56	101.98	0.11	-9.99

Compound	Normalized Values (A and B Sets)	Responses

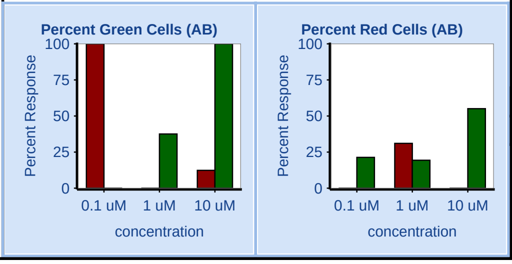

1 uM	95.87	0.80	-4.77	125.09	0.18	25.20
10 uM	110.79	0.83	78.89	141.84	0.18	26.07

Compound	Normalized Values (A and B Sets)	Responses

Enoxolone

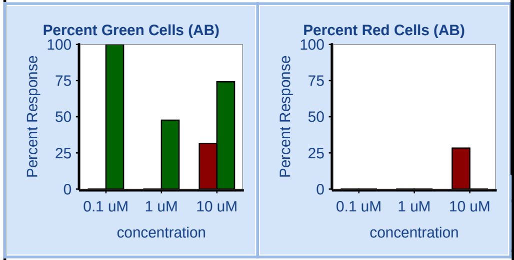

Average Response

Dose	Green Signal	Actual Pct Green Cells	Normalized Pct Green Cells	Red Signal	Actual Pct Red Cells	Normalized Pct Red Cells
0.1 uM	89.32	0.81	28.36	72.38	0.01	-66.95
1 uM	81.70	0.73	-885.90	79.56	0.04	-24.48
10 uM	94.96	0.87	52.91	105.07	0.15	14.14

Compound	Normalized Values (A and B Sets)	Responses

Entecavir Hydrate

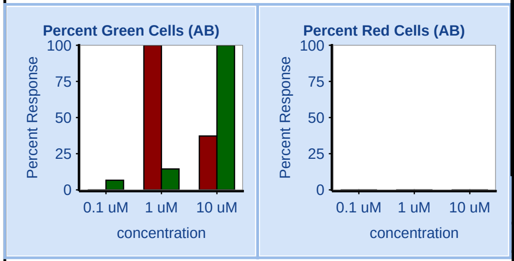

Average Response

Dose	Green Signal	Actual Pct Green Cells	Normalized Pct Green Cells	Red Signal	Actual Pct Red Cells	Normalized Pct Red Cells
0.1 uM	76.08	0.69	-121.53	87.01	0.04	-51.14
1 uM	94.01	0.83	57.85	91.58	0.05	-32.53
10 uM	95.63	0.83	70.72	101.31	0.06	-27.00

Compound	Normalized Values (A and B Sets)	Responses

Enzalutamide (MDV3100)

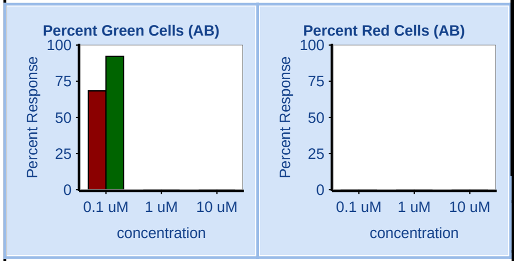

Average Response

Dose	Green Signal	Actual Pct Green Cells	Normalized Pct Green Cells	Red Signal	Actual Pct Red Cells	Normalized Pct Red Cells
0.1 uM	96.68	0.89	80.27	83.66	0.04	-53.52
1 uM	77.44	0.63	-191.88	90.83	0.04	-36.89
10 uM	81.11	0.68	-27.22	71.40	0.01	-46.77

Compound	Normalized Values (A and B Sets)	Responses

Epalrestat

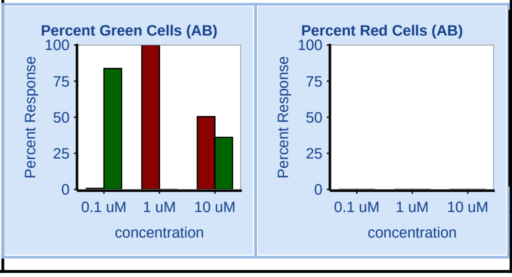

Average Response

Dose	Green Signal	Actual Pct Green Cells	Normalized Pct Green Cells	Red Signal	Actual Pct Red Cells	Normalized Pct Red Cells
0.1 uM	95.56	0.85	42.25	87.09	0.06	-44.06
1 uM	106.67	0.84	90.39	90.92	0.08	-22.02
10 uM	89.10	0.79	43.23	102.77	0.09	-16.18

Compound | Normalized Values (A and B Sets) | Responses

Epinastine HCl

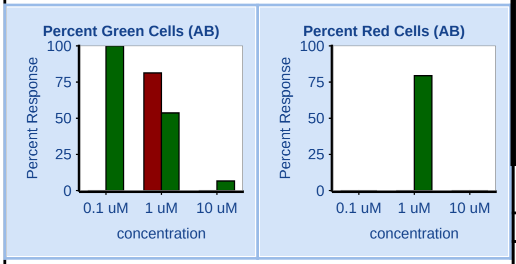

Average Response						
Dose	Green Signal	Actual Pct Green Cells	Normalized Pct Green Cells	Red Signal	Actual Pct Red Cells	Normalized Pct Red Cells
0.1 uM	89.79	0.78	-500.25	90.96	0.10	-14.18
1 uM	90.00	0.81	67.46	111.48	0.18	20.84
10 uM	78.71	0.64	-166.17	87.41	0.08	-19.10

Compound | Normalized Values (A and B Sets) | Responses

Epinephrine Bitartrate

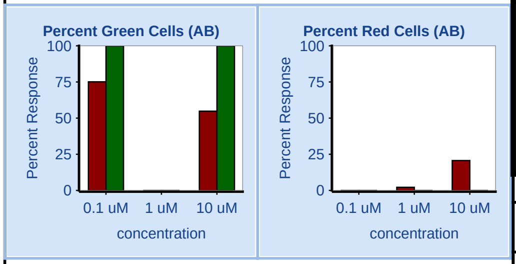

Average Response						
Dose	Green Signal	Actual Pct Green Cells	Normalized Pct Green Cells	Red Signal	Actual Pct Red Cells	Normalized Pct Red Cells
0.1 uM	93.13	0.88	93.02	74.71	0.01	-69.29
1 uM	80.15	0.74	-507.15	89.38	0.06	-24.03
10 uM	104.46	0.92	95.99	101.22	0.13	7.07

Compound | Normalized Values (A and B Sets) | Responses

Epinephrine HCl

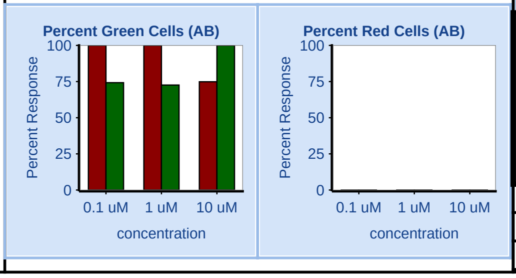

Average Response						
Dose	Green Signal	Actual Pct Green Cells	Normalized Pct Green Cells	Red Signal	Actual Pct Red Cells	Normalized Pct Red Cells
0.1 uM	101.93	0.90	190.53	81.33	0.05	-40.18
1 uM	103.87	0.87	104.30	93.28	0.09	-21.79
10 uM	104.66	0.87	89.36	93.46	0.06	-30.52

Compound | Normalized Values (A and B Sets) | Responses

Epothilone A

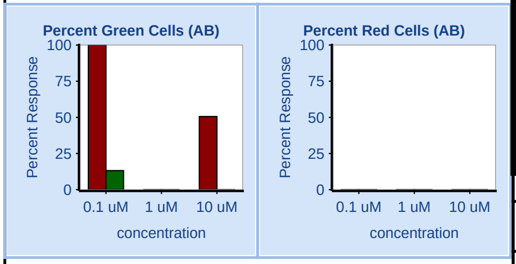

Average Response						
Dose	Green Signal	Actual Pct Green Cells	Normalized Pct Green Cells	Red Signal	Actual Pct Red Cells	Normalized Pct Red Cells
0.1 uM	94.99	0.83	58.41	94.58	0.07	-37.87
1 uM	87.80	0.65	-154.99	88.23	0.07	-27.62
10 uM	95.30	0.76	19.00	110.12	0.10	-10.21

Compound | Normalized Values (A and B Sets) | Responses

Epothilone B (EPO906, Patupilone)

Average Response						
Dose	Green Signal	Actual Pct Green Cells	Normalized Pct Green Cells	Red Signal	Actual Pct Red Cells	Normalized Pct Red Cells
0.1 uM	102.74	0.82	-9.27	112.20	0.14	1.73

Compound	Normalized Values (A and B Sets)	Responses

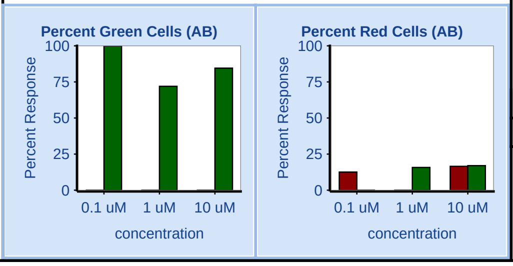

1 uM	113.66	0.83	8.74	95.29	0.12	-2.67
10 uM	105.40	0.74	18.62	110.14	0.16	16.77

Compound	Normalized Values (A and B Sets)	Responses

Eprazinone 2HCl

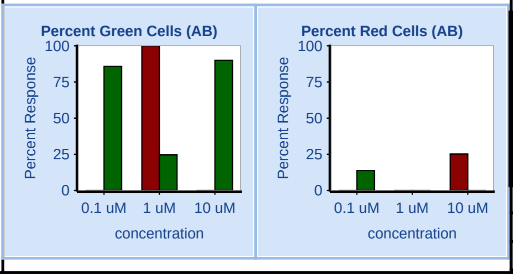

Average Response

Dose	Green Signal	Actual Pct Green Cells	Normalized Pct Green Cells	Red Signal	Actual Pct Red Cells	Normalized Pct Red Cells
0.1 uM	94.58	0.86	-35.69	89.02	0.09	-16.29
1 uM	110.34	0.86	99.48	64.62	0.02	-56.68
10 uM	94.34	0.82	21.92	95.28	0.09	-11.42

Compound	Normalized Values (A and B Sets)	Responses

Eprosartan Mesylate

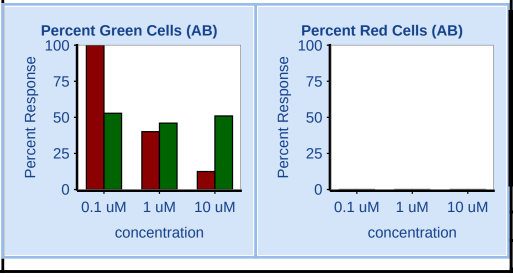

Average Response

Dose	Green Signal	Actual Pct Green Cells	Normalized Pct Green Cells	Red Signal	Actual Pct Red Cells	Normalized Pct Red Cells
0.1 uM	104.42	0.89	193.49	80.03	0.04	-42.63
1 uM	91.18	0.78	43.03	82.80	0.05	-43.04
10 uM	90.87	0.80	31.71	87.97	0.04	-43.05

Compound	Normalized Values (A and B Sets)	Responses

EPZ-6438

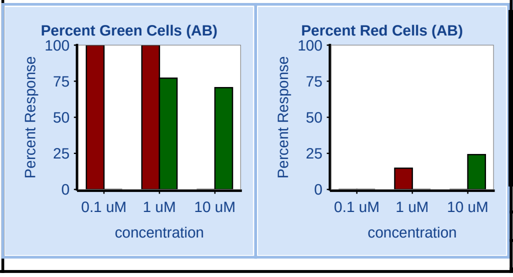

Average Response

Dose	Green Signal	Actual Pct Green Cells	Normalized Pct Green Cells	Red Signal	Actual Pct Red Cells	Normalized Pct Red Cells
0.1 uM	75.32	0.63	-65.13	91.51	0.10	-12.12
1 uM	108.93	0.89	120.57	98.91	0.13	-1.21
10 uM	90.96	0.80	9.43	87.32	0.10	-11.28

Compound	Normalized Values (A and B Sets)	Responses

EPZ004777

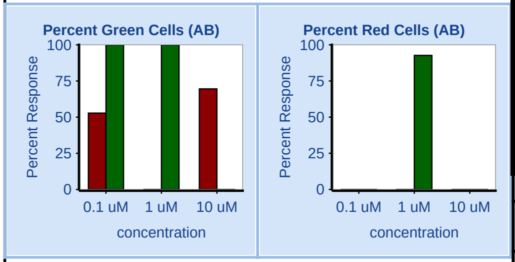

Average Response

Dose	Green Signal	Actual Pct Green Cells	Normalized Pct Green Cells	Red Signal	Actual Pct Red Cells	Normalized Pct Red Cells
0.1 uM	91.47	0.86	76.70	87.34	0.05	-43.06
1 uM	105.11	0.91	-146.32	100.60	0.14	33.60
10 uM	83.84	0.80	-7.90	71.13	0.05	-29.45

Compound	Normalized Values (A and B Sets)	Responses

EPZ004777 HCl

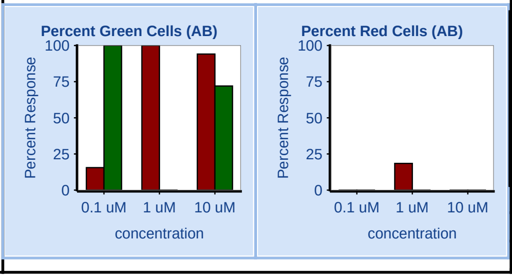

Average Response						
Dose	Green Signal	Actual Pct Green Cells	Normalized Pct Green Cells	Red Signal	Actual Pct Red Cells	Normalized Pct Red Cells
0.1 uM	100.84	0.90	64.02	92.05	0.09	-18.76
1 uM	98.62	0.83	76.16	103.65	0.11	-10.54
10 uM	98.66	0.85	83.05	64.16	0.02	-55.97

Compound	Normalized Values (A and B Sets)	Responses

EPZ5676

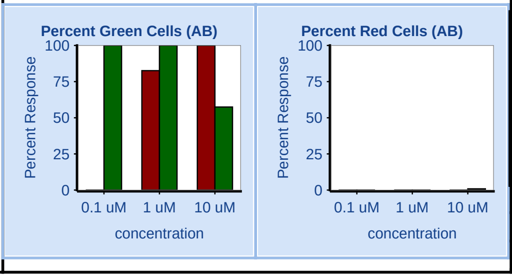

Average Response						
Dose	Green Signal	Actual Pct Green Cells	Normalized Pct Green Cells	Red Signal	Actual Pct Red Cells	Normalized Pct Red Cells
0.1 uM	98.33	0.89	-83.50	85.98	0.07	-29.16
1 uM	109.14	0.87	113.99	90.94	0.08	-24.96
10 uM	100.14	0.85	102.32	96.08	0.11	-7.46

Compound	Normalized Values (A and B Sets)	Responses

EPZ005687

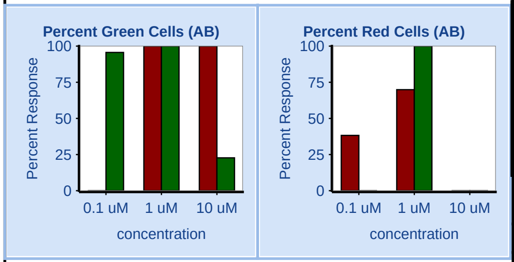

Average Response						
Dose	Green Signal	Actual Pct Green Cells	Normalized Pct Green Cells	Red Signal	Actual Pct Red Cells	Normalized Pct Red Cells
0.1 uM	87.26	0.78	-421.50	100.60	0.15	12.35
1 uM	114.67	0.92	144.13	129.60	0.30	86.86
10 uM	88.62	0.80	61.83	82.87	0.04	-41.28

Compound	Normalized Values (A and B Sets)	Responses

Equol

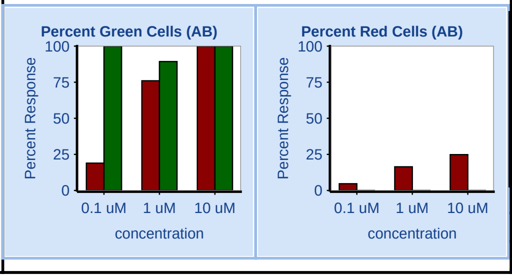

Average Response						
Dose	Green Signal	Actual Pct Green Cells	Normalized Pct Green Cells	Red Signal	Actual Pct Red Cells	Normalized Pct Red Cells
0.1 uM	96.55	0.86	66.01	99.98	0.10	-6.45
1 uM	102.15	0.86	82.67	95.64	0.09	-14.40
10 uM	104.90	0.93	353.30	111.50	0.12	-0.26

Compound	Normalized Values (A and B Sets)	Responses

Erastin

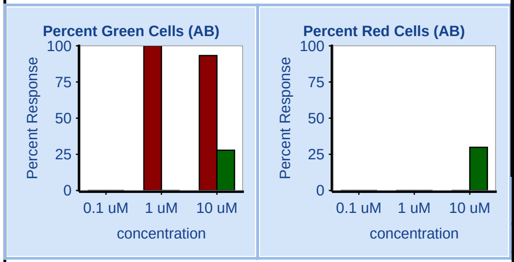

Average Response						
Dose	Green Signal	Actual Pct Green Cells	Normalized Pct Green Cells	Red Signal	Actual Pct Red Cells	Normalized Pct Red Cells
0.1 uM	65.88	0.52	-214.27	70.73	0.01	-67.12
1 uM	87.28	0.76	45.24	63.09	0.02	-38.76
10 uM	89.72	0.87	60.56	78.27	0.10	-7.73

Compound	Normalized Values (A and B Sets)	Responses

Erdosteine

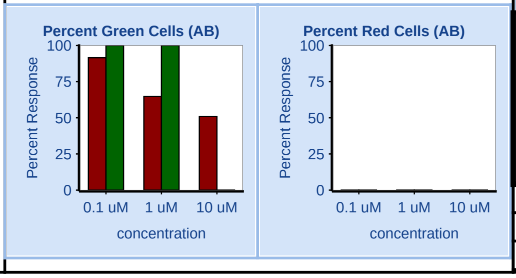

Average Response

Dose	Green Signal	Actual Pct Green Cells	Normalized Pct Green Cells	Red Signal	Actual Pct Red Cells	Normalized Pct Red Cells
0.1 uM	103.91	0.90	96.67	93.68	0.05	-48.25
1 uM	110.12	0.90	89.96	88.19	0.05	-33.66
10 uM	91.13	0.76	18.49	95.09	0.05	-31.44

Compound	Normalized Values (A and B Sets)	Responses

Ergosterol

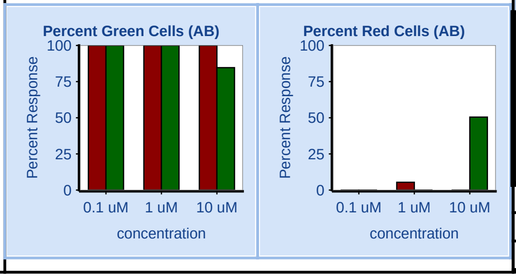

Average Response

Dose	Green Signal	Actual Pct Green Cells	Normalized Pct Green Cells	Red Signal	Actual Pct Red Cells	Normalized Pct Red Cells
0.1 uM	106.19	0.93	138.86	94.03	0.07	-31.36
1 uM	102.44	0.92	145.88	96.99	0.09	-6.11
10 uM	106.35	0.93	112.95	92.34	0.13	5.09

Compound	Normalized Values (A and B Sets)	Responses

Erlotinib HCl (OSI-744)

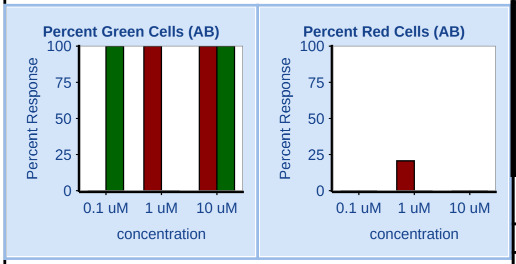

Average Response

Dose	Green Signal	Actual Pct Green Cells	Normalized Pct Green Cells	Red Signal	Actual Pct Red Cells	Normalized Pct Red Cells
0.1 uM	77.85	0.61	-172.21	74.93	0.02	-64.77
1 uM	73.53	0.55	-36.98	89.21	0.09	-16.72
10 uM	92.67	0.94	123.49	79.54	0.04	-31.64

Compound	Normalized Values (A and B Sets)	Responses

Erteberel (LY500307)

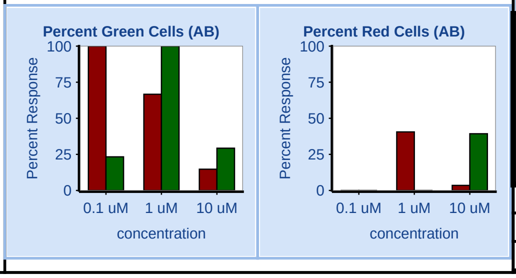

Average Response

Dose	Green Signal	Actual Pct Green Cells	Normalized Pct Green Cells	Red Signal	Actual Pct Red Cells	Normalized Pct Red Cells
0.1 uM	105.51	0.86	86.31	95.69	0.08	-29.41
1 uM	113.33	0.91	98.28	112.65	0.15	8.22
10 uM	102.15	0.76	22.02	114.84	0.17	21.41

Compound	Normalized Values (A and B Sets)	Responses

Erythritol

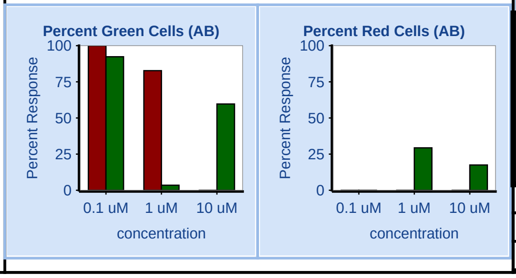

Average Response

Dose	Green Signal	Actual Pct Green Cells	Normalized Pct Green Cells	Red Signal	Actual Pct Red Cells	Normalized Pct Red Cells
0.1 uM	106.11	0.92	212.84	92.01	0.09	-19.09
1 uM	94.05	0.78	43.11	109.48	0.16	14.33
10 uM	94.34	0.79	14.94	102.23	0.13	3.64

Compound | Normalized Values (A and B Sets) | Responses

Erythromycin

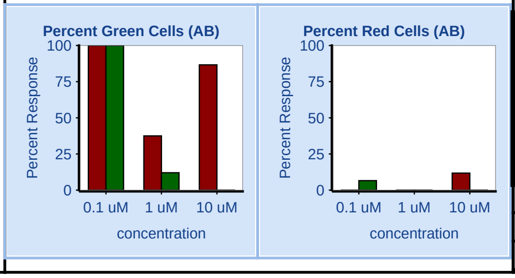

Average Response

Dose	Green Signal	Actual Pct Green Cells	Normalized Pct Green Cells	Red Signal	Actual Pct Red Cells	Normalized Pct Red Cells
0.1 uM	116.01	0.94	142.93	99.39	0.10	-15.31
1 uM	100.39	0.81	24.76	92.25	0.07	-26.19
10 uM	95.21	0.76	18.07	120.76	0.13	0.23

Compound | Normalized Values (A and B Sets) | Responses

Escitalopram Oxalate

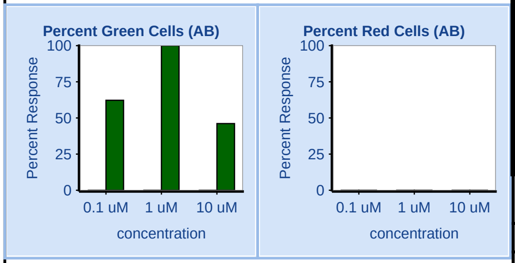

Average Response

Dose	Green Signal	Actual Pct Green Cells	Normalized Pct Green Cells	Red Signal	Actual Pct Red Cells	Normalized Pct Red Cells
0.1 uM	86.30	0.81	-195.35	74.44	0.03	-48.51
1 uM	85.26	0.65	-41.57	70.28	0.02	-56.94
10 uM	84.33	0.76	-25.89	94.00	0.05	-36.60

Compound | Normalized Values (A and B Sets) | Responses

Esomeprazole Magnesium

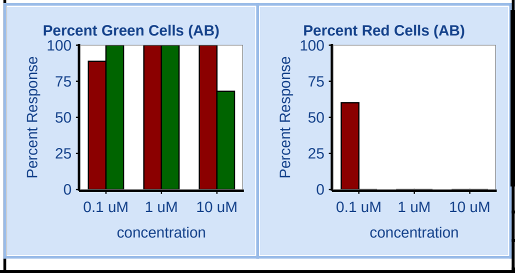

Average Response

Dose	Green Signal	Actual Pct Green Cells	Normalized Pct Green Cells	Red Signal	Actual Pct Red Cells	Normalized Pct Red Cells
0.1 uM	104.50	0.92	106.98	125.54	0.18	15.78
1 uM	112.84	0.94	156.61	90.32	0.05	-32.97
10 uM	102.85	0.88	99.53	108.97	0.08	-16.98

Compound | Normalized Values (A and B Sets) | Responses

Esomeprazole Sodium

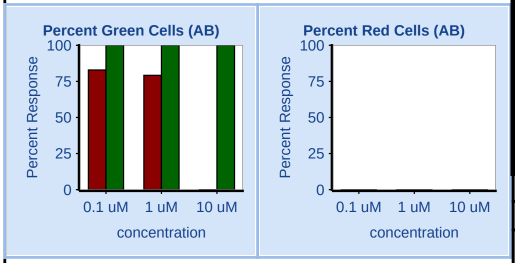

Average Response

Dose	Green Signal	Actual Pct Green Cells	Normalized Pct Green Cells	Red Signal	Actual Pct Red Cells	Normalized Pct Red Cells
0.1 uM	95.37	0.89	95.09	86.26	0.06	-22.69
1 uM	104.89	0.88	97.36	84.84	0.03	-40.22
10 uM	89.55	0.83	-510.53	75.72	0.02	-50.28

Compound | Normalized Values (A and B Sets) | Responses

Estradiol Benzoate

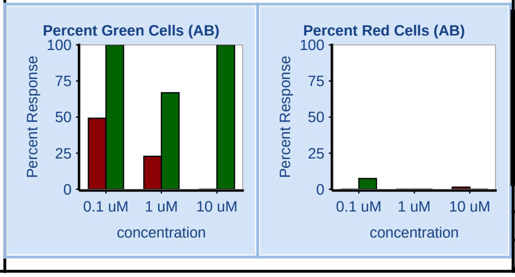

Average Response

Dose	Green Signal	Actual Pct Green Cells	Normalized Pct Green Cells	Red Signal	Actual Pct Red Cells	Normalized Pct Red Cells
0.1 uM	98.85	0.91	86.34	98.10	0.10	-14.53
1 uM	99.44	0.78	44.84	84.61	0.07	-33.74
10 uM	103.74	0.87	59.30	99.06	0.09	-17.33

Compound | Normalized Values (A and B Sets) | Responses

Estradiol Cypionate

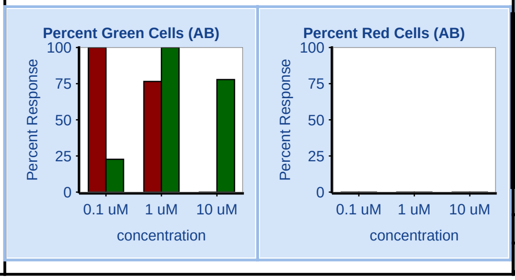

Average Response						
Dose	Green Signal	Actual Pct Green Cells	Normalized Pct Green Cells	Red Signal	Actual Pct Red Cells	Normalized Pct Red Cells
0.1 uM	95.75	0.88	225.59	59.95	0.01	-57.29
1 uM	94.18	0.84	89.93	81.06	0.06	-35.89
10 uM	93.88	0.80	5.28	67.09	0.01	-60.10

Compound | Normalized Values (A and B Sets) | Responses

Ethacridine lactate monohydrate

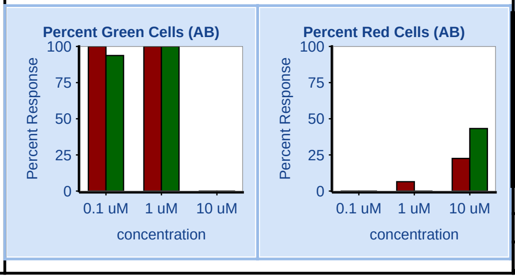

Average Response						
Dose	Green Signal	Actual Pct Green Cells	Normalized Pct Green Cells	Red Signal	Actual Pct Red Cells	Normalized Pct Red Cells
0.1 uM	97.26	0.90	121.86	84.35	0.06	-31.51
1 uM	108.02	0.90	129.00	105.12	0.14	2.41
10 uM	84.96	0.63	-83.51	115.49	0.18	32.91

Compound | Normalized Values (A and B Sets) | Responses

Etodolac

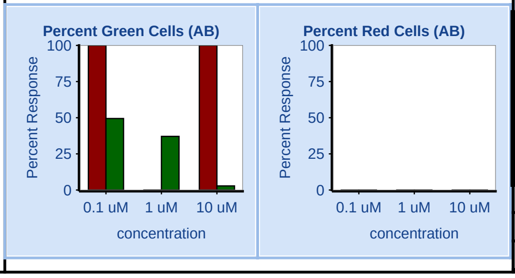

Average Response						
Dose	Green Signal	Actual Pct Green Cells	Normalized Pct Green Cells	Red Signal	Actual Pct Red Cells	Normalized Pct Red Cells
0.1 uM	95.24	0.87	93.52	87.68	0.05	-47.30
1 uM	94.66	0.80	-13.80	91.57	0.07	-27.00
10 uM	100.63	0.82	57.83	103.75	0.07	-24.28

Compound | Normalized Values (A and B Sets) | Responses

Etofibrate

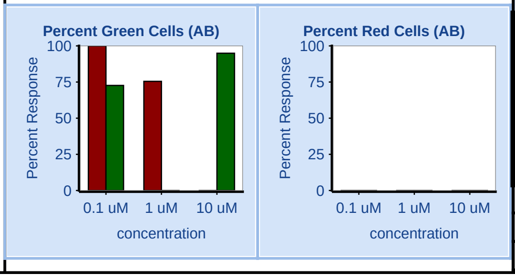

Average Response						
Dose	Green Signal	Actual Pct Green Cells	Normalized Pct Green Cells	Red Signal	Actual Pct Red Cells	Normalized Pct Red Cells
0.1 uM	97.68	0.90	167.52	92.38	0.06	-31.13
1 uM	94.81	0.76	30.00	96.49	0.10	-18.25
10 uM	97.09	0.83	35.39	87.65	0.07	-24.59

Compound | Normalized Values (A and B Sets) | Responses

ETP-46464

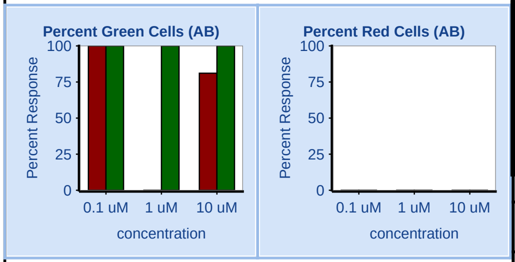

Average Response						
Dose	Green Signal	Actual Pct Green Cells	Normalized Pct Green Cells	Red Signal	Actual Pct Red Cells	Normalized Pct Red Cells
0.1 uM	113.47	0.95	155.25	73.07	0.02	-63.47
1 uM	95.37	0.89	-104.57	80.50	0.05	-16.16
10 uM	101.20	0.94	118.37	78.94	0.04	-34.82

Compound | Normalized Values (A and B Sets) | Responses

Etravirine (TMC125)

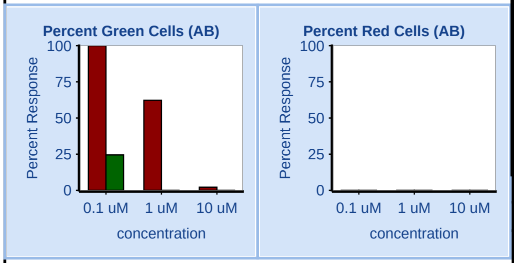

Average Response

Dose	Green Signal	Actual Pct Green Cells	Normalized Pct Green Cells	Red Signal	Actual Pct Red Cells	Normalized Pct Red Cells
0.1 uM	94.86	0.84	63.18	87.72	0.05	-41.85
1 uM	93.16	0.83	24.50	81.70	0.04	-31.84
10 uM	77.45	0.69	-110.87	80.61	0.07	-21.26

Compound | Normalized Values (A and B Sets) | Responses

EUK 134

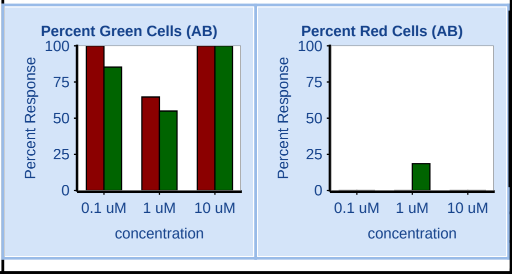

Average Response

Dose	Green Signal	Actual Pct Green Cells	Normalized Pct Green Cells	Red Signal	Actual Pct Red Cells	Normalized Pct Red Cells
0.1 uM	99.09	0.90	162.30	99.16	0.11	-9.57
1 uM	97.85	0.80	59.77	102.60	0.13	-1.95
10 uM	105.04	0.90	122.35	102.32	0.10	-8.26

Compound | Normalized Values (A and B Sets) | Responses

Evacetrapib (LY2484595)

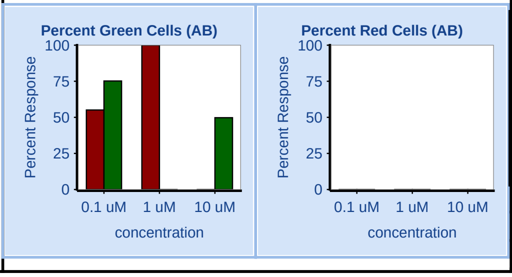

Average Response

Dose	Green Signal	Actual Pct Green Cells	Normalized Pct Green Cells	Red Signal	Actual Pct Red Cells	Normalized Pct Red Cells
0.1 uM	90.41	0.87	65.15	68.91	0.02	-52.15
1 uM	90.62	0.75	10.28	67.83	0.02	-60.16
10 uM	90.98	0.79	23.64	62.36	0.01	-58.15

Compound | Normalized Values (A and B Sets) | Responses

Everolimus (RAD001)

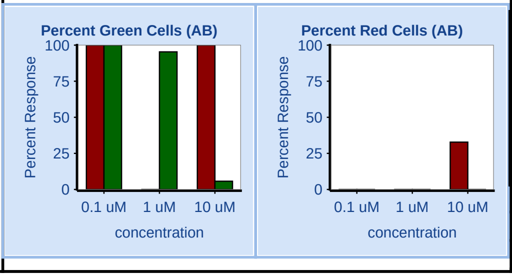

Average Response

Dose	Green Signal	Actual Pct Green Cells	Normalized Pct Green Cells	Red Signal	Actual Pct Red Cells	Normalized Pct Red Cells
0.1 uM	105.71	0.95	133.76	83.89	0.05	-51.64
1 uM	91.26	0.83	-9.51	91.10	0.04	-38.10
10 uM	99.91	0.87	84.93	122.26	0.13	-5.00

Compound | Normalized Values (A and B Sets) | Responses

Evodiamine

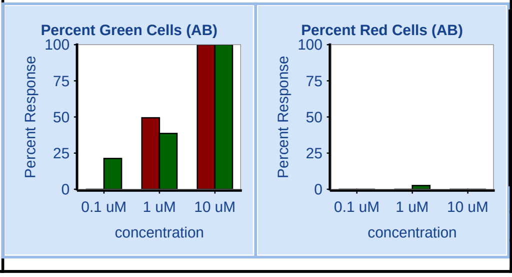

Average Response

Dose	Green Signal	Actual Pct Green Cells	Normalized Pct Green Cells	Red Signal	Actual Pct Red Cells	Normalized Pct Red Cells
0.1 uM	79.81	0.76	-32.73	69.32	0.01	-48.80
1 uM	95.11	0.79	44.03	93.63	0.09	-15.35
10 uM	112.94	0.95	346.71	87.98	0.06	-32.18

Compound | Normalized Values (A and B Sets) | Responses

EX 527 (Selisistat)

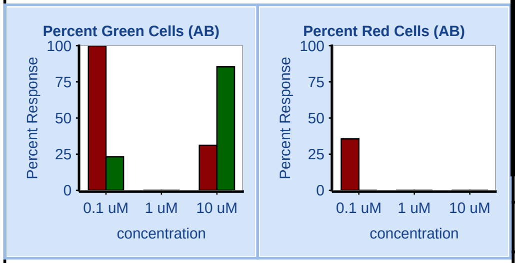

Average Response						
Dose	Green Signal	Actual Pct Green Cells	Normalized Pct Green Cells	Red Signal	Actual Pct Red Cells	Normalized Pct Red Cells
0.1 uM	102.65	0.86	90.50	113.46	0.17	10.37
1 uM	72.71	0.54	-250.72	95.49	0.09	-15.90
10 uM	96.91	0.81	58.28	98.13	0.08	-22.36

Compound | Normalized Values (A and B Sets) | Responses

Farnesyl Thiosalicylic Acid

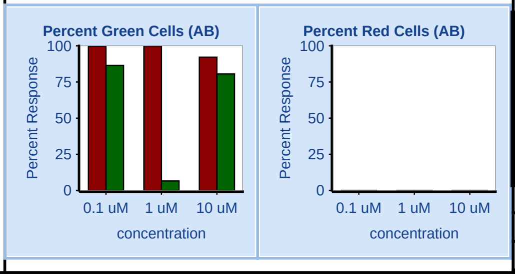

Average Response						
Dose	Green Signal	Actual Pct Green Cells	Normalized Pct Green Cells	Red Signal	Actual Pct Red Cells	Normalized Pct Red Cells
0.1 uM	93.75	0.88	93.16	71.93	0.01	-67.53
1 uM	93.47	0.87	162.62	72.31	0.02	-39.08
10 uM	95.35	0.90	86.38	62.53	0.03	-37.90

Compound | Normalized Values (A and B Sets) | Responses

Fasudil (HA-1077) HCl

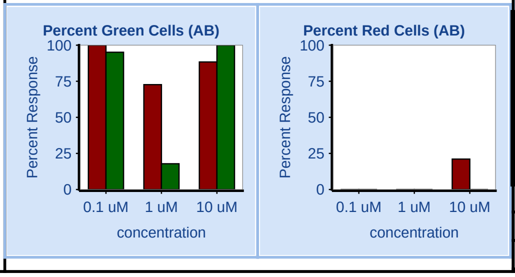

Average Response						
Dose	Green Signal	Actual Pct Green Cells	Normalized Pct Green Cells	Red Signal	Actual Pct Red Cells	Normalized Pct Red Cells
0.1 uM	102.67	0.91	108.29	101.81	0.08	-24.64
1 uM	96.48	0.82	45.26	87.94	0.05	-34.79
10 uM	118.94	0.90	116.10	112.87	0.11	-12.06

Compound | Normalized Values (A and B Sets) | Responses

Febuxostat

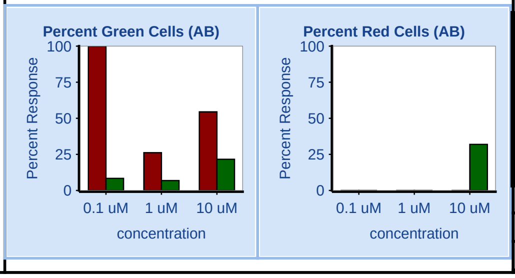

Average Response						
Dose	Green Signal	Actual Pct Green Cells	Normalized Pct Green Cells	Red Signal	Actual Pct Red Cells	Normalized Pct Red Cells
0.1 uM	94.64	0.83	60.32	80.43	0.03	-57.85
1 uM	94.70	0.80	16.53	99.10	0.05	-32.59
10 uM	92.69	0.79	38.05	125.78	0.14	9.83

Compound | Normalized Values (A and B Sets) | Responses

Fenoprofen Calcium

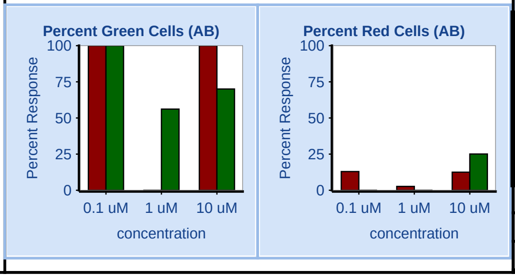

Average Response						
Dose	Green Signal	Actual Pct Green Cells	Normalized Pct Green Cells	Red Signal	Actual Pct Red Cells	Normalized Pct Red Cells
0.1 uM	115.56	0.94	137.65	117.04	0.14	0.90
1 uM	96.46	0.81	1.93	104.63	0.11	-5.17
10 uM	112.41	0.89	107.16	127.12	0.17	18.79

Compound	Normalized Values (A and B Sets)	Responses

Fenoprofen calcium hydrate

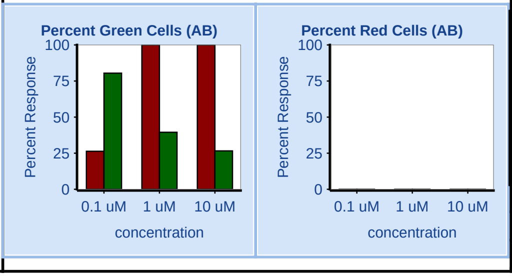

Average Response						
Dose	Green Signal	Actual Pct Green Cells	Normalized Pct Green Cells	Red Signal	Actual Pct Red Cells	Normalized Pct Red Cells
0.1 uM	95.03	0.88	53.40	80.04	0.05	-38.60
1 uM	95.22	0.83	76.95	83.19	0.03	-50.56
10 uM	96.01	0.81	82.48	66.71	0.01	-56.34

Compound	Normalized Values (A and B Sets)	Responses

Fenretinide

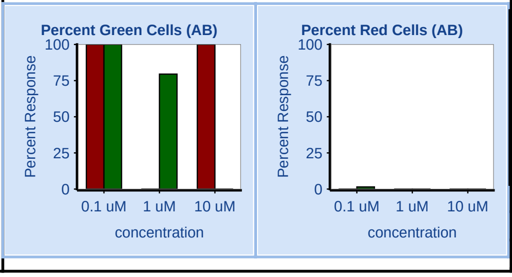

Average Response						
Dose	Green Signal	Actual Pct Green Cells	Normalized Pct Green Cells	Red Signal	Actual Pct Red Cells	Normalized Pct Red Cells
0.1 uM	104.35	0.94	143.42	103.24	0.10	-11.26
1 uM	96.15	0.88	1.93	89.56	0.05	-28.29
10 uM	96.46	0.85	42.26	96.52	0.10	-7.46

Compound	Normalized Values (A and B Sets)	Responses

Fenticonazole Nitrate

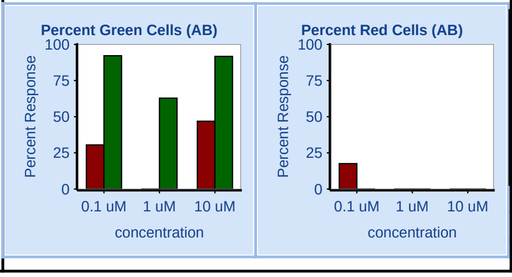

Average Response						
Dose	Green Signal	Actual Pct Green Cells	Normalized Pct Green Cells	Red Signal	Actual Pct Red Cells	Normalized Pct Red Cells
0.1 uM	98.51	0.87	61.34	108.58	0.13	-5.64
1 uM	91.57	0.76	-88.08	95.98	0.08	-21.38
10 uM	99.65	0.83	69.31	100.52	0.09	-13.13

Compound	Normalized Values (A and B Sets)	Responses

Ferrostatin-1 (Fer-1)

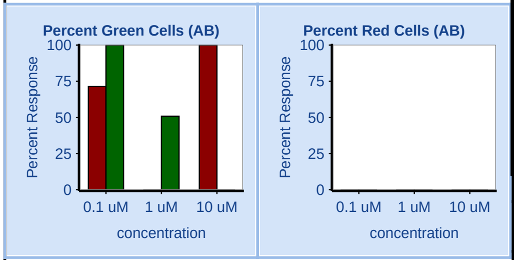

Average Response						
Dose	Green Signal	Actual Pct Green Cells	Normalized Pct Green Cells	Red Signal	Actual Pct Red Cells	Normalized Pct Red Cells
0.1 uM	97.38	0.90	107.09	68.02	0.01	-70.14
1 uM	83.22	0.75	-730.91	75.53	0.04	-28.12
10 uM	91.50	0.86	46.98	87.86	0.05	-27.65

Compound	Normalized Values (A and B Sets)	Responses

Fesoterodine Fumarate

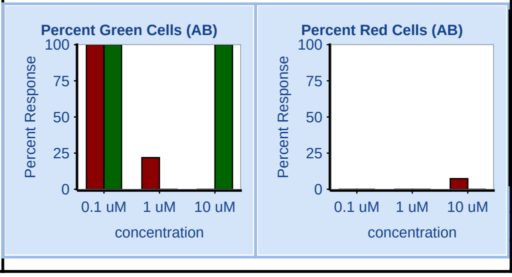

Average Response						
Dose	Green Signal	Actual Pct Green Cells	Normalized Pct Green Cells	Red Signal	Actual Pct Red Cells	Normalized Pct Red Cells
0.1 uM	102.67	0.92	126.35	68.35	0.02	-46.96
1 uM	87.41	0.71	3.42	74.83	0.03	-41.47
10 uM	98.18	0.87	-95.50	101.54	0.11	-5.62

Compound | Normalized Values (A and B Sets) | Responses

FG-4592

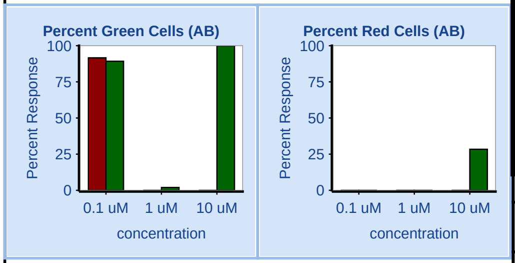

Average Response						
Dose	Green Signal	Actual Pct Green Cells	Normalized Pct Green Cells	Red Signal	Actual Pct Red Cells	Normalized Pct Red Cells
0.1 uM	96.15	0.88	90.45	88.13	0.06	-39.10
1 uM	86.49	0.79	-288.75	86.05	0.07	-15.23
10 uM	94.24	0.86	42.42	87.97	0.11	-3.71

Compound | Normalized Values (A and B Sets) | Responses

Fidaxomicin

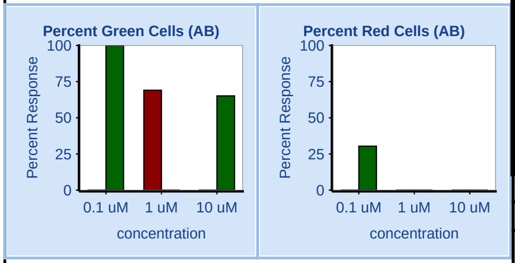

Average Response						
Dose	Green Signal	Actual Pct Green Cells	Normalized Pct Green Cells	Red Signal	Actual Pct Red Cells	Normalized Pct Red Cells
0.1 uM	96.71	0.89	43.87	104.52	0.13	0.64
1 uM	83.72	0.68	-38.30	74.64	0.03	-54.05
10 uM	82.86	0.64	-226.68	80.26	0.04	-40.47

Compound | Normalized Values (A and B Sets) | Responses

Flavopiridol HCl

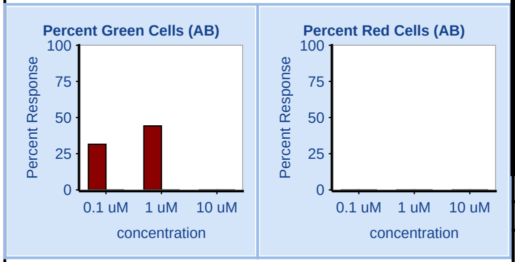

Average Response						
Dose	Green Signal	Actual Pct Green Cells	Normalized Pct Green Cells	Red Signal	Actual Pct Red Cells	Normalized Pct Red Cells
0.1 uM	87.14	0.80	5.20	88.14	0.06	-24.46
1 uM	93.37	0.70	-11.37	80.11	0.04	-36.17
10 uM	86.53	0.72	-853.25	88.33	0.09	-13.98

Compound | Normalized Values (A and B Sets) | Responses

Flubendazole

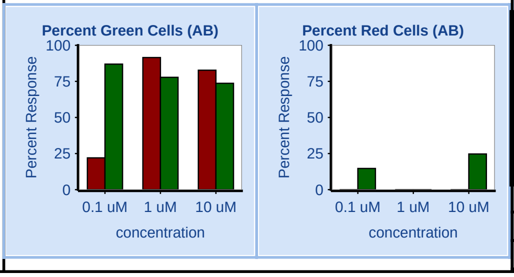

Average Response						
Dose	Green Signal	Actual Pct Green Cells	Normalized Pct Green Cells	Red Signal	Actual Pct Red Cells	Normalized Pct Red Cells
0.1 uM	97.42	0.86	54.49	101.15	0.09	-17.33
1 uM	107.06	0.88	84.67	96.72	0.08	-20.19
10 uM	105.89	0.84	78.21	112.14	0.11	-0.53

Compound | Normalized Values (A and B Sets) | Responses

Flupirtine maleate

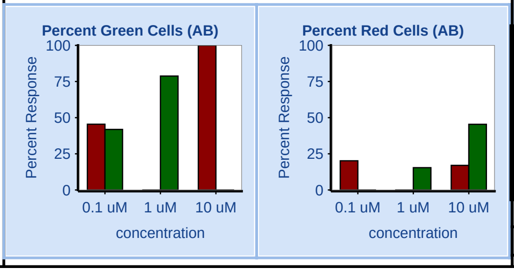

Average Response						
Dose	Green Signal	Actual Pct Green Cells	Normalized Pct Green Cells	Red Signal	Actual Pct Red Cells	Normalized Pct Red Cells
0.1 uM	90.94	0.83	43.68	106.32	0.13	-5.65
1 uM	90.19	0.77	-82.65	108.30	0.12	-1.54

Compound	Normalized Values (A and B Sets)	Responses						
		10 uM	121.33	0.84	61.40	129.87	0.19	31.22

Compound	Normalized Values (A and B Sets)	Responses

Flurbiprofen

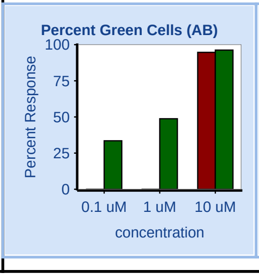

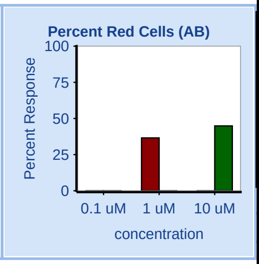

Average Response

Dose	Green Signal	Actual Pct Green Cells	Normalized Pct Green Cells	Red Signal	Actual Pct Red Cells	Normalized Pct Red Cells
0.1 uM	84.76	0.78	-25.15	110.15	0.12	-8.18
1 uM	93.49	0.75	-96.73	112.65	0.13	3.09
10 uM	114.36	0.87	95.46	122.04	0.16	18.15

Compound	Normalized Values (A and B Sets)	Responses

Fluvastatin Sodium

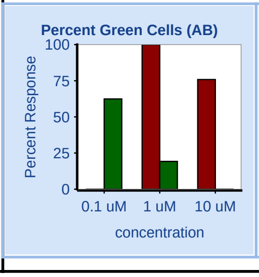

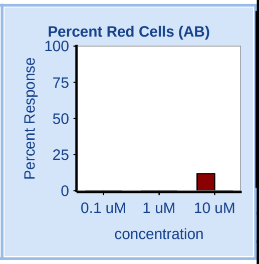

Average Response

Dose	Green Signal	Actual Pct Green Cells	Normalized Pct Green Cells	Red Signal	Actual Pct Red Cells	Normalized Pct Red Cells
0.1 uM	90.59	0.82	13.31	101.51	0.07	-36.55
1 uM	101.89	0.84	66.20	95.85	0.07	-26.53
10 uM	101.46	0.78	34.18	114.27	0.14	3.34

Compound	Normalized Values (A and B Sets)	Responses

Foretinib (GSK1363089)

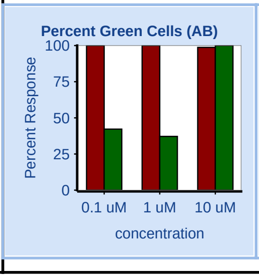

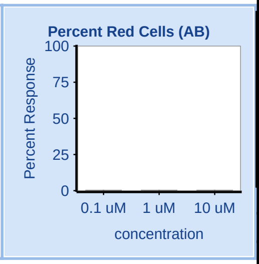

Average Response

Dose	Green Signal	Actual Pct Green Cells	Normalized Pct Green Cells	Red Signal	Actual Pct Red Cells	Normalized Pct Red Cells
0.1 uM	90.90	0.86	82.03	88.30	0.04	-50.16
1 uM	94.40	0.86	83.25	75.40	0.01	-50.87
10 uM	100.19	0.88	103.43	101.50	0.06	-28.35

Compound	Normalized Values (A and B Sets)	Responses

Forskolin

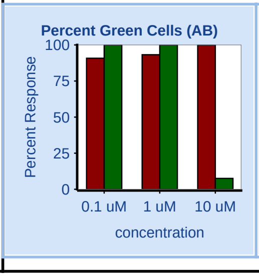

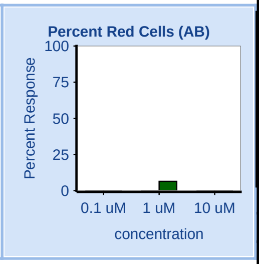

Average Response

Dose	Green Signal	Actual Pct Green Cells	Normalized Pct Green Cells	Red Signal	Actual Pct Red Cells	Normalized Pct Red Cells
0.1 uM	97.90	0.89	98.61	87.43	0.05	-31.00
1 uM	105.00	0.90	107.16	98.69	0.09	-13.86
10 uM	95.35	0.90	255.59	78.12	0.03	-45.92

Compound	Normalized Values (A and B Sets)	Responses

Fosaprepitant dimeglumine salt

Average Response

Dose	Green Signal	Actual Pct Green Cells	Normalized Pct Green Cells	Red Signal	Actual Pct Red Cells	Normalized Pct Red Cells

Compound	Normalized Values (A and B Sets)	Responses

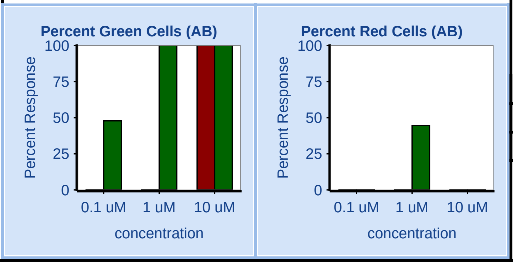

0.1 uM	93.01	0.85	13.65	79.45	0.04	-41.33
1 uM	96.17	0.78	47.55	87.02	0.11	-12.13
10 uM	110.24	0.91	128.50	95.83	0.08	-19.84

Compound	Normalized Values (A and B Sets)	Responses

Fosfomycin Tromethamine

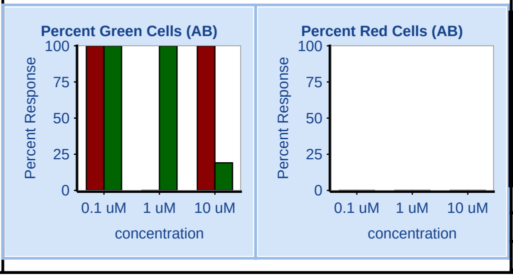

Average Response						
Dose	Green Signal	Actual Pct Green Cells	Normalized Pct Green Cells	Red Signal	Actual Pct Red Cells	Normalized Pct Red Cells
0.1 uM	111.14	0.93	162.20	93.93	0.09	-17.67
1 uM	90.62	0.70	-7.95	88.77	0.07	-31.98
10 uM	98.02	0.81	86.09	98.97	0.10	-9.08

Compound	Normalized Values (A and B Sets)	Responses

Fostamatinib (R788)

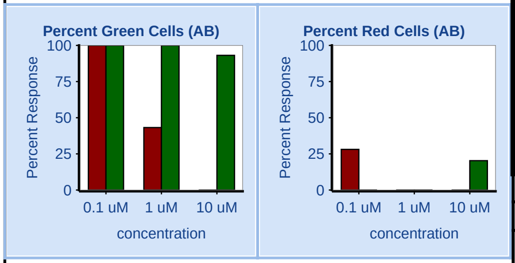

Average Response						
Dose	Green Signal	Actual Pct Green Cells	Normalized Pct Green Cells	Red Signal	Actual Pct Red Cells	Normalized Pct Red Cells
0.1 uM	106.32	0.92	123.23	94.62	0.12	-2.81
1 uM	99.83	0.85	86.05	69.64	0.02	-48.11
10 uM	107.02	0.80	-687.63	96.27	0.11	-4.08

Compound	Normalized Values (A and B Sets)	Responses

FR 180204

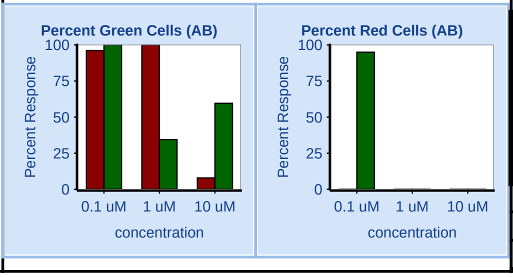

Average Response						
Dose	Green Signal	Actual Pct Green Cells	Normalized Pct Green Cells	Red Signal	Actual Pct Red Cells	Normalized Pct Red Cells
0.1 uM	98.59	0.92	126.18	108.31	0.16	23.85
1 uM	96.85	0.88	168.19	79.87	0.05	-20.14
10 uM	85.06	0.85	33.74	91.33	0.08	-14.41

Compound	Normalized Values (A and B Sets)	Responses

Gadodiamide

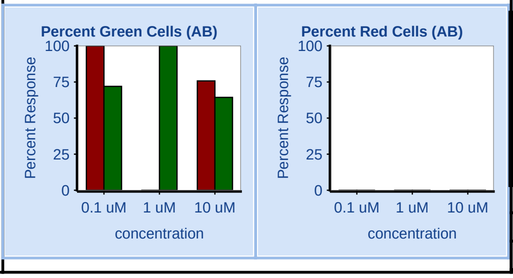

Average Response						
Dose	Green Signal	Actual Pct Green Cells	Normalized Pct Green Cells	Red Signal	Actual Pct Red Cells	Normalized Pct Red Cells
0.1 uM	102.82	0.90	106.66	95.74	0.07	-37.09
1 uM	105.93	0.87	27.93	87.38	0.05	-34.32
10 uM	97.42	0.83	70.04	100.04	0.07	-23.48

Compound	Normalized Values (A and B Sets)	Responses

Galeterone

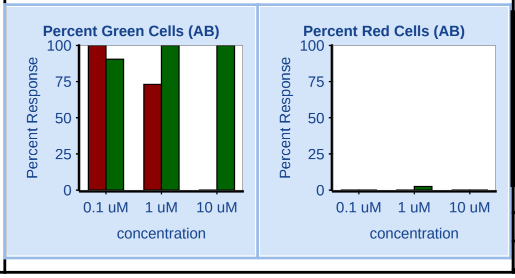

Average Response

Dose	Green Signal	Actual Pct Green Cells	Normalized Pct Green Cells	Red Signal	Actual Pct Red Cells	Normalized Pct Red Cells
0.1 uM	105.50	0.92	126.00	92.12	0.08	-17.03
1 uM	105.14	0.88	96.95	95.37	0.09	-17.79
10 uM	102.82	0.91	-17.09	86.11	0.05	-35.37

Compound	Normalized Values (A and B Sets)	Responses

Ganetespib (STA-9090)

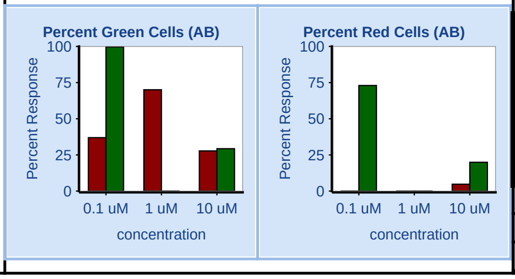

Average Response

Dose	Green Signal	Actual Pct Green Cells	Normalized Pct Green Cells	Red Signal	Actual Pct Red Cells	Normalized Pct Red Cells
0.1 uM	117.27	0.88	68.31	113.45	0.16	21.38
1 uM	96.42	0.80	32.47	103.18	0.10	-9.64
10 uM	99.62	0.77	28.53	123.76	0.15	12.33

Compound	Normalized Values (A and B Sets)	Responses

GANT 61

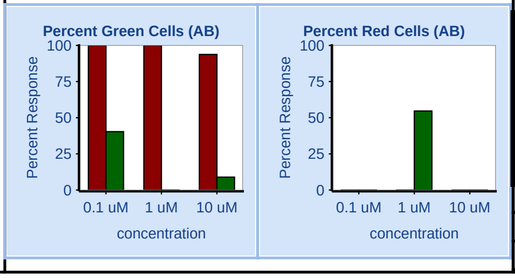

Average Response

Dose	Green Signal	Actual Pct Green Cells	Normalized Pct Green Cells	Red Signal	Actual Pct Red Cells	Normalized Pct Red Cells
0.1 uM	90.45	0.85	71.43	74.18	0.02	-62.95
1 uM	92.53	0.81	79.39	92.47	0.10	13.08
10 uM	89.18	0.86	51.34	69.77	0.04	-34.71

Compound	Normalized Values (A and B Sets)	Responses

Gastrodin

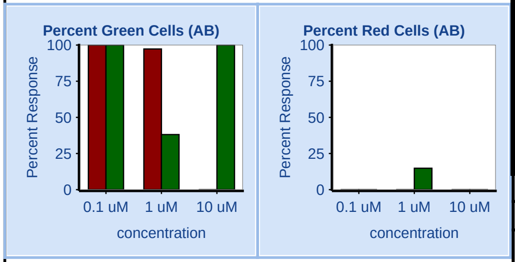

Average Response

Dose	Green Signal	Actual Pct Green Cells	Normalized Pct Green Cells	Red Signal	Actual Pct Red Cells	Normalized Pct Red Cells
0.1 uM	98.93	0.92	121.13	82.34	0.05	-34.04
1 uM	106.10	0.84	67.70	86.78	0.08	-18.57
10 uM	94.17	0.88	-136.39	76.76	0.03	-44.48

Compound	Normalized Values (A and B Sets)	Responses

GDC-0068

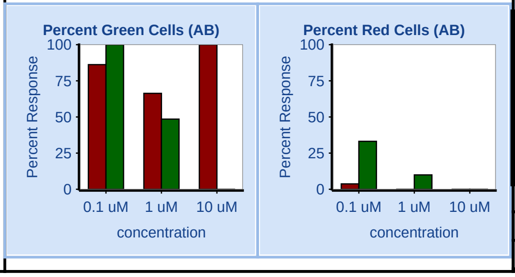

Average Response

Dose	Green Signal	Actual Pct Green Cells	Normalized Pct Green Cells	Red Signal	Actual Pct Red Cells	Normalized Pct Red Cells
0.1 uM	105.04	0.93	146.55	113.05	0.15	18.44
1 uM	92.63	0.81	57.43	95.85	0.09	-16.95
10 uM	95.02	0.90	341.81	75.40	0.03	-45.23

Compound	Normalized Values (A and B Sets)	Responses

GDC-0152

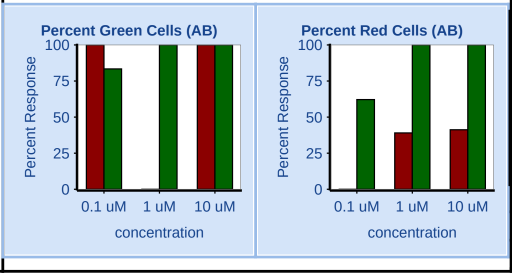

Average Response

Dose	Green Signal	Actual Pct Green Cells	Normalized Pct Green Cells	Red Signal	Actual Pct Red Cells	Normalized Pct Red Cells
0.1 uM	113.31	0.90	109.64	101.80	0.14	8.50
1 uM	123.08	0.91	-75.22	120.25	0.24	71.49
10 uM	127.99	0.95	126.57	130.87	0.33	93.99

Compound	Normalized Values (A and B Sets)	Responses

GDC-0349

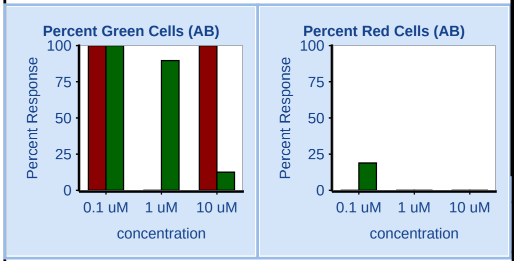

Average Response

Dose	Green Signal	Actual Pct Green Cells	Normalized Pct Green Cells	Red Signal	Actual Pct Red Cells	Normalized Pct Red Cells
0.1 uM	101.84	0.91	123.20	88.31	0.08	-27.48
1 uM	90.00	0.86	-140.49	62.23	0.01	-44.78
10 uM	88.18	0.90	84.44	92.42	0.08	-15.82

Compound	Normalized Values (A and B Sets)	Responses

GDC-0879

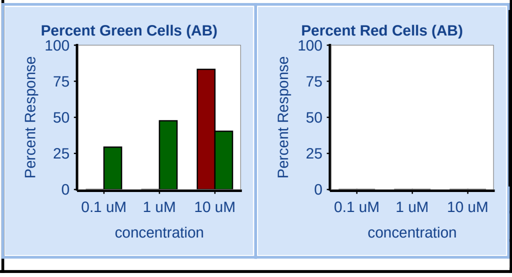

Average Response

Dose	Green Signal	Actual Pct Green Cells	Normalized Pct Green Cells	Red Signal	Actual Pct Red Cells	Normalized Pct Red Cells
0.1 uM	86.34	0.79	-2.73	72.69	0.02	-63.48
1 uM	91.57	0.81	4.22	67.01	0.01	-50.78
10 uM	87.56	0.82	61.81	86.95	0.02	-46.25

Compound	Normalized Values (A and B Sets)	Responses

GDC-0980 (RG7422)

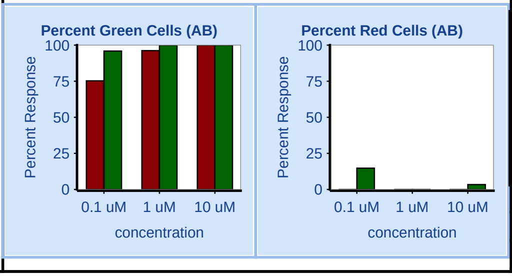

Average Response

Dose	Green Signal	Actual Pct Green Cells	Normalized Pct Green Cells	Red Signal	Actual Pct Red Cells	Normalized Pct Red Cells
0.1 uM	94.02	0.88	85.60	102.54	0.12	3.50
1 uM	104.21	0.91	112.14	90.28	0.06	-28.55
10 uM	114.50	0.98	474.69	110.29	0.12	-2.21

Compound	Normalized Values (A and B Sets)	Responses

Gefitinib (ZD1839)

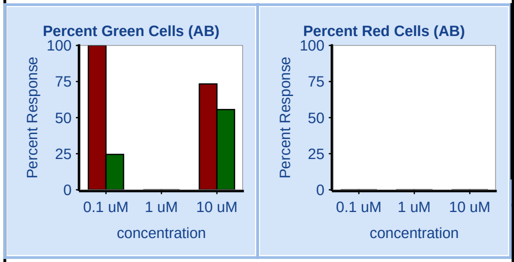

Average Response

Dose	Green Signal	Actual Pct Green Cells	Normalized Pct Green Cells	Red Signal	Actual Pct Red Cells	Normalized Pct Red Cells
0.1 uM	91.28	0.84	62.35	67.41	0.01	-67.89
1 uM	53.94	0.30	-2445.50	62.22	0.01	-44.27

Compound	Normalized Values (A and B Sets)	Responses						
		10 uM	85.54	0.88	64.46	64.85	0.01	-47.25

Compound	Normalized Values (A and B Sets)	Responses

Geldanamycin

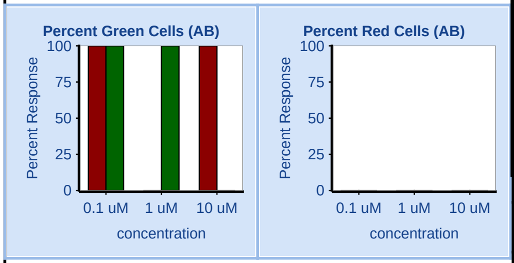

Average Response						
Dose	Green Signal	Actual Pct Green Cells	Normalized Pct Green Cells	Red Signal	Actual Pct Red Cells	Normalized Pct Red Cells
0.1 uM	104.51	0.92	130.69	91.81	0.05	-42.25
1 uM	93.25	0.86	-267.19	83.62	0.04	-26.98
10 uM	97.09	0.84	34.38	78.55	0.07	-19.41

Compound	Normalized Values (A and B Sets)	Responses

Genipin

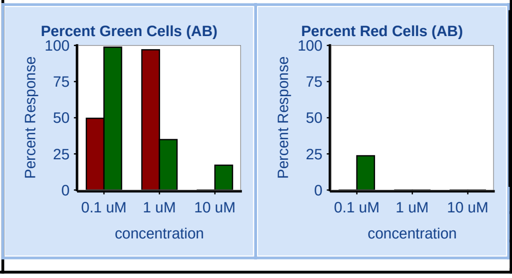

Average Response						
Dose	Green Signal	Actual Pct Green Cells	Normalized Pct Green Cells	Red Signal	Actual Pct Red Cells	Normalized Pct Red Cells
0.1 uM	92.33	0.87	74.21	105.15	0.12	4.43
1 uM	94.11	0.84	65.95	92.63	0.06	-29.28
10 uM	91.34	0.86	-17.11	0.14	0.00	-58.71

Compound	Normalized Values (A and B Sets)	Responses

Geniposide

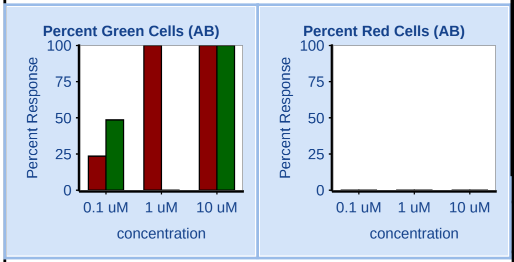

Average Response						
Dose	Green Signal	Actual Pct Green Cells	Normalized Pct Green Cells	Red Signal	Actual Pct Red Cells	Normalized Pct Red Cells
0.1 uM	89.37	0.83	36.07	81.30	0.05	-32.87
1 uM	75.06	0.55	-115.80	62.99	0.01	-50.19
10 uM	105.67	0.92	212.14	92.78	0.07	-27.39

Compound	Normalized Values (A and B Sets)	Responses

Geniposidic acid

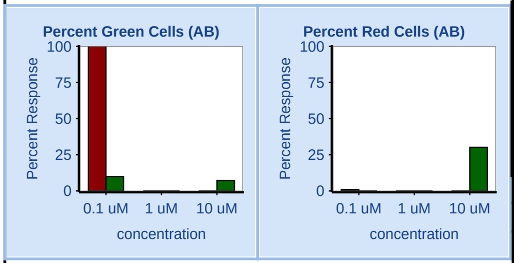

Average Response						
Dose	Green Signal	Actual Pct Green Cells	Normalized Pct Green Cells	Red Signal	Actual Pct Red Cells	Normalized Pct Red Cells
0.1 uM	96.17	0.88	81.80	94.39	0.09	-14.58
1 uM	78.01	0.61	-46.46	77.98	0.04	-37.75
10 uM	88.43	0.84	-187.67	109.25	0.13	5.01

Compound	Normalized Values (A and B Sets)	Responses

Gestodene

Average Response						
Dose	Green Signal			Red Signal	Actual Pct Red Cells	

Compound	Normalized Values (A and B Sets)	Responses

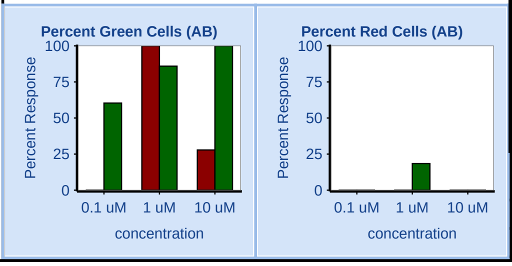

		Actual Pct Green Cells	Normalized Pct Green Cells			Normalized Pct Red Cells
0.1 uM	85.54	0.73	-106.04	92.43	0.06	-35.14
1 uM	109.12	0.91	126.58	107.31	0.11	-5.56
10 uM	104.50	0.84	82.40	97.18	0.07	-24.19

Compound	Normalized Values (A and B Sets)	Responses

Gimeracil

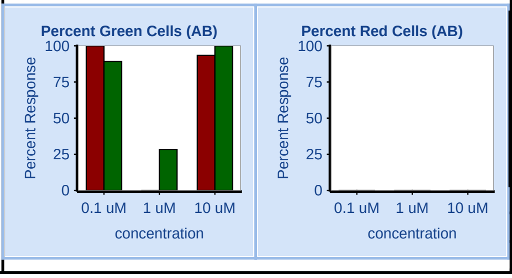

Average Response

Dose	Green Signal	Actual Pct Green Cells	Normalized Pct Green Cells	Red Signal	Actual Pct Red Cells	Normalized Pct Red Cells
0.1 uM	109.59	0.92	126.13	80.04	0.03	-56.36
1 uM	91.59	0.81	13.64	88.80	0.05	-35.69
10 uM	109.58	0.88	103.34	102.76	0.07	-23.41

Compound	Normalized Values (A and B Sets)	Responses

Ginkgolide A

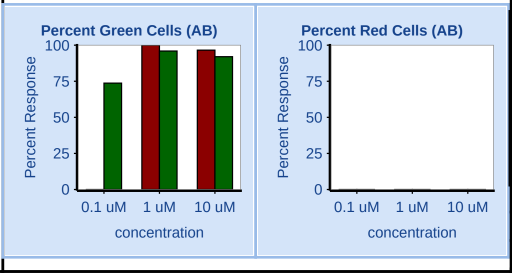

Average Response

Dose	Green Signal	Actual Pct Green Cells	Normalized Pct Green Cells	Red Signal	Actual Pct Red Cells	Normalized Pct Red Cells
0.1 uM	95.21	0.84	28.08	88.79	0.06	-42.47
1 uM	110.70	0.91	120.20	93.35	0.06	-27.80
10 uM	104.53	0.87	94.29	100.25	0.06	-26.76

Compound	Normalized Values (A and B Sets)	Responses

Ginkgolide B

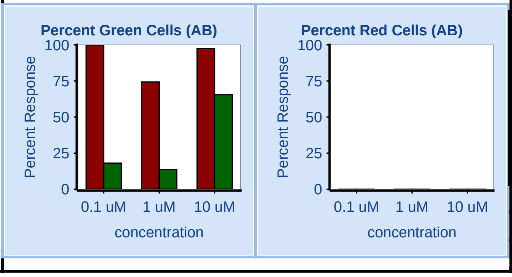

Average Response

Dose	Green Signal	Actual Pct Green Cells	Normalized Pct Green Cells	Red Signal	Actual Pct Red Cells	Normalized Pct Red Cells
0.1 uM	91.42	0.85	77.61	74.74	0.01	-66.44
1 uM	98.28	0.82	43.98	98.98	0.08	-19.55
10 uM	98.27	0.85	81.46	88.49	0.02	-43.41

Compound	Normalized Values (A and B Sets)	Responses

Givinostat (ITF2357)

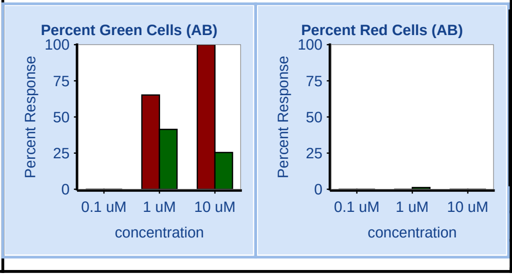

Average Response

Dose	Green Signal	Actual Pct Green Cells	Normalized Pct Green Cells	Red Signal	Actual Pct Red Cells	Normalized Pct Red Cells
0.1 uM	79.07	0.75	-53.97	74.57	0.03	-40.31
1 uM	104.77	0.81	53.30	99.88	0.10	-12.82
10 uM	110.37	0.90	269.26	90.83	0.05	-36.31

Compound | Normalized Values (A and B Sets) | Responses

Glycyrrhizic Acid

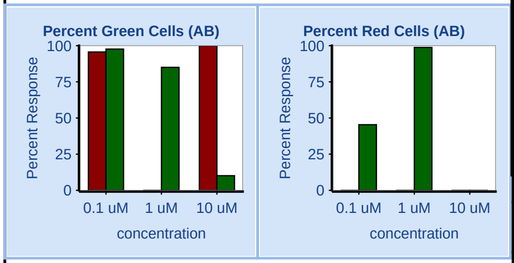

Average Response

Dose	Green Signal	Actual Pct Green Cells	Normalized Pct Green Cells	Red Signal	Actual Pct Red Cells	Normalized Pct Red Cells
0.1 uM	99.81	0.89	96.66	96.51	0.11	-7.46
1 uM	89.21	0.75	-819.22	98.43	0.14	36.82
10 uM	91.37	0.87	55.43	72.25	0.03	-35.90

Compound | Normalized Values (A and B Sets) | Responses

Golgicide A

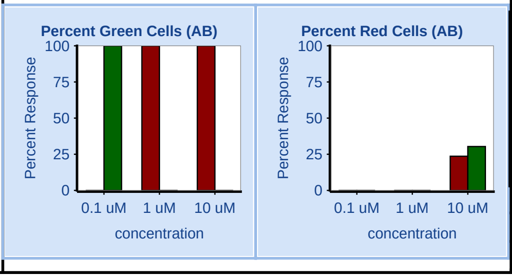

Average Response

Dose	Green Signal	Actual Pct Green Cells	Normalized Pct Green Cells	Red Signal	Actual Pct Red Cells	Normalized Pct Red Cells
0.1 uM	101.21	0.88	89.94	76.89	0.02	-62.33
1 uM	88.10	0.77	76.80	64.79	0.01	-41.51
10 uM	88.53	0.80	-3.85	120.25	0.18	27.00

Compound | Normalized Values (A and B Sets) | Responses

Golvatinib (E7050)

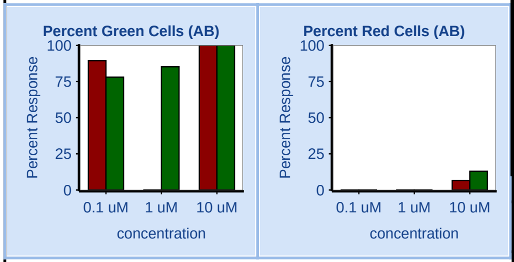

Average Response

Dose	Green Signal	Actual Pct Green Cells	Normalized Pct Green Cells	Red Signal	Actual Pct Red Cells	Normalized Pct Red Cells
0.1 uM	94.07	0.87	83.79	100.34	0.09	-19.35
1 uM	88.43	0.83	-319.92	72.36	0.02	-36.25
10 uM	125.03	0.97	150.00	105.40	0.14	9.83

Compound | Normalized Values (A and B Sets) | Responses

Gramine

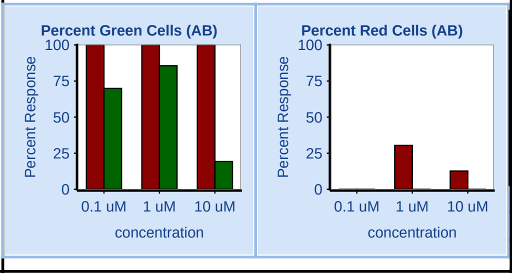

Average Response

Dose	Green Signal	Actual Pct Green Cells	Normalized Pct Green Cells	Red Signal	Actual Pct Red Cells	Normalized Pct Red Cells
0.1 uM	100.16	0.89	107.22	71.95	0.01	-70.78
1 uM	101.20	0.91	238.57	95.51	0.10	-11.74
10 uM	101.69	0.90	86.94	87.57	0.08	-15.52

Compound | Normalized Values (A and B Sets) | Responses

Granisetron HCl

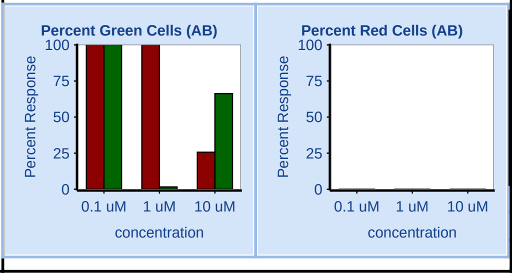

Average Response

Dose	Green Signal	Actual Pct Green Cells	Normalized Pct Green Cells	Red Signal	Actual Pct Red Cells	Normalized Pct Red Cells
0.1 uM	103.60	0.94	142.51	88.94	0.05	-44.93
1 uM	95.80	0.83	74.16	78.53	0.02	-47.11
10 uM	88.73	0.79	45.96	86.77	0.03	-43.08

Compound | Normalized Values (A and B Sets) | Responses

GSK 3787

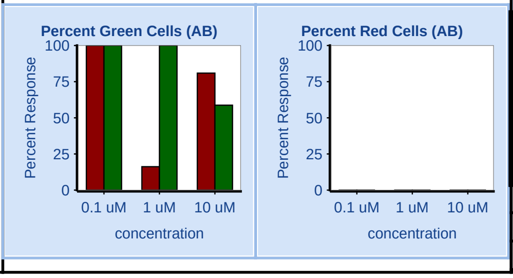

Average Response

Dose	Green Signal	Actual Pct Green Cells	Normalized Pct Green Cells	Red Signal	Actual Pct Red Cells	Normalized Pct Red Cells
0.1 uM	98.08	0.91	117.79	70.01	0.02	-64.81
1 uM	99.04	0.91	67.97	57.12	0.01	-44.50
10 uM	88.39	0.89	69.84	56.56	0.00	-49.50

Compound | Normalized Values (A and B Sets) | Responses

GSK 264220A

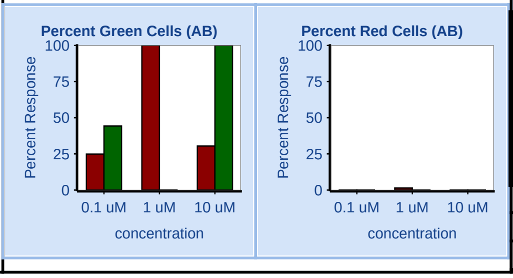

Average Response

Dose	Green Signal	Actual Pct Green Cells	Normalized Pct Green Cells	Red Signal	Actual Pct Red Cells	Normalized Pct Red Cells
0.1 uM	88.58	0.82	34.61	87.87	0.05	-46.05
1 uM	93.42	0.81	71.35	82.04	0.05	-29.99
10 uM	92.56	0.89	75.31	87.89	0.06	-24.53

Compound | Normalized Values (A and B Sets) | Responses

GSK 269962

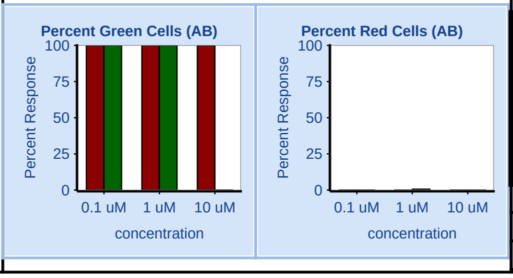

Average Response

Dose	Green Signal	Actual Pct Green Cells	Normalized Pct Green Cells	Red Signal	Actual Pct Red Cells	Normalized Pct Red Cells
0.1 uM	110.02	0.92	125.35	88.93	0.07	-30.51
1 uM	113.96	0.96	331.22	87.25	0.06	-11.26
10 uM	92.39	0.80	-2.57	79.13	0.04	-35.12

Compound | Normalized Values (A and B Sets) | Responses

GSK 650394

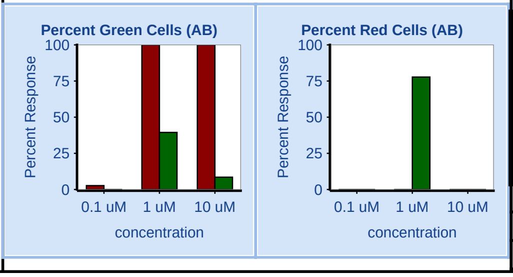

Average Response

Dose	Green Signal	Actual Pct Green Cells	Normalized Pct Green Cells	Red Signal	Actual Pct Red Cells	Normalized Pct Red Cells
0.1 uM	85.02	0.78	-0.33	70.81	0.01	-66.88
1 uM	97.33	0.88	126.41	103.42	0.14	30.16
10 uM	86.29	0.87	61.95	71.60	0.03	-39.36

Compound | Normalized Values (A and B Sets) | Responses

GSK J4 HCl

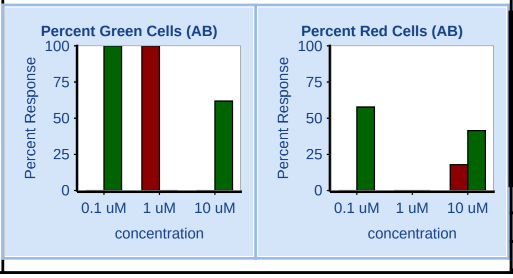

Average Response

Dose	Green Signal	Actual Pct Green Cells	Normalized Pct Green Cells	Red Signal	Actual Pct Red Cells	Normalized Pct Red Cells
0.1 uM	102.40	0.90	11.29	96.56	0.14	6.84
1 uM	82.46	0.64	-71.53	85.53	0.05	-43.37
10 uM	104.17	0.76	-38.18	109.20	0.17	29.53

Compound | Normalized Values (A and B Sets) | Responses

GSK256066

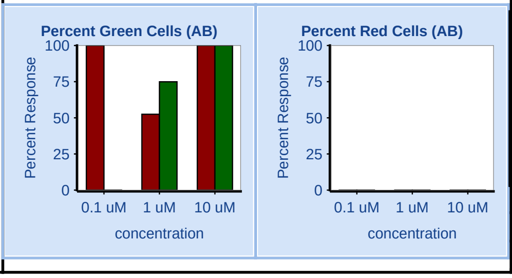

Average Response

Dose	Green Signal	Actual Pct Green Cells	Normalized Pct Green Cells	Red Signal	Actual Pct Red Cells	Normalized Pct Red Cells
0.1 uM	91.89	0.85	45.16	79.88	0.05	-32.67
1 uM	91.44	0.82	63.70	87.35	0.06	-28.19
10 uM	96.46	0.94	420.67	82.35	0.03	-45.55

Compound | Normalized Values (A and B Sets) | Responses

GSK429286A

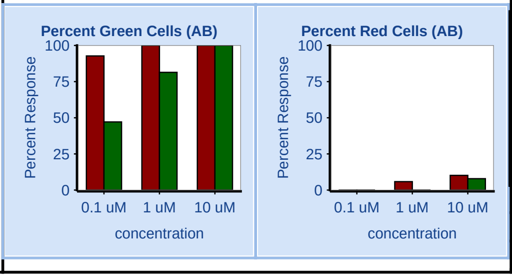

Average Response

Dose	Green Signal	Actual Pct Green Cells	Normalized Pct Green Cells	Red Signal	Actual Pct Red Cells	Normalized Pct Red Cells
0.1 uM	96.45	0.86	69.95	89.91	0.06	-35.16
1 uM	114.99	0.90	114.93	107.45	0.11	-6.66
10 uM	127.96	0.91	126.00	117.07	0.15	9.03

Compound | Normalized Values (A and B Sets) | Responses

GSK461364

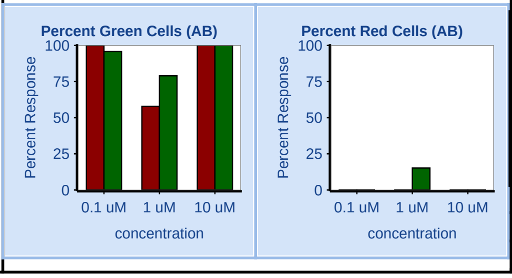

Average Response

Dose	Green Signal	Actual Pct Green Cells	Normalized Pct Green Cells	Red Signal	Actual Pct Red Cells	Normalized Pct Red Cells
0.1 uM	105.77	0.89	97.85	89.00	0.07	-21.09
1 uM	99.65	0.83	68.47	109.72	0.14	6.68
10 uM	110.97	0.96	413.70	75.44	0.04	-38.36

Compound | Normalized Values (A and B Sets) | Responses

GSK690693

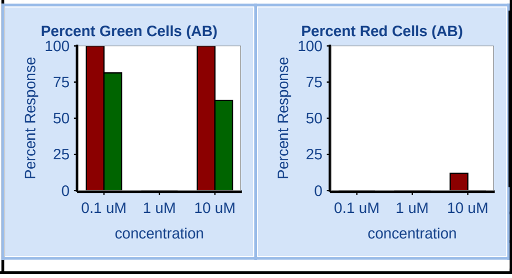

Average Response

Dose	Green Signal	Actual Pct Green Cells	Normalized Pct Green Cells	Red Signal	Actual Pct Red Cells	Normalized Pct Red Cells
0.1 uM	102.20	0.92	127.65	87.31	0.04	-54.03
1 uM	85.86	0.72	-51.75	78.63	0.03	-43.49
10 uM	98.52	0.86	87.21	110.91	0.09	-16.84

Compound | Normalized Values (A and B Sets) | Responses

GSK837149A

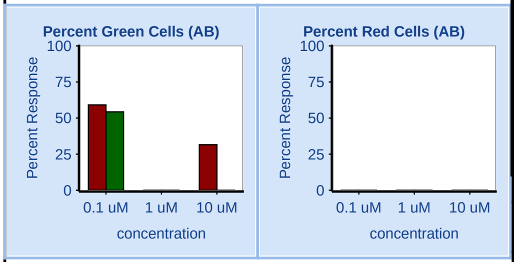

Average Response

Dose	Green Signal	Actual Pct Green Cells	Normalized Pct Green Cells	Red Signal	Actual Pct Red Cells	Normalized Pct Red Cells
0.1 uM	91.22	0.84	56.74	83.24	0.03	-57.02
1 uM	84.92	0.77	-308.85	68.20	0.01	-42.32
10 uM	84.10	0.79	-15.13	77.28	0.04	-33.61

Compound | Normalized Values (A and B Sets) | Responses

GSK923295

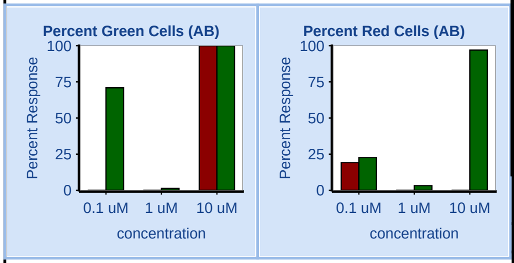

Average Response

Dose	Green Signal	Actual Pct Green Cells	Normalized Pct Green Cells	Red Signal	Actual Pct Red Cells	Normalized Pct Red Cells
0.1 uM	94.82	0.73	-563.22	103.70	0.17	20.84
1 uM	94.46	0.66	-40.40	88.92	0.12	-3.96
10 uM	116.06	0.90	123.18	108.91	0.19	34.34

Compound | Normalized Values (A and B Sets) | Responses

GSK1059615

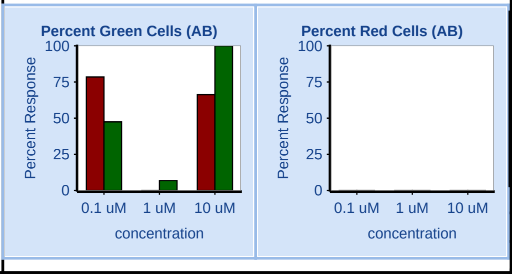

Average Response

Dose	Green Signal	Actual Pct Green Cells	Normalized Pct Green Cells	Red Signal	Actual Pct Red Cells	Normalized Pct Red Cells
0.1 uM	93.56	0.85	62.93	104.29	0.09	-25.15
1 uM	89.10	0.79	-4.70	75.55	0.02	-47.70
10 uM	94.55	0.86	88.05	97.24	0.03	-40.66

Compound | Normalized Values (A and B Sets) | Responses

GSK1070916

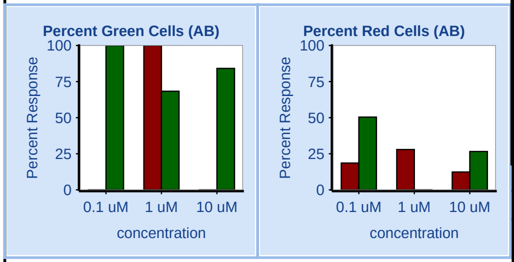

Average Response

Dose	Green Signal	Actual Pct Green Cells	Normalized Pct Green Cells	Red Signal	Actual Pct Red Cells	Normalized Pct Red Cells
0.1 uM	98.21	0.83	41.32	114.32	0.19	34.50
1 uM	104.72	0.90	98.50	102.32	0.14	5.05
10 uM	95.03	0.70	-1419.71	113.83	0.16	19.48

Compound | Normalized Values (A and B Sets) | Responses

GSK1292263

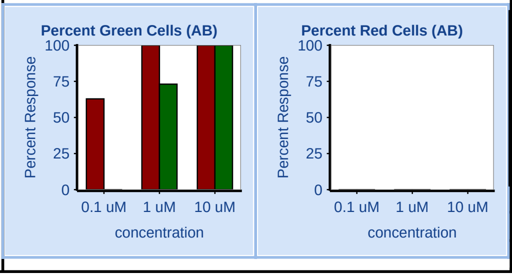

Average Response

Dose	Green Signal	Actual Pct Green Cells	Normalized Pct Green Cells	Red Signal	Actual Pct Red Cells	Normalized Pct Red Cells
0.1 uM	77.34	0.72	-95.44	75.87	0.04	-36.76
1 uM	98.70	0.87	87.27	73.58	0.02	-48.50
10 uM	108.01	0.96	485.28	71.11	0.02	-50.76

Compound | Normalized Values (A and B Sets) | Responses

GSK1838705A

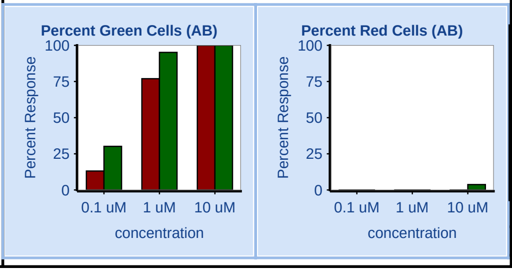

Average Response

Dose	Green Signal	Actual Pct Green Cells	Normalized Pct Green Cells	Red Signal	Actual Pct Red Cells	Normalized Pct Red Cells
0.1 uM	93.77	0.82	21.64	84.00	0.07	-23.06
1 uM	101.65	0.86	86.06	94.30	0.08	-17.97

Compound	Normalized Values (A and B Sets)	Responses						
		10 uM	97.44	0.96	492.01	100.92	0.06	-29.11

Compound	Normalized Values (A and B Sets)	Responses

GSK2126458 (GSK458)

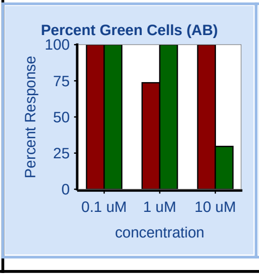

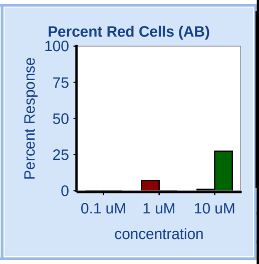

Average Response						
Dose	Green Signal	Actual Pct Green Cells	Normalized Pct Green Cells	Red Signal	Actual Pct Red Cells	Normalized Pct Red Cells
0.1 uM	94.37	0.93	131.79	76.54	0.04	-37.72
1 uM	112.54	0.90	105.93	103.60	0.13	1.67
10 uM	107.95	0.89	145.69	110.41	0.15	14.19

Compound	Normalized Values (A and B Sets)	Responses

GSK2334470

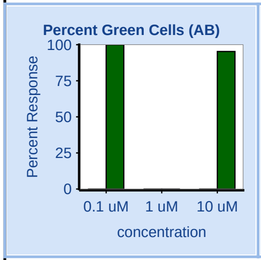

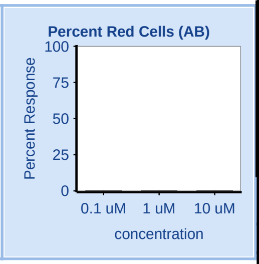

Average Response						
Dose	Green Signal	Actual Pct Green Cells	Normalized Pct Green Cells	Red Signal	Actual Pct Red Cells	Normalized Pct Red Cells
0.1 uM	93.18	0.84	-275.85	95.36	0.09	-15.80
1 uM	82.20	0.70	-15.10	91.71	0.08	-25.43
10 uM	92.50	0.80	-9.58	98.99	0.09	-13.09

Compound	Normalized Values (A and B Sets)	Responses

GSK2636771

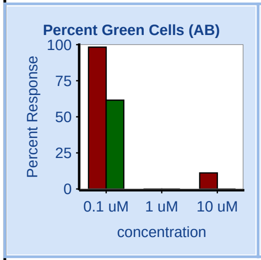

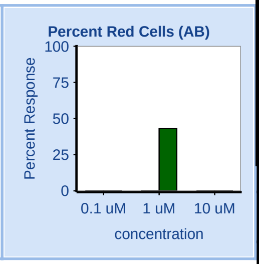

Average Response						
Dose	Green Signal	Actual Pct Green Cells	Normalized Pct Green Cells	Red Signal	Actual Pct Red Cells	Normalized Pct Red Cells
0.1 uM	91.61	0.87	79.91	78.55	0.02	-63.92
1 uM	85.60	0.78	-228.55	84.65	0.09	7.51
10 uM	75.80	0.68	-118.47	62.81	0.02	-42.68

Compound	Normalized Values (A and B Sets)	Responses

Guanidine HCl

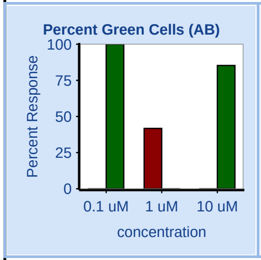

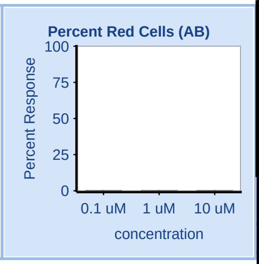

Average Response						
Dose	Green Signal	Actual Pct Green Cells	Normalized Pct Green Cells	Red Signal	Actual Pct Red Cells	Normalized Pct Red Cells
0.1 uM	87.59	0.84	-225.68	65.06	0.01	-59.17
1 uM	83.24	0.68	-30.08	86.09	0.04	-44.74
10 uM	90.15	0.80	0.55	61.42	0.01	-58.41

Compound	Normalized Values (A and B Sets)	Responses

Guanosine

Average Response						
Dose				Red Signal		

Compound	Normalized Values (A and B Sets)	Responses

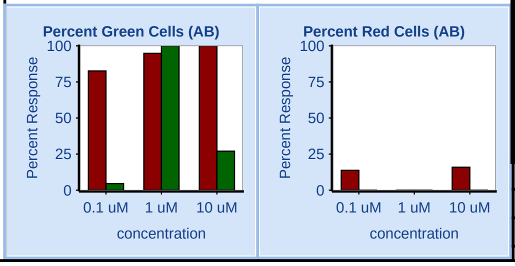

	Green Signal	Actual Pct Green Cells	Normalized Pct Green Cells		Actual Pct Red Cells	Normalized Pct Red Cells
0.1 uM	89.58	0.84	43.60	86.52	0.08	-21.62
1 uM	106.39	0.90	102.80	87.54	0.04	-37.23
10 uM	99.40	0.88	67.62	95.35	0.09	-14.34

Compound	Normalized Values (A and B Sets)	Responses

GW 9508

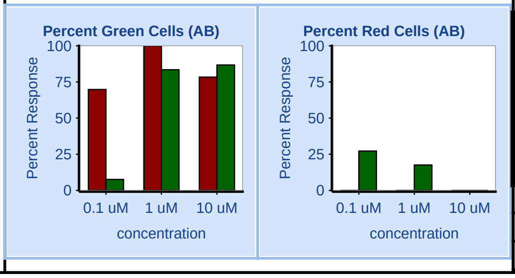

Average Response

Dose	Green Signal	Actual Pct Green Cells	Normalized Pct Green Cells	Red Signal	Actual Pct Red Cells	Normalized Pct Red Cells
0.1 uM	88.05	0.82	38.68	98.02	0.10	-12.92
1 uM	102.09	0.92	255.87	82.17	0.07	-5.25
10 uM	92.92	0.90	82.56	86.31	0.04	-31.82

Compound	Normalized Values (A and B Sets)	Responses

GW0742

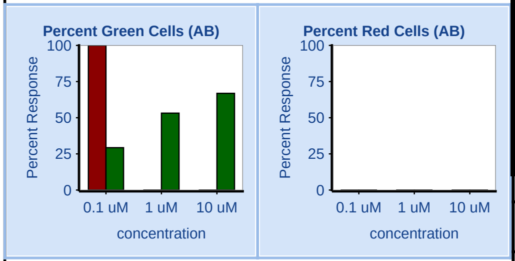

Average Response

Dose	Green Signal	Actual Pct Green Cells	Normalized Pct Green Cells	Red Signal	Actual Pct Red Cells	Normalized Pct Red Cells
0.1 uM	96.04	0.88	91.82	83.69	0.04	-51.99
1 uM	91.47	0.84	-179.27	73.43	0.02	-39.31
10 uM	85.83	0.81	-6.45	78.98	0.04	-34.01

Compound	Normalized Values (A and B Sets)	Responses

GW3965 HCl

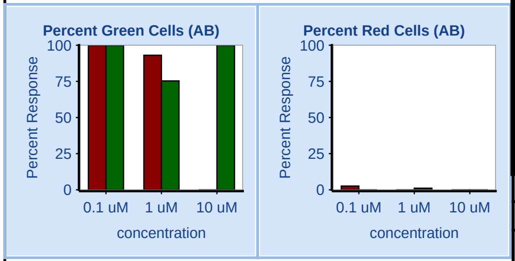

Average Response

Dose	Green Signal	Actual Pct Green Cells	Normalized Pct Green Cells	Red Signal	Actual Pct Red Cells	Normalized Pct Red Cells
0.1 uM	96.34	0.91	116.87	97.08	0.08	-18.09
1 uM	98.45	0.87	84.20	95.79	0.08	-21.56
10 uM	91.00	0.87	-259.17	80.23	0.03	-42.64

Compound	Normalized Values (A and B Sets)	Responses

GW4064

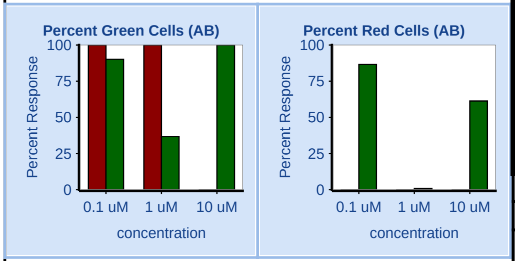

Average Response

Dose	Green Signal	Actual Pct Green Cells	Normalized Pct Green Cells	Red Signal	Actual Pct Red Cells	Normalized Pct Red Cells
0.1 uM	99.95	0.92	118.77	102.43	0.16	26.60
1 uM	97.04	0.85	70.87	85.42	0.08	-20.04
10 uM	98.21	0.83	-652.70	105.38	0.15	10.68

Compound | Normalized Values (A and B Sets) | Responses

GW5074

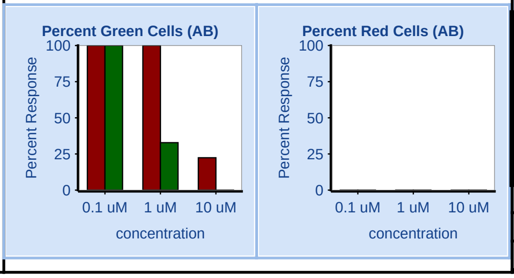

Average Response

Dose	Green Signal	Actual Pct Green Cells	Normalized Pct Green Cells	Red Signal	Actual Pct Red Cells	Normalized Pct Red Cells
0.1 uM	96.19	0.92	171.18	82.24	0.04	-42.15
1 uM	100.58	0.85	89.77	85.61	0.05	-43.58
10 uM	77.54	0.61	-51.34	98.67	0.09	-13.40

Compound | Normalized Values (A and B Sets) | Responses

GW9508

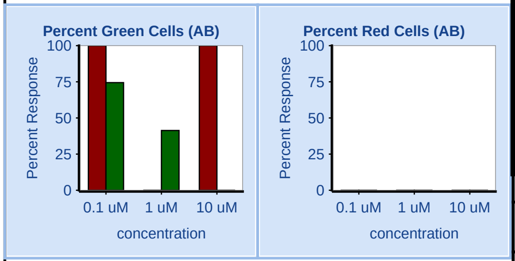

Average Response

Dose	Green Signal	Actual Pct Green Cells	Normalized Pct Green Cells	Red Signal	Actual Pct Red Cells	Normalized Pct Red Cells
0.1 uM	98.81	0.90	110.33	76.43	0.02	-60.53
1 uM	92.22	0.84	-116.03	80.72	0.05	-24.24
10 uM	83.33	0.81	2.90	85.46	0.05	-29.86

Compound | Normalized Values (A and B Sets) | Responses

GW9662

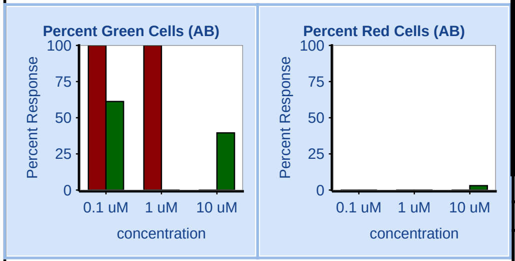

Average Response

Dose	Green Signal	Actual Pct Green Cells	Normalized Pct Green Cells	Red Signal	Actual Pct Red Cells	Normalized Pct Red Cells
0.1 uM	99.92	0.90	230.98	85.38	0.05	-37.95
1 uM	79.15	0.60	-98.22	90.39	0.07	-31.31
10 uM	78.49	0.65	-182.26	110.79	0.12	0.22

Compound | Normalized Values (A and B Sets) | Responses

GW441756

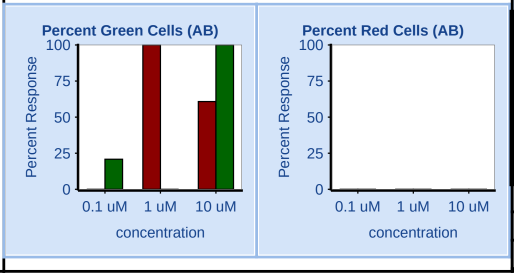

Average Response

Dose	Green Signal	Actual Pct Green Cells	Normalized Pct Green Cells	Red Signal	Actual Pct Red Cells	Normalized Pct Red Cells
0.1 uM	83.87	0.82	-58.25	65.87	0.02	-52.74
1 uM	93.08	0.81	63.30	67.29	0.03	-51.82
10 uM	101.19	0.89	89.96	77.47	0.03	-47.97

Compound | Normalized Values (A and B Sets) | Responses

GW501516

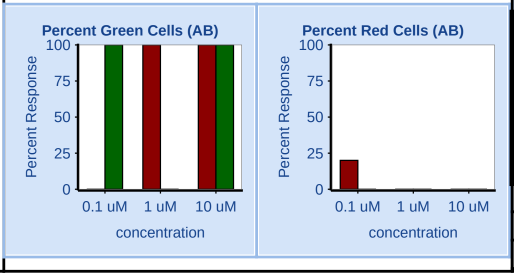

Average Response

Dose	Green Signal	Actual Pct Green Cells	Normalized Pct Green Cells	Red Signal	Actual Pct Red Cells	Normalized Pct Red Cells
0.1 uM	102.22	0.86	34.19	102.30	0.11	-23.20
1 uM	95.31	0.77	25.97	105.75	0.11	-8.31
10 uM	111.87	0.92	127.29	105.94	0.09	-17.48

Compound | Normalized Values (A and B Sets) | Responses

GW788388

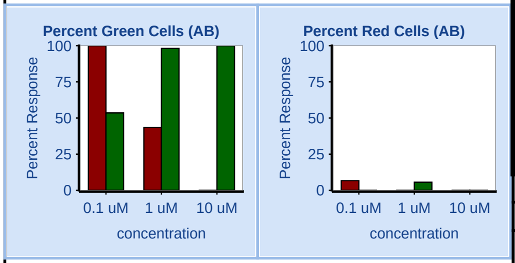

Average Response						
Dose	Green Signal	Actual Pct Green Cells	Normalized Pct Green Cells	Red Signal	Actual Pct Red Cells	Normalized Pct Red Cells
0.1 uM	98.39	0.91	108.73	94.70	0.11	-6.24
1 uM	99.19	0.83	70.83	86.93	0.08	-22.18
10 uM	106.99	0.89	-209.99	103.52	0.10	-8.51

Compound | Normalized Values (A and B Sets) | Responses

GW842166X

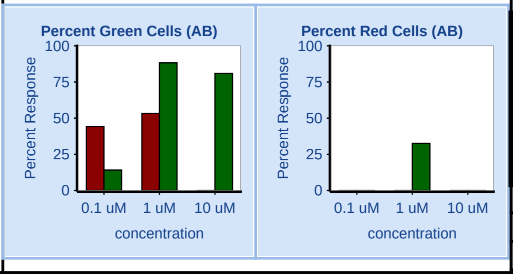

Average Response						
Dose	Green Signal	Actual Pct Green Cells	Normalized Pct Green Cells	Red Signal	Actual Pct Red Cells	Normalized Pct Red Cells
0.1 uM	88.50	0.83	29.08	83.66	0.06	-29.36
1 uM	95.12	0.83	70.78	104.39	0.12	-0.80
10 uM	94.46	0.89	32.57	99.11	0.09	-13.13

Compound | Normalized Values (A and B Sets) | Responses

Gynostemma Extract

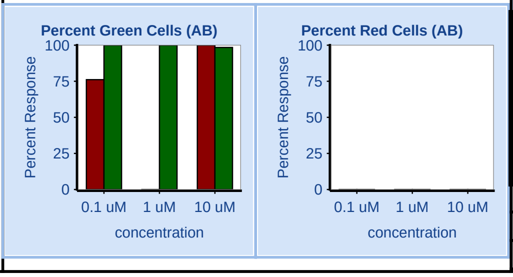

Average Response						
Dose	Green Signal	Actual Pct Green Cells	Normalized Pct Green Cells	Red Signal	Actual Pct Red Cells	Normalized Pct Red Cells
0.1 uM	99.05	0.90	109.09	89.23	0.04	-51.43
1 uM	99.60	0.88	-69.76	91.79	0.09	-4.65
10 uM	97.48	0.92	99.94	74.21	0.03	-38.56

Compound | Normalized Values (A and B Sets) | Responses

GZD824

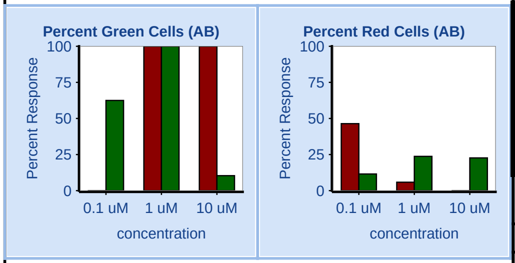

Average Response						
Dose	Green Signal	Actual Pct Green Cells	Normalized Pct Green Cells	Red Signal	Actual Pct Red Cells	Normalized Pct Red Cells
0.1 uM	87.12	0.80	-224.65	114.02	0.18	28.98
1 uM	116.38	0.90	132.55	107.59	0.16	14.79
10 uM	89.09	0.83	122.22	101.02	0.08	-23.42

Compound | Normalized Values (A and B Sets) | Responses

HA14-1

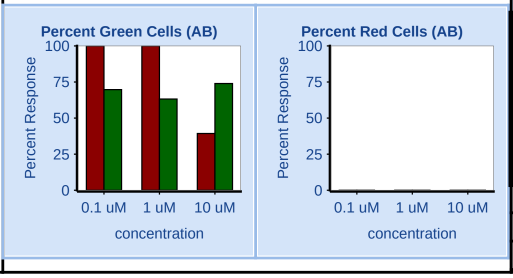

Average Response						
Dose	Green Signal	Actual Pct Green Cells	Normalized Pct Green Cells	Red Signal	Actual Pct Red Cells	Normalized Pct Red Cells
0.1 uM	99.76	0.89	105.18	71.54	0.02	-64.64
1 uM	98.26	0.88	101.80	82.41	0.03	-42.19
10 uM	92.23	0.81	56.63	80.58	0.02	-45.59

Compound	Normalized Values (A and B Sets)	Responses

Halobetasol Propionate

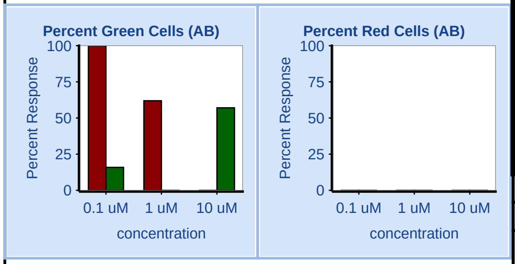

Average Response						
Dose	Green Signal	Actual Pct Green Cells	Normalized Pct Green Cells	Red Signal	Actual Pct Red Cells	Normalized Pct Red Cells
0.1 uM	93.43	0.84	70.29	88.97	0.09	-16.67
1 uM	78.26	0.61	-89.16	84.87	0.07	-28.61
10 uM	84.26	0.70	-121.85	79.92	0.05	-34.79

Compound	Normalized Values (A and B Sets)	Responses

HC-030031

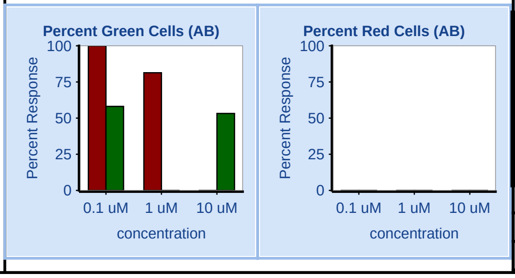

Average Response						
Dose	Green Signal	Actual Pct Green Cells	Normalized Pct Green Cells	Red Signal	Actual Pct Red Cells	Normalized Pct Red Cells
0.1 uM	94.24	0.89	155.45	75.05	0.02	-55.15
1 uM	82.74	0.71	-11.40	76.14	0.03	-54.70
10 uM	82.30	0.74	-56.70	79.32	0.02	-51.82

Compound	Normalized Values (A and B Sets)	Responses

Hematoxylin

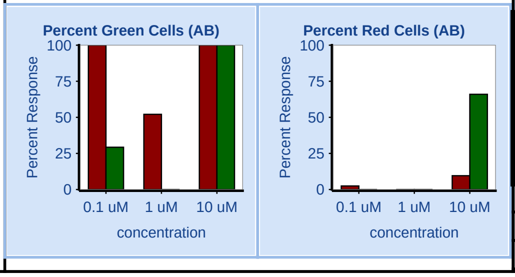

Average Response						
Dose	Green Signal	Actual Pct Green Cells	Normalized Pct Green Cells	Red Signal	Actual Pct Red Cells	Normalized Pct Red Cells
0.1 uM	92.51	0.88	78.84	87.03	0.06	-27.45
1 uM	86.53	0.74	14.86	75.52	0.02	-45.84
10 uM	98.07	0.92	223.92	119.45	0.20	37.77

Compound	Normalized Values (A and B Sets)	Responses

HER2-Inhibitor-1

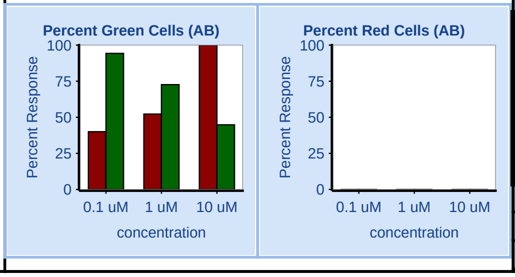

Average Response						
Dose	Green Signal	Actual Pct Green Cells	Normalized Pct Green Cells	Red Signal	Actual Pct Red Cells	Normalized Pct Red Cells
0.1 uM	94.13	0.86	67.17	71.92	0.03	-43.54
1 uM	94.62	0.82	62.45	80.06	0.03	-41.09
10 uM	97.54	0.91	319.72	81.23	0.02	-47.01

Compound	Normalized Values (A and B Sets)	Responses

Hesperadin

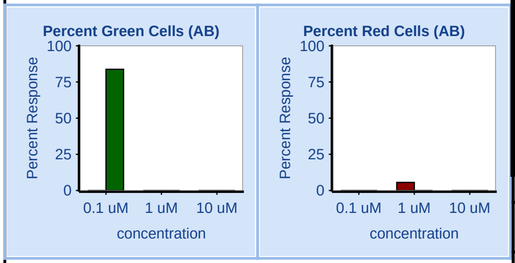

Average Response						
Dose	Green Signal	Actual Pct Green Cells	Normalized Pct Green Cells	Red Signal	Actual Pct Red Cells	Normalized Pct Red Cells
0.1 uM	94.44	0.85	37.76	87.72	0.08	-36.59
1 uM	98.02	0.70	-112.74	108.27	0.12	-2.79
10 uM	86.12	0.63	-53.63	75.46	0.04	-36.99

Compound	Normalized Values (A and B Sets)	Responses

Hexestrol

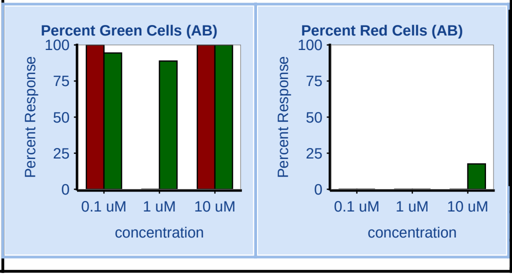

Average Response

Dose	Green Signal	Actual Pct Green Cells	Normalized Pct Green Cells	Red Signal	Actual Pct Red Cells	Normalized Pct Red Cells
0.1 uM	104.33	0.90	108.87	77.21	0.04	-39.34
1 uM	81.24	0.65	-11.62	90.14	0.06	-27.95
10 uM	105.21	0.92	228.11	106.31	0.12	-1.37

Compound	Normalized Values (A and B Sets)	Responses

HLI 373

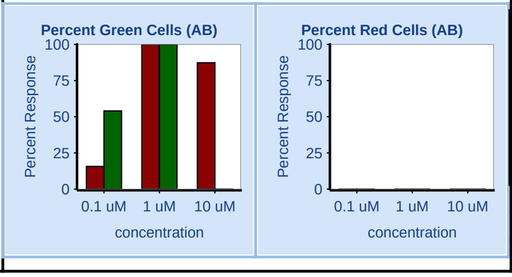

Average Response

Dose	Green Signal	Actual Pct Green Cells	Normalized Pct Green Cells	Red Signal	Actual Pct Red Cells	Normalized Pct Red Cells
0.1 uM	87.39	0.82	34.87	88.34	0.06	-36.21
1 uM	107.35	0.94	193.90	78.88	0.04	-28.54
10 uM	91.99	0.85	41.90	76.47	0.08	-17.74

Compound	Normalized Values (A and B Sets)	Responses

HMN-214

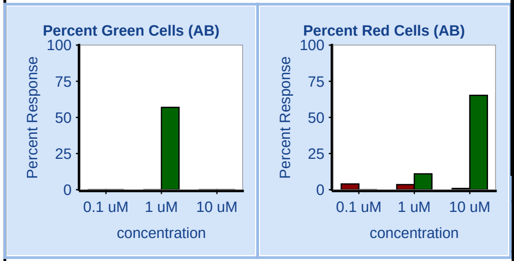

Average Response

Dose	Green Signal	Actual Pct Green Cells	Normalized Pct Green Cells	Red Signal	Actual Pct Red Cells	Normalized Pct Red Cells
0.1 uM	87.20	0.76	-16.51	102.95	0.10	-25.82
1 uM	94.09	0.74	-122.03	109.86	0.14	7.25
10 uM	96.25	0.67	-38.94	129.37	0.19	33.07

Compound	Normalized Values (A and B Sets)	Responses

Homatropine Methylbromide

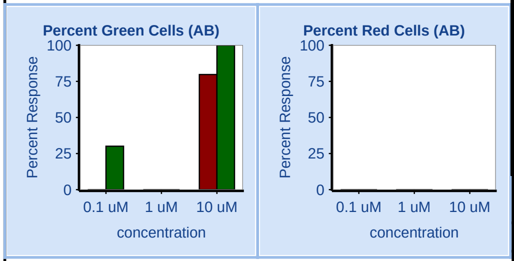

Average Response

Dose	Green Signal	Actual Pct Green Cells	Normalized Pct Green Cells	Red Signal	Actual Pct Red Cells	Normalized Pct Red Cells
0.1 uM	81.32	0.75	-365.93	88.35	0.06	-33.72
1 uM	82.07	0.66	-46.88	60.10	0.01	-63.45
10 uM	96.37	0.88	92.14	83.63	0.05	-38.19

Compound	Normalized Values (A and B Sets)	Responses

Hordenine

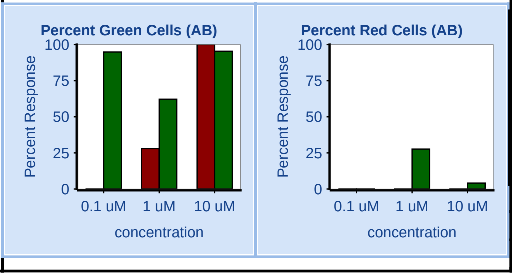

Average Response

Dose	Green Signal	Actual Pct Green Cells	Normalized Pct Green Cells	Red Signal	Actual Pct Red Cells	Normalized Pct Red Cells
0.1 uM	87.43	0.81	24.23	94.17	0.08	-18.54
1 uM	94.26	0.78	45.10	103.41	0.12	-2.09
10 uM	104.40	0.94	441.20	96.75	0.08	-20.00

Compound | Normalized Values (A and B Sets) | Responses

Hydralazine HCl

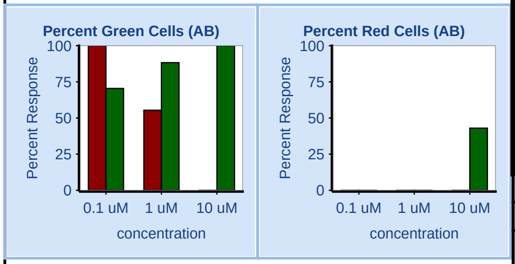

Average Response

Dose	Green Signal	Actual Pct Green Cells	Normalized Pct Green Cells	Red Signal	Actual Pct Red Cells	Normalized Pct Red Cells
0.1 uM	100.05	0.91	108.78	80.58	0.03	-40.73
1 uM	95.29	0.83	71.83	93.48	0.08	-20.21
10 uM	102.16	0.88	-156.66	102.68	0.13	2.02

Compound | Normalized Values (A and B Sets) | Responses

Hyodeoxycholic acid (HDCA)

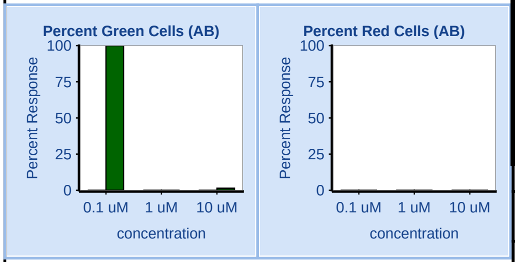

Average Response

Dose	Green Signal	Actual Pct Green Cells	Normalized Pct Green Cells	Red Signal	Actual Pct Red Cells	Normalized Pct Red Cells
0.1 uM	93.89	0.81	19.49	77.10	0.02	-63.94
1 uM	80.60	0.73	-379.02	87.73	0.06	-19.29
10 uM	72.08	0.58	-234.32	81.13	0.05	-27.23

Compound | Normalized Values (A and B Sets) | Responses

Hyoscyamine

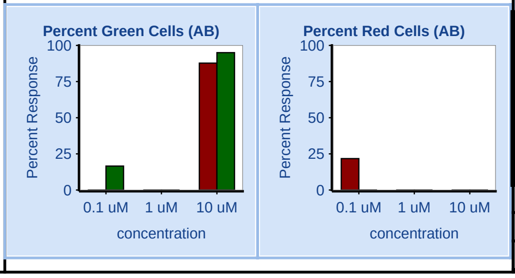

Average Response

Dose	Green Signal	Actual Pct Green Cells	Normalized Pct Green Cells	Red Signal	Actual Pct Red Cells	Normalized Pct Red Cells
0.1 uM	86.99	0.81	-88.75	91.88	0.10	-16.61
1 uM	82.51	0.69	-21.04	84.10	0.05	-44.00
10 uM	97.40	0.87	91.40	78.98	0.03	-49.44

Compound | Normalized Values (A and B Sets) | Responses

I-BET-762

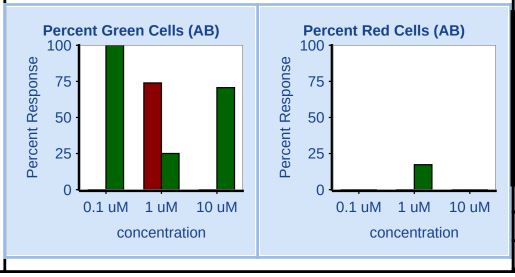

Average Response

Dose	Green Signal	Actual Pct Green Cells	Normalized Pct Green Cells	Red Signal	Actual Pct Red Cells	Normalized Pct Red Cells
0.1 uM	98.98	0.88	-47.95	86.19	0.07	-27.59
1 uM	90.31	0.79	49.49	105.84	0.13	1.01
10 uM	102.39	0.80	7.75	95.47	0.08	-21.59

Compound | Normalized Values (A and B Sets) | Responses

I-BET151 (GSK1210151A)

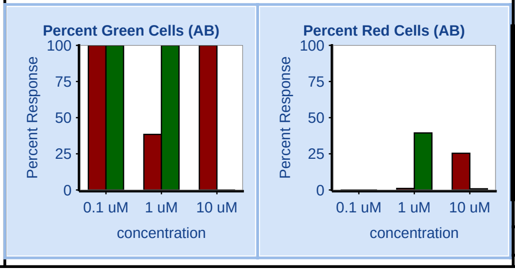

Average Response

Dose	Green Signal	Actual Pct Green Cells	Normalized Pct Green Cells	Red Signal	Actual Pct Red Cells	Normalized Pct Red Cells
0.1 uM	105.87	0.94	148.81	92.67	0.07	-21.89
1 uM	101.84	0.84	76.51	114.98	0.17	20.25

Compound | Normalized Values (A and B Sets) | Responses

10 uM	93.74	0.78	-38.92	109.36	0.15	13.10

Compound | Normalized Values (A and B Sets) | Responses

Ibrutinib (PCI-32765)

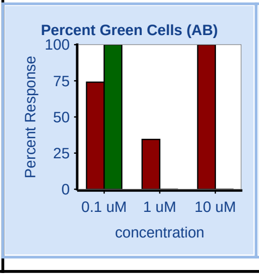

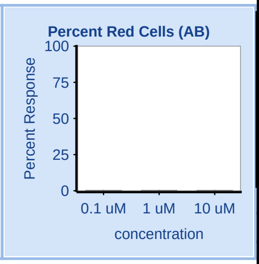

Average Response

Dose	Green Signal	Actual Pct Green Cells	Normalized Pct Green Cells	Red Signal	Actual Pct Red Cells	Normalized Pct Red Cells
0.1 uM	97.43	0.90	110.10	77.59	0.03	-39.14
1 uM	80.25	0.64	-43.41	77.60	0.03	-43.64
10 uM	90.38	0.88	122.40	72.71	0.02	-48.01

Compound | Normalized Values (A and B Sets) | Responses

Icariin

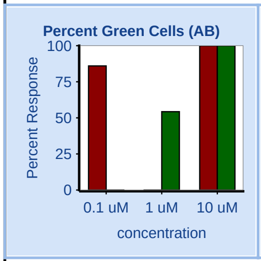

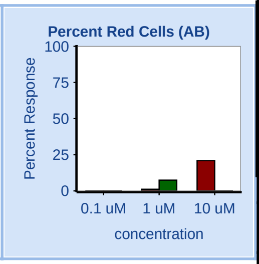

Average Response

Dose	Green Signal	Actual Pct Green Cells	Normalized Pct Green Cells	Red Signal	Actual Pct Red Cells	Normalized Pct Red Cells
0.1 uM	79.78	0.67	-84.12	89.92	0.06	-35.67
1 uM	89.98	0.85	-110.21	94.29	0.11	4.29
10 uM	111.58	0.97	144.94	92.13	0.11	0.42

Compound | Normalized Values (A and B Sets) | Responses

ICG-001

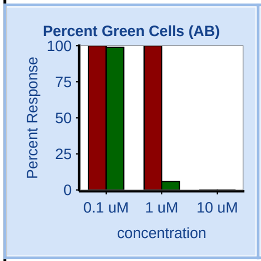

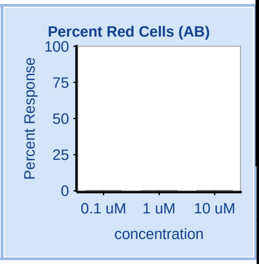

Average Response

Dose	Green Signal	Actual Pct Green Cells	Normalized Pct Green Cells	Red Signal	Actual Pct Red Cells	Normalized Pct Red Cells
0.1 uM	97.38	0.91	110.21	79.31	0.04	-36.89
1 uM	97.02	0.83	59.29	72.48	0.02	-47.97
10 uM	68.07	0.57	-2035.19	77.40	0.04	-39.84

Compound | Normalized Values (A and B Sets) | Responses

ICI 182,780

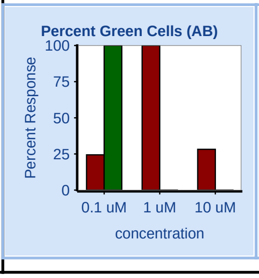

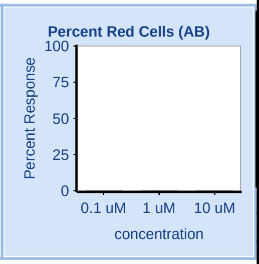

Average Response

Dose	Green Signal	Actual Pct Green Cells	Normalized Pct Green Cells	Red Signal	Actual Pct Red Cells	Normalized Pct Red Cells
0.1 uM	99.90	0.90	107.88	75.66	0.02	-64.07
1 uM	90.51	0.83	145.53	92.15	0.08	-7.88
10 uM	76.16	0.73	-74.95	78.21	0.05	-28.88

Compound | Normalized Values (A and B Sets) | Responses

Icotinib

Average Response

Dose	Green Signal			Red Signal	Actual Pct Red Cells	

Compound	Normalized Values (A and B Sets)	Responses

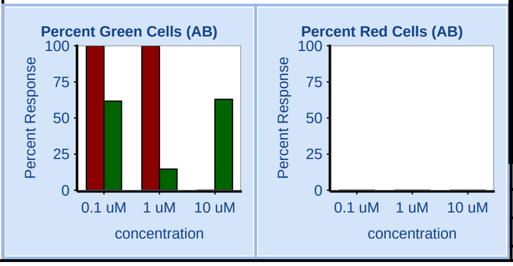

		Actual Pct Green Cells	Normalized Pct Green Cells			Normalized Pct Red Cells
0.1 uM	92.13	0.88	138.43	54.65	0.01	-58.83
1 uM	97.53	0.83	79.57	62.49	0.01	-64.10
10 uM	86.35	0.76	-38.21	100.47	0.09	-17.98

Compound	Normalized Values (A and B Sets)	Responses

IEM 1754 dihydrobroMide

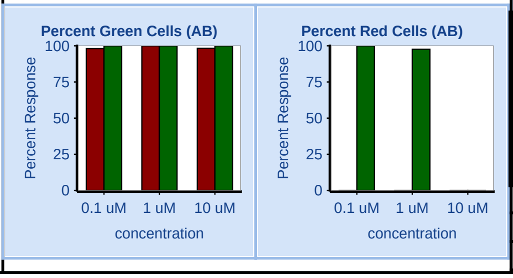

Average Response						
Dose	Green Signal	Actual Pct Green Cells	Normalized Pct Green Cells	Red Signal	Actual Pct Red Cells	Normalized Pct Red Cells
0.1 uM	97.09	0.89	102.33	107.35	0.19	39.25
1 uM	101.32	0.92	176.76	105.77	0.17	46.23
10 uM	100.55	0.92	105.08	81.75	0.05	-26.89

Compound	Normalized Values (A and B Sets)	Responses

Ifenprodil Tartrate

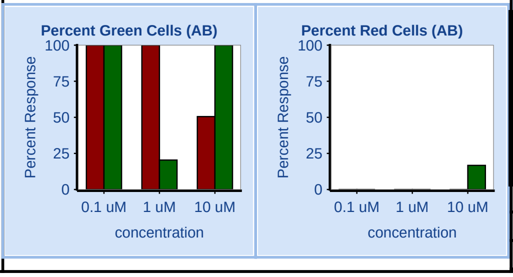

Average Response						
Dose	Green Signal	Actual Pct Green Cells	Normalized Pct Green Cells	Red Signal	Actual Pct Red Cells	Normalized Pct Red Cells
0.1 uM	98.13	0.90	114.28	74.32	0.05	-40.11
1 uM	101.60	0.86	98.29	86.32	0.05	-42.80
10 uM	97.83	0.86	76.15	93.27	0.08	-22.24

Compound	Normalized Values (A and B Sets)	Responses

Ilomastat (GM6001, Galardin)

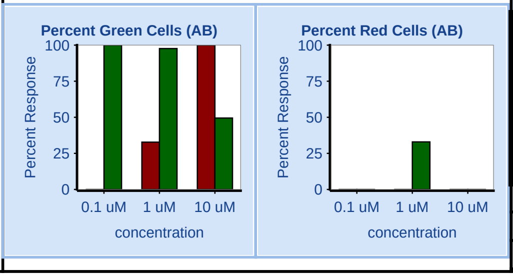

Average Response						
Dose	Green Signal	Actual Pct Green Cells	Normalized Pct Green Cells	Red Signal	Actual Pct Red Cells	Normalized Pct Red Cells
0.1 uM	99.97	0.89	-1.66	91.11	0.08	-23.39
1 uM	95.98	0.81	65.22	97.66	0.12	-7.77
10 uM	102.80	0.86	136.34	90.17	0.06	-29.72

Compound	Normalized Values (A and B Sets)	Responses

Iloperidone

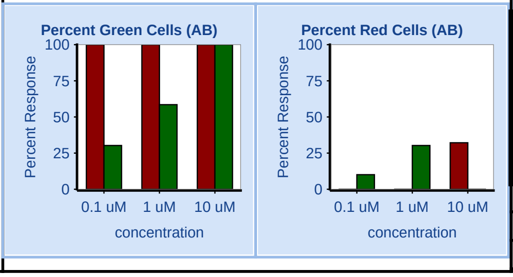

Average Response						
Dose	Green Signal	Actual Pct Green Cells	Normalized Pct Green Cells	Red Signal	Actual Pct Red Cells	Normalized Pct Red Cells
0.1 uM	94.68	0.85	76.55	110.36	0.13	-2.33
1 uM	108.08	0.88	105.41	112.56	0.13	4.62
10 uM	108.93	0.91	120.04	130.99	0.17	15.76

Compound	Normalized Values (A and B Sets)	Responses

Imidapril HCl

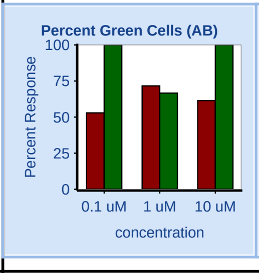

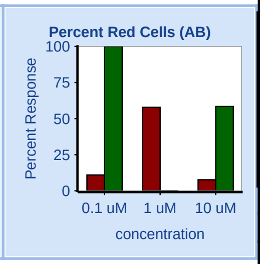

Average Response

Dose	Green Signal	Actual Pct Green Cells	Normalized Pct Green Cells	Red Signal	Actual Pct Red Cells	Normalized Pct Red Cells
0.1 uM	113.47	0.90	85.72	144.49	0.28	88.19
1 uM	106.00	0.86	69.14	116.02	0.17	17.64
10 uM	108.00	0.85	82.36	126.51	0.19	33.03

Compound	Normalized Values (A and B Sets)	Responses

INCB28060

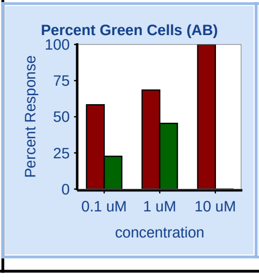

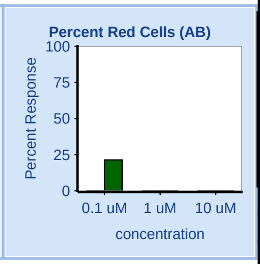

Average Response

Dose	Green Signal	Actual Pct Green Cells	Normalized Pct Green Cells	Red Signal	Actual Pct Red Cells	Normalized Pct Red Cells
0.1 uM	90.60	0.84	40.49	98.11	0.11	-3.11
1 uM	93.89	0.81	56.96	75.30	0.03	-43.83
10 uM	91.59	0.87	167.62	76.33	0.03	-44.41

Compound	Normalized Values (A and B Sets)	Responses

Indacaterol Maleate

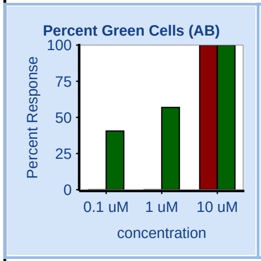

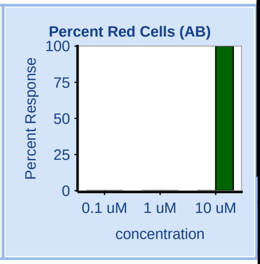

Average Response

Dose	Green Signal	Actual Pct Green Cells	Normalized Pct Green Cells	Red Signal	Actual Pct Red Cells	Normalized Pct Red Cells
0.1 uM	85.55	0.74	-39.73	78.76	0.03	-58.77
1 uM	89.37	0.81	-336.39	70.01	0.04	-27.90
10 uM	113.67	0.96	141.02	96.63	0.19	28.51

Compound	Normalized Values (A and B Sets)	Responses

Indirubin

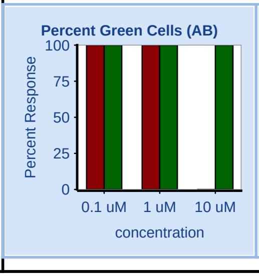

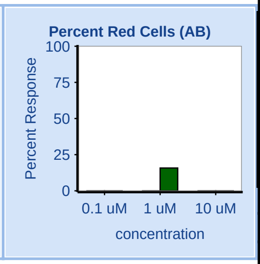

Average Response

Dose	Green Signal	Actual Pct Green Cells	Normalized Pct Green Cells	Red Signal	Actual Pct Red Cells	Normalized Pct Red Cells
0.1 uM	99.02	0.92	122.56	85.06	0.05	-29.13
1 uM	115.75	0.95	132.20	106.60	0.14	4.95
10 uM	92.74	0.90	-55.19	86.35	0.05	-35.36

Compound	Normalized Values (A and B Sets)	Responses

INK 128 (MLN0128)

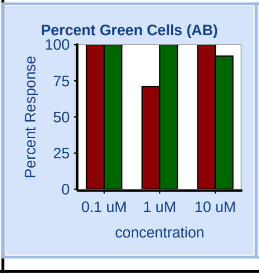

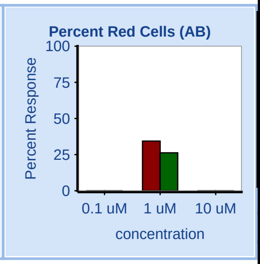

Average Response

Dose	Green Signal	Actual Pct Green Cells	Normalized Pct Green Cells	Red Signal	Actual Pct Red Cells	Normalized Pct Red Cells
0.1 uM	102.33	0.91	120.60	82.19	0.05	-31.02
1 uM	105.92	0.87	87.86	118.65	0.19	30.31
10 uM	104.25	0.93	375.12	100.62	0.09	-13.47

Compound | Normalized Values (A and B Sets) | Responses

Inosine

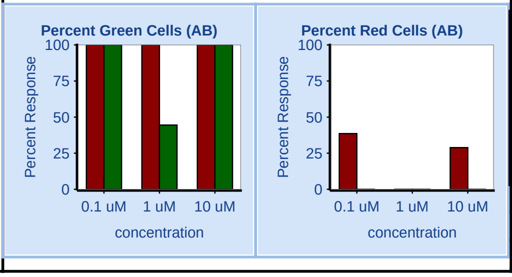

Average Response

Dose	Green Signal	Actual Pct Green Cells	Normalized Pct Green Cells	Red Signal	Actual Pct Red Cells	Normalized Pct Red Cells
0.1 uM	109.96	0.95	162.98	107.88	0.14	9.97
1 uM	105.47	0.87	83.25	89.19	0.06	-27.40
10 uM	111.83	0.94	410.14	101.93	0.10	-7.87

Compound | Normalized Values (A and B Sets) | Responses

IOWH032

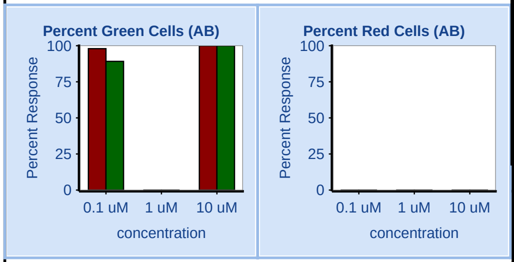

Average Response

Dose	Green Signal	Actual Pct Green Cells	Normalized Pct Green Cells	Red Signal	Actual Pct Red Cells	Normalized Pct Red Cells
0.1 uM	94.00	0.88	93.57	68.71	0.01	-69.71
1 uM	68.81	0.52	-1794.11	75.41	0.02	-39.64
10 uM	99.89	0.95	127.14	60.17	0.02	-42.63

Compound | Normalized Values (A and B Sets) | Responses

IOX2

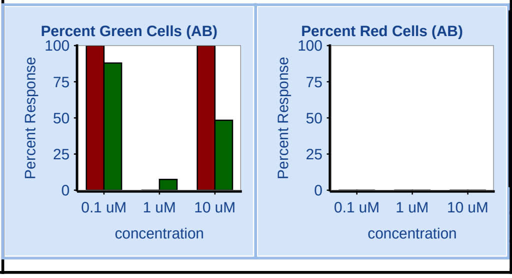

Average Response

Dose	Green Signal	Actual Pct Green Cells	Normalized Pct Green Cells	Red Signal	Actual Pct Red Cells	Normalized Pct Red Cells
0.1 uM	97.51	0.90	142.46	68.94	0.01	-56.63
1 uM	83.98	0.72	2.27	67.87	0.01	-60.40
10 uM	94.43	0.83	83.34	69.25	0.02	-55.42

Compound | Normalized Values (A and B Sets) | Responses

IPI-145 (INK1197)

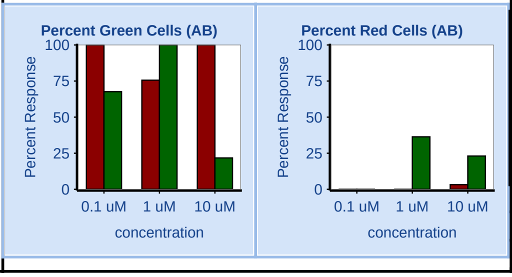

Average Response

Dose	Green Signal	Actual Pct Green Cells	Normalized Pct Green Cells	Red Signal	Actual Pct Red Cells	Normalized Pct Red Cells
0.1 uM	96.47	0.90	173.46	94.13	0.09	-19.52
1 uM	98.16	0.84	87.87	97.66	0.12	-5.48
10 uM	108.04	0.85	145.22	113.47	0.14	13.15

Compound | Normalized Values (A and B Sets) | Responses

Ipratropium Bromide

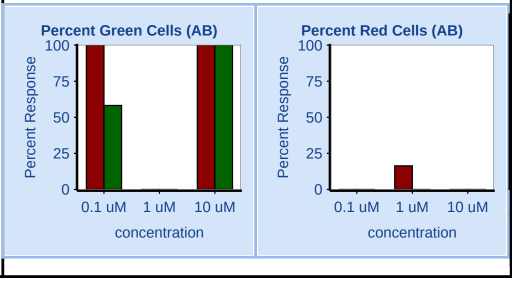

Average Response

Dose	Green Signal	Actual Pct Green Cells	Normalized Pct Green Cells	Red Signal	Actual Pct Red Cells	Normalized Pct Red Cells
0.1 uM	99.63	0.89	102.52	83.17	0.03	-58.15
1 uM	89.43	0.80	-159.14	90.95	0.10	-9.56

Compound	Normalized Values (A and B Sets)	Responses

10 uM	112.95	0.96	135.08	92.04	0.10	-6.27

Compound	Normalized Values (A and B Sets)	Responses

Irinotecan

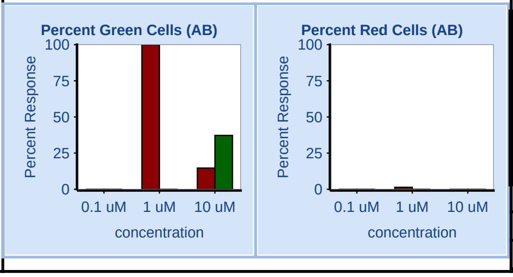

Average Response

Dose	Green Signal	Actual Pct Green Cells	Normalized Pct Green Cells	Red Signal	Actual Pct Red Cells	Normalized Pct Red Cells
0.1 uM	82.01	0.74	-40.53	92.07	0.06	-42.42
1 uM	95.90	0.66	-33.57	106.34	0.12	-4.78
10 uM	101.74	0.76	25.95	80.69	0.06	-26.47

Compound	Normalized Values (A and B Sets)	Responses

Irsogladine

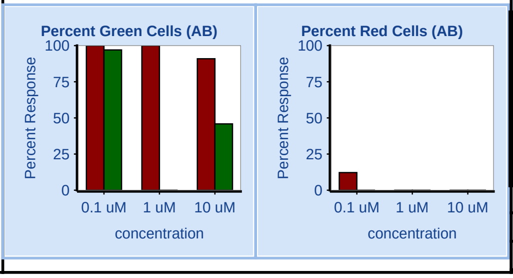

Average Response

Dose	Green Signal	Actual Pct Green Cells	Normalized Pct Green Cells	Red Signal	Actual Pct Red Cells	Normalized Pct Red Cells
0.1 uM	104.47	0.91	105.26	106.43	0.12	-14.91
1 uM	97.94	0.80	44.95	89.63	0.04	-36.89
10 uM	97.96	0.83	68.39	106.95	0.08	-23.11

Compound	Normalized Values (A and B Sets)	Responses

Isoconazole nitrate

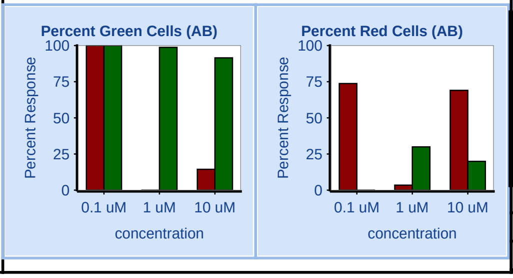

Average Response

Dose	Green Signal	Actual Pct Green Cells	Normalized Pct Green Cells	Red Signal	Actual Pct Red Cells	Normalized Pct Red Cells
0.1 uM	107.33	0.92	131.52	108.11	0.15	19.13
1 uM	100.01	0.88	-46.39	98.10	0.13	16.70
10 uM	92.37	0.87	52.97	116.82	0.21	44.48

Compound	Normalized Values (A and B Sets)	Responses

Isosorbide

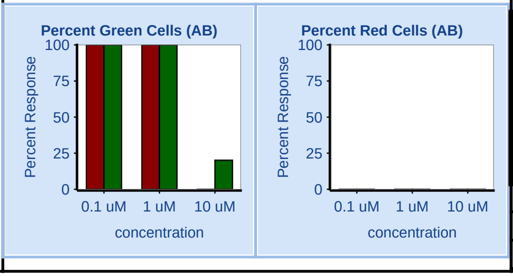

Average Response

Dose	Green Signal	Actual Pct Green Cells	Normalized Pct Green Cells	Red Signal	Actual Pct Red Cells	Normalized Pct Red Cells
0.1 uM	98.04	0.92	145.13	87.01	0.06	-33.33
1 uM	105.33	0.88	112.90	80.58	0.05	-40.84
10 uM	89.93	0.76	4.92	75.09	0.03	-49.68

Compound	Normalized Values (A and B Sets)	Responses

Isotretinoin

Average Response

Dose	Green Signal	Actual Pct Green Cells	Normalized Pct Green Cells	Red Signal	Actual Pct Red Cells	Normalized Pct Red Cells

Compound	Normalized Values (A and B Sets)	Responses

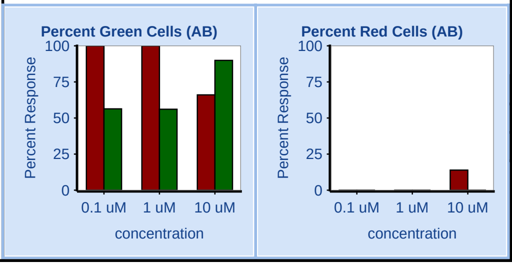

0.1 uM	98.68	0.87	83.49	76.57	0.02	-63.10
1 uM	101.21	0.87	83.97	90.69	0.05	-33.89
10 uM	93.44	0.84	77.97	126.62	0.13	-2.18

Compound	Normalized Values (A and B Sets)	Responses

Isovaleramide

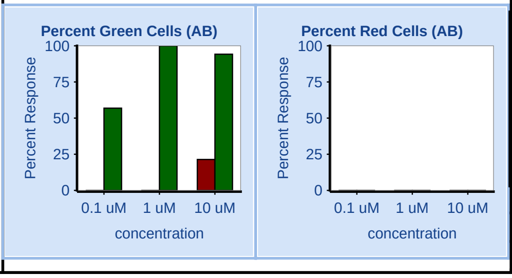

Average Response

Dose	Green Signal	Actual Pct Green Cells	Normalized Pct Green Cells	Red Signal	Actual Pct Red Cells	Normalized Pct Red Cells
0.1 uM	90.11	0.85	-6.21	89.18	0.07	-27.46
1 uM	89.22	0.65	-38.04	76.65	0.03	-52.41
10 uM	98.73	0.85	57.79	75.23	0.02	-52.55

Compound	Normalized Values (A and B Sets)	Responses

Ispinesib (SB-715992)

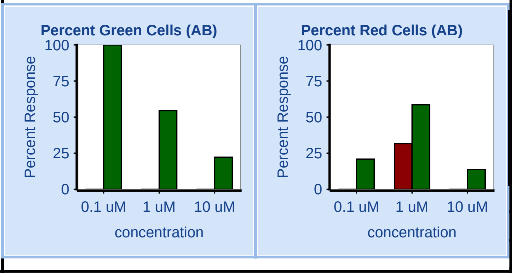

Average Response

Dose	Green Signal	Actual Pct Green Cells	Normalized Pct Green Cells	Red Signal	Actual Pct Red Cells	Normalized Pct Red Cells
0.1 uM	105.46	0.84	-2.51	118.10	0.15	9.39
1 uM	108.76	0.78	-45.56	127.69	0.22	45.06
10 uM	86.45	0.61	-68.44	101.29	0.12	0.80

Compound	Normalized Values (A and B Sets)	Responses

Istradefylline

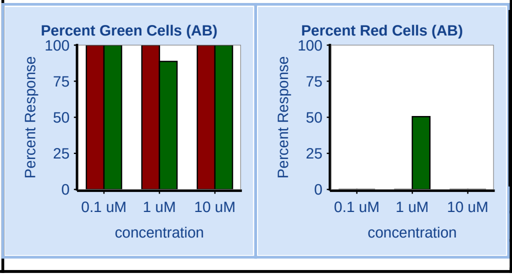

Average Response

Dose	Green Signal	Actual Pct Green Cells	Normalized Pct Green Cells	Red Signal	Actual Pct Red Cells	Normalized Pct Red Cells
0.1 uM	102.56	0.92	129.56	85.16	0.06	-25.08
1 uM	108.03	0.91	102.95	104.85	0.14	8.42
10 uM	97.47	0.93	293.41	89.23	0.04	-37.85

Compound	Normalized Values (A and B Sets)	Responses

Itraconazole

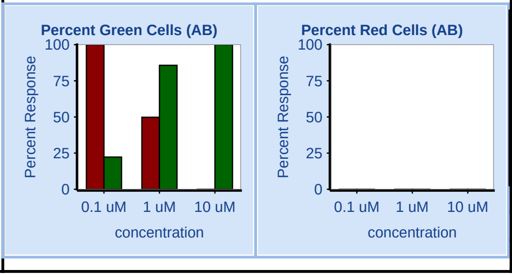

Average Response

Dose	Green Signal	Actual Pct Green Cells	Normalized Pct Green Cells	Red Signal	Actual Pct Red Cells	Normalized Pct Red Cells
0.1 uM	103.58	0.89	87.30	78.04	0.03	-39.68
1 uM	95.24	0.83	67.77	80.53	0.03	-42.16
10 uM	95.01	0.90	35.78	63.57	0.01	-55.37

Compound | Normalized Values (A and B Sets) | Responses

ITX 3

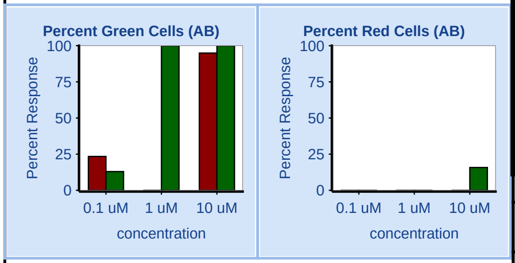

Average Response						
Dose	Green Signal	Actual Pct Green Cells	Normalized Pct Green Cells	Red Signal	Actual Pct Red Cells	Normalized Pct Red Cells
0.1 uM	87.58	0.80	18.19	69.98	0.01	-68.87
1 uM	95.85	0.86	-195.06	64.68	0.02	-38.25
10 uM	96.87	0.92	101.80	71.10	0.08	-17.77

Compound | Normalized Values (A and B Sets) | Responses

IU1

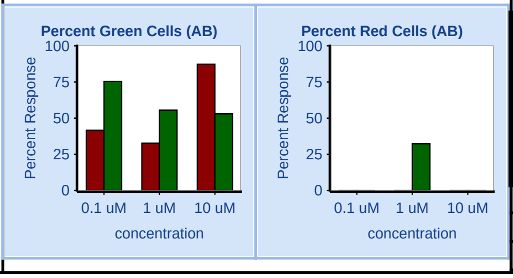

Average Response						
Dose	Green Signal	Actual Pct Green Cells	Normalized Pct Green Cells	Red Signal	Actual Pct Red Cells	Normalized Pct Red Cells
0.1 uM	98.42	0.87	58.49	84.64	0.06	-34.73
1 uM	94.92	0.78	44.12	95.15	0.11	-12.73
10 uM	94.14	0.82	70.14	84.50	0.04	-44.44

Compound | Normalized Values (A and B Sets) | Responses

Ivabradine HCl

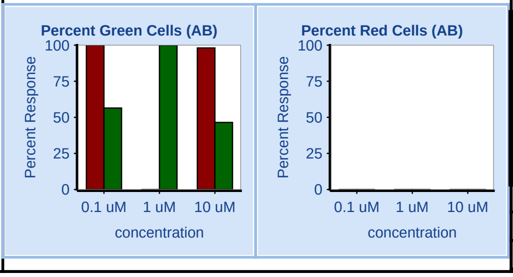

Average Response						
Dose	Green Signal	Actual Pct Green Cells	Normalized Pct Green Cells	Red Signal	Actual Pct Red Cells	Normalized Pct Red Cells
0.1 uM	103.86	0.88	100.39	91.85	0.06	-37.26
1 uM	100.76	0.86	29.02	104.02	0.09	-17.45
10 uM	97.34	0.84	72.28	90.41	0.03	-40.98

Compound | Normalized Values (A and B Sets) | Responses

Ivacaftor (VX-770)

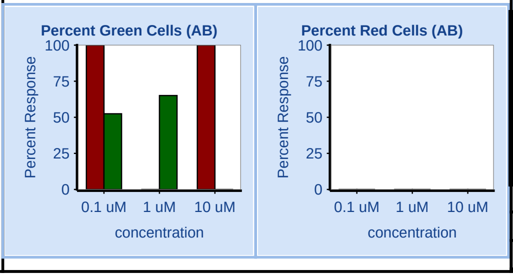

Average Response						
Dose	Green Signal	Actual Pct Green Cells	Normalized Pct Green Cells	Red Signal	Actual Pct Red Cells	Normalized Pct Red Cells
0.1 uM	92.21	0.87	81.66	84.04	0.04	-51.82
1 uM	95.65	0.83	22.30	79.40	0.03	-45.94
10 uM	94.43	0.82	53.80	95.50	0.04	-36.59

Compound | Normalized Values (A and B Sets) | Responses

IWP-2

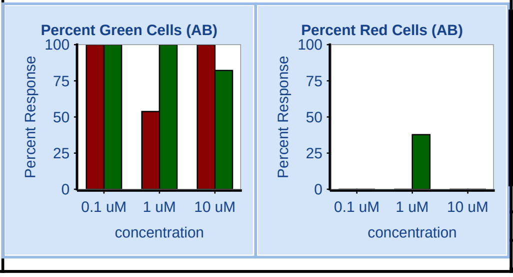

Average Response						
Dose	Green Signal	Actual Pct Green Cells	Normalized Pct Green Cells	Red Signal	Actual Pct Red Cells	Normalized Pct Red Cells
0.1 uM	100.57	0.92	161.16	87.12	0.07	-28.45
1 uM	104.12	0.84	85.32	91.30	0.12	-9.47
10 uM	106.13	0.89	133.30	83.30	0.07	-27.37

Compound	Normalized Values (A and B Sets)	Responses

IWR-1-endo

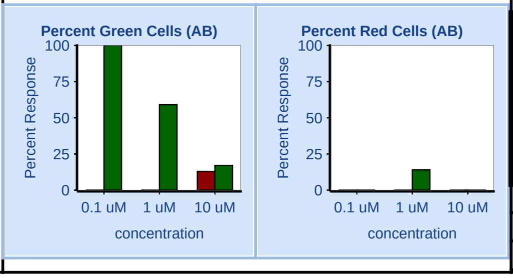

Average Response						
Dose	Green Signal	Actual Pct Green Cells	Normalized Pct Green Cells	Red Signal	Actual Pct Red Cells	Normalized Pct Red Cells
0.1 uM	96.76	0.90	37.86	80.20	0.05	-40.10
1 uM	89.18	0.71	-5.94	97.58	0.11	-9.42
10 uM	86.69	0.76	15.04	89.62	0.04	-39.78

Compound	Normalized Values (A and B Sets)	Responses

JIB-04

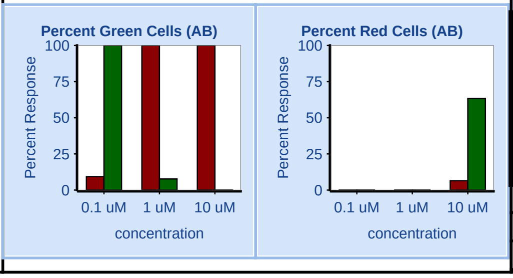

Average Response						
Dose	Green Signal	Actual Pct Green Cells	Normalized Pct Green Cells	Red Signal	Actual Pct Red Cells	Normalized Pct Red Cells
0.1 uM	92.56	0.84	55.55	83.72	0.04	-52.29
1 uM	95.57	0.87	191.99	75.48	0.02	-34.63
10 uM	95.37	0.85	36.88	111.08	0.20	34.89

Compound	Normalized Values (A and B Sets)	Responses

JK 184

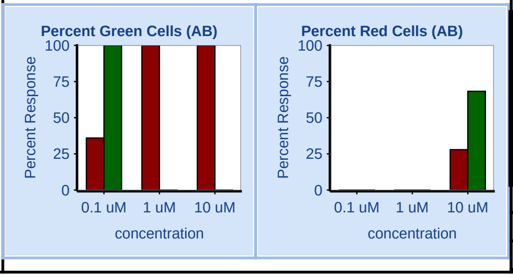

Average Response						
Dose	Green Signal	Actual Pct Green Cells	Normalized Pct Green Cells	Red Signal	Actual Pct Red Cells	Normalized Pct Red Cells
0.1 uM	102.64	0.87	77.08	87.70	0.08	-25.15
1 uM	87.30	0.73	-41.06	82.80	0.06	-23.25
10 uM	108.82	0.84	34.17	113.24	0.23	48.10

Compound	Normalized Values (A and B Sets)	Responses

JNJ-7706621

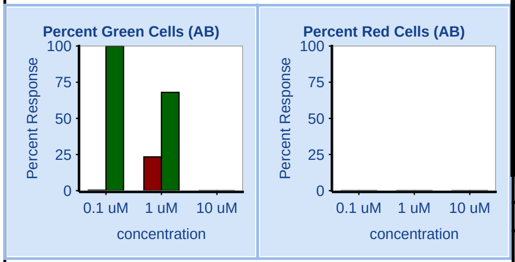

Average Response						
Dose	Green Signal	Actual Pct Green Cells	Normalized Pct Green Cells	Red Signal	Actual Pct Red Cells	Normalized Pct Red Cells
0.1 uM	94.81	0.88	59.92	101.11	0.07	-37.10
1 uM	99.09	0.85	45.67	78.64	0.03	-43.61
10 uM	76.51	0.57	-102.26	100.22	0.09	-11.56

Compound	Normalized Values (A and B Sets)	Responses

JNJ-7777120

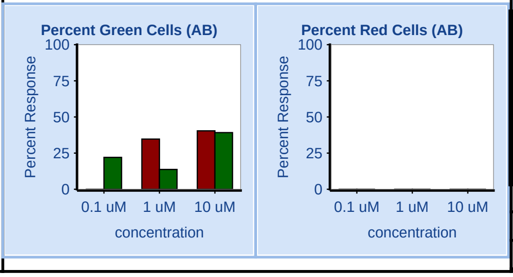

Average Response						
Dose	Green Signal	Actual Pct Green Cells	Normalized Pct Green Cells	Red Signal	Actual Pct Red Cells	Normalized Pct Red Cells
0.1 uM	86.76	0.82	-57.93	51.34	0.00	-62.00
1 uM	86.74	0.75	24.21	90.55	0.05	-44.04
10 uM	89.26	0.79	39.79	65.51	0.01	-56.16

Compound | Normalized Values (A and B Sets) | Responses

JNJ-26854165 (Serdemetan)

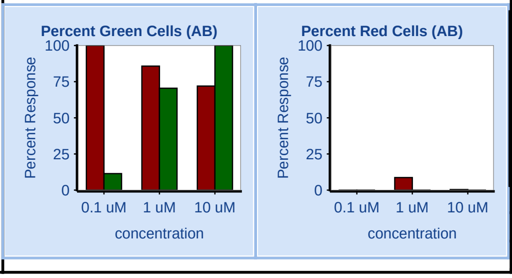

Average Response						
Dose	Green Signal	Actual Pct Green Cells	Normalized Pct Green Cells	Red Signal	Actual Pct Red Cells	Normalized Pct Red Cells
0.1 uM	100.75	0.85	87.20	89.99	0.07	-35.98
1 uM	111.50	0.87	78.13	100.96	0.12	-2.13
10 uM	114.88	0.88	108.04	103.80	0.09	-15.52

Compound | Normalized Values (A and B Sets) | Responses

JNJ-38877605

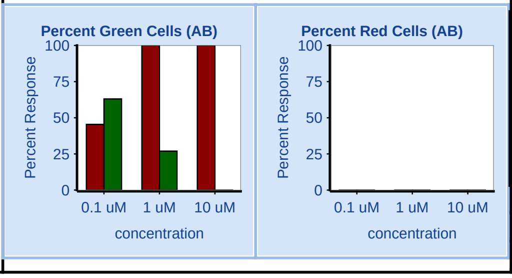

Average Response						
Dose	Green Signal	Actual Pct Green Cells	Normalized Pct Green Cells	Red Signal	Actual Pct Red Cells	Normalized Pct Red Cells
0.1 uM	92.31	0.85	54.24	73.52	0.02	-63.56
1 uM	101.03	0.85	88.12	84.75	0.05	-34.06
10 uM	88.07	0.76	12.75	85.11	0.02	-44.70

Compound | Normalized Values (A and B Sets) | Responses

JNK-IN-8

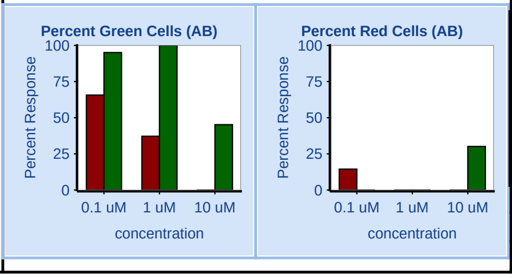

Average Response						
Dose	Green Signal	Actual Pct Green Cells	Normalized Pct Green Cells	Red Signal	Actual Pct Red Cells	Normalized Pct Red Cells
0.1 uM	95.96	0.89	80.38	94.67	0.10	-13.11
1 uM	102.46	0.83	81.30	86.42	0.05	-40.24
10 uM	96.39	0.71	-95.54	91.97	0.11	-7.35

Compound | Normalized Values (A and B Sets) | Responses

JSH-23

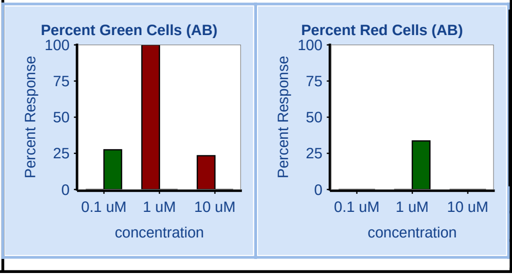

Average Response						
Dose	Green Signal	Actual Pct Green Cells	Normalized Pct Green Cells	Red Signal	Actual Pct Red Cells	Normalized Pct Red Cells
0.1 uM	86.32	0.79	10.22	71.93	0.02	-64.62
1 uM	97.59	0.85	222.30	100.30	0.10	8.02
10 uM	85.55	0.82	7.98	61.93	0.01	-45.19

Compound | Normalized Values (A and B Sets) | Responses

JTC-801

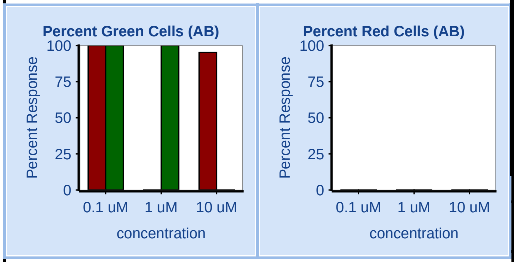

Average Response						
Dose	Green Signal	Actual Pct Green Cells	Normalized Pct Green Cells	Red Signal	Actual Pct Red Cells	Normalized Pct Red Cells
0.1 uM	110.84	0.93	139.29	95.68	0.08	-21.61
1 uM	102.19	0.89	-111.45	74.69	0.03	-35.23
10 uM	91.95	0.83	22.97	95.25	0.07	-20.02

Compound | Normalized Values (A and B Sets) | Responses

JZL 184

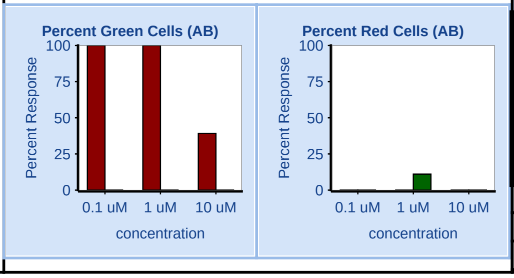

Average Response

Dose	Green Signal	Actual Pct Green Cells	Normalized Pct Green Cells	Red Signal	Actual Pct Red Cells	Normalized Pct Red Cells
0.1 uM	82.37	0.65	-97.34	83.21	0.04	-46.80
1 uM	97.38	0.83	101.35	88.58	0.07	-7.33
10 uM	81.99	0.76	-42.07	56.39	0.01	-47.28

Compound | Normalized Values (A and B Sets) | Responses

JZL184

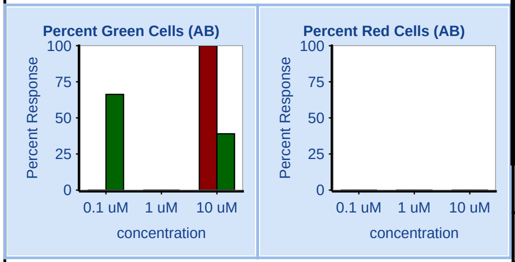

Average Response

Dose	Green Signal	Actual Pct Green Cells	Normalized Pct Green Cells	Red Signal	Actual Pct Red Cells	Normalized Pct Red Cells
0.1 uM	89.59	0.81	-207.63	84.42	0.09	-19.85
1 uM	66.25	0.43	-201.21	84.25	0.08	-26.29
10 uM	97.93	0.82	84.93	88.63	0.08	-21.00

Compound | Normalized Values (A and B Sets) | Responses

Kaempferol

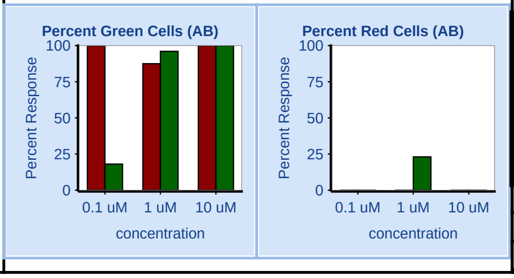

Average Response

Dose	Green Signal	Actual Pct Green Cells	Normalized Pct Green Cells	Red Signal	Actual Pct Red Cells	Normalized Pct Red Cells
0.1 uM	87.37	0.87	74.86	78.93	0.04	-37.69
1 uM	102.56	0.88	91.76	95.85	0.11	-7.10
10 uM	103.84	0.93	245.65	88.64	0.07	-27.27

Compound | Normalized Values (A and B Sets) | Responses

Ki8751

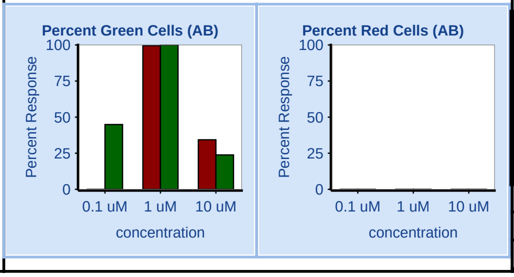

Average Response

Dose	Green Signal	Actual Pct Green Cells	Normalized Pct Green Cells	Red Signal	Actual Pct Red Cells	Normalized Pct Red Cells
0.1 uM	89.72	0.81	19.13	97.58	0.08	-32.01
1 uM	102.15	0.91	106.78	101.70	0.10	-14.00
10 uM	92.87	0.77	29.07	96.19	0.07	-25.04

Compound | Normalized Values (A and B Sets) | Responses

Ki16198

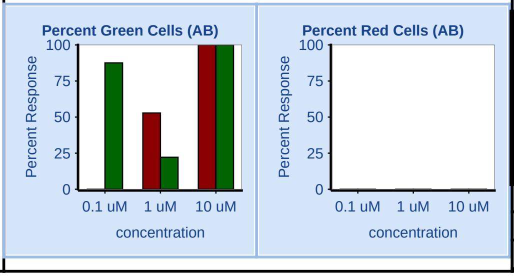

Average Response

Dose	Green Signal	Actual Pct Green Cells	Normalized Pct Green Cells	Red Signal	Actual Pct Red Cells	Normalized Pct Red Cells
0.1 uM	87.69	0.86	-30.68	57.43	0.01	-59.44
1 uM	88.25	0.77	37.50	77.69	0.03	-54.31
10 uM	98.99	0.90	128.37	59.00	0.01	-58.91

Compound	Normalized Values (A and B Sets)	Responses

Ki16425

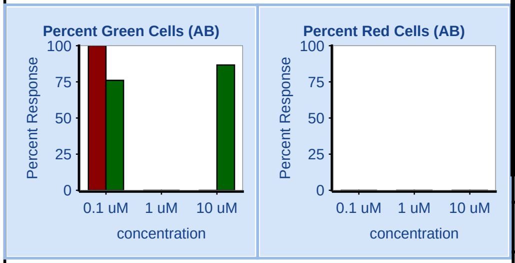

Average Response

Dose	Green Signal	Actual Pct Green Cells	Normalized Pct Green Cells	Red Signal	Actual Pct Red Cells	Normalized Pct Red Cells
0.1 uM	100.29	0.89	91.70	69.16	0.01	-66.78
1 uM	71.74	0.52	-407.34	69.35	0.02	-46.49
10 uM	82.65	0.74	17.06	99.62	0.04	-34.89

Compound	Normalized Values (A and B Sets)	Responses

KPT-185

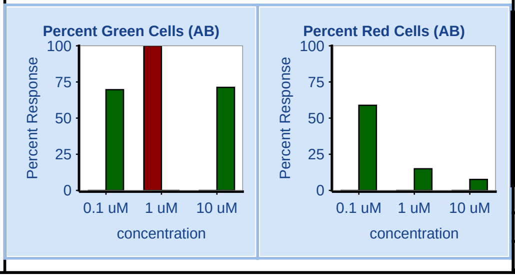

Average Response

Dose	Green Signal	Actual Pct Green Cells	Normalized Pct Green Cells	Red Signal	Actual Pct Red Cells	Normalized Pct Red Cells
0.1 uM	101.86	0.84	-79.67	108.32	0.17	24.54
1 uM	90.82	0.70	-27.00	100.35	0.13	-0.33
10 uM	89.55	0.73	-92.56	89.94	0.10	-12.36

Compound	Normalized Values (A and B Sets)	Responses

KPT-276

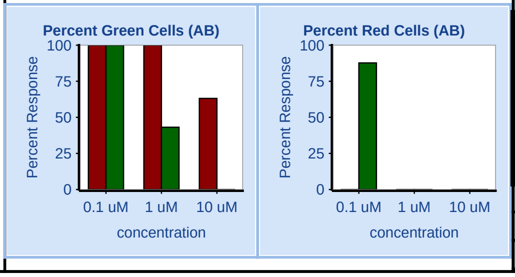

Average Response

Dose	Green Signal	Actual Pct Green Cells	Normalized Pct Green Cells	Red Signal	Actual Pct Red Cells	Normalized Pct Red Cells
0.1 uM	112.31	0.92	127.77	114.93	0.18	39.50
1 uM	96.43	0.88	173.09	85.67	0.06	-20.02
10 uM	83.16	0.72	-75.18	68.51	0.05	-31.16

Compound	Normalized Values (A and B Sets)	Responses

KU-60019

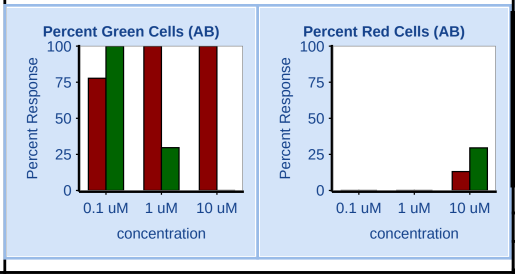

Average Response

Dose	Green Signal	Actual Pct Green Cells	Normalized Pct Green Cells	Red Signal	Actual Pct Red Cells	Normalized Pct Red Cells
0.1 uM	113.14	0.92	104.32	90.86	0.07	-36.89
1 uM	103.93	0.85	83.63	99.08	0.10	-13.02
10 uM	92.19	0.73	-11.72	129.70	0.17	21.30

Compound	Normalized Values (A and B Sets)	Responses

KU-0063794

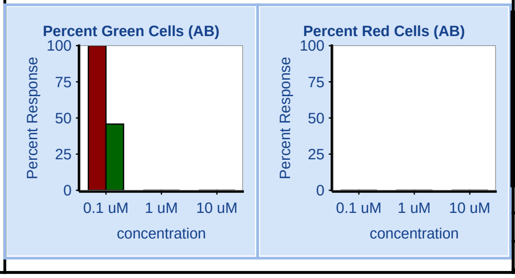

Average Response

Dose	Green Signal	Actual Pct Green Cells	Normalized Pct Green Cells	Red Signal	Actual Pct Red Cells	Normalized Pct Red Cells
0.1 uM	93.84	0.87	87.60	70.31	0.02	-61.52
1 uM	83.22	0.74	-43.86	94.83	0.04	-36.86
10 uM	76.06	0.68	-32.05	114.42	0.08	-22.54

Compound	Normalized Values (A and B Sets)	Responses

KW-2449

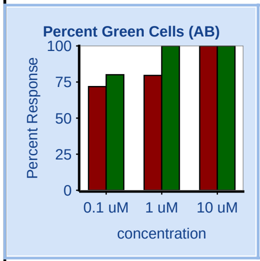

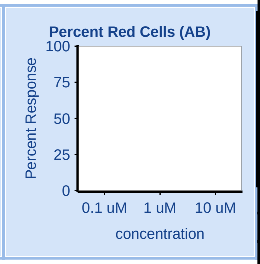

Average Response

Dose	Green Signal	Actual Pct Green Cells	Normalized Pct Green Cells	Red Signal	Actual Pct Red Cells	Normalized Pct Red Cells
0.1 uM	92.80	0.87	75.95	68.60	0.02	-44.75
1 uM	101.18	0.89	103.08	76.49	0.02	-45.46
10 uM	101.29	0.94	422.88	92.93	0.05	-32.68

Compound	Normalized Values (A and B Sets)	Responses

KW-2478

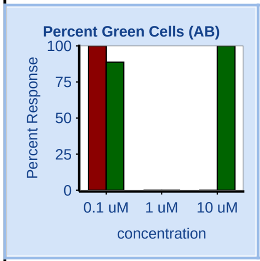

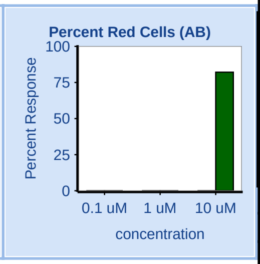

Average Response

Dose	Green Signal	Actual Pct Green Cells	Normalized Pct Green Cells	Red Signal	Actual Pct Red Cells	Normalized Pct Red Cells
0.1 uM	97.33	0.90	109.07	83.71	0.04	-36.33
1 uM	83.66	0.66	-21.64	81.24	0.05	-31.76
10 uM	135.09	0.90	-81.46	128.72	0.21	40.75

Compound	Normalized Values (A and B Sets)	Responses

KX2-391

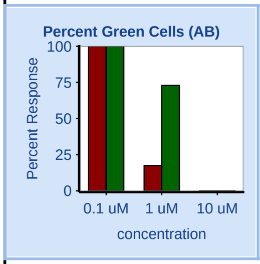

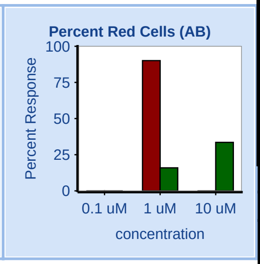

Average Response

Dose	Green Signal	Actual Pct Green Cells	Normalized Pct Green Cells	Red Signal	Actual Pct Red Cells	Normalized Pct Red Cells
0.1 uM	113.97	0.90	109.83	79.23	0.06	-27.34
1 uM	115.56	0.78	45.30	128.97	0.25	53.01
10 uM	91.88	0.68	-1279.61	92.15	0.14	8.10

Compound	Normalized Values (A and B Sets)	Responses

KY02111

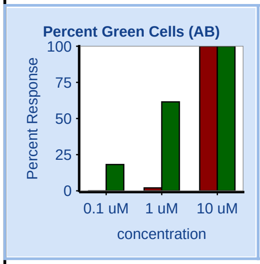

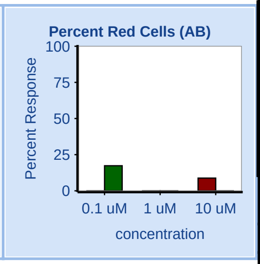

Average Response

Dose	Green Signal	Actual Pct Green Cells	Normalized Pct Green Cells	Red Signal	Actual Pct Red Cells	Normalized Pct Red Cells
0.1 uM	84.60	0.80	-111.17	90.18	0.09	-17.76
1 uM	92.96	0.76	31.78	81.69	0.06	-37.91
10 uM	113.21	0.93	144.17	98.62	0.10	-9.88

Compound	Normalized Values (A and B Sets)	Responses

L-(+)-Rhamnose Monohydrate

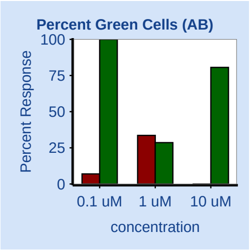

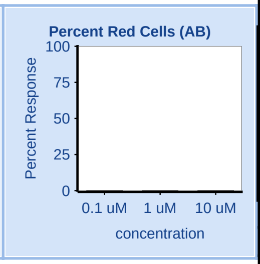

Average Response

Dose	Green Signal	Actual Pct Green Cells	Normalized Pct Green Cells	Red Signal	Actual Pct Red Cells	Normalized Pct Red Cells
0.1 uM	92.70	0.87	86.51	63.05	0.01	-50.24
1 uM	90.40	0.76	31.11	82.66	0.04	-39.94

Compound	Normalized Values (A and B Sets)	Responses

10 uM	85.06	0.81	-571.80	69.38	0.01	-54.14

Compound	Normalized Values (A and B Sets)	Responses

L-Adrenaline

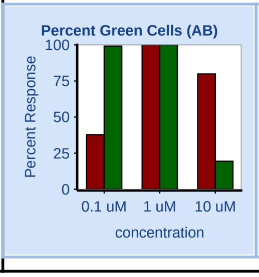

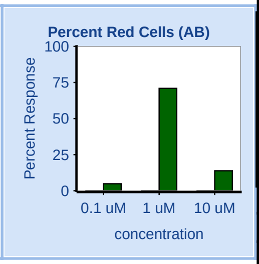

Average Response

Dose	Green Signal	Actual Pct Green Cells	Normalized Pct Green Cells	Red Signal	Actual Pct Red Cells	Normalized Pct Red Cells
0.1 uM	91.50	0.86	68.47	101.44	0.09	-18.26
1 uM	100.52	0.92	180.80	93.50	0.12	22.05
10 uM	94.93	0.86	49.67	91.26	0.10	-9.88

Compound	Normalized Values (A and B Sets)	Responses

L-Ascorbyl 6-palmitate

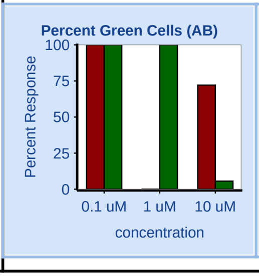

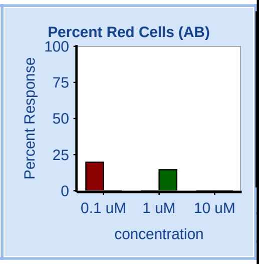

Average Response

Dose	Green Signal	Actual Pct Green Cells	Normalized Pct Green Cells	Red Signal	Actual Pct Red Cells	Normalized Pct Red Cells
0.1 uM	103.30	0.93	141.59	99.80	0.08	-20.96
1 uM	101.61	0.92	-45.52	90.91	0.08	-2.53
10 uM	85.99	0.85	38.84	78.24	0.04	-35.64

Compound	Normalized Values (A and B Sets)	Responses

L-NNA

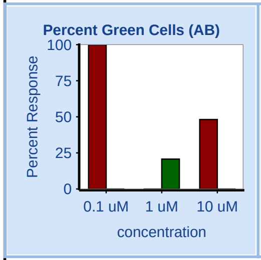

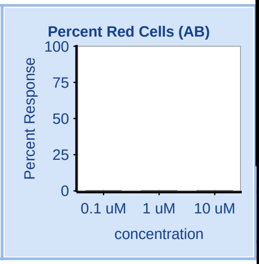

Average Response

Dose	Green Signal	Actual Pct Green Cells	Normalized Pct Green Cells	Red Signal	Actual Pct Red Cells	Normalized Pct Red Cells
0.1 uM	93.54	0.85	67.03	75.64	0.02	-62.01
1 uM	85.45	0.80	-323.70	73.03	0.02	-39.30
10 uM	70.93	0.59	-192.11	80.18	0.04	-33.56

Compound	Normalized Values (A and B Sets)	Responses

Laetrile

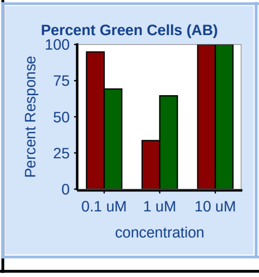

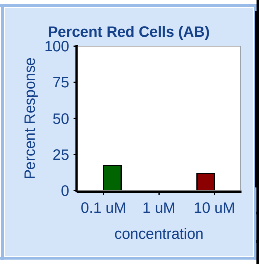

Average Response

Dose	Green Signal	Actual Pct Green Cells	Normalized Pct Green Cells	Red Signal	Actual Pct Red Cells	Normalized Pct Red Cells
0.1 uM	98.36	0.88	82.05	97.46	0.11	-1.29
1 uM	94.36	0.79	49.05	85.12	0.05	-33.54
10 uM	110.82	0.96	411.99	100.32	0.09	-15.52

Compound	Normalized Values (A and B Sets)	Responses

Lafutidine

Average Response

Dose	Green Signal			Red Signal	Actual Pct Red Cells	

Compound	Normalized Values (A and B Sets)	Responses

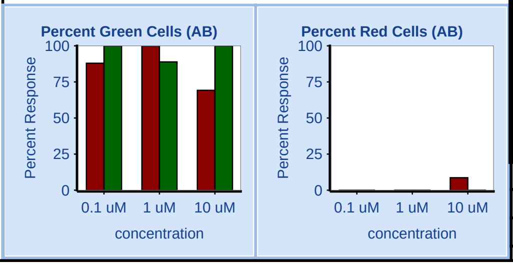

		Actual Pct Green Cells	Normalized Pct Green Cells			Normalized Pct Red Cells
0.1 uM	105.38	0.91	100.73	81.76	0.04	-51.56
1 uM	108.03	0.90	116.82	105.57	0.09	-14.61
10 uM	108.24	0.86	88.86	110.31	0.10	-12.90

Compound	Normalized Values (A and B Sets)	Responses

Lapatinib (GW-572016) Ditosylate

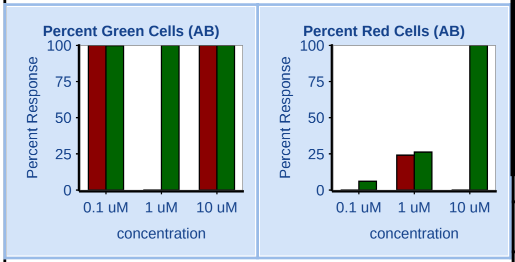

Average Response						
Dose	Green Signal	Actual Pct Green Cells	Normalized Pct Green Cells	Red Signal	Actual Pct Red Cells	Normalized Pct Red Cells
0.1 uM	101.64	0.89	104.82	100.83	0.12	-0.31
1 uM	101.92	0.82	-624.80	103.80	0.16	25.24
10 uM	122.56	0.98	161.66	120.21	0.26	63.34

Compound	Normalized Values (A and B Sets)	Responses

Lappaconite HBr

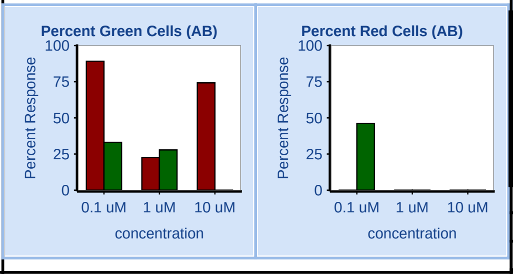

Average Response						
Dose	Green Signal	Actual Pct Green Cells	Normalized Pct Green Cells	Red Signal	Actual Pct Red Cells	Normalized Pct Red Cells
0.1 uM	92.09	0.86	61.15	92.85	0.11	1.79
1 uM	86.24	0.75	25.22	73.40	0.02	-47.31
10 uM	90.10	0.85	4.53	70.96	0.02	-47.24

Compound	Normalized Values (A and B Sets)	Responses

LAQ824 (Dacinostat)

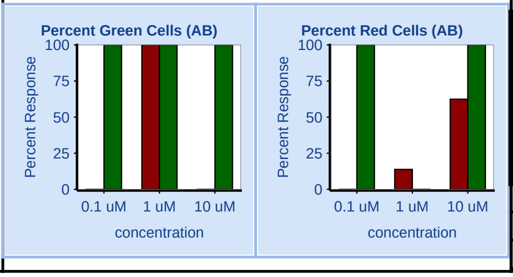

Average Response						
Dose	Green Signal	Actual Pct Green Cells	Normalized Pct Green Cells	Red Signal	Actual Pct Red Cells	Normalized Pct Red Cells
0.1 uM	97.68	0.87	31.62	151.84	0.21	48.57
1 uM	117.82	0.92	132.37	121.69	0.13	-0.19
10 uM	76.22	0.66	-25.36	161.40	0.35	98.19

Compound	Normalized Values (A and B Sets)	Responses

Laquinimod

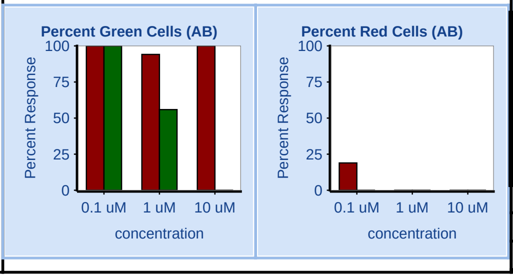

Average Response						
Dose	Green Signal	Actual Pct Green Cells	Normalized Pct Green Cells	Red Signal	Actual Pct Red Cells	Normalized Pct Red Cells
0.1 uM	103.70	0.94	144.81	110.38	0.13	7.26
1 uM	99.87	0.85	74.99	93.74	0.07	-26.40
10 uM	91.53	0.83	184.00	99.31	0.09	-17.54

Compound	Normalized Values (A and B Sets)	Responses

Latrepirdine

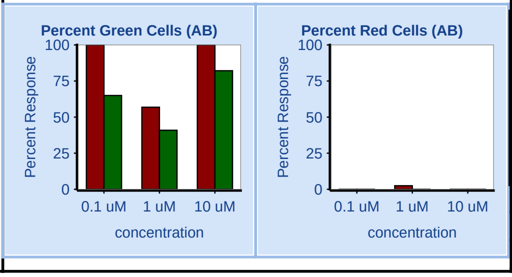

Average Response						
Dose	Green Signal	Actual Pct Green Cells	Normalized Pct Green Cells	Red Signal	Actual Pct Red Cells	Normalized Pct Red Cells
0.1 uM	107.70	0.91	131.91	88.85	0.04	-52.24
1 uM	94.75	0.84	48.87	95.57	0.07	-23.87
10 uM	97.25	0.87	95.92	91.33	0.04	-37.60

Compound	Normalized Values (A and B Sets)	Responses

LDE225 (NVP-LDE225,Erismodegib)

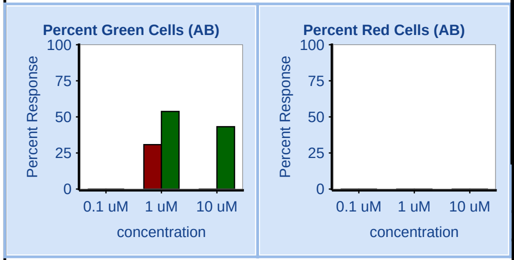

Average Response						
Dose	Green Signal	Actual Pct Green Cells	Normalized Pct Green Cells	Red Signal	Actual Pct Red Cells	Normalized Pct Red Cells
0.1 uM	68.67	0.59	-222.57	77.99	0.04	-36.57
1 uM	86.23	0.78	42.27	63.95	0.01	-51.17
10 uM	85.93	0.84	-244.53	72.76	0.02	-51.25

Compound	Normalized Values (A and B Sets)	Responses

LDK378

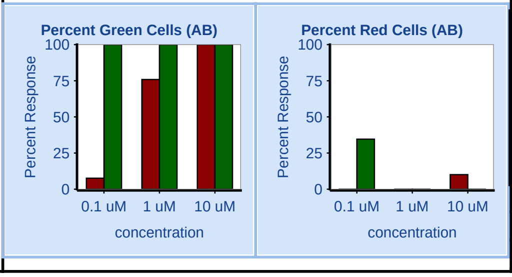

Average Response						
Dose	Green Signal	Actual Pct Green Cells	Normalized Pct Green Cells	Red Signal	Actual Pct Red Cells	Normalized Pct Red Cells
0.1 uM	103.98	0.91	71.04	102.57	0.15	14.83
1 uM	107.51	0.89	129.70	86.11	0.08	-24.99
10 uM	99.59	0.91	129.91	103.35	0.11	-5.86

Compound	Normalized Values (A and B Sets)	Responses

LDN-57444

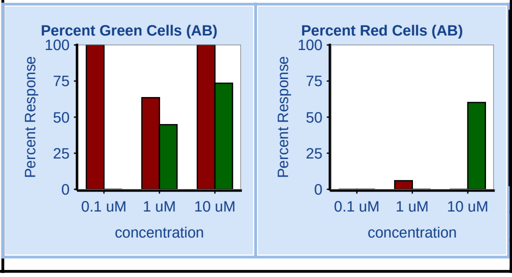

Average Response						
Dose	Green Signal	Actual Pct Green Cells	Normalized Pct Green Cells	Red Signal	Actual Pct Red Cells	Normalized Pct Red Cells
0.1 uM	79.48	0.62	-27.09	81.26	0.06	-35.29
1 uM	95.30	0.80	54.13	98.51	0.09	-18.94
10 uM	108.00	0.88	130.49	103.56	0.13	5.53

Compound	Normalized Values (A and B Sets)	Responses

Lenvatinib (E7080)

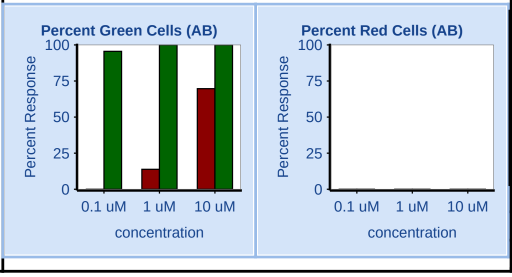

Average Response						
Dose	Green Signal	Actual Pct Green Cells	Normalized Pct Green Cells	Red Signal	Actual Pct Red Cells	Normalized Pct Red Cells
0.1 uM	89.72	0.81	-14.89	86.87	0.05	-48.94
1 uM	99.75	0.87	57.59	83.52	0.03	-42.32
10 uM	105.19	0.89	110.85	92.67	0.04	-35.04

Compound | Normalized Values (A and B Sets) | Responses

Levobetaxolol HCl

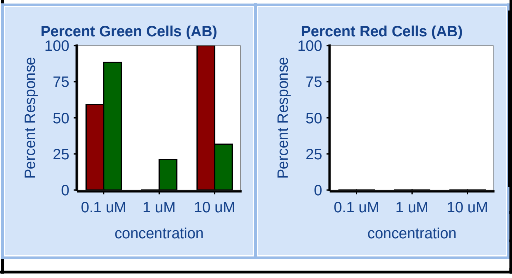

Average Response						
Dose	Green Signal	Actual Pct Green Cells	Normalized Pct Green Cells	Red Signal	Actual Pct Red Cells	Normalized Pct Red Cells
0.1 uM	96.28	0.88	73.88	68.96	0.02	-55.85
1 uM	85.08	0.70	-15.82	73.11	0.03	-51.70
10 uM	101.59	0.85	130.72	65.26	0.02	-55.79

Compound | Normalized Values (A and B Sets) | Responses

Levodropropizine

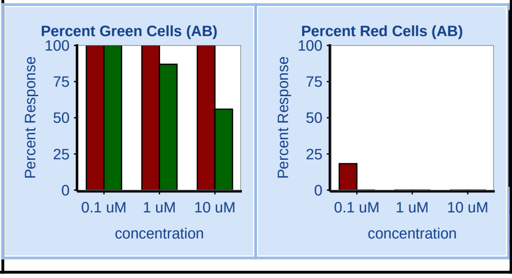

Average Response						
Dose	Green Signal	Actual Pct Green Cells	Normalized Pct Green Cells	Red Signal	Actual Pct Red Cells	Normalized Pct Red Cells
0.1 uM	108.46	0.93	208.58	108.68	0.14	8.70
1 uM	104.10	0.87	105.00	96.39	0.10	-18.17
10 uM	102.51	0.86	124.83	81.25	0.03	-45.59

Compound | Normalized Values (A and B Sets) | Responses

Levosimendan

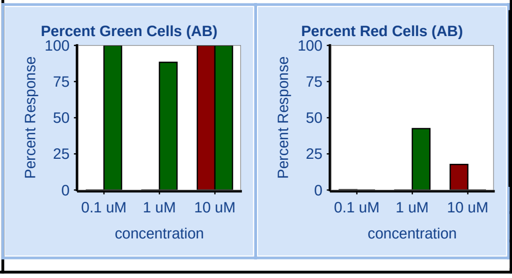

Average Response						
Dose	Green Signal	Actual Pct Green Cells	Normalized Pct Green Cells	Red Signal	Actual Pct Red Cells	Normalized Pct Red Cells
0.1 uM	99.33	0.84	45.74	91.60	0.07	-23.12
1 uM	92.61	0.75	32.38	107.57	0.15	10.90
10 uM	119.38	0.95	271.91	105.10	0.12	0.15

Compound | Normalized Values (A and B Sets) | Responses

Licofelone

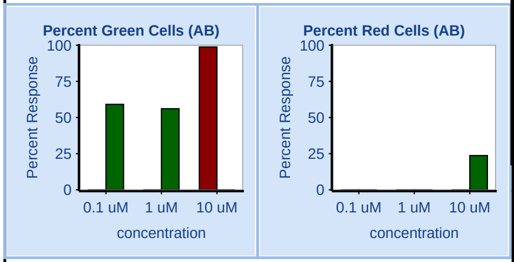

Average Response						
Dose	Green Signal	Actual Pct Green Cells	Normalized Pct Green Cells	Red Signal	Actual Pct Red Cells	Normalized Pct Red Cells
0.1 uM	89.23	0.80	20.02	88.97	0.04	-49.84
1 uM	70.30	0.53	-2199.04	73.09	0.02	-39.88
10 uM	90.31	0.85	39.11	86.68	0.09	-12.48

Compound | Normalized Values (A and B Sets) | Responses

Linagliptin

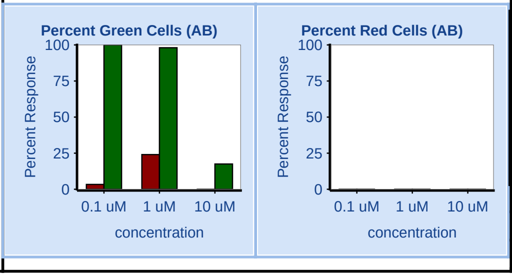

Average Response						
Dose	Green Signal	Actual Pct Green Cells	Normalized Pct Green Cells	Red Signal	Actual Pct Red Cells	Normalized Pct Red Cells
0.1 uM	99.10	0.89	56.37	88.21	0.07	-27.39
1 uM	95.92	0.80	61.04	75.99	0.02	-57.99
10 uM	83.95	0.71	-72.38	79.00	0.02	-54.85

Compound | Normalized Values (A and B Sets) | Responses

Lincomycin HCl

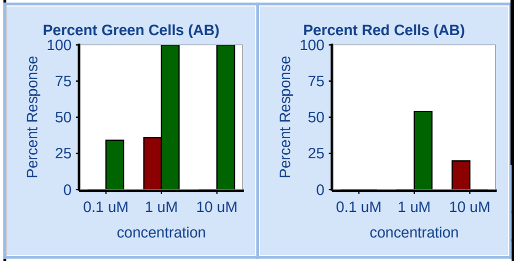

Average Response						
Dose	Green Signal	Actual Pct Green Cells	Normalized Pct Green Cells	Red Signal	Actual Pct Red Cells	Normalized Pct Red Cells
0.1 uM	75.08	0.67	-117.45	81.74	0.04	-36.26
1 uM	101.14	0.84	76.01	107.67	0.15	10.13
10 uM	88.36	0.81	-750.47	102.66	0.10	-9.62

Compound | Normalized Values (A and B Sets) | Responses

Liothyronine Sodium

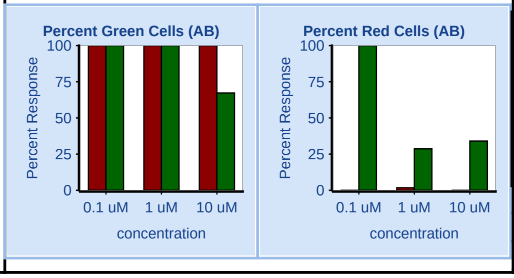

Average Response						
Dose	Green Signal	Actual Pct Green Cells	Normalized Pct Green Cells	Red Signal	Actual Pct Red Cells	Normalized Pct Red Cells
0.1 uM	104.69	0.95	224.64	116.07	0.21	44.43
1 uM	115.63	0.94	162.48	107.83	0.16	15.17
10 uM	108.47	0.89	159.33	103.66	0.14	10.93

Compound | Normalized Values (A and B Sets) | Responses

Lithium Citrate

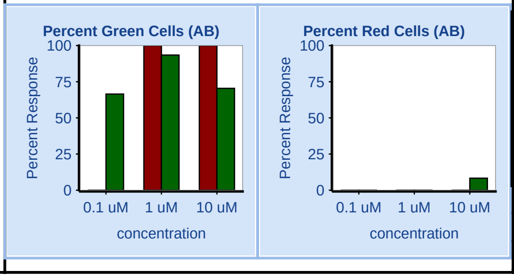

Average Response						
Dose	Green Signal	Actual Pct Green Cells	Normalized Pct Green Cells	Red Signal	Actual Pct Red Cells	Normalized Pct Red Cells
0.1 uM	91.31	0.85	-2.60	76.12	0.03	-45.97
1 uM	105.17	0.87	109.77	93.29	0.09	-22.40
10 uM	100.10	0.85	86.79	103.21	0.11	-4.64

Compound | Normalized Values (A and B Sets) | Responses

Lomeguatrib

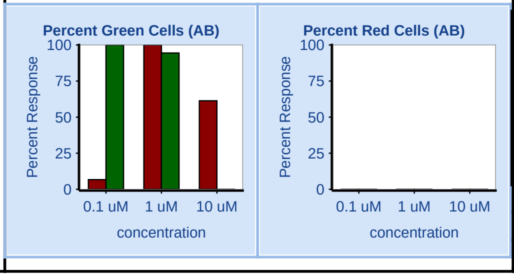

Average Response						
Dose	Green Signal	Actual Pct Green Cells	Normalized Pct Green Cells	Red Signal	Actual Pct Red Cells	Normalized Pct Red Cells
0.1 uM	93.53	0.84	55.12	72.90	0.01	-67.80
1 uM	98.69	0.90	98.10	74.43	0.02	-35.85
10 uM	84.60	0.80	-8.51	64.36	0.01	-45.56

Compound | Normalized Values (A and B Sets) | Responses

Lomerizine HCl

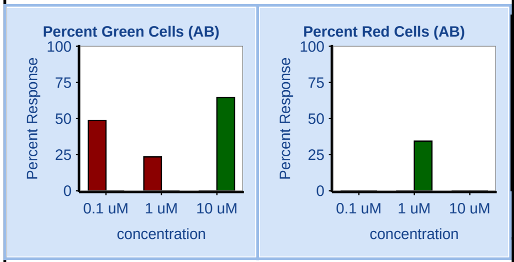

Average Response						
Dose	Green Signal	Actual Pct Green Cells	Normalized Pct Green Cells	Red Signal	Actual Pct Red Cells	Normalized Pct Red Cells
0.1 uM	69.78	0.54	-179.34	72.82	0.03	-47.73
1 uM	85.20	0.73	4.77	100.24	0.12	-7.71

Compound	Normalized Values (A and B Sets)	Responses

10 uM	94.70	0.80	23.97	72.53	0.01	-56.67

Compound	Normalized Values (A and B Sets)	Responses

Lonafarnib

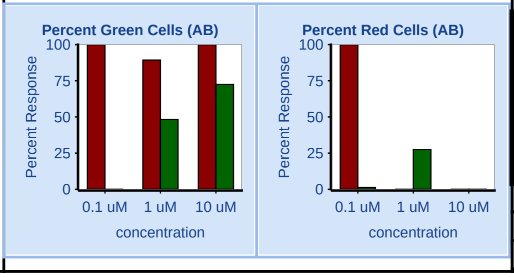

Average Response

Dose	Green Signal	Actual Pct Green Cells	Normalized Pct Green Cells	Red Signal	Actual Pct Red Cells	Normalized Pct Red Cells
0.1 uM	103.49	0.88	75.69	123.85	0.24	53.15
1 uM	99.32	0.84	68.85	108.99	0.14	7.43
10 uM	101.40	0.92	312.98	87.54	0.04	-37.52

Compound	Normalized Values (A and B Sets)	Responses

Lonidamine

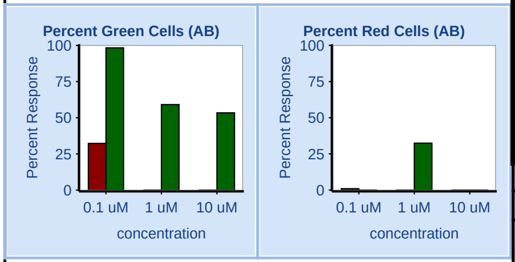

Average Response

Dose	Green Signal	Actual Pct Green Cells	Normalized Pct Green Cells	Red Signal	Actual Pct Red Cells	Normalized Pct Red Cells
0.1 uM	96.66	0.86	65.31	89.48	0.08	-16.38
1 uM	87.87	0.73	21.89	108.84	0.15	9.99
10 uM	76.64	0.66	-1723.14	80.30	0.03	-43.71

Compound	Normalized Values (A and B Sets)	Responses

Lopinavir

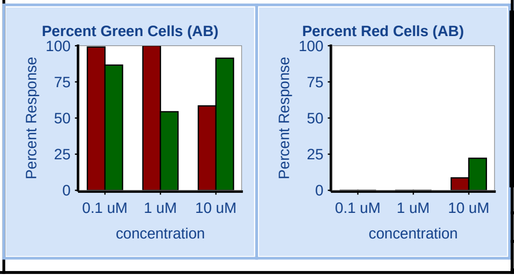

Average Response

Dose	Green Signal	Actual Pct Green Cells	Normalized Pct Green Cells	Red Signal	Actual Pct Red Cells	Normalized Pct Red Cells
0.1 uM	99.28	0.89	92.85	76.70	0.02	-62.40
1 uM	103.67	0.87	94.78	94.71	0.05	-36.15
10 uM	98.55	0.84	74.89	131.87	0.16	15.40

Compound	Normalized Values (A and B Sets)	Responses

Lorcaserin HCl

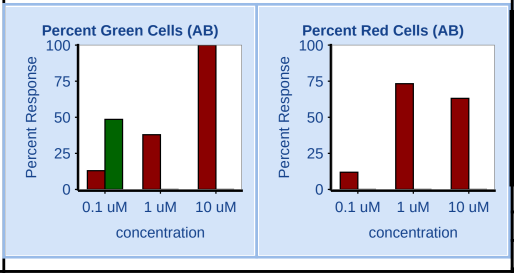

Average Response

Dose	Green Signal	Actual Pct Green Cells	Normalized Pct Green Cells	Red Signal	Actual Pct Red Cells	Normalized Pct Red Cells
0.1 uM	94.86	0.85	30.76	94.94	0.11	-9.44
1 uM	90.85	0.73	6.35	110.08	0.17	20.62
10 uM	100.17	0.77	75.53	105.94	0.14	11.59

Compound	Normalized Values (A and B Sets)	Responses

Lornoxicam

Average Response

Dose	Green Signal	Actual Pct Green Cells		Red Signal	Actual Pct Red Cells	Normalized Pct Red Cells

Compound | Normalized Values (A and B Sets) | Responses

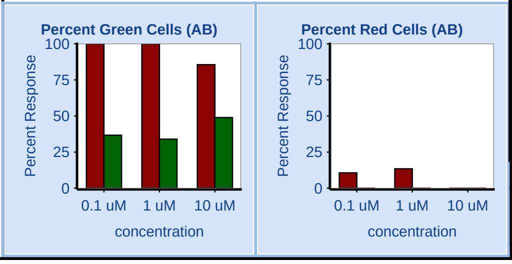

			Normalized Pct Green Cells			
0.1 uM	98.11	0.86	79.61	102.60	0.09	-29.07
1 uM	100.21	0.85	76.42	107.21	0.10	-10.66
10 uM	93.78	0.83	67.27	103.53	0.06	-27.48

Compound | Normalized Values (A and B Sets) | Responses

Losartan Potassium (DuP 753)

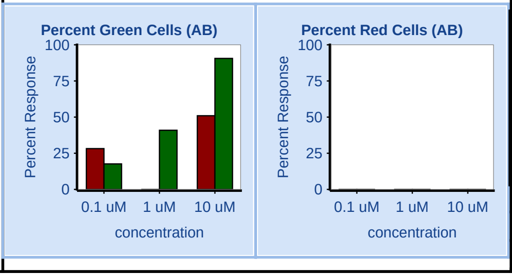

Average Response

Dose	Green Signal	Actual Pct Green Cells	Normalized Pct Green Cells	Red Signal	Actual Pct Red Cells	Normalized Pct Red Cells
0.1 uM	90.44	0.80	22.91	87.65	0.05	-43.78
1 uM	93.20	0.81	12.67	75.48	0.02	-47.52
10 uM	94.12	0.83	70.77	84.94	0.02	-44.92

Compound | Normalized Values (A and B Sets) | Responses

Lubiprostone

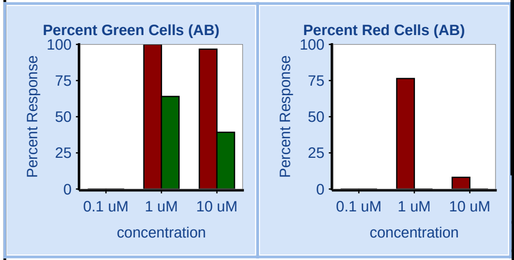

Average Response

Dose	Green Signal	Actual Pct Green Cells	Normalized Pct Green Cells	Red Signal	Actual Pct Red Cells	Normalized Pct Red Cells
0.1 uM	75.49	0.57	-123.83	84.62	0.04	-49.33
1 uM	109.97	0.87	90.14	113.97	0.17	19.48
10 uM	100.01	0.83	67.99	103.56	0.08	-22.10

Compound | Normalized Values (A and B Sets) | Responses

Luliconazole

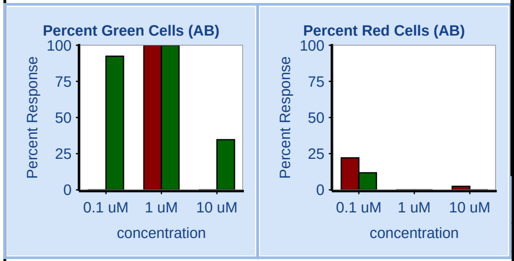

Average Response

Dose	Green Signal	Actual Pct Green Cells	Normalized Pct Green Cells	Red Signal	Actual Pct Red Cells	Normalized Pct Red Cells
0.1 uM	92.66	0.81	-269.20	104.65	0.16	16.95
1 uM	112.93	0.92	146.75	85.93	0.06	-36.95
10 uM	87.82	0.75	-23.75	94.47	0.08	-18.50

Compound | Normalized Values (A and B Sets) | Responses

Lumiracoxib

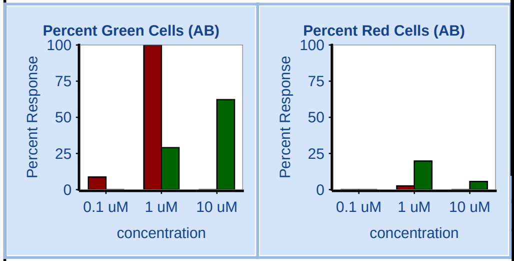

Average Response

Dose	Green Signal	Actual Pct Green Cells	Normalized Pct Green Cells	Red Signal	Actual Pct Red Cells	Normalized Pct Red Cells
0.1 uM	80.60	0.79	-17.99	75.32	0.02	-53.67
1 uM	95.28	0.84	83.91	113.49	0.15	11.18
10 uM	81.46	0.71	-112.46	100.91	0.10	-8.17

Compound	Normalized Values (A and B Sets)	Responses

LY 320135

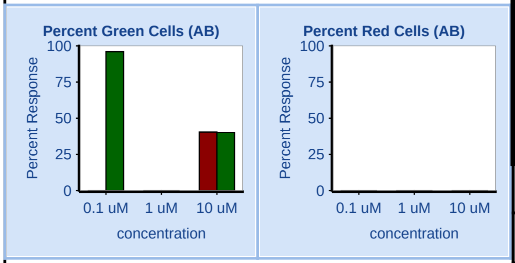

Average Response

Dose	Green Signal	Actual Pct Green Cells	Normalized Pct Green Cells	Red Signal	Actual Pct Red Cells	Normalized Pct Red Cells
0.1 uM	76.34	0.62	-156.41	83.86	0.04	-51.75
1 uM	89.86	0.80	-140.70	69.47	0.02	-39.12
10 uM	88.39	0.85	40.25	79.53	0.04	-33.81

Compound	Normalized Values (A and B Sets)	Responses

LY 333531

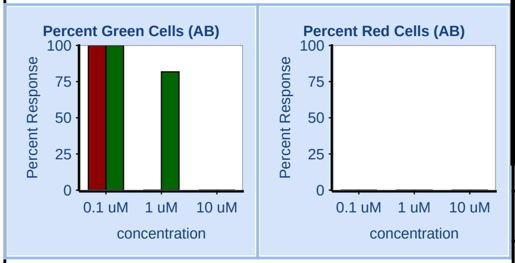

Average Response

Dose	Green Signal	Actual Pct Green Cells	Normalized Pct Green Cells	Red Signal	Actual Pct Red Cells	Normalized Pct Red Cells
0.1 uM	99.71	0.92	132.57	71.18	0.02	-65.46
1 uM	91.28	0.84	-266.85	87.72	0.07	-11.65
10 uM	59.37	0.47	-319.11	54.08	0.01	-48.87

Compound	Normalized Values (A and B Sets)	Responses

LY404039

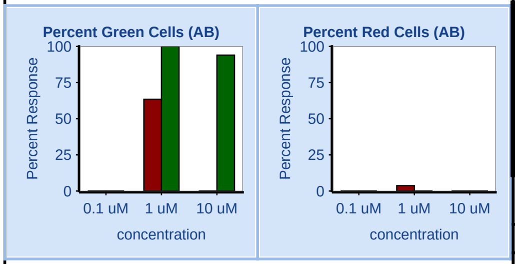

Average Response

Dose	Green Signal	Actual Pct Green Cells	Normalized Pct Green Cells	Red Signal	Actual Pct Red Cells	Normalized Pct Red Cells
0.1 uM	77.54	0.64	-600.20	90.52	0.08	-22.75
1 uM	101.06	0.84	84.91	96.41	0.10	-16.33
10 uM	97.30	0.84	42.78	80.55	0.03	-47.11

Compound	Normalized Values (A and B Sets)	Responses

LY411575

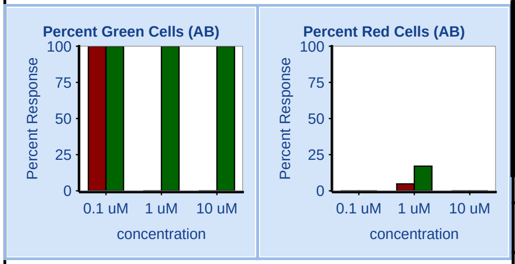

Average Response

Dose	Green Signal	Actual Pct Green Cells	Normalized Pct Green Cells	Red Signal	Actual Pct Red Cells	Normalized Pct Red Cells
0.1 uM	107.65	0.92	133.02	80.79	0.03	-57.12
1 uM	101.09	0.88	-311.51	100.62	0.12	10.94
10 uM	98.35	0.87	48.80	89.09	0.07	-19.47

Compound	Normalized Values (A and B Sets)	Responses

LY2109761

Average Response

Dose	Green Signal	Actual Pct Green Cells	Normalized Pct Green Cells	Red Signal	Actual Pct Red Cells	Normalized Pct Red Cells

Compound	Normalized Values (A and B Sets)	Responses

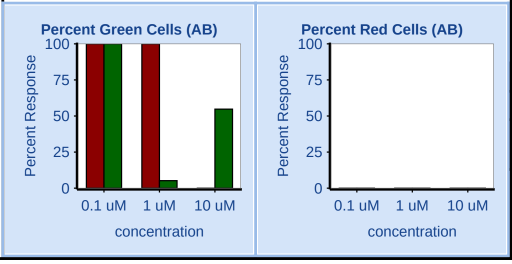

0.1 uM	104.07	0.93	139.83	70.31	0.03	-37.78
1 uM	97.44	0.83	57.63	74.75	0.03	-41.12
10 uM	82.80	0.79	-667.59	98.29	0.08	-22.08

Compound	Normalized Values (A and B Sets)	Responses

LY2157299

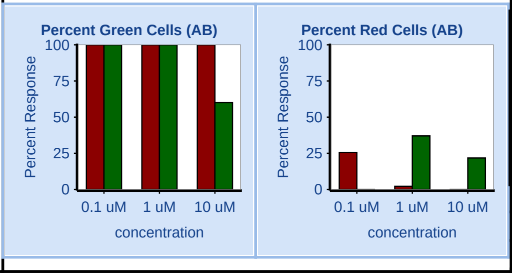

Average Response

Dose	Green Signal	Actual Pct Green Cells	Normalized Pct Green Cells	Red Signal	Actual Pct Red Cells	Normalized Pct Red Cells
0.1 uM	102.36	0.92	127.33	94.45	0.11	-6.70
1 uM	119.88	0.94	125.29	112.13	0.17	19.58
10 uM	102.97	0.92	383.40	100.19	0.11	-4.23

Compound	Normalized Values (A and B Sets)	Responses

LY2228820

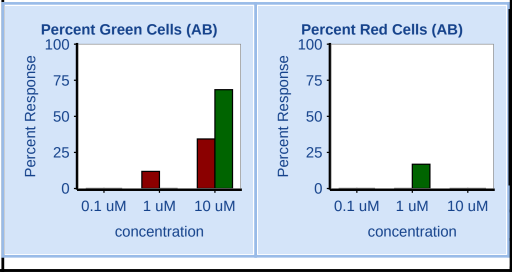

Average Response

Dose	Green Signal	Actual Pct Green Cells	Normalized Pct Green Cells	Red Signal	Actual Pct Red Cells	Normalized Pct Red Cells
0.1 uM	85.00	0.76	-19.87	98.40	0.08	-29.04
1 uM	94.93	0.78	-1.95	107.37	0.12	-0.34
10 uM	86.91	0.80	51.39	97.35	0.04	-39.23

Compound	Normalized Values (A and B Sets)	Responses

LY2603618

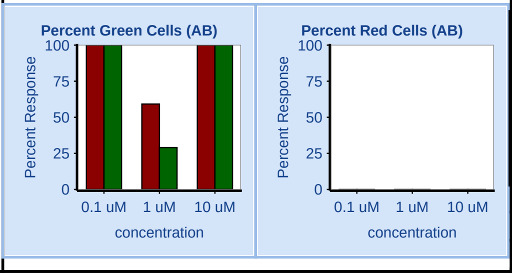

Average Response

Dose	Green Signal	Actual Pct Green Cells	Normalized Pct Green Cells	Red Signal	Actual Pct Red Cells	Normalized Pct Red Cells
0.1 uM	104.26	0.94	148.78	73.76	0.03	-43.10
1 uM	92.18	0.79	44.12	98.11	0.09	-17.18
10 uM	107.68	0.93	239.49	94.06	0.07	-23.43

Compound	Normalized Values (A and B Sets)	Responses

LY2608204

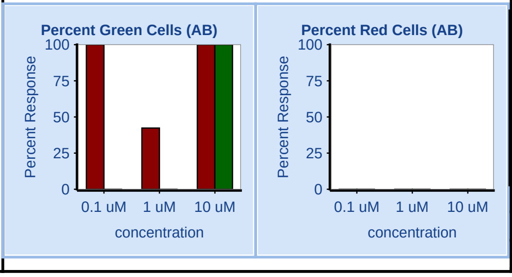

Average Response

Dose	Green Signal	Actual Pct Green Cells	Normalized Pct Green Cells	Red Signal	Actual Pct Red Cells	Normalized Pct Red Cells
0.1 uM	87.11	0.82	18.82	69.59	0.02	-45.36
1 uM	86.56	0.73	10.14	74.07	0.02	-45.09
10 uM	101.24	0.94	286.40	65.09	0.01	-55.91

Compound | Normalized Values (A and B Sets) | Responses

LY2784544

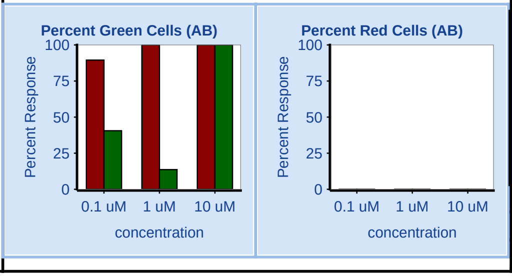

Average Response						
Dose	Green Signal	Actual Pct Green Cells	Normalized Pct Green Cells	Red Signal	Actual Pct Red Cells	Normalized Pct Red Cells
0.1 uM	89.66	0.86	65.03	81.21	0.03	-40.55
1 uM	98.62	0.83	59.81	78.35	0.03	-41.73
10 uM	123.90	0.93	219.53	100.08	0.08	-22.08

Compound | Normalized Values (A and B Sets) | Responses

LY2811376

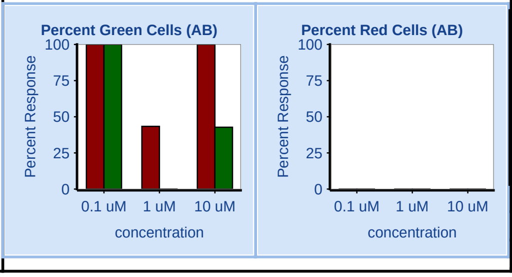

Average Response						
Dose	Green Signal	Actual Pct Green Cells	Normalized Pct Green Cells	Red Signal	Actual Pct Red Cells	Normalized Pct Red Cells
0.1 uM	103.38	0.94	127.84	85.44	0.03	-55.04
1 uM	84.05	0.70	-43.09	93.37	0.06	-31.28
10 uM	99.86	0.84	73.22	100.05	0.06	-29.31

Compound | Normalized Values (A and B Sets) | Responses

LY2835219

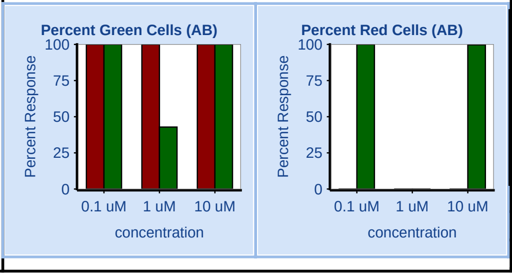

Average Response						
Dose	Green Signal	Actual Pct Green Cells	Normalized Pct Green Cells	Red Signal	Actual Pct Red Cells	Normalized Pct Red Cells
0.1 uM	106.83	0.97	295.29	106.18	0.19	37.32
1 uM	96.81	0.88	114.58	72.77	0.02	-56.83
10 uM	126.77	0.93	150.74	94.52	0.16	16.70

Compound | Normalized Values (A and B Sets) | Responses

LY2886721

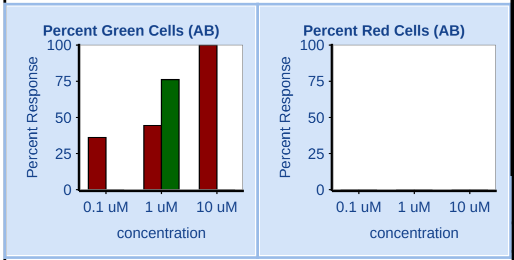

Average Response						
Dose	Green Signal	Actual Pct Green Cells	Normalized Pct Green Cells	Red Signal	Actual Pct Red Cells	Normalized Pct Red Cells
0.1 uM	75.99	0.69	-117.32	84.15	0.05	-28.13
1 uM	91.31	0.81	60.21	65.40	0.01	-51.87
10 uM	92.03	0.90	299.15	82.97	0.04	-40.01

Compound | Normalized Values (A and B Sets) | Responses

M344

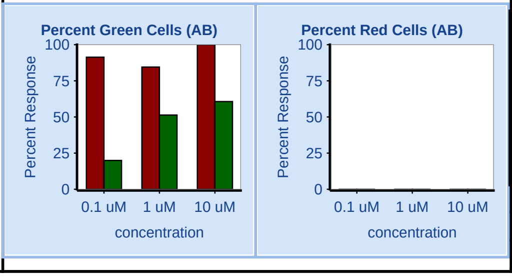

Average Response						
Dose	Green Signal	Actual Pct Green Cells	Normalized Pct Green Cells	Red Signal	Actual Pct Red Cells	Normalized Pct Red Cells
0.1 uM	94.78	0.85	55.65	77.56	0.04	-38.40
1 uM	96.89	0.84	67.93	89.56	0.08	-18.45
10 uM	121.82	0.94	502.48	82.74	0.03	-45.44

Compound	Normalized Values (A and B Sets)	Responses

Macitentan

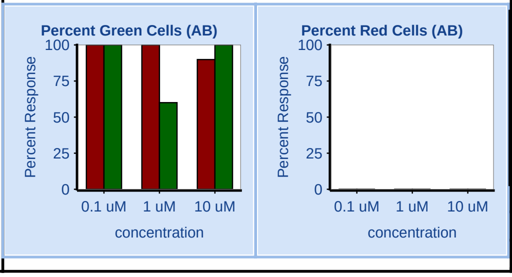

Average Response						
Dose	Green Signal	Actual Pct Green Cells	Normalized Pct Green Cells	Red Signal	Actual Pct Red Cells	Normalized Pct Red Cells
0.1 uM	104.34	0.92	122.90	75.02	0.02	-61.01
1 uM	101.49	0.90	197.61	80.70	0.05	-30.42
10 uM	98.20	0.93	114.16	68.91	0.01	-44.62

Compound	Normalized Values (A and B Sets)	Responses

Magnolol

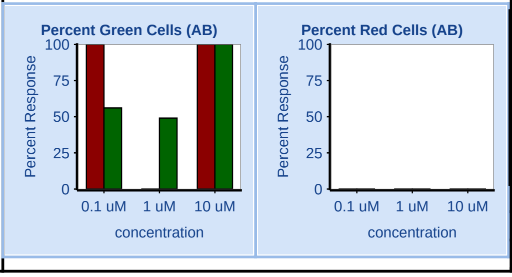

Average Response						
Dose	Green Signal	Actual Pct Green Cells	Normalized Pct Green Cells	Red Signal	Actual Pct Red Cells	Normalized Pct Red Cells
0.1 uM	99.07	0.91	115.86	78.12	0.02	-43.57
1 uM	82.44	0.74	22.76	94.20	0.05	-32.31
10 uM	94.81	0.92	196.82	65.11	0.01	-53.04

Compound	Normalized Values (A and B Sets)	Responses

Malotilate

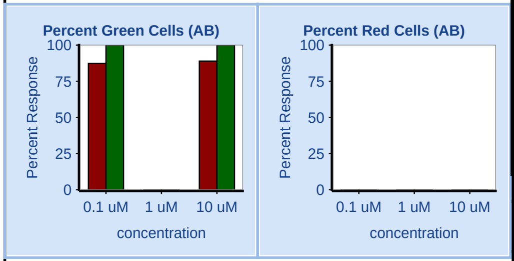

Average Response						
Dose	Green Signal	Actual Pct Green Cells	Normalized Pct Green Cells	Red Signal	Actual Pct Red Cells	Normalized Pct Red Cells
0.1 uM	106.25	0.91	98.62	106.54	0.12	-12.00
1 uM	74.19	0.56	-160.66	95.85	0.08	-20.59
10 uM	101.77	0.89	109.10	105.76	0.08	-21.49

Compound	Normalized Values (A and B Sets)	Responses

Manidipine

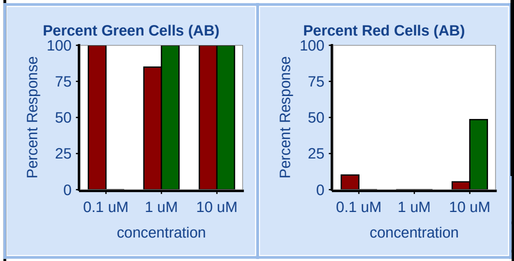

Average Response						
Dose	Green Signal	Actual Pct Green Cells	Normalized Pct Green Cells	Red Signal	Actual Pct Red Cells	Normalized Pct Red Cells
0.1 uM	89.92	0.72	-102.78	89.47	0.08	-19.48
1 uM	106.28	0.88	95.48	93.03	0.08	-20.58
10 uM	108.29	0.95	369.03	121.45	0.18	26.97

Compound	Normalized Values (A and B Sets)	Responses

Manidipine 2HCl

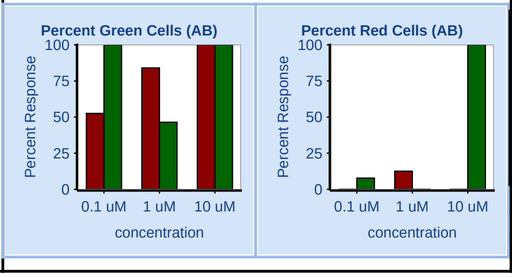

Average Response						
Dose	Green Signal	Actual Pct Green Cells	Normalized Pct Green Cells	Red Signal	Actual Pct Red Cells	Normalized Pct Red Cells
0.1 uM	100.56	0.89	96.92	98.95	0.12	0.69
1 uM	103.73	0.83	65.24	102.96	0.13	3.39
10 uM	122.53	0.97	489.34	131.56	0.25	61.40

Compound | Normalized Values (A and B Sets) | Responses

Maraviroc

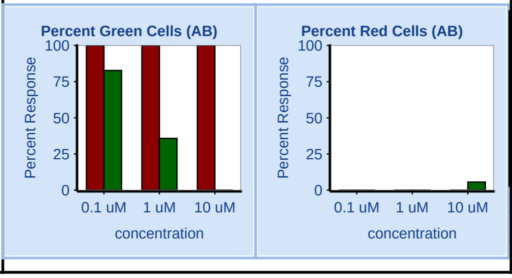

Average Response

Dose	Green Signal	Actual Pct Green Cells	Normalized Pct Green Cells	Red Signal	Actual Pct Red Cells	Normalized Pct Red Cells
0.1 uM	109.29	0.91	121.45	85.76	0.05	-48.67
1 uM	109.86	0.88	115.23	92.79	0.05	-33.09
10 uM	92.72	0.77	15.86	117.65	0.12	-0.82

Compound | Normalized Values (A and B Sets) | Responses

Marbofloxacin

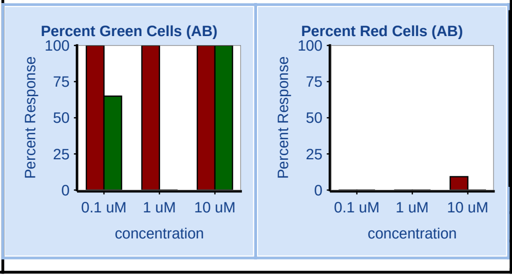

Average Response

Dose	Green Signal	Actual Pct Green Cells	Normalized Pct Green Cells	Red Signal	Actual Pct Red Cells	Normalized Pct Red Cells
0.1 uM	97.79	0.88	97.11	78.25	0.02	-66.07
1 uM	91.34	0.82	98.43	83.02	0.04	-26.98
10 uM	104.87	0.94	117.83	91.73	0.08	-13.52

Compound | Normalized Values (A and B Sets) | Responses

MC1568

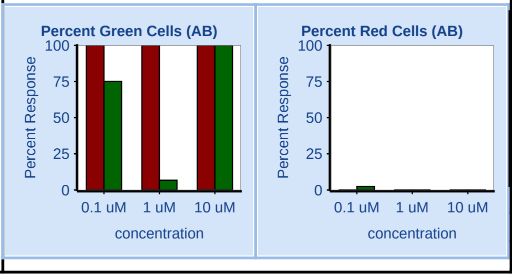

Average Response

Dose	Green Signal	Actual Pct Green Cells	Normalized Pct Green Cells	Red Signal	Actual Pct Red Cells	Normalized Pct Red Cells
0.1 uM	102.89	0.90	106.41	103.46	0.10	-15.84
1 uM	98.52	0.84	73.55	93.30	0.06	-29.66
10 uM	115.83	0.92	130.27	109.61	0.09	-15.83

Compound | Normalized Values (A and B Sets) | Responses

Mdivi-1

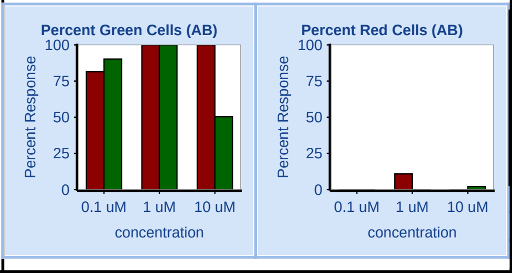

Average Response

Dose	Green Signal	Actual Pct Green Cells	Normalized Pct Green Cells	Red Signal	Actual Pct Red Cells	Normalized Pct Red Cells
0.1 uM	102.03	0.89	85.83	91.50	0.09	-19.81
1 uM	103.95	0.89	124.74	105.98	0.14	3.79
10 uM	101.82	0.85	119.10	100.64	0.12	-1.91

Compound | Normalized Values (A and B Sets) | Responses

ME0328

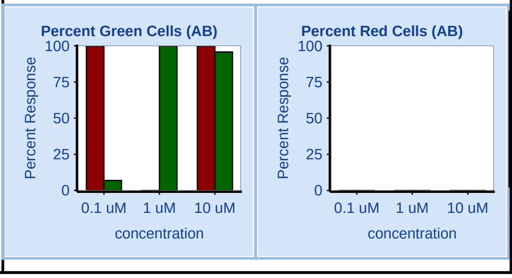

Average Response

Dose	Green Signal	Actual Pct Green Cells	Normalized Pct Green Cells	Red Signal	Actual Pct Red Cells	Normalized Pct Red Cells
0.1 uM	95.16	0.85	71.08	90.63	0.07	-30.73
1 uM	103.75	0.89	-88.96	90.32	0.09	-5.47
10 uM	98.39	0.92	100.32	89.29	0.07	-17.99

Compound | Normalized Values (A and B Sets) | Responses

Mecarbinate

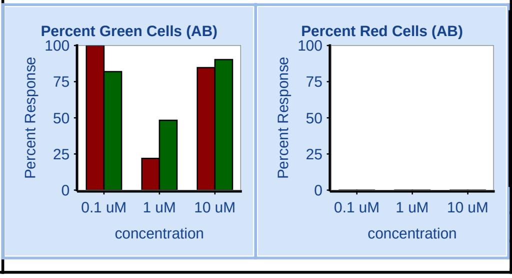

Average Response

Dose	Green Signal	Actual Pct Green Cells	Normalized Pct Green Cells	Red Signal	Actual Pct Red Cells	Normalized Pct Red Cells
0.1 uM	110.82	0.92	130.87	88.17	0.05	-47.38
1 uM	96.83	0.83	35.13	95.04	0.07	-26.76
10 uM	106.63	0.86	87.50	101.41	0.07	-22.63

Compound | Normalized Values (A and B Sets) | Responses

Medetomidine HCl

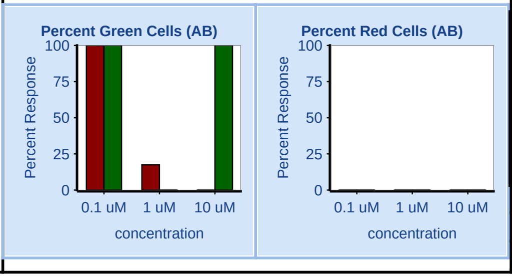

Average Response

Dose	Green Signal	Actual Pct Green Cells	Normalized Pct Green Cells	Red Signal	Actual Pct Red Cells	Normalized Pct Red Cells
0.1 uM	108.69	0.93	145.54	80.20	0.05	-37.78
1 uM	82.54	0.66	-47.51	79.29	0.04	-47.07
10 uM	96.64	0.83	26.24	85.58	0.04	-44.00

Compound | Normalized Values (A and B Sets) | Responses

Medroxyprogesterone acetate

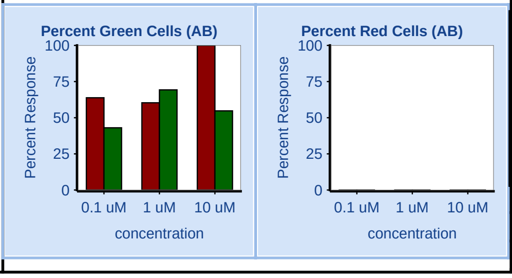

Average Response

Dose	Green Signal	Actual Pct Green Cells	Normalized Pct Green Cells	Red Signal	Actual Pct Red Cells	Normalized Pct Red Cells
0.1 uM	91.86	0.85	53.43	84.02	0.05	-32.79
1 uM	95.41	0.82	64.80	60.97	0.01	-51.06
10 uM	93.73	0.89	143.69	74.01	0.02	-50.76

Compound | Normalized Values (A and B Sets) | Responses

MEK162 (ARRY-162, ARRY-438162)

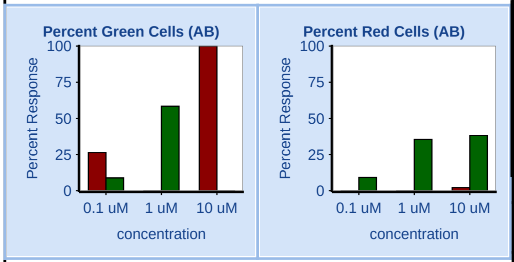

Average Response

Dose	Green Signal	Actual Pct Green Cells	Normalized Pct Green Cells	Red Signal	Actual Pct Red Cells	Normalized Pct Red Cells
0.1 uM	86.50	0.80	17.57	98.55	0.09	-21.56
1 uM	86.69	0.79	-492.83	83.58	0.09	6.48
10 uM	98.42	0.85	45.03	101.84	0.16	20.18

Compound | Normalized Values (A and B Sets) | Responses

Mepenzolate Bromide

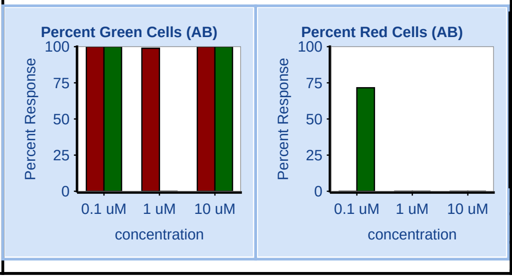

Average Response

Dose	Green Signal	Actual Pct Green Cells	Normalized Pct Green Cells	Red Signal	Actual Pct Red Cells	Normalized Pct Red Cells
0.1 uM	108.45	0.95	184.59	110.48	0.18	27.01
1 uM	86.17	0.70	-23.25	86.15	0.05	-41.17
10 uM	112.04	0.93	145.89	74.17	0.02	-55.07

Compound	Normalized Values (A and B Sets)	Responses
**Mepiroxol**	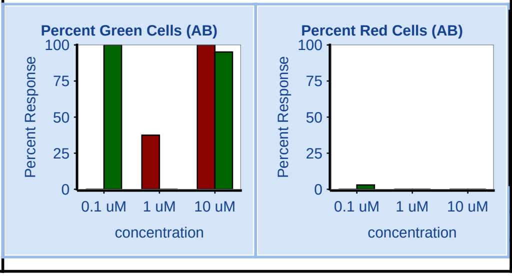	see table below

Average Response

Dose	Green Signal	Actual Pct Green Cells	Normalized Pct Green Cells	Red Signal	Actual Pct Red Cells	Normalized Pct Red Cells
0.1 uM	99.01	0.89	-36.98	86.19	0.07	-26.85
1 uM	88.59	0.75	18.09	82.14	0.05	-41.12
10 uM	105.87	0.90	136.32	94.01	0.08	-21.22

Compound	Normalized Values (A and B Sets)	Responses
**Meprednisone**	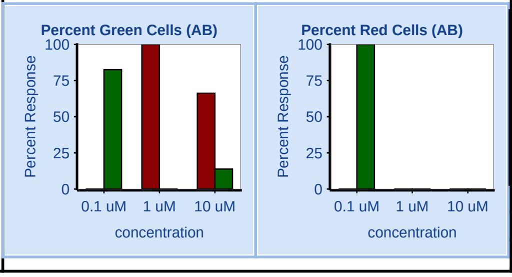	see table below

Average Response

Dose	Green Signal	Actual Pct Green Cells	Normalized Pct Green Cells	Red Signal	Actual Pct Red Cells	Normalized Pct Red Cells
0.1 uM	92.94	0.82	38.01	115.01	0.24	72.33
1 uM	97.68	0.87	211.00	72.26	0.03	-33.13
10 uM	91.86	0.85	40.08	66.27	0.02	-41.23

Compound	Normalized Values (A and B Sets)	Responses
**Meropenem**	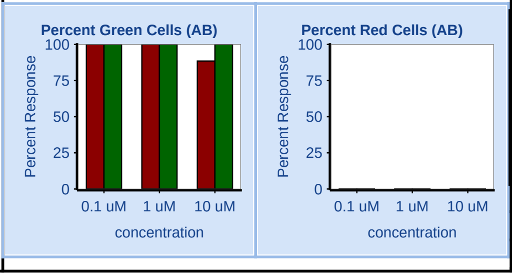	see table below

Average Response

Dose	Green Signal	Actual Pct Green Cells	Normalized Pct Green Cells	Red Signal	Actual Pct Red Cells	Normalized Pct Red Cells
0.1 uM	102.29	0.91	101.90	86.16	0.05	-49.83
1 uM	111.22	0.94	159.33	75.73	0.01	-50.94
10 uM	103.09	0.88	105.57	95.97	0.03	-39.93

Compound	Normalized Values (A and B Sets)	Responses
**Methazolamide**	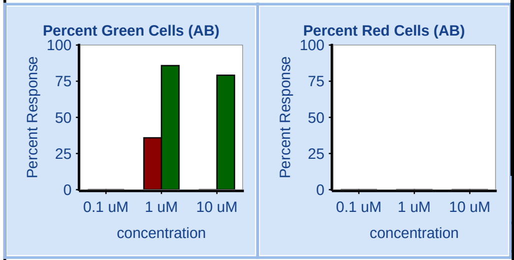	see table below

Average Response

Dose	Green Signal	Actual Pct Green Cells	Normalized Pct Green Cells	Red Signal	Actual Pct Red Cells	Normalized Pct Red Cells
0.1 uM	73.01	0.66	-252.26	70.08	0.02	-53.73
1 uM	95.16	0.80	60.84	85.56	0.07	-31.98
10 uM	90.45	0.81	23.19	80.05	0.02	-51.48

Compound	Normalized Values (A and B Sets)	Responses
**Methscopolamine**	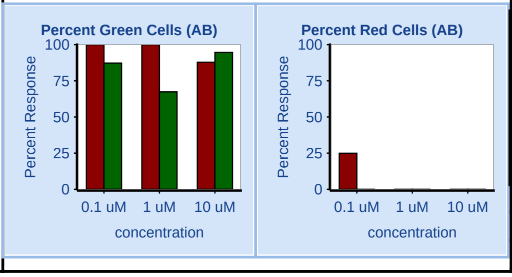	see table below

Average Response

Dose	Green Signal	Actual Pct Green Cells	Normalized Pct Green Cells	Red Signal	Actual Pct Red Cells	Normalized Pct Red Cells
0.1 uM	100.43	0.91	109.21	104.32	0.11	-23.84
1 uM	106.29	0.91	138.52	88.60	0.04	-40.68
10 uM	101.50	0.86	91.25	109.05	0.08	-19.90

Compound	Normalized Values (A and B Sets)	Responses

Methyclothiazide

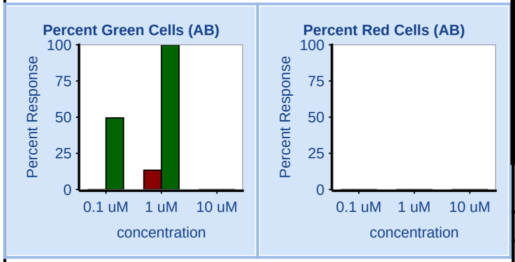

Average Response						
Dose	Green Signal	Actual Pct Green Cells	Normalized Pct Green Cells	Red Signal	Actual Pct Red Cells	Normalized Pct Red Cells
0.1 uM	84.93	0.77	-345.13	61.10	0.01	-58.54
1 uM	94.45	0.82	73.66	77.24	0.03	-51.80
10 uM	74.94	0.61	-112.12	77.91	0.04	-41.72

Compound	Normalized Values (A and B Sets)	Responses

Methyl-Hesperidin

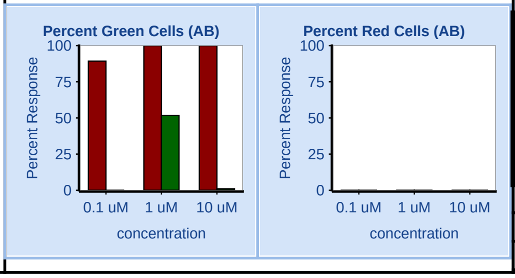

Average Response						
Dose	Green Signal	Actual Pct Green Cells	Normalized Pct Green Cells	Red Signal	Actual Pct Red Cells	Normalized Pct Red Cells
0.1 uM	87.56	0.82	23.30	49.65	0.01	-52.33
1 uM	93.87	0.86	80.00	84.04	0.04	-38.03
10 uM	97.90	0.91	382.24	82.01	0.04	-40.17

Compound	Normalized Values (A and B Sets)	Responses

Mezlocillin Sodium

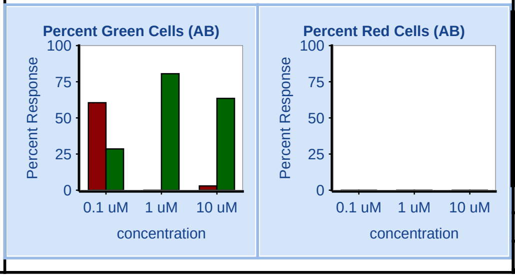

Average Response						
Dose	Green Signal	Actual Pct Green Cells	Normalized Pct Green Cells	Red Signal	Actual Pct Red Cells	Normalized Pct Red Cells
0.1 uM	91.19	0.83	44.53	77.28	0.02	-60.01
1 uM	96.66	0.86	-74.88	71.85	0.03	-37.21
10 uM	87.75	0.85	33.22	59.56	0.01	-46.27

Compound	Normalized Values (A and B Sets)	Responses

MG 149

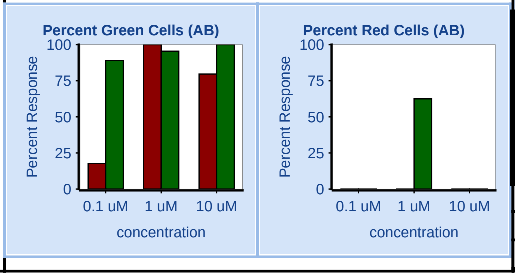

Average Response						
Dose	Green Signal	Actual Pct Green Cells	Normalized Pct Green Cells	Red Signal	Actual Pct Red Cells	Normalized Pct Red Cells
0.1 uM	91.51	0.84	53.39	98.12	0.10	-13.92
1 uM	102.86	0.91	169.55	102.76	0.14	26.30
10 uM	101.43	0.93	112.81	83.33	0.04	-34.22

Compound	Normalized Values (A and B Sets)	Responses

MG-132

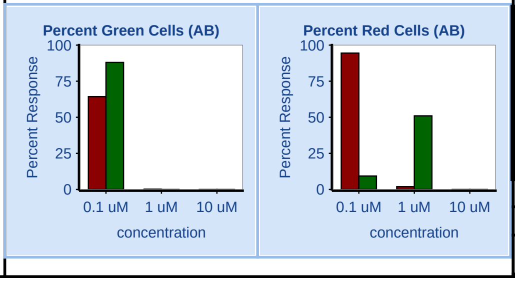

Average Response						
Dose	Green Signal	Actual Pct Green Cells	Normalized Pct Green Cells	Red Signal	Actual Pct Red Cells	Normalized Pct Red Cells
0.1 uM	99.21	0.87	76.17	123.60	0.24	51.90
1 uM	87.24	0.56	-89.44	119.32	0.18	26.48
10 uM	84.16	0.74		97.22	0.09	-16.07

Compound	Normalized Values (A and B Sets)	Responses						
					-613.11			

Compound	Normalized Values (A and B Sets)	Responses

MGCD-265

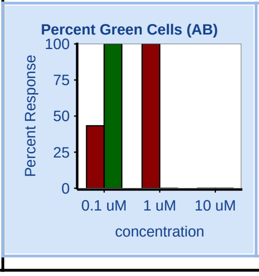

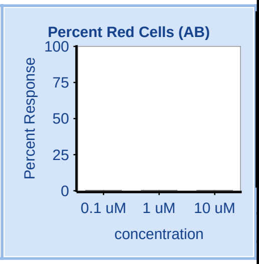

Average Response

Dose	Green Signal	Actual Pct Green Cells	Normalized Pct Green Cells	Red Signal	Actual Pct Red Cells	Normalized Pct Red Cells
0.1 uM	95.62	0.89	73.50	62.77	0.02	-65.24
1 uM	97.98	0.82	69.14	86.57	0.04	-38.97
10 uM	81.40	0.68	-36.67	87.94	0.03	-40.29

Compound	Normalized Values (A and B Sets)	Responses

Milciclib (PHA-848125)

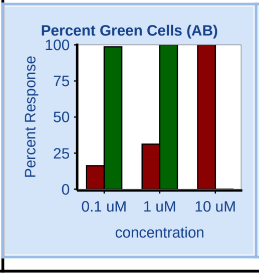

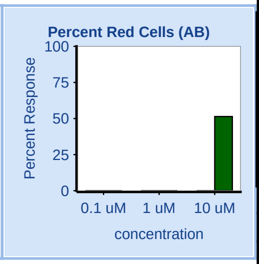

Average Response

Dose	Green Signal	Actual Pct Green Cells	Normalized Pct Green Cells	Red Signal	Actual Pct Red Cells	Normalized Pct Red Cells
0.1 uM	90.86	0.85	57.47	87.31	0.06	-28.29
1 uM	99.18	0.84	77.75	96.37	0.10	-11.97
10 uM	101.45	0.79	25.51	108.89	0.16	16.68

Compound	Normalized Values (A and B Sets)	Responses

Mirabegron

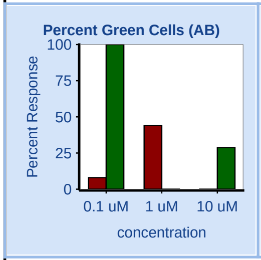

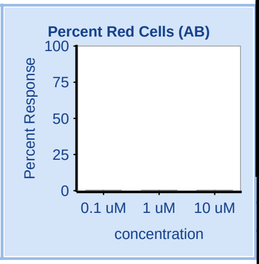

Average Response

Dose	Green Signal	Actual Pct Green Cells	Normalized Pct Green Cells	Red Signal	Actual Pct Red Cells	Normalized Pct Red Cells
0.1 uM	94.32	0.90	60.40	61.52	0.01	-56.33
1 uM	87.65	0.75	18.39	64.26	0.01	-65.04
10 uM	78.27	0.66	-154.97	81.09	0.05	-37.43

Compound	Normalized Values (A and B Sets)	Responses

Mizoribine

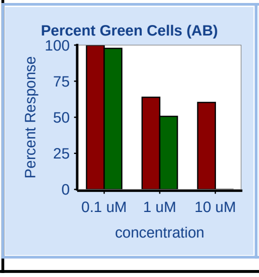

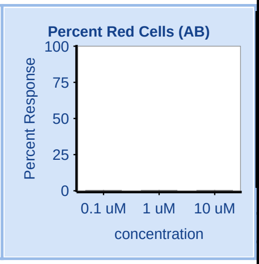

Average Response

Dose	Green Signal	Actual Pct Green Cells	Normalized Pct Green Cells	Red Signal	Actual Pct Red Cells	Normalized Pct Red Cells
0.1 uM	104.15	0.90	98.79	97.54	0.08	-33.00
1 uM	97.82	0.85	57.23	84.65	0.03	-43.60
10 uM	81.74	0.67	-49.03	102.04	0.05	-32.47

Compound	Normalized Values (A and B Sets)	Responses

MK-0752

Average Response

Dose	Green Signal	Actual Pct Green Cells		Red Signal	Actual Pct Red Cells	Normalized Pct Red Cells

Compound	Normalized Values (A and B Sets)	Responses

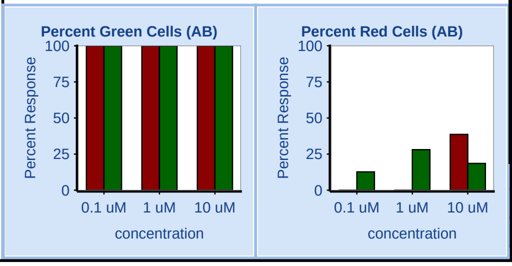

			Normalized Pct Green Cells			
0.1 uM	102.12	0.90	110.53	101.16	0.13	4.59
1 uM	110.64	0.92	114.18	98.96	0.13	0.97
10 uM	106.92	0.92	188.80	117.37	0.18	28.59

Compound	Normalized Values (A and B Sets)	Responses

MK-801 (Dizocilpine)

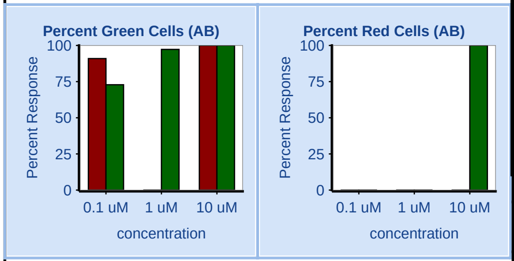

Average Response						
Dose	Green Signal	Actual Pct Green Cells	Normalized Pct Green Cells	Red Signal	Actual Pct Red Cells	Normalized Pct Red Cells
0.1 uM	94.29	0.87	81.90	81.70	0.03	-57.49
1 uM	88.35	0.83	-366.70	66.34	0.01	-42.54
10 uM	110.58	0.95	130.64	92.56	0.20	34.46

Compound	Normalized Values (A and B Sets)	Responses

MK-1775

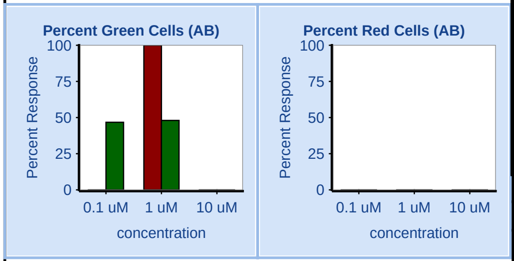

Average Response						
Dose	Green Signal	Actual Pct Green Cells	Normalized Pct Green Cells	Red Signal	Actual Pct Red Cells	Normalized Pct Red Cells
0.1 uM	92.11	0.82	19.14	77.65	0.02	-62.58
1 uM	115.02	0.87	90.79	84.82	0.07	-26.23
10 uM	68.49	0.45	-173.73	80.34	0.06	-27.79

Compound	Normalized Values (A and B Sets)	Responses

MK-2048

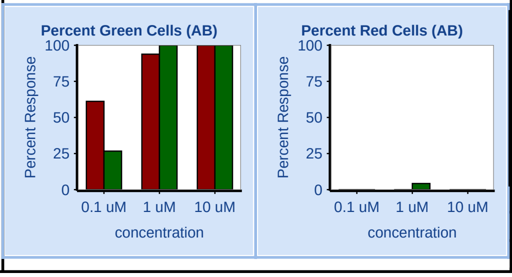

Average Response						
Dose	Green Signal	Actual Pct Green Cells	Normalized Pct Green Cells	Red Signal	Actual Pct Red Cells	Normalized Pct Red Cells
0.1 uM	95.13	0.84	43.93	80.42	0.06	-29.05
1 uM	106.18	0.89	97.52	96.36	0.09	-16.89
10 uM	104.67	0.95	381.03	93.92	0.07	-26.69

Compound	Normalized Values (A and B Sets)	Responses

MK-2461

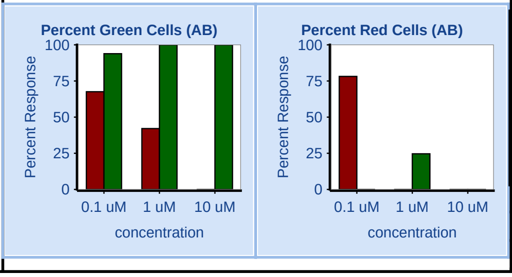

Average Response						
Dose	Green Signal	Actual Pct Green Cells	Normalized Pct Green Cells	Red Signal	Actual Pct Red Cells	Normalized Pct Red Cells
0.1 uM	94.29	0.87	80.72	104.92	0.16	14.41
1 uM	101.40	0.83	74.12	89.49	0.09	-13.73
10 uM	99.90	0.92	65.08	83.06	0.04	-41.64

Compound	Normalized Values (A and B Sets)	Responses

MK-2866 (GTx-024)

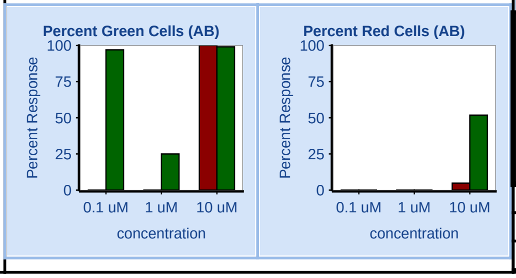

Average Response						
Dose	Green Signal	Actual Pct Green Cells	Normalized Pct Green Cells	Red Signal	Actual Pct Red Cells	Normalized Pct Red Cells
0.1 uM	96.56	0.82	-12.78	89.64	0.05	-46.97
1 uM	93.45	0.80	8.65	83.79	0.03	-42.46
10 uM	103.18	0.88	103.56	134.79	0.18	28.35

Compound	Normalized Values (A and B Sets)	Responses

MK-3207 HCl

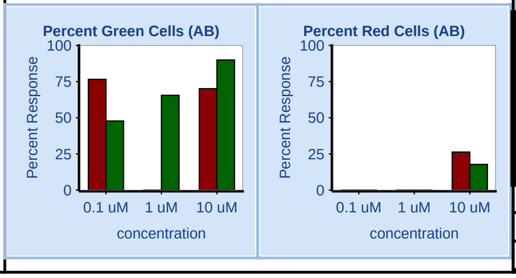

Average Response						
Dose	Green Signal	Actual Pct Green Cells	Normalized Pct Green Cells	Red Signal	Actual Pct Red Cells	Normalized Pct Red Cells
0.1 uM	95.79	0.85	62.16	76.58	0.02	-61.07
1 uM	101.84	0.80	-32.04	89.67	0.07	-24.10
10 uM	103.72	0.85	80.04	132.17	0.18	22.00

Compound	Normalized Values (A and B Sets)	Responses

MK-5108 (VX-689)

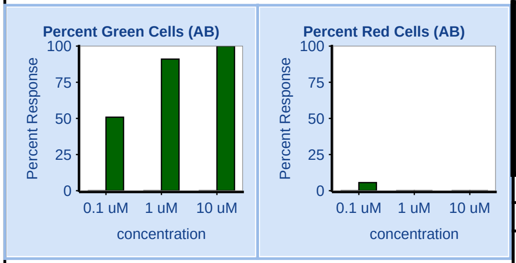

Average Response						
Dose	Green Signal	Actual Pct Green Cells	Normalized Pct Green Cells	Red Signal	Actual Pct Red Cells	Normalized Pct Red Cells
0.1 uM	87.21	0.79	1.07	96.82	0.09	-10.81
1 uM	90.47	0.72	23.63	95.24	0.09	-15.03
10 uM	103.56	0.87	-333.39	88.14	0.07	-25.02

Compound	Normalized Values (A and B Sets)	Responses

MK-8245

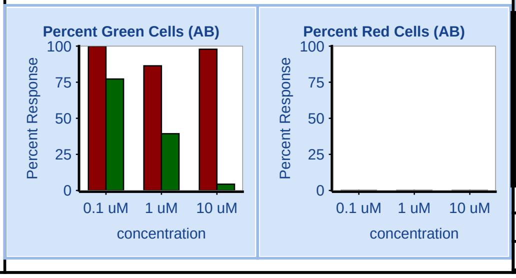

Average Response						
Dose	Green Signal	Actual Pct Green Cells	Normalized Pct Green Cells	Red Signal	Actual Pct Red Cells	Normalized Pct Red Cells
0.1 uM	103.50	0.90	113.58	82.18	0.02	-61.21
1 uM	95.10	0.84	62.86	93.47	0.04	-39.12
10 uM	97.05	0.81	51.12	85.22	0.03	-42.15

Compound	Normalized Values (A and B Sets)	Responses

MK-8776 (SCH 900776)

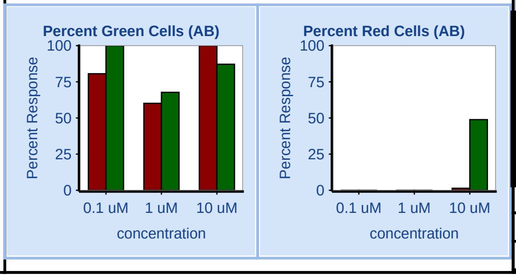

Average Response						
Dose	Green Signal	Actual Pct Green Cells	Normalized Pct Green Cells	Red Signal	Actual Pct Red Cells	Normalized Pct Red Cells
0.1 uM	99.92	0.91	117.78	90.17	0.07	-19.28
1 uM	98.25	0.82	63.94	83.98	0.05	-31.43
10 uM	113.70	0.92	312.10	112.19	0.18	25.12

Compound	Normalized Values (A and B Sets)	Responses

ML 141

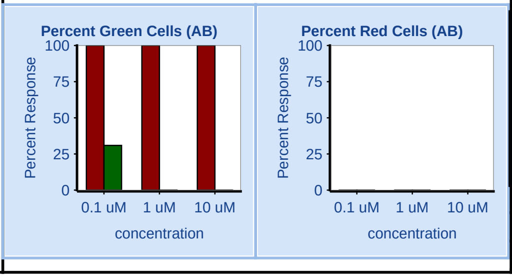

Average Response

Dose	Green Signal	Actual Pct Green Cells	Normalized Pct Green Cells	Red Signal	Actual Pct Red Cells	Normalized Pct Red Cells
0.1 uM	95.22	0.86	78.61	61.40	0.02	-62.25
1 uM	89.93	0.84	64.14	73.38	0.03	-31.33
10 uM	81.28	0.76	-36.19	57.15	0.01	-47.42

Compound	Normalized Values (A and B Sets)	Responses

ML130 (Nodinitib-1)

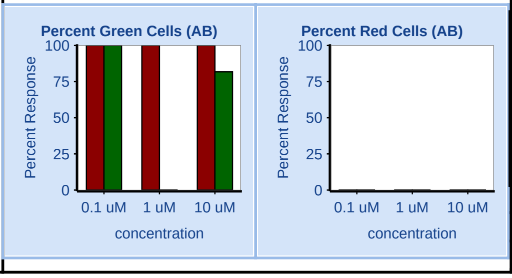

Average Response

Dose	Green Signal	Actual Pct Green Cells	Normalized Pct Green Cells	Red Signal	Actual Pct Red Cells	Normalized Pct Red Cells
0.1 uM	95.07	0.92	181.73	66.79	0.01	-57.98
1 uM	86.56	0.78	39.09	63.64	0.01	-62.64
10 uM	99.21	0.88	129.63	90.44	0.06	-33.98

Compound	Normalized Values (A and B Sets)	Responses

ML133 HCl

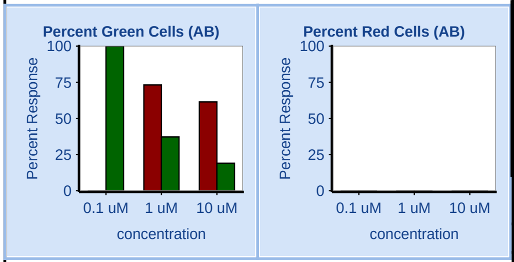

Average Response

Dose	Green Signal	Actual Pct Green Cells	Normalized Pct Green Cells	Red Signal	Actual Pct Red Cells	Normalized Pct Red Cells
0.1 uM	80.33	0.65	-127.75	91.24	0.08	-15.24
1 uM	95.38	0.81	55.18	79.32	0.04	-37.42
10 uM	93.72	0.87	40.22	79.57	0.03	-43.85

Compound	Normalized Values (A and B Sets)	Responses

ML161

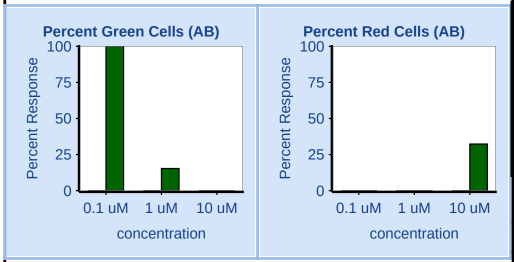

Average Response

Dose	Green Signal	Actual Pct Green Cells	Normalized Pct Green Cells	Red Signal	Actual Pct Red Cells	Normalized Pct Red Cells
0.1 uM	88.50	0.79	-366.09	69.46	0.02	-52.54
1 uM	82.56	0.70	-11.31	76.40	0.02	-55.94
10 uM	83.05	0.73	-14.86	94.34	0.09	-15.39

Compound	Normalized Values (A and B Sets)	Responses

MLN0905

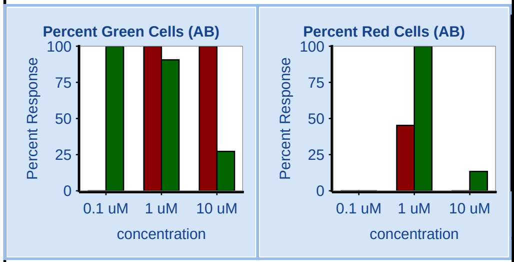

Average Response

Dose	Green Signal	Actual Pct Green Cells	Normalized Pct Green Cells	Red Signal	Actual Pct Red Cells	Normalized Pct Red Cells
0.1 uM	76.22	0.66	-917.66	87.66	0.07	-27.78
1 uM	108.58	0.87	104.99	124.84	0.28	74.83

Compound	Normalized Values (A and B Sets)	Responses

10 uM	106.79	0.85	133.72	110.04	0.11	-5.36

Compound	Normalized Values (A and B Sets)	Responses

MLN2238

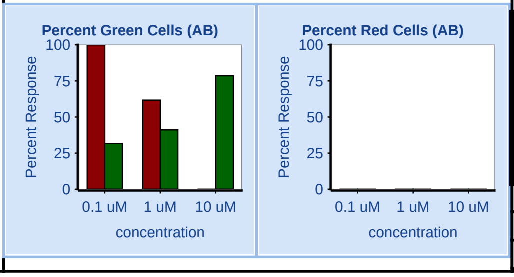

Average Response

Dose	Green Signal	Actual Pct Green Cells	Normalized Pct Green Cells	Red Signal	Actual Pct Red Cells	Normalized Pct Red Cells
0.1 uM	99.07	0.88	83.29	82.51	0.06	-27.75
1 uM	95.81	0.80	51.38	98.05	0.07	-23.95
10 uM	109.77	0.88	-3.98	97.58	0.07	-25.04

Compound	Normalized Values (A and B Sets)	Responses

MLN8054

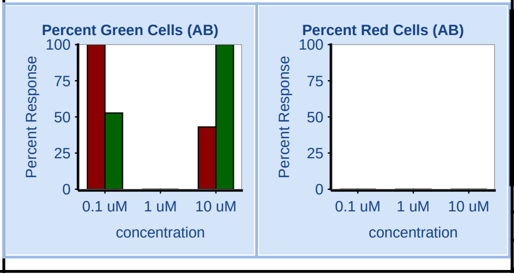

Average Response

Dose	Green Signal	Actual Pct Green Cells	Normalized Pct Green Cells	Red Signal	Actual Pct Red Cells	Normalized Pct Red Cells
0.1 uM	90.26	0.87	79.70	69.03	0.01	-66.38
1 uM	80.60	0.68	-68.44	84.66	0.03	-41.62
10 uM	110.99	0.87	100.63	85.24	0.03	-41.14

Compound	Normalized Values (A and B Sets)	Responses

MLN9708

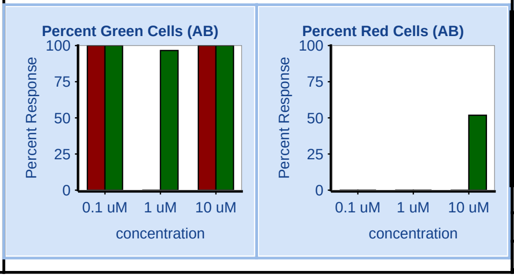

Average Response

Dose	Green Signal	Actual Pct Green Cells	Normalized Pct Green Cells	Red Signal	Actual Pct Red Cells	Normalized Pct Red Cells
0.1 uM	104.17	0.94	150.83	88.21	0.08	-19.40
1 uM	100.28	0.68	3.84	104.07	0.09	-13.80
10 uM	112.17	0.97	323.68	115.06	0.14	7.73

Compound	Normalized Values (A and B Sets)	Responses

MM-102

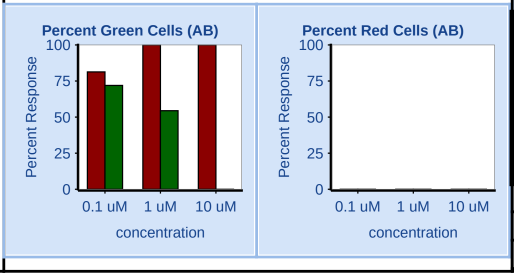

Average Response

Dose	Green Signal	Actual Pct Green Cells	Normalized Pct Green Cells	Red Signal	Actual Pct Red Cells	Normalized Pct Red Cells
0.1 uM	95.99	0.86	76.62	76.46	0.02	-66.25
1 uM	97.37	0.89	184.64	66.09	0.02	-38.14
10 uM	87.19	0.86	48.27	70.70	0.01	-44.64

Compound	Normalized Values (A and B Sets)	Responses

Moexipril HCl

Average Response

Dose	Green Signal	Actual Pct Green Cells	Normalized Pct Green Cells	Red Signal	Actual Pct Red Cells	Normalized Pct Red Cells

Compound	Normalized Values (A and B Sets)	Responses

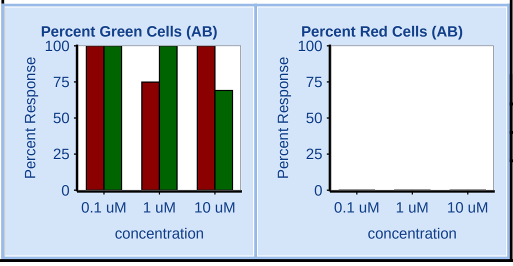

0.1 uM	108.33	0.92	109.90	98.91	0.09	-31.13
1 uM	104.88	0.89	89.77	107.83	0.11	-9.05
10 uM	101.52	0.87	93.78	105.38	0.07	-24.36

Compound	Normalized Values (A and B Sets)	Responses

Moguisteine

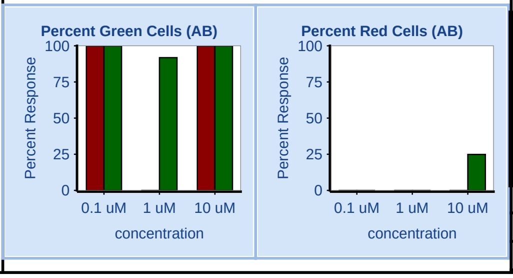

Average Response

Dose	Green Signal	Actual Pct Green Cells	Normalized Pct Green Cells	Red Signal	Actual Pct Red Cells	Normalized Pct Red Cells
0.1 uM	110.25	0.93	137.41	77.62	0.03	-59.57
1 uM	96.91	0.87	-57.48	71.60	0.03	-35.53
10 uM	104.55	0.94	125.95	92.21	0.10	-7.47

Compound	Normalized Values (A and B Sets)	Responses

Morin Hydrate

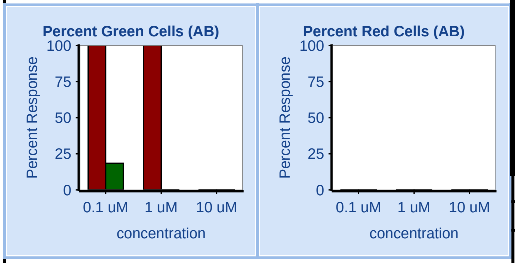

Average Response

Dose	Green Signal	Actual Pct Green Cells	Normalized Pct Green Cells	Red Signal	Actual Pct Red Cells	Normalized Pct Red Cells
0.1 uM	88.34	0.86	61.35	67.06	0.01	-49.45
1 uM	91.32	0.79	35.84	70.97	0.02	-47.68
10 uM	83.92	0.79	-129.57	79.57	0.03	-45.35

Compound	Normalized Values (A and B Sets)	Responses

Motesanib Diphosphate (AMG-706)

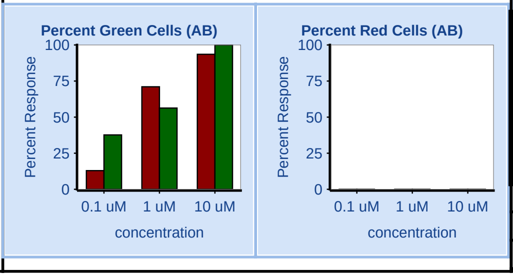

Average Response

Dose	Green Signal	Actual Pct Green Cells	Normalized Pct Green Cells	Red Signal	Actual Pct Red Cells	Normalized Pct Red Cells
0.1 uM	87.53	0.82	25.31	89.71	0.04	-50.45
1 uM	95.27	0.85	63.66	78.03	0.02	-49.03
10 uM	103.20	0.91	124.56	75.78	0.02	-45.24

Compound	Normalized Values (A and B Sets)	Responses

Moxalactam Disodium

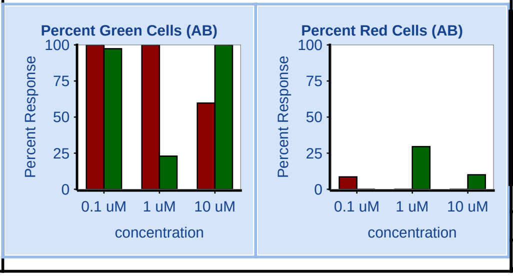

Average Response

Dose	Green Signal	Actual Pct Green Cells	Normalized Pct Green Cells	Red Signal	Actual Pct Red Cells	Normalized Pct Red Cells
0.1 uM	103.19	0.92	202.78	99.98	0.11	-10.42
1 uM	102.60	0.84	84.60	94.12	0.12	-10.13
10 uM	103.04	0.88	85.05	98.54	0.10	-12.93

Compound	Normalized Values (A and B Sets)	Responses

Mozavaptan

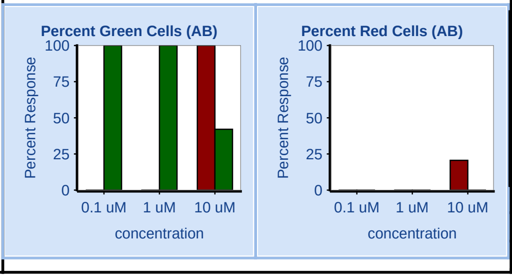

Average Response

Dose	Green Signal	Actual Pct Green Cells	Normalized Pct Green Cells	Red Signal	Actual Pct Red Cells	Normalized Pct Red Cells
0.1 uM	91.04	0.87	-75.11	75.84	0.01	-56.78
1 uM	92.59	0.78	45.34	67.76	0.01	-62.13
10 uM	105.73	0.86	135.79	102.54	0.12	0.98

Compound	Normalized Values (A and B Sets)	Responses

MPEP

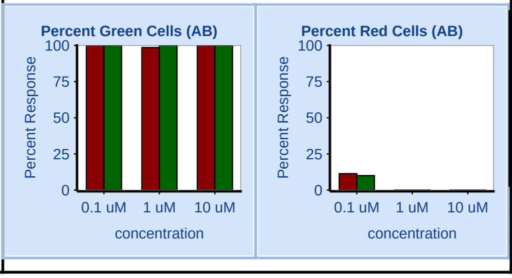

Average Response

Dose	Green Signal	Actual Pct Green Cells	Normalized Pct Green Cells	Red Signal	Actual Pct Red Cells	Normalized Pct Red Cells
0.1 uM	108.88	0.94	142.32	103.97	0.14	10.67
1 uM	107.36	0.89	100.07	86.88	0.05	-34.03
10 uM	106.03	0.95	326.19	85.98	0.04	-37.81

Compound	Normalized Values (A and B Sets)	Responses

MRS 2578

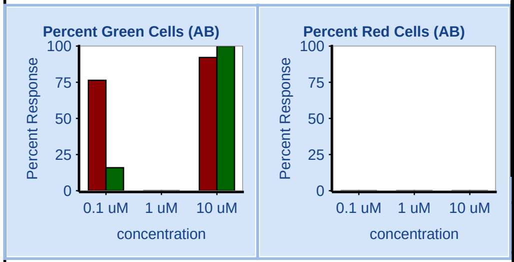

Average Response

Dose	Green Signal	Actual Pct Green Cells	Normalized Pct Green Cells	Red Signal	Actual Pct Red Cells	Normalized Pct Red Cells
0.1 uM	94.11	0.83	46.15	75.17	0.02	-62.72
1 uM	84.31	0.77	-164.95	62.21	0.02	-36.23
10 uM	108.45	0.94	121.82	70.31	0.03	-39.42

Compound	Normalized Values (A and B Sets)	Responses

MRT-10

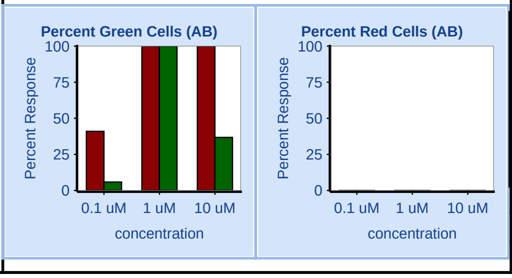

Average Response

Dose	Green Signal	Actual Pct Green Cells	Normalized Pct Green Cells	Red Signal	Actual Pct Red Cells	Normalized Pct Red Cells
0.1 uM	88.68	0.80	23.42	74.20	0.02	-63.96
1 uM	107.27	0.94	246.85	69.47	0.03	-33.86
10 uM	91.47	0.90	89.70	62.99	0.01	-46.33

Compound	Normalized Values (A and B Sets)	Responses

Mubritinib (TAK 165)

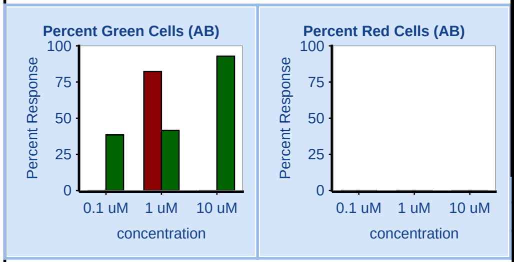

Average Response

Dose	Green Signal	Actual Pct Green Cells	Normalized Pct Green Cells	Red Signal	Actual Pct Red Cells	Normalized Pct Red Cells
0.1 uM	85.92	0.78	-16.51	69.15	0.03	-42.57
1 uM	96.26	0.83	61.91	73.05	0.02	-46.97
10 uM	91.17	0.80	-675.81	82.21	0.04	-41.31

Compound	Normalized Values (A and B Sets)	Responses

Mycophenolate Mofetil

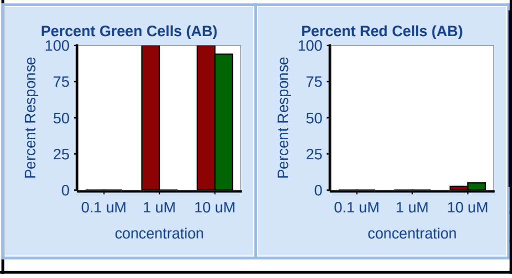

Average Response

Dose	Green Signal	Actual Pct Green Cells	Normalized Pct Green Cells	Red Signal	Actual Pct Red Cells	Normalized Pct Red Cells
0.1 uM	84.29	0.72	-34.69	76.77	0.03	-60.31
1 uM	89.68	0.74	16.15	76.52	0.02	-50.41
10 uM	116.00	0.89	104.14	121.94	0.13	3.81

Compound	Normalized Values (A and B Sets)	Responses

Myricetin

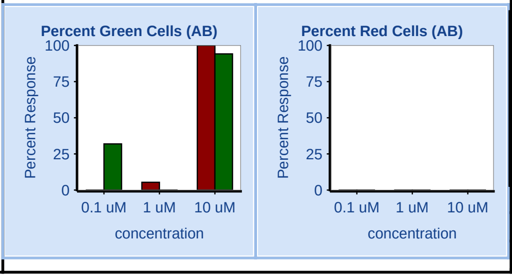

Average Response

Dose	Green Signal	Actual Pct Green Cells	Normalized Pct Green Cells	Red Signal	Actual Pct Red Cells	Normalized Pct Red Cells
0.1 uM	82.57	0.75	-38.86	68.68	0.02	-44.89
1 uM	84.19	0.70	-0.28	75.29	0.03	-42.57
10 uM	103.80	0.94	439.15	83.82	0.03	-42.50

Compound	Normalized Values (A and B Sets)	Responses

Myricitrin

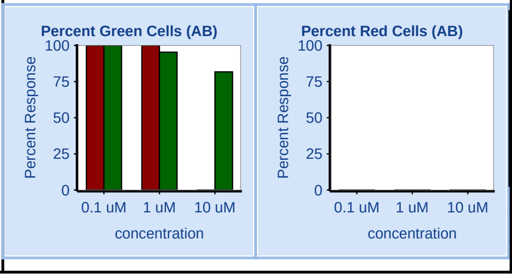

Average Response

Dose	Green Signal	Actual Pct Green Cells	Normalized Pct Green Cells	Red Signal	Actual Pct Red Cells	Normalized Pct Red Cells
0.1 uM	104.01	0.94	144.66	67.74	0.03	-42.90
1 uM	103.78	0.91	104.03	77.42	0.03	-44.28
10 uM	91.02	0.88	-1.48	75.11	0.03	-44.71

Compound	Normalized Values (A and B Sets)	Responses

n/a

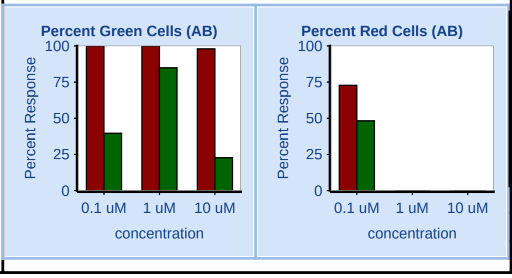

Average Response

Dose	Green Signal	Actual Pct Green Cells	Normalized Pct Green Cells	Red Signal	Actual Pct Red Cells	Normalized Pct Red Cells
0.1 uM	108.00	0.86	81.49	139.96	0.26	60.46
1 uM	86.45	0.64	-38.42	127.71	0.20	34.00
10 uM	92.16	0.80	46.26	119.73	0.13	2.02

Compound	Normalized Values (A and B Sets)	Responses

Nafamostat Mesylate

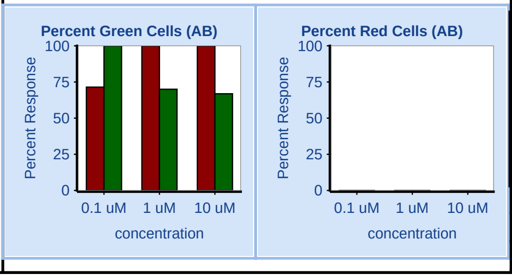

Average Response

Dose	Green Signal	Actual Pct Green Cells	Normalized Pct Green Cells	Red Signal	Actual Pct Red Cells	Normalized Pct Red Cells
0.1 uM	102.26	0.91	96.77	86.15	0.03	-57.57
1 uM	102.51	0.88	101.36	78.14	0.02	-48.95
10 uM	103.19	0.88	97.00	102.11	0.06	-27.83

Compound	Normalized Values (A and B Sets)	Responses

Nafcillin Sodium

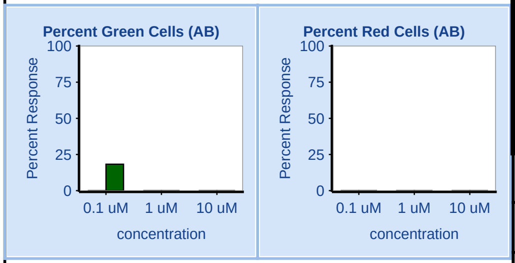

Average Response						
Dose	Green Signal	Actual Pct Green Cells	Normalized Pct Green Cells	Red Signal	Actual Pct Red Cells	Normalized Pct Red Cells
0.1 uM	80.40	0.76	-288.28	80.22	0.04	-42.97
1 uM	70.69	0.51	-157.91	74.68	0.04	-48.87
10 uM	79.38	0.66	-115.81	78.44	0.03	-49.59

Compound	Normalized Values (A and B Sets)	Responses

Naltrexone HCl

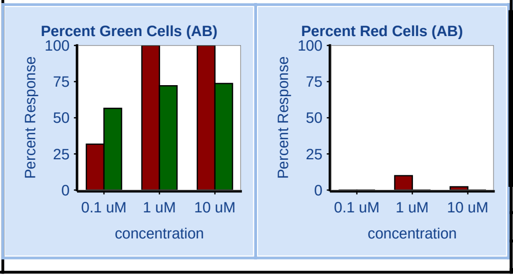

Average Response						
Dose	Green Signal	Actual Pct Green Cells	Normalized Pct Green Cells	Red Signal	Actual Pct Red Cells	Normalized Pct Red Cells
0.1 uM	92.62	0.84	44.14	94.83	0.06	-40.67
1 uM	109.90	0.88	98.16	102.02	0.09	-17.96
10 uM	114.33	0.91	114.07	111.20	0.10	-13.89

Compound	Normalized Values (A and B Sets)	Responses

Nanchangmycin

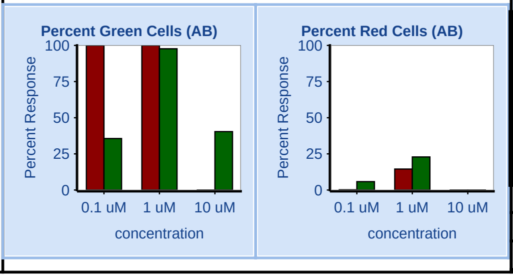

Average Response						
Dose	Green Signal	Actual Pct Green Cells	Normalized Pct Green Cells	Red Signal	Actual Pct Red Cells	Normalized Pct Red Cells
0.1 uM	106.83	0.88	104.31	108.59	0.14	3.00
1 uM	119.35	0.90	112.39	120.77	0.17	18.69
10 uM	84.36	0.65	-40.70	77.79	0.04	-36.18

Compound	Normalized Values (A and B Sets)	Responses

Naratriptan

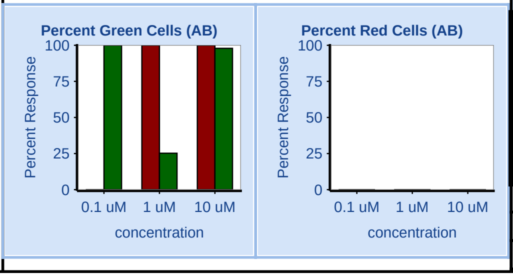

Average Response						
Dose	Green Signal	Actual Pct Green Cells	Normalized Pct Green Cells	Red Signal	Actual Pct Red Cells	Normalized Pct Red Cells
0.1 uM	101.26	0.86	27.10	90.67	0.07	-30.65
1 uM	105.17	0.85	85.59	86.99	0.06	-31.64
10 uM	106.15	0.88	101.37	112.33	0.10	-11.47

Compound	Normalized Values (A and B Sets)	Responses

Naringin

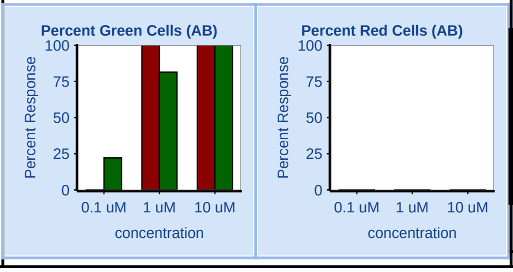

Average Response						
Dose	Green Signal	Actual Pct Green Cells	Normalized Pct Green Cells	Red Signal	Actual Pct Red Cells	Normalized Pct Red Cells
0.1 uM	74.86	0.63	-156.86	85.96	0.07	-22.65

Compound	Normalized Values (A and B Sets)	Responses

1 uM	105.55	0.91	105.90	79.32	0.04	-38.41
10 uM	105.75	0.93	258.93	94.90	0.09	-13.44

Compound	Normalized Values (A and B Sets)	Responses

Naringin Dihydrochalcone

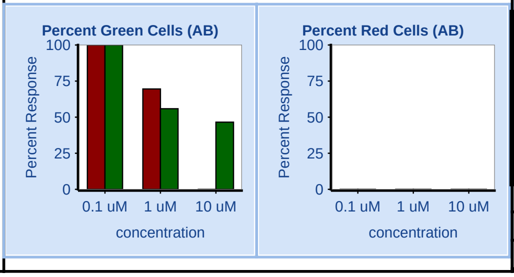

Average Response

Dose	Green Signal	Actual Pct Green Cells	Normalized Pct Green Cells	Red Signal	Actual Pct Red Cells	Normalized Pct Red Cells
0.1 uM	95.37	0.91	118.16	72.92	0.02	-47.22
1 uM	95.43	0.82	62.69	72.87	0.02	-46.31
10 uM	94.28	0.87	10.38	84.59	0.05	-34.44

Compound	Normalized Values (A and B Sets)	Responses

Natamycin

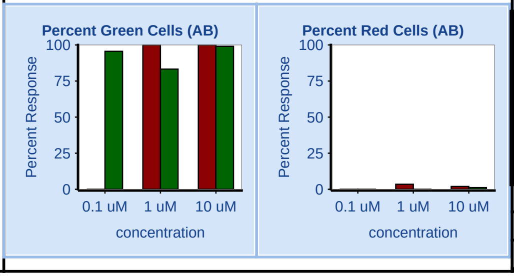

Average Response

Dose	Green Signal	Actual Pct Green Cells	Normalized Pct Green Cells	Red Signal	Actual Pct Red Cells	Normalized Pct Red Cells
0.1 uM	90.72	0.79	-51.43	104.23	0.09	-24.62
1 uM	111.16	0.91	129.88	97.58	0.08	-21.45
10 uM	112.88	0.92	122.36	124.39	0.13	1.57

Compound	Normalized Values (A and B Sets)	Responses

Nebivolol

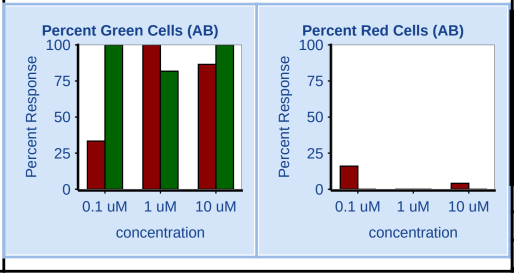

Average Response

Dose	Green Signal	Actual Pct Green Cells	Normalized Pct Green Cells	Red Signal	Actual Pct Red Cells	Normalized Pct Red Cells
0.1 uM	97.46	0.89	72.34	115.99	0.14	-1.73
1 uM	108.51	0.90	121.32	95.68	0.06	-29.99
10 uM	108.17	0.89	108.93	114.51	0.09	-17.83

Compound	Normalized Values (A and B Sets)	Responses

Necrostatin-1

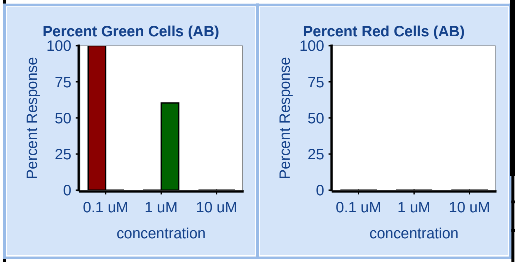

Average Response

Dose	Green Signal	Actual Pct Green Cells	Normalized Pct Green Cells	Red Signal	Actual Pct Red Cells	Normalized Pct Red Cells
0.1 uM	89.71	0.80	25.76	73.84	0.02	-61.85
1 uM	90.48	0.87	2.29	75.30	0.02	-39.87
10 uM	65.91	0.52	-283.06	60.38	0.01	-46.57

Compound	Normalized Values (A and B Sets)	Responses

Nefiracetam

Average Response

Dose	Green Signal			Red Signal	Actual Pct Red Cells	

Compound	Normalized Values (A and B Sets)	Responses

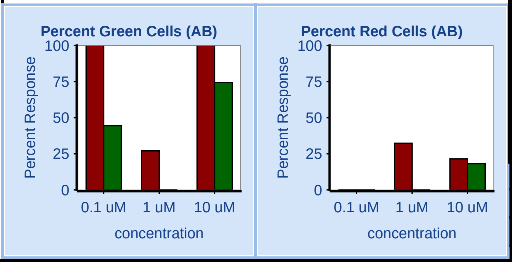

		Actual Pct Green Cells	Normalized Pct Green Cells			Normalized Pct Red Cells
0.1 uM	98.81	0.86	74.65	103.19	0.10	-19.67
1 uM	94.68	0.76	-7.37	114.53	0.14	5.74
10 uM	105.71	0.86	87.92	128.67	0.17	19.83

Compound	Normalized Values (A and B Sets)	Responses

Nelarabine

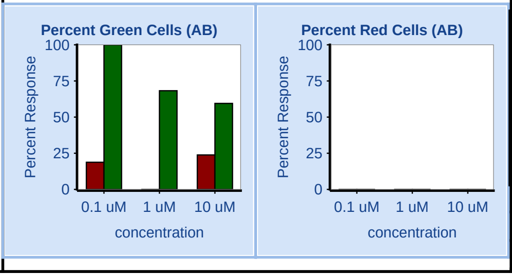

Average Response

Dose	Green Signal	Actual Pct Green Cells	Normalized Pct Green Cells	Red Signal	Actual Pct Red Cells	Normalized Pct Red Cells
0.1 uM	93.85	0.86	73.16	84.62	0.03	-55.44
1 uM	92.28	0.86	-47.65	79.83	0.04	-31.57
10 uM	89.98	0.86	41.65	70.42	0.03	-38.52

Compound	Normalized Values (A and B Sets)	Responses

Neohesperidin

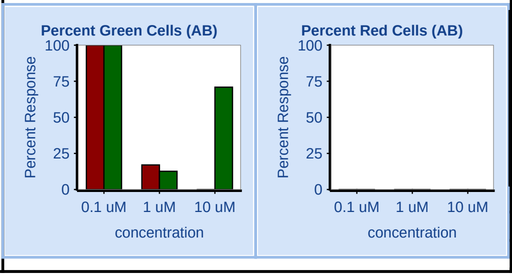

Average Response

Dose	Green Signal	Actual Pct Green Cells	Normalized Pct Green Cells	Red Signal	Actual Pct Red Cells	Normalized Pct Red Cells
0.1 uM	97.25	0.91	116.07	78.64	0.03	-41.28
1 uM	84.43	0.73	14.83	88.44	0.05	-35.76
10 uM	91.85	0.87	-54.12	82.16	0.04	-38.93

Compound	Normalized Values (A and B Sets)	Responses

Neohesperidin Dihydrochalcone (Nhdc)

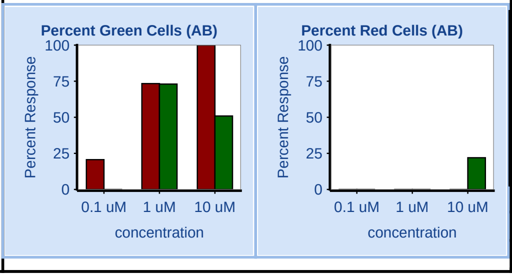

Average Response

Dose	Green Signal	Actual Pct Green Cells	Normalized Pct Green Cells	Red Signal	Actual Pct Red Cells	Normalized Pct Red Cells
0.1 uM	82.00	0.76	-39.41	83.99	0.04	-37.83
1 uM	94.15	0.84	73.19	67.18	0.01	-51.15
10 uM	95.26	0.91	319.49	104.92	0.11	-8.37

Compound	Normalized Values (A and B Sets)	Responses

Nepafenac

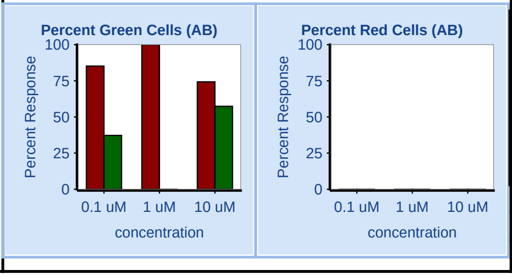

Average Response

Dose	Green Signal	Actual Pct Green Cells	Normalized Pct Green Cells	Red Signal	Actual Pct Red Cells	Normalized Pct Red Cells
0.1 uM	92.96	0.84	61.21	104.83	0.09	-30.50
1 uM	93.52	0.82	70.62	81.44	0.02	-46.51
10 uM	94.67	0.83	65.84	98.61	0.06	-27.23

Compound	Normalized Values (A and B Sets)	Responses

Nepicastat (SYN-117) HCl

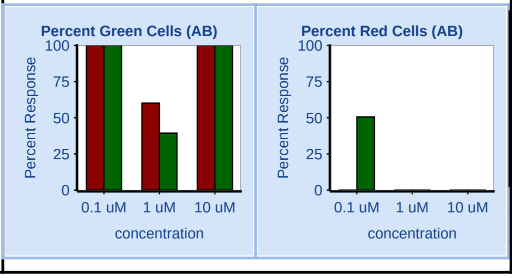

Average Response						
Dose	Green Signal	Actual Pct Green Cells	Normalized Pct Green Cells	Red Signal	Actual Pct Red Cells	Normalized Pct Red Cells
0.1 uM	103.75	0.91	115.88	106.73	0.15	18.43
1 uM	94.97	0.80	49.83	90.59	0.08	-19.36
10 uM	104.83	0.95	381.96	95.84	0.07	-27.26

Compound	Normalized Values (A and B Sets)	Responses

Neratinib (HKI-272)

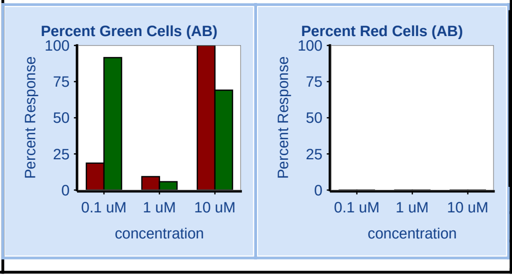

Average Response						
Dose	Green Signal	Actual Pct Green Cells	Normalized Pct Green Cells	Red Signal	Actual Pct Red Cells	Normalized Pct Red Cells
0.1 uM	91.87	0.85	55.12	72.90	0.02	-46.56
1 uM	82.37	0.72	7.53	77.50	0.02	-47.15
10 uM	96.29	0.93	403.46	84.42	0.03	-45.35

Compound	Normalized Values (A and B Sets)	Responses

Nexturastat A

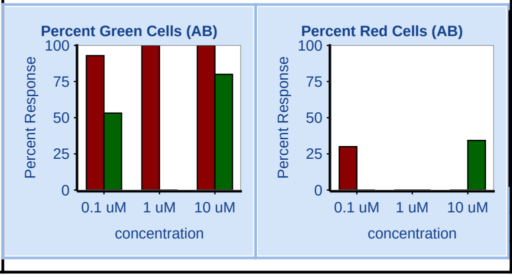

Average Response						
Dose	Green Signal	Actual Pct Green Cells	Normalized Pct Green Cells	Red Signal	Actual Pct Red Cells	Normalized Pct Red Cells
0.1 uM	96.55	0.86	73.07	108.03	0.14	11.40
1 uM	98.19	0.85	212.80	86.52	0.07	-14.48
10 uM	107.10	0.93	110.62	107.77	0.15	16.34

Compound	Normalized Values (A and B Sets)	Responses

NFkB Activation Inhibitor III

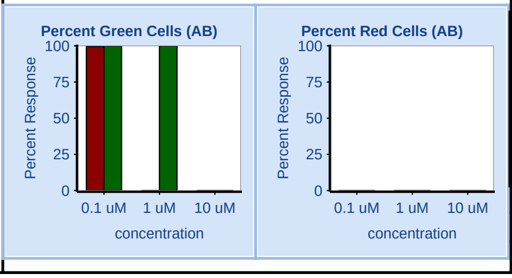

Average Response						
Dose	Green Signal	Actual Pct Green Cells	Normalized Pct Green Cells	Red Signal	Actual Pct Red Cells	Normalized Pct Red Cells
0.1 uM	99.73	0.90	106.88	88.91	0.05	-43.11
1 uM	96.26	0.90	10.05	74.92	0.03	-34.38
10 uM	83.93	0.76	-45.44	57.10	0.01	-45.37

Compound	Normalized Values (A and B Sets)	Responses

Nicaraven

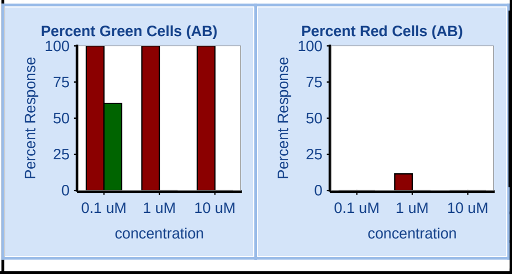

Average Response						
Dose	Green Signal	Actual Pct Green Cells	Normalized Pct Green Cells	Red Signal	Actual Pct Red Cells	Normalized Pct Red Cells
0.1 uM	94.56	0.89	187.68	94.25	0.10	-15.24
1 uM	90.59	0.66	-55.24	104.85	0.13	-0.20
10 uM	93.32	0.79	82.01	95.85	0.08	-18.98

Compound | Normalized Values (A and B Sets) | Responses

Nicotine Ditartrate

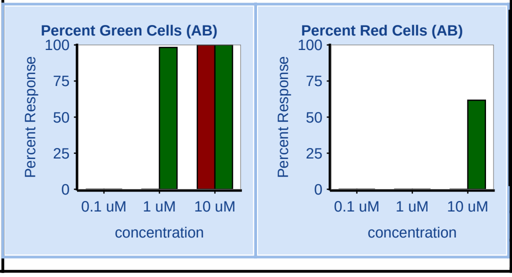

Average Response						
Dose	Green Signal	Actual Pct Green Cells	Normalized Pct Green Cells	Red Signal	Actual Pct Red Cells	Normalized Pct Red Cells
0.1 uM	83.56	0.79	-82.29	86.56	0.06	-31.31
1 uM	96.30	0.78	47.92	88.84	0.07	-32.09
10 uM	112.38	0.92	143.61	104.25	0.14	11.25

Compound | Normalized Values (A and B Sets) | Responses

Nintedanib (BIBF 1120)

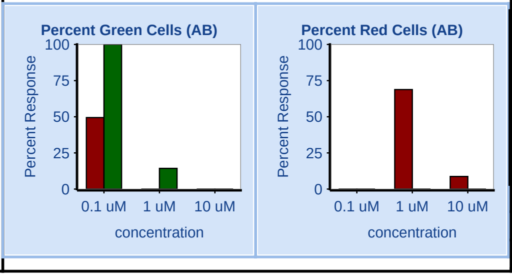

Average Response						
Dose	Green Signal	Actual Pct Green Cells	Normalized Pct Green Cells	Red Signal	Actual Pct Red Cells	Normalized Pct Red Cells
0.1 uM	95.42	0.89	97.10	94.66	0.07	-32.02
1 uM	94.90	0.84	-24.47	98.63	0.16	8.24
10 uM	80.63	0.75	-53.01	87.15	0.09	-9.59

Compound | Normalized Values (A and B Sets) | Responses

Nitarsone

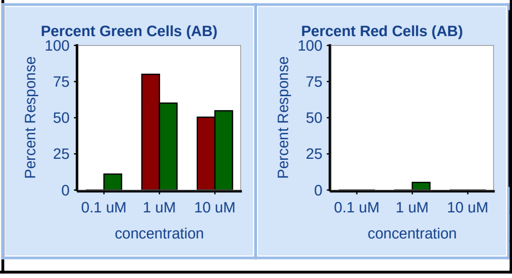

Average Response						
Dose	Green Signal	Actual Pct Green Cells	Normalized Pct Green Cells	Red Signal	Actual Pct Red Cells	Normalized Pct Red Cells
0.1 uM	90.29	0.82	-32.02	71.87	0.04	-44.69
1 uM	99.39	0.82	70.07	88.72	0.08	-26.34
10 uM	94.56	0.81	52.60	78.20	0.02	-52.73

Compound | Normalized Values (A and B Sets) | Responses

NLG919

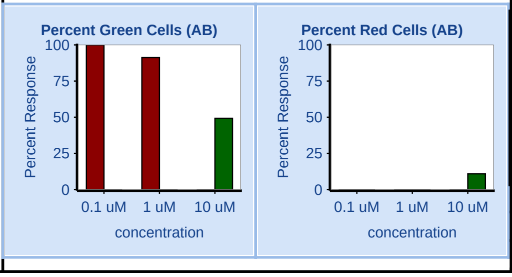

Average Response						
Dose	Green Signal	Actual Pct Green Cells	Normalized Pct Green Cells	Red Signal	Actual Pct Red Cells	Normalized Pct Red Cells
0.1 uM	94.07	0.84	120.48	81.80	0.04	-41.86
1 uM	97.01	0.78	41.95	97.66	0.10	-14.57
10 uM	90.24	0.79	14.11	88.48	0.08	-24.28

Compound | Normalized Values (A and B Sets) | Responses

Nocodazole

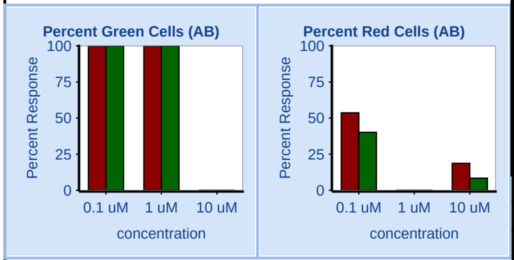

Average Response						
Dose	Green Signal	Actual Pct Green Cells	Normalized Pct Green Cells	Red Signal	Actual Pct Red Cells	Normalized Pct Red Cells
0.1 uM	107.50	0.93	141.78	122.72	0.22	46.90
1 uM	119.00	0.93	119.48	92.44	0.08	-19.20
10 uM	96.97	0.75	-853.41	96.13	0.15	13.53

Compound	Normalized Values (A and B Sets)	Responses

Nomifensine Maleate

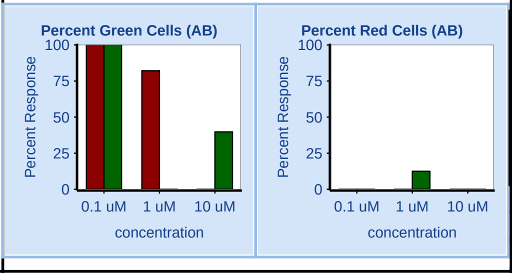

Average Response						
Dose	Green Signal	Actual Pct Green Cells	Normalized Pct Green Cells	Red Signal	Actual Pct Red Cells	Normalized Pct Red Cells
0.1 uM	100.40	0.90	108.16	77.34	0.04	-42.73
1 uM	93.70	0.76	28.19	89.01	0.10	-20.01
10 uM	91.27	0.78	18.87	79.98	0.04	-44.11

Compound	Normalized Values (A and B Sets)	Responses

Noradrenaline bitartrate monohydrate

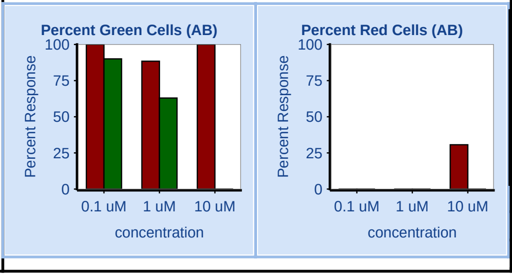

Average Response						
Dose	Green Signal	Actual Pct Green Cells	Normalized Pct Green Cells	Red Signal	Actual Pct Red Cells	Normalized Pct Red Cells
0.1 uM	98.72	0.90	108.73	82.51	0.05	-32.00
1 uM	97.38	0.85	75.70	85.80	0.05	-34.36
10 uM	97.16	0.85	137.31	101.67	0.12	-1.75

Compound	Normalized Values (A and B Sets)	Responses

Noscapine HCl

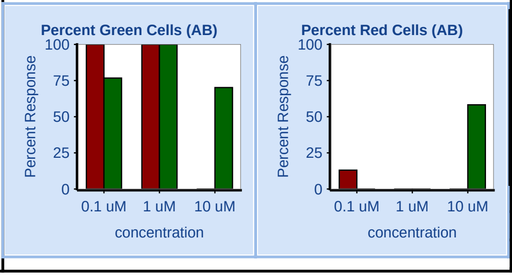

Average Response						
Dose	Green Signal	Actual Pct Green Cells	Normalized Pct Green Cells	Red Signal	Actual Pct Red Cells	Normalized Pct Red Cells
0.1 uM	99.00	0.89	114.89	100.22	0.12	-2.80
1 uM	106.79	0.90	127.25	90.94	0.07	-31.38
10 uM	94.62	0.78	-13.07	113.63	0.16	21.42

Compound	Normalized Values (A and B Sets)	Responses

NPS-2143

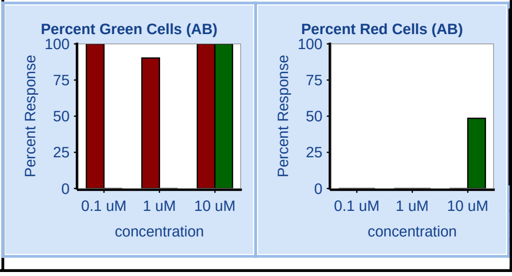

Average Response						
Dose	Green Signal	Actual Pct Green Cells	Normalized Pct Green Cells	Red Signal	Actual Pct Red Cells	Normalized Pct Red Cells
0.1 uM	86.81	0.76	-49.47	72.35	0.04	-37.45
1 uM	87.19	0.70	-18.94	77.03	0.02	-44.75
10 uM	125.42	0.98	486.31	118.73	0.17	21.34

Compound	Normalized Values (A and B Sets)	Responses

NSC 23766

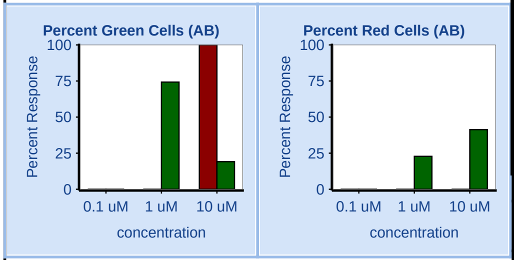

Average Response						
Dose	Green Signal	Actual Pct Green Cells	Normalized Pct Green Cells	Red Signal	Actual Pct Red Cells	Normalized Pct Red Cells
0.1 uM	84.93	0.73	-46.93	80.98	0.05	-43.45
1 uM	87.86	0.81	-419.82	90.76	0.09	1.86
10 uM	94.34	0.88	64.75	88.02	0.12	0.50

Compound | Normalized Values (A and B Sets) | Responses

NSC 405020

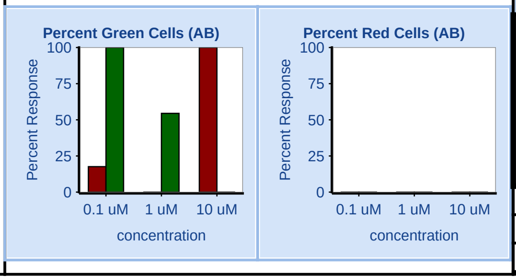

Average Response

Dose	Green Signal	Actual Pct Green Cells	Normalized Pct Green Cells	Red Signal	Actual Pct Red Cells	Normalized Pct Red Cells
0.1 uM	88.59	0.85	60.03	90.23	0.07	-28.79
1 uM	91.79	0.86	7.59	88.69	0.08	-10.13
10 uM	93.11	0.84	31.95	75.95	0.05	-29.67

Compound | Normalized Values (A and B Sets) | Responses

NSC 663284

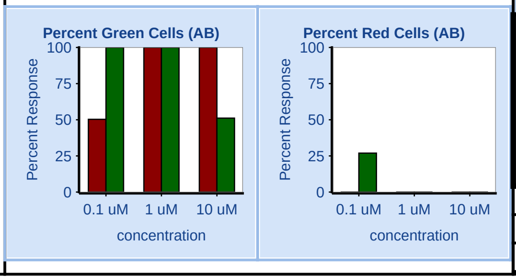

Average Response

Dose	Green Signal	Actual Pct Green Cells	Normalized Pct Green Cells	Red Signal	Actual Pct Red Cells	Normalized Pct Red Cells
0.1 uM	99.85	0.89	97.60	98.11	0.09	-19.40
1 uM	111.70	0.95	249.36	73.79	0.02	-37.33
10 uM	100.28	0.90	88.99	74.12	0.05	-27.90

Compound | Normalized Values (A and B Sets) | Responses

NU6027

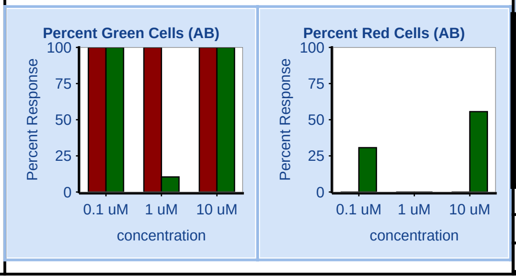

Average Response

Dose	Green Signal	Actual Pct Green Cells	Normalized Pct Green Cells	Red Signal	Actual Pct Red Cells	Normalized Pct Red Cells
0.1 uM	101.38	0.91	155.27	104.00	0.14	8.80
1 uM	98.76	0.82	67.60	92.40	0.09	-21.08
10 uM	117.25	0.97	196.74	111.88	0.17	27.60

Compound | Normalized Values (A and B Sets) | Responses

NU7441 (KU-57788)

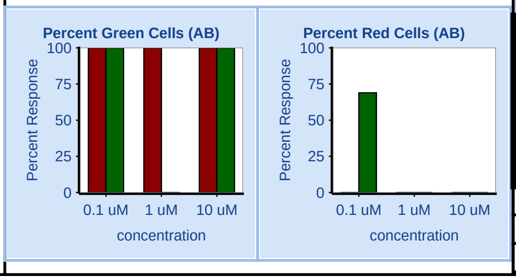

Average Response

Dose	Green Signal	Actual Pct Green Cells	Normalized Pct Green Cells	Red Signal	Actual Pct Red Cells	Normalized Pct Red Cells
0.1 uM	112.75	0.96	167.36	112.57	0.17	28.71
1 uM	99.74	0.81	46.22	85.74	0.05	-34.83
10 uM	104.26	0.95	256.97	81.35	0.04	-41.41

Compound | Normalized Values (A and B Sets) | Responses

Nutlin-3

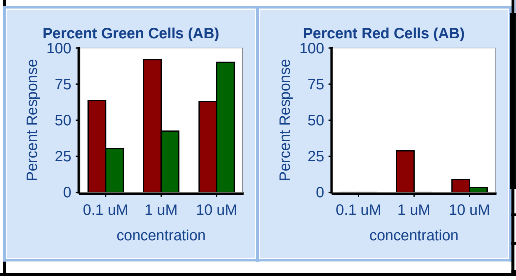

Average Response

Dose	Green Signal	Actual Pct Green Cells	Normalized Pct Green Cells	Red Signal	Actual Pct Red Cells	Normalized Pct Red Cells
0.1 uM	93.83	0.83	46.99	76.44	0.03	-58.29
1 uM	95.88	0.85	67.18	104.10	0.10	-12.92
10 uM	94.76	0.84	76.56	124.50	0.14	6.19

Compound	Normalized Values (A and B Sets)	Responses

NVP-ADW742

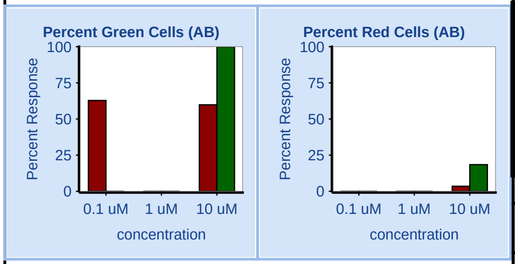

Average Response						
Dose	Green Signal	Actual Pct Green Cells	Normalized Pct Green Cells	Red Signal	Actual Pct Red Cells	Normalized Pct Red Cells
0.1 uM	86.64	0.78	17.96	88.75	0.05	-45.15
1 uM	73.74	0.61	-158.83	82.95	0.02	-47.50
10 uM	113.69	0.85	87.32	124.40	0.15	10.97

Compound	Normalized Values (A and B Sets)	Responses

NVP-AEW541

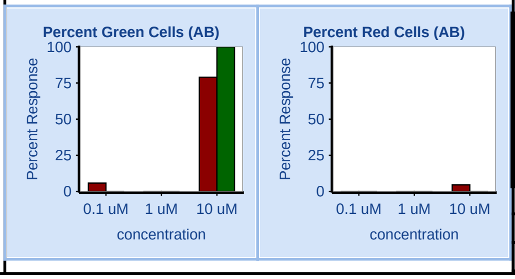

Average Response						
Dose	Green Signal	Actual Pct Green Cells	Normalized Pct Green Cells	Red Signal	Actual Pct Red Cells	Normalized Pct Red Cells
0.1 uM	74.41	0.62	-85.15	75.40	0.02	-64.60
1 uM	84.68	0.74	-45.46	85.10	0.04	-37.81
10 uM	115.85	0.87	100.47	120.25	0.13	0.17

Compound	Normalized Values (A and B Sets)	Responses

NVP-BHG712

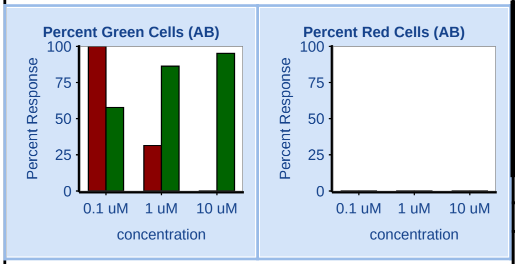

Average Response						
Dose	Green Signal	Actual Pct Green Cells	Normalized Pct Green Cells	Red Signal	Actual Pct Red Cells	Normalized Pct Red Cells
0.1 uM	94.87	0.89	89.32	74.10	0.02	-43.70
1 uM	93.93	0.81	58.93	72.78	0.01	-51.37
10 uM	99.10	0.85	-312.12	73.03	0.02	-46.94

Compound	Normalized Values (A and B Sets)	Responses

NVP-BSK805 2HCl

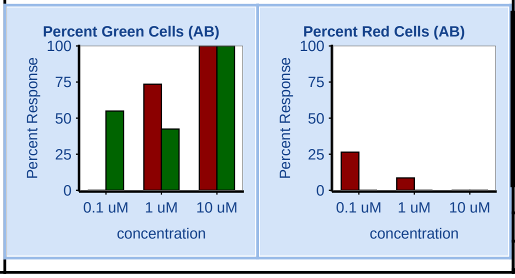

Average Response						
Dose	Green Signal	Actual Pct Green Cells	Normalized Pct Green Cells	Red Signal	Actual Pct Red Cells	Normalized Pct Red Cells
0.1 uM	83.26	0.74	-47.65	96.10	0.12	0.31
1 uM	91.86	0.82	57.97	104.31	0.12	-4.67
10 uM	88.43	0.93	249.63	86.70	0.03	-45.43

Compound	Normalized Values (A and B Sets)	Responses

NVP-BVU972

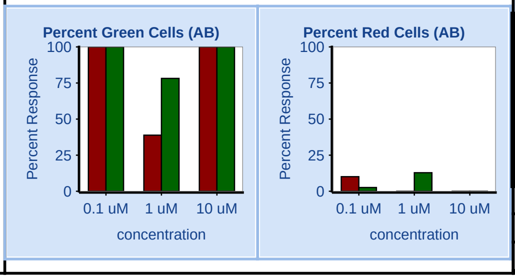

Average Response						
Dose	Green Signal	Actual Pct Green Cells	Normalized Pct Green Cells	Red Signal	Actual Pct Red Cells	Normalized Pct Red Cells
0.1 uM	102.63	0.92	129.49	100.93	0.13	6.35
1 uM	90.08	0.81	58.53	94.43	0.11	-7.15
10 uM	104.18	0.95	420.31	89.51	0.08	-22.21

Compound	Normalized Values (A and B Sets)	Responses

NXY-059

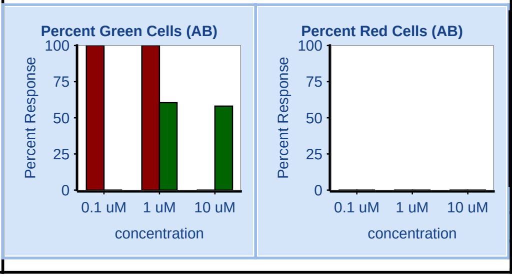

Average Response

Dose	Green Signal	Actual Pct Green Cells	Normalized Pct Green Cells	Red Signal	Actual Pct Red Cells	Normalized Pct Red Cells
0.1 uM	97.69	0.81	143.65	82.83	0.05	-37.22
1 uM	96.98	0.84	83.98	97.41	0.08	-27.29
10 uM	93.24	0.79	9.73	77.78	0.03	-48.23

Compound	Normalized Values (A and B Sets)	Responses

Nystatin (Fungicidin)

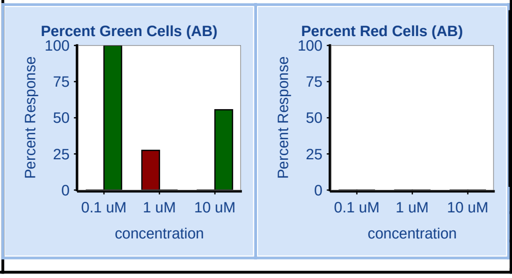

Average Response

Dose	Green Signal	Actual Pct Green Cells	Normalized Pct Green Cells	Red Signal	Actual Pct Red Cells	Normalized Pct Red Cells
0.1 uM	99.06	0.86	45.64	106.78	0.11	-18.22
1 uM	90.39	0.78	3.98	94.54	0.07	-25.97
10 uM	91.50	0.74	13.68	87.43	0.04	-33.69

Compound	Normalized Values (A and B Sets)	Responses

OAC1

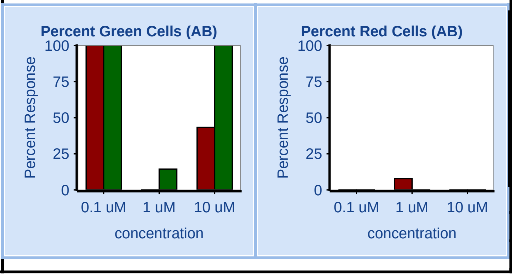

Average Response

Dose	Green Signal	Actual Pct Green Cells	Normalized Pct Green Cells	Red Signal	Actual Pct Red Cells	Normalized Pct Red Cells
0.1 uM	114.31	0.95	152.37	85.06	0.04	-49.42
1 uM	96.86	0.84	1.46	88.96	0.08	-18.77
10 uM	97.18	0.90	79.98	85.20	0.08	-16.37

Compound	Normalized Values (A and B Sets)	Responses

Obatoclax Mesylate (GX15-070)

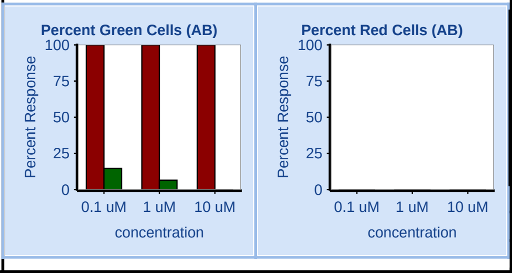

Average Response

Dose	Green Signal	Actual Pct Green Cells	Normalized Pct Green Cells	Red Signal	Actual Pct Red Cells	Normalized Pct Red Cells
0.1 uM	95.05	0.85	81.27	109.84	0.12	-8.45
1 uM	104.58	0.84	78.24	7.97	0.02	-46.73
10 uM	115.49	0.77	21.66	0.00	0.00	-52.84

Compound	Normalized Values (A and B Sets)	Responses

OC000459

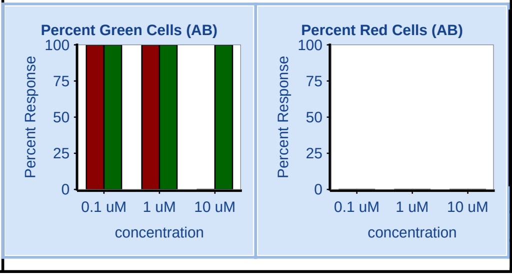

Average Response

Dose	Green Signal	Actual Pct Green Cells	Normalized Pct Green Cells	Red Signal	Actual Pct Red Cells	Normalized Pct Red Cells
0.1 uM	109.42	0.94	149.60	87.71	0.08	-18.16
1 uM	108.00	0.91	106.94	88.49	0.06	-27.41
10 uM	99.40	0.90	-62.76	93.51	0.07	-26.61

Compound | Normalized Values (A and B Sets) | Responses

Odanacatib (MK-0822)

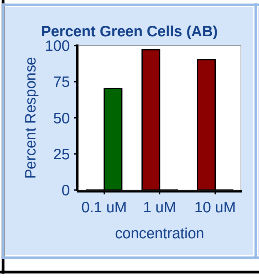

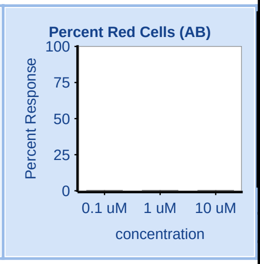

Average Response

Dose	Green Signal	Actual Pct Green Cells	Normalized Pct Green Cells	Red Signal	Actual Pct Red Cells	Normalized Pct Red Cells
0.1 uM	89.23	0.84	30.56	79.83	0.02	-63.79
1 uM	91.37	0.78	25.10	102.61	0.08	-19.41
10 uM	93.06	0.78	31.81	85.27	0.04	-38.75

Compound | Normalized Values (A and B Sets) | Responses

OG-L002

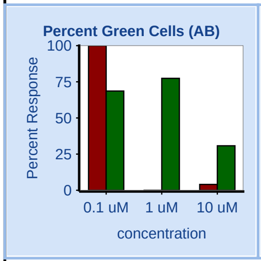

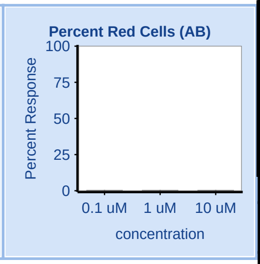

Average Response

Dose	Green Signal	Actual Pct Green Cells	Normalized Pct Green Cells	Red Signal	Actual Pct Red Cells	Normalized Pct Red Cells
0.1 uM	97.91	0.89	100.47	91.61	0.06	-39.09
1 uM	94.43	0.86	-114.42	62.91	0.01	-43.93
10 uM	90.40	0.83	17.40	76.78	0.04	-34.35

Compound | Normalized Values (A and B Sets) | Responses

Oleanolic Acid

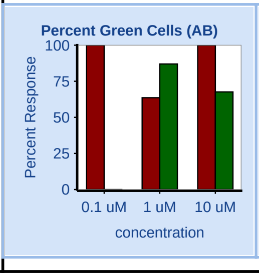

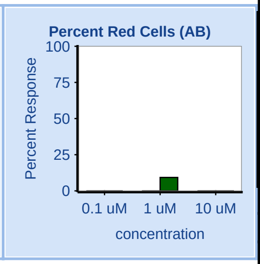

Average Response

Dose	Green Signal	Actual Pct Green Cells	Normalized Pct Green Cells	Red Signal	Actual Pct Red Cells	Normalized Pct Red Cells
0.1 uM	88.04	0.83	28.59	78.19	0.04	-36.33
1 uM	101.54	0.84	75.30	100.39	0.11	-7.97
10 uM	97.24	0.90	151.54	74.22	0.02	-47.06

Compound | Normalized Values (A and B Sets) | Responses

Oligomycin A

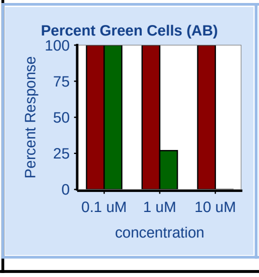

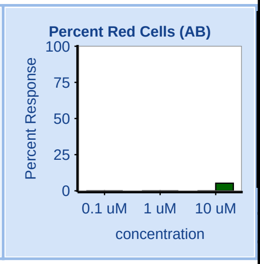

Average Response

Dose	Green Signal	Actual Pct Green Cells	Normalized Pct Green Cells	Red Signal	Actual Pct Red Cells	Normalized Pct Red Cells
0.1 uM	119.47	0.95	146.21	96.40	0.08	-31.21
1 uM	102.60	0.86	95.35	100.36	0.08	-19.59
10 uM	97.23	0.79	26.73	113.68	0.11	-4.91

Compound | Normalized Values (A and B Sets) | Responses

Olsalazine Sodium

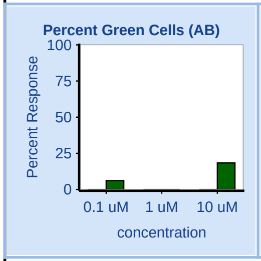

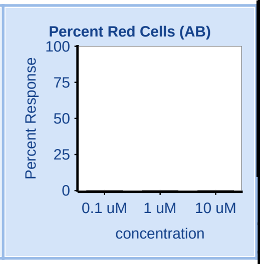

Average Response

Dose	Green Signal	Actual Pct Green Cells	Normalized Pct Green Cells	Red Signal	Actual Pct Red Cells	Normalized Pct Red Cells
0.1 uM	82.97	0.78	-183.46	55.97	0.01	-59.38
1 uM	77.74	0.59	-88.91	73.25	0.02	-57.57
10 uM	84.40	0.73	-37.59	92.29	0.06	-35.01

Compound	Normalized Values (A and B Sets)	Responses

Omecamtiv mecarbil (CK-1827452)

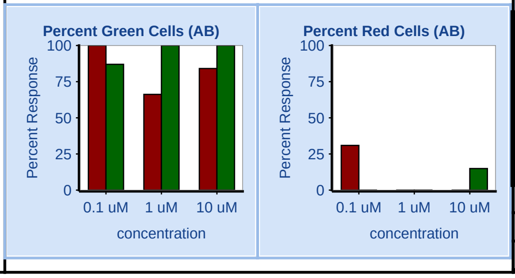

Average Response						
Dose	Green Signal	Actual Pct Green Cells	Normalized Pct Green Cells	Red Signal	Actual Pct Red Cells	Normalized Pct Red Cells
0.1 uM	98.68	0.90	99.75	102.94	0.12	-0.74
1 uM	104.61	0.86	86.76	78.62	0.03	-41.48
10 uM	102.29	0.94	154.33	100.74	0.09	-16.28

Compound	Normalized Values (A and B Sets)	Responses

ONX-0914 (PR-957)

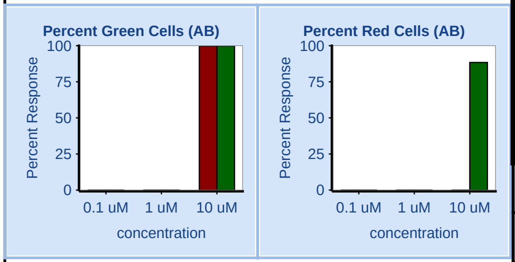

Average Response						
Dose	Green Signal	Actual Pct Green Cells	Normalized Pct Green Cells	Red Signal	Actual Pct Red Cells	Normalized Pct Red Cells
0.1 uM	98.89	0.66	-647.83	79.04	0.06	-31.37
1 uM	95.61	0.52	-137.48	79.35	0.01	-63.14
10 uM	100.52	0.97	181.11	98.05	0.14	9.44

Compound	Normalized Values (A and B Sets)	Responses

Oprozomib (ONX 0912)

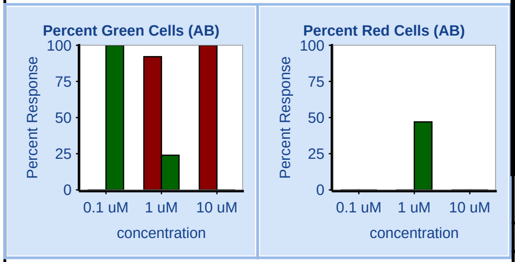

Average Response						
Dose	Green Signal	Actual Pct Green Cells	Normalized Pct Green Cells	Red Signal	Actual Pct Red Cells	Normalized Pct Red Cells
0.1 uM	99.28	0.86	-118.36	98.92	0.08	-24.69
1 uM	104.54	0.80	58.03	101.66	0.14	4.95
10 uM	90.81	0.77	87.26	83.61	0.04	-42.92

Compound	Normalized Values (A and B Sets)	Responses

Org 27569

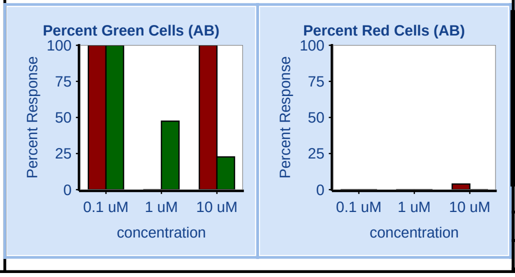

Average Response						
Dose	Green Signal	Actual Pct Green Cells	Normalized Pct Green Cells	Red Signal	Actual Pct Red Cells	Normalized Pct Red Cells
0.1 uM	109.36	0.94	126.81	98.72	0.06	-43.92
1 uM	98.07	0.81	4.38	58.89	0.01	-54.13
10 uM	100.47	0.84	72.63	105.80	0.09	-18.48

Compound	Normalized Values (A and B Sets)	Responses

Oridonin

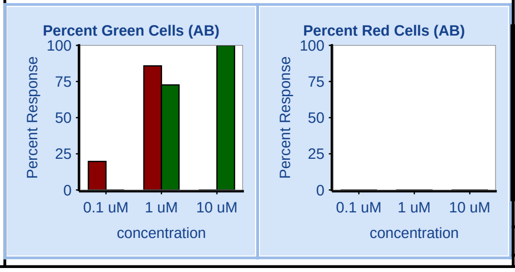

Average Response						
Dose	Green Signal	Actual Pct Green Cells	Normalized Pct Green Cells	Red Signal	Actual Pct Red Cells	Normalized Pct Red Cells
0.1 uM	85.66	0.79	-11.98	77.09	0.04	-35.31
1 uM	97.31	0.86	79.23	87.93	0.05	-33.82

Compound	Normalized Values (A and B Sets)	Responses						
		10 uM	103.42	0.88	-132.82	82.05	0.06	-29.81

Compound	Normalized Values (A and B Sets)	Responses						
**Orotic acid (6-Carboxyuracil)**	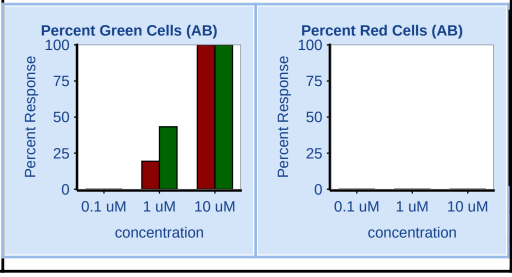	Average Response						
		Dose	Green Signal	Actual Pct Green Cells	Normalized Pct Green Cells	Red Signal	Actual Pct Red Cells	Normalized Pct Red Cells
		0.1 uM	81.35	0.78	-13.93	101.48	0.10	-8.20
		1 uM	88.34	0.76	31.34	94.92	0.07	-26.08
		10 uM	103.25	0.94	349.64	76.13	0.02	-50.03

Compound	Normalized Values (A and B Sets)	Responses						
**Oseltamivir phosphate**	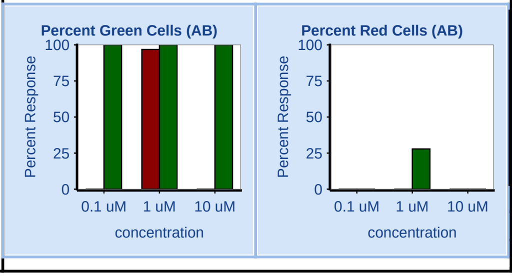	Average Response						
		Dose	Green Signal	Actual Pct Green Cells	Normalized Pct Green Cells	Red Signal	Actual Pct Red Cells	Normalized Pct Red Cells
		0.1 uM	95.56	0.87	81.54	98.53	0.10	-10.09
		1 uM	105.73	0.89	98.94	102.03	0.12	-2.43
		10 uM	99.82	0.91	73.31	103.43	0.09	-14.26

Compound	Normalized Values (A and B Sets)	Responses						
**OSI-027**	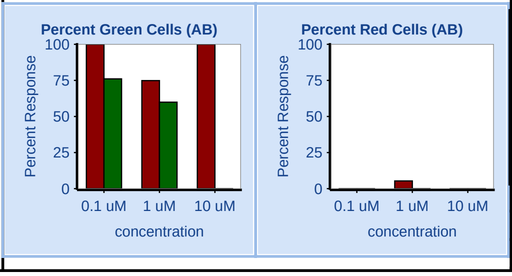	Average Response						
		Dose	Green Signal	Actual Pct Green Cells	Normalized Pct Green Cells	Red Signal	Actual Pct Red Cells	Normalized Pct Red Cells
		0.1 uM	95.37	0.89	95.24	78.24	0.05	-33.67
		1 uM	96.55	0.83	67.38	93.63	0.09	-14.11
		10 uM	90.43	0.91	399.61	90.03	0.07	-23.91

Compound	Normalized Values (A and B Sets)	Responses						
**OSI-420**	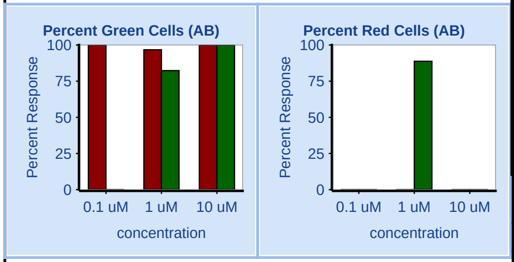	Average Response						
		Dose	Green Signal	Actual Pct Green Cells	Normalized Pct Green Cells	Red Signal	Actual Pct Red Cells	Normalized Pct Red Cells
		0.1 uM	83.52	0.71	-111.89	60.21	0.01	-51.55
		1 uM	95.99	0.88	89.51	119.59	0.20	32.68
		10 uM	94.46	0.94	332.16	94.50	0.05	-32.39

Compound	Normalized Values (A and B Sets)	Responses						
**OSI-906 (Linsitinib)**		Average Response						
		Dose	Green Signal	Actual Pct Green Cells		Red Signal	Actual Pct Red Cells	Normalized Pct Red Cells

Compound	Normalized Values (A and B Sets)	Responses

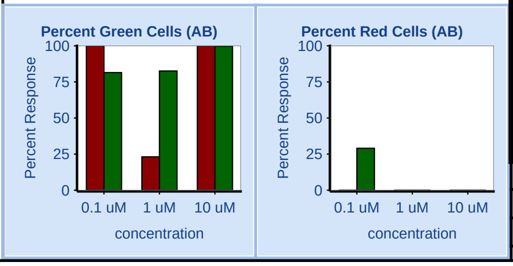

			Normalized Pct Green Cells			
0.1 uM	98.73	0.91	116.82	104.62	0.11	-8.57
1 uM	92.13	0.86	52.85	80.70	0.02	-46.41
10 uM	101.84	0.89	105.96	101.68	0.08	-19.34

Compound	Normalized Values (A and B Sets)	Responses

OSI-930

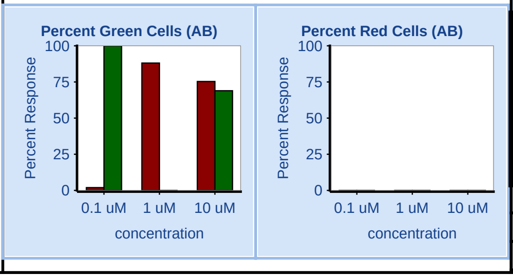

Average Response

Dose	Green Signal	Actual Pct Green Cells	Normalized Pct Green Cells	Red Signal	Actual Pct Red Cells	Normalized Pct Red Cells
0.1 uM	91.25	0.87	54.45	67.18	0.00	-70.77
1 uM	92.48	0.81	39.27	84.48	0.04	-40.51
10 uM	99.35	0.84	72.12	115.40	0.10	-11.35

Compound	Normalized Values (A and B Sets)	Responses

OSU-03012 (AR-12)

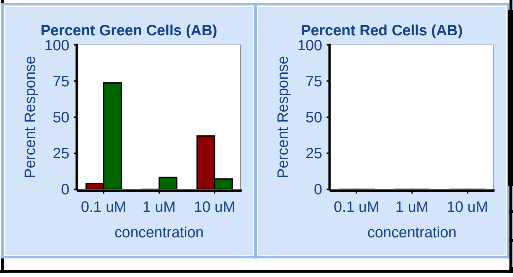

Average Response

Dose	Green Signal	Actual Pct Green Cells	Normalized Pct Green Cells	Red Signal	Actual Pct Red Cells	Normalized Pct Red Cells
0.1 uM	94.44	0.84	38.82	79.81	0.03	-58.65
1 uM	91.07	0.78	-22.51	79.51	0.02	-46.79
10 uM	90.13	0.76	22.03	77.62	0.01	-47.98

Compound	Normalized Values (A and B Sets)	Responses

Otilonium Bromide

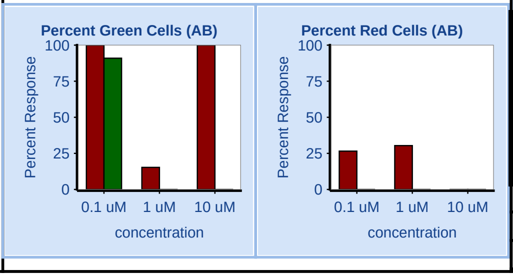

Average Response

Dose	Green Signal	Actual Pct Green Cells	Normalized Pct Green Cells	Red Signal	Actual Pct Red Cells	Normalized Pct Red Cells
0.1 uM	107.03	0.92	213.92	97.25	0.11	-8.58
1 uM	87.51	0.71	-11.05	106.80	0.12	-3.36
10 uM	88.25	0.61	30.56	85.96	0.04	-42.13

Compound	Normalized Values (A and B Sets)	Responses

OTSSP167

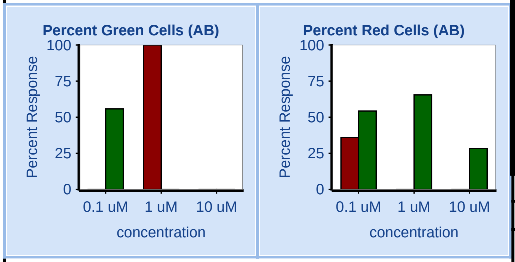

Average Response

Dose	Green Signal	Actual Pct Green Cells	Normalized Pct Green Cells	Red Signal	Actual Pct Red Cells	Normalized Pct Red Cells
0.1 uM	107.84	0.85	21.29	112.56	0.21	45.07
1 uM	101.61	0.70	-22.40	109.83	0.17	20.57
10 uM	88.75	0.64	-129.06	100.69	0.13	6.07

Compound | Normalized Values (A and B Sets) | Responses

OTX015

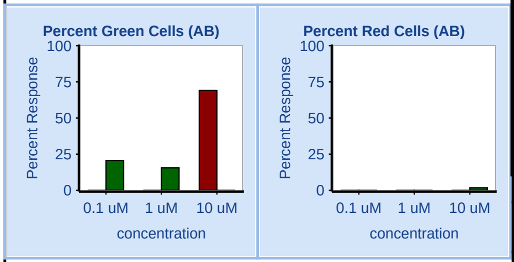

Average Response						
Dose	Green Signal	Actual Pct Green Cells	Normalized Pct Green Cells	Red Signal	Actual Pct Red Cells	Normalized Pct Red Cells
0.1 uM	100.95	0.79	8.43	91.49	0.08	-24.82
1 uM	96.37	0.80	-275.98	77.78	0.04	-29.24
10 uM	95.16	0.75	-50.19	97.77	0.09	-9.90

Compound | Normalized Values (A and B Sets) | Responses

Ouabain

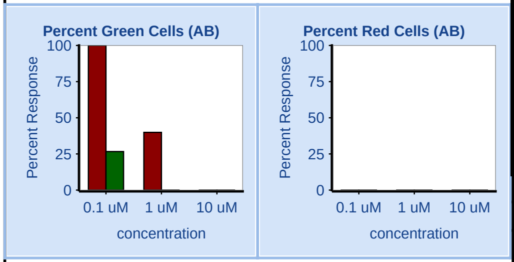

Average Response						
Dose	Green Signal	Actual Pct Green Cells	Normalized Pct Green Cells	Red Signal	Actual Pct Red Cells	Normalized Pct Red Cells
0.1 uM	99.31	0.85	96.26	82.74	0.09	-17.31
1 uM	92.25	0.74	11.68	86.13	0.08	-28.19
10 uM	70.36	0.53	-318.59	49.68	0.02	-55.14

Compound | Normalized Values (A and B Sets) | Responses

Oxaliplatin

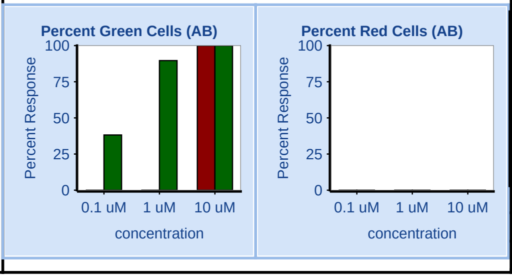

Average Response						
Dose	Green Signal	Actual Pct Green Cells	Normalized Pct Green Cells	Red Signal	Actual Pct Red Cells	Normalized Pct Red Cells
0.1 uM	81.34	0.77	-38.70	81.02	0.03	-60.10
1 uM	96.74	0.85	38.70	82.43	0.03	-45.41
10 uM	116.49	0.94	139.16	96.11	0.05	-30.83

Compound | Normalized Values (A and B Sets) | Responses

Oxeladin Citrate

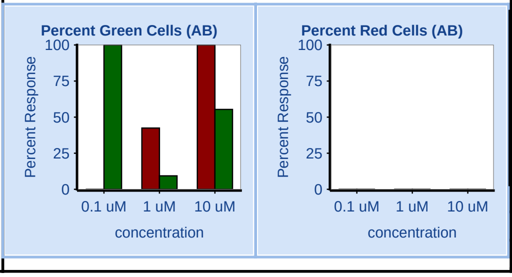

Average Response						
Dose	Green Signal	Actual Pct Green Cells	Normalized Pct Green Cells	Red Signal	Actual Pct Red Cells	Normalized Pct Red Cells
0.1 uM	89.57	0.88	-71.66	79.52	0.04	-44.59
1 uM	93.30	0.76	25.88	76.75	0.03	-54.31
10 uM	94.26	0.83	81.23	89.73	0.04	-42.03

Compound | Normalized Values (A and B Sets) | Responses

Oxybutynin

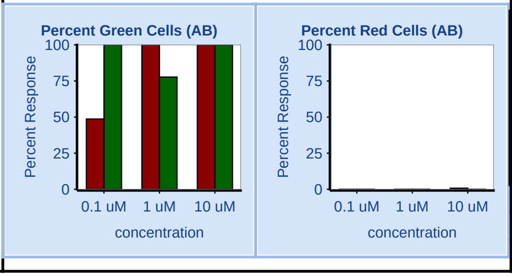

Average Response						
Dose	Green Signal	Actual Pct Green Cells	Normalized Pct Green Cells	Red Signal	Actual Pct Red Cells	Normalized Pct Red Cells
0.1 uM	100.09	0.89	76.86	83.91	0.04	-54.14
1 uM	105.97	0.89	104.70	78.65	0.03	-43.35
10 uM	112.46	0.95	142.86	108.90	0.08	-20.21

Compound | Normalized Values (A and B Sets) | Responses

Oxymatrine

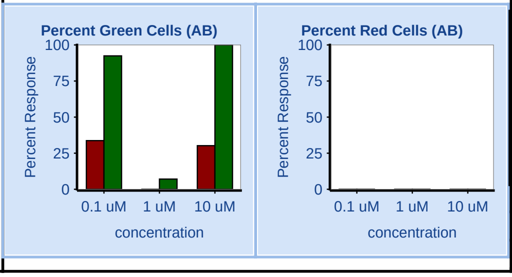

Average Response						
Dose	Green Signal	Actual Pct Green Cells	Normalized Pct Green Cells	Red Signal	Actual Pct Red Cells	Normalized Pct Red Cells
0.1 uM	92.44	0.86	63.07	80.59	0.03	-39.79
1 uM	82.20	0.67	-11.79	90.81	0.05	-34.04
10 uM	90.21	0.90	70.99	72.10	0.01	-51.73

Compound | Normalized Values (A and B Sets) | Responses

Oxymetholone

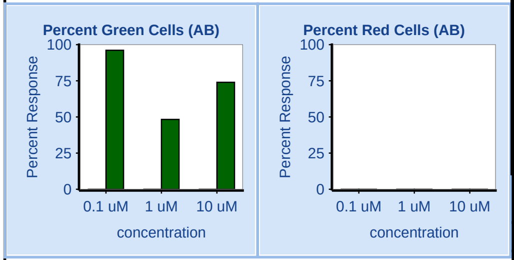

Average Response						
Dose	Green Signal	Actual Pct Green Cells	Normalized Pct Green Cells	Red Signal	Actual Pct Red Cells	Normalized Pct Red Cells
0.1 uM	90.05	0.85	-107.80	81.43	0.06	-34.79
1 uM	86.84	0.70	-12.29	80.46	0.03	-52.10
10 uM	89.42	0.78	-17.71	77.62	0.03	-48.19

Compound | Normalized Values (A and B Sets) | Responses

Oxytetracycline (Terramycin)

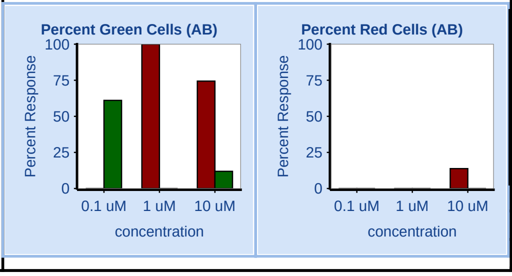

Average Response						
Dose	Green Signal	Actual Pct Green Cells	Normalized Pct Green Cells	Red Signal	Actual Pct Red Cells	Normalized Pct Red Cells
0.1 uM	92.37	0.82	9.42	83.24	0.04	-50.43
1 uM	95.92	0.77	23.33	94.79	0.06	-31.45
10 uM	95.70	0.80	43.17	113.06	0.10	-12.44

Compound | Normalized Values (A and B Sets) | Responses

Ozagrel

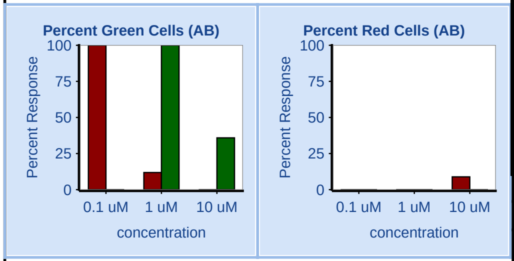

Average Response						
Dose	Green Signal	Actual Pct Green Cells	Normalized Pct Green Cells	Red Signal	Actual Pct Red Cells	Normalized Pct Red Cells
0.1 uM	87.82	0.74	-69.24	84.38	0.05	-30.06
1 uM	100.24	0.81	65.72	85.35	0.05	-34.62
10 uM	81.31	0.75	-984.62	93.31	0.08	-20.01

Compound | Normalized Values (A and B Sets) | Responses

P276-00

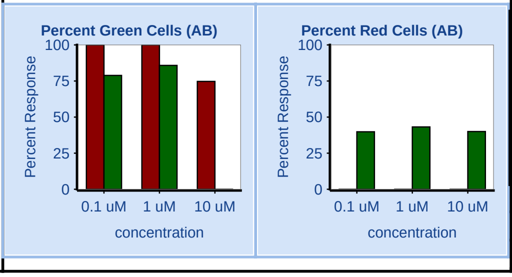

Average Response						
Dose	Green Signal	Actual Pct Green Cells	Normalized Pct Green Cells	Red Signal	Actual Pct Red Cells	Normalized Pct Red Cells
0.1 uM	111.07	0.90	112.08	89.55	0.10	-16.88
1 uM	103.43	0.90	135.05	81.76	0.10	10.67
10 uM	86.50	0.76	-38.90	92.67	0.15	12.52

Compound	Normalized Values (A and B Sets)	Responses

P22077

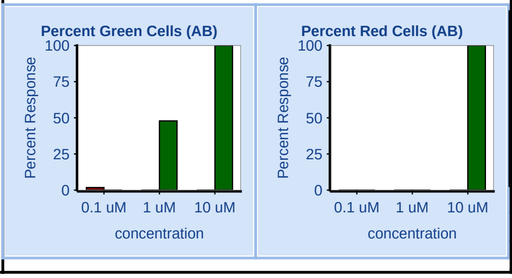

Average Response						
Dose	Green Signal	Actual Pct Green Cells	Normalized Pct Green Cells	Red Signal	Actual Pct Red Cells	Normalized Pct Red Cells
0.1 uM	82.07	0.73	-63.27	81.09	0.05	-39.65
1 uM	90.09	0.74	17.91	79.83	0.03	-49.39
10 uM	99.04	0.84	36.76	123.89	0.23	54.52

Compound	Normalized Values (A and B Sets)	Responses

PA-824

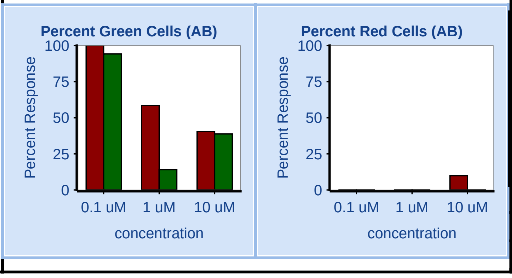

Average Response						
Dose	Green Signal	Actual Pct Green Cells	Normalized Pct Green Cells	Red Signal	Actual Pct Red Cells	Normalized Pct Red Cells
0.1 uM	108.03	0.93	130.94	87.88	0.07	-31.20
1 uM	98.21	0.82	36.31	95.99	0.09	-16.69
10 uM	90.22	0.79	39.66	119.22	0.13	-0.91

Compound	Normalized Values (A and B Sets)	Responses

PAC-1

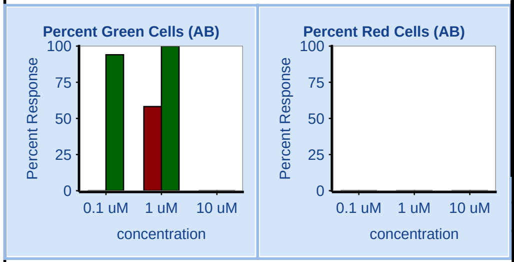

Average Response						
Dose	Green Signal	Actual Pct Green Cells	Normalized Pct Green Cells	Red Signal	Actual Pct Red Cells	Normalized Pct Red Cells
0.1 uM	90.33	0.83	40.80	77.49	0.03	-38.93
1 uM	98.72	0.85	81.39	75.45	0.03	-42.53
10 uM	90.65	0.79	-373.09	80.67	0.06	-28.37

Compound	Normalized Values (A and B Sets)	Responses

Pacritinib (SB1518)

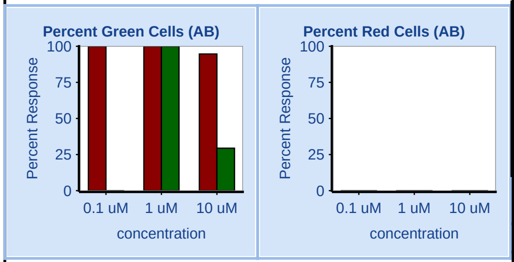

Average Response						
Dose	Green Signal	Actual Pct Green Cells	Normalized Pct Green Cells	Red Signal	Actual Pct Red Cells	Normalized Pct Red Cells
0.1 uM	73.91	0.58	-155.49	58.85	0.01	-71.30
1 uM	98.78	0.93	148.76	65.51	0.02	-36.73
10 uM	98.39	0.88	62.05	89.35	0.08	-18.26

Compound	Normalized Values (A and B Sets)	Responses

Paeoniflorin

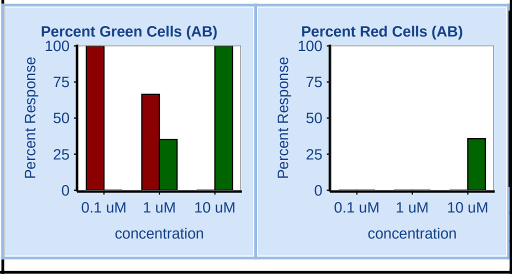

Average Response						
Dose	Green Signal	Actual Pct Green Cells	Normalized Pct Green Cells	Red Signal	Actual Pct Red Cells	Normalized Pct Red Cells
0.1 uM	89.84	0.81	3.99	83.13	0.04	-34.36
1 uM	94.06	0.80	50.80	66.25	0.01	-51.46
10 uM	98.79	0.90	62.78	101.77	0.11	-5.48

Compound | Normalized Values (A and B Sets) | Responses

Palbociclib (PD-0332991) HCl

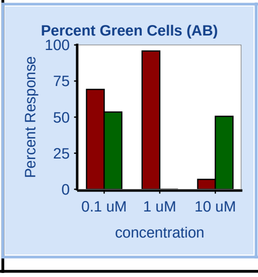

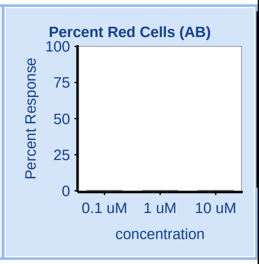

Average Response						
Dose	Green Signal	Actual Pct Green Cells	Normalized Pct Green Cells	Red Signal	Actual Pct Red Cells	Normalized Pct Red Cells
0.1 uM	88.40	0.85	61.36	76.68	0.02	-63.55
1 uM	83.76	0.66	-48.22	78.66	0.04	-39.31
10 uM	81.90	0.76	28.69	95.62	0.03	-41.41

Compound | Normalized Values (A and B Sets) | Responses

Palomid 529 (P529)

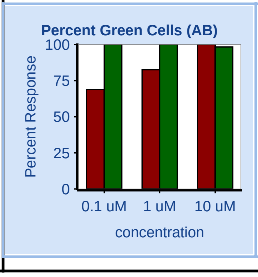

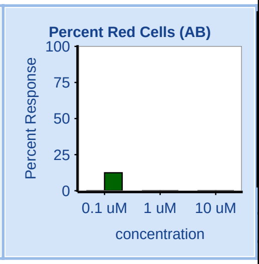

Average Response						
Dose	Green Signal	Actual Pct Green Cells	Normalized Pct Green Cells	Red Signal	Actual Pct Red Cells	Normalized Pct Red Cells
0.1 uM	98.58	0.92	127.47	92.60	0.08	-13.80
1 uM	107.26	0.89	101.84	80.30	0.04	-39.37
10 uM	101.51	0.93	354.84	71.44	0.03	-46.52

Compound | Normalized Values (A and B Sets) | Responses

Panobinostat (LBH589)

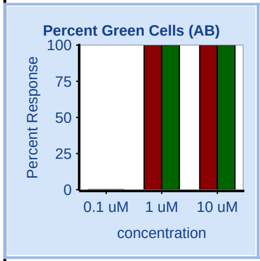

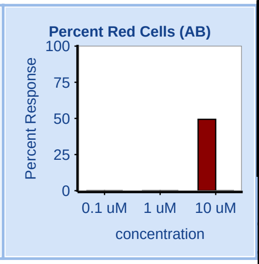

Average Response						
Dose	Green Signal	Actual Pct Green Cells	Normalized Pct Green Cells	Red Signal	Actual Pct Red Cells	Normalized Pct Red Cells
0.1 uM	92.95	0.68	-125.20	103.99	0.11	-13.47
1 uM	107.96	0.95	163.52	120.78	0.08	-22.38
10 uM	105.84	0.92	129.28	130.82	0.16	10.22

Compound | Normalized Values (A and B Sets) | Responses

Pasiniazid

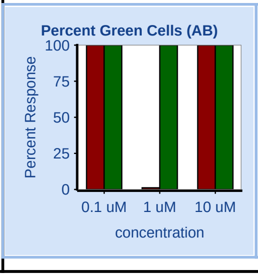

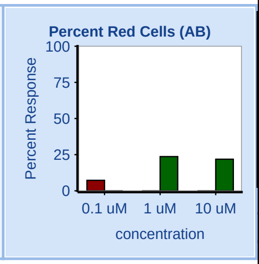

Average Response						
Dose	Green Signal	Actual Pct Green Cells	Normalized Pct Green Cells	Red Signal	Actual Pct Red Cells	Normalized Pct Red Cells
0.1 uM	116.49	0.98	269.97	98.19	0.10	-13.43
1 uM	99.18	0.80	59.10	95.61	0.12	-8.52
10 uM	112.54	0.92	163.64	97.11	0.12	0.03

Compound | Normalized Values (A and B Sets) | Responses

PB 28 dihydrochloride

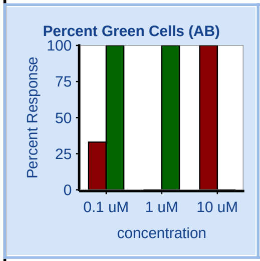

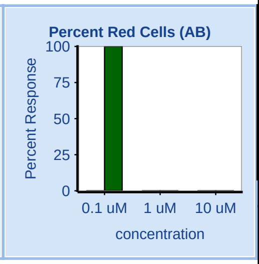

Average Response						
Dose	Green Signal	Actual Pct Green Cells	Normalized Pct Green Cells	Red Signal	Actual Pct Red Cells	Normalized Pct Red Cells
0.1 uM	105.35	0.90	104.85	103.68	0.16	19.11
1 uM	74.41	0.59	-2017.62	76.57	0.02	-37.05

Compound	Normalized Values (A and B Sets)	Responses

10 uM	91.55	0.83	26.35	64.60	0.01	-44.88

Compound	Normalized Values (A and B Sets)	Responses

PCI-34051

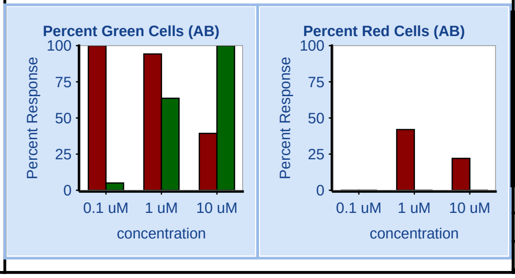

Average Response

Dose	Green Signal	Actual Pct Green Cells	Normalized Pct Green Cells	Red Signal	Actual Pct Red Cells	Normalized Pct Red Cells
0.1 uM	94.06	0.83	67.57	83.41	0.05	-48.28
1 uM	103.29	0.87	78.94	119.81	0.16	16.90
10 uM	112.31	0.85	85.45	123.03	0.15	9.73

Compound	Normalized Values (A and B Sets)	Responses

PD 166285

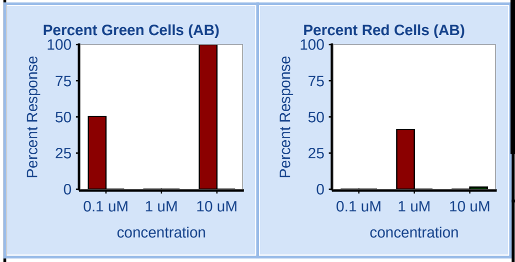

Average Response

Dose	Green Signal	Actual Pct Green Cells	Normalized Pct Green Cells	Red Signal	Actual Pct Red Cells	Normalized Pct Red Cells
0.1 uM	68.14	0.55	-189.93	75.60	0.05	-48.09
1 uM	78.08	0.53	-1109.32	99.09	0.14	7.79
10 uM	74.54	0.77	-28.61	93.53	0.07	-21.98

Compound	Normalized Values (A and B Sets)	Responses

PD123319

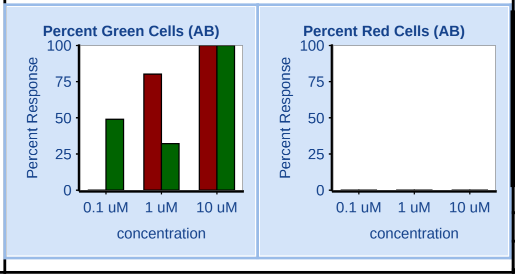

Average Response

Dose	Green Signal	Actual Pct Green Cells	Normalized Pct Green Cells	Red Signal	Actual Pct Red Cells	Normalized Pct Red Cells
0.1 uM	93.75	0.85	3.72	87.72	0.07	-27.36
1 uM	94.70	0.80	56.19	82.77	0.04	-44.92
10 uM	110.21	0.94	172.49	81.06	0.04	-44.01

Compound	Normalized Values (A and B Sets)	Responses

PD128907 HCl

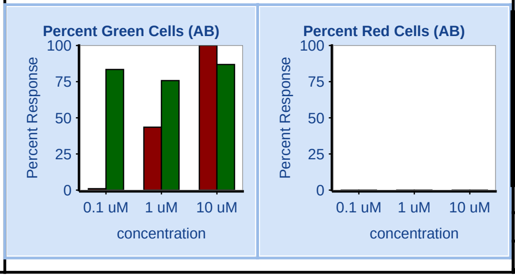

Average Response

Dose	Green Signal	Actual Pct Green Cells	Normalized Pct Green Cells	Red Signal	Actual Pct Red Cells	Normalized Pct Red Cells
0.1 uM	88.06	0.83	42.20	77.24	0.04	-37.06
1 uM	93.96	0.81	59.63	65.72	0.02	-48.15
10 uM	94.35	0.91	246.91	69.99	0.01	-54.06

Compound	Normalized Values (A and B Sets)	Responses

PD168393

Average Response

Dose	Green Signal			Red Signal	Actual Pct Red Cells	

Compound	Normalized Values (A and B Sets)	Responses

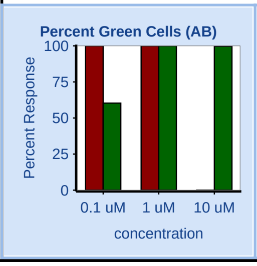

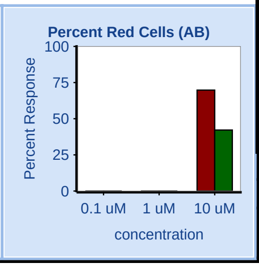

		Actual Pct Green Cells	Normalized Pct Green Cells			Normalized Pct Red Cells
0.1 uM	101.82	0.89	177.34	91.62	0.09	-16.63
1 uM	103.38	0.89	122.05	81.81	0.05	-41.53
10 uM	90.05	0.72	-141.07	126.13	0.22	55.99

Compound	Normalized Values (A and B Sets)	Responses

PD173074

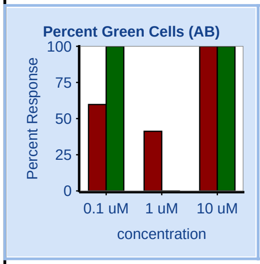

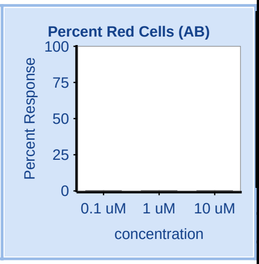

Average Response						
Dose	Green Signal	Actual Pct Green Cells	Normalized Pct Green Cells	Red Signal	Actual Pct Red Cells	Normalized Pct Red Cells
0.1 uM	98.63	0.89	79.99	109.28	0.10	-23.00
1 uM	80.55	0.67	-57.03	82.34	0.02	-46.51
10 uM	107.98	0.93	134.47	102.01	0.04	-36.53

Compound	Normalized Values (A and B Sets)	Responses

PD318088

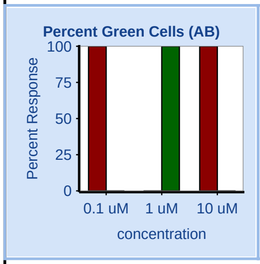

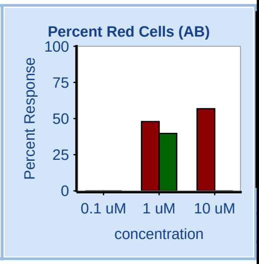

Average Response						
Dose	Green Signal	Actual Pct Green Cells	Normalized Pct Green Cells	Red Signal	Actual Pct Red Cells	Normalized Pct Red Cells
0.1 uM	98.31	0.84	92.92	92.54	0.06	-42.17
1 uM	98.35	0.81	-68.40	131.62	0.22	43.81
10 uM	98.56	0.78	26.23	122.45	0.16	9.16

Compound	Normalized Values (A and B Sets)	Responses

Pefloxacin Mesylate Dihydrate

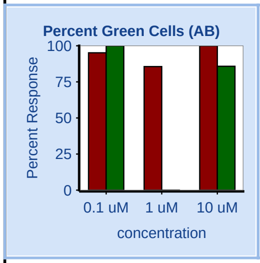

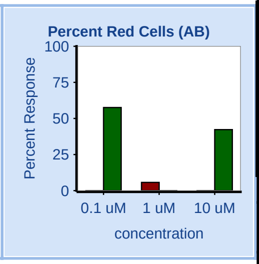

Average Response						
Dose	Green Signal	Actual Pct Green Cells	Normalized Pct Green Cells	Red Signal	Actual Pct Red Cells	Normalized Pct Red Cells
0.1 uM	100.84	0.91	107.88	106.10	0.16	16.28
1 uM	71.26	0.49	-179.13	83.15	0.07	-29.08
10 uM	103.07	0.89	135.50	95.17	0.12	-3.16

Compound	Normalized Values (A and B Sets)	Responses

Pelitinib (EKB-569)

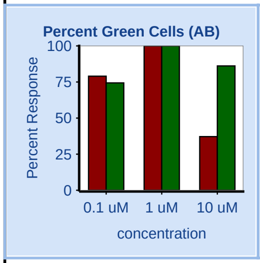

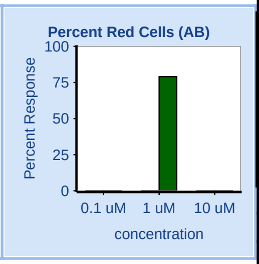

Average Response						
Dose	Green Signal	Actual Pct Green Cells	Normalized Pct Green Cells	Red Signal	Actual Pct Red Cells	Normalized Pct Red Cells
0.1 uM	99.65	0.87	76.70	85.97	0.04	-51.21
1 uM	114.14	0.95	141.66	132.89	0.20	37.20
10 uM	105.95	0.81	61.66	85.13	0.02	-44.90

Compound	Normalized Values (A and B Sets)	Responses

Penciclovir

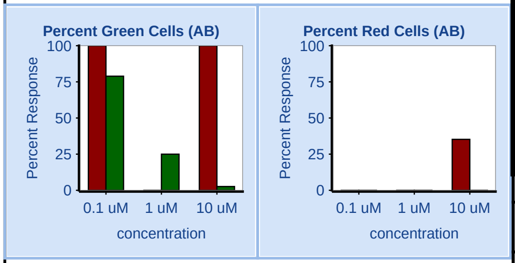

Average Response

Dose	Green Signal	Actual Pct Green Cells	Normalized Pct Green Cells	Red Signal	Actual Pct Red Cells	Normalized Pct Red Cells
0.1 uM	105.50	0.91	119.52	89.19	0.04	-50.67
1 uM	90.74	0.82	-176.71	79.46	0.04	-29.36
10 uM	104.44	0.89	78.86	102.15	0.13	6.83

Compound	Normalized Values (A and B Sets)	Responses

Penfluridol

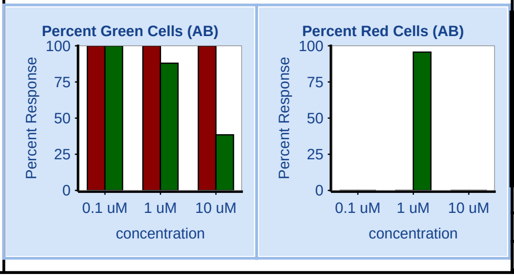

Average Response

Dose	Green Signal	Actual Pct Green Cells	Normalized Pct Green Cells	Red Signal	Actual Pct Red Cells	Normalized Pct Red Cells
0.1 uM	102.33	0.93	132.62	69.92	0.02	-53.76
1 uM	108.93	0.85	96.28	109.37	0.17	17.83
10 uM	88.96	0.86	137.89	76.86	0.00	-64.01

Compound	Normalized Values (A and B Sets)	Responses

Pentamidine

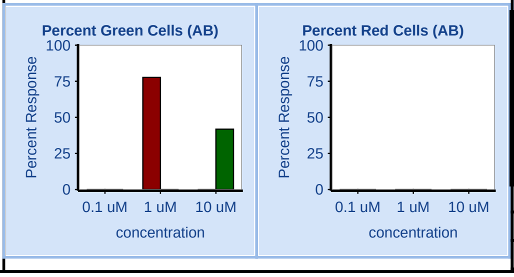

Average Response

Dose	Green Signal	Actual Pct Green Cells	Normalized Pct Green Cells	Red Signal	Actual Pct Red Cells	Normalized Pct Red Cells
0.1 uM	84.07	0.79	-93.76	65.18	0.05	-39.90
1 uM	86.01	0.74	10.00	73.19	0.02	-56.90
10 uM	88.12	0.77	-0.96	75.20	0.04	-42.63

Compound	Normalized Values (A and B Sets)	Responses

Pentostatin

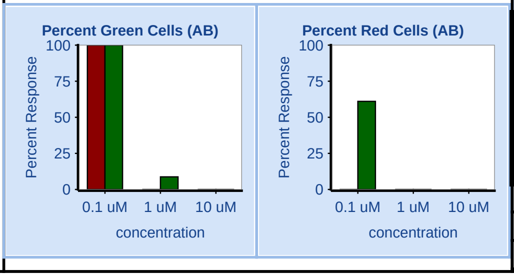

Average Response

Dose	Green Signal	Actual Pct Green Cells	Normalized Pct Green Cells	Red Signal	Actual Pct Red Cells	Normalized Pct Red Cells
0.1 uM	110.34	0.93	132.82	95.40	0.12	-0.54
1 uM	89.60	0.83	-69.40	74.55	0.03	-33.44
10 uM	77.39	0.75	-61.31	75.32	0.03	-37.58

Compound	Normalized Values (A and B Sets)	Responses

PF 477736

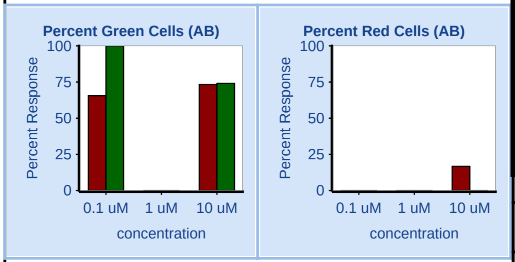

Average Response

Dose	Green Signal	Actual Pct Green Cells	Normalized Pct Green Cells	Red Signal	Actual Pct Red Cells	Normalized Pct Red Cells
0.1 uM	100.08	0.92	121.30	101.06	0.07	-31.70
1 uM	77.93	0.71	-467.13	84.81	0.04	-31.66
10 uM	103.24	0.89	73.65	97.46	0.13	8.07

Compound	Normalized Values (A and B Sets)	Responses

PF-543

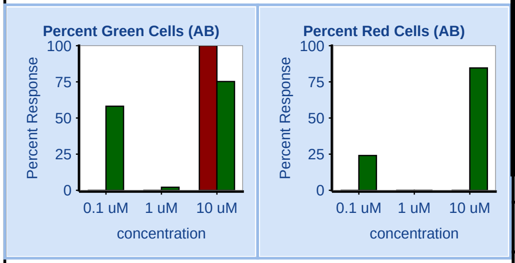

Average Response						
Dose	Green Signal	Actual Pct Green Cells	Normalized Pct Green Cells	Red Signal	Actual Pct Red Cells	Normalized Pct Red Cells
0.1 uM	90.50	0.84	-49.74	96.45	0.10	-13.77
1 uM	77.12	0.57	-103.05	77.34	0.03	-51.04
10 uM	100.47	0.87	117.04	113.27	0.18	27.95

Compound	Normalized Values (A and B Sets)	Responses

PF-3845

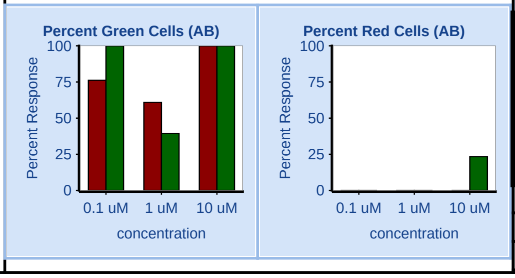

Average Response						
Dose	Green Signal	Actual Pct Green Cells	Normalized Pct Green Cells	Red Signal	Actual Pct Red Cells	Normalized Pct Red Cells
0.1 uM	96.66	0.89	93.38	92.74	0.07	-21.18
1 uM	93.78	0.80	50.19	86.90	0.05	-33.53
10 uM	100.63	0.91	182.03	114.85	0.14	9.44

Compound	Normalized Values (A and B Sets)	Responses

PF-477736

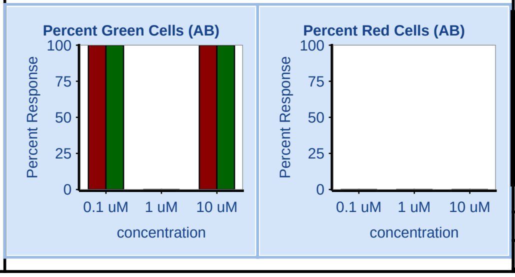

Average Response						
Dose	Green Signal	Actual Pct Green Cells	Normalized Pct Green Cells	Red Signal	Actual Pct Red Cells	Normalized Pct Red Cells
0.1 uM	102.32	0.94	227.44	60.97	0.01	-58.88
1 uM	81.95	0.69	-21.43	69.40	0.01	-61.99
10 uM	115.34	0.92	146.96	97.38	0.07	-27.70

Compound	Normalized Values (A and B Sets)	Responses

PF-00562271

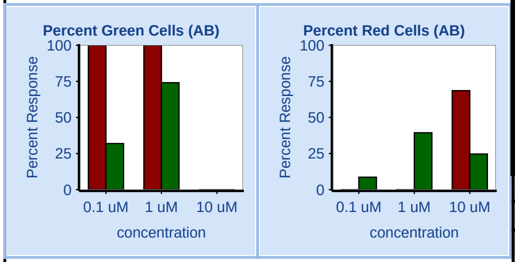

Average Response						
Dose	Green Signal	Actual Pct Green Cells	Normalized Pct Green Cells	Red Signal	Actual Pct Red Cells	Normalized Pct Red Cells
0.1 uM	102.61	0.89	91.21	95.51	0.12	0.24
1 uM	105.60	0.87	87.28	94.30	0.12	-0.23
10 uM	98.55	0.80	-282.75	133.27	0.22	46.64

Compound	Normalized Values (A and B Sets)	Responses

PF-562271

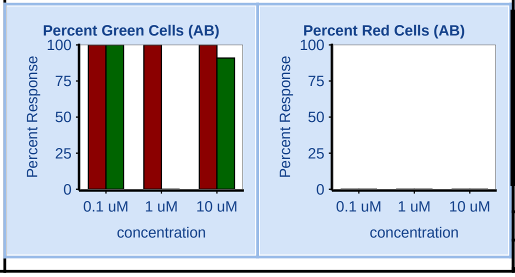

Average Response						
Dose	Green Signal	Actual Pct Green Cells	Normalized Pct Green Cells	Red Signal	Actual Pct Red Cells	Normalized Pct Red Cells
0.1 uM	109.47	0.96	248.50	70.83	0.03	-50.31
1 uM	90.82	0.79	44.23	71.65	0.02	-55.98
10 uM	118.24	0.88	119.42	88.99	0.09	-17.59

Compound	Normalized Values (A and B Sets)	Responses

PF-573228

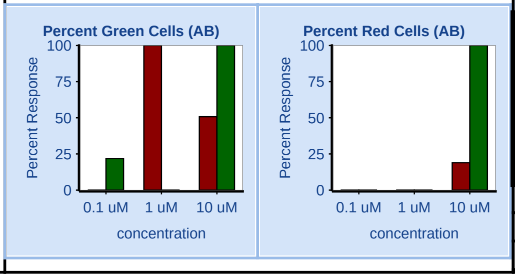

Average Response						
Dose	Green Signal	Actual Pct Green Cells	Normalized Pct Green Cells	Red Signal	Actual Pct Red Cells	Normalized Pct Red Cells
0.1 uM	83.42	0.73	-75.38	81.94	0.04	-47.12
1 uM	95.25	0.79	36.44	85.00	0.04	-40.15
10 uM	99.90	0.86	94.74	150.24	0.27	69.57

Compound	Normalized Values (A and B Sets)	Responses

PF-2545920

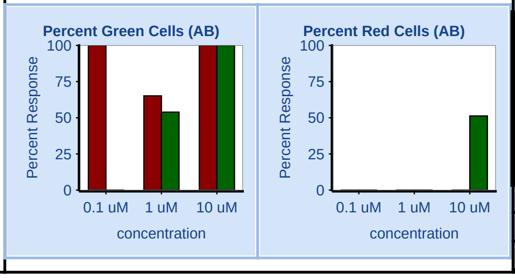

Average Response						
Dose	Green Signal	Actual Pct Green Cells	Normalized Pct Green Cells	Red Signal	Actual Pct Red Cells	Normalized Pct Red Cells
0.1 uM	89.52	0.83	24.63	79.76	0.03	-42.28
1 uM	95.22	0.82	59.53	95.82	0.08	-21.94
10 uM	102.01	0.94	221.15	112.48	0.13	4.13

Compound	Normalized Values (A and B Sets)	Responses

PF-3716556

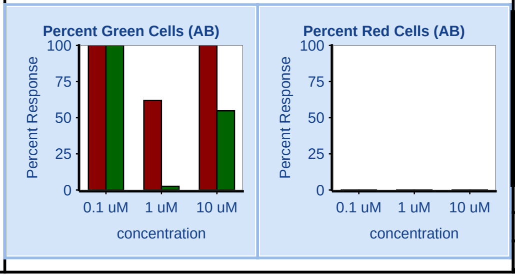

Average Response						
Dose	Green Signal	Actual Pct Green Cells	Normalized Pct Green Cells	Red Signal	Actual Pct Red Cells	Normalized Pct Red Cells
0.1 uM	100.49	0.91	114.07	83.85	0.04	-34.17
1 uM	92.85	0.77	32.28	75.87	0.04	-40.10
10 uM	101.67	0.93	433.43	79.90	0.03	-42.52

Compound	Normalized Values (A and B Sets)	Responses

PF-04620110

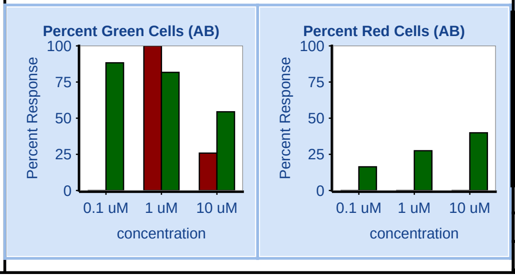

Average Response						
Dose	Green Signal	Actual Pct Green Cells	Normalized Pct Green Cells	Red Signal	Actual Pct Red Cells	Normalized Pct Red Cells
0.1 uM	96.82	0.88	36.37	96.77	0.11	-8.35
1 uM	97.49	0.85	94.37	99.86	0.11	-10.15
10 uM	94.14	0.81	40.14	96.18	0.11	-8.53

Compound	Normalized Values (A and B Sets)	Responses

PF-04691502

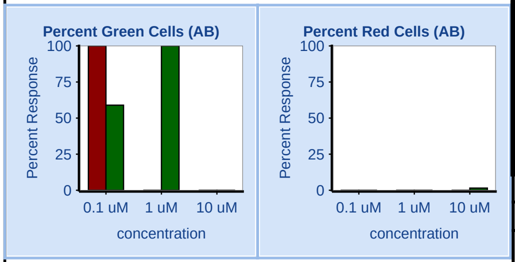

Average Response						
Dose	Green Signal	Actual Pct Green Cells	Normalized Pct Green Cells	Red Signal	Actual Pct Red Cells	Normalized Pct Red Cells
0.1 uM	91.53	0.88	84.47	70.24	0.02	-43.55
1 uM	88.21	0.79	56.58	96.60	0.07	-22.79
10 uM	96.14	0.80	-434.61	107.11	0.11	-8.05

Compound	Normalized Values (A and B Sets)	Responses
**PF-4708671**	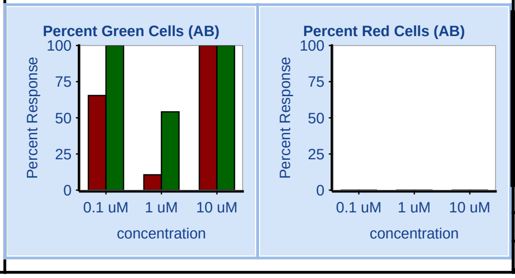	see table below

Average Response						
Dose	Green Signal	Actual Pct Green Cells	Normalized Pct Green Cells	Red Signal	Actual Pct Red Cells	Normalized Pct Red Cells
0.1 uM	91.98	0.90	105.42	73.98	0.03	-43.36
1 uM	87.40	0.76	32.36	72.45	0.02	-46.89
10 uM	98.85	0.92	136.56	83.66	0.05	-35.24

Compound	Normalized Values (A and B Sets)	Responses
**PF-04929113 (SNX-5422)**	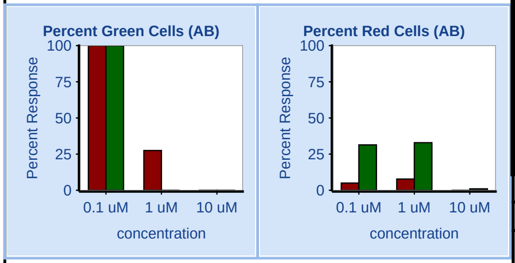	see table below

Average Response						
Dose	Green Signal	Actual Pct Green Cells	Normalized Pct Green Cells	Red Signal	Actual Pct Red Cells	Normalized Pct Red Cells
0.1 uM	121.17	0.95	152.78	101.85	0.15	18.17
1 uM	93.64	0.71	1.71	113.34	0.17	20.32
10 uM	96.37	0.81	-307.38	89.79	0.08	-20.02

Compound	Normalized Values (A and B Sets)	Responses
**PF-4981517**	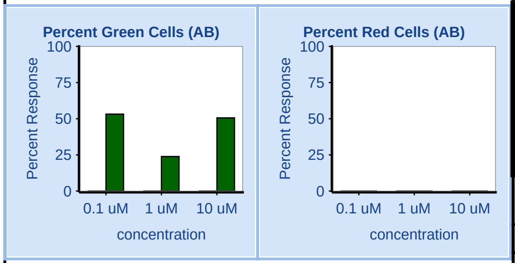	see table below

Average Response						
Dose	Green Signal	Actual Pct Green Cells	Normalized Pct Green Cells	Red Signal	Actual Pct Red Cells	Normalized Pct Red Cells
0.1 uM	86.00	0.81	-144.67	75.27	0.03	-49.84
1 uM	82.95	0.67	-31.38	63.44	0.01	-63.25
10 uM	91.69	0.79	21.09	97.35	0.08	-22.97

Compound	Normalized Values (A and B Sets)	Responses
**PF-05212384 (PKI-587)**	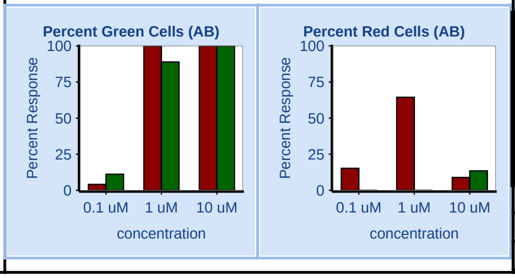	see table below

Average Response						
Dose	Green Signal	Actual Pct Green Cells	Normalized Pct Green Cells	Red Signal	Actual Pct Red Cells	Normalized Pct Red Cells
0.1 uM	87.21	0.80	7.62	99.07	0.11	-3.52
1 uM	113.30	0.91	106.38	115.56	0.19	28.49
10 uM	106.59	0.95	390.85	112.63	0.15	11.20

Compound	Normalized Values (A and B Sets)	Responses
**PF-5274857**	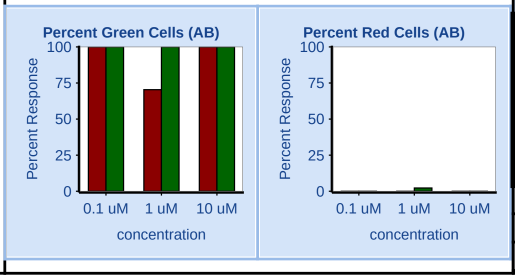	see table below

Average Response						
Dose	Green Signal	Actual Pct Green Cells	Normalized Pct Green Cells	Red Signal	Actual Pct Red Cells	Normalized Pct Red Cells
0.1 uM	107.33	0.95	152.69	80.08	0.05	-31.99
1 uM	106.81	0.88	98.53	97.67	0.10	-12.59
10 uM	109.71	0.94	452.52	90.43	0.07	-26.57

Compound | Normalized Values (A and B Sets) | Responses

PFI-1 (PF-6405761)

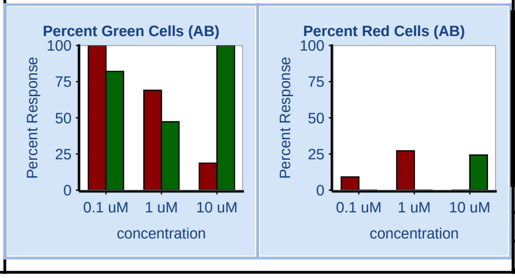

Average Response

Dose	Green Signal	Actual Pct Green Cells	Normalized Pct Green Cells	Red Signal	Actual Pct Red Cells	Normalized Pct Red Cells
0.1 uM	99.46	0.90	102.88	105.21	0.10	-23.60
1 uM	91.45	0.85	58.17	113.02	0.13	1.61
10 uM	92.92	0.82	66.34	115.27	0.12	1.02

Compound | Normalized Values (A and B Sets) | Responses

PH-797804

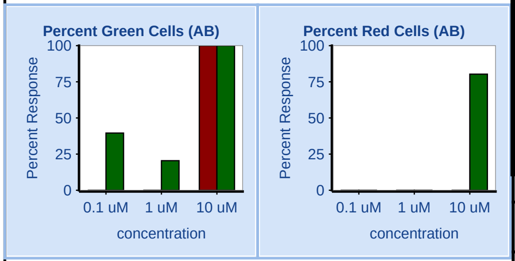

Average Response

Dose	Green Signal	Actual Pct Green Cells	Normalized Pct Green Cells	Red Signal	Actual Pct Red Cells	Normalized Pct Red Cells
0.1 uM	90.87	0.80	17.58	86.90	0.06	-34.96
1 uM	81.07	0.74	-693.84	88.10	0.08	-8.54
10 uM	108.86	0.97	146.33	96.66	0.18	24.55

Compound | Normalized Values (A and B Sets) | Responses

PHA-665752

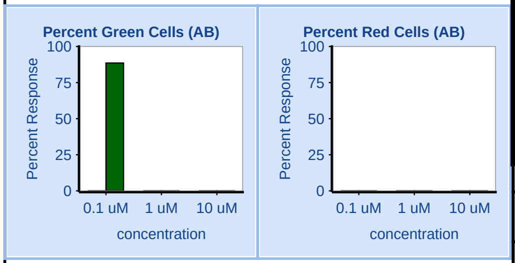

Average Response

Dose	Green Signal	Actual Pct Green Cells	Normalized Pct Green Cells	Red Signal	Actual Pct Red Cells	Normalized Pct Red Cells
0.1 uM	93.89	0.83	9.33	70.38	0.02	-64.62
1 uM	90.53	0.65	-152.39	77.56	0.02	-48.54
10 uM	4.08	0.02	-470.85	98.41	0.05	-33.62

Compound | Normalized Values (A and B Sets) | Responses

PHA-680632

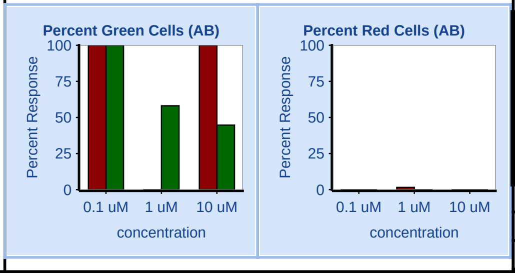

Average Response

Dose	Green Signal	Actual Pct Green Cells	Normalized Pct Green Cells	Red Signal	Actual Pct Red Cells	Normalized Pct Red Cells
0.1 uM	106.75	0.93	114.78	84.20	0.06	-36.10
1 uM	93.06	0.78	-49.74	108.62	0.13	-0.05
10 uM	97.77	0.86	84.67	96.36	0.06	-28.73

Compound | Normalized Values (A and B Sets) | Responses

PHA-793887

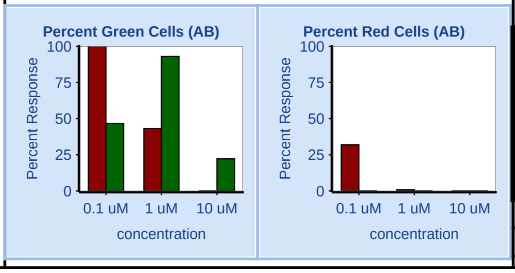

Average Response

Dose	Green Signal	Actual Pct Green Cells	Normalized Pct Green Cells	Red Signal	Actual Pct Red Cells	Normalized Pct Red Cells
0.1 uM	105.35	0.89	118.48	106.30	0.11	-21.34
1 uM	103.92	0.87	68.11	96.94	0.09	-16.71

Compound | Normalized Values (A and B Sets) | Responses

10 uM	81.45	0.57	-92.08	84.28	0.07	-18.16

Compound | Normalized Values (A and B Sets) | Responses

Phentolamine Mesylate

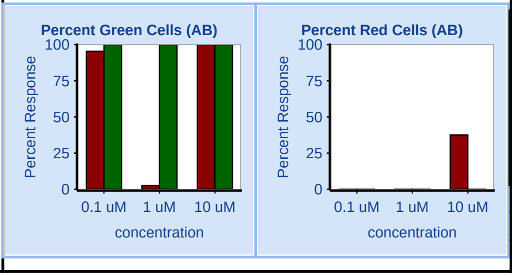

Average Response

Dose	Green Signal	Actual Pct Green Cells	Normalized Pct Green Cells	Red Signal	Actual Pct Red Cells	Normalized Pct Red Cells
0.1 uM	108.02	0.92	105.35	95.54	0.07	-32.24
1 uM	103.50	0.87	54.36	103.65	0.09	-16.73
10 uM	116.38	0.94	139.71	132.23	0.16	9.10

Compound | Normalized Values (A and B Sets) | Responses

Phenytoin sodium

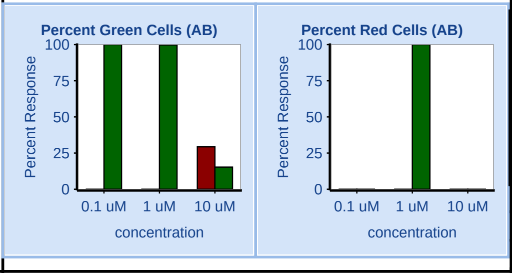

Average Response

Dose	Green Signal	Actual Pct Green Cells	Normalized Pct Green Cells	Red Signal	Actual Pct Red Cells	Normalized Pct Red Cells
0.1 uM	98.10	0.87	74.91	76.84	0.03	-56.56
1 uM	95.93	0.89	46.69	99.58	0.16	51.74
10 uM	88.60	0.84	22.30	72.50	0.02	-40.40

Compound | Normalized Values (A and B Sets) | Responses

PHT-427

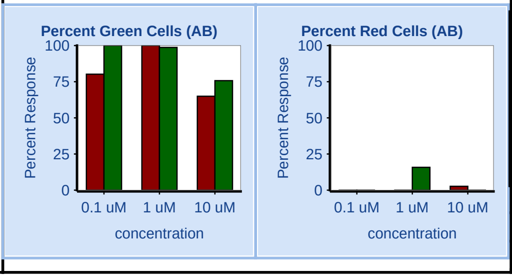

Average Response

Dose	Green Signal	Actual Pct Green Cells	Normalized Pct Green Cells	Red Signal	Actual Pct Red Cells	Normalized Pct Red Cells
0.1 uM	105.84	0.90	92.77	95.31	0.07	-38.65
1 uM	110.95	0.91	115.74	114.89	0.14	7.87
10 uM	97.17	0.83	70.35	108.42	0.09	-17.50

Compound | Normalized Values (A and B Sets) | Responses

Piceatannol

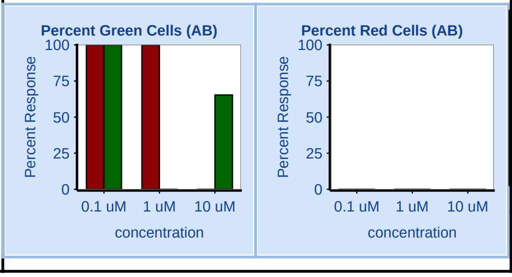

Average Response

Dose	Green Signal	Actual Pct Green Cells	Normalized Pct Green Cells	Red Signal	Actual Pct Red Cells	Normalized Pct Red Cells
0.1 uM	108.68	0.94	249.48	89.20	0.09	-20.96
1 uM	92.95	0.76	26.98	93.30	0.09	-20.08
10 uM	91.56	0.80	17.11	91.81	0.08	-20.95

Compound | Normalized Values (A and B Sets) | Responses

Picrotoxinin

Average Response

Dose	Green Signal	Actual Pct Green Cells	Normalized Pct Green Cells	Red Signal	Actual Pct Red Cells	Normalized Pct Red Cells

Compound	Normalized Values (A and B Sets)	Responses

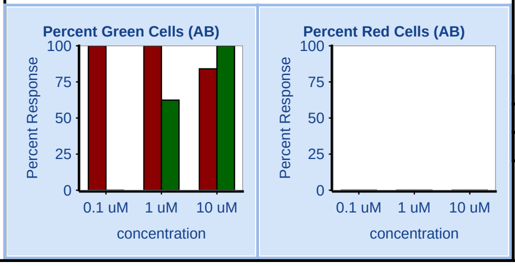

0.1 uM	86.24	0.76	30.64	86.37	0.07	-27.50
1 uM	99.23	0.85	93.19	95.09	0.09	-19.59
10 uM	99.81	0.88	97.33	73.17	0.03	-50.07

Compound	Normalized Values (A and B Sets)	Responses

Pifithrin-?

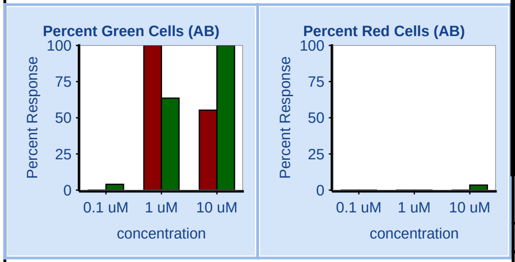

Average Response						
Dose	Green Signal	Actual Pct Green Cells	Normalized Pct Green Cells	Red Signal	Actual Pct Red Cells	Normalized Pct Red Cells
0.1 uM	76.04	0.65	-741.66	72.84	0.03	-50.87
1 uM	100.18	0.87	107.67	62.47	0.01	-63.53
10 uM	100.28	0.89	90.27	95.23	0.08	-21.53

Compound	Normalized Values (A and B Sets)	Responses

Pifithrin-? (PFT?)

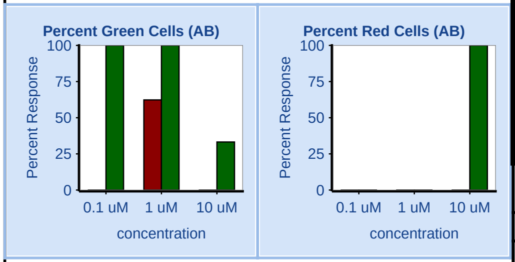

Average Response						
Dose	Green Signal	Actual Pct Green Cells	Normalized Pct Green Cells	Red Signal	Actual Pct Red Cells	Normalized Pct Red Cells
0.1 uM	90.06	0.85	-178.22	74.07	0.04	-45.32
1 uM	96.50	0.85	95.42	74.57	0.04	-48.93
10 uM	82.64	0.70	-105.15	116.13	0.22	49.22

Compound	Normalized Values (A and B Sets)	Responses

PIK-75

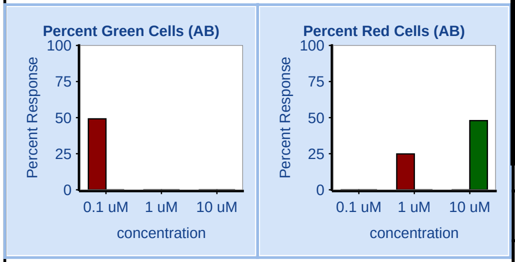

Average Response						
Dose	Green Signal	Actual Pct Green Cells	Normalized Pct Green Cells	Red Signal	Actual Pct Red Cells	Normalized Pct Red Cells
0.1 uM	100.01	0.80	23.01	74.39	0.05	-41.10
1 uM	86.65	0.71	-791.68	96.22	0.13	5.32
10 uM	82.92	0.65	-150.06	90.03	0.15	13.82

Compound	Normalized Values (A and B Sets)	Responses

PIK-93

Average Response						
Dose	Green Signal	Actual Pct Green Cells	Normalized Pct Green Cells	Red Signal	Actual Pct Red Cells	Normalized Pct Red Cells
0.1 uM	100.43	0.88	54.06	79.78	0.04	-54.41

Compound | Normalized Values (A and B Sets) | Responses

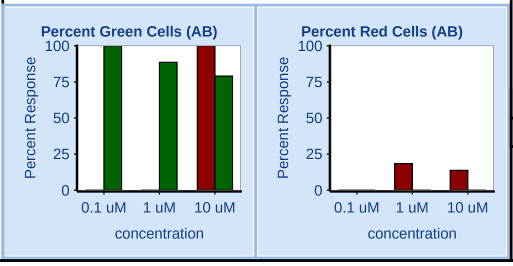

1 uM	94.35	0.82	-14.74	108.89	0.11	-6.19
10 uM	108.41	0.88	100.42	120.79	0.13	-1.51

Compound | Normalized Values (A and B Sets) | Responses

PIK-293

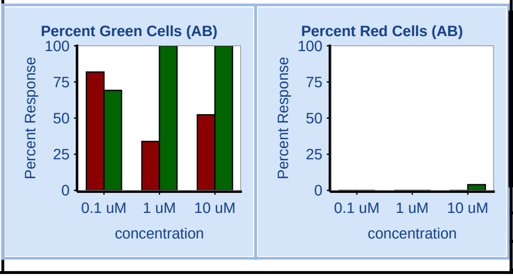

Average Response						
Dose	Green Signal	Actual Pct Green Cells	Normalized Pct Green Cells	Red Signal	Actual Pct Red Cells	Normalized Pct Red Cells
0.1 uM	89.89	0.87	75.53	73.09	0.03	-40.92
1 uM	96.04	0.82	69.38	85.96	0.07	-26.50
10 uM	90.27	0.90	85.43	101.52	0.10	-9.32

Compound | Normalized Values (A and B Sets) | Responses

PIK-294

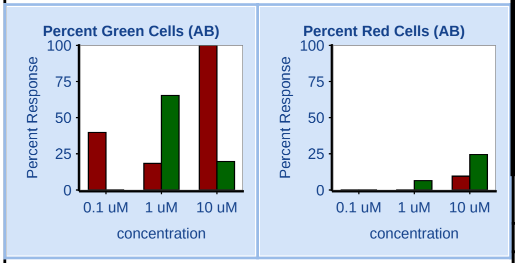

Average Response						
Dose	Green Signal	Actual Pct Green Cells	Normalized Pct Green Cells	Red Signal	Actual Pct Red Cells	Normalized Pct Red Cells
0.1 uM	73.64	0.64	-183.29	75.80	0.03	-42.90
1 uM	92.26	0.77	41.92	92.71	0.08	-20.14
10 uM	88.81	0.88	124.54	122.14	0.16	17.09

Compound | Normalized Values (A and B Sets) | Responses

PIM-1 Inhibitor2

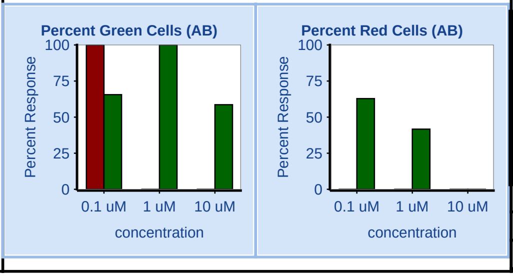

Average Response						
Dose	Green Signal	Actual Pct Green Cells	Normalized Pct Green Cells	Red Signal	Actual Pct Red Cells	Normalized Pct Red Cells
0.1 uM	109.14	0.90	114.71	95.60	0.13	1.30
1 uM	102.46	0.92	19.75	101.74	0.12	17.32
10 uM	88.84	0.82	7.65	77.38	0.04	-32.93

Compound | Normalized Values (A and B Sets) | Responses

Pimasertib (AS-703026)

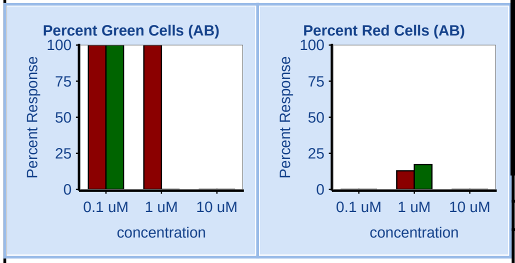

Average Response						
Dose	Green Signal	Actual Pct Green Cells	Normalized Pct Green Cells	Red Signal	Actual Pct Red Cells	Normalized Pct Red Cells
0.1 uM	111.81	0.95	145.94	94.63	0.06	-38.44
1 uM	89.66	0.80	47.01	120.17	0.16	15.07
10 uM	74.87	0.55	-103.42	64.41	0.03	-40.20

Compound | Normalized Values (A and B Sets) | Responses

Pimecrolimus

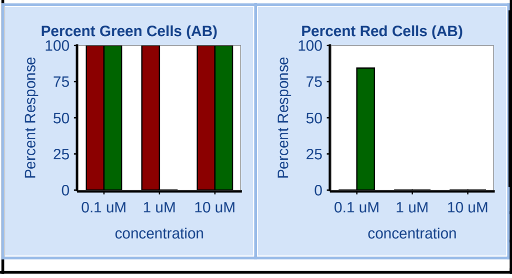

Average Response

Dose	Green Signal	Actual Pct Green Cells	Normalized Pct Green Cells	Red Signal	Actual Pct Red Cells	Normalized Pct Red Cells
0.1 uM	111.04	0.94	133.33	102.67	0.17	22.06
1 uM	81.93	0.63	-78.51	87.82	0.07	-30.49
10 uM	116.44	0.94	169.18	92.72	0.06	-30.07

Compound | Normalized Values (A and B Sets) | Responses

Pimobendan

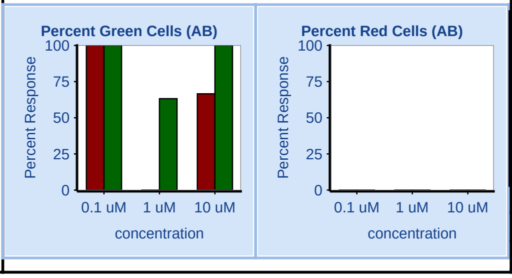

Average Response

Dose	Green Signal	Actual Pct Green Cells	Normalized Pct Green Cells	Red Signal	Actual Pct Red Cells	Normalized Pct Red Cells
0.1 uM	109.78	0.94	133.48	98.53	0.08	-33.66
1 uM	96.01	0.84	30.70	110.17	0.10	-13.45
10 uM	109.55	0.88	107.67	107.67	0.08	-16.36

Compound | Normalized Values (A and B Sets) | Responses

Pinacidil

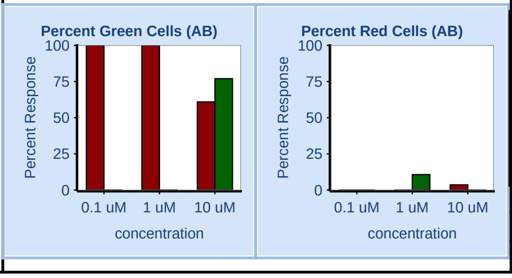

Average Response

Dose	Green Signal	Actual Pct Green Cells	Normalized Pct Green Cells	Red Signal	Actual Pct Red Cells	Normalized Pct Red Cells
0.1 uM	77.93	0.73	0.03	92.90	0.07	-26.56
1 uM	93.91	0.76	23.45	105.80	0.13	-0.89
10 uM	97.21	0.84	68.98	96.28	0.09	-16.53

Compound | Normalized Values (A and B Sets) | Responses

Pirarubicin

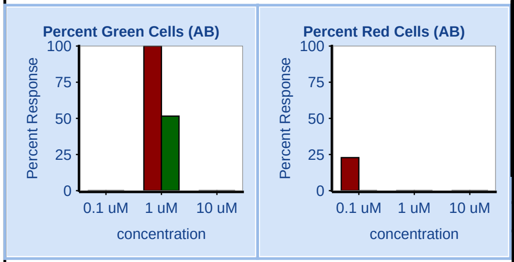

Average Response

Dose	Green Signal	Actual Pct Green Cells	Normalized Pct Green Cells	Red Signal	Actual Pct Red Cells	Normalized Pct Red Cells
0.1 uM	88.33	0.74	-24.91	102.74	0.12	-14.77
1 uM	101.00	0.88	114.40	11.72	0.02	-49.58
10 uM	79.55	0.49	-156.31	0.24	0.00	-52.84

Compound | Normalized Values (A and B Sets) | Responses

Pirfenidone

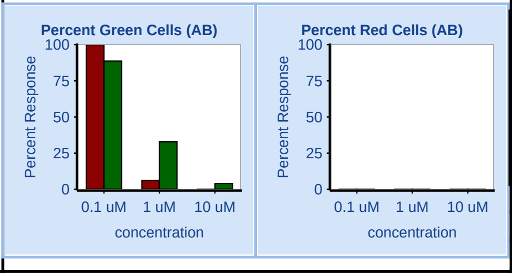

Average Response

Dose	Green Signal	Actual Pct Green Cells	Normalized Pct Green Cells	Red Signal	Actual Pct Red Cells	Normalized Pct Red Cells
0.1 uM	92.24	0.90	158.43	75.79	0.04	-43.86
1 uM	86.89	0.75	19.47	68.33	0.02	-58.42
10 uM	81.63	0.69	-84.44	61.43	0.02	-54.59

Compound | Normalized Values (A and B Sets) | Responses

Pitavastatin Calcium

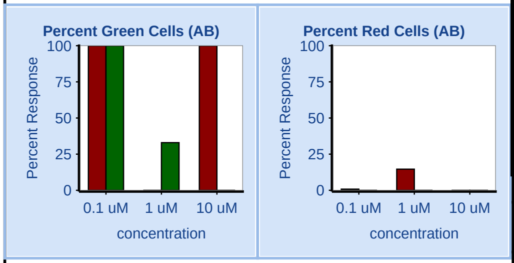

Average Response						
Dose	Green Signal	Actual Pct Green Cells	Normalized Pct Green Cells	Red Signal	Actual Pct Red Cells	Normalized Pct Red Cells
0.1 uM	106.76	0.93	133.56	91.13	0.08	-37.75
1 uM	95.75	0.65	-236.25	106.52	0.12	-2.56
10 uM	86.25	0.70	-37.56	93.29	0.05	-29.44

Compound | Normalized Values (A and B Sets) | Responses

PJ34

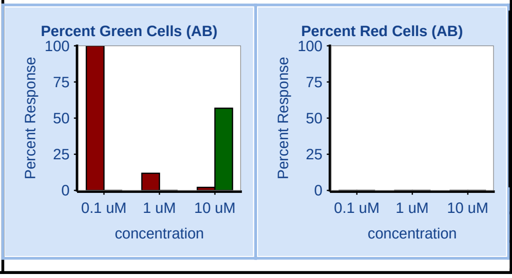

Average Response						
Dose	Green Signal	Actual Pct Green Cells	Normalized Pct Green Cells	Red Signal	Actual Pct Red Cells	Normalized Pct Red Cells
0.1 uM	88.44	0.82	85.13	63.73	0.02	-55.43
1 uM	82.67	0.70	-13.44	72.14	0.03	-54.94
10 uM	90.84	0.80	29.46	98.96	0.08	-19.45

Compound | Normalized Values (A and B Sets) | Responses

Plinabulin (NPI-2358)

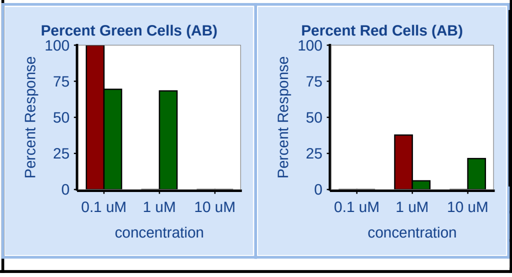

Average Response						
Dose	Green Signal	Actual Pct Green Cells	Normalized Pct Green Cells	Red Signal	Actual Pct Red Cells	Normalized Pct Red Cells
0.1 uM	119.70	0.89	98.16	91.02	0.08	-30.68
1 uM	108.86	0.78	-65.03	121.19	0.17	21.88
10 uM	93.88	0.65	-46.63	98.78	0.12	1.23

Compound | Normalized Values (A and B Sets) | Responses

PluriSIn #1 (NSC 14613)

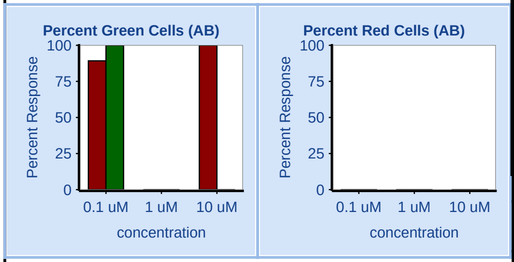

Average Response						
Dose	Green Signal	Actual Pct Green Cells	Normalized Pct Green Cells	Red Signal	Actual Pct Red Cells	Normalized Pct Red Cells
0.1 uM	97.23	0.90	108.06	66.95	0.02	-61.22
1 uM	81.05	0.75	-123.05	66.62	0.02	-34.80
10 uM	80.69	0.72	-80.14	76.59	0.03	-36.49

Compound | Normalized Values (A and B Sets) | Responses

PMSF

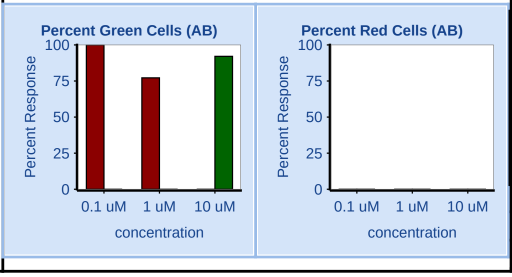

Average Response						
Dose	Green Signal	Actual Pct Green Cells	Normalized Pct Green Cells	Red Signal	Actual Pct Red Cells	Normalized Pct Red Cells
0.1 uM	87.82	0.80	129.95	86.07	0.05	-39.30
1 uM	90.36	0.76	29.72	82.28	0.05	-41.65
10 uM	92.83	0.81	5.71	61.48	0.01	-56.87

Compound | Normalized Values (A and B Sets) | Responses

PNU-120596

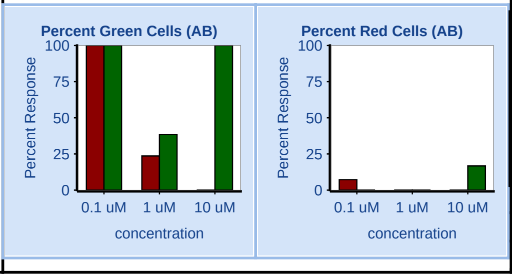

Average Response

Dose	Green Signal	Actual Pct Green Cells	Normalized Pct Green Cells	Red Signal	Actual Pct Red Cells	Normalized Pct Red Cells
0.1 uM	107.40	0.95	158.14	96.22	0.09	-13.12
1 uM	90.04	0.76	30.99	79.37	0.04	-39.02
10 uM	92.93	0.90	47.69	106.55	0.10	-11.56

Compound | Normalized Values (A and B Sets) | Responses

Pomalidomide

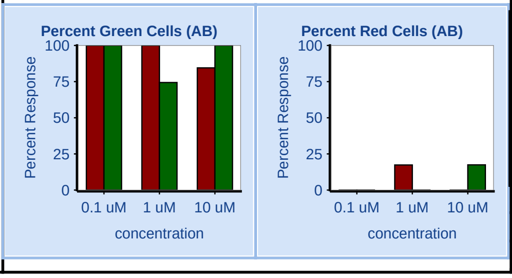

Average Response

Dose	Green Signal	Actual Pct Green Cells	Normalized Pct Green Cells	Red Signal	Actual Pct Red Cells	Normalized Pct Red Cells
0.1 uM	116.72	0.95	132.82	93.00	0.05	-45.70
1 uM	112.75	0.91	139.30	103.69	0.09	-14.96
10 uM	108.80	0.88	100.90	108.32	0.10	-7.09

Compound | Normalized Values (A and B Sets) | Responses

Ponatinib (AP24534)

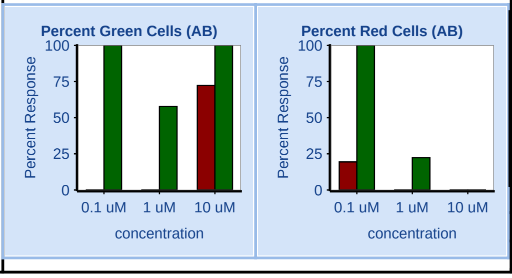

Average Response

Dose	Green Signal	Actual Pct Green Cells	Normalized Pct Green Cells	Red Signal	Actual Pct Red Cells	Normalized Pct Red Cells
0.1 uM	96.38	0.84	-5.20	139.01	0.26	69.46
1 uM	106.62	0.80	-25.68	99.93	0.14	7.03
10 uM	99.33	0.87	95.92	93.37	0.03	-39.36

Compound | Normalized Values (A and B Sets) | Responses

Posaconazole

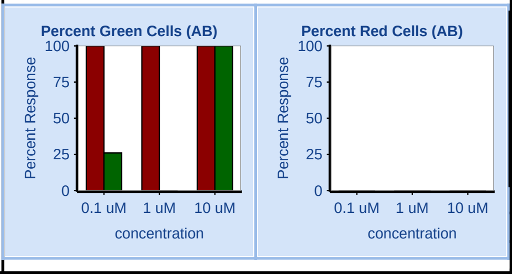

Average Response

Dose	Green Signal	Actual Pct Green Cells	Normalized Pct Green Cells	Red Signal	Actual Pct Red Cells	Normalized Pct Red Cells
0.1 uM	95.23	0.86	87.15	88.32	0.04	-54.26
1 uM	84.43	0.76	19.85	69.95	0.01	-52.51
10 uM	108.66	0.90	116.26	97.25	0.04	-38.03

Compound | Normalized Values (A and B Sets) | Responses

Potassium Iodide

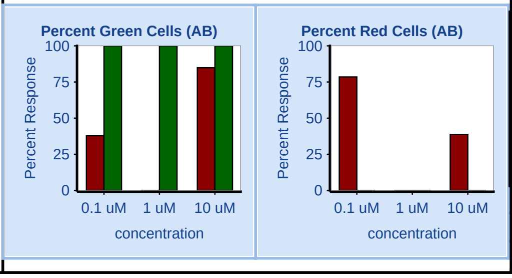

Average Response

Dose	Green Signal	Actual Pct Green Cells	Normalized Pct Green Cells	Red Signal	Actual Pct Red Cells	Normalized Pct Red Cells
0.1 uM	105.63	0.90	77.01	109.67	0.16	0.83
1 uM	99.58	0.83	-14.62	84.31	0.05	-35.61
10 uM	106.96	0.88	102.71	115.84	0.14	-0.53

Compound | Normalized Values (A and B Sets) | Responses

PP1

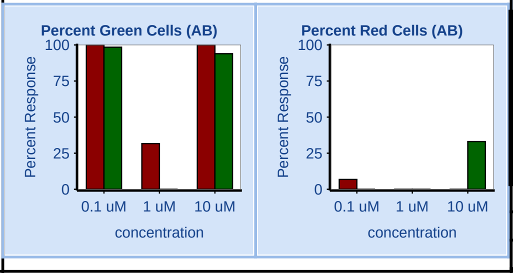

Average Response						
Dose	Green Signal	Actual Pct Green Cells	Normalized Pct Green Cells	Red Signal	Actual Pct Red Cells	Normalized Pct Red Cells
0.1 uM	99.79	0.91	180.25	97.44	0.11	-7.07
1 uM	83.10	0.68	-34.58	80.17	0.05	-41.99
10 uM	98.80	0.88	108.84	95.18	0.10	-9.70

Compound | Normalized Values (A and B Sets) | Responses

PP2

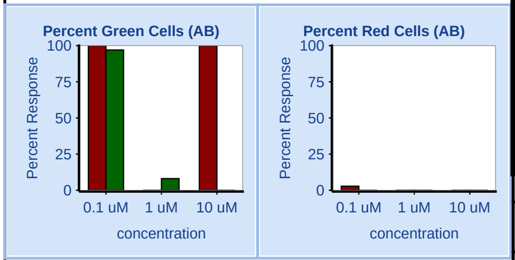

Average Response						
Dose	Green Signal	Actual Pct Green Cells	Normalized Pct Green Cells	Red Signal	Actual Pct Red Cells	Normalized Pct Red Cells
0.1 uM	93.06	0.89	104.86	104.02	0.10	-9.43
1 uM	86.58	0.82	-158.10	91.89	0.08	-7.77
10 uM	89.23	0.86	49.02	78.32	0.05	-30.89

Compound | Normalized Values (A and B Sets) | Responses

PP242

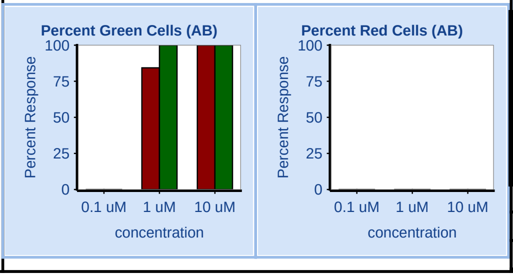

Average Response						
Dose	Green Signal	Actual Pct Green Cells	Normalized Pct Green Cells	Red Signal	Actual Pct Red Cells	Normalized Pct Red Cells
0.1 uM	82.06	0.77	-30.63	75.93	0.03	-41.98
1 uM	97.40	0.89	102.03	79.92	0.03	-40.69
10 uM	101.12	0.95	460.03	82.24	0.03	-44.55

Compound | Normalized Values (A and B Sets) | Responses

Pracinostat (SB939)

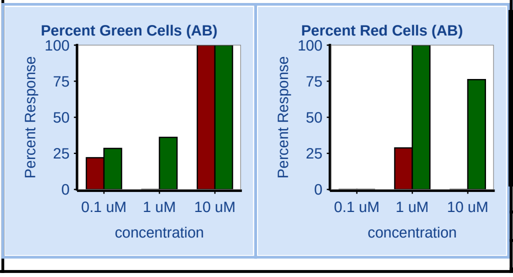

Average Response						
Dose	Green Signal	Actual Pct Green Cells	Normalized Pct Green Cells	Red Signal	Actual Pct Red Cells	Normalized Pct Red Cells
0.1 uM	93.29	0.81	25.22	89.87	0.06	-43.99
1 uM	105.67	0.81	5.93	142.80	0.27	66.37
10 uM	120.57	0.92	126.37	136.48	0.19	34.85

Compound | Normalized Values (A and B Sets) | Responses

Pramipexole 2HCl Monohydrate

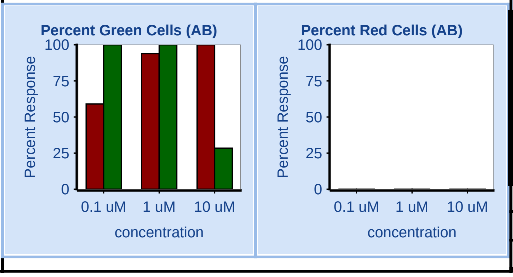

Average Response						
Dose	Green Signal	Actual Pct Green Cells	Normalized Pct Green Cells	Red Signal	Actual Pct Red Cells	Normalized Pct Red Cells
0.1 uM	101.29	0.90	87.38	97.37	0.07	-35.17
1 uM	107.18	0.90	99.62	95.22	0.09	-15.66
10 uM	109.30	0.85	77.70	108.34	0.09	-13.26

Compound | Normalized Values (A and B Sets) | Responses

Pramiracetam

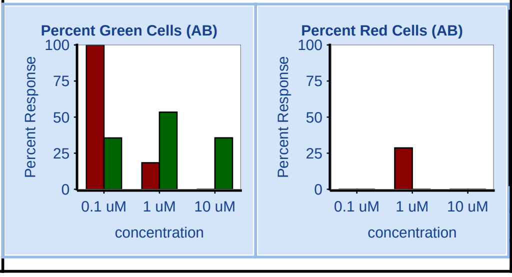

Average Response

Dose	Green Signal	Actual Pct Green Cells	Normalized Pct Green Cells	Red Signal	Actual Pct Red Cells	Normalized Pct Red Cells
0.1 uM	101.52	0.90	98.00	78.98	0.04	-35.19
1 uM	90.84	0.76	35.92	100.89	0.12	-3.97
10 uM	95.63	0.86	-63.11	84.81	0.05	-35.20

Compound | Normalized Values (A and B Sets) | Responses

Pranlukast

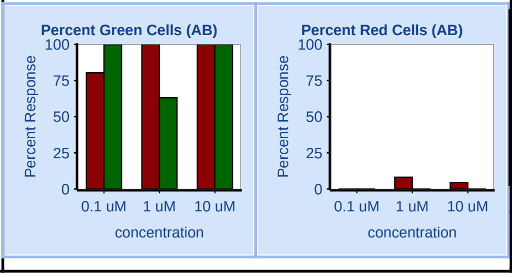

Average Response

Dose	Green Signal	Actual Pct Green Cells	Normalized Pct Green Cells	Red Signal	Actual Pct Red Cells	Normalized Pct Red Cells
0.1 uM	98.81	0.90	90.17	93.79	0.05	-44.42
1 uM	104.93	0.87	82.64	98.06	0.09	-18.48
10 uM	109.90	0.89	108.28	111.31	0.09	-18.44

Compound | Normalized Values (A and B Sets) | Responses

Prasugrel

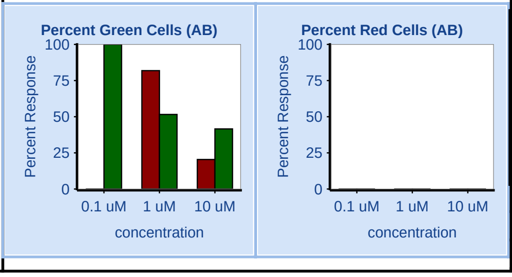

Average Response

Dose	Green Signal	Actual Pct Green Cells	Normalized Pct Green Cells	Red Signal	Actual Pct Red Cells	Normalized Pct Red Cells
0.1 uM	99.00	0.89	55.80	82.50	0.02	-58.67
1 uM	97.05	0.85	66.72	74.70	0.02	-48.96
10 uM	85.86	0.77	30.99	86.38	0.03	-43.19

Compound | Normalized Values (A and B Sets) | Responses

Prednisolone Acetate

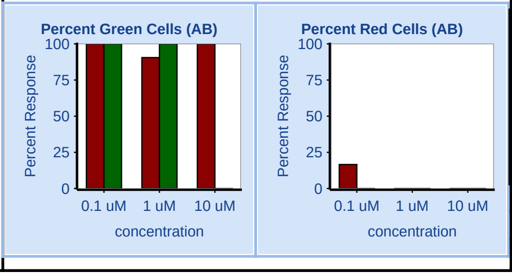

Average Response

Dose	Green Signal	Actual Pct Green Cells	Normalized Pct Green Cells	Red Signal	Actual Pct Red Cells	Normalized Pct Red Cells
0.1 uM	102.05	0.94	146.48	90.68	0.09	-16.92
1 uM	100.82	0.89	101.85	72.80	0.02	-47.37
10 uM	93.07	0.84	210.34	76.02	0.03	-46.17

Compound | Normalized Values (A and B Sets) | Responses

Prilocaine

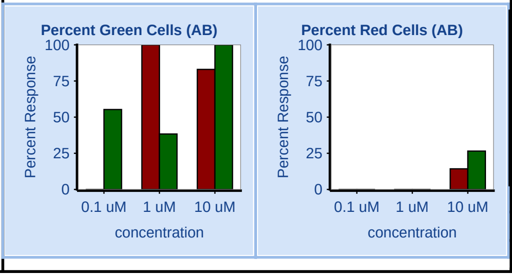

Average Response

Dose	Green Signal	Actual Pct Green Cells	Normalized Pct Green Cells	Red Signal	Actual Pct Red Cells	Normalized Pct Red Cells
0.1 uM	87.10	0.78	-27.12	81.58	0.03	-58.43
1 uM	109.11	0.88	112.54	93.32	0.05	-33.38
10 uM	111.42	0.87	100.12	130.41	0.17	20.39

Compound	Normalized Values (A and B Sets)	Responses

Procodazole

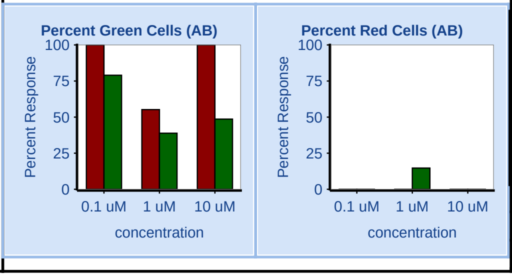

Average Response

Dose	Green Signal	Actual Pct Green Cells	Normalized Pct Green Cells	Red Signal	Actual Pct Red Cells	Normalized Pct Red Cells
0.1 uM	99.66	0.89	127.00	74.09	0.02	-53.09
1 uM	96.11	0.79	47.03	105.78	0.13	0.13
10 uM	96.36	0.84	95.63	80.12	0.04	-41.06

Compound	Normalized Values (A and B Sets)	Responses

Propafenone HCl

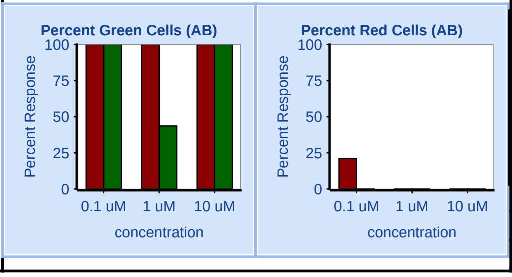

Average Response

Dose	Green Signal	Actual Pct Green Cells	Normalized Pct Green Cells	Red Signal	Actual Pct Red Cells	Normalized Pct Red Cells
0.1 uM	109.55	0.92	124.00	98.78	0.11	-4.94
1 uM	110.24	0.86	75.35	82.95	0.06	-31.31
10 uM	104.21	0.93	386.27	86.23	0.05	-35.91

Compound	Normalized Values (A and B Sets)	Responses

PRT062607 (P505-15, BIIB057) HCl

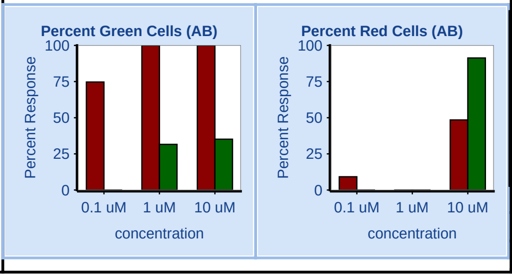

Average Response

Dose	Green Signal	Actual Pct Green Cells	Normalized Pct Green Cells	Red Signal	Actual Pct Red Cells	Normalized Pct Red Cells
0.1 uM	79.35	0.72	-46.86	97.98	0.08	-23.98
1 uM	94.61	0.87	124.46	88.36	0.07	-8.47
10 uM	101.69	0.91	95.79	120.16	0.27	69.91

Compound	Normalized Values (A and B Sets)	Responses

Prucalopride

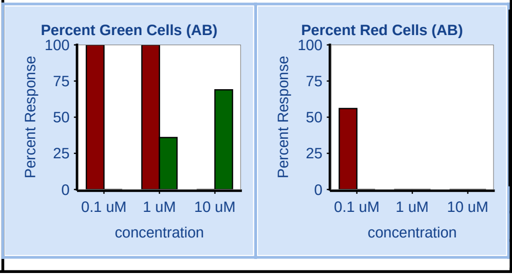

Average Response

Dose	Green Signal	Actual Pct Green Cells	Normalized Pct Green Cells	Red Signal	Actual Pct Red Cells	Normalized Pct Red Cells
0.1 uM	91.71	0.85	192.01	97.75	0.12	-2.64
1 uM	93.45	0.85	93.13	92.28	0.05	-41.96
10 uM	88.68	0.80	12.35	63.31	0.01	-59.72

Compound	Normalized Values (A and B Sets)	Responses

Prucalopride Succinat

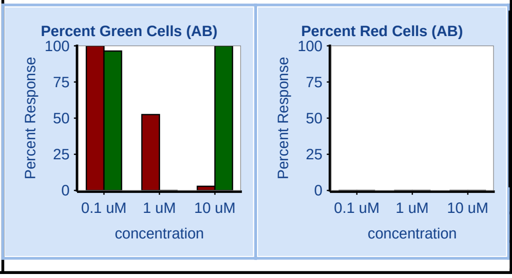

Average Response

Dose	Green Signal	Actual Pct Green Cells	Normalized Pct Green Cells	Red Signal	Actual Pct Red Cells	Normalized Pct Red Cells
0.1 uM	97.63	0.92	188.61	80.81	0.04	-45.82
1 uM	87.89	0.75	18.25	86.44	0.05	-39.62
10 uM	96.67	0.86	58.41	80.61	0.04	-43.36

Compound | Normalized Values (A and B Sets) | Responses

PRX-08066 Maleic acid

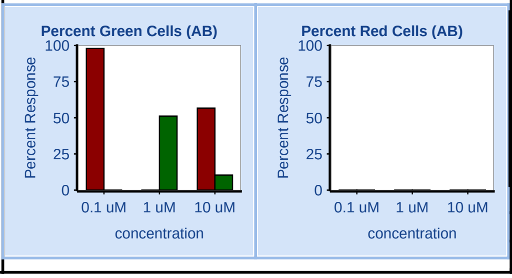

Average Response

Dose	Green Signal	Actual Pct Green Cells	Normalized Pct Green Cells	Red Signal	Actual Pct Red Cells	Normalized Pct Red Cells
0.1 uM	91.65	0.80	21.82	82.03	0.05	-45.75
1 uM	92.45	0.86	-20.14	61.72	0.01	-42.32
10 uM	86.47	0.85	33.57	61.33	0.01	-46.87

Compound | Normalized Values (A and B Sets) | Responses

Puerarin

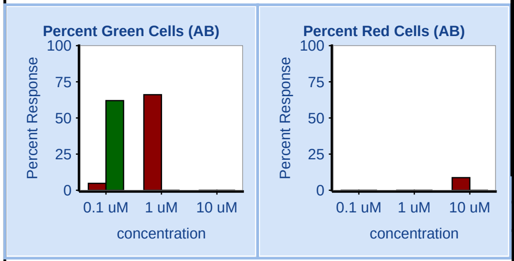

Average Response

Dose	Green Signal	Actual Pct Green Cells	Normalized Pct Green Cells	Red Signal	Actual Pct Red Cells	Normalized Pct Red Cells
0.1 uM	84.39	0.83	33.39	95.09	0.08	-18.37
1 uM	88.81	0.75	17.71	86.06	0.06	-27.84
10 uM	86.43	0.81	-125.03	105.34	0.11	-5.37

Compound | Normalized Values (A and B Sets) | Responses

Purmorphamine

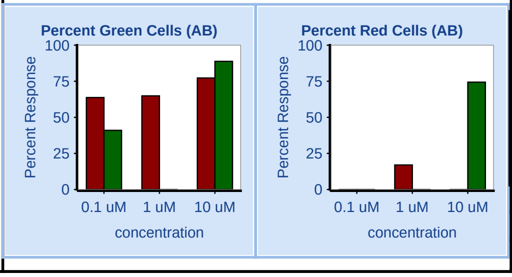

Average Response

Dose	Green Signal	Actual Pct Green Cells	Normalized Pct Green Cells	Red Signal	Actual Pct Red Cells	Normalized Pct Red Cells
0.1 uM	91.75	0.85	52.37	97.35	0.12	-4.89
1 uM	87.49	0.71	-15.03	99.67	0.13	-1.67
10 uM	110.15	0.86	83.04	106.05	0.17	25.60

Compound | Normalized Values (A and B Sets) | Responses

PYR-41

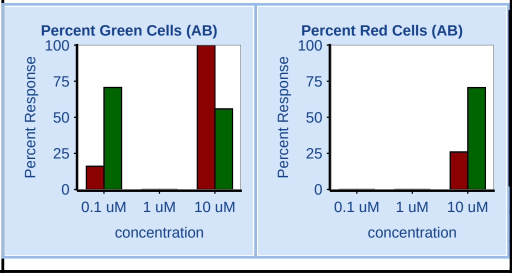

Average Response

Dose	Green Signal	Actual Pct Green Cells	Normalized Pct Green Cells	Red Signal	Actual Pct Red Cells	Normalized Pct Red Cells
0.1 uM	98.28	0.87	43.38	93.31	0.10	-14.87
1 uM	76.65	0.61	-77.39	87.46	0.06	-35.97
10 uM	108.01	0.85	107.69	123.68	0.21	48.31

Compound | Normalized Values (A and B Sets) | Responses

Pyrilamine Maleate

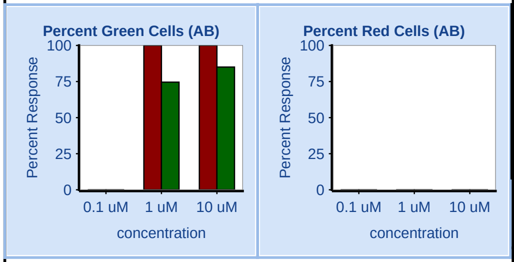

Average Response

Dose	Green Signal	Actual Pct Green Cells	Normalized Pct Green Cells	Red Signal	Actual Pct Red Cells	Normalized Pct Red Cells
0.1 uM	66.93	0.55	-1155.69	84.58	0.06	-32.42
1 uM	98.63	0.88	110.79	94.79	0.06	-34.78

Compound	Normalized Values (A and B Sets)	Responses						
		10 uM	104.93	0.87	101.17	75.28	0.03	-45.62

Compound	Normalized Values (A and B Sets)	Responses						
**Pyroxamide**	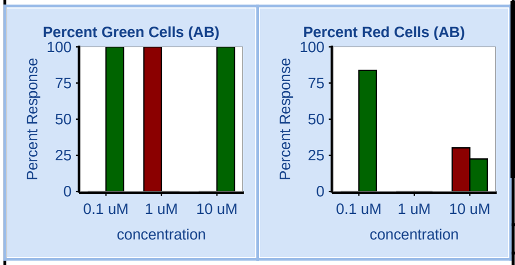	Average Response						
		Dose	Green Signal	Actual Pct Green Cells	Normalized Pct Green Cells	Red Signal	Actual Pct Red Cells	Normalized Pct Red Cells
		0.1 uM	83.05	0.66	-122.05	99.58	0.13	5.15
		1 uM	91.68	0.81	85.80	63.16	0.01	-45.61
		10 uM	91.81	0.87	55.68	114.49	0.17	26.26

Compound	Normalized Values (A and B Sets)	Responses						
**QNZ**	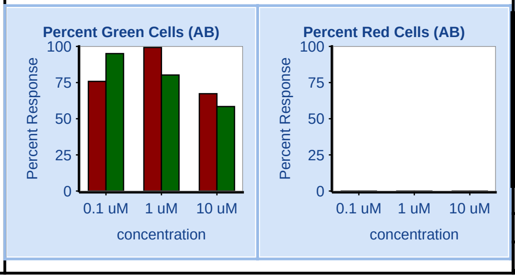	Average Response						
		Dose	Green Signal	Actual Pct Green Cells	Normalized Pct Green Cells	Red Signal	Actual Pct Red Cells	Normalized Pct Red Cells
		0.1 uM	93.57	0.87	85.46	82.37	0.05	-45.94
		1 uM	93.31	0.89	89.77	61.77	0.01	-43.32
		10 uM	89.86	0.88	62.89	59.09	0.01	-47.49

Compound	Normalized Values (A and B Sets)	Responses						
**Quercetin Dihydrate**	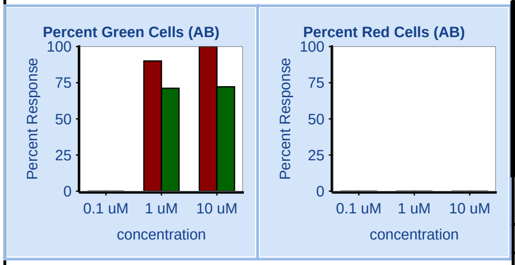	Average Response						
		Dose	Green Signal	Actual Pct Green Cells	Normalized Pct Green Cells	Red Signal	Actual Pct Red Cells	Normalized Pct Red Cells
		0.1 uM	77.08	0.69	-121.08	72.65	0.02	-43.65
		1 uM	99.86	0.86	80.62	77.77	0.03	-41.82
		10 uM	97.27	0.90	166.36	81.08	0.03	-45.59

Compound	Normalized Values (A and B Sets)	Responses						
**Quetiapine Fumarate**	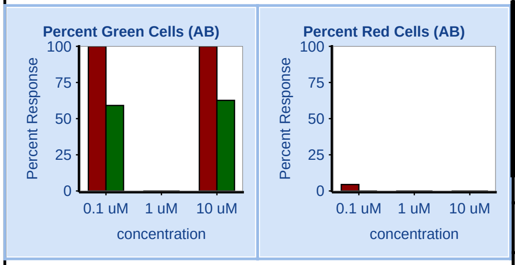	Average Response						
		Dose	Green Signal	Actual Pct Green Cells	Normalized Pct Green Cells	Red Signal	Actual Pct Red Cells	Normalized Pct Red Cells
		0.1 uM	98.50	0.88	92.84	97.58	0.08	-37.16
		1 uM	69.74	0.50	-409.85	98.73	0.08	-22.86
		10 uM	103.46	0.89	101.33	94.41	0.05	-28.01

Compound	Normalized Values (A and B Sets)	Responses						
**Quinine HCl Dihydrate**		Average Response						
		Dose	Green Signal			Red Signal	Actual Pct Red Cells	

Compound	Normalized Values (A and B Sets)	Responses
	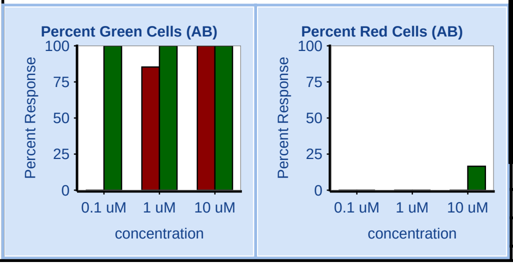	(table below)

		Actual Pct Green Cells	Normalized Pct Green Cells			Normalized Pct Red Cells
0.1 uM	98.84	0.84	50.80	94.32	0.09	-12.20
1 uM	109.24	0.88	96.13	88.17	0.08	-21.70
10 uM	101.29	0.91	205.14	100.53	0.10	-12.48

Compound	Normalized Values (A and B Sets)	Responses
**Quizartinib (AC220)**	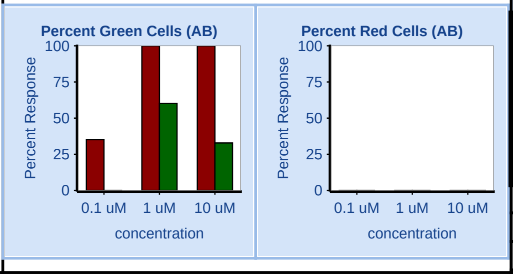	(table below)

Average Response						
Dose	Green Signal	Actual Pct Green Cells	Normalized Pct Green Cells	Red Signal	Actual Pct Red Cells	Normalized Pct Red Cells
0.1 uM	87.86	0.78	14.07	91.32	0.07	-41.94
1 uM	104.26	0.88	101.29	81.19	0.03	-44.42
10 uM	100.33	0.86	83.85	104.39	0.07	-25.25

Compound	Normalized Values (A and B Sets)	Responses
**R-(-)-Apomorphine HCl Hemihydrate**	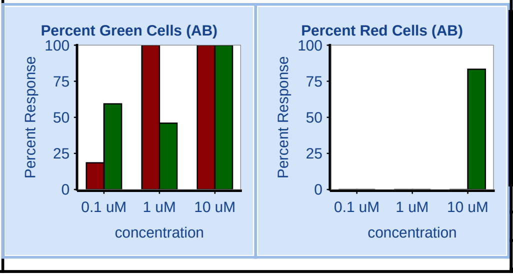	(table below)

Average Response						
Dose	Green Signal	Actual Pct Green Cells	Normalized Pct Green Cells	Red Signal	Actual Pct Red Cells	Normalized Pct Red Cells
0.1 uM	96.94	0.86	38.93	90.43	0.09	-19.66
1 uM	100.42	0.84	86.98	95.72	0.09	-19.18
10 uM	114.10	0.91	127.80	109.63	0.18	29.18

Compound	Normalized Values (A and B Sets)	Responses
**R406**	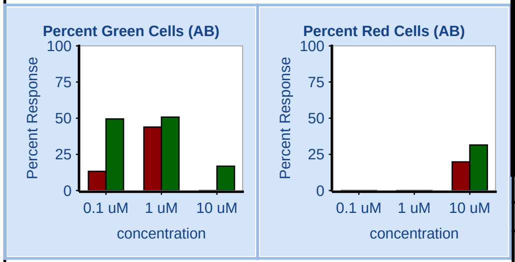	(table below)

Average Response						
Dose	Green Signal	Actual Pct Green Cells	Normalized Pct Green Cells	Red Signal	Actual Pct Red Cells	Normalized Pct Red Cells
0.1 uM	87.69	0.83	31.38	80.15	0.05	-31.25
1 uM	90.75	0.79	47.32	72.47	0.02	-45.56
10 uM	104.59	0.82	-396.97	108.54	0.18	25.64

Compound	Normalized Values (A and B Sets)	Responses
**R406 (free base)**	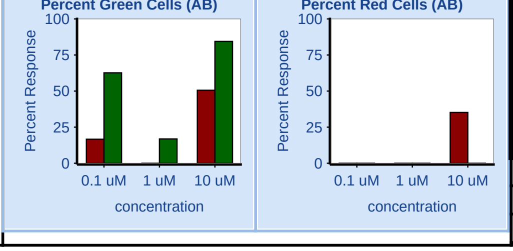	(table below)

Average Response						
Dose	Green Signal	Actual Pct Green Cells	Normalized Pct Green Cells	Red Signal	Actual Pct Red Cells	Normalized Pct Red Cells
0.1 uM	96.10	0.84	39.65	86.13	0.04	-50.31
1 uM	96.84	0.79	-0.16	99.34	0.08	-19.28
10 uM	104.26	0.82	67.48	125.73	0.17	17.52

Compound	Normalized Values (A and B Sets)	Responses
**R428 (BGB324)**	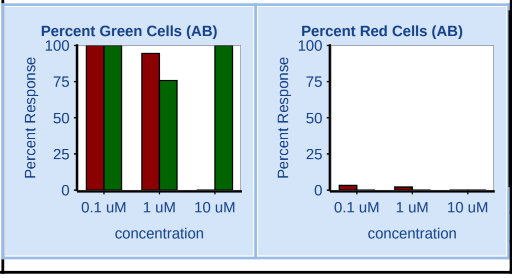	see table below

Average Response						
Dose	Green Signal	Actual Pct Green Cells	Normalized Pct Green Cells	Red Signal	Actual Pct Red Cells	Normalized Pct Red Cells
0.1 uM	101.97	0.92	122.11	90.47	0.08	-19.60
1 uM	99.41	0.87	85.12	96.32	0.08	-21.83
10 uM	80.35	0.88	-42.67	81.74	0.04	-38.03

Compound	Normalized Values (A and B Sets)	Responses
**R547**	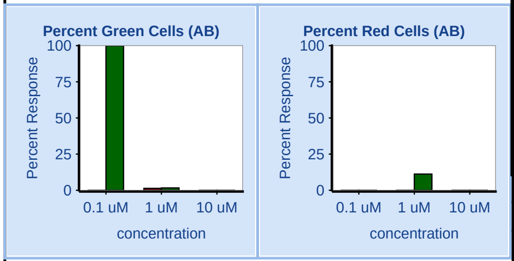	see table below

Average Response						
Dose	Green Signal	Actual Pct Green Cells	Normalized Pct Green Cells	Red Signal	Actual Pct Red Cells	Normalized Pct Red Cells
0.1 uM	93.04	0.86	71.55	62.97	0.02	-47.27
1 uM	85.54	0.70	1.47	99.58	0.11	-7.69
10 uM	88.64	0.77	-393.16	87.92	0.04	-37.52

Compound	Normalized Values (A and B Sets)	Responses
**Ractopamine HCl**	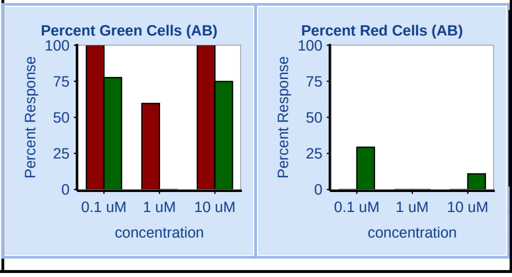	see table below

Average Response						
Dose	Green Signal	Actual Pct Green Cells	Normalized Pct Green Cells	Red Signal	Actual Pct Red Cells	Normalized Pct Red Cells
0.1 uM	98.54	0.89	130.69	95.63	0.12	-1.37
1 uM	79.80	0.66	-51.21	84.70	0.04	-45.91
10 uM	104.04	0.88	134.38	97.91	0.09	-18.39

Compound	Normalized Values (A and B Sets)	Responses
**RAF265 (CHIR-265)**	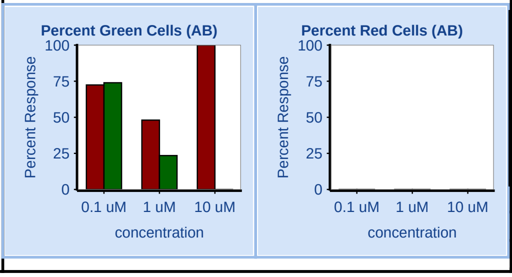	see table below

Average Response						
Dose	Green Signal	Actual Pct Green Cells	Normalized Pct Green Cells	Red Signal	Actual Pct Red Cells	Normalized Pct Red Cells
0.1 uM	90.73	0.87	73.18	71.33	0.02	-45.85
1 uM	85.21	0.77	35.78	81.71	0.04	-38.15
10 uM	96.74	0.86	162.65	90.71	0.10	-9.66

Compound	Normalized Values (A and B Sets)	Responses
**Raf265 derivative**	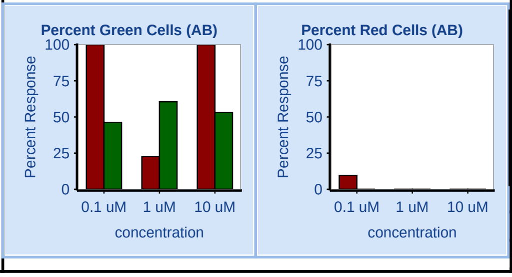	see table below

Average Response						
Dose	Green Signal	Actual Pct Green Cells	Normalized Pct Green Cells	Red Signal	Actual Pct Red Cells	Normalized Pct Red Cells
0.1 uM	97.24	0.89	92.70	105.26	0.12	-0.62
1 uM	88.33	0.78	41.58	74.70	0.02	-45.48
10 uM	91.39	0.90	198.19	81.92	0.05	-33.07

Compound | Normalized Values (A and B Sets) | Responses

Raltegravir (MK-0518)

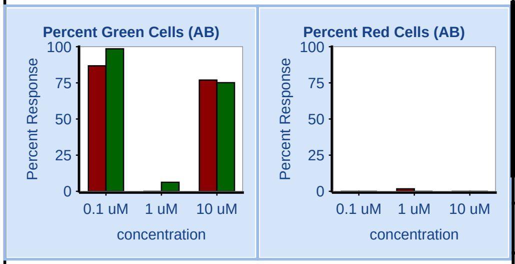

Average Response

Dose	Green Signal	Actual Pct Green Cells	Normalized Pct Green Cells	Red Signal	Actual Pct Red Cells	Normalized Pct Red Cells
0.1 uM	104.41	0.90	92.67	93.10	0.07	-34.36
1 uM	86.18	0.72	-109.34	95.05	0.08	-19.09
10 uM	99.55	0.84	76.04	94.24	0.05	-32.19

Compound | Normalized Values (A and B Sets) | Responses

Raltitrexed

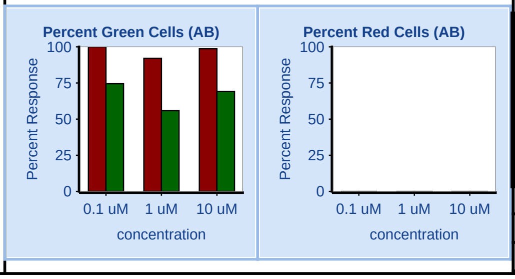

Average Response

Dose	Green Signal	Actual Pct Green Cells	Normalized Pct Green Cells	Red Signal	Actual Pct Red Cells	Normalized Pct Red Cells
0.1 uM	100.89	0.88	90.45	83.82	0.03	-57.47
1 uM	106.11	0.86	73.92	89.22	0.05	-32.81
10 uM	103.95	0.86	83.88	109.11	0.10	-10.43

Compound | Normalized Values (A and B Sets) | Responses

Ranitidine

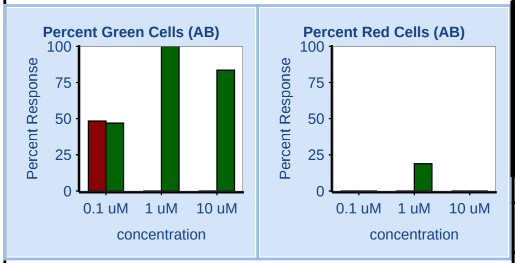

Average Response

Dose	Green Signal	Actual Pct Green Cells	Normalized Pct Green Cells	Red Signal	Actual Pct Red Cells	Normalized Pct Red Cells
0.1 uM	94.17	0.84	47.69	89.33	0.06	-41.96
1 uM	94.12	0.77	-115.48	108.27	0.11	-6.25
10 uM	93.83	0.78	38.65	86.36	0.03	-37.54

Compound | Normalized Values (A and B Sets) | Responses

Ranolazine 2HCl

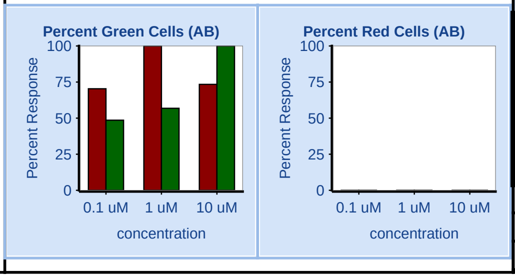

Average Response

Dose	Green Signal	Actual Pct Green Cells	Normalized Pct Green Cells	Red Signal	Actual Pct Red Cells	Normalized Pct Red Cells
0.1 uM	95.82	0.85	59.47	99.07	0.08	-33.43
1 uM	102.97	0.87	90.96	89.14	0.05	-35.60
10 uM	102.54	0.86	91.44	100.32	0.05	-30.86

Compound | Normalized Values (A and B Sets) | Responses

Rasagiline Mesylate

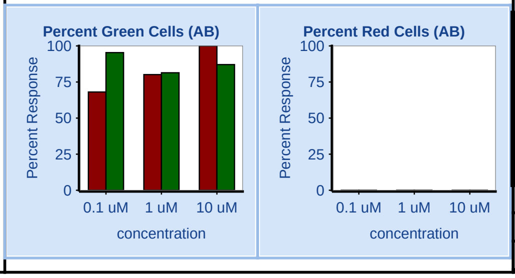

Average Response

Dose	Green Signal	Actual Pct Green Cells	Normalized Pct Green Cells	Red Signal	Actual Pct Red Cells	Normalized Pct Red Cells
0.1 uM	112.76	0.89	81.73	100.27	0.10	-19.52
1 uM	107.06	0.88	80.78	97.50	0.08	-22.24
10 uM	103.89	0.87	95.97	103.16	0.07	-23.63

Compound	Normalized Values (A and B Sets)	Responses

Reboxetine mesylate

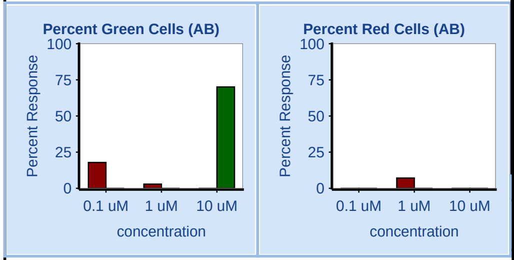

Average Response						
Dose	Green Signal	Actual Pct Green Cells	Normalized Pct Green Cells	Red Signal	Actual Pct Red Cells	Normalized Pct Red Cells
0.1 uM	78.74	0.74	-47.37	75.91	0.03	-48.74
1 uM	71.46	0.52	-151.44	93.78	0.08	-24.62
10 uM	90.68	0.80	16.79	80.20	0.04	-44.62

Compound	Normalized Values (A and B Sets)	Responses

Regorafenib (BAY 73-4506)

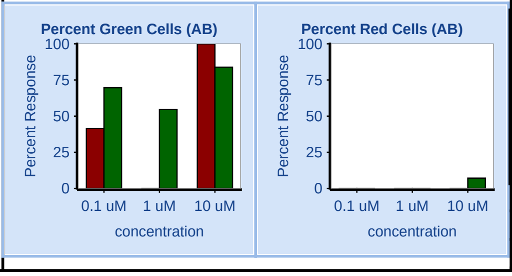

Average Response						
Dose	Green Signal	Actual Pct Green Cells	Normalized Pct Green Cells	Red Signal	Actual Pct Red Cells	Normalized Pct Red Cells
0.1 uM	97.23	0.86	55.51	81.16	0.04	-53.18
1 uM	96.24	0.80	-20.98	98.44	0.10	-12.83
10 uM	96.04	0.87	95.41	122.03	0.13	1.32

Compound	Normalized Values (A and B Sets)	Responses

Resminostat

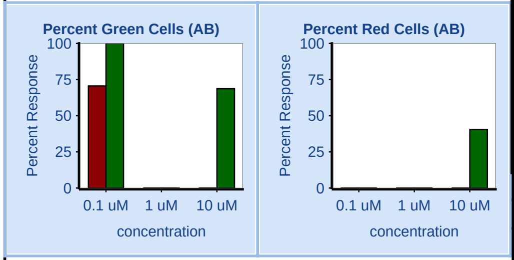

Average Response						
Dose	Green Signal	Actual Pct Green Cells	Normalized Pct Green Cells	Red Signal	Actual Pct Red Cells	Normalized Pct Red Cells
0.1 uM	99.00	0.89	100.30	81.67	0.06	-24.26
1 uM	84.08	0.67	-13.60	74.49	0.04	-39.69
10 uM	106.25	0.80	-608.36	115.37	0.14	6.08

Compound	Normalized Values (A and B Sets)	Responses

Retapamulin

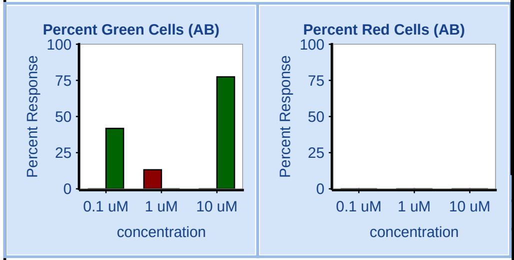

Average Response						
Dose	Green Signal	Actual Pct Green Cells	Normalized Pct Green Cells	Red Signal	Actual Pct Red Cells	Normalized Pct Red Cells
0.1 uM	86.71	0.83	-59.72	67.78	0.01	-57.53
1 uM	79.49	0.63	-68.47	67.54	0.01	-62.72
10 uM	80.13	0.69	-172.54	87.35	0.03	-47.07

Compound	Normalized Values (A and B Sets)	Responses

RG108

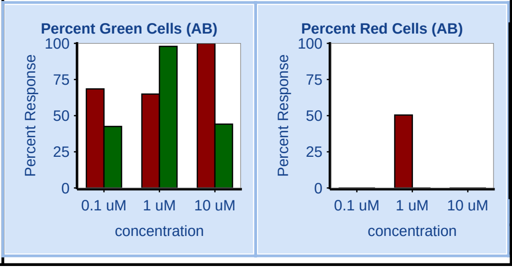

Average Response						
Dose	Green Signal	Actual Pct Green Cells	Normalized Pct Green Cells	Red Signal	Actual Pct Red Cells	Normalized Pct Red Cells
0.1 uM	96.85	0.85	55.53	87.09	0.07	-21.00
1 uM	100.87	0.85	81.43	108.52	0.15	9.25

Compound | Normalized Values (A and B Sets) | Responses

10 uM	104.80	0.93	435.83	100.45	0.09	-14.94

Compound | Normalized Values (A and B Sets) | Responses

RG2833 (RGFP109)

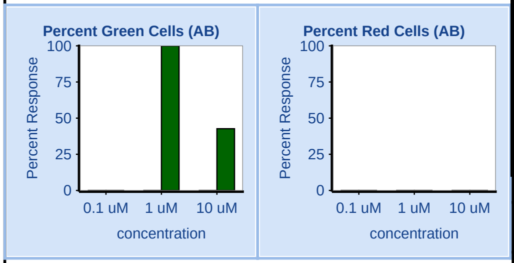

Average Response						
Dose	Green Signal	Actual Pct Green Cells	Normalized Pct Green Cells	Red Signal	Actual Pct Red Cells	Normalized Pct Red Cells
0.1 uM	84.80	0.77	-9.66	72.67	0.02	-63.84
1 uM	86.92	0.76	-931.03	60.64	0.01	-43.34
10 uM	92.43	0.83	17.89	92.31	0.10	-8.78

Compound | Normalized Values (A and B Sets) | Responses

RGD (Arg-Gly-Asp) Peptides

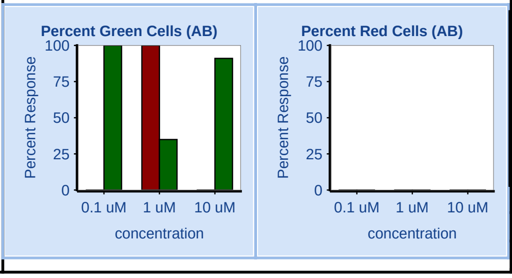

Average Response						
Dose	Green Signal	Actual Pct Green Cells	Normalized Pct Green Cells	Red Signal	Actual Pct Red Cells	Normalized Pct Red Cells
0.1 uM	96.81	0.86	71.22	66.69	0.01	-70.06
1 uM	102.53	0.87	80.58	68.62	0.01	-43.49
10 uM	88.51	0.84	18.62	66.50	0.03	-39.42

Compound | Normalized Values (A and B Sets) | Responses

Rheochrysidin

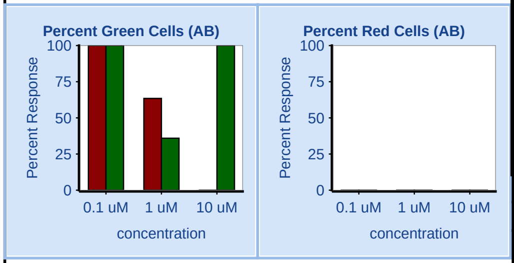

Average Response						
Dose	Green Signal	Actual Pct Green Cells	Normalized Pct Green Cells	Red Signal	Actual Pct Red Cells	Normalized Pct Red Cells
0.1 uM	100.78	0.93	138.22	90.57	0.05	-31.11
1 uM	92.42	0.80	49.70	76.45	0.03	-42.36
10 uM	95.16	0.87	-231.74	101.93	0.09	-15.22

Compound | Normalized Values (A and B Sets) | Responses

Ribitol

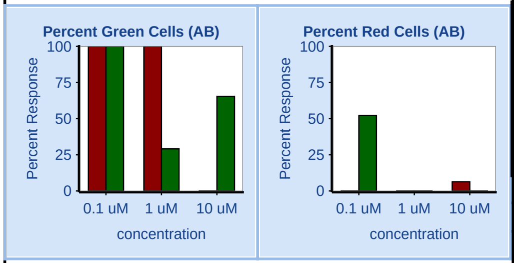

Average Response						
Dose	Green Signal	Actual Pct Green Cells	Normalized Pct Green Cells	Red Signal	Actual Pct Red Cells	Normalized Pct Red Cells
0.1 uM	104.38	0.94	144.25	104.10	0.14	16.17
1 uM	100.30	0.84	67.16	97.83	0.08	-21.51
10 uM	88.62	0.82	-458.34	105.57	0.10	-8.91

Compound | Normalized Values (A and B Sets) | Responses

Ribostamycin Sulfate

Average Response						
Dose	Green Signal			Red Signal	Actual Pct Red Cells	

Compound	Normalized Values (A and B Sets)	Responses

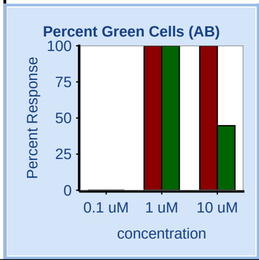

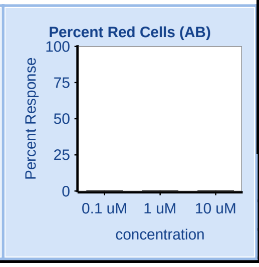

		Actual Pct Green Cells	Normalized Pct Green Cells			Normalized Pct Red Cells
0.1 uM	83.21	0.77	-146.36	90.55	0.08	-23.88
1 uM	107.22	0.90	132.53	75.23	0.03	-53.31
10 uM	98.37	0.83	91.14	93.33	0.09	-14.23

Compound	Normalized Values (A and B Sets)	Responses

Ridaforolimus (Deforolimus, MK-8669)

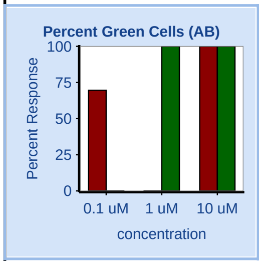

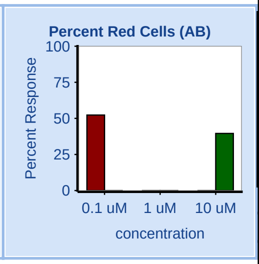

Average Response						
Dose	Green Signal	Actual Pct Green Cells	Normalized Pct Green Cells	Red Signal	Actual Pct Red Cells	Normalized Pct Red Cells
0.1 uM	86.89	0.79	16.78	102.56	0.11	-2.57
1 uM	97.06	0.92	-8.84	73.13	0.02	-38.94
10 uM	99.14	0.95	127.50	93.62	0.12	0.40

Compound	Normalized Values (A and B Sets)	Responses

Rifabutin

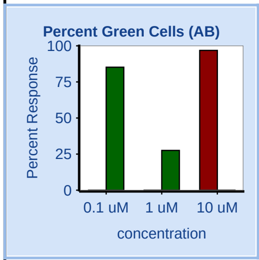

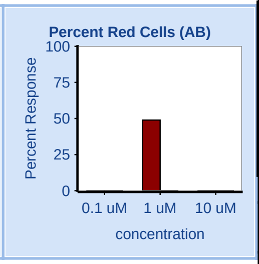

Average Response						
Dose	Green Signal	Actual Pct Green Cells	Normalized Pct Green Cells	Red Signal	Actual Pct Red Cells	Normalized Pct Red Cells
0.1 uM	93.29	0.81	-12.15	90.77	0.05	-42.86
1 uM	84.62	0.72	-130.96	111.50	0.14	6.54
10 uM	90.43	0.75	6.09	110.45	0.08	-17.71

Compound	Normalized Values (A and B Sets)	Responses

Rigosertib (ON-01910)

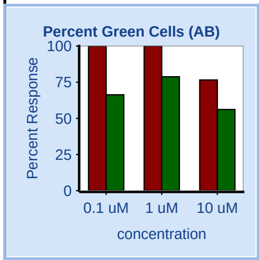

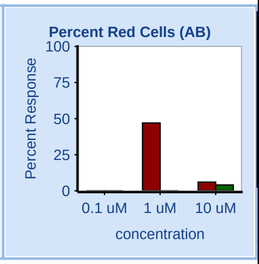

Average Response						
Dose	Green Signal	Actual Pct Green Cells	Normalized Pct Green Cells	Red Signal	Actual Pct Red Cells	Normalized Pct Red Cells
0.1 uM	122.39	0.92	135.98	96.99	0.10	-16.96
1 uM	121.10	0.90	119.76	117.80	0.17	19.12
10 uM	108.96	0.83	66.33	113.79	0.14	5.06

Compound	Normalized Values (A and B Sets)	Responses

Rilpivirine

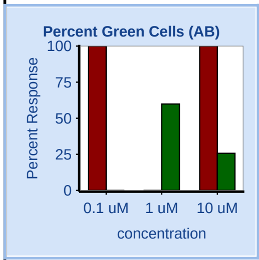

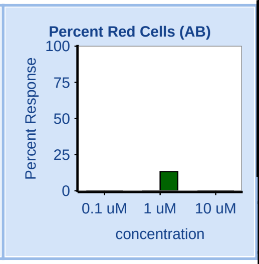

Average Response						
Dose	Green Signal	Actual Pct Green Cells	Normalized Pct Green Cells	Red Signal	Actual Pct Red Cells	Normalized Pct Red Cells
0.1 uM	88.05	0.73	-25.01	80.04	0.04	-49.86
1 uM	89.34	0.79	-526.77	93.74	0.09	-0.49
10 uM	102.04	0.90	90.21	77.24	0.04	-33.75

Compound	Normalized Values (A and B Sets)	Responses

Rimantadine

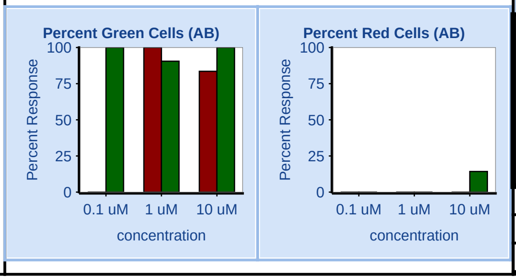

Average Response

Dose	Green Signal	Actual Pct Green Cells	Normalized Pct Green Cells	Red Signal	Actual Pct Red Cells	Normalized Pct Red Cells
0.1 uM	92.53	0.84	17.32	81.18	0.05	-47.32
1 uM	103.24	0.90	107.65	89.99	0.07	-26.07
10 uM	104.35	0.87	98.03	112.14	0.12	-0.62

Compound	Normalized Values (A and B Sets)	Responses

Rimonabant

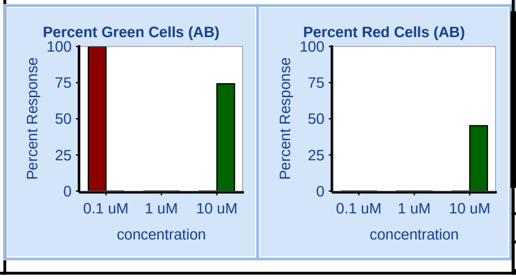

Average Response

Dose	Green Signal	Actual Pct Green Cells	Normalized Pct Green Cells	Red Signal	Actual Pct Red Cells	Normalized Pct Red Cells
0.1 uM	79.53	0.70	-21.39	79.43	0.05	-39.36
1 uM	83.79	0.71	-6.79	95.79	0.07	-31.61
10 uM	95.55	0.82	33.02	92.32	0.11	-9.29

Compound	Normalized Values (A and B Sets)	Responses

Ritodrine HCl

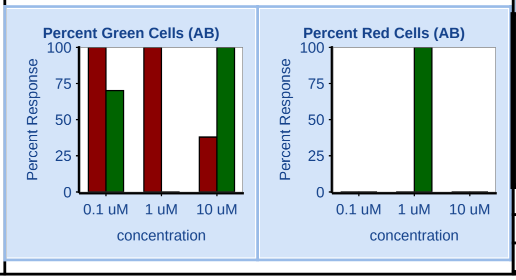

Average Response

Dose	Green Signal	Actual Pct Green Cells	Normalized Pct Green Cells	Red Signal	Actual Pct Red Cells	Normalized Pct Red Cells
0.1 uM	94.77	0.88	91.71	90.38	0.06	-36.46
1 uM	87.67	0.80	73.89	101.99	0.16	50.19
10 uM	103.39	0.91	85.87	79.17	0.05	-29.14

Compound	Normalized Values (A and B Sets)	Responses

Rivaroxaban

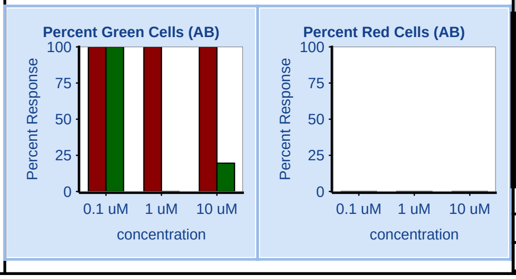

Average Response

Dose	Green Signal	Actual Pct Green Cells	Normalized Pct Green Cells	Red Signal	Actual Pct Red Cells	Normalized Pct Red Cells
0.1 uM	105.42	0.95	242.05	86.81	0.05	-38.77
1 uM	96.89	0.81	62.82	92.01	0.08	-24.05
10 uM	95.43	0.81	93.04	86.39	0.05	-35.78

Compound	Normalized Values (A and B Sets)	Responses

Rivastigmine Tartrate

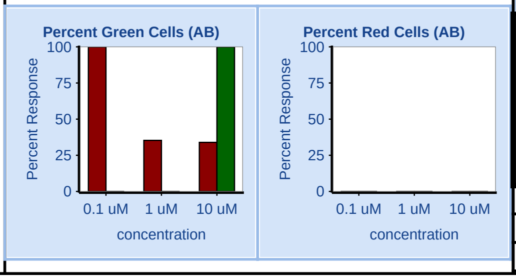

Average Response

Dose	Green Signal	Actual Pct Green Cells	Normalized Pct Green Cells	Red Signal	Actual Pct Red Cells	Normalized Pct Red Cells
0.1 uM	86.36	0.76	21.28	83.65	0.04	-51.80
1 uM	90.17	0.72	-29.02	88.09	0.05	-32.96
10 uM	103.95	0.85	85.20	102.81	0.06	-28.27

Compound	Normalized Values (A and B Sets)	Responses

RKI-1447

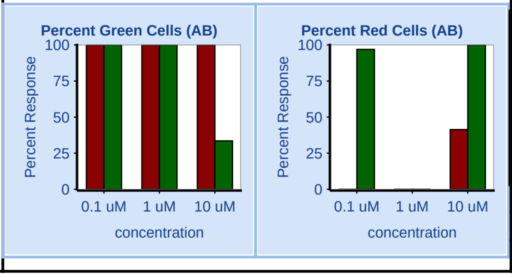

Average Response

Dose	Green Signal	Actual Pct Green Cells	Normalized Pct Green Cells	Red Signal	Actual Pct Red Cells	Normalized Pct Red Cells
0.1 uM	130.05	0.96	248.84	107.94	0.21	46.29
1 uM	117.50	0.88	113.07	93.45	0.09	-21.67
10 uM	112.64	0.85	141.30	136.87	0.28	83.65

Compound	Normalized Values (A and B Sets)	Responses

RO4929097

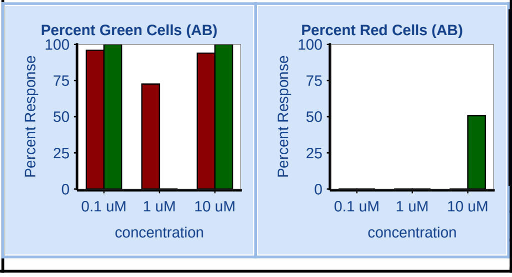

Average Response

Dose	Green Signal	Actual Pct Green Cells	Normalized Pct Green Cells	Red Signal	Actual Pct Red Cells	Normalized Pct Red Cells
0.1 uM	102.78	0.91	101.73	87.92	0.04	-51.24
1 uM	89.34	0.76	8.85	112.20	0.10	-9.23
10 uM	102.24	0.87	99.10	126.63	0.16	20.03

Compound	Normalized Values (A and B Sets)	Responses

Rocilinostat (ACY-1215)

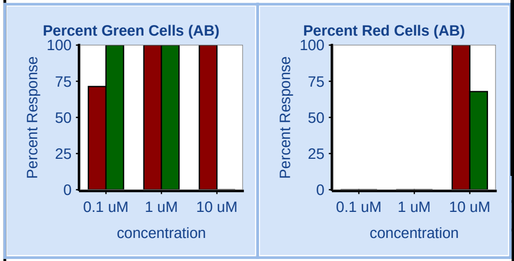

Average Response

Dose	Green Signal	Actual Pct Green Cells	Normalized Pct Green Cells	Red Signal	Actual Pct Red Cells	Normalized Pct Red Cells
0.1 uM	100.70	0.91	112.69	92.62	0.07	-33.11
1 uM	99.46	0.92	207.65	87.55	0.06	-15.44
10 uM	78.76	0.64	-144.64	151.65	0.33	96.30

Compound	Normalized Values (A and B Sets)	Responses

Rocuronium Bromide

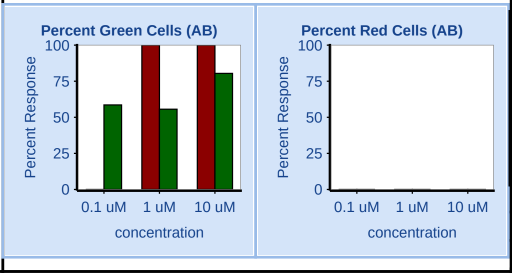

Average Response

Dose	Green Signal	Actual Pct Green Cells	Normalized Pct Green Cells	Red Signal	Actual Pct Red Cells	Normalized Pct Red Cells
0.1 uM	90.40	0.83	28.11	84.42	0.04	-52.89
1 uM	106.35	0.90	134.74	96.69	0.07	-26.48
10 uM	97.04	0.87	91.46	96.36	0.05	-31.93

Compound	Normalized Values (A and B Sets)	Responses

Roflumilast

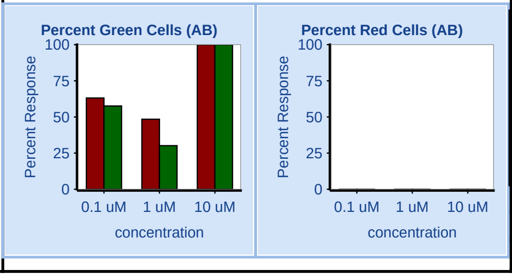

Average Response

Dose	Green Signal	Actual Pct Green Cells	Normalized Pct Green Cells	Red Signal	Actual Pct Red Cells	Normalized Pct Red Cells
0.1 uM	85.57	0.86	60.36	59.63	0.01	-50.44
1 uM	87.13	0.78	39.30	85.48	0.04	-39.59
10 uM	95.09	0.93	345.75	85.99	0.04	-38.50

Compound	Normalized Values (A and B Sets)	Responses
**Rosuvastatin Calcium**	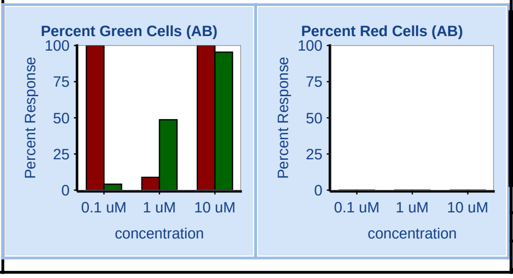	see table below

Average Response						
Dose	Green Signal	Actual Pct Green Cells	Normalized Pct Green Cells	Red Signal	Actual Pct Red Cells	Normalized Pct Red Cells
0.1 uM	93.35	0.87	66.88	94.29	0.07	-21.69
1 uM	86.14	0.75	28.76	86.35	0.04	-38.23
10 uM	99.19	0.93	337.18	80.28	0.03	-42.34

Compound	Normalized Values (A and B Sets)	Responses
**Rotundine**	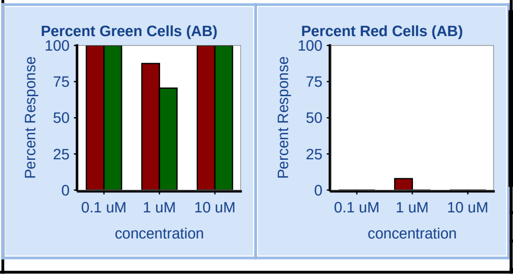	see table below

Average Response						
Dose	Green Signal	Actual Pct Green Cells	Normalized Pct Green Cells	Red Signal	Actual Pct Red Cells	Normalized Pct Red Cells
0.1 uM	96.42	0.91	118.57	94.72	0.08	-17.80
1 uM	98.57	0.86	79.02	96.90	0.09	-17.22
10 uM	117.05	0.97	529.72	84.15	0.04	-39.10

Compound	Normalized Values (A and B Sets)	Responses
**Roxithromycin**	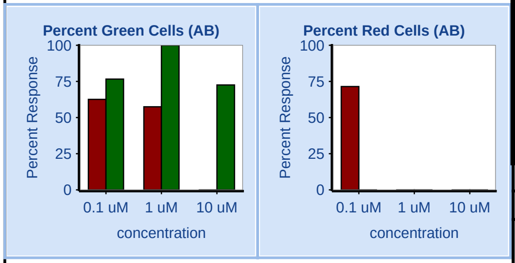	see table below

Average Response						
Dose	Green Signal	Actual Pct Green Cells	Normalized Pct Green Cells	Red Signal	Actual Pct Red Cells	Normalized Pct Red Cells
0.1 uM	95.94	0.86	69.61	104.69	0.16	14.49
1 uM	99.80	0.85	83.03	81.75	0.04	-38.85
10 uM	84.21	0.74	-1087.06	97.27	0.08	-19.40

Compound	Normalized Values (A and B Sets)	Responses
**RS-127445**	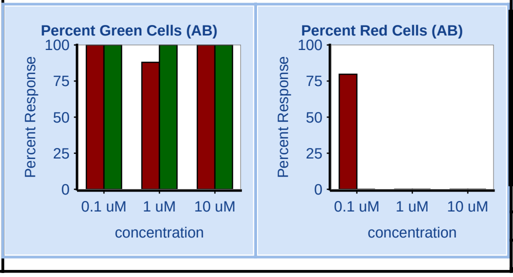	see table below

Average Response						
Dose	Green Signal	Actual Pct Green Cells	Normalized Pct Green Cells	Red Signal	Actual Pct Red Cells	Normalized Pct Red Cells
0.1 uM	104.53	0.93	131.39	106.39	0.16	12.78
1 uM	104.98	0.89	100.41	70.41	0.02	-48.96
10 uM	104.50	0.94	385.21	81.26	0.04	-40.03

Compound	Normalized Values (A and B Sets)	Responses
**Rufinamide**	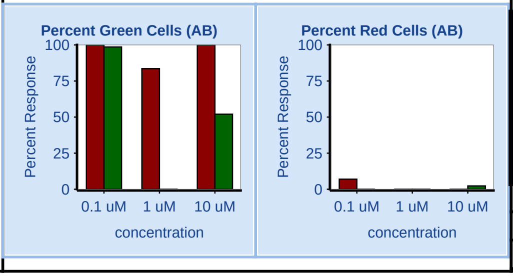	see table below

Average Response						
Dose	Green Signal	Actual Pct Green Cells	Normalized Pct Green Cells	Red Signal	Actual Pct Red Cells	Normalized Pct Red Cells
0.1 uM	103.98	0.91	112.55	105.72	0.11	-16.77
1 uM	88.52	0.70	-29.78	93.11	0.09	-18.43
10 uM	98.94	0.85	81.75	110.52	0.12	0.17

Compound	Normalized Values (A and B Sets)	Responses

Rupatadine Fumarate

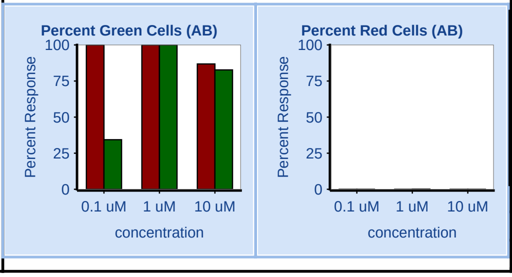

Average Response

Dose	Green Signal	Actual Pct Green Cells	Normalized Pct Green Cells	Red Signal	Actual Pct Red Cells	Normalized Pct Red Cells
0.1 uM	90.09	0.87	127.40	72.60	0.03	-50.60
1 uM	107.80	0.89	123.75	92.59	0.08	-27.38
10 uM	97.53	0.86	84.76	64.32	0.01	-58.47

Compound	Normalized Values (A and B Sets)	Responses

Rutaecarpine

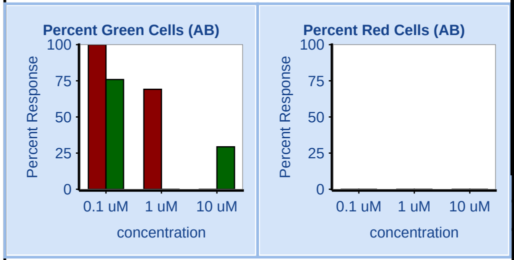

Average Response

Dose	Green Signal	Actual Pct Green Cells	Normalized Pct Green Cells	Red Signal	Actual Pct Red Cells	Normalized Pct Red Cells
0.1 uM	97.81	0.90	104.39	75.74	0.02	-45.54
1 uM	79.75	0.65	-45.50	71.69	0.02	-47.33
10 uM	88.81	0.85	-111.20	84.59	0.04	-41.65

Compound	Normalized Values (A and B Sets)	Responses

Ruxolitinib (INCB018424)

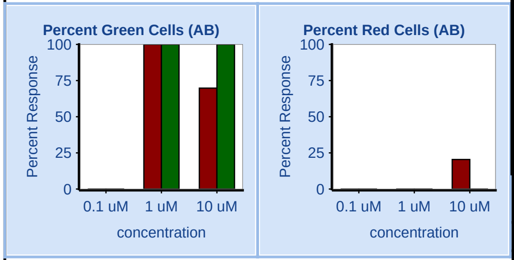

Average Response

Dose	Green Signal	Actual Pct Green Cells	Normalized Pct Green Cells	Red Signal	Actual Pct Red Cells	Normalized Pct Red Cells
0.1 uM	68.57	0.53	-162.74	76.44	0.02	-62.56
1 uM	107.61	0.91	107.04	82.42	0.04	-41.03
10 uM	109.56	0.89	110.25	113.67	0.11	-11.87

Compound	Normalized Values (A and B Sets)	Responses

RVX-208

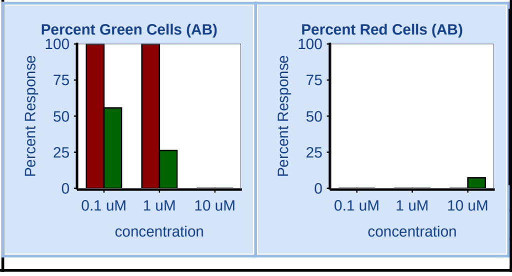

Average Response

Dose	Green Signal	Actual Pct Green Cells	Normalized Pct Green Cells	Red Signal	Actual Pct Red Cells	Normalized Pct Red Cells
0.1 uM	101.48	0.88	95.37	81.18	0.06	-39.75
1 uM	96.51	0.88	190.72	81.80	0.05	-25.95
10 uM	80.98	0.79	-19.58	88.34	0.08	-16.30

Compound	Normalized Values (A and B Sets)	Responses

S- (+)-Rolipram

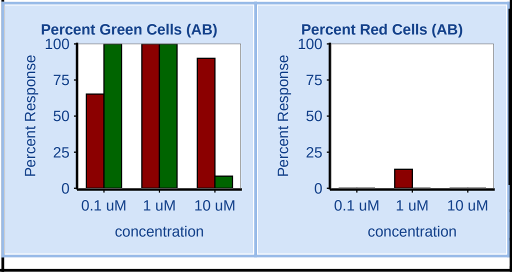

Average Response

Dose	Green Signal	Actual Pct Green Cells	Normalized Pct Green Cells	Red Signal	Actual Pct Red Cells	Normalized Pct Red Cells
0.1 uM	97.69	0.89	99.99	80.63	0.03	-57.36
1 uM	98.00	0.91	110.01	97.38	0.10	-3.71
10 uM	87.61	0.86	49.20	76.35	0.04	-34.56

Compound	Normalized Values (A and B Sets)	Responses

S-Ruxolitinib (INCB018424)

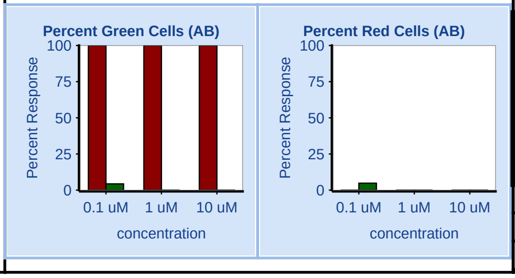

Average Response

Dose	Green Signal	Actual Pct Green Cells	Normalized Pct Green Cells	Red Signal	Actual Pct Red Cells	Normalized Pct Red Cells
0.1 uM	85.26	0.83	52.29	82.21	0.07	-26.51
1 uM	89.08	0.77	27.76	60.67	0.01	-63.96
10 uM	95.67	0.79	94.95	87.09	0.05	-36.40

Compound	Normalized Values (A and B Sets)	Responses

S3I-201

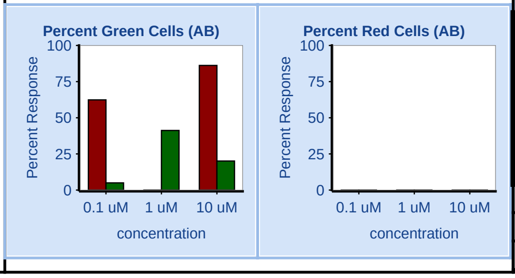

Average Response

Dose	Green Signal	Actual Pct Green Cells	Normalized Pct Green Cells	Red Signal	Actual Pct Red Cells	Normalized Pct Red Cells
0.1 uM	87.24	0.81	33.67	99.97	0.07	-41.80
1 uM	92.85	0.81	8.35	69.12	0.01	-54.00
10 uM	95.96	0.81	53.14	98.21	0.05	-34.09

Compound	Normalized Values (A and B Sets)	Responses

SA4503

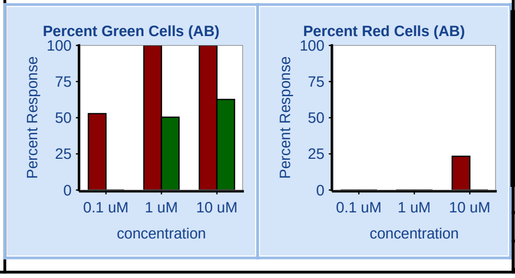

Average Response

Dose	Green Signal	Actual Pct Green Cells	Normalized Pct Green Cells	Red Signal	Actual Pct Red Cells	Normalized Pct Red Cells
0.1 uM	76.24	0.67	-87.16	72.61	0.02	-64.23
1 uM	97.50	0.88	126.94	63.80	0.01	-44.57
10 uM	105.02	0.93	112.75	93.45	0.09	-9.43

Compound	Normalized Values (A and B Sets)	Responses

Safinamide Mesylate

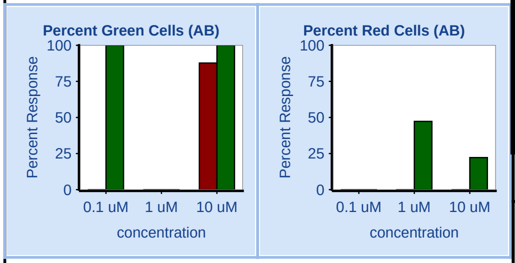

Average Response

Dose	Green Signal	Actual Pct Green Cells	Normalized Pct Green Cells	Red Signal	Actual Pct Red Cells	Normalized Pct Red Cells
0.1 uM	80.35	0.54	-235.70	85.93	0.06	-39.42
1 uM	82.64	0.68	-1004.47	96.33	0.14	23.26
10 uM	103.47	0.94	117.96	91.83	0.12	3.14

Compound	Normalized Values (A and B Sets)	Responses

Salidroside

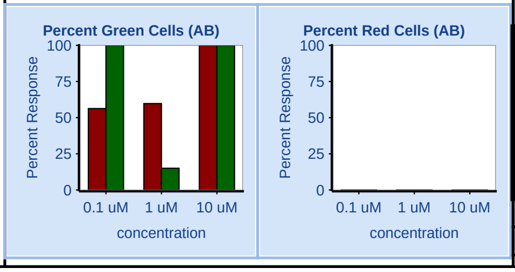

Average Response

Dose	Green Signal	Actual Pct Green Cells	Normalized Pct Green Cells	Red Signal	Actual Pct Red Cells	Normalized Pct Red Cells
0.1 uM	93.85	0.88	84.38	67.23	0.03	-40.06
1 uM	89.97	0.78	37.38	71.69	0.01	-49.48

Compound	Normalized Values (A and B Sets)	Responses

10 uM	98.44	0.93	279.79	82.25	0.04	-40.57

Compound	Normalized Values (A and B Sets)	Responses

Salinomycin

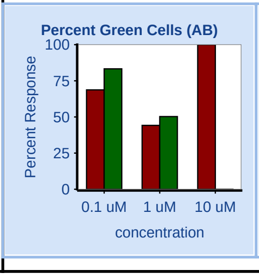

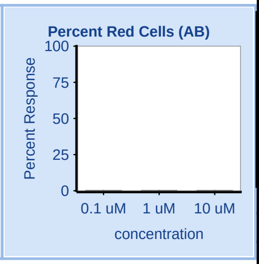

Average Response

Dose	Green Signal	Actual Pct Green Cells	Normalized Pct Green Cells	Red Signal	Actual Pct Red Cells	Normalized Pct Red Cells
0.1 uM	91.35	0.87	76.01	76.98	0.03	-41.74
1 uM	93.45	0.79	47.23	69.51	0.01	-49.13
10 uM	103.87	0.88	221.08	78.49	0.05	-34.19

Compound	Normalized Values (A and B Sets)	Responses

Salubrinal

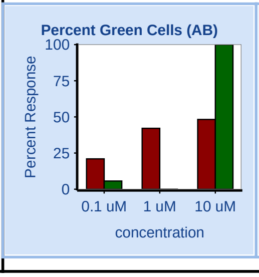

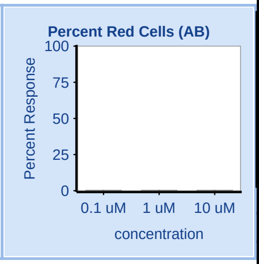

Average Response

Dose	Green Signal	Actual Pct Green Cells	Normalized Pct Green Cells	Red Signal	Actual Pct Red Cells	Normalized Pct Red Cells
0.1 uM	87.29	0.82	13.33	91.08	0.07	-27.36
1 uM	78.78	0.63	-69.76	79.26	0.05	-43.56
10 uM	101.29	0.88	81.03	100.23	0.10	-9.36

Compound	Normalized Values (A and B Sets)	Responses

SANT-1

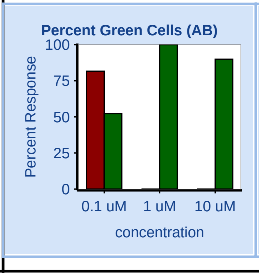

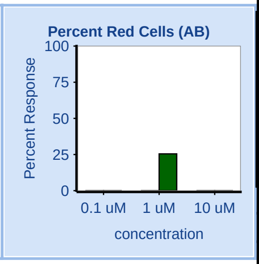

Average Response

Dose	Green Signal	Actual Pct Green Cells	Normalized Pct Green Cells	Red Signal	Actual Pct Red Cells	Normalized Pct Red Cells
0.1 uM	95.83	0.86	66.95	82.08	0.05	-39.64
1 uM	93.97	0.78	48.22	108.03	0.13	0.23
10 uM	94.67	0.81	12.81	88.13	0.06	-31.64

Compound	Normalized Values (A and B Sets)	Responses

SANT-2

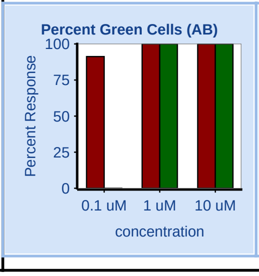

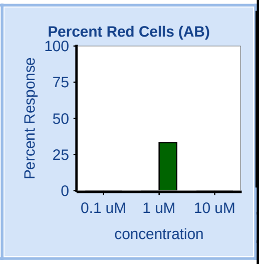

Average Response

Dose	Green Signal	Actual Pct Green Cells	Normalized Pct Green Cells	Red Signal	Actual Pct Red Cells	Normalized Pct Red Cells
0.1 uM	90.40	0.82	41.80	79.26	0.03	-54.31
1 uM	103.08	0.92	149.65	86.43	0.08	2.04
10 uM	100.29	0.95	132.25	89.25	0.08	-14.28

Compound	Normalized Values (A and B Sets)	Responses

SAR131675

Average Response

Dose	Green Signal	Actual Pct Green Cells	Normalized Pct Green Cells	Red Signal	Actual Pct Red Cells	Normalized Pct Red Cells

Compound	Normalized Values (A and B Sets)	Responses

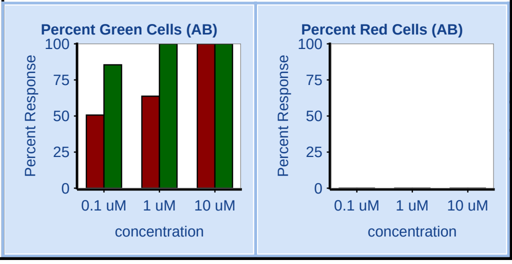

0.1 uM	93.75	0.86	68.07	96.77	0.09	-14.20
1 uM	108.30	0.87	92.98	88.32	0.06	-30.50
10 uM	105.95	0.94	342.52	94.70	0.07	-25.32

Compound	Normalized Values (A and B Sets)	Responses

SAR245409 (XL765)

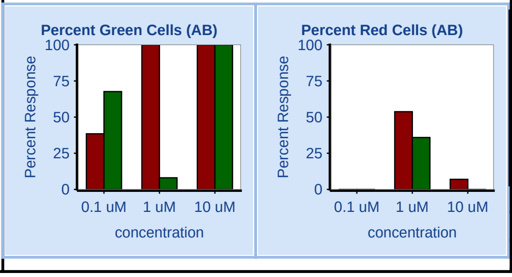

Average Response

Dose	Green Signal	Actual Pct Green Cells	Normalized Pct Green Cells	Red Signal	Actual Pct Red Cells	Normalized Pct Red Cells
0.1 uM	99.60	0.85	53.05	93.70	0.09	-24.52
1 uM	107.33	0.85	95.92	130.76	0.22	44.82
10 uM	113.66	0.94	141.21	110.22	0.10	-13.64

Compound	Normalized Values (A and B Sets)	Responses

Saxagliptin

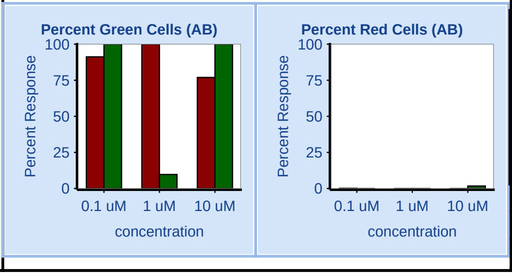

Average Response

Dose	Green Signal	Actual Pct Green Cells	Normalized Pct Green Cells	Red Signal	Actual Pct Red Cells	Normalized Pct Red Cells
0.1 uM	102.71	0.90	97.25	105.71	0.12	-12.64
1 uM	99.10	0.83	70.81	84.08	0.05	-36.87
10 uM	100.28	0.87	95.01	113.55	0.11	-5.66

Compound	Normalized Values (A and B Sets)	Responses

SB 265610

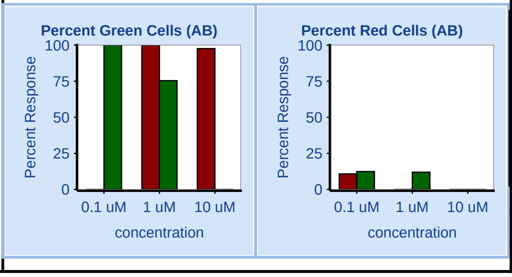

Average Response

Dose	Green Signal	Actual Pct Green Cells	Normalized Pct Green Cells	Red Signal	Actual Pct Red Cells	Normalized Pct Red Cells
0.1 uM	88.16	0.83	39.79	108.93	0.14	11.60
1 uM	95.35	0.89	124.93	93.59	0.09	-0.97
10 uM	89.46	0.85	35.57	79.61	0.04	-32.76

Compound	Normalized Values (A and B Sets)	Responses

SB269970 HCl

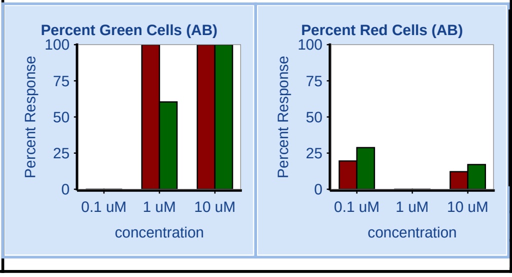

Average Response

Dose	Green Signal	Actual Pct Green Cells	Normalized Pct Green Cells	Red Signal	Actual Pct Red Cells	Normalized Pct Red Cells
0.1 uM	82.05	0.72	-74.09	111.61	0.17	24.17
1 uM	104.05	0.87	83.57	73.42	0.06	-27.55
10 uM	114.37	0.97	411.51	113.55	0.15	14.61

Compound | Normalized Values (A and B Sets) | Responses

SB271046

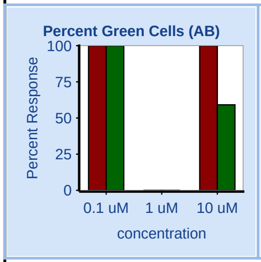

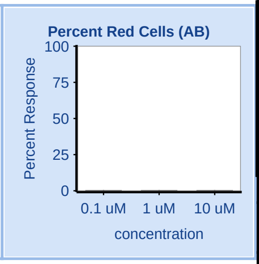

Average Response

Dose	Green Signal	Actual Pct Green Cells	Normalized Pct Green Cells	Red Signal	Actual Pct Red Cells	Normalized Pct Red Cells
0.1 uM	103.31	0.92	126.96	78.15	0.02	-62.78
1 uM	81.17	0.71	-664.64	75.98	0.03	-33.95
10 uM	101.37	0.91	97.66	68.93	0.02	-40.91

Compound | Normalized Values (A and B Sets) | Responses

SB408124

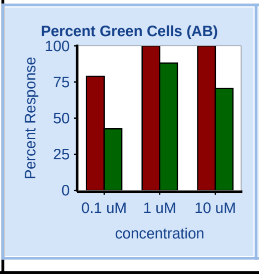

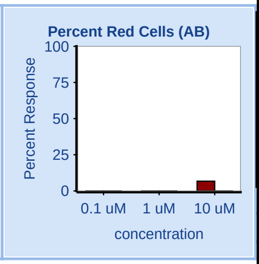

Average Response

Dose	Green Signal	Actual Pct Green Cells	Normalized Pct Green Cells	Red Signal	Actual Pct Red Cells	Normalized Pct Red Cells
0.1 uM	96.26	0.85	60.75	80.90	0.03	-57.72
1 uM	101.98	0.89	101.20	84.79	0.04	-41.43
10 uM	106.13	0.87	93.22	112.15	0.10	-14.91

Compound | Normalized Values (A and B Sets) | Responses

SB415286

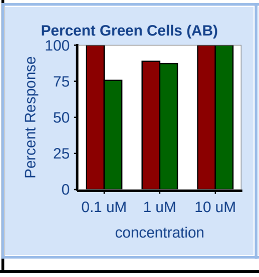

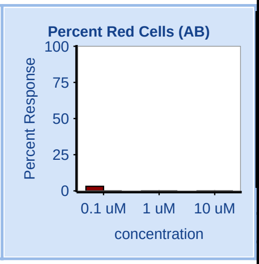

Average Response

Dose	Green Signal	Actual Pct Green Cells	Normalized Pct Green Cells	Red Signal	Actual Pct Red Cells	Normalized Pct Red Cells
0.1 uM	104.37	0.92	118.29	92.11	0.08	-17.97
1 uM	106.39	0.87	88.05	84.11	0.04	-37.58
10 uM	107.23	0.93	186.76	86.53	0.05	-35.13

Compound | Normalized Values (A and B Sets) | Responses

SB505124

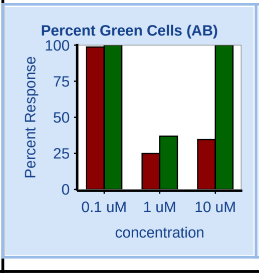

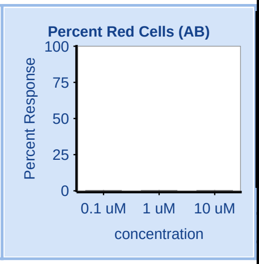

Average Response

Dose	Green Signal	Actual Pct Green Cells	Normalized Pct Green Cells	Red Signal	Actual Pct Red Cells	Normalized Pct Red Cells
0.1 uM	99.10	0.90	110.76	66.22	0.01	-47.82
1 uM	90.19	0.76	30.91	84.04	0.06	-29.56
10 uM	96.70	0.92	111.27	86.76	0.05	-36.75

Compound | Normalized Values (A and B Sets) | Responses

SB525334

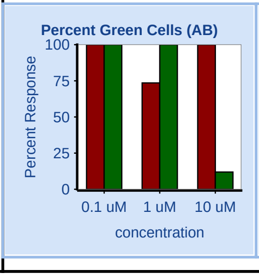

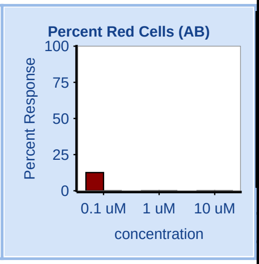

Average Response

Dose	Green Signal	Actual Pct Green Cells	Normalized Pct Green Cells	Red Signal	Actual Pct Red Cells	Normalized Pct Red Cells
0.1 uM	105.53	0.92	115.56	104.59	0.11	-19.57
1 uM	115.53	0.91	101.36	93.09	0.07	-26.62
10 uM	97.01	0.83	64.42	103.16	0.07	-23.82

Compound	Normalized Values (A and B Sets)	Responses
**SB590885**	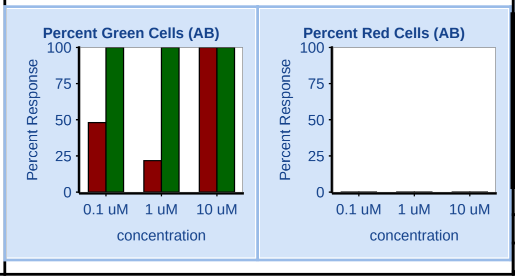	see table below

Average Response						
Dose	Green Signal	Actual Pct Green Cells	Normalized Pct Green Cells	Red Signal	Actual Pct Red Cells	Normalized Pct Red Cells
0.1 uM	96.41	0.89	95.50	89.68	0.07	-24.70
1 uM	99.19	0.83	74.92	89.31	0.05	-32.52
10 uM	105.34	0.97	554.19	89.81	0.04	-38.90

Compound	Normalized Values (A and B Sets)	Responses
**SB705498**	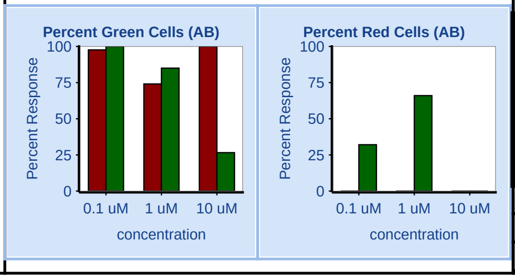	see table below

Average Response						
Dose	Green Signal	Actual Pct Green Cells	Normalized Pct Green Cells	Red Signal	Actual Pct Red Cells	Normalized Pct Red Cells
0.1 uM	109.08	0.93	134.86	95.06	0.13	7.85
1 uM	102.85	0.85	79.56	117.80	0.20	32.53
10 uM	96.19	0.91	297.99	91.23	0.08	-21.84

Compound	Normalized Values (A and B Sets)	Responses
**SB742457**	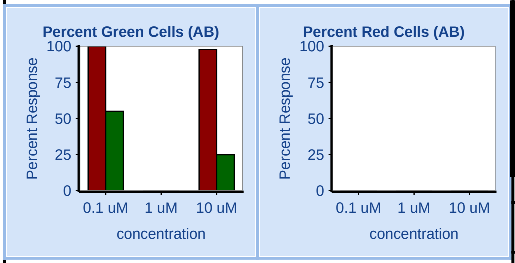	see table below

Average Response						
Dose	Green Signal	Actual Pct Green Cells	Normalized Pct Green Cells	Red Signal	Actual Pct Red Cells	Normalized Pct Red Cells
0.1 uM	91.72	0.88	142.10	73.39	0.01	-57.35
1 uM	71.38	0.53	-132.30	77.27	0.04	-46.45
10 uM	92.04	0.80	61.29	90.66	0.05	-38.85

Compound	Normalized Values (A and B Sets)	Responses
**SB743921**	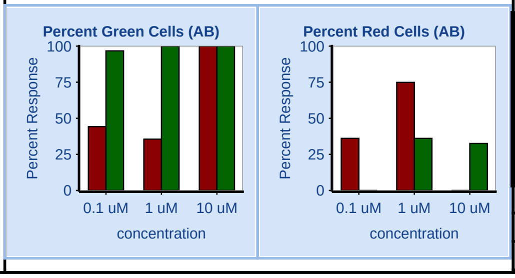	see table below

Average Response						
Dose	Green Signal	Actual Pct Green Cells	Normalized Pct Green Cells	Red Signal	Actual Pct Red Cells	Normalized Pct Red Cells
0.1 uM	103.59	0.86	70.48	101.19	0.14	7.40
1 uM	111.56	0.82	68.87	131.97	0.25	55.48
10 uM	97.47	0.92	151.41	113.23	0.11	-6.53

Compound	Normalized Values (A and B Sets)	Responses
**SC-514**	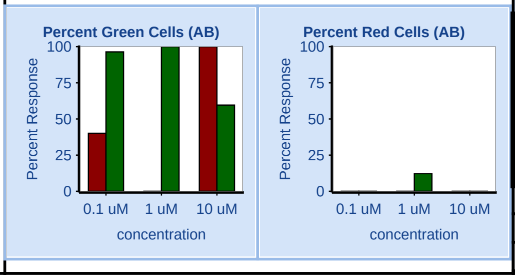	see table below

Average Response						
Dose	Green Signal	Actual Pct Green Cells	Normalized Pct Green Cells	Red Signal	Actual Pct Red Cells	Normalized Pct Red Cells
0.1 uM	94.89	0.89	68.26	90.67	0.09	-17.64
1 uM	94.31	0.74	19.73	98.15	0.12	-7.84
10 uM	108.09	0.87	132.12	86.32	0.07	-28.21

Compound	Normalized Values (A and B Sets)	Responses

SC144

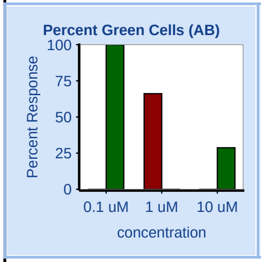

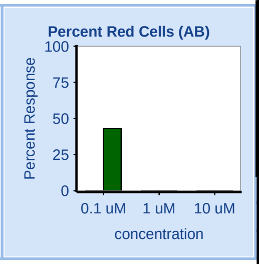

Average Response						
Dose	Green Signal	Actual Pct Green Cells	Normalized Pct Green Cells	Red Signal	Actual Pct Red Cells	Normalized Pct Red Cells
0.1 uM	102.93	0.87	-64.35	99.32	0.14	7.62
1 uM	93.46	0.73	4.65	83.96	0.06	-36.46
10 uM	86.64	0.68	-126.76	87.81	0.05	-36.31

Compound	Normalized Values (A and B Sets)	Responses

SCH 79797

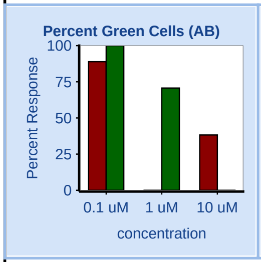

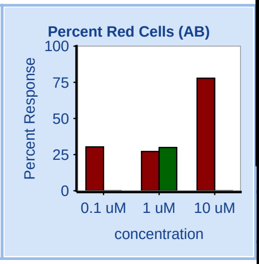

Average Response						
Dose	Green Signal	Actual Pct Green Cells	Normalized Pct Green Cells	Red Signal	Actual Pct Red Cells	Normalized Pct Red Cells
0.1 uM	106.76	0.91	118.78	103.42	0.12	0.06
1 uM	104.31	0.85	-133.75	110.16	0.17	28.54
10 uM	68.23	0.55	-232.03	104.10	0.17	27.81

Compound	Normalized Values (A and B Sets)	Responses

SCH527123

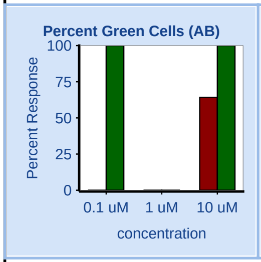

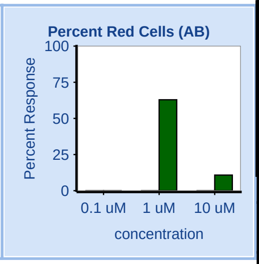

Average Response						
Dose	Green Signal	Actual Pct Green Cells	Normalized Pct Green Cells	Red Signal	Actual Pct Red Cells	Normalized Pct Red Cells
0.1 uM	93.95	0.85	58.37	96.21	0.09	-22.05
1 uM	89.46	0.80	-165.64	94.48	0.13	25.02
10 uM	96.67	0.91	86.98	86.40	0.09	-12.01

Compound	Normalized Values (A and B Sets)	Responses

SCH772984

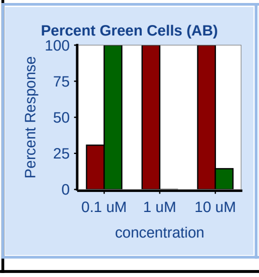

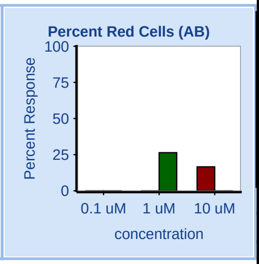

Average Response						
Dose	Green Signal	Actual Pct Green Cells	Normalized Pct Green Cells	Red Signal	Actual Pct Red Cells	Normalized Pct Red Cells
0.1 uM	98.89	0.90	70.26	90.53	0.08	-21.95
1 uM	88.72	0.78	40.21	105.15	0.14	4.31
10 uM	108.38	0.84	139.33	101.97	0.12	2.28

Compound	Normalized Values (A and B Sets)	Responses

Schisandrin B (Sch B)

Average Response						
Dose	Green Signal	Actual Pct Green Cells	Normalized Pct Green Cells	Red Signal	Actual Pct Red Cells	Normalized Pct Red Cells
0.1 uM	84.43	0.78	-130.24	60.54	0.01	-59.59

Compound	Normalized Values (A and B Sets)	Responses

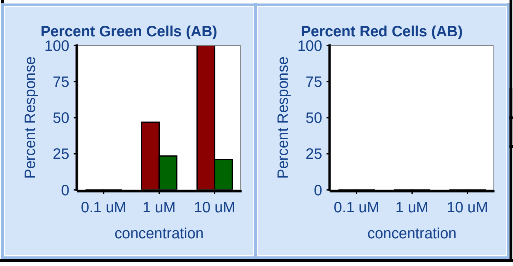

1 uM	89.30	0.77	35.22	64.18	0.01	-64.28
10 uM	99.87	0.84	128.53	76.48	0.03	-49.48

Compound	Normalized Values (A and B Sets)	Responses

Sclareol

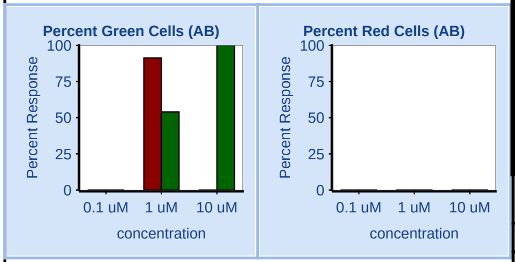

Average Response						
Dose	Green Signal	Actual Pct Green Cells	Normalized Pct Green Cells	Red Signal	Actual Pct Red Cells	Normalized Pct Red Cells
0.1 uM	74.14	0.64	-147.98	72.91	0.03	-43.56
1 uM	93.27	0.85	72.70	78.62	0.02	-44.69
10 uM	94.54	0.89	6.84	92.07	0.05	-35.54

Compound	Normalized Values (A and B Sets)	Responses

Sclareolide

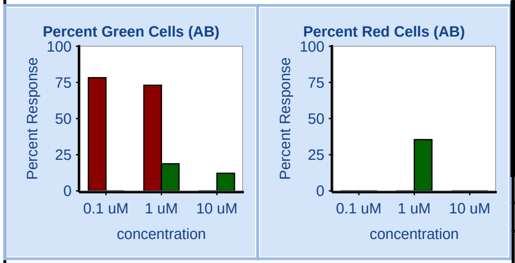

Average Response						
Dose	Green Signal	Actual Pct Green Cells	Normalized Pct Green Cells	Red Signal	Actual Pct Red Cells	Normalized Pct Red Cells
0.1 uM	85.12	0.80	-1.29	88.83	0.06	-29.53
1 uM	91.12	0.80	45.90	95.42	0.11	-7.83
10 uM	92.75	0.84	-223.73	72.80	0.02	-50.68

Compound	Normalized Values (A and B Sets)	Responses

Scopine

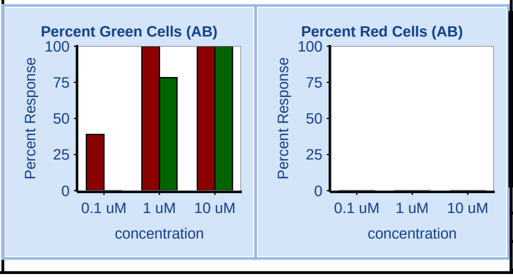

Average Response						
Dose	Green Signal	Actual Pct Green Cells	Normalized Pct Green Cells	Red Signal	Actual Pct Red Cells	Normalized Pct Red Cells
0.1 uM	84.66	0.80	-3.72	89.45	0.07	-19.26
1 uM	103.76	0.89	93.27	88.27	0.06	-30.27
10 uM	91.84	0.90	116.48	76.09	0.03	-42.63

Compound	Normalized Values (A and B Sets)	Responses

Scopine HCl

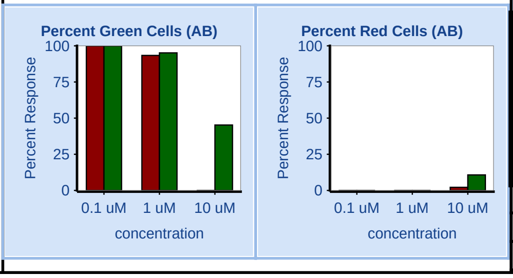

Average Response						
Dose	Green Signal	Actual Pct Green Cells	Normalized Pct Green Cells	Red Signal	Actual Pct Red Cells	Normalized Pct Red Cells
0.1 uM	104.69	0.94	147.38	80.75	0.05	-34.84
1 uM	104.32	0.88	94.25	78.42	0.04	-38.27
10 uM	91.92	0.87	-40.31	112.45	0.14	6.43

Compound	Normalized Values (A and B Sets)	Responses

Scopolamine HBr

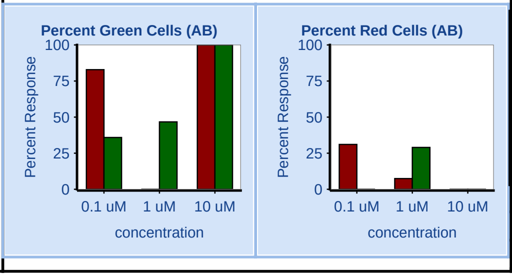

Average Response

Dose	Green Signal	Actual Pct Green Cells	Normalized Pct Green Cells	Red Signal	Actual Pct Red Cells	Normalized Pct Red Cells
0.1 uM	92.25	0.86	59.38	95.59	0.11	-8.37
1 uM	86.68	0.72	17.25	114.84	0.17	18.21
10 uM	111.79	0.93	277.73	87.99	0.05	-32.09

Compound	Normalized Values (A and B Sets)	Responses

Scriptaid

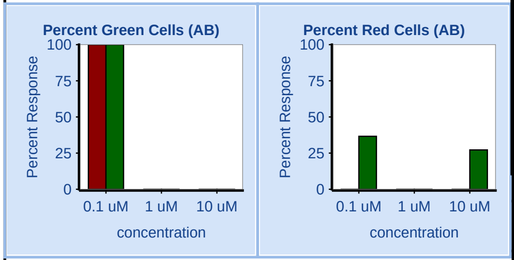

Average Response

Dose	Green Signal	Actual Pct Green Cells	Normalized Pct Green Cells	Red Signal	Actual Pct Red Cells	Normalized Pct Red Cells
0.1 uM	112.98	0.94	147.49	91.50	0.11	-9.03
1 uM	75.73	0.68	-769.91	63.28	0.02	-35.95
10 uM	95.20	0.78	-31.70	92.04	0.12	2.96

Compound	Normalized Values (A and B Sets)	Responses

SecinH3

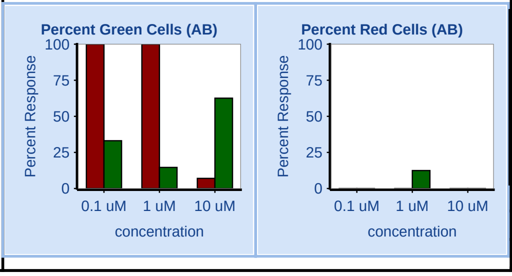

Average Response

Dose	Green Signal	Actual Pct Green Cells	Normalized Pct Green Cells	Red Signal	Actual Pct Red Cells	Normalized Pct Red Cells
0.1 uM	95.95	0.88	99.84	68.09	0.01	-70.23
1 uM	91.78	0.86	102.19	94.32	0.08	-3.98
10 uM	90.25	0.85	34.81	76.98	0.04	-32.99

Compound	Normalized Values (A and B Sets)	Responses

Semagacestat (LY450139)

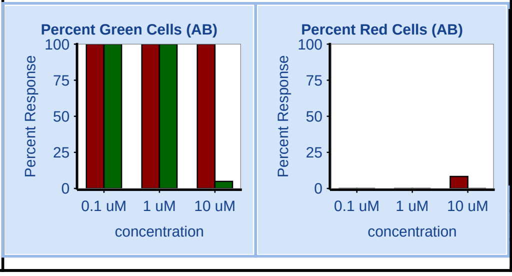

Average Response

Dose	Green Signal	Actual Pct Green Cells	Normalized Pct Green Cells	Red Signal	Actual Pct Red Cells	Normalized Pct Red Cells
0.1 uM	117.01	0.94	137.32	93.23	0.07	-37.33
1 uM	111.39	0.93	129.42	91.06	0.07	-25.71
10 uM	99.18	0.83	64.98	114.41	0.10	-11.94

Compound	Normalized Values (A and B Sets)	Responses

Sertaconazole nitrate

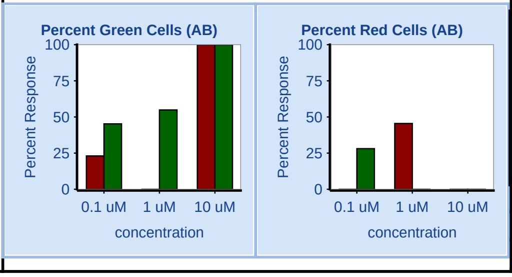

Average Response

Dose	Green Signal	Actual Pct Green Cells	Normalized Pct Green Cells	Red Signal	Actual Pct Red Cells	Normalized Pct Red Cells
0.1 uM	92.16	0.82	34.16	91.43	0.09	-17.47
1 uM	96.48	0.87	24.46	103.71	0.16	14.27
10 uM	103.65	0.95	134.81	84.11	0.08	-17.19

Compound	Normalized Values (A and B Sets)	Responses
**Sesamin**	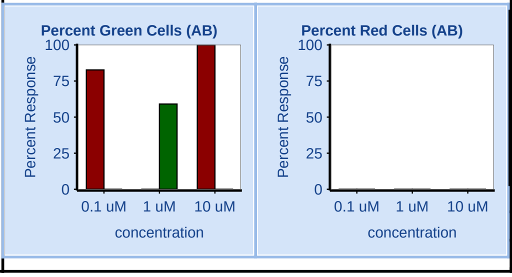	see table below

Average Response

Dose	Green Signal	Actual Pct Green Cells	Normalized Pct Green Cells	Red Signal	Actual Pct Red Cells	Normalized Pct Red Cells
0.1 uM	84.80	0.72	-90.71	84.86	0.05	-31.91
1 uM	86.86	0.74	26.72	77.86	0.03	-44.27
10 uM	94.39	0.86	49.82	92.49	0.06	-30.04

Compound	Normalized Values (A and B Sets)	Responses
**SGC 0946**	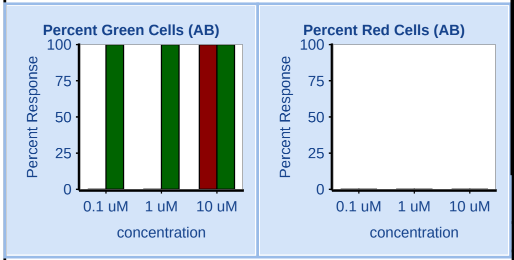	see table below

Average Response

Dose	Green Signal	Actual Pct Green Cells	Normalized Pct Green Cells	Red Signal	Actual Pct Red Cells	Normalized Pct Red Cells
0.1 uM	85.88	0.73	-752.12	79.30	0.05	-40.31
1 uM	91.56	0.75	26.72	97.75	0.09	-22.00
10 uM	102.06	0.89	124.75	77.46	0.01	-57.09

Compound	Normalized Values (A and B Sets)	Responses
**SGC-CBP30**	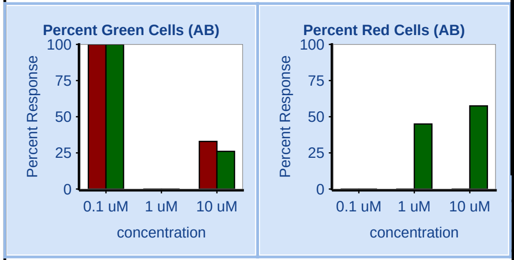	see table below

Average Response

Dose	Green Signal	Actual Pct Green Cells	Normalized Pct Green Cells	Red Signal	Actual Pct Red Cells	Normalized Pct Red Cells
0.1 uM	105.07	0.92	129.18	84.05	0.03	-53.31
1 uM	84.05	0.77	-272.25	93.40	0.11	12.71
10 uM	92.76	0.84	29.51	99.75	0.16	17.40

Compound	Normalized Values (A and B Sets)	Responses
**SGI-1027**	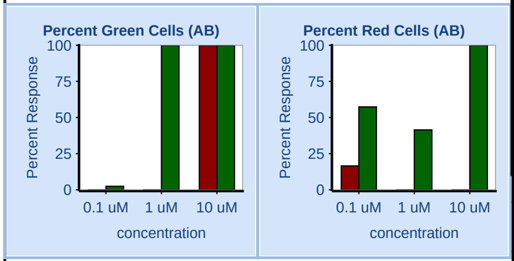	see table below

Average Response

Dose	Green Signal	Actual Pct Green Cells	Normalized Pct Green Cells	Red Signal	Actual Pct Red Cells	Normalized Pct Red Cells
0.1 uM	79.06	0.73	-42.90	116.83	0.18	36.94
1 uM	89.23	0.81	-654.72	100.07	0.11	12.20
10 uM	94.85	0.97	151.04	124.48	0.30	78.06

Compound	Normalized Values (A and B Sets)	Responses
**SGI-1776 free base**	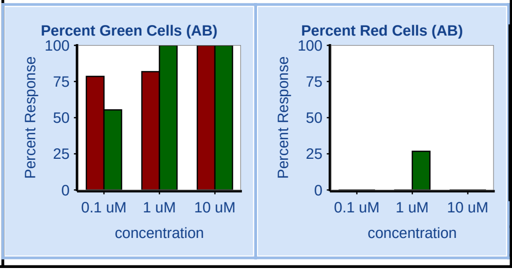	see table below

Average Response

Dose	Green Signal	Actual Pct Green Cells	Normalized Pct Green Cells	Red Signal	Actual Pct Red Cells	Normalized Pct Red Cells
0.1 uM	91.99	0.86	66.97	83.24	0.05	-32.76
1 uM	96.75	0.88	95.46	103.72	0.10	-8.82

Compound	Normalized Values (A and B Sets)	Responses

10 uM	97.83	0.95	526.47	77.14	0.06	-31.80

Compound | Normalized Values (A and B Sets) | Responses

SGX-523

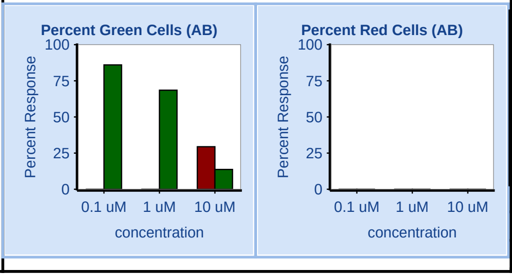

Average Response

Dose	Green Signal	Actual Pct Green Cells	Normalized Pct Green Cells	Red Signal	Actual Pct Red Cells	Normalized Pct Red Cells
0.1 uM	91.87	0.85	38.22	89.59	0.05	-42.56
1 uM	92.45	0.82	-0.74	71.34	0.01	-51.76
10 uM	86.16	0.76	21.55	96.49	0.04	-36.23

Compound | Normalized Values (A and B Sets) | Responses

Shikimic Acid

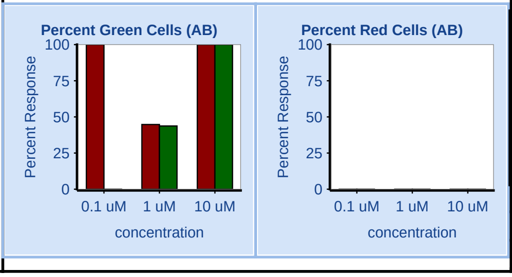

Average Response

Dose	Green Signal	Actual Pct Green Cells	Normalized Pct Green Cells	Red Signal	Actual Pct Red Cells	Normalized Pct Red Cells
0.1 uM	89.71	0.85	43.60	70.14	0.02	-45.95
1 uM	89.37	0.79	44.23	67.14	0.01	-51.62
10 uM	97.49	0.94	363.39	94.16	0.06	-31.73

Compound | Normalized Values (A and B Sets) | Responses

SID 7969543

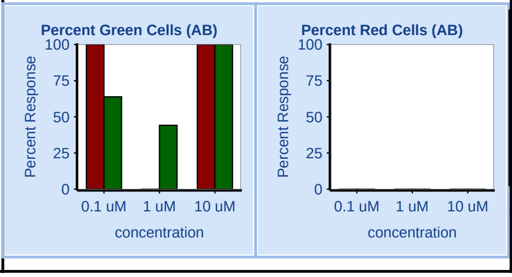

Average Response

Dose	Green Signal	Actual Pct Green Cells	Normalized Pct Green Cells	Red Signal	Actual Pct Red Cells	Normalized Pct Red Cells
0.1 uM	97.00	0.87	86.64	75.98	0.02	-64.29
1 uM	91.18	0.84	-96.92	67.34	0.02	-41.50
10 uM	100.80	0.94	122.47	88.21	0.06	-22.00

Compound | Normalized Values (A and B Sets) | Responses

Sildenafil Citrate

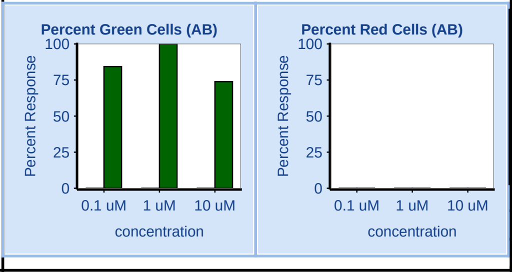

Average Response

Dose	Green Signal	Actual Pct Green Cells	Normalized Pct Green Cells	Red Signal	Actual Pct Red Cells	Normalized Pct Red Cells
0.1 uM	89.66	0.82	-1.52	84.02	0.03	-55.15
1 uM	95.86	0.81	-44.56	87.09	0.03	-42.02
10 uM	90.91	0.76	29.44	96.18	0.04	-34.36

Compound | Normalized Values (A and B Sets) | Responses

Silodosin

Average Response

Dose	Green Signal	Actual Pct Green Cells	Normalized Pct Green Cells	Red Signal	Actual Pct Red Cells	Normalized Pct Red Cells

Compound | Normalized Values (A and B Sets) | Responses

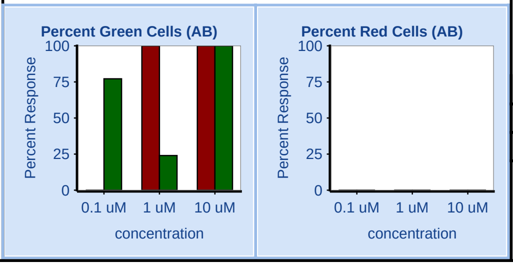

0.1 uM	93.79	0.84	32.14	86.56	0.05	-49.54
1 uM	103.37	0.86	96.70	87.63	0.04	-39.81
10 uM	112.57	0.89	111.89	99.94	0.07	-25.79

Compound | Normalized Values (A and B Sets) | Responses

Silymarin

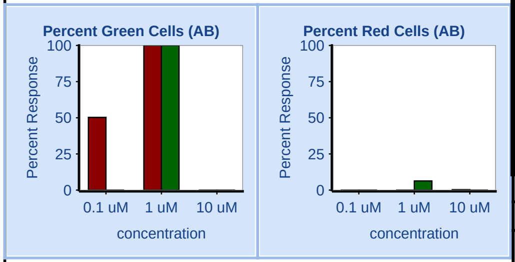

Average Response						
Dose	Green Signal	Actual Pct Green Cells	Normalized Pct Green Cells	Red Signal	Actual Pct Red Cells	Normalized Pct Red Cells
0.1 uM	89.52	0.81	9.57	84.06	0.06	-27.20
1 uM	110.65	0.91	109.43	104.86	0.11	-4.64
10 uM	92.04	0.84	-182.75	93.76	0.07	-22.57

Compound | Normalized Values (A and B Sets) | Responses

Sirtinol

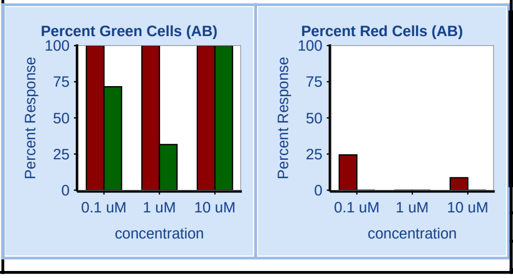

Average Response						
Dose	Green Signal	Actual Pct Green Cells	Normalized Pct Green Cells	Red Signal	Actual Pct Red Cells	Normalized Pct Red Cells
0.1 uM	102.72	0.92	117.39	99.82	0.12	1.80
1 uM	102.21	0.85	70.57	82.79	0.06	-27.03
10 uM	103.56	0.96	341.57	111.36	0.13	2.94

Compound | Normalized Values (A and B Sets) | Responses

Sitafloxacin Hydrate

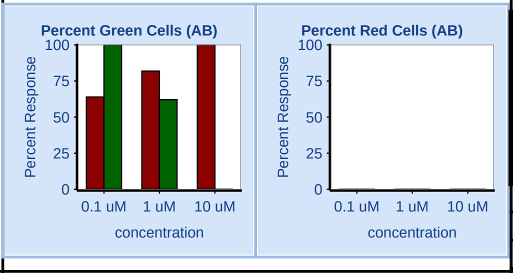

Average Response						
Dose	Green Signal	Actual Pct Green Cells	Normalized Pct Green Cells	Red Signal	Actual Pct Red Cells	Normalized Pct Red Cells
0.1 uM	87.36	0.88	85.49	90.40	0.04	-37.21
1 uM	94.33	0.84	72.00	65.25	0.01	-52.70
10 uM	96.75	0.89	297.88	96.57	0.07	-24.37

Compound | Normalized Values (A and B Sets) | Responses

Sitagliptin phosphate monohydrate

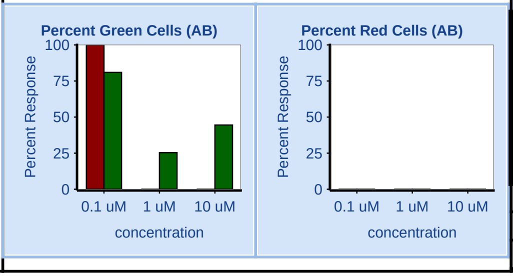

Average Response						
Dose	Green Signal	Actual Pct Green Cells	Normalized Pct Green Cells	Red Signal	Actual Pct Red Cells	Normalized Pct Red Cells
0.1 uM	90.68	0.89	123.81	73.32	0.02	-51.56
1 uM	84.42	0.73	6.63	66.82	0.01	-62.54
10 uM	86.60	0.73	-65.95	82.69	0.04	-43.51

Compound | Normalized Values (A and B Sets) | Responses

Sitaxentan sodium

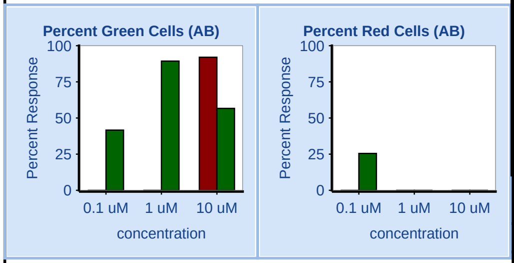

Average Response						
Dose	Green Signal	Actual Pct Green Cells	Normalized Pct Green Cells	Red Signal	Actual Pct Red Cells	Normalized Pct Red Cells
0.1 uM	84.34	0.78	-245.20	84.94	0.09	-17.57
1 uM	93.85	0.77	41.20	76.68	0.03	-51.39
10 uM	97.96	0.83	74.34	93.21	0.07	-26.84

Compound | Normalized Values (A and B Sets) | Responses

SJ 172550

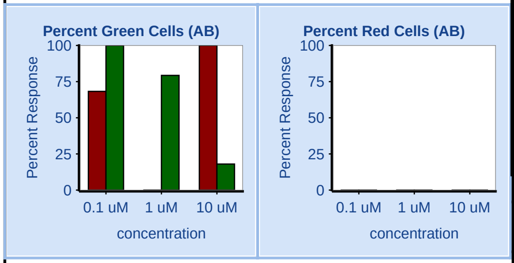

Average Response						
Dose	Green Signal	Actual Pct Green Cells	Normalized Pct Green Cells	Red Signal	Actual Pct Red Cells	Normalized Pct Red Cells
0.1 uM	103.34	0.89	98.84	72.53	0.01	-68.25
1 uM	89.13	0.83	-319.55	70.98	0.02	-41.85
10 uM	92.49	0.89	72.59	76.72	0.03	-36.11

Compound | Normalized Values (A and B Sets) | Responses

SK1-I

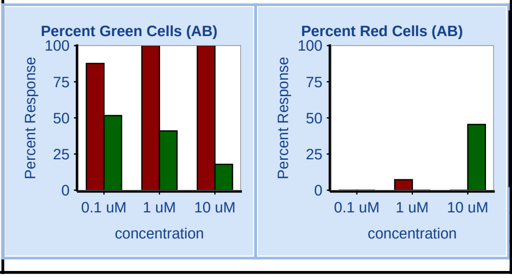

Average Response						
Dose	Green Signal	Actual Pct Green Cells	Normalized Pct Green Cells	Red Signal	Actual Pct Red Cells	Normalized Pct Red Cells
0.1 uM	92.49	0.85	69.62	76.54	0.02	-62.32
1 uM	94.67	0.87	96.79	87.24	0.06	-27.67
10 uM	96.47	0.90	90.08	87.78	0.12	-1.17

Compound | Normalized Values (A and B Sets) | Responses

Skepinone-L

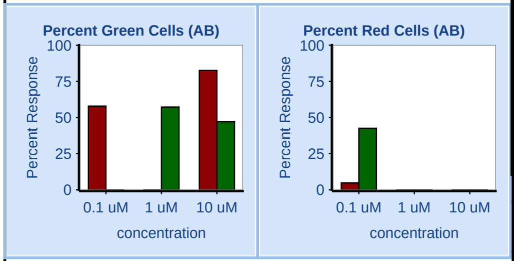

Average Response						
Dose	Green Signal	Actual Pct Green Cells	Normalized Pct Green Cells	Red Signal	Actual Pct Red Cells	Normalized Pct Red Cells
0.1 uM	92.04	0.79	13.24	111.39	0.16	23.70
1 uM	84.79	0.74	-814.36	81.20	0.05	-30.86
10 uM	94.12	0.88	64.83	72.69	0.04	-33.47

Compound | Normalized Values (A and B Sets) | Responses

SKF 91488

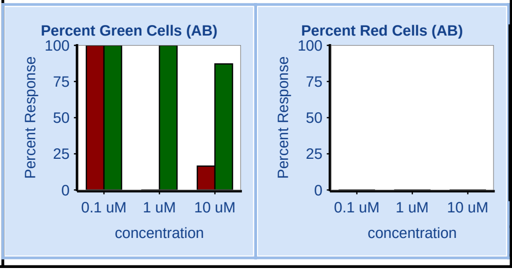

Average Response						
Dose	Green Signal	Actual Pct Green Cells	Normalized Pct Green Cells	Red Signal	Actual Pct Red Cells	Normalized Pct Red Cells
0.1 uM	103.91	0.92	125.16	84.81	0.04	-51.32
1 uM	99.50	0.90	-35.73	75.07	0.02	-38.78

Compound	Normalized Values (A and B Sets)	Responses

Dose	Green Signal	Actual Pct Green Cells	Normalized Pct Green Cells	Red Signal	Actual Pct Red Cells	Normalized Pct Red Cells
10 uM	92.04	0.87	51.84	69.76	0.02	-42.59

Compound	Normalized Values (A and B Sets)	Responses

SKI II

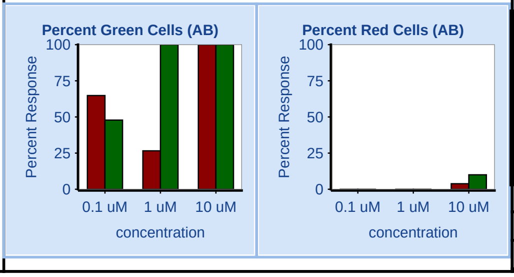

Average Response

Dose	Green Signal	Actual Pct Green Cells	Normalized Pct Green Cells	Red Signal	Actual Pct Red Cells	Normalized Pct Red Cells
0.1 uM	92.40	0.84	56.32	72.16	0.02	-63.01
1 uM	99.43	0.90	70.68	82.49	0.06	-14.94
10 uM	124.19	0.97	149.55	95.33	0.13	6.91

Compound	Normalized Values (A and B Sets)	Responses

SMER 3

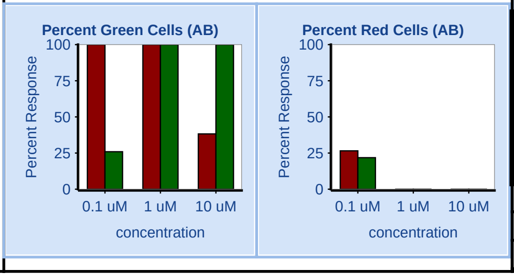

Average Response

Dose	Green Signal	Actual Pct Green Cells	Normalized Pct Green Cells	Red Signal	Actual Pct Red Cells	Normalized Pct Red Cells
0.1 uM	93.82	0.86	77.38	113.74	0.16	24.17
1 uM	101.14	0.94	264.55	85.26	0.07	-10.97
10 uM	103.48	0.92	94.82	88.91	0.07	-21.00

Compound	Normalized Values (A and B Sets)	Responses

SMI-4a

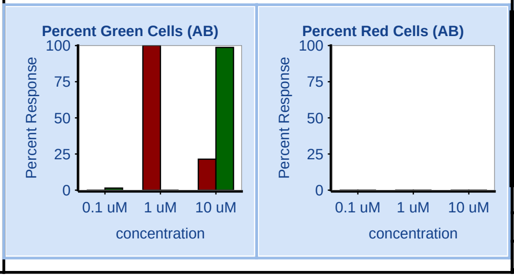

Average Response

Dose	Green Signal	Actual Pct Green Cells	Normalized Pct Green Cells	Red Signal	Actual Pct Red Cells	Normalized Pct Red Cells
0.1 uM	87.74	0.75	-24.11	82.59	0.05	-46.20
1 uM	94.50	0.84	126.34	84.26	0.06	-16.45
10 uM	91.77	0.88	60.06	75.88	0.03	-38.81

Compound	Normalized Values (A and B Sets)	Responses

SN-38

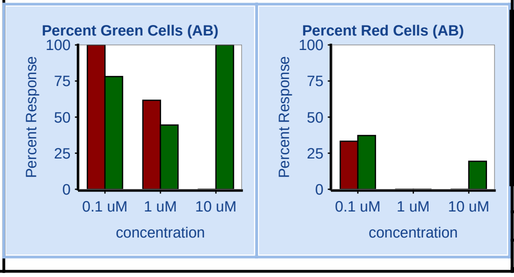

Average Response

Dose	Green Signal	Actual Pct Green Cells	Normalized Pct Green Cells	Red Signal	Actual Pct Red Cells	Normalized Pct Red Cells
0.1 uM	113.75	0.89	119.97	111.88	0.20	35.26
1 uM	97.43	0.79	53.08	92.83	0.09	-21.44
10 uM	107.68	0.78	-75.31	79.96	0.09	-18.57

Compound	Normalized Values (A and B Sets)	Responses

SNS-032 (BMS-387032)

Average Response

Dose	Green Signal	Actual Pct Green Cells	Normalized Pct Green Cells	Red Signal	Actual Pct Red Cells	Normalized Pct Red Cells

Compound	Normalized Values (A and B Sets)	Responses

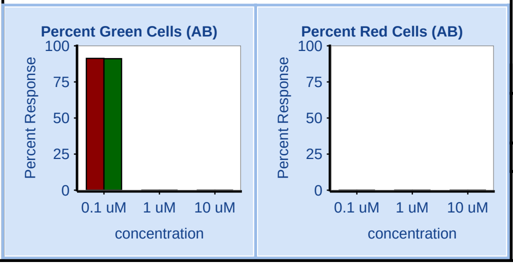

0.1 uM	97.47	0.89	91.20	85.45	0.03	-55.37
1 uM	77.21	0.55	-378.34	92.35	0.10	-11.88
10 uM	85.61	0.62	-70.62	106.36	0.10	-9.16

Compound	Normalized Values (A and B Sets)	Responses

SNS-314 Mesylate

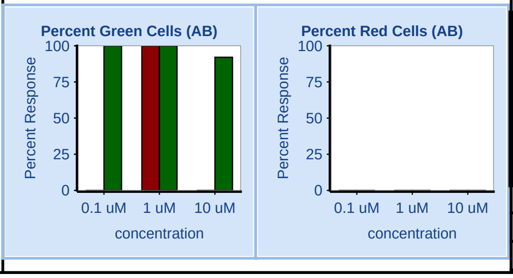

Average Response

Dose	Green Signal	Actual Pct Green Cells	Normalized Pct Green Cells	Red Signal	Actual Pct Red Cells	Normalized Pct Red Cells
0.1 uM	98.30	0.88	43.45	92.10	0.06	-41.33
1 uM	106.59	0.93	150.87	86.18	0.05	-33.58
10 uM	92.37	0.78	42.91	85.39	0.02	-43.71

Compound	Normalized Values (A and B Sets)	Responses

SNX-2112 (PF-04928473)

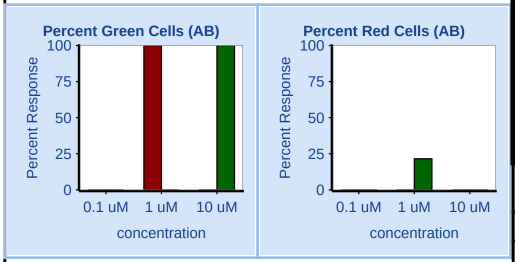

Average Response

Dose	Green Signal	Actual Pct Green Cells	Normalized Pct Green Cells	Red Signal	Actual Pct Red Cells	Normalized Pct Red Cells
0.1 uM	82.25	0.68	-121.82	91.59	0.11	-5.27
1 uM	105.75	0.84	60.00	94.17	0.13	0.05
10 uM	108.00	0.86	-287.97	76.92	0.06	-28.49

Compound	Normalized Values (A and B Sets)	Responses

Sodium 4-aminohippurate Hydrate

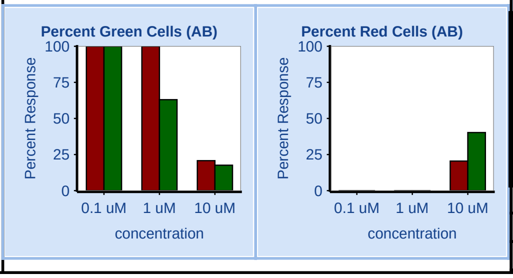

Average Response

Dose	Green Signal	Actual Pct Green Cells	Normalized Pct Green Cells	Red Signal	Actual Pct Red Cells	Normalized Pct Red Cells
0.1 uM	102.44	0.93	221.08	87.25	0.06	-35.41
1 uM	104.31	0.87	103.85	98.76	0.11	-11.50
10 uM	91.42	0.76	19.20	117.52	0.18	30.37

Compound	Normalized Values (A and B Sets)	Responses

Sodium ascorbate

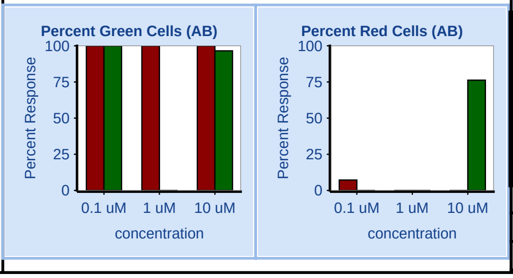

Average Response

Dose	Green Signal	Actual Pct Green Cells	Normalized Pct Green Cells	Red Signal	Actual Pct Red Cells	Normalized Pct Red Cells
0.1 uM	111.18	0.96	222.77	92.71	0.09	-18.31
1 uM	88.40	0.74	6.18	93.48	0.08	-26.08
10 uM	110.00	0.91	150.04	109.45	0.16	20.96

Compound | Normalized Values (A and B Sets) | Responses

Sodium Danshensu

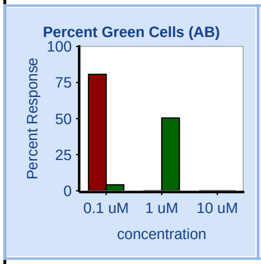

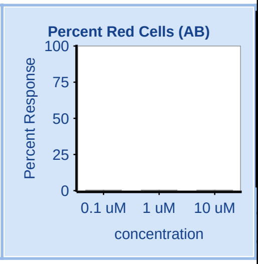

Average Response

Dose	Green Signal	Actual Pct Green Cells	Normalized Pct Green Cells	Red Signal	Actual Pct Red Cells	Normalized Pct Red Cells
0.1 uM	88.61	0.84	42.40	76.17	0.03	-41.57
1 uM	86.76	0.71	9.66	85.62	0.05	-34.96
10 uM	87.66	0.84	-40.43	96.44	0.08	-21.22

Compound | Normalized Values (A and B Sets) | Responses

Sodium Nitrite

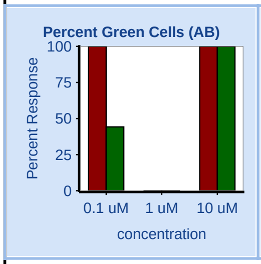

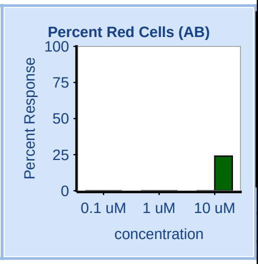

Average Response

Dose	Green Signal	Actual Pct Green Cells	Normalized Pct Green Cells	Red Signal	Actual Pct Red Cells	Normalized Pct Red Cells
0.1 uM	95.42	0.86	86.11	70.85	0.03	-46.11
1 uM	82.09	0.68	-33.34	79.37	0.03	-50.31
10 uM	108.21	0.92	148.16	99.45	0.11	-7.63

Compound | Normalized Values (A and B Sets) | Responses

Sodium Nitroprusside

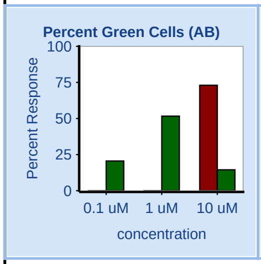

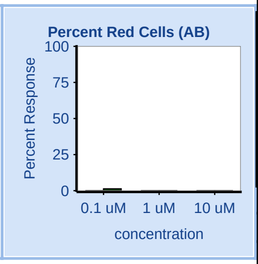

Average Response

Dose	Green Signal	Actual Pct Green Cells	Normalized Pct Green Cells	Red Signal	Actual Pct Red Cells	Normalized Pct Red Cells
0.1 uM	86.22	0.81	-84.25	85.56	0.06	-30.01
1 uM	86.73	0.70	-11.29	77.66	0.04	-47.76
10 uM	92.03	0.78	43.76	83.13	0.04	-43.52

Compound | Normalized Values (A and B Sets) | Responses

Sodium Phenylbutyrate

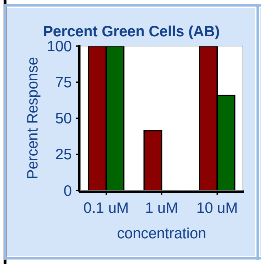

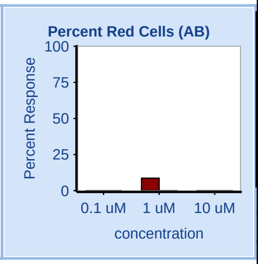

Average Response

Dose	Green Signal	Actual Pct Green Cells	Normalized Pct Green Cells	Red Signal	Actual Pct Red Cells	Normalized Pct Red Cells
0.1 uM	102.06	0.94	157.88	76.66	0.03	-48.33
1 uM	87.58	0.74	11.54	93.45	0.08	-23.54
10 uM	109.07	0.89	159.12	88.82	0.06	-31.11

Compound | Normalized Values (A and B Sets) | Responses

Sodium Picosulfate

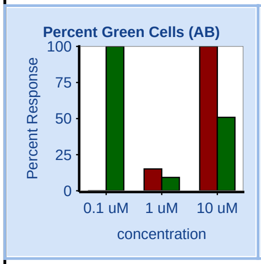

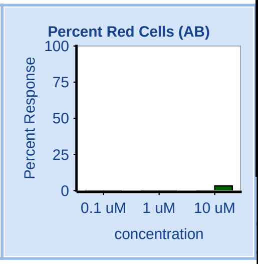

Average Response

Dose	Green Signal	Actual Pct Green Cells	Normalized Pct Green Cells	Red Signal	Actual Pct Red Cells	Normalized Pct Red Cells
0.1 uM	89.01	0.82	-250.26	78.75	0.04	-44.94
1 uM	85.34	0.74	12.18	76.64	0.02	-56.27
10 uM	92.92	0.83	76.52	83.33	0.06	-30.87

Compound	Normalized Values (A and B Sets)	Responses

Sodium salicylate

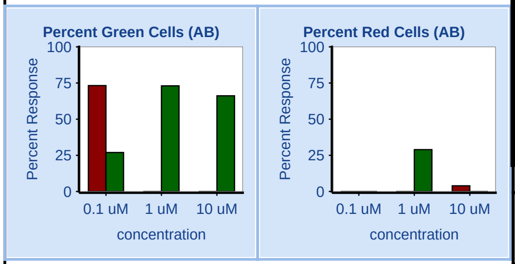

Average Response

Dose	Green Signal	Actual Pct Green Cells	Normalized Pct Green Cells	Red Signal	Actual Pct Red Cells	Normalized Pct Red Cells
0.1 uM	92.11	0.83	50.09	82.38	0.03	-53.92
1 uM	76.71	0.59	-1867.36	90.83	0.09	3.84
10 uM	88.95	0.84	24.17	98.99	0.11	-2.38

Compound	Normalized Values (A and B Sets)	Responses

Solifenacin succinate

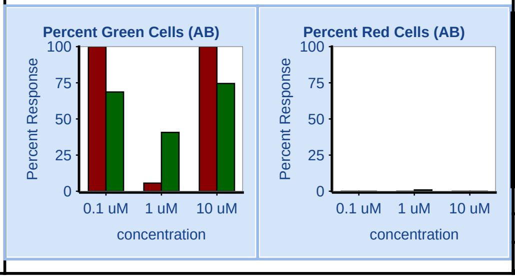

Average Response

Dose	Green Signal	Actual Pct Green Cells	Normalized Pct Green Cells	Red Signal	Actual Pct Red Cells	Normalized Pct Red Cells
0.1 uM	99.79	0.90	177.11	84.39	0.06	-33.02
1 uM	90.50	0.75	23.16	87.82	0.08	-30.19
10 uM	96.50	0.87	109.54	92.85	0.04	-40.35

Compound	Normalized Values (A and B Sets)	Responses

Sophocarpine

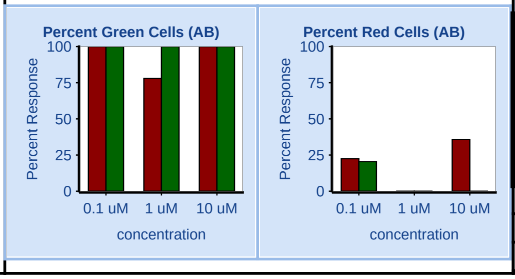

Average Response

Dose	Green Signal	Actual Pct Green Cells	Normalized Pct Green Cells	Red Signal	Actual Pct Red Cells	Normalized Pct Red Cells
0.1 uM	99.24	0.92	125.60	109.69	0.16	21.44
1 uM	107.31	0.90	104.99	90.73	0.08	-20.78
10 uM	116.04	0.96	516.07	110.12	0.14	8.35

Compound	Normalized Values (A and B Sets)	Responses

Sorafenib Tosylate

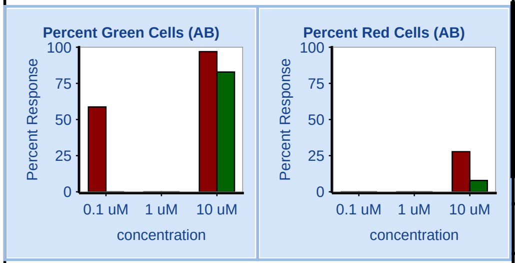

Average Response

Dose	Green Signal	Actual Pct Green Cells	Normalized Pct Green Cells	Red Signal	Actual Pct Red Cells	Normalized Pct Red Cells
0.1 uM	86.92	0.78	15.51	104.79	0.08	-33.62
1 uM	74.28	0.60	-117.27	74.56	0.02	-48.74
10 uM	106.53	0.86	89.92	131.00	0.17	17.82

Compound	Normalized Values (A and B Sets)	Responses

Sotrastaurin

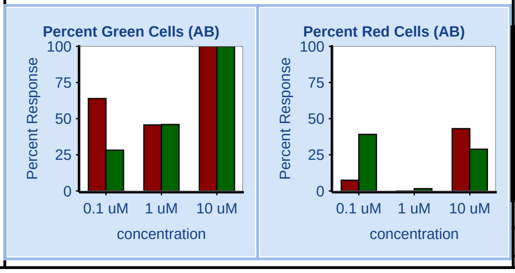

Average Response

Dose	Green Signal	Actual Pct Green Cells	Normalized Pct Green Cells	Red Signal	Actual Pct Red Cells	Normalized Pct Red Cells
0.1 uM	92.82	0.84	46.06	106.02	0.16	23.29
1 uM	91.05	0.79	45.81	97.16	0.11	-5.21

Compound | Normalized Values (A and B Sets) | Responses

Dose	Green Signal	Actual Pct Green Cells	Normalized Pct Green Cells	Red Signal	Actual Pct Red Cells	Normalized Pct Red Cells
10 uM	107.59	0.94	266.63	116.29	0.20	35.99

Compound | Normalized Values (A and B Sets) | Responses

Spiramycin

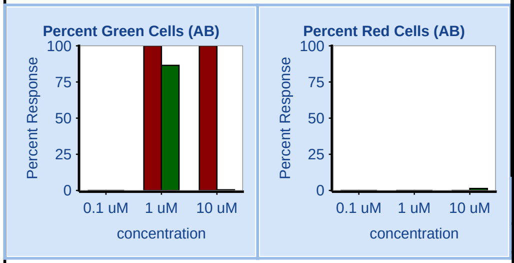

Average Response

Dose	Green Signal	Actual Pct Green Cells	Normalized Pct Green Cells	Red Signal	Actual Pct Red Cells	Normalized Pct Red Cells
0.1 uM	74.94	0.64	-380.67	73.04	0.05	-35.34
1 uM	98.41	0.87	110.69	86.87	0.07	-34.16
10 uM	86.32	0.79	70.97	88.39	0.06	-31.81

Compound | Normalized Values (A and B Sets) | Responses

Src I1

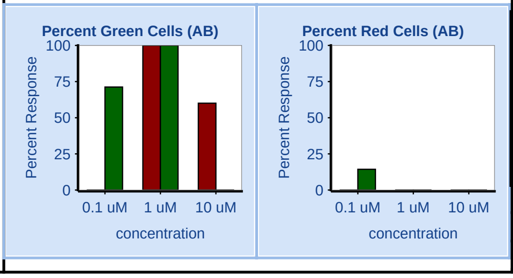

Average Response

Dose	Green Signal	Actual Pct Green Cells	Normalized Pct Green Cells	Red Signal	Actual Pct Red Cells	Normalized Pct Red Cells
0.1 uM	85.62	0.78	-4.86	94.89	0.08	-25.21
1 uM	102.08	0.91	151.64	67.97	0.01	-41.71
10 uM	88.39	0.83	16.91	67.68	0.02	-43.85

Compound | Normalized Values (A and B Sets) | Responses

SRT1720

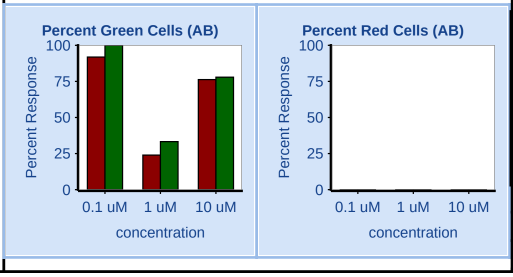

Average Response

Dose	Green Signal	Actual Pct Green Cells	Normalized Pct Green Cells	Red Signal	Actual Pct Red Cells	Normalized Pct Red Cells
0.1 uM	103.27	0.91	100.43	100.31	0.07	-37.08
1 uM	89.81	0.82	28.61	86.01	0.03	-44.51
10 uM	85.07	0.84	77.09	95.27	0.00	-52.84

Compound | Normalized Values (A and B Sets) | Responses

SSR128129E

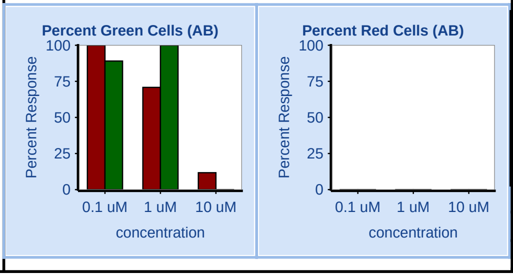

Average Response

Dose	Green Signal	Actual Pct Green Cells	Normalized Pct Green Cells	Red Signal	Actual Pct Red Cells	Normalized Pct Red Cells
0.1 uM	109.33	0.92	239.01	75.59	0.04	-44.13
1 uM	106.86	0.87	111.62	86.78	0.05	-39.33
10 uM	76.38	0.58	-70.39	77.83	0.03	-48.22

Compound | Normalized Values (A and B Sets) | Responses

STAT5 Inhibitor

Average Response

Dose	Green Signal	Actual Pct Green Cells	Normalized Pct Green Cells	Red Signal	Actual Pct Red Cells	Normalized Pct Red Cells

Compound	Normalized Values (A and B Sets)	Responses

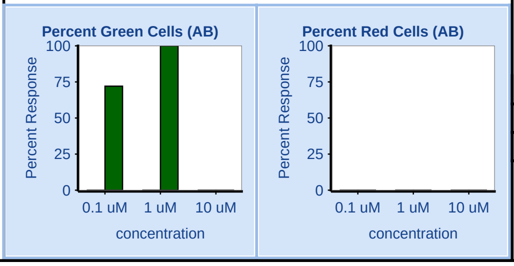

0.1 uM	85.94	0.77	-9.61	75.17	0.02	-65.36
1 uM	99.80	0.93	83.06	92.76	0.05	-20.45
10 uM	77.37	0.71	-93.96	74.78	0.04	-33.07

Compound	Normalized Values (A and B Sets)	Responses

Stattic

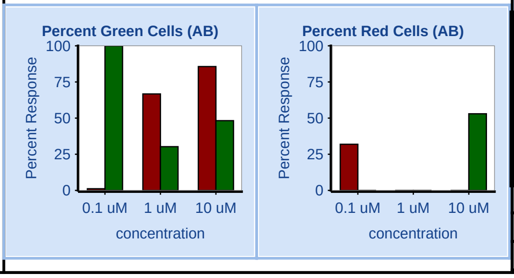

Average Response

Dose	Green Signal	Actual Pct Green Cells	Normalized Pct Green Cells	Red Signal	Actual Pct Red Cells	Normalized Pct Red Cells
0.1 uM	104.77	0.91	70.31	98.24	0.12	-3.93
1 uM	96.62	0.79	48.47	78.71	0.06	-35.65
10 uM	110.14	0.82	66.94	106.65	0.14	7.77

Compound	Normalized Values (A and B Sets)	Responses

Stavudine (d4T)

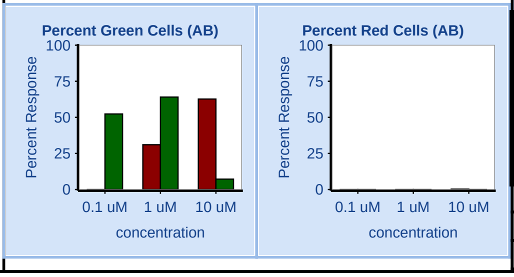

Average Response

Dose	Green Signal	Actual Pct Green Cells	Normalized Pct Green Cells	Red Signal	Actual Pct Red Cells	Normalized Pct Red Cells
0.1 uM	87.65	0.80	-4.10	94.79	0.05	-46.88
1 uM	96.09	0.85	47.54	84.35	0.03	-42.27
10 uM	91.44	0.78	34.94	109.65	0.08	-19.67

Compound	Normalized Values (A and B Sets)	Responses

StemRegenin 1 (SR1)

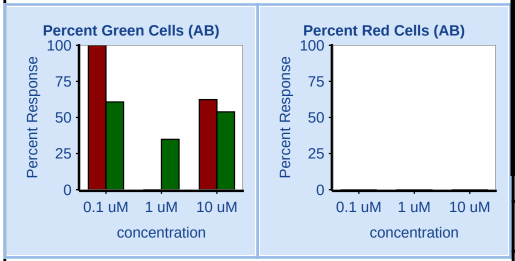

Average Response

Dose	Green Signal	Actual Pct Green Cells	Normalized Pct Green Cells	Red Signal	Actual Pct Red Cells	Normalized Pct Red Cells
0.1 uM	104.97	0.87	86.66	91.40	0.08	-25.79
1 uM	84.70	0.78	-509.03	63.70	0.02	-41.29
10 uM	88.34	0.87	58.13	79.85	0.04	-35.27

Compound	Normalized Values (A and B Sets)	Responses

Stigmasterol

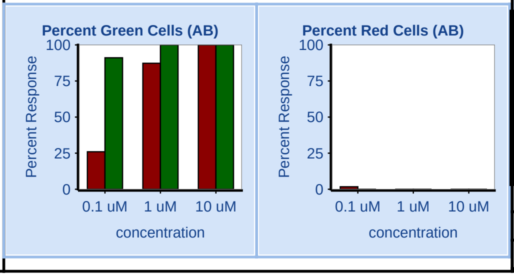

Average Response

Dose	Green Signal	Actual Pct Green Cells	Normalized Pct Green Cells	Red Signal	Actual Pct Red Cells	Normalized Pct Red Cells
0.1 uM	90.53	0.85	58.54	92.72	0.09	-12.53
1 uM	105.81	0.89	100.71	89.85	0.07	-24.95
10 uM	99.68	0.93	207.43	102.06	0.10	-12.63

Compound	Normalized Values (A and B Sets)	Responses

Streptomycin sulfate

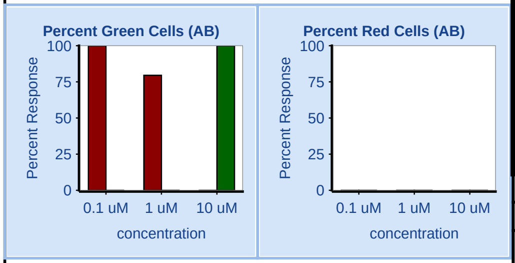

Average Response

Dose	Green Signal	Actual Pct Green Cells	Normalized Pct Green Cells	Red Signal	Actual Pct Red Cells	Normalized Pct Red Cells
0.1 uM	91.83	0.83	22.89	90.14	0.06	-26.76
1 uM	81.47	0.64	-54.90	78.04	0.02	-45.40
10 uM	99.42	0.89	-101.36	80.80	0.03	-42.03

Compound	Normalized Values (A and B Sets)	Responses

Succinylcholine Chloride Dihydrate

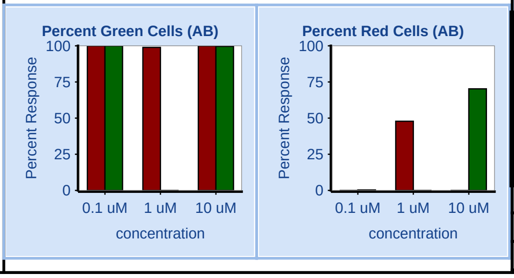

Average Response

Dose	Green Signal	Actual Pct Green Cells	Normalized Pct Green Cells	Red Signal	Actual Pct Red Cells	Normalized Pct Red Cells
0.1 uM	115.56	0.96	282.14	75.35	0.06	-30.42
1 uM	96.76	0.79	47.99	110.67	0.17	23.07
10 uM	105.43	0.90	142.00	116.49	0.18	32.22

Compound	Normalized Values (A and B Sets)	Responses

Sulbactam sodium

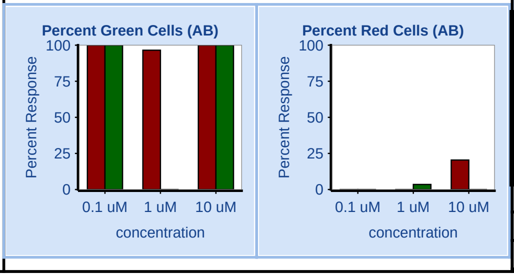

Average Response

Dose	Green Signal	Actual Pct Green Cells	Normalized Pct Green Cells	Red Signal	Actual Pct Red Cells	Normalized Pct Red Cells
0.1 uM	101.90	0.93	132.49	88.41	0.07	-23.54
1 uM	93.15	0.80	41.71	100.61	0.10	-10.76
10 uM	116.27	0.98	514.32	104.35	0.10	-12.00

Compound	Normalized Values (A and B Sets)	Responses

Sulfacetamide Sodium

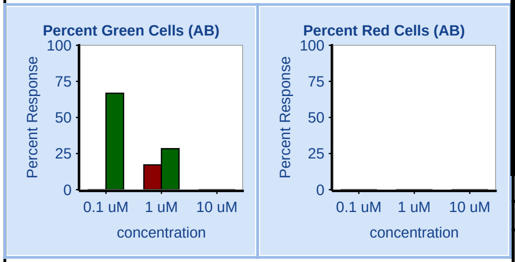

Average Response

Dose	Green Signal	Actual Pct Green Cells	Normalized Pct Green Cells	Red Signal	Actual Pct Red Cells	Normalized Pct Red Cells
0.1 uM	92.23	0.86	18.57	82.48	0.04	-43.18
1 uM	88.73	0.75	22.76	75.44	0.03	-54.35
10 uM	72.60	0.55	-203.83	95.17	0.08	-23.92

Compound	Normalized Values (A and B Sets)	Responses

Sunitinib Malate

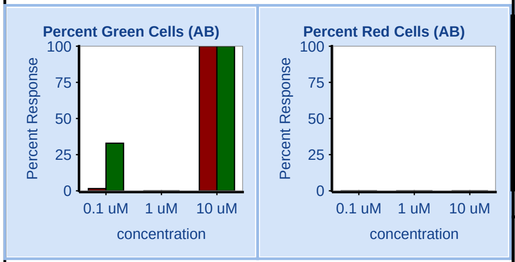

Average Response

Dose	Green Signal	Actual Pct Green Cells	Normalized Pct Green Cells	Red Signal	Actual Pct Red Cells	Normalized Pct Red Cells
0.1 uM	86.27	0.81	17.23	78.81	0.02	-63.80
1 uM	77.21	0.64	-183.06	107.23	0.05	-32.41

Compound	Normalized Values (A and B Sets)	Responses

Dose	Green Signal	Actual Pct Green Cells	Normalized Pct Green Cells	Red Signal	Actual Pct Red Cells	Normalized Pct Red Cells
10 uM	203.30	0.98	169.03	107.05	0.06	-27.00

Compound	Normalized Values (A and B Sets)	Responses

Suplatast Tosylate

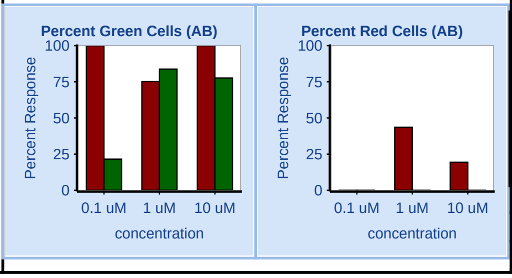

Average Response

Dose	Green Signal	Actual Pct Green Cells	Normalized Pct Green Cells	Red Signal	Actual Pct Red Cells	Normalized Pct Red Cells
0.1 uM	104.26	0.85	83.81	87.16	0.05	-44.92
1 uM	98.87	0.88	79.48	109.09	0.12	-1.89
10 uM	103.41	0.87	92.62	107.37	0.10	-15.66

Compound	Normalized Values (A and B Sets)	Responses

Suvorexant (MK-4305)

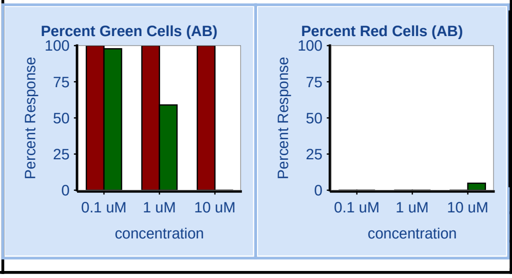

Average Response

Dose	Green Signal	Actual Pct Green Cells	Normalized Pct Green Cells	Red Signal	Actual Pct Red Cells	Normalized Pct Red Cells
0.1 uM	99.75	0.91	119.18	72.05	0.01	-69.91
1 uM	106.66	0.90	222.38	69.25	0.02	-40.10
10 uM	86.35	0.86	44.23	66.94	0.06	-24.41

Compound	Normalized Values (A and B Sets)	Responses

Synephrine HCl

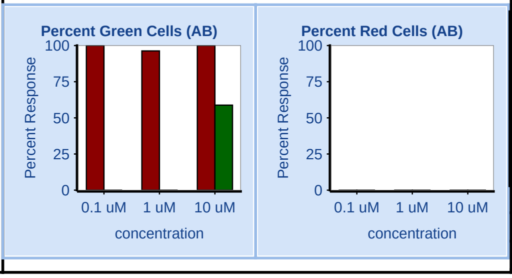

Average Response

Dose	Green Signal	Actual Pct Green Cells	Normalized Pct Green Cells	Red Signal	Actual Pct Red Cells	Normalized Pct Red Cells
0.1 uM	91.05	0.77	-42.78	76.97	0.03	-42.20
1 uM	96.00	0.80	42.75	79.12	0.04	-36.46
10 uM	100.28	0.90	199.00	86.94	0.06	-29.81

Compound	Normalized Values (A and B Sets)	Responses

T0070907

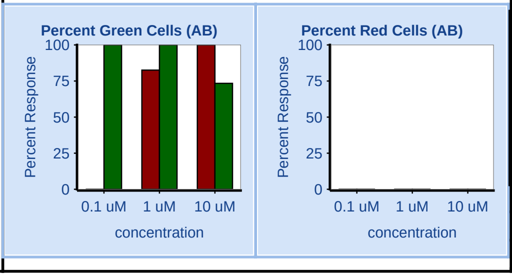

Average Response

Dose	Green Signal	Actual Pct Green Cells	Normalized Pct Green Cells	Red Signal	Actual Pct Red Cells	Normalized Pct Red Cells
0.1 uM	94.56	0.91	9.08	65.38	0.02	-55.90
1 uM	98.67	0.88	120.18	79.36	0.03	-51.87
10 uM	102.99	0.89	144.20	71.01	0.02	-54.79

Compound	Normalized Values (A and B Sets)	Responses

T0901317

Average Response

Dose	Green Signal	Actual Pct Green Cells	Normalized Pct Green Cells	Red Signal	Actual Pct Red Cells	Normalized Pct Red Cells

Compound	Normalized Values (A and B Sets)	Responses

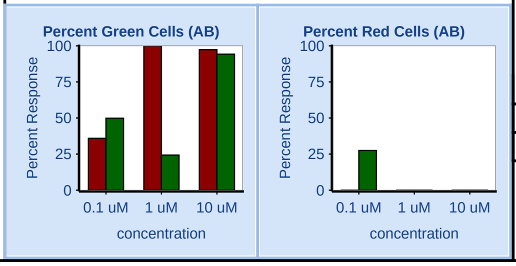

0.1 uM	89.75	0.86	42.89	88.37	0.09	-14.95
1 uM	105.17	0.87	102.76	97.01	0.10	-16.34
10 uM	95.38	0.87	95.79	84.47	0.04	-43.92

Compound	Normalized Values (A and B Sets)	Responses

TAE226 (NVP-TAE226)

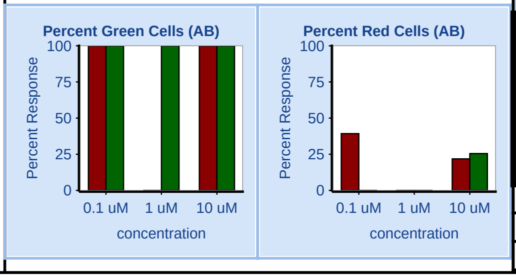

Average Response

Dose	Green Signal	Actual Pct Green Cells	Normalized Pct Green Cells	Red Signal	Actual Pct Red Cells	Normalized Pct Red Cells
0.1 uM	108.93	0.95	162.06	94.20	0.11	-6.64
1 uM	88.84	0.78	49.25	79.44	0.04	-37.30
10 uM	113.87	0.95	470.80	113.96	0.17	23.64

Compound	Normalized Values (A and B Sets)	Responses

TAK-285

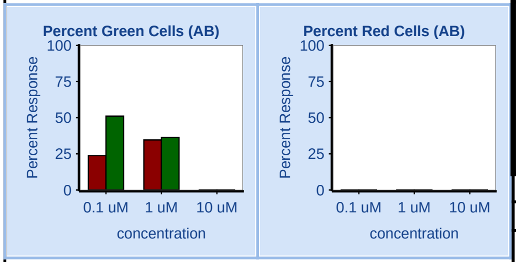

Average Response

Dose	Green Signal	Actual Pct Green Cells	Normalized Pct Green Cells	Red Signal	Actual Pct Red Cells	Normalized Pct Red Cells
0.1 uM	91.61	0.83	37.44	79.34	0.05	-30.28
1 uM	88.69	0.77	35.54	76.95	0.03	-42.30
10 uM	82.48	0.82	-279.10	89.55	0.06	-31.62

Compound	Normalized Values (A and B Sets)	Responses

TAK-438

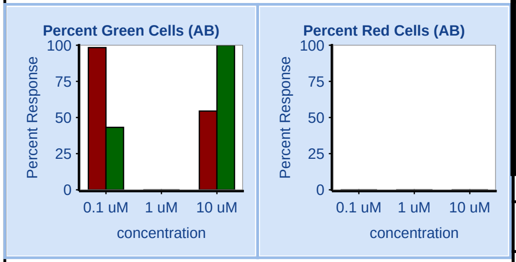

Average Response

Dose	Green Signal	Actual Pct Green Cells	Normalized Pct Green Cells	Red Signal	Actual Pct Red Cells	Normalized Pct Red Cells
0.1 uM	93.84	0.85	70.79	87.41	0.04	-50.42
1 uM	70.85	0.61	-893.04	73.53	0.04	-23.62
10 uM	93.84	0.92	94.96	74.83	0.03	-39.25

Compound	Normalized Values (A and B Sets)	Responses

TAK-632

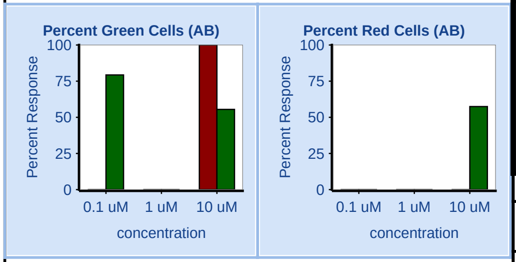

Average Response

Dose	Green Signal	Actual Pct Green Cells	Normalized Pct Green Cells	Red Signal	Actual Pct Red Cells	Normalized Pct Red Cells
0.1 uM	84.86	0.80	17.10	71.62	0.03	-56.47
1 uM	80.21	0.75	-332.25	66.74	0.02	-37.51
10 uM	85.75	0.91	94.64	95.98	0.14	9.89

Compound | Normalized Values (A and B Sets) | Responses

TAK-700 (Orteronel)

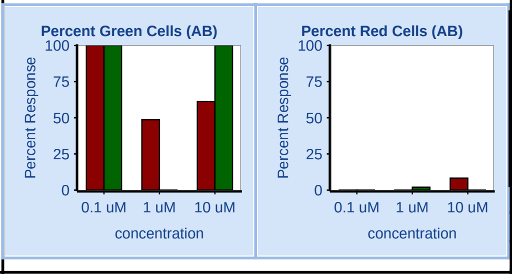

Average Response

Dose	Green Signal	Actual Pct Green Cells	Normalized Pct Green Cells	Red Signal	Actual Pct Red Cells	Normalized Pct Red Cells
0.1 uM	108.14	0.92	111.47	99.10	0.09	-21.40
1 uM	95.43	0.75	-6.58	107.56	0.10	-9.34
10 uM	98.36	0.85	83.81	115.14	0.11	-8.92

Compound | Normalized Values (A and B Sets) | Responses

TAK-715

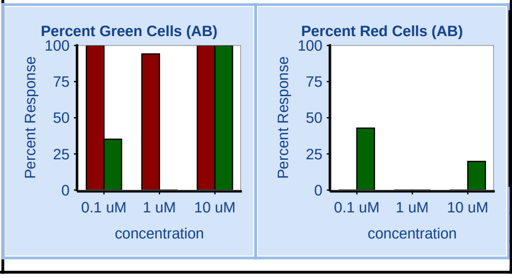

Average Response

Dose	Green Signal	Actual Pct Green Cells	Normalized Pct Green Cells	Red Signal	Actual Pct Red Cells	Normalized Pct Red Cells
0.1 uM	87.82	0.85	73.61	86.79	0.10	-10.10
1 uM	88.39	0.76	22.42	65.22	0.01	-62.76
10 uM	102.55	0.92	145.29	107.19	0.12	1.32

Compound | Normalized Values (A and B Sets) | Responses

TAK-733

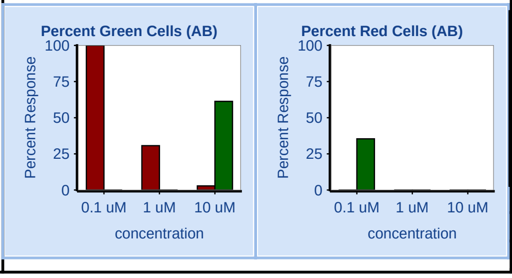

Average Response

Dose	Green Signal	Actual Pct Green Cells	Normalized Pct Green Cells	Red Signal	Actual Pct Red Cells	Normalized Pct Red Cells
0.1 uM	91.26	0.86	56.01	95.42	0.13	9.72
1 uM	86.89	0.72	6.63	81.07	0.07	-26.02
10 uM	84.04	0.88	32.09	85.46	0.03	-43.06

Compound | Normalized Values (A and B Sets) | Responses

TAK-875

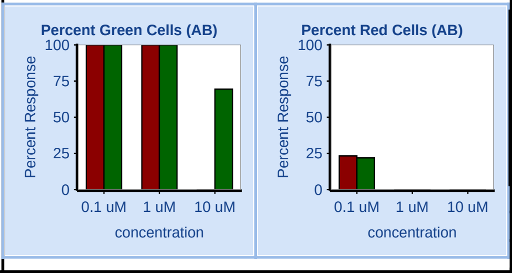

Average Response

Dose	Green Signal	Actual Pct Green Cells	Normalized Pct Green Cells	Red Signal	Actual Pct Red Cells	Normalized Pct Red Cells
0.1 uM	100.81	0.93	135.84	113.51	0.17	22.52
1 uM	113.61	0.93	116.93	91.73	0.08	-22.50
10 uM	94.51	0.88	27.74	79.48	0.04	-41.01

Compound | Normalized Values (A and B Sets) | Responses

TAK-901

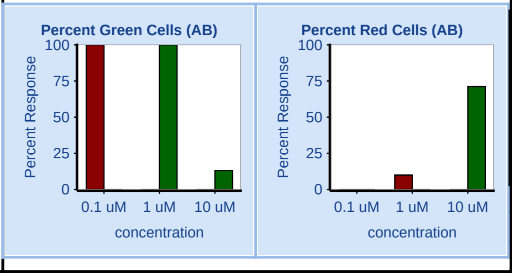

Average Response

Dose	Green Signal	Actual Pct Green Cells	Normalized Pct Green Cells	Red Signal	Actual Pct Red Cells	Normalized Pct Red Cells
0.1 uM	92.27	0.82	45.97	79.05	0.03	-55.48
1 uM	102.08	0.91	-81.85	93.17	0.10	-7.85
10 uM	99.77	0.81	-4.40	94.21	0.17	21.80

Compound	Normalized Values (A and B Sets)	Responses

Talc

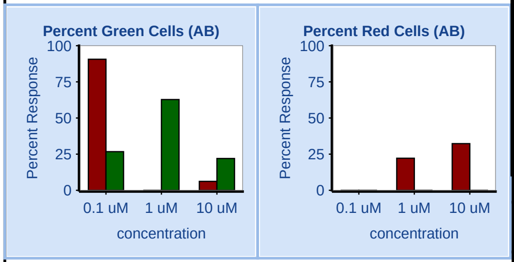

Average Response

Dose	Green Signal	Actual Pct Green Cells	Normalized Pct Green Cells	Red Signal	Actual Pct Red Cells	Normalized Pct Red Cells
0.1 uM	94.07	0.84	58.68	98.36	0.09	-28.16
1 uM	90.02	0.73	-133.29	117.36	0.14	8.54
10 uM	84.93	0.75	14.09	117.77	0.12	-8.84

Compound	Normalized Values (A and B Sets)	Responses

TAME

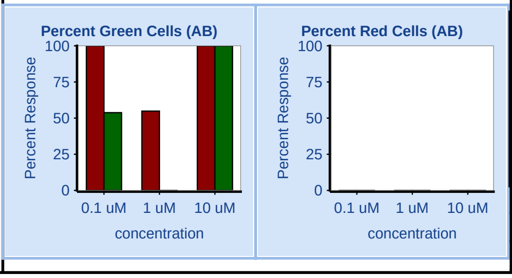

Average Response

Dose	Green Signal	Actual Pct Green Cells	Normalized Pct Green Cells	Red Signal	Actual Pct Red Cells	Normalized Pct Red Cells
0.1 uM	96.29	0.89	94.43	81.73	0.04	-35.66
1 uM	93.29	0.76	23.62	77.16	0.03	-40.76
10 uM	100.28	0.94	314.10	79.83	0.02	-49.90

Compound	Normalized Values (A and B Sets)	Responses

Tamibarotene

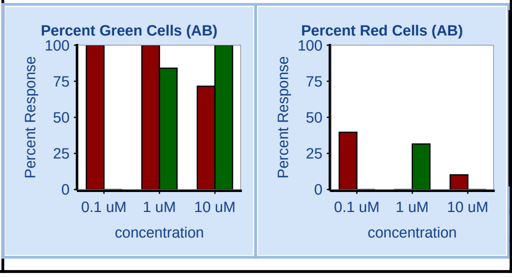

Average Response

Dose	Green Signal	Actual Pct Green Cells	Normalized Pct Green Cells	Red Signal	Actual Pct Red Cells	Normalized Pct Red Cells
0.1 uM	84.04	0.71	-33.17	97.38	0.12	-5.48
1 uM	104.95	0.88	113.75	99.36	0.13	-3.94
10 uM	102.37	0.87	88.28	107.76	0.13	4.60

Compound	Normalized Values (A and B Sets)	Responses

Tanshinone I

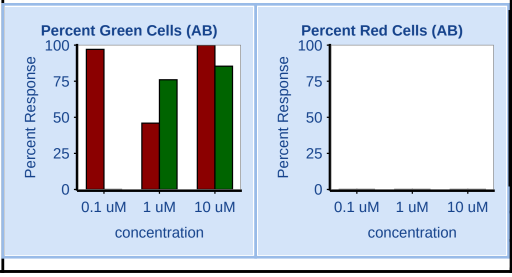

Average Response

Dose	Green Signal	Actual Pct Green Cells	Normalized Pct Green Cells	Red Signal	Actual Pct Red Cells	Normalized Pct Red Cells
0.1 uM	81.49	0.74	-72.62	75.41	0.03	-37.62
1 uM	92.32	0.81	60.98	76.38	0.02	-48.24
10 uM	94.77	0.92	289.56	77.33	0.02	-49.93

Compound	Normalized Values (A and B Sets)	Responses

Tariquidar

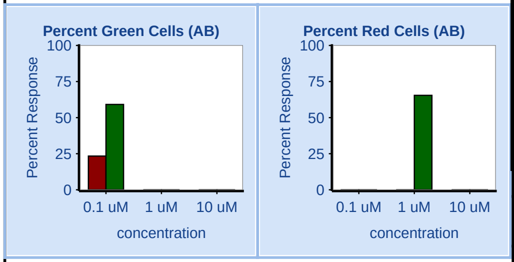

Average Response

Dose	Green Signal	Actual Pct Green Cells	Normalized Pct Green Cells	Red Signal	Actual Pct Red Cells	Normalized Pct Red Cells
0.1 uM	89.58	0.82	41.25	90.51	0.06	-39.96
1 uM	79.51	0.71	-748.04	91.79	0.11	18.83
10 uM	75.16	0.69		70.32	0.01	-45.15

Compound	Normalized Values (A and B Sets)	Responses

			-116.01			

Compound	Normalized Values (A and B Sets)	Responses

Tasisulam

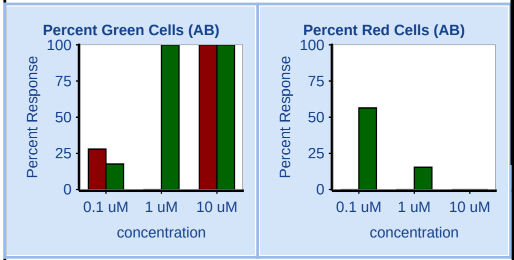

Average Response

Dose	Green Signal	Actual Pct Green Cells	Normalized Pct Green Cells	Red Signal	Actual Pct Red Cells	Normalized Pct Red Cells
0.1 uM	85.07	0.80	22.74	96.68	0.11	-8.98
1 uM	85.34	0.75	-1007.14	85.27	0.07	-5.42
10 uM	98.54	0.94	116.20	62.64	0.01	-46.01

Compound	Normalized Values (A and B Sets)	Responses

Tazarotene

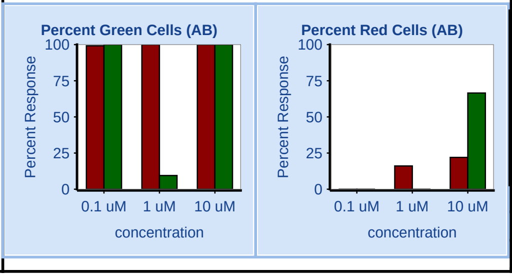

Average Response

Dose	Green Signal	Actual Pct Green Cells	Normalized Pct Green Cells	Red Signal	Actual Pct Red Cells	Normalized Pct Red Cells
0.1 uM	103.51	0.91	100.36	87.04	0.06	-45.45
1 uM	102.58	0.83	65.19	101.76	0.10	-14.78
10 uM	121.78	0.92	126.62	140.88	0.22	44.27

Compound	Normalized Values (A and B Sets)	Responses

TCID

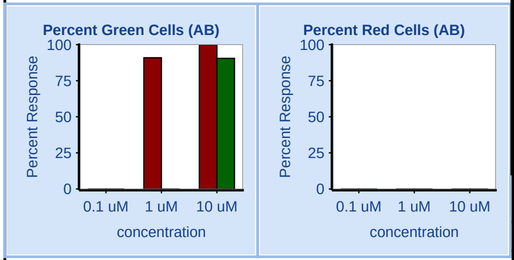

Average Response

Dose	Green Signal	Actual Pct Green Cells	Normalized Pct Green Cells	Red Signal	Actual Pct Red Cells	Normalized Pct Red Cells
0.1 uM	70.79	0.64	-668.06	77.31	0.05	-39.48
1 uM	87.24	0.73	0.73	72.35	0.03	-52.24
10 uM	105.22	0.89	127.40	92.40	0.07	-26.64

Compound	Normalized Values (A and B Sets)	Responses

TCS PIM-1 4a

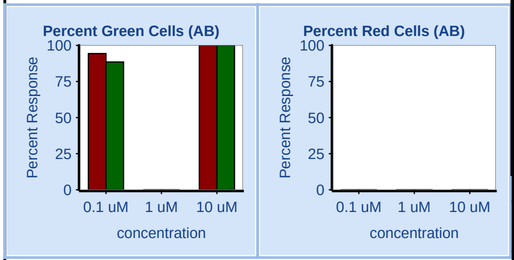

Average Response

Dose	Green Signal	Actual Pct Green Cells	Normalized Pct Green Cells	Red Signal	Actual Pct Red Cells	Normalized Pct Red Cells
0.1 uM	99.65	0.88	91.45	81.04	0.04	-50.38
1 uM	85.52	0.78	-318.52	66.01	0.03	-35.89
10 uM	102.24	0.94	118.76	76.98	0.05	-30.10

Compound	Normalized Values (A and B Sets)	Responses

Tebipenem Pivoxil

Average Response

Dose						

Compound	Normalized Values (A and B Sets)	Responses

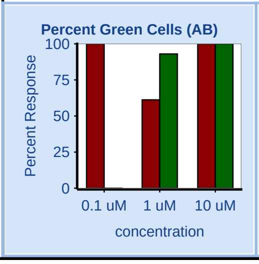

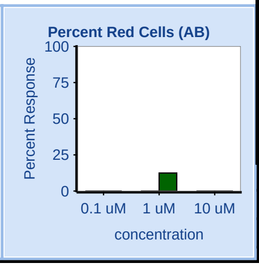

	Green Signal	Actual Pct Green Cells	Normalized Pct Green Cells	Red Signal	Actual Pct Red Cells	Normalized Pct Red Cells
0.1 uM	85.69	0.80	-12.58	81.48	0.03	-38.56
1 uM	98.46	0.85	77.12	96.19	0.09	-14.27
10 uM	103.54	0.94	444.08	83.31	0.03	-42.73

Compound	Normalized Values (A and B Sets)	Responses

Telaprevir (VX-950)

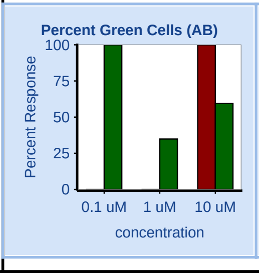

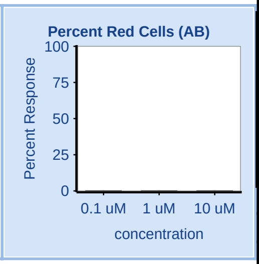

Average Response						
Dose	Green Signal	Actual Pct Green Cells	Normalized Pct Green Cells	Red Signal	Actual Pct Red Cells	Normalized Pct Red Cells
0.1 uM	100.07	0.84	2.88	101.79	0.08	-31.65
1 uM	95.91	0.81	7.25	94.44	0.06	-29.84
10 uM	104.31	0.86	87.69	99.72	0.06	-27.80

Compound	Normalized Values (A and B Sets)	Responses

Telatinib

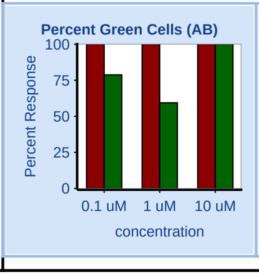

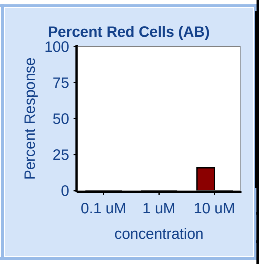

Average Response						
Dose	Green Signal	Actual Pct Green Cells	Normalized Pct Green Cells	Red Signal	Actual Pct Red Cells	Normalized Pct Red Cells
0.1 uM	94.19	0.89	92.96	75.86	0.02	-46.59
1 uM	105.41	0.88	87.44	76.70	0.03	-42.67
10 uM	106.14	0.97	484.81	95.44	0.08	-17.98

Compound	Normalized Values (A and B Sets)	Responses

Telbivudine

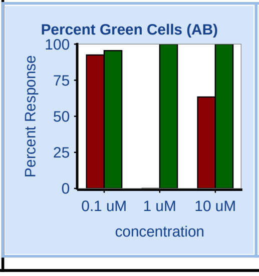

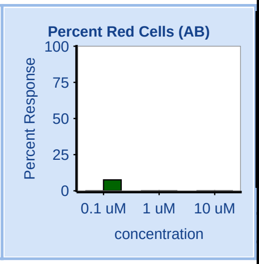

Average Response						
Dose	Green Signal	Actual Pct Green Cells	Normalized Pct Green Cells	Red Signal	Actual Pct Red Cells	Normalized Pct Red Cells
0.1 uM	106.07	0.90	93.98	107.82	0.12	-6.83
1 uM	111.17	0.88	49.11	100.61	0.08	-19.40
10 uM	104.09	0.86	89.11	102.01	0.06	-26.71

Compound	Normalized Values (A and B Sets)	Responses

Temocapril HCl

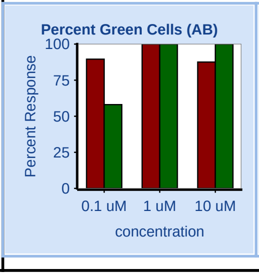

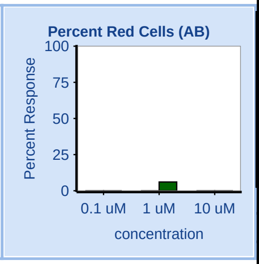

Average Response						
Dose	Green Signal	Actual Pct Green Cells	Normalized Pct Green Cells	Red Signal	Actual Pct Red Cells	Normalized Pct Red Cells
0.1 uM	98.29	0.86	73.81	97.32	0.08	-33.57
1 uM	111.87	0.91	113.18	101.05	0.10	-11.38
10 uM	107.93	0.88	101.61	109.71	0.10	-12.25

Compound	Normalized Values (A and B Sets)	Responses

Tempol

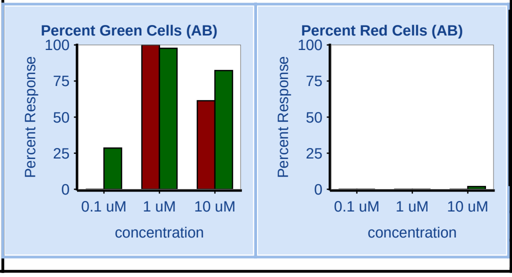

Average Response						
Dose	Green Signal	Actual Pct Green Cells	Normalized Pct Green Cells	Red Signal	Actual Pct Red Cells	Normalized Pct Red Cells
0.1 uM	86.09	0.82	-71.81	77.53	0.03	-49.73
1 uM	99.04	0.89	124.31	75.93	0.04	-46.91
10 uM	95.31	0.85	71.81	98.21	0.09	-17.26

Compound	Normalized Values (A and B Sets)	Responses

Temsirolimus (CCI-779, NSC 683864)

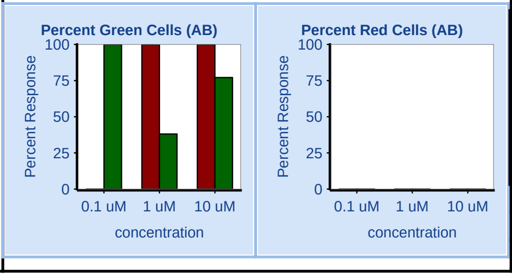

Average Response						
Dose	Green Signal	Actual Pct Green Cells	Normalized Pct Green Cells	Red Signal	Actual Pct Red Cells	Normalized Pct Red Cells
0.1 uM	88.88	0.85	20.13	73.45	0.02	-64.08
1 uM	93.54	0.88	114.22	87.65	0.03	-45.40
10 uM	99.77	0.91	117.64	98.83	0.05	-33.30

Compound	Normalized Values (A and B Sets)	Responses

Tenatoprazole

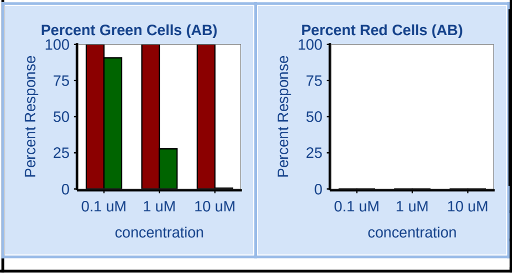

Average Response						
Dose	Green Signal	Actual Pct Green Cells	Normalized Pct Green Cells	Red Signal	Actual Pct Red Cells	Normalized Pct Red Cells
0.1 uM	107.20	0.91	163.19	81.28	0.05	-37.64
1 uM	94.75	0.84	82.67	85.38	0.06	-37.73
10 uM	96.30	0.80	89.85	85.77	0.05	-39.65

Compound	Normalized Values (A and B Sets)	Responses

Teniposide

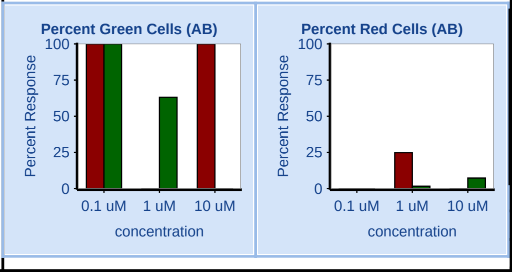

Average Response						
Dose	Green Signal	Actual Pct Green Cells	Normalized Pct Green Cells	Red Signal	Actual Pct Red Cells	Normalized Pct Red Cells
0.1 uM	105.82	0.91	101.88	97.64	0.08	-31.46
1 uM	111.99	0.81	-17.05	119.81	0.15	13.15
10 uM	79.97	0.62	-89.76	101.59	0.08	-14.11

Compound	Normalized Values (A and B Sets)	Responses

Tenofovir

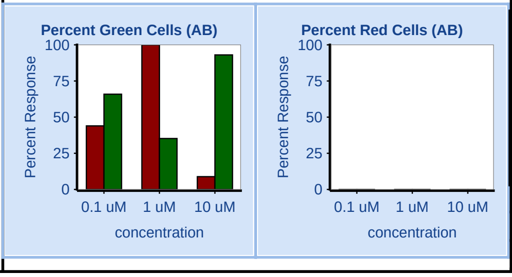

Average Response						
Dose	Green Signal	Actual Pct Green Cells	Normalized Pct Green Cells	Red Signal	Actual Pct Red Cells	Normalized Pct Red Cells
0.1 uM	97.84	0.85	54.91	81.27	0.03	-52.96
1 uM	107.08	0.87	107.73	89.70	0.05	-36.26
10 uM	94.18	0.80	50.94	104.58	0.07	-19.79

Compound	Normalized Values (A and B Sets)	Responses

Tenofovir Disoproxil Fumarate

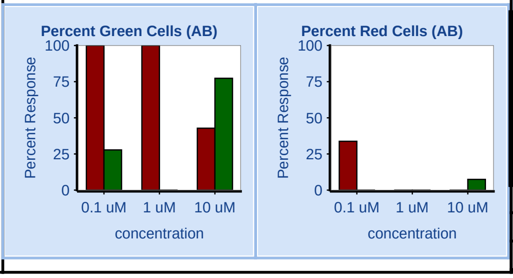

Average Response						
Dose	Green Signal	Actual Pct Green Cells	Normalized Pct Green Cells	Red Signal	Actual Pct Red Cells	Normalized Pct Red Cells
0.1 uM	97.83	0.84	69.29	109.98	0.13	-10.46
1 uM	87.85	0.75	10.75	80.17	0.03	-45.50
10 uM	97.06	0.81	60.09	118.06	0.12	-1.81

Compound	Normalized Values (A and B Sets)	Responses

Tenovin-1

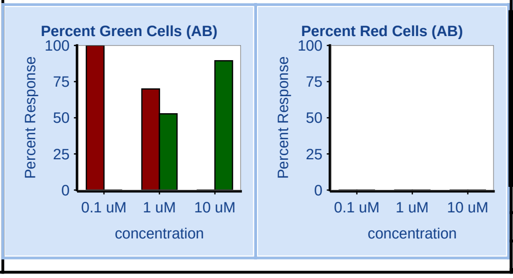

Average Response						
Dose	Green Signal	Actual Pct Green Cells	Normalized Pct Green Cells	Red Signal	Actual Pct Red Cells	Normalized Pct Red Cells
0.1 uM	98.28	0.86	82.83	76.74	0.03	-59.12
1 uM	92.63	0.87	61.32	73.64	0.03	-28.68
10 uM	82.08	0.75	-64.53	71.19	0.04	-34.13

Compound	Normalized Values (A and B Sets)	Responses

Terazosin HCl

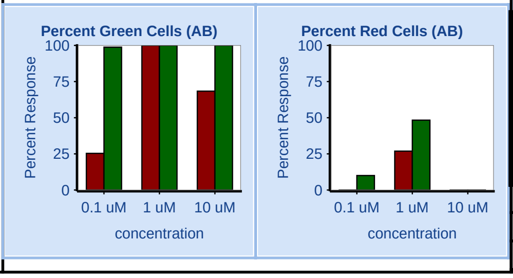

Average Response						
Dose	Green Signal	Actual Pct Green Cells	Normalized Pct Green Cells	Red Signal	Actual Pct Red Cells	Normalized Pct Red Cells
0.1 uM	100.21	0.88	62.09	113.02	0.14	4.64
1 uM	118.26	0.91	115.64	127.62	0.21	37.61
10 uM	102.06	0.87	96.20	94.52	0.06	-26.14

Compound	Normalized Values (A and B Sets)	Responses

Terbinafine

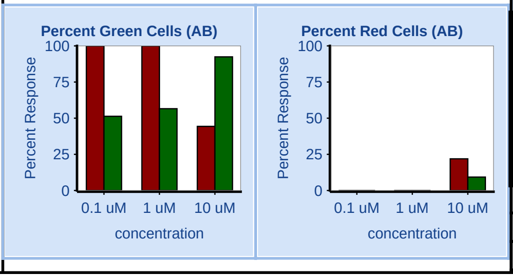

Average Response						
Dose	Green Signal	Actual Pct Green Cells	Normalized Pct Green Cells	Red Signal	Actual Pct Red Cells	Normalized Pct Red Cells
0.1 uM	97.59	0.88	96.58	81.12	0.02	-61.22
1 uM	103.18	0.88	109.48	101.40	0.08	-21.35
10 uM	99.65	0.82	68.38	131.56	0.16	15.58

Compound	Normalized Values (A and B Sets)	Responses

Teriflunomide

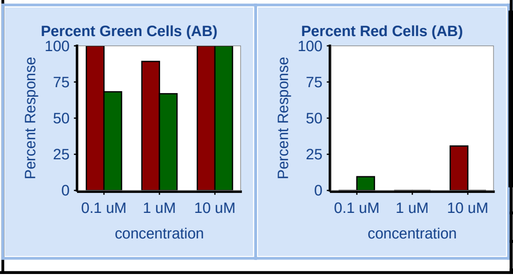

Average Response						
Dose	Green Signal	Actual Pct Green Cells	Normalized Pct Green Cells	Red Signal	Actual Pct Red Cells	Normalized Pct Red Cells
0.1 uM	95.41	0.89	170.14	105.88	0.13	4.46
1 uM	99.79	0.83	78.05	96.01	0.10	-15.74
10 uM	105.87	0.91	124.10	103.53	0.12	2.86

Compound | Normalized Values (A and B Sets) | Responses

Tetrahydropapaverine HCl

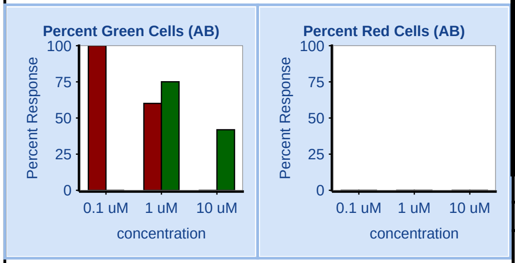

Average Response						
Dose	Green Signal	Actual Pct Green Cells	Normalized Pct Green Cells	Red Signal	Actual Pct Red Cells	Normalized Pct Red Cells
0.1 uM	95.97	0.85	47.05	80.07	0.03	-40.01
1 uM	92.81	0.83	67.58	71.29	0.01	-51.70
10 uM	87.53	0.81	-490.62	73.35	0.02	-51.07

Compound | Normalized Values (A and B Sets) | Responses

TG100-115

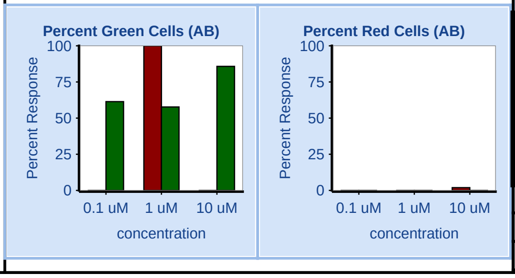

Average Response						
Dose	Green Signal	Actual Pct Green Cells	Normalized Pct Green Cells	Red Signal	Actual Pct Red Cells	Normalized Pct Red Cells
0.1 uM	91.46	0.80	-10.81	95.43	0.06	-46.02
1 uM	102.19	0.88	103.76	73.55	0.02	-49.79
10 uM	86.95	0.77	35.59	106.01	0.07	-26.77

Compound | Normalized Values (A and B Sets) | Responses

TG100713

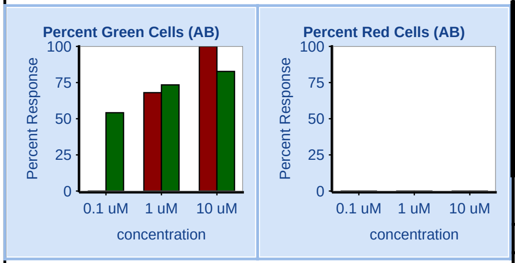

Average Response						
Dose	Green Signal	Actual Pct Green Cells	Normalized Pct Green Cells	Red Signal	Actual Pct Red Cells	Normalized Pct Red Cells
0.1 uM	81.57	0.80	-222.68	69.45	0.01	-56.12
1 uM	89.36	0.82	70.73	72.26	0.03	-51.90
10 uM	90.02	0.87	108.64	87.03	0.05	-38.15

Compound | Normalized Values (A and B Sets) | Responses

TG101209

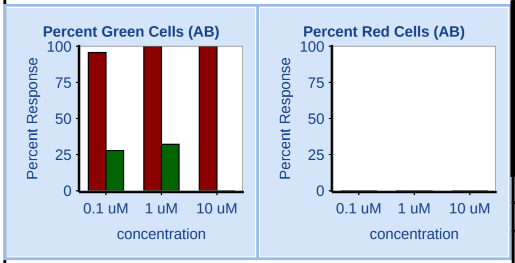

Average Response						
Dose	Green Signal	Actual Pct Green Cells	Normalized Pct Green Cells	Red Signal	Actual Pct Red Cells	Normalized Pct Red Cells
0.1 uM	91.43	0.86	61.73	90.97	0.06	-25.41
1 uM	100.80	0.88	83.69	76.25	0.03	-44.47
10 uM	77.94	0.68	-156.96	89.18	0.05	-33.31

Compound | Normalized Values (A and B Sets) | Responses

TG101348 (SAR302503)

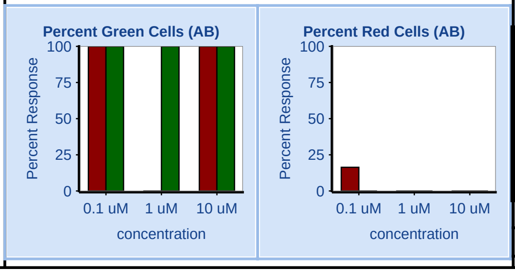

Average Response						
Dose	Green Signal	Actual Pct Green Cells	Normalized Pct Green Cells	Red Signal	Actual Pct Red Cells	Normalized Pct Red Cells
0.1 uM	108.69	0.94	148.06	98.34	0.11	-5.08
1 uM	91.47	0.77	45.00	88.89	0.07	-26.24

Compound	Normalized Values (A and B Sets)	Responses						
		10 uM	106.32	0.96	467.05	87.84	0.04	-40.04

Compound	Normalized Values (A and B Sets)	Responses
**Thiamet G**	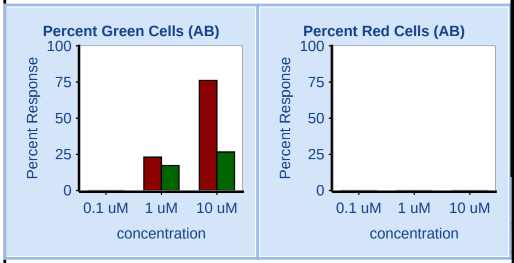	Average Response (see table below)

Average Response

Dose	Green Signal	Actual Pct Green Cells	Normalized Pct Green Cells	Red Signal	Actual Pct Red Cells	Normalized Pct Red Cells
0.1 uM	74.36	0.65	-116.92	83.05	0.03	-52.91
1 uM	93.84	0.85	20.18	82.81	0.05	-20.94
10 uM	91.27	0.87	51.35	69.32	0.03	-37.54

Compound	Normalized Values (A and B Sets)	Responses
**Thiazovivin**	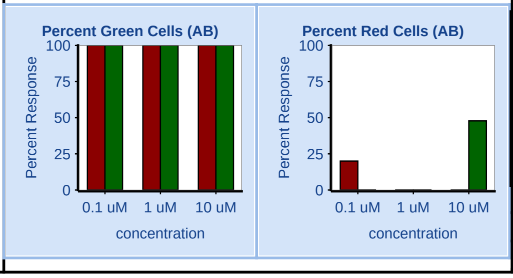	Average Response (see table below)

Average Response

Dose	Green Signal	Actual Pct Green Cells	Normalized Pct Green Cells	Red Signal	Actual Pct Red Cells	Normalized Pct Red Cells
0.1 uM	108.21	0.94	143.59	97.86	0.09	-17.28
1 uM	112.48	0.95	179.38	76.11	0.04	-30.96
10 uM	122.76	0.97	145.55	102.86	0.17	21.14

Compound	Normalized Values (A and B Sets)	Responses
**Thiostrepton**	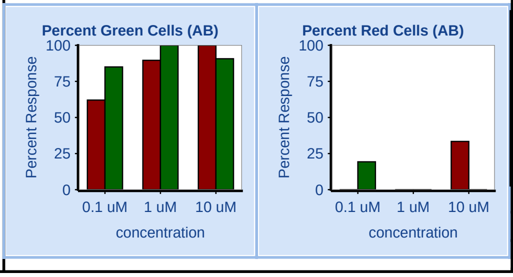	Average Response (see table below)

Average Response

Dose	Green Signal	Actual Pct Green Cells	Normalized Pct Green Cells	Red Signal	Actual Pct Red Cells	Normalized Pct Red Cells
0.1 uM	99.42	0.88	73.54	92.48	0.10	-9.88
1 uM	102.44	0.88	114.18	82.12	0.05	-44.46
10 uM	117.45	0.87	96.15	103.89	0.13	9.70

Compound	Normalized Values (A and B Sets)	Responses
**Tianeptine sodium**	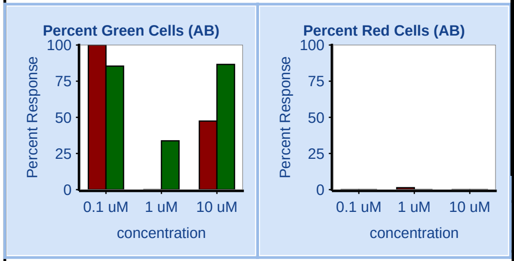	Average Response (see table below)

Average Response

Dose	Green Signal	Actual Pct Green Cells	Normalized Pct Green Cells	Red Signal	Actual Pct Red Cells	Normalized Pct Red Cells
0.1 uM	104.94	0.90	104.13	89.08	0.05	-44.49
1 uM	80.76	0.64	-256.57	102.93	0.09	-17.76
10 uM	97.20	0.82	67.00	99.36	0.06	-28.80

Compound	Normalized Values (A and B Sets)	Responses
**Ticagrelor**		Average Response (see table below)

Average Response

Dose	Green Signal	Actual Pct Green Cells		Red Signal	Actual Pct Red Cells	Normalized Pct Red Cells

Compound	Normalized Values (A and B Sets)	Responses

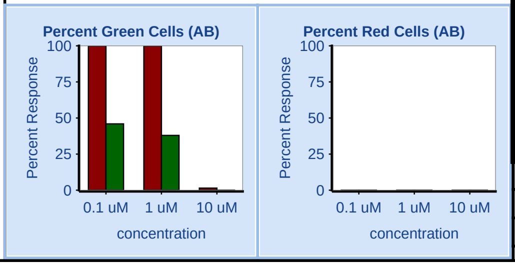

			Normalized Pct Green Cells			
0.1 uM	93.47	0.87	111.90	100.10	0.11	-9.48
1 uM	94.82	0.85	88.30	78.62	0.02	-56.03
10 uM	76.97	0.61	-59.52	98.75	0.07	-26.55

Compound	Normalized Values (A and B Sets)	Responses

Ticarcillin sodium

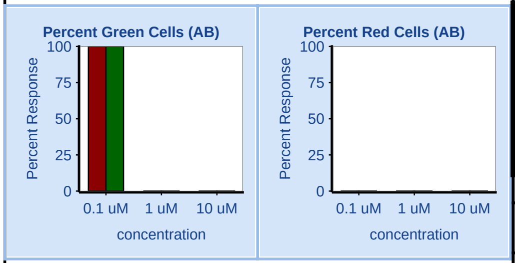

Average Response						
Dose	Green Signal	Actual Pct Green Cells	Normalized Pct Green Cells	Red Signal	Actual Pct Red Cells	Normalized Pct Red Cells
0.1 uM	96.71	0.92	191.94	85.56	0.07	-28.71
1 uM	66.14	0.46	-198.38	73.23	0.02	-58.16
10 uM	79.24	0.71	-52.35	71.15	0.01	-57.11

Compound	Normalized Values (A and B Sets)	Responses

Tideglusib

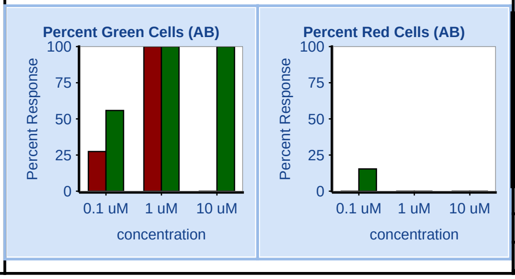

Average Response						
Dose	Green Signal	Actual Pct Green Cells	Normalized Pct Green Cells	Red Signal	Actual Pct Red Cells	Normalized Pct Red Cells
0.1 uM	97.44	0.84	41.63	97.56	0.11	-1.43
1 uM	107.92	0.91	109.54	84.53	0.04	-36.47
10 uM	101.56	0.91	28.30	91.77	0.05	-35.00

Compound	Normalized Values (A and B Sets)	Responses

Tie2 kinase inhibitor

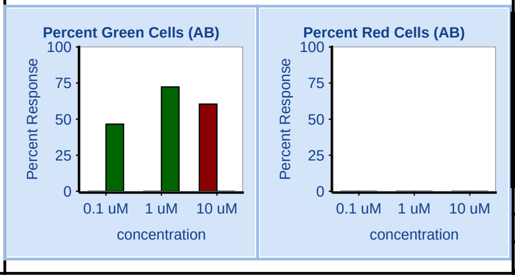

Average Response						
Dose	Green Signal	Actual Pct Green Cells	Normalized Pct Green Cells	Red Signal	Actual Pct Red Cells	Normalized Pct Red Cells
0.1 uM	89.96	0.79	-16.89	99.10	0.08	-27.21
1 uM	93.71	0.80	-40.46	92.01	0.04	-39.77
10 uM	84.69	0.77	22.49	98.58	0.05	-31.81

Compound	Normalized Values (A and B Sets)	Responses

Tigecycline

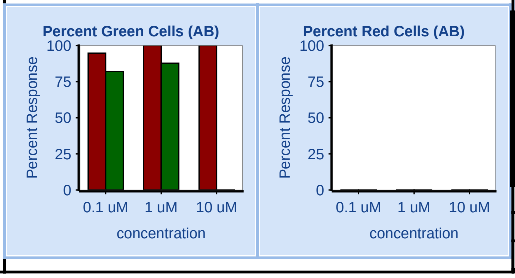

Average Response						
Dose	Green Signal	Actual Pct Green Cells	Normalized Pct Green Cells	Red Signal	Actual Pct Red Cells	Normalized Pct Red Cells
0.1 uM	99.45	0.89	88.44	78.63	0.03	-59.69
1 uM	105.05	0.89	103.52	100.44	0.06	-28.40
10 uM	95.05	0.79	32.75	99.00	0.07	-26.20

Compound	Normalized Values (A and B Sets)	Responses

Tilmicosin

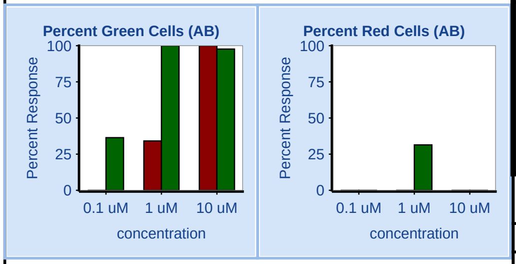

Average Response

Dose	Green Signal	Actual Pct Green Cells	Normalized Pct Green Cells	Red Signal	Actual Pct Red Cells	Normalized Pct Red Cells
0.1 uM	82.94	0.75	-361.83	78.35	0.04	-44.02
1 uM	98.73	0.81	67.43	99.00	0.12	-5.79
10 uM	103.54	0.91	144.66	77.11	0.03	-48.81

Compound	Normalized Values (A and B Sets)	Responses

Tiopronin

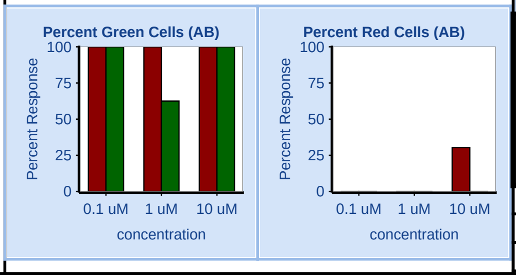

Average Response

Dose	Green Signal	Actual Pct Green Cells	Normalized Pct Green Cells	Red Signal	Actual Pct Red Cells	Normalized Pct Red Cells
0.1 uM	108.55	0.92	121.97	90.81	0.06	-39.83
1 uM	104.53	0.87	90.89	101.48	0.08	-19.61
10 uM	114.52	0.91	117.33	117.96	0.14	1.78

Compound	Normalized Values (A and B Sets)	Responses

Tiotropium Bromide hydrate

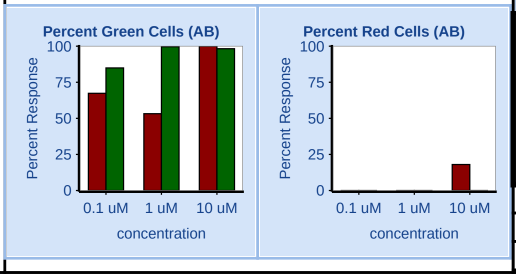

Average Response

Dose	Green Signal	Actual Pct Green Cells	Normalized Pct Green Cells	Red Signal	Actual Pct Red Cells	Normalized Pct Red Cells
0.1 uM	96.47	0.87	76.14	76.98	0.04	-33.36
1 uM	95.82	0.84	76.41	94.43	0.06	-27.49
10 uM	101.91	0.90	113.59	95.82	0.09	-15.43

Compound	Normalized Values (A and B Sets)	Responses

Tioxolone

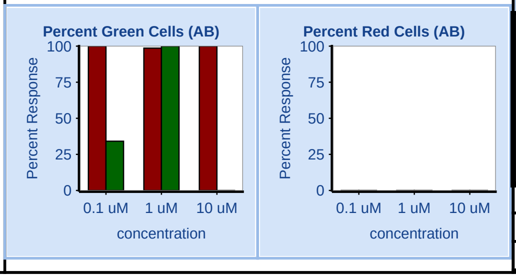

Average Response

Dose	Green Signal	Actual Pct Green Cells	Normalized Pct Green Cells	Red Signal	Actual Pct Red Cells	Normalized Pct Red Cells
0.1 uM	103.25	0.90	104.19	80.42	0.04	-35.39
1 uM	112.82	0.90	104.81	93.87	0.07	-22.51
10 uM	95.04	0.89	244.30	85.74	0.04	-37.31

Compound	Normalized Values (A and B Sets)	Responses

Tipifarnib

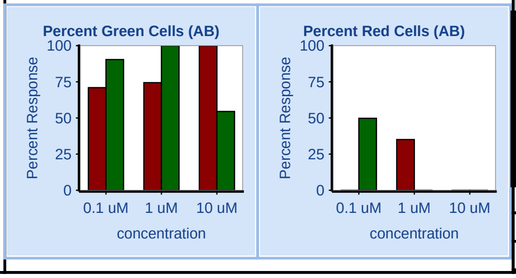

Average Response

Dose	Green Signal	Actual Pct Green Cells	Normalized Pct Green Cells	Red Signal	Actual Pct Red Cells	Normalized Pct Red Cells
0.1 uM	98.97	0.89	80.68	108.62	0.13	4.90
1 uM	103.72	0.90	91.42	103.86	0.11	-7.75
10 uM	99.42	0.85	80.16	97.50	0.05	-33.73

Compound | Normalized Values (A and B Sets) | Responses

Tivantinib (ARQ 197)

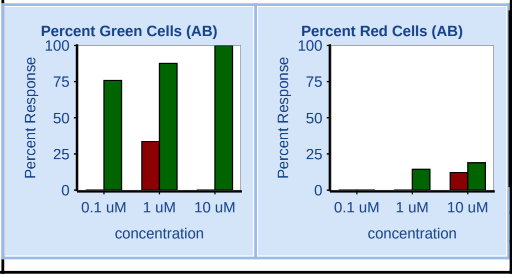

Average Response

Dose	Green Signal	Actual Pct Green Cells	Normalized Pct Green Cells	Red Signal	Actual Pct Red Cells	Normalized Pct Red Cells
0.1 uM	89.16	0.83	36.80	75.37	0.02	-44.01
1 uM	105.42	0.81	60.59	94.71	0.12	-1.82
10 uM	117.25	0.92	80.24	105.65	0.15	15.54

Compound | Normalized Values (A and B Sets) | Responses

Tivozanib (AV-951)

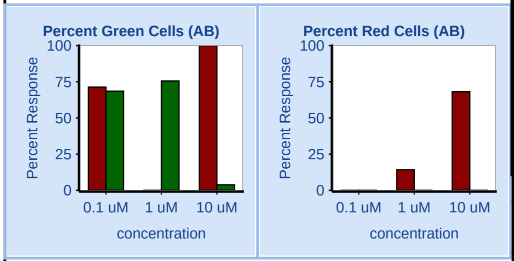

Average Response

Dose	Green Signal	Actual Pct Green Cells	Normalized Pct Green Cells	Red Signal	Actual Pct Red Cells	Normalized Pct Red Cells
0.1 uM	92.86	0.86	69.97	73.62	0.02	-64.70
1 uM	87.37	0.81	-401.72	88.70	0.08	-20.33
10 uM	100.20	0.90	90.59	106.15	0.16	22.84

Compound | Normalized Values (A and B Sets) | Responses

Tofacitinib (CP-690550) Citrate

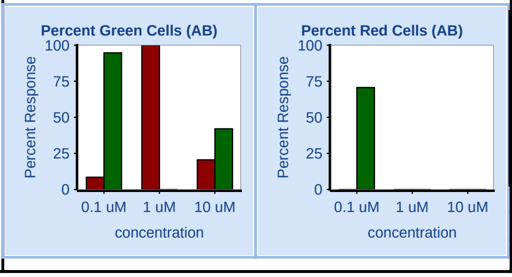

Average Response

Dose	Green Signal	Actual Pct Green Cells	Normalized Pct Green Cells	Red Signal	Actual Pct Red Cells	Normalized Pct Red Cells
0.1 uM	98.62	0.88	51.62	104.82	0.16	16.61
1 uM	96.68	0.81	64.40	78.60	0.05	-43.16
10 uM	95.32	0.79	31.32	95.15	0.09	-14.06

Compound | Normalized Values (A and B Sets) | Responses

Tofacitinib (CP-690550,Tasocitinib)

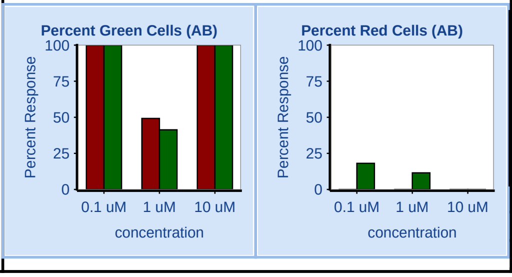

Average Response

Dose	Green Signal	Actual Pct Green Cells	Normalized Pct Green Cells	Red Signal	Actual Pct Red Cells	Normalized Pct Red Cells
0.1 uM	101.44	0.92	133.08	95.75	0.10	-6.01
1 uM	94.11	0.79	45.28	95.96	0.08	-18.07
10 uM	114.12	0.94	434.49	88.76	0.05	-33.66

Compound | Normalized Values (A and B Sets) | Responses

Tolcapone

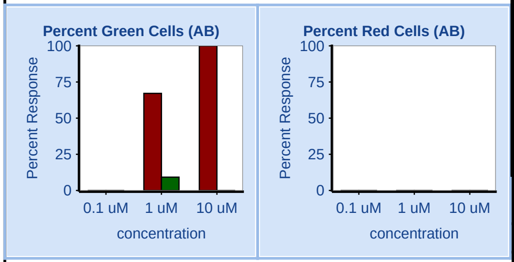

Average Response

Dose	Green Signal	Actual Pct Green Cells	Normalized Pct Green Cells	Red Signal	Actual Pct Red Cells	Normalized Pct Red Cells
0.1 uM	79.25	0.74	-203.85	74.56	0.02	-52.76
1 uM	92.97	0.77	38.17	82.57	0.05	-43.86
10 uM	82.05	0.69	45.97	86.13	0.04	-43.05

Compound	Normalized Values (A and B Sets)	Responses

Tolmetin Sodium

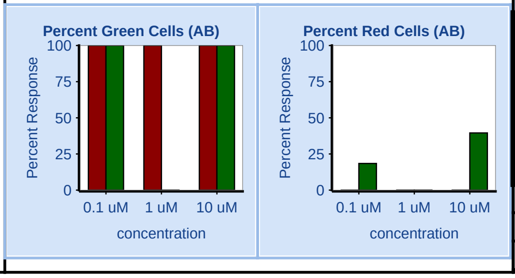

Average Response

Dose	Green Signal	Actual Pct Green Cells	Normalized Pct Green Cells	Red Signal	Actual Pct Red Cells	Normalized Pct Red Cells
0.1 uM	109.31	0.95	176.76	95.65	0.12	-1.98
1 uM	92.31	0.82	66.13	93.44	0.10	-18.99
10 uM	111.39	0.94	165.82	102.81	0.14	7.72

Compound	Normalized Values (A and B Sets)	Responses

Tolterodine tartrate

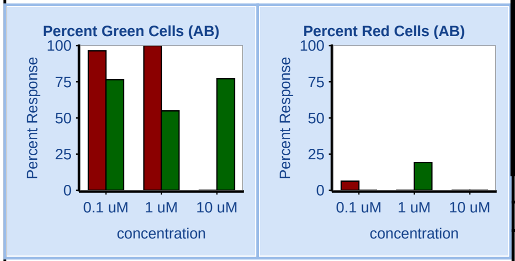

Average Response

Dose	Green Signal	Actual Pct Green Cells	Normalized Pct Green Cells	Red Signal	Actual Pct Red Cells	Normalized Pct Red Cells
0.1 uM	95.13	0.88	86.44	87.26	0.07	-21.85
1 uM	109.28	0.88	85.31	97.29	0.10	-9.96
10 uM	92.82	0.86	-159.02	76.73	0.03	-46.04

Compound	Normalized Values (A and B Sets)	Responses

Tolvaptan

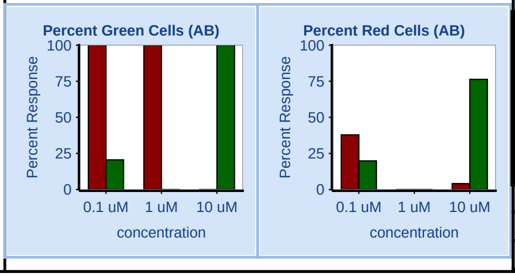

Average Response

Dose	Green Signal	Actual Pct Green Cells	Normalized Pct Green Cells	Red Signal	Actual Pct Red Cells	Normalized Pct Red Cells
0.1 uM	99.93	0.89	91.99	113.58	0.18	28.86
1 uM	100.11	0.84	59.05	93.12	0.09	-14.72
10 uM	107.97	0.92	53.30	116.73	0.21	40.21

Compound	Normalized Values (A and B Sets)	Responses

Torcetrapib

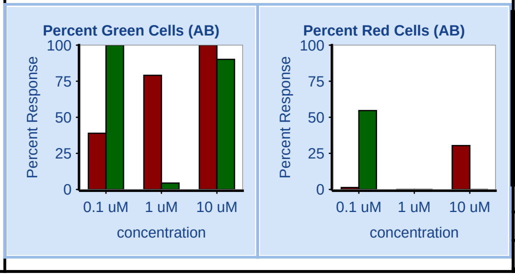

Average Response

Dose	Green Signal	Actual Pct Green Cells	Normalized Pct Green Cells	Red Signal	Actual Pct Red Cells	Normalized Pct Red Cells
0.1 uM	108.71	0.90	110.19	106.60	0.17	28.01
1 uM	95.52	0.79	41.76	77.88	0.04	-39.97
10 uM	108.26	0.92	324.32	99.31	0.11	-5.76

Compound	Normalized Values (A and B Sets)	Responses

Torin 1

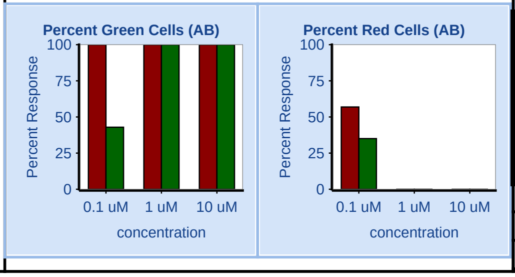

Average Response

Dose	Green Signal	Actual Pct Green Cells	Normalized Pct Green Cells	Red Signal	Actual Pct Red Cells	Normalized Pct Red Cells
0.1 uM	98.93	0.91	113.12	123.12	0.22	45.97
1 uM	111.00	0.95	123.91	92.21	0.08	-20.51
10 uM	107.82	0.94	400.41	106.41	0.10	-11.02

Compound	Normalized Values (A and B Sets)	Responses

Torin 2

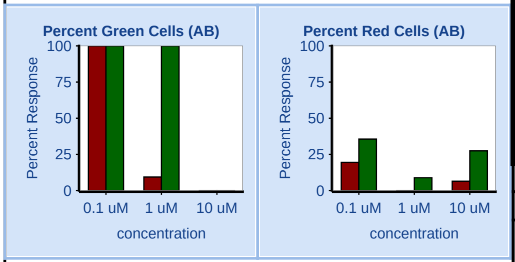

Average Response						
Dose	Green Signal	Actual Pct Green Cells	Normalized Pct Green Cells	Red Signal	Actual Pct Red Cells	Normalized Pct Red Cells
0.1 uM	106.77	0.95	154.97	111.56	0.17	27.50
1 uM	106.13	0.82	70.25	90.62	0.12	-2.53
10 uM	66.69	0.50	-1558.68	97.89	0.16	16.89

Compound	Normalized Values (A and B Sets)	Responses

Tosedostat (CHR2797)

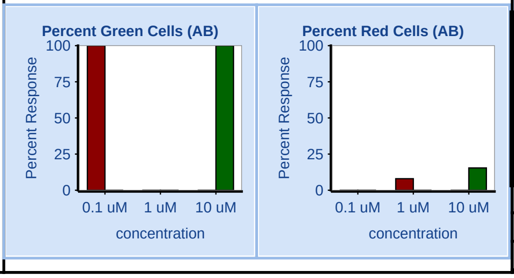

Average Response						
Dose	Green Signal	Actual Pct Green Cells	Normalized Pct Green Cells	Red Signal	Actual Pct Red Cells	Normalized Pct Red Cells
0.1 uM	87.43	0.72	-3.27	98.90	0.09	-29.28
1 uM	82.78	0.64	-94.28	95.46	0.09	-15.66
10 uM	91.08	0.65	-32.37	91.43	0.10	-6.53

Compound	Normalized Values (A and B Sets)	Responses

Trametinib (GSK1120212)

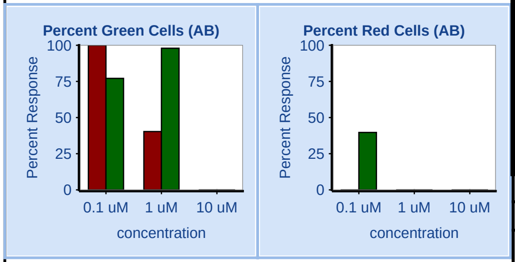

Average Response						
Dose	Green Signal	Actual Pct Green Cells	Normalized Pct Green Cells	Red Signal	Actual Pct Red Cells	Normalized Pct Red Cells
0.1 uM	94.77	0.90	101.14	102.65	0.14	12.31
1 uM	99.99	0.83	69.16	92.83	0.09	-16.01
10 uM	96.66	0.84	-117.17	72.94	0.04	-40.17

Compound	Normalized Values (A and B Sets)	Responses

Triamcinolone Acetonide

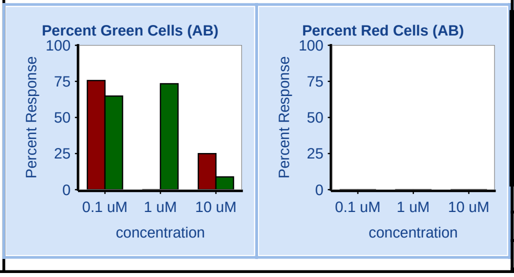

Average Response						
Dose	Green Signal	Actual Pct Green Cells	Normalized Pct Green Cells	Red Signal	Actual Pct Red Cells	Normalized Pct Red Cells
0.1 uM	94.42	0.86	70.22	90.77	0.06	-44.45
1 uM	93.54	0.83	14.24	89.31	0.05	-33.11
10 uM	91.36	0.75	16.88	95.37	0.06	-31.31

Compound	Normalized Values (A and B Sets)	Responses

Trichostatin A (TSA)

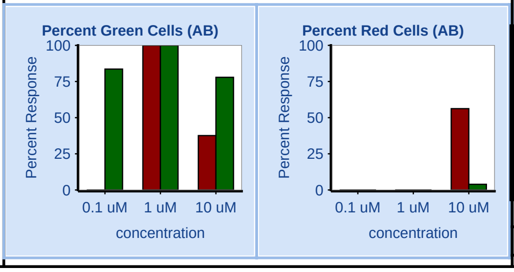

Average Response						
Dose	Green Signal	Actual Pct Green Cells	Normalized Pct Green Cells	Red Signal	Actual Pct Red Cells	Normalized Pct Red Cells
0.1 uM	90.47	0.85	34.31	77.97	0.03	-54.36
1 uM	120.06	0.95	175.60	107.00	0.07	-24.04

Compound	Normalized Values (A and B Sets)	Responses						
		10 uM	101.74	0.81	57.77	143.41	0.21	30.08

Compound	Normalized Values (A and B Sets)	Responses
**Triclabendazole**	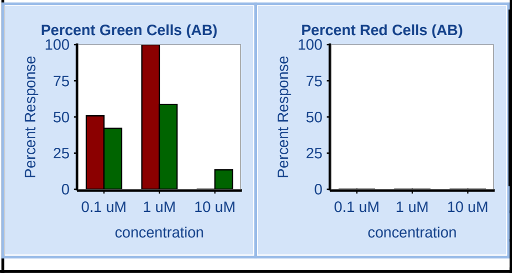	Average Response

Dose	Green Signal	Actual Pct Green Cells	Normalized Pct Green Cells	Red Signal	Actual Pct Red Cells	Normalized Pct Red Cells
0.1 uM	91.94	0.85	46.49	69.90	0.02	-53.40
1 uM	97.28	0.84	83.64	74.16	0.03	-54.02
10 uM	81.38	0.70	-75.19	68.50	0.01	-59.29

Compound	Normalized Values (A and B Sets)	Responses
**Trilostane**	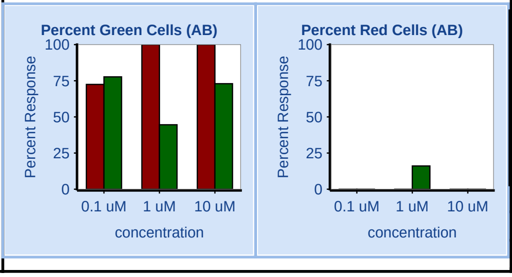	Average Response

Dose	Green Signal	Actual Pct Green Cells	Normalized Pct Green Cells	Red Signal	Actual Pct Red Cells	Normalized Pct Red Cells
0.1 uM	100.32	0.87	75.08	89.70	0.06	-41.89
1 uM	104.38	0.86	90.79	105.04	0.10	-10.10
10 uM	100.81	0.87	95.43	106.33	0.07	-26.42

Compound	Normalized Values (A and B Sets)	Responses
**Trimebutine**	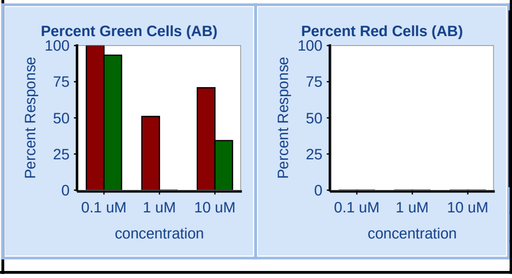	Average Response

Dose	Green Signal	Actual Pct Green Cells	Normalized Pct Green Cells	Red Signal	Actual Pct Red Cells	Normalized Pct Red Cells
0.1 uM	107.78	0.92	122.00	98.98	0.08	-32.36
1 uM	98.28	0.80	24.82	94.56	0.06	-29.88
10 uM	95.43	0.81	52.55	102.24	0.07	-27.56

Compound	Normalized Values (A and B Sets)	Responses
**Trometamol**	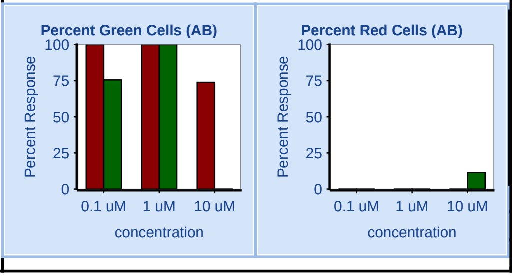	Average Response

Dose	Green Signal	Actual Pct Green Cells	Normalized Pct Green Cells	Red Signal	Actual Pct Red Cells	Normalized Pct Red Cells
0.1 uM	101.80	0.89	101.06	85.18	0.04	-52.19
1 uM	102.51	0.92	171.38	76.70	0.04	-27.22
10 uM	80.65	0.71	-88.87	84.50	0.09	-14.23

Compound	Normalized Values (A and B Sets)	Responses
**Trospium chloride**		Average Response

Dose	Green Signal	Actual Pct Green Cells	Normalized Pct Green Cells	Red Signal	Actual Pct Red Cells	Normalized Pct Red Cells

Compound	Normalized Values (A and B Sets)	Responses
	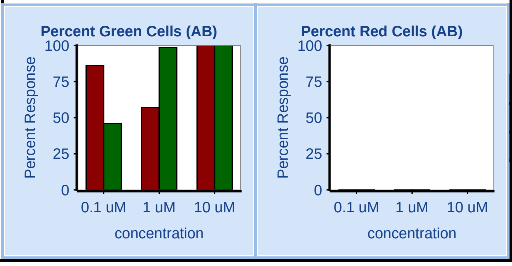	see table below

0.1 uM	89.71	0.86	66.13	82.28	0.03	-39.02
1 uM	101.66	0.85	77.94	91.65	0.08	-20.67
10 uM	99.23	0.92	138.97	80.09	0.03	-42.47

Compound	Normalized Values (A and B Sets)	Responses
**Troxerutin**	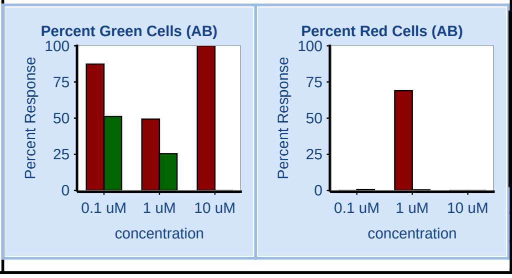	Average Response (see table below)

Average Response						
Dose	Green Signal	Actual Pct Green Cells	Normalized Pct Green Cells	Red Signal	Actual Pct Red Cells	Normalized Pct Red Cells
0.1 uM	96.32	0.87	69.30	84.77	0.07	-18.29
1 uM	90.07	0.78	37.31	125.44	0.20	34.60
10 uM	96.45	0.88	236.82	74.80	0.01	-51.91

Compound	Normalized Values (A and B Sets)	Responses
**TSU-68 (SU6668, Orantinib)**	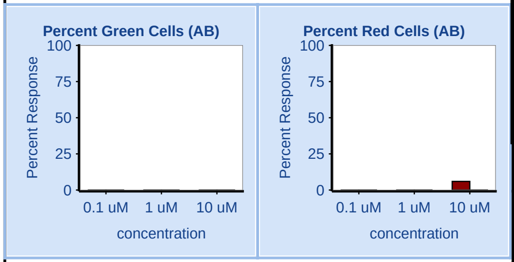	Average Response (see table below)

Average Response						
Dose	Green Signal	Actual Pct Green Cells	Normalized Pct Green Cells	Red Signal	Actual Pct Red Cells	Normalized Pct Red Cells
0.1 uM	0.85	0.00	-695.34	89.88	0.05	-43.43
1 uM	0.89	0.01	-3282.57	78.08	0.04	-29.05
10 uM	0.00	0.00	-768.78	91.13	0.09	-12.02

Compound	Normalized Values (A and B Sets)	Responses
**TTNPB (Arotinoid Acid)**	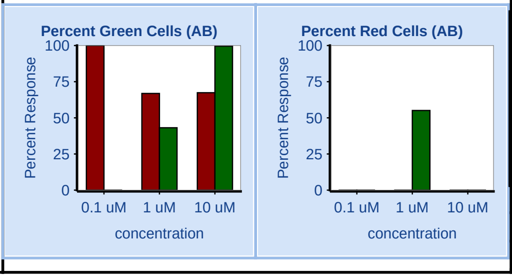	Average Response (see table below)

Average Response						
Dose	Green Signal	Actual Pct Green Cells	Normalized Pct Green Cells	Red Signal	Actual Pct Red Cells	Normalized Pct Red Cells
0.1 uM	86.62	0.72	78.41	71.52	0.03	-49.26
1 uM	97.03	0.80	55.01	110.80	0.16	15.55
10 uM	108.12	0.87	83.49	73.72	0.04	-44.54

Compound	Normalized Values (A and B Sets)	Responses
**TTP 22**	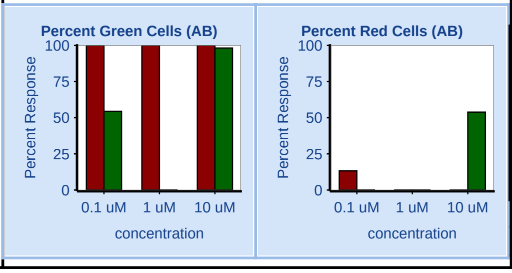	Average Response (see table below)

Average Response						
Dose	Green Signal	Actual Pct Green Cells	Normalized Pct Green Cells	Red Signal	Actual Pct Red Cells	Normalized Pct Red Cells
0.1 uM	99.60	0.89	99.77	104.26	0.09	-19.16
1 uM	93.71	0.85	191.50	78.11	0.03	-35.73

Compound	Normalized Values (A and B Sets)	Responses

10 uM	105.03	0.94	123.80	87.54	0.12	1.71

Compound	Normalized Values (A and B Sets)	Responses

Tubastatin A

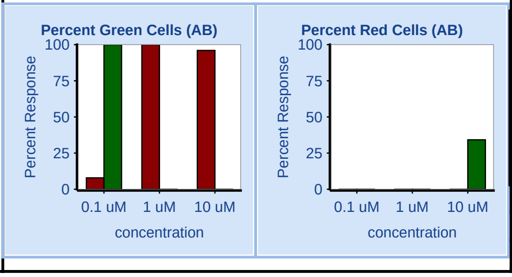

Average Response

Dose	Green Signal	Actual Pct Green Cells	Normalized Pct Green Cells	Red Signal	Actual Pct Red Cells	Normalized Pct Red Cells
0.1 uM	92.83	0.85	60.38	82.75	0.03	-58.53
1 uM	92.19	0.85	118.66	64.43	0.01	-44.49
10 uM	91.92	0.86	46.36	101.28	0.14	9.63

Compound	Normalized Values (A and B Sets)	Responses

Tubastatin A HCl

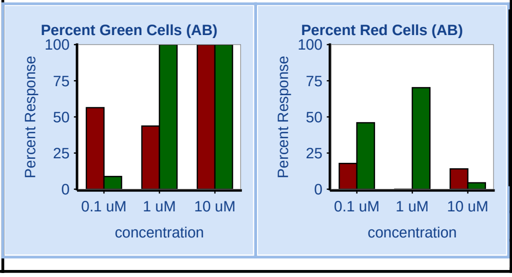

Average Response

Dose	Green Signal	Actual Pct Green Cells	Normalized Pct Green Cells	Red Signal	Actual Pct Red Cells	Normalized Pct Red Cells
0.1 uM	88.50	0.83	32.60	116.63	0.18	31.89
1 uM	106.95	0.86	92.10	106.42	0.17	21.29
10 uM	105.11	0.95	426.44	107.72	0.14	9.25

Compound	Normalized Values (A and B Sets)	Responses

TW-37

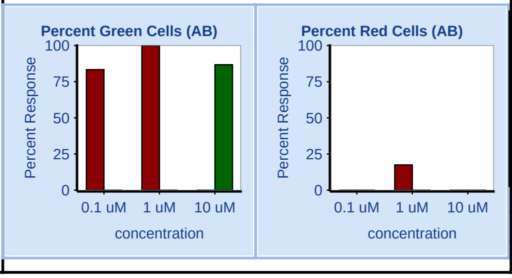

Average Response

Dose	Green Signal	Actual Pct Green Cells	Normalized Pct Green Cells	Red Signal	Actual Pct Red Cells	Normalized Pct Red Cells
0.1 uM	85.70	0.80	38.99	75.17	0.02	-63.45
1 uM	77.56	0.57	-98.07	110.07	0.12	-2.87
10 uM	95.63	0.72	5.23	92.70	0.08	-17.19

Compound	Normalized Values (A and B Sets)	Responses

Tylosin tartrate

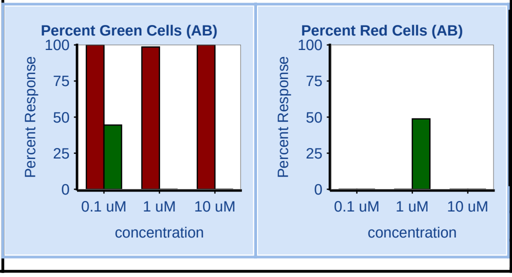

Average Response

Dose	Green Signal	Actual Pct Green Cells	Normalized Pct Green Cells	Red Signal	Actual Pct Red Cells	Normalized Pct Red Cells
0.1 uM	91.73	0.87	135.43	91.36	0.07	-29.81
1 uM	83.91	0.76	26.43	107.82	0.15	6.06
10 uM	85.89	0.78	76.61	82.92	0.04	-44.06

Compound	Normalized Values (A and B Sets)	Responses

Tyrphostin AG 879

Average Response

Dose	Green Signal	Actual Pct Green Cells	Normalized Pct Green Cells	Red Signal	Actual Pct Red Cells	Normalized Pct Red Cells

Compound	Normalized Values (A and B Sets)	Responses

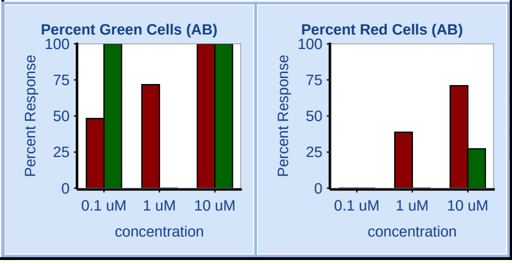

0.1 uM	101.55	0.91	121.19	93.70	0.10	-8.17
1 uM	89.66	0.76	24.76	104.94	0.15	9.81
10 uM	118.83	0.97	497.61	126.60	0.22	49.14

Compound	Normalized Values (A and B Sets)	Responses

U 73122

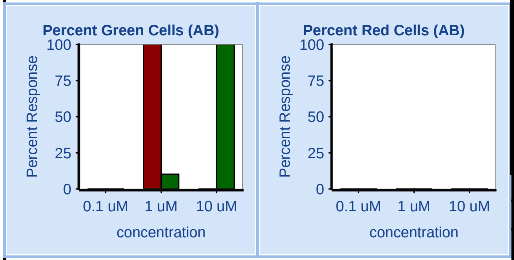

Average Response						
Dose	Green Signal	Actual Pct Green Cells	Normalized Pct Green Cells	Red Signal	Actual Pct Red Cells	Normalized Pct Red Cells
0.1 uM	78.67	0.66	-99.39	76.16	0.02	-62.10
1 uM	97.32	0.87	181.44	77.76	0.04	-29.48
10 uM	80.37	0.65	-171.28	62.87	0.02	-41.27

Compound	Normalized Values (A and B Sets)	Responses

U-104

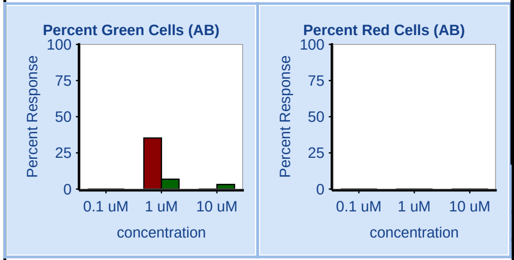

Average Response						
Dose	Green Signal	Actual Pct Green Cells	Normalized Pct Green Cells	Red Signal	Actual Pct Red Cells	Normalized Pct Red Cells
0.1 uM	81.92	0.75	-110.11	71.02	0.02	-53.55
1 uM	86.14	0.75	21.04	82.85	0.05	-40.74
10 uM	76.98	0.62	-195.22	67.49	0.01	-58.65

Compound	Normalized Values (A and B Sets)	Responses

U0126-EtOH

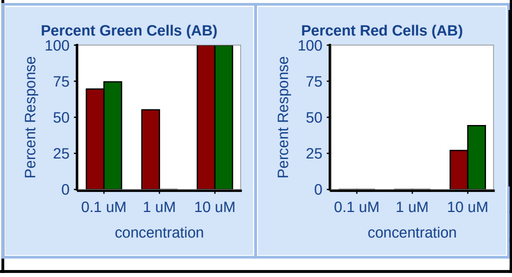

Average Response						
Dose	Green Signal	Actual Pct Green Cells	Normalized Pct Green Cells	Red Signal	Actual Pct Red Cells	Normalized Pct Red Cells
0.1 uM	92.45	0.87	72.08	89.47	0.04	-47.39
1 uM	88.74	0.78	12.70	87.67	0.03	-44.74
10 uM	110.41	0.94	145.32	147.81	0.21	35.62

Compound	Normalized Values (A and B Sets)	Responses

UK 383367

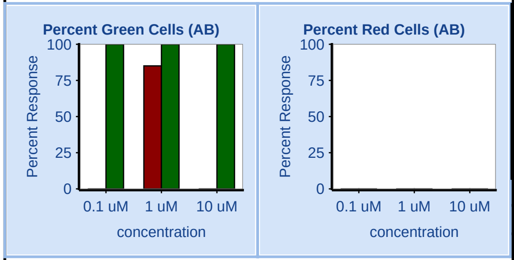

Average Response						
Dose	Green Signal	Actual Pct Green Cells	Normalized Pct Green Cells	Red Signal	Actual Pct Red Cells	Normalized Pct Red Cells
0.1 uM	90.99	0.82	31.90	83.83	0.05	-31.10
1 uM	103.72	0.91	114.61	71.77	0.01	-49.66
10 uM	86.62	0.77		89.46	0.05	-35.85

Compound	Normalized Values (A and B Sets)	Responses

			-1011.07			

Compound	Normalized Values (A and B Sets)	Responses

Ulipristal

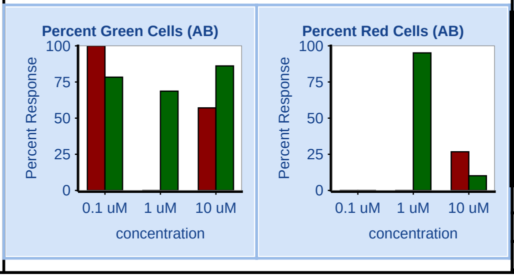

Average Response

Dose	Green Signal	Actual Pct Green Cells	Normalized Pct Green Cells	Red Signal	Actual Pct Red Cells	Normalized Pct Red Cells
0.1 uM	99.61	0.89	97.96	88.77	0.06	-40.68
1 uM	101.63	0.88	33.29	106.22	0.17	43.86
10 uM	103.43	0.89	71.60	100.09	0.16	18.43

Compound	Normalized Values (A and B Sets)	Responses

UNC 0224

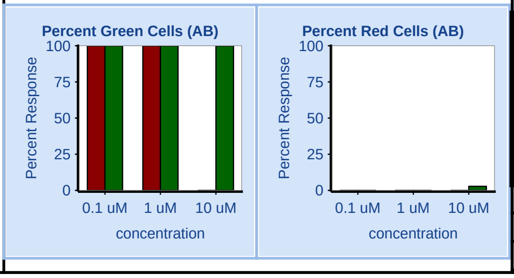

Average Response

Dose	Green Signal	Actual Pct Green Cells	Normalized Pct Green Cells	Red Signal	Actual Pct Red Cells	Normalized Pct Red Cells
0.1 uM	109.99	0.92	128.67	75.84	0.02	-63.78
1 uM	110.55	0.96	255.93	67.90	0.02	-39.35
10 uM	101.27	0.80	-23.41	85.54	0.11	-2.37

Compound	Normalized Values (A and B Sets)	Responses

UNC669

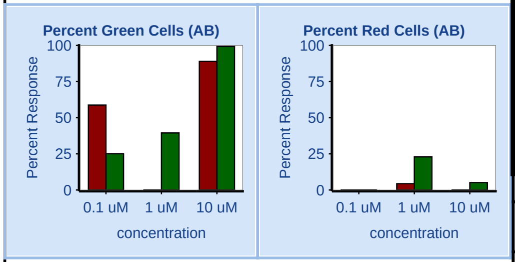

Average Response

Dose	Green Signal	Actual Pct Green Cells	Normalized Pct Green Cells	Red Signal	Actual Pct Red Cells	Normalized Pct Red Cells
0.1 uM	91.30	0.82	41.92	60.88	0.01	-71.55
1 uM	86.50	0.76	-639.71	100.18	0.13	13.64
10 uM	99.84	0.91	94.11	91.40	0.09	-13.40

Compound	Normalized Values (A and B Sets)	Responses

UNC1215

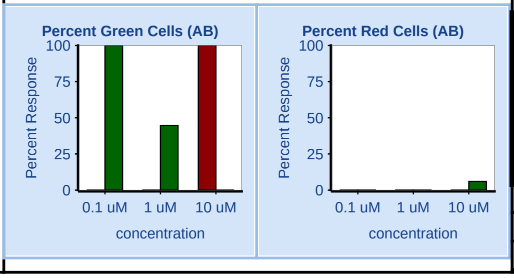

Average Response

Dose	Green Signal	Actual Pct Green Cells	Normalized Pct Green Cells	Red Signal	Actual Pct Red Cells	Normalized Pct Red Cells
0.1 uM	87.60	0.77	-14.75	89.55	0.07	-30.79
1 uM	89.33	0.85	-88.44	74.91	0.04	-27.14
10 uM	93.25	0.82	17.85	87.95	0.10	-7.26

Compound	Normalized Values (A and B Sets)	Responses

UNC1999

Average Response

Dose	Green Signal			Red Signal	Actual Pct Red Cells	

Compound	Normalized Values (A and B Sets)	Responses

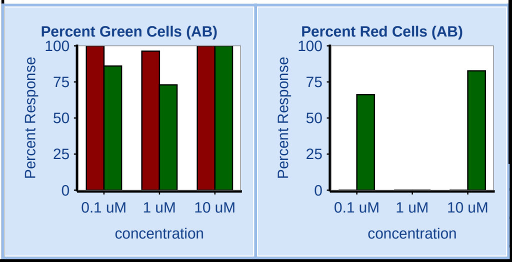

		Actual Pct Green Cells	Normalized Pct Green Cells			Normalized Pct Red Cells
0.1 uM	101.33	0.91	204.82	99.67	0.13	5.52
1 uM	98.17	0.84	84.65	98.08	0.10	-15.88
10 uM	109.49	0.94	150.74	105.96	0.14	10.58

Compound	Normalized Values (A and B Sets)	Responses

UPF 1069

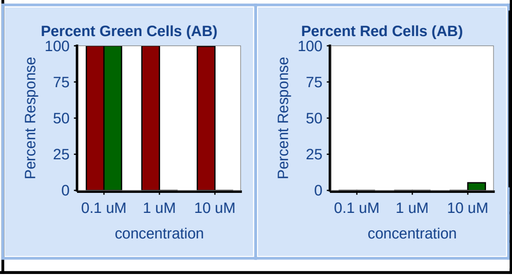

Average Response

Dose	Green Signal	Actual Pct Green Cells	Normalized Pct Green Cells	Red Signal	Actual Pct Red Cells	Normalized Pct Red Cells
0.1 uM	111.49	0.95	152.71	73.32	0.03	-58.64
1 uM	103.26	0.86	216.30	66.07	0.02	-38.92
10 uM	89.85	0.85	37.25	73.65	0.06	-23.91

Compound	Normalized Values (A and B Sets)	Responses

URB597

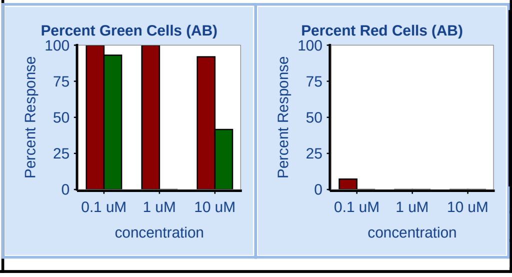

Average Response

Dose	Green Signal	Actual Pct Green Cells	Normalized Pct Green Cells	Red Signal	Actual Pct Red Cells	Normalized Pct Red Cells
0.1 uM	102.47	0.92	119.63	86.84	0.08	-21.20
1 uM	98.81	0.82	50.98	80.77	0.04	-37.84
10 uM	89.11	0.88	66.77	81.24	0.03	-42.94

Compound	Normalized Values (A and B Sets)	Responses

Uridine

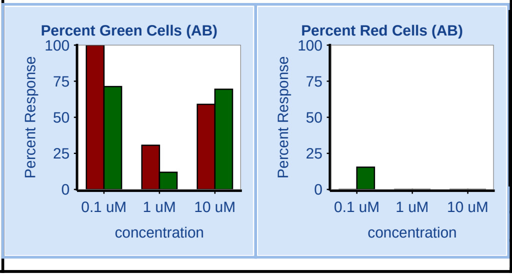

Average Response

Dose	Green Signal	Actual Pct Green Cells	Normalized Pct Green Cells	Red Signal	Actual Pct Red Cells	Normalized Pct Red Cells
0.1 uM	107.08	0.91	120.23	103.92	0.11	-9.38
1 uM	93.66	0.81	21.26	85.52	0.06	-31.54
10 uM	95.99	0.82	64.20	89.78	0.03	-39.35

Compound	Normalized Values (A and B Sets)	Responses

Ursolic Acid

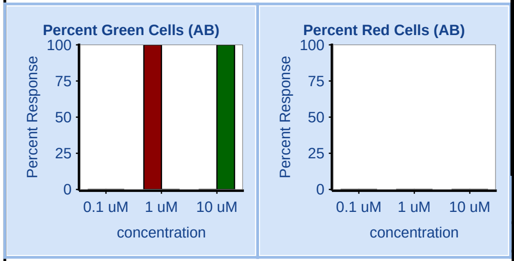

Average Response

Dose	Green Signal	Actual Pct Green Cells	Normalized Pct Green Cells	Red Signal	Actual Pct Red Cells	Normalized Pct Red Cells
0.1 uM	74.05	0.68	-129.24	80.04	0.03	-39.38
1 uM	96.40	0.80	43.02	93.88	0.07	-26.06
10 uM	93.69	0.88	-49.24	82.66	0.03	-43.55

Compound	Normalized Values (A and B Sets)	Responses

Valganciclovir HCl

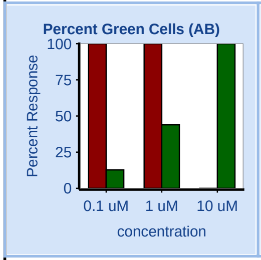

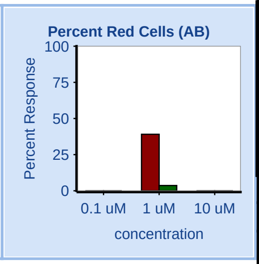

Average Response

Dose	Green Signal	Actual Pct Green Cells	Normalized Pct Green Cells	Red Signal	Actual Pct Red Cells	Normalized Pct Red Cells
0.1 uM	90.34	0.87	194.49	81.41	0.05	-36.60
1 uM	93.37	0.83	77.32	115.41	0.17	21.38
10 uM	96.20	0.76	-131.96	95.00	0.08	-22.10

Compound	Normalized Values (A and B Sets)	Responses

Valnemulin HCl

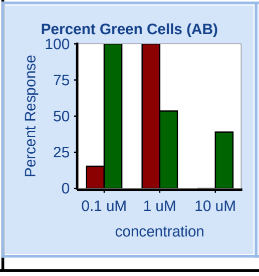

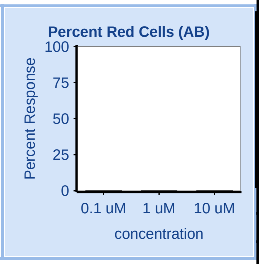

Average Response

Dose	Green Signal	Actual Pct Green Cells	Normalized Pct Green Cells	Red Signal	Actual Pct Red Cells	Normalized Pct Red Cells
0.1 uM	98.27	0.89	60.30	82.89	0.06	-32.73
1 uM	98.84	0.85	95.51	86.87	0.05	-44.56
10 uM	86.67	0.77	8.72	84.36	0.07	-29.48

Compound	Normalized Values (A and B Sets)	Responses

Vanillin

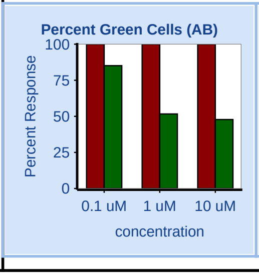

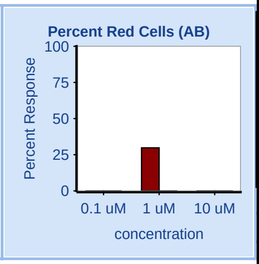

Average Response

Dose	Green Signal	Actual Pct Green Cells	Normalized Pct Green Cells	Red Signal	Actual Pct Red Cells	Normalized Pct Red Cells
0.1 uM	96.36	0.91	211.42	93.03	0.06	-30.64
1 uM	98.60	0.83	78.05	105.54	0.12	-5.00
10 uM	99.75	0.83	86.26	87.47	0.04	-39.26

Compound	Normalized Values (A and B Sets)	Responses

Vanillylacetone

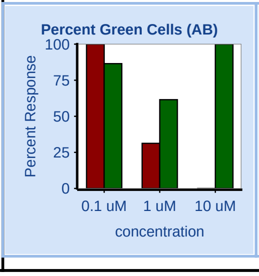

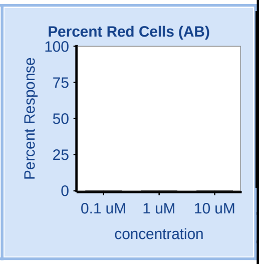

Average Response

Dose	Green Signal	Actual Pct Green Cells	Normalized Pct Green Cells	Red Signal	Actual Pct Red Cells	Normalized Pct Red Cells
0.1 uM	100.75	0.90	106.44	77.90	0.03	-40.13
1 uM	92.79	0.79	46.39	69.21	0.02	-46.55
10 uM	96.16	0.89	-20.39	72.20	0.02	-51.38

Compound	Normalized Values (A and B Sets)	Responses

Vardenafil HCl Trihydrate

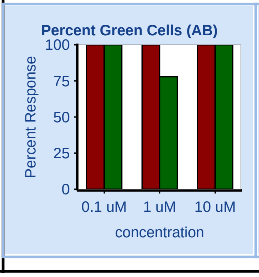

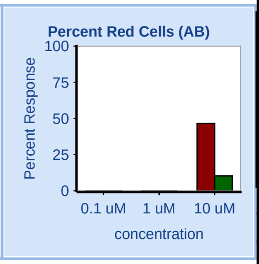

Average Response

Dose	Green Signal	Actual Pct Green Cells	Normalized Pct Green Cells	Red Signal	Actual Pct Red Cells	Normalized Pct Red Cells
0.1 uM	110.32	0.95	156.37	87.84	0.08	-20.23
1 uM	104.18	0.88	91.48	73.37	0.03	-42.54
10 uM	117.60	0.97	512.84	118.47	0.18	28.47

Compound | Normalized Values (A and B Sets) | Responses

Varenicline Tartrate

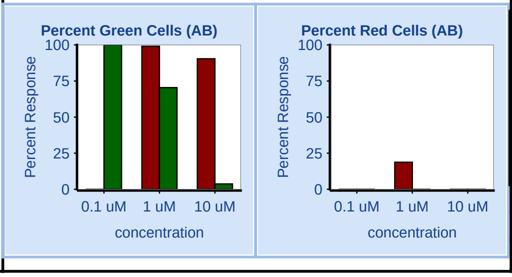

Average Response

Dose	Green Signal	Actual Pct Green Cells	Normalized Pct Green Cells	Red Signal	Actual Pct Red Cells	Normalized Pct Red Cells
0.1 uM	109.15	0.89	63.12	89.71	0.06	-42.71
1 uM	102.88	0.87	84.76	102.67	0.10	-12.13
10 uM	92.86	0.80	47.08	106.57	0.09	-14.79

Compound | Normalized Values (A and B Sets) | Responses

Varespladib (LY315920)

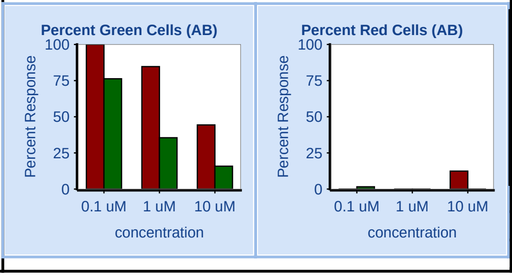

Average Response

Dose	Green Signal	Actual Pct Green Cells	Normalized Pct Green Cells	Red Signal	Actual Pct Red Cells	Normalized Pct Red Cells
0.1 uM	104.17	0.90	109.93	100.27	0.11	-13.92
1 uM	98.69	0.84	60.11	105.66	0.12	-4.16
10 uM	88.76	0.77	30.09	115.67	0.12	-5.71

Compound | Normalized Values (A and B Sets) | Responses

Varlitinib

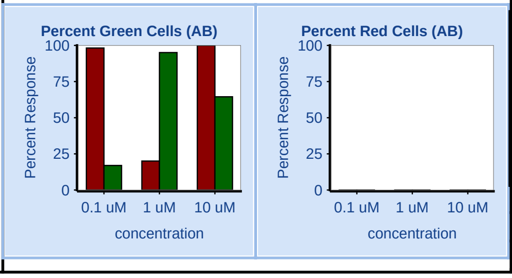

Average Response

Dose	Green Signal	Actual Pct Green Cells	Normalized Pct Green Cells	Red Signal	Actual Pct Red Cells	Normalized Pct Red Cells
0.1 uM	95.93	0.86	57.60	90.95	0.09	-12.00
1 uM	95.91	0.80	57.55	86.10	0.04	-38.13
10 uM	102.24	0.92	303.76	74.21	0.02	-49.34

Compound | Normalized Values (A and B Sets) | Responses

Vatalanib (PTK787) 2HCl

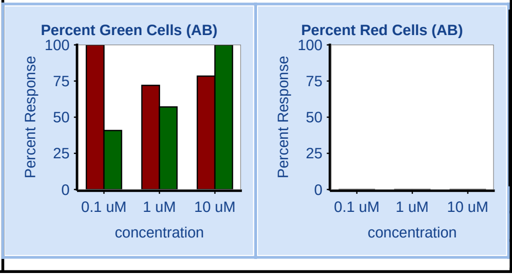

Average Response

Dose	Green Signal	Actual Pct Green Cells	Normalized Pct Green Cells	Red Signal	Actual Pct Red Cells	Normalized Pct Red Cells
0.1 uM	100.62	0.89	115.19	80.89	0.03	-59.85
1 uM	98.03	0.85	64.55	76.92	0.02	-46.97
10 uM	98.77	0.88	102.96	81.96	0.02	-46.25

Compound | Normalized Values (A and B Sets) | Responses

VE-821

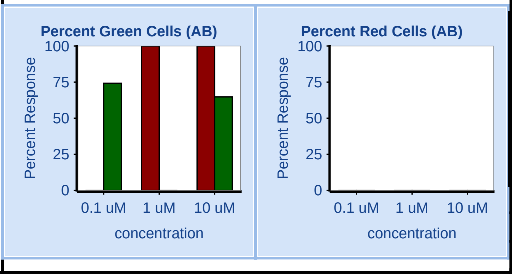

Average Response

Dose	Green Signal	Actual Pct Green Cells	Normalized Pct Green Cells	Red Signal	Actual Pct Red Cells	Normalized Pct Red Cells
0.1 uM	89.19	0.81	30.64	81.68	0.03	-54.22
1 uM	98.93	0.84	172.01	78.21	0.05	-18.23
10 uM	102.18	0.91	90.94	80.32	0.06	-25.45

Compound | Normalized Values (A and B Sets) | Responses

Verteporfin

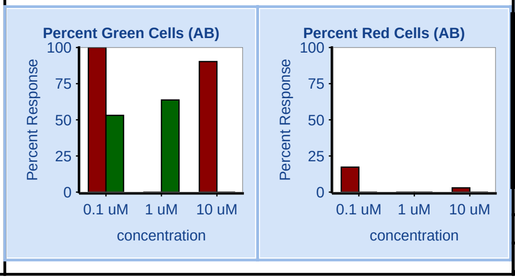

Average Response

Dose	Green Signal	Actual Pct Green Cells	Normalized Pct Green Cells	Red Signal	Actual Pct Red Cells	Normalized Pct Red Cells
0.1 uM	107.18	0.89	108.62	101.43	0.10	-24.48
1 uM	100.86	0.83	14.80	99.06	0.08	-19.81
10 uM	93.47	0.77	19.23	104.69	0.08	-22.80

Compound | Normalized Values (A and B Sets) | Responses

Vilazodone HCl

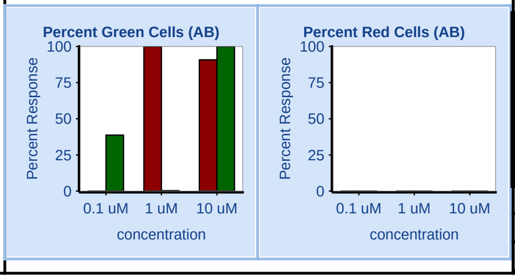

Average Response

Dose	Green Signal	Actual Pct Green Cells	Normalized Pct Green Cells	Red Signal	Actual Pct Red Cells	Normalized Pct Red Cells
0.1 uM	89.04	0.83	-52.85	79.36	0.05	-37.09
1 uM	100.01	0.85	89.37	81.90	0.06	-39.45
10 uM	114.59	0.92	115.28	90.36	0.06	-32.17

Compound | Normalized Values (A and B Sets) | Responses

Vildagliptin (LAF-237)

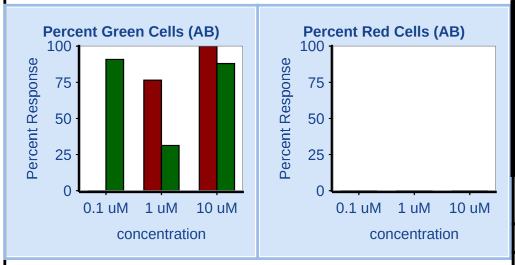

Average Response

Dose	Green Signal	Actual Pct Green Cells	Normalized Pct Green Cells	Red Signal	Actual Pct Red Cells	Normalized Pct Red Cells
0.1 uM	90.22	0.81	-244.25	79.65	0.04	-41.97
1 uM	93.80	0.80	53.92	79.03	0.03	-49.63
10 uM	102.21	0.88	114.35	91.34	0.05	-36.77

Compound | Normalized Values (A and B Sets) | Responses

Vinblastine

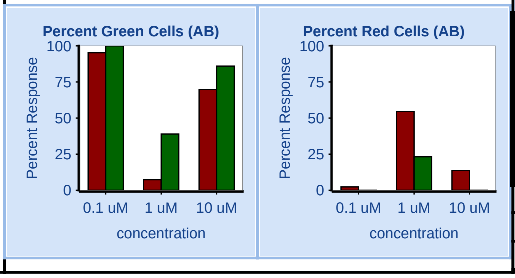

Average Response

Dose	Green Signal	Actual Pct Green Cells	Normalized Pct Green Cells	Red Signal	Actual Pct Red Cells	Normalized Pct Red Cells
0.1 uM	124.48	0.93	114.21	97.03	0.09	-28.85
1 uM	111.64	0.82	23.10	121.35	0.21	38.84
10 uM	112.89	0.84	77.93	101.48	0.10	-13.34

Compound | Normalized Values (A and B Sets) | Responses

Vinflunine Tartrate

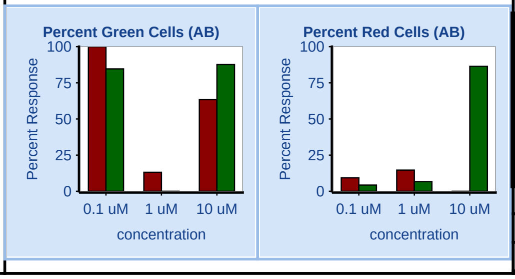

Average Response

Dose	Green Signal	Actual Pct Green Cells	Normalized Pct Green Cells	Red Signal	Actual Pct Red Cells	Normalized Pct Red Cells
0.1 uM	108.62	0.91	111.82	99.59	0.13	6.78
1 uM	92.68	0.69	-7.43	104.48	0.15	10.67
10 uM	113.88	0.89	75.49	115.46	0.19	33.31

Compound	Normalized Values (A and B Sets)	Responses

Vinorelbine

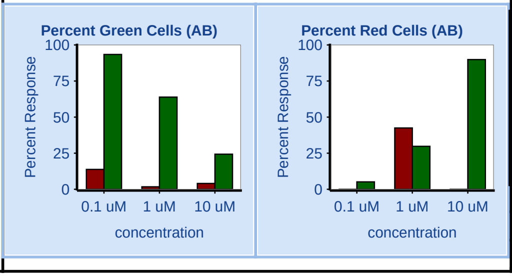

Average Response

Dose	Green Signal	Actual Pct Green Cells	Normalized Pct Green Cells	Red Signal	Actual Pct Red Cells	Normalized Pct Red Cells
0.1 uM	115.96	0.87	53.59	108.52	0.14	2.11
1 uM	123.92	0.84	32.80	120.47	0.20	36.12
10 uM	110.73	0.74	14.18	122.19	0.21	41.16

Compound	Normalized Values (A and B Sets)	Responses

Vitamin B12

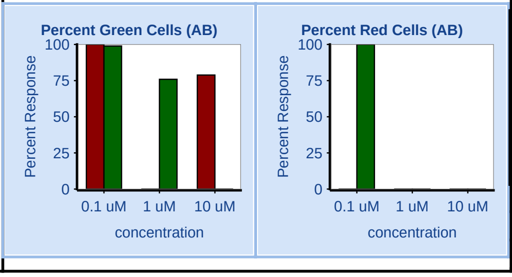

Average Response

Dose	Green Signal	Actual Pct Green Cells	Normalized Pct Green Cells	Red Signal	Actual Pct Red Cells	Normalized Pct Red Cells
0.1 uM	105.52	0.92	123.92	114.70	0.17	26.92
1 uM	101.01	0.84	32.91	85.35	0.04	-41.38
10 uM	86.34	0.70	-32.37	102.51	0.06	-28.33

Compound	Normalized Values (A and B Sets)	Responses

Vitamin D2

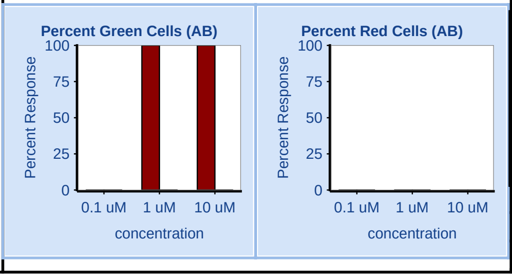

Average Response

Dose	Green Signal	Actual Pct Green Cells	Normalized Pct Green Cells	Red Signal	Actual Pct Red Cells	Normalized Pct Red Cells
0.1 uM	83.65	0.79	-60.94	59.31	0.01	-58.33
1 uM	87.20	0.76	21.15	76.62	0.04	-50.03
10 uM	89.27	0.77	64.11	68.74	0.02	-55.78

Compound	Normalized Values (A and B Sets)	Responses

Vitamin D3

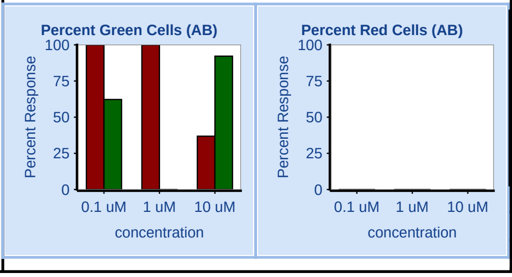

Average Response

Dose	Green Signal	Actual Pct Green Cells	Normalized Pct Green Cells	Red Signal	Actual Pct Red Cells	Normalized Pct Red Cells
0.1 uM	98.26	0.89	179.29	60.90	0.02	-55.06
1 uM	88.65	0.79	46.33	80.77	0.03	-52.03
10 uM	97.52	0.85	64.54	83.15	0.04	-40.57

Compound	Normalized Values (A and B Sets)	Responses

Voglibose

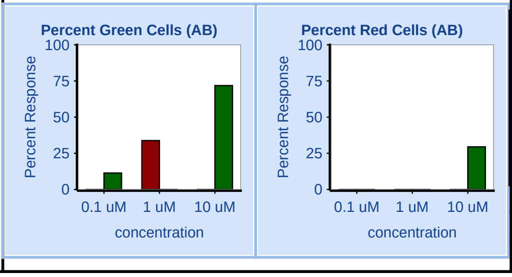

Average Response

Dose	Green Signal	Actual Pct Green Cells	Normalized Pct Green Cells	Red Signal	Actual Pct Red Cells	Normalized Pct Red Cells
0.1 uM	85.20	0.81	-40.00	71.14	0.02	-51.53
1 uM	88.61	0.73	9.23	81.05	0.04	-46.68
10 uM	92.65	0.80	9.39	94.50	0.10	-13.74

Compound	Normalized Values (A and B Sets)	Responses

Volasertib (BI 6727)

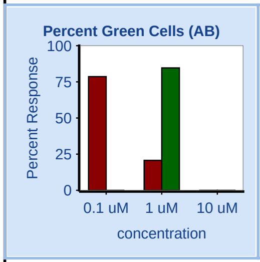

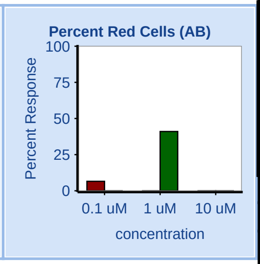

Average Response

Dose	Green Signal	Actual Pct Green Cells	Normalized Pct Green Cells	Red Signal	Actual Pct Red Cells	Normalized Pct Red Cells
0.1 uM	105.22	0.82	24.17	95.97	0.12	0.07
1 uM	104.05	0.79	52.68	108.69	0.13	1.37
10 uM	101.72	0.81	-103.81	96.00	0.09	-15.00

Compound	Normalized Values (A and B Sets)	Responses

Vorinostat (SAHA, MK0683)

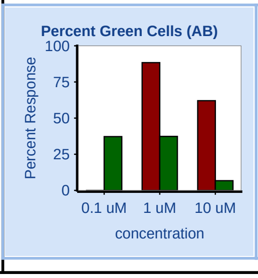

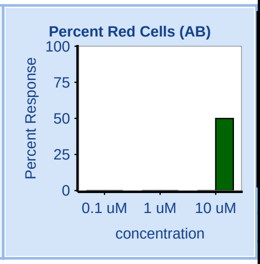

Average Response

Dose	Green Signal	Actual Pct Green Cells	Normalized Pct Green Cells	Red Signal	Actual Pct Red Cells	Normalized Pct Red Cells
0.1 uM	91.03	0.77	-5.19	74.77	0.03	-55.61
1 uM	92.30	0.86	62.86	55.82	0.02	-40.27
10 uM	98.53	0.85	34.38	101.94	0.15	12.94

Compound	Normalized Values (A and B Sets)	Responses

Vortioxetine (Lu AA21004) HBr

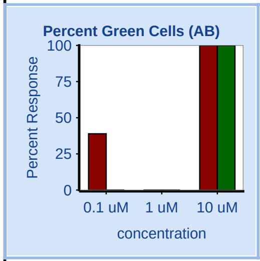

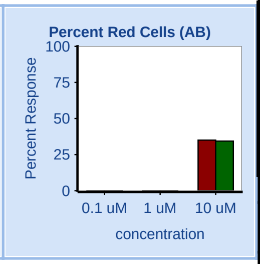

Average Response

Dose	Green Signal	Actual Pct Green Cells	Normalized Pct Green Cells	Red Signal	Actual Pct Red Cells	Normalized Pct Red Cells
0.1 uM	84.79	0.78	8.69	81.07	0.03	-56.09
1 uM	80.38	0.74	-285.58	84.39	0.04	-23.44
10 uM	121.18	0.93	110.69	109.34	0.19	34.70

Compound	Normalized Values (A and B Sets)	Responses

VU 0357121

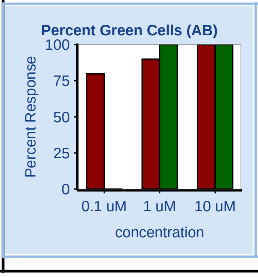

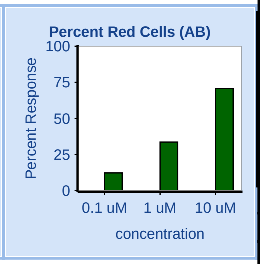

Average Response

Dose	Green Signal	Actual Pct Green Cells	Normalized Pct Green Cells	Red Signal	Actual Pct Red Cells	Normalized Pct Red Cells
0.1 uM	89.78	0.81	6.98	97.27	0.11	-3.72
1 uM	107.42	0.89	97.40	102.79	0.13	0.46
10 uM	105.08	0.94	355.87	106.64	0.15	10.87

Compound	Normalized Values (A and B Sets)	Responses

VU 0361737

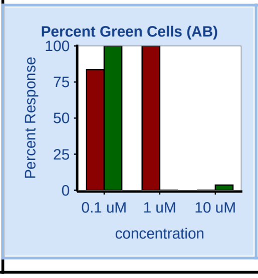

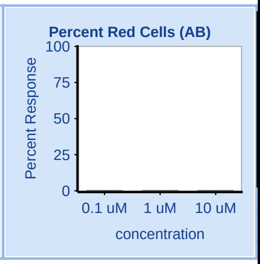

Average Response

Dose	Green Signal	Actual Pct Green Cells	Normalized Pct Green Cells	Red Signal	Actual Pct Red Cells	Normalized Pct Red Cells
0.1 uM	98.75	0.93	117.61	77.23	0.02	-52.31
1 uM	90.98	0.80	56.69	78.50	0.04	-48.52
10 uM	85.72	0.73	-25.60	96.01	0.09	-18.31

Compound	Normalized Values (A and B Sets)	Responses

VU 0364439

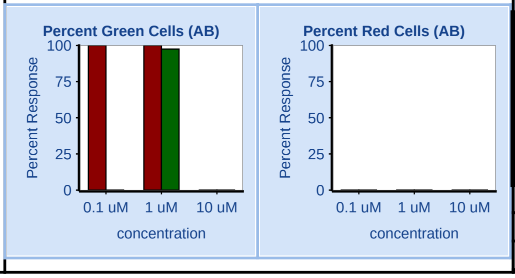

Average Response

Dose	Green Signal	Actual Pct Green Cells	Normalized Pct Green Cells	Red Signal	Actual Pct Red Cells	Normalized Pct Red Cells
0.1 uM	77.30	0.66	-88.11	73.51	0.02	-61.68
1 uM	103.53	0.92	235.24	79.89	0.03	-34.52
10 uM	78.30	0.74	-63.16	88.22	0.07	-19.98

Compound	Normalized Values (A and B Sets)	Responses

VU 0364770

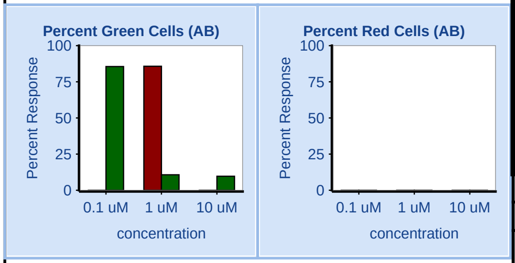

Average Response

Dose	Green Signal	Actual Pct Green Cells	Normalized Pct Green Cells	Red Signal	Actual Pct Red Cells	Normalized Pct Red Cells
0.1 uM	89.84	0.87	0.51	65.50	0.01	-56.54
1 uM	86.62	0.79	48.23	69.73	0.01	-62.02
10 uM	79.18	0.65	-143.81	98.19	0.07	-24.89

Compound	Normalized Values (A and B Sets)	Responses

VUF 10166

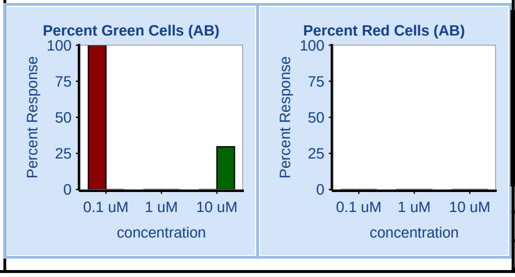

Average Response

Dose	Green Signal	Actual Pct Green Cells	Normalized Pct Green Cells	Red Signal	Actual Pct Red Cells	Normalized Pct Red Cells
0.1 uM	77.21	0.70	33.39	63.24	0.01	-61.04
1 uM	78.96	0.63	-65.59	83.15	0.05	-43.02
10 uM	82.71	0.72	-69.79	71.86	0.02	-52.10

Compound	Normalized Values (A and B Sets)	Responses

VX-222 (VCH-222, Lomibuvir)

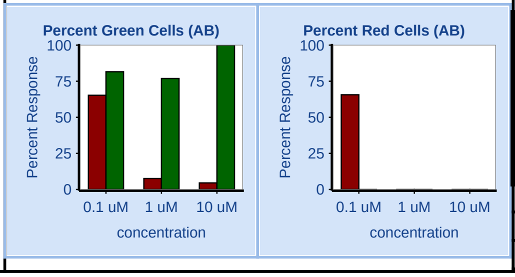

Average Response

Dose	Green Signal	Actual Pct Green Cells	Normalized Pct Green Cells	Red Signal	Actual Pct Red Cells	Normalized Pct Red Cells
0.1 uM	98.02	0.88	73.42	119.18	0.17	8.37
1 uM	103.41	0.85	42.24	88.53	0.05	-32.98
10 uM	96.81	0.82	70.14	106.86	0.07	-22.81

Compound	Normalized Values (A and B Sets)	Responses

VX-661

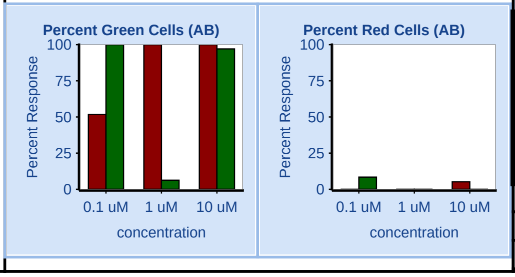

Average Response

Dose	Green Signal	Actual Pct Green Cells	Normalized Pct Green Cells	Red Signal	Actual Pct Red Cells	Normalized Pct Red Cells
0.1 uM	96.54	0.90	81.01	90.32	0.09	-18.74
1 uM	99.07	0.83	78.17	89.23	0.06	-35.06
10 uM	101.26	0.90	133.77	95.44	0.08	-17.35

Compound	Normalized Values (A and B Sets)	Responses

VX-745

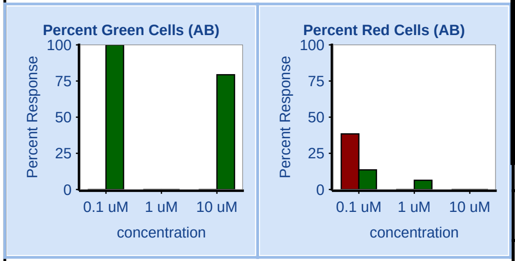

Average Response						
Dose	Green Signal	Actual Pct Green Cells	Normalized Pct Green Cells	Red Signal	Actual Pct Red Cells	Normalized Pct Red Cells
0.1 uM	90.36	0.83	43.88	115.55	0.16	26.00
1 uM	84.06	0.74	-614.67	92.61	0.09	-2.25
10 uM	79.35	0.71	-104.07	90.88	0.07	-19.14

Compound	Normalized Values (A and B Sets)	Responses

VX-765

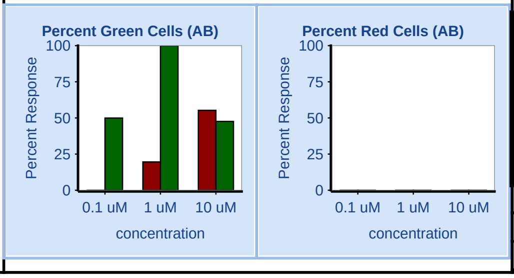

Average Response						
Dose	Green Signal	Actual Pct Green Cells	Normalized Pct Green Cells	Red Signal	Actual Pct Red Cells	Normalized Pct Red Cells
0.1 uM	82.23	0.75	-36.43	80.72	0.04	-36.86
1 uM	94.74	0.81	61.32	70.28	0.01	-49.76
10 uM	97.33	0.88	51.44	85.89	0.04	-38.35

Compound	Normalized Values (A and B Sets)	Responses

VX-809 (Lumacaftor)

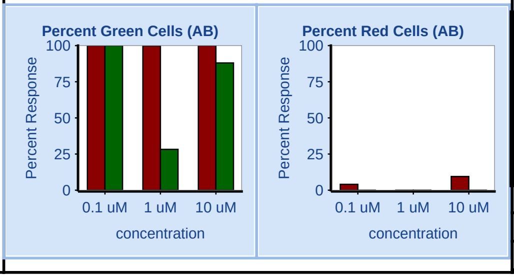

Average Response						
Dose	Green Signal	Actual Pct Green Cells	Normalized Pct Green Cells	Red Signal	Actual Pct Red Cells	Normalized Pct Red Cells
0.1 uM	113.88	0.95	149.75	97.66	0.09	-30.47
1 uM	101.45	0.84	64.05	85.71	0.03	-42.18
10 uM	116.23	0.89	108.90	104.81	0.09	-17.96

Compound	Normalized Values (A and B Sets)	Responses

WAY-600

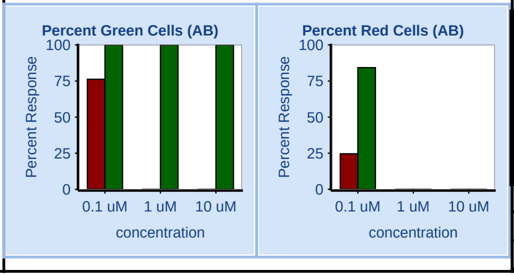

Average Response						
Dose	Green Signal	Actual Pct Green Cells	Normalized Pct Green Cells	Red Signal	Actual Pct Red Cells	Normalized Pct Red Cells
0.1 uM	94.73	0.89	95.97	123.68	0.23	54.46
1 uM	92.57	0.77	50.67	62.67	0.02	-47.67
10 uM	108.01	0.91	-22.29	79.23	0.03	-42.52

Compound	Normalized Values (A and B Sets)	Responses

WAY-100635 Maleate

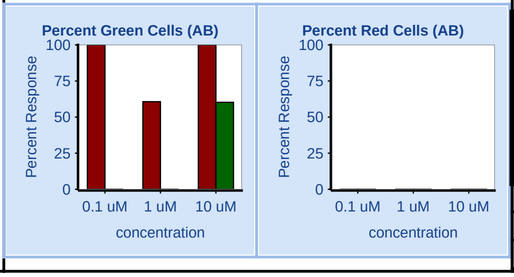

Average Response						
Dose	Green Signal	Actual Pct Green Cells	Normalized Pct Green Cells	Red Signal	Actual Pct Red Cells	Normalized Pct Red Cells
0.1 uM	98.20	0.84	35.95	86.23	0.05	-30.04
1 uM	84.44	0.70	-13.18	75.86	0.02	-47.24
10 uM	97.76	0.90	228.65	94.81	0.06	-30.01

Compound	Normalized Values (A and B Sets)	Responses
**WHI-P154**	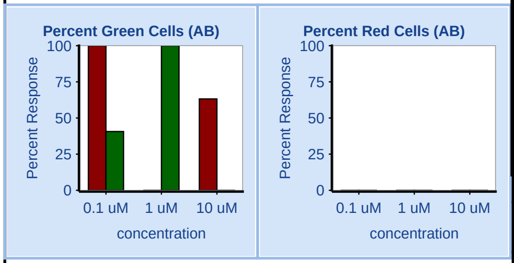	see table below

Average Response

Dose	Green Signal	Actual Pct Green Cells	Normalized Pct Green Cells	Red Signal	Actual Pct Red Cells	Normalized Pct Red Cells
0.1 uM	95.39	0.87	81.83	79.29	0.03	-55.16
1 uM	98.55	0.89	-105.33	80.48	0.04	-28.99
10 uM	82.44	0.81	1.52	86.11	0.07	-22.18

Compound	Normalized Values (A and B Sets)	Responses
**Wnt-C59 (C59)**	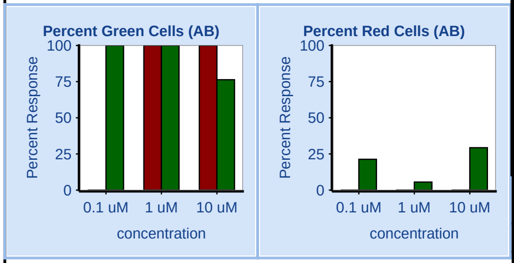	see table below

Average Response

Dose	Green Signal	Actual Pct Green Cells	Normalized Pct Green Cells	Red Signal	Actual Pct Red Cells	Normalized Pct Red Cells
0.1 uM	92.77	0.80	-443.11	95.96	0.10	-10.99
1 uM	107.07	0.89	127.23	94.50	0.10	-17.94
10 uM	104.92	0.89	141.44	110.26	0.13	5.73

Compound	Normalized Values (A and B Sets)	Responses
**WP1066**	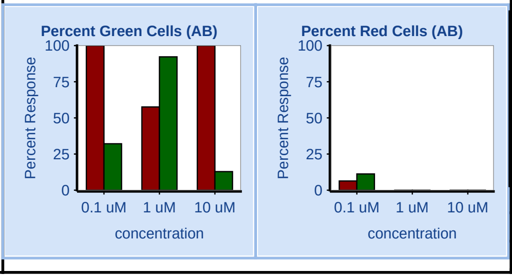	see table below

Average Response

Dose	Green Signal	Actual Pct Green Cells	Normalized Pct Green Cells	Red Signal	Actual Pct Red Cells	Normalized Pct Red Cells
0.1 uM	103.10	0.88	79.66	105.52	0.14	8.80
1 uM	97.94	0.84	74.90	84.63	0.04	-36.78
10 uM	98.98	0.90	258.51	104.10	0.08	-21.62

Compound	Normalized Values (A and B Sets)	Responses
**WYE-354**	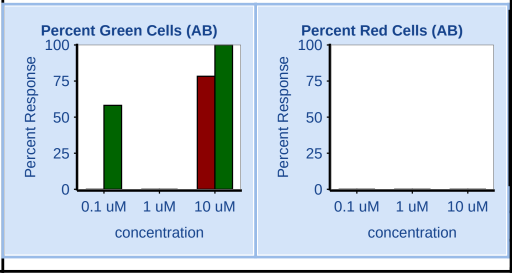	see table below

Average Response

Dose	Green Signal	Actual Pct Green Cells	Normalized Pct Green Cells	Red Signal	Actual Pct Red Cells	Normalized Pct Red Cells
0.1 uM	87.08	0.79	-23.35	80.84	0.02	-61.95
1 uM	80.04	0.69	-70.33	80.00	0.02	-50.35
10 uM	91.40	0.87	93.83	96.53	0.03	-40.11

Compound	Normalized Values (A and B Sets)	Responses
**WYE-125132 (WYE-132)**	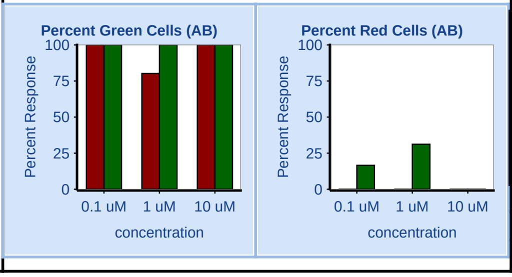	see table below

Average Response

Dose	Green Signal	Actual Pct Green Cells	Normalized Pct Green Cells	Red Signal	Actual Pct Red Cells	Normalized Pct Red Cells
0.1 uM	104.79	0.95	153.54	105.70	0.12	0.73
1 uM	109.84	0.91	115.58	105.42	0.15	9.49
10 uM	106.84	0.94	407.92	96.81	0.08	-22.66

Compound	Normalized Values (A and B Sets)	Responses

WZ811

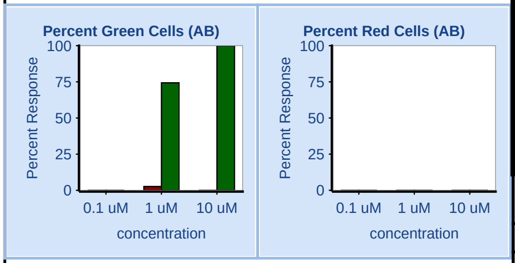

Average Response						
Dose	Green Signal	Actual Pct Green Cells	Normalized Pct Green Cells	Red Signal	Actual Pct Red Cells	Normalized Pct Red Cells
0.1 uM	73.64	0.64	-159.84	60.20	0.01	-58.71
1 uM	89.87	0.77	38.50	72.58	0.02	-59.51
10 uM	98.83	0.86	49.93	59.93	0.00	-61.59

Compound	Normalized Values (A and B Sets)	Responses

WZ3146

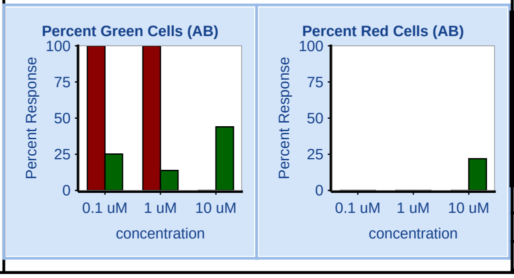

Average Response						
Dose	Green Signal	Actual Pct Green Cells	Normalized Pct Green Cells	Red Signal	Actual Pct Red Cells	Normalized Pct Red Cells
0.1 uM	92.69	0.84	64.42	72.28	0.02	-61.65
1 uM	100.54	0.85	93.74	95.14	0.06	-28.23
10 uM	103.51	0.62	-57.78	104.00	0.12	0.08

Compound	Normalized Values (A and B Sets)	Responses

WZ4002

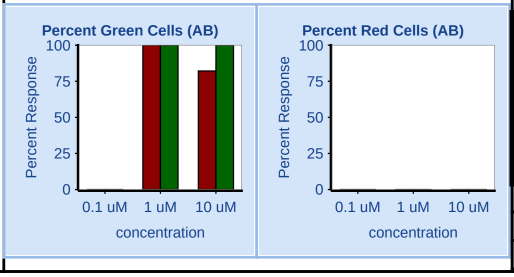

Average Response						
Dose	Green Signal	Actual Pct Green Cells	Normalized Pct Green Cells	Red Signal	Actual Pct Red Cells	Normalized Pct Red Cells
0.1 uM	87.20	0.77	-3.46	90.55	0.06	-39.95
1 uM	107.12	0.92	128.98	78.05	0.03	-44.47
10 uM	100.02	0.87	95.35	93.05	0.03	-38.31

Compound	Normalized Values (A and B Sets)	Responses

WZ8040

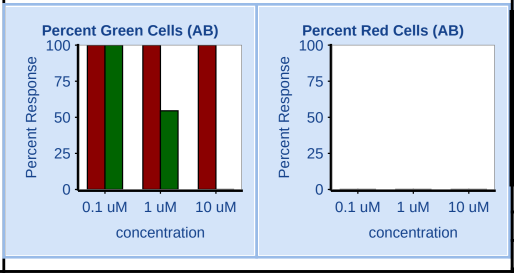

Average Response						
Dose	Green Signal	Actual Pct Green Cells	Normalized Pct Green Cells	Red Signal	Actual Pct Red Cells	Normalized Pct Red Cells
0.1 uM	106.24	0.92	117.44	93.53	0.06	-45.32
1 uM	104.24	0.88	109.66	80.14	0.02	-47.55
10 uM	96.97	0.82	48.43	90.96	0.02	-46.76

Compound	Normalized Values (A and B Sets)	Responses

XAV-939

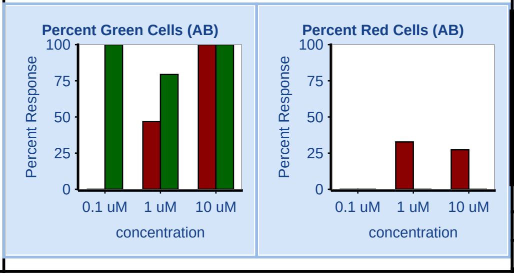

Average Response						
Dose	Green Signal	Actual Pct Green Cells	Normalized Pct Green Cells	Red Signal	Actual Pct Red Cells	Normalized Pct Red Cells
0.1 uM	98.41	0.85	25.33	97.34	0.09	-25.65
1 uM	102.31	0.86	63.10	111.52	0.13	0.92
10 uM	109.83	0.91	120.25	102.55	0.11	-13.87

Compound	Normalized Values (A and B Sets)	Responses

XL019

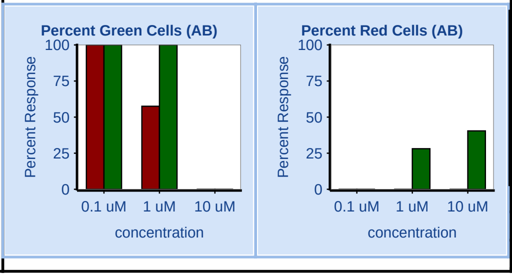

Average Response						
Dose	Green Signal	Actual Pct Green Cells	Normalized Pct Green Cells	Red Signal	Actual Pct Red Cells	Normalized Pct Red Cells
0.1 uM	96.01	0.92	171.23	70.43	0.02	-53.95
1 uM	103.29	0.86	106.22	98.10	0.11	-12.22
10 uM	83.76	0.69	-79.90	104.42	0.13	3.67

Compound	Normalized Values (A and B Sets)	Responses

XL147

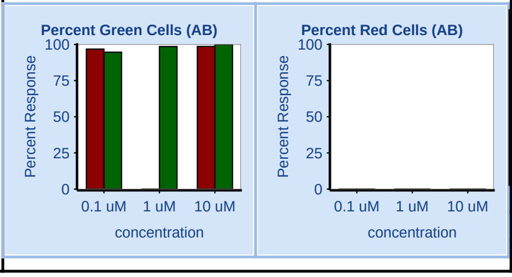

Average Response						
Dose	Green Signal	Actual Pct Green Cells	Normalized Pct Green Cells	Red Signal	Actual Pct Red Cells	Normalized Pct Red Cells
0.1 uM	100.02	0.90	95.74	80.59	0.03	-56.35
1 uM	94.41	0.82	-19.23	107.94	0.11	-7.50
10 uM	102.30	0.89	108.70	102.03	0.06	-28.01

Compound	Normalized Values (A and B Sets)	Responses

XL335

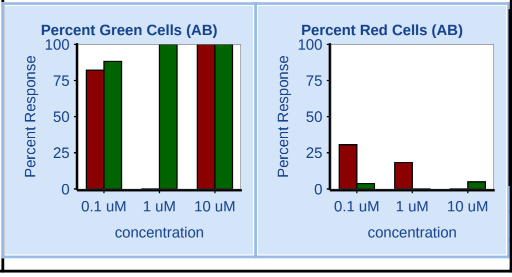

Average Response						
Dose	Green Signal	Actual Pct Green Cells	Normalized Pct Green Cells	Red Signal	Actual Pct Red Cells	Normalized Pct Red Cells
0.1 uM	99.27	0.88	85.25	104.99	0.16	17.15
1 uM	96.50	0.76	44.66	102.89	0.13	1.39
10 uM	110.17	0.95	441.62	105.49	0.12	-3.41

Compound	Normalized Values (A and B Sets)	Responses

XL388

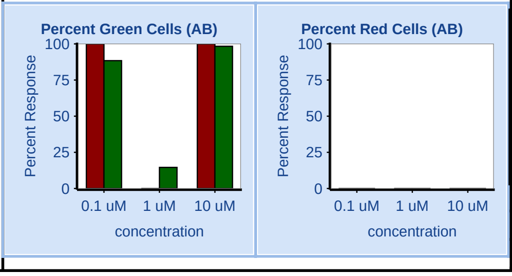

Average Response						
Dose	Green Signal	Actual Pct Green Cells	Normalized Pct Green Cells	Red Signal	Actual Pct Red Cells	Normalized Pct Red Cells
0.1 uM	107.09	0.92	250.95	77.79	0.04	-44.91
1 uM	88.51	0.72	3.12	79.35	0.05	-40.30
10 uM	104.41	0.91	145.66	93.69	0.08	-19.34

Compound	Normalized Values (A and B Sets)	Responses

XL888

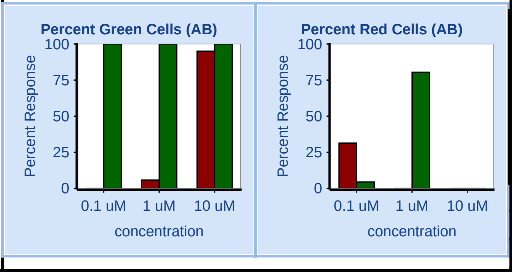

Average Response						
Dose	Green Signal	Actual Pct Green Cells	Normalized Pct Green Cells	Red Signal	Actual Pct Red Cells	Normalized Pct Red Cells
0.1 uM	128.25	0.89	50.16	108.87	0.16	17.87
1 uM	109.87	0.81	68.32	93.21	0.16	14.15
10 uM	117.83	0.91	114.59	100.83	0.10	-9.18

Compound | Normalized Values (A and B Sets) | Responses

Xylazine HCl

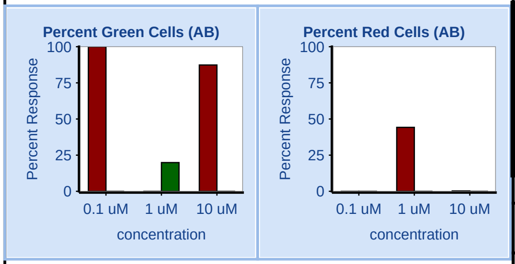

Average Response

Dose	Green Signal	Actual Pct Green Cells	Normalized Pct Green Cells	Red Signal	Actual Pct Red Cells	Normalized Pct Red Cells
0.1 uM	81.17	0.68	-72.95	87.53	0.05	-41.46
1 uM	89.90	0.82	-196.07	105.61	0.15	8.52
10 uM	82.34	0.71	-83.92	86.47	0.08	-13.74

Compound | Normalized Values (A and B Sets) | Responses

Y-27632 2HCl

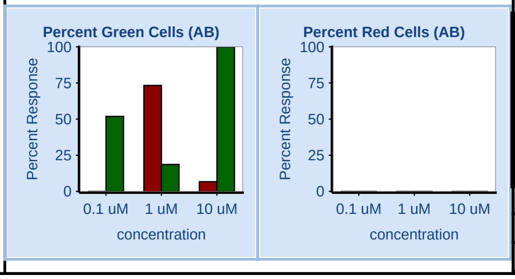

Average Response

Dose	Green Signal	Actual Pct Green Cells	Normalized Pct Green Cells	Red Signal	Actual Pct Red Cells	Normalized Pct Red Cells
0.1 uM	88.74	0.79	-15.39	85.53	0.05	-45.82
1 uM	91.94	0.82	45.97	101.44	0.06	-27.69
10 uM	95.56	0.80	54.46	99.26	0.06	-24.95

Compound | Normalized Values (A and B Sets) | Responses

YM155 (Sepantronium Bromide)

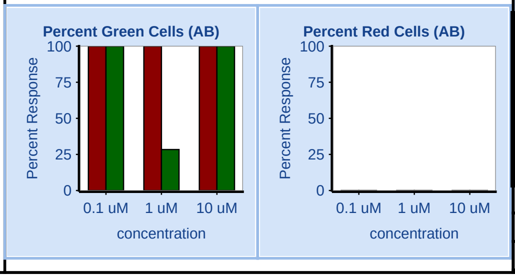

Average Response

Dose	Green Signal	Actual Pct Green Cells	Normalized Pct Green Cells	Red Signal	Actual Pct Red Cells	Normalized Pct Red Cells
0.1 uM	106.55	0.95	130.30	99.01	0.04	-55.10
1 uM	95.57	0.85	72.24	83.78	0.03	-44.81
10 uM	101.60	0.88	104.40	99.56	0.05	-30.26

Compound | Normalized Values (A and B Sets) | Responses

YO-01027

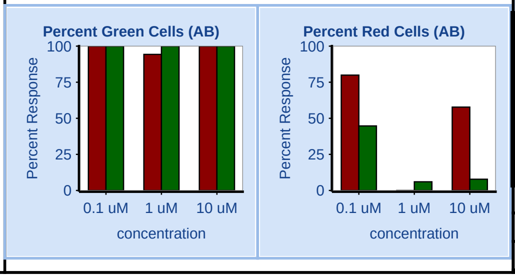

Average Response

Dose	Green Signal	Actual Pct Green Cells	Normalized Pct Green Cells	Red Signal	Actual Pct Red Cells	Normalized Pct Red Cells
0.1 uM	104.68	0.92	132.30	126.72	0.25	62.32
1 uM	114.36	0.92	119.68	98.55	0.12	-2.15
10 uM	111.98	0.95	334.78	121.27	0.19	32.78

Compound | Normalized Values (A and B Sets) | Responses

Z-VAD-FMK

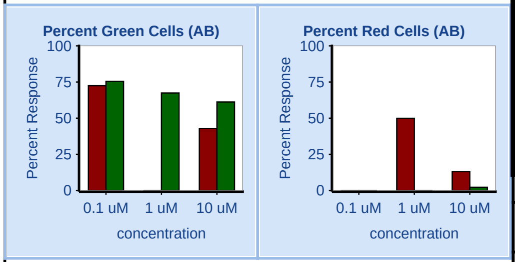

Average Response

Dose	Green Signal	Actual Pct Green Cells	Normalized Pct Green Cells	Red Signal	Actual Pct Red Cells	Normalized Pct Red Cells
0.1 uM	96.57	0.86	73.95	93.54	0.08	-27.19
1 uM	88.80	0.78	-596.59	99.99	0.14	2.33
10 uM	94.24	0.87	52.05	99.16	0.13	7.64

Compound | Normalized Values (A and B Sets) | Responses

Zaltoprofen

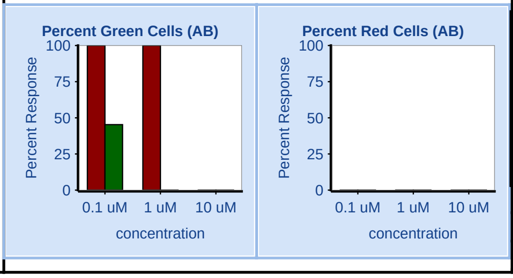

Average Response						
Dose	Green Signal	Actual Pct Green Cells	Normalized Pct Green Cells	Red Signal	Actual Pct Red Cells	Normalized Pct Red Cells
0.1 uM	89.64	0.87	138.57	82.22	0.04	-40.48
1 uM	91.03	0.76	23.32	83.18	0.04	-48.99
10 uM	84.83	0.71	-29.74	76.27	0.03	-46.38

Compound | Normalized Values (A and B Sets) | Responses

Zanamivir

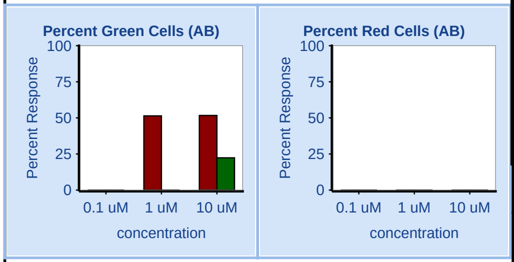

Average Response						
Dose	Green Signal	Actual Pct Green Cells	Normalized Pct Green Cells	Red Signal	Actual Pct Red Cells	Normalized Pct Red Cells
0.1 uM	82.34	0.76	-120.15	65.40	0.01	-58.94
1 uM	68.48	0.51	-167.85	73.71	0.02	-56.17
10 uM	88.93	0.78	37.02	89.79	0.06	-32.98

Compound | Normalized Values (A and B Sets) | Responses

ZCL278

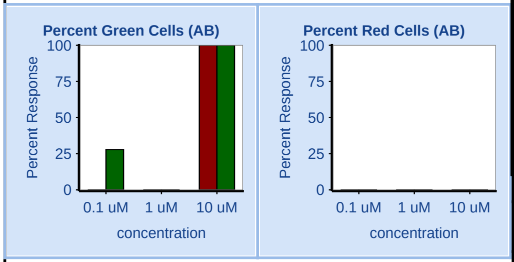

Average Response						
Dose	Green Signal	Actual Pct Green Cells	Normalized Pct Green Cells	Red Signal	Actual Pct Red Cells	Normalized Pct Red Cells
0.1 uM	85.00	0.77	-5.89	76.38	0.02	-62.37
1 uM	87.64	0.81	-153.67	79.76	0.04	-32.34
10 uM	96.19	0.93	114.12	72.48	0.04	-33.02

Compound | Normalized Values (A and B Sets) | Responses

Zebularine

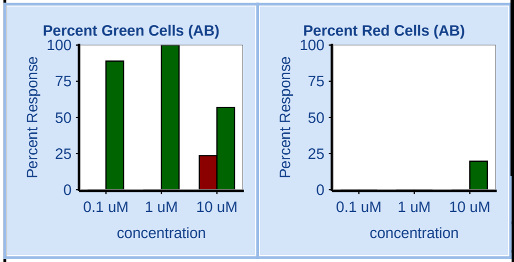

Average Response						
Dose	Green Signal	Actual Pct Green Cells	Normalized Pct Green Cells	Red Signal	Actual Pct Red Cells	Normalized Pct Red Cells
0.1 uM	85.39	0.82	-219.80	73.39	0.03	-49.49
1 uM	103.61	0.81	69.38	84.61	0.07	-31.98
10 uM	92.23	0.81	40.17	104.25	0.12	-2.05

Compound | Normalized Values (A and B Sets) | Responses

Zibotentan (ZD4054)

Average Response						
Dose	Green Signal	Actual Pct Green Cells	Normalized Pct Green Cells	Red Signal	Actual Pct Red Cells	Normalized Pct Red Cells
0.1 uM	100.64	0.88	71.68	115.57	0.14	6.73

Compound	Normalized Values (A and B Sets)	Responses

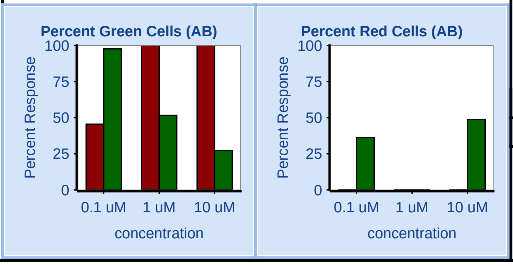

1 uM	109.91	0.87	93.08	99.11	0.09	-17.00
10 uM	99.14	0.83	66.97	125.16	0.17	21.33

Compound	Normalized Values (A and B Sets)	Responses

Zinc Pyrithione

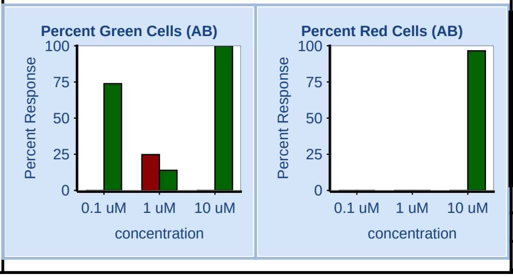

Average Response

Dose	Green Signal	Actual Pct Green Cells	Normalized Pct Green Cells	Red Signal	Actual Pct Red Cells	Normalized Pct Red Cells
0.1 uM	87.46	0.84	-85.46	81.94	0.06	-34.06
1 uM	90.74	0.75	19.34	88.84	0.08	-23.44
10 uM	85.32	0.85	43.99	124.12	0.15	13.52

Compound	Normalized Values (A and B Sets)	Responses

ZM 39923 HCl

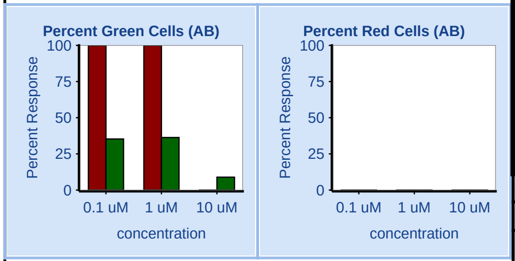

Average Response

Dose	Green Signal	Actual Pct Green Cells	Normalized Pct Green Cells	Red Signal	Actual Pct Red Cells	Normalized Pct Red Cells
0.1 uM	101.46	0.87	84.50	84.76	0.03	-54.46
1 uM	100.92	0.88	196.28	77.66	0.05	-21.66
10 uM	77.93	0.71	-105.19	78.97	0.04	-31.64

Compound	Normalized Values (A and B Sets)	Responses

ZM 306416

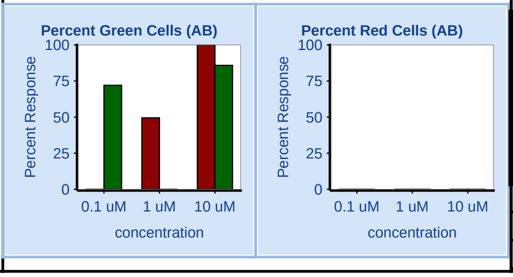

Average Response

Dose	Green Signal	Actual Pct Green Cells	Normalized Pct Green Cells	Red Signal	Actual Pct Red Cells	Normalized Pct Red Cells
0.1 uM	89.37	0.85	-18.91	75.50	0.04	-42.15
1 uM	89.67	0.75	20.43	75.03	0.03	-52.41
10 uM	93.25	0.89	129.47	65.03	0.01	-57.51

Compound	Normalized Values (A and B Sets)	Responses

ZM 323881 HCl

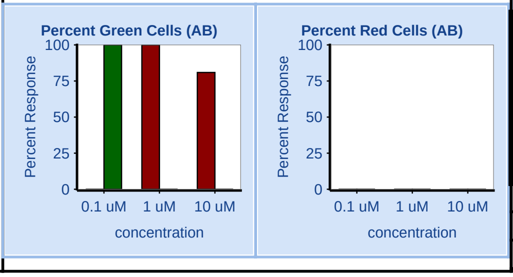

Average Response

Dose	Green Signal	Actual Pct Green Cells	Normalized Pct Green Cells	Red Signal	Actual Pct Red Cells	Normalized Pct Red Cells
0.1 uM	89.84	0.87	-10.88	66.54	0.01	-60.41
1 uM	91.51	0.81	58.17	69.60	0.01	-62.58
10 uM	80.57	0.72	20.91	75.63	0.03	-47.99

Compound	Normalized Values (A and B Sets)	Responses
**ZM 336372**	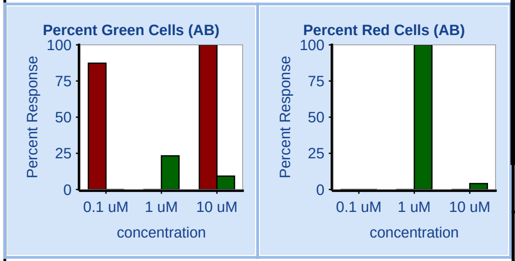	see table below

Average Response

Dose	Green Signal	Actual Pct Green Cells	Normalized Pct Green Cells	Red Signal	Actual Pct Red Cells	Normalized Pct Red Cells
0.1 uM	67.90	0.51	-217.92	71.17	0.01	-66.85
1 uM	86.25	0.78	-472.55	107.34	0.20	71.09
10 uM	91.01	0.88	62.34	85.31	0.08	-14.60

Compound	Normalized Values (A and B Sets)	Responses
**Zosuquidar (LY335979) 3HCl**	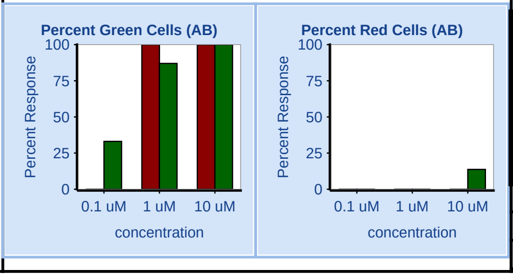	see table below

Average Response

Dose	Green Signal	Actual Pct Green Cells	Normalized Pct Green Cells	Red Signal	Actual Pct Red Cells	Normalized Pct Red Cells
0.1 uM	89.67	0.80	12.21	95.09	0.06	-37.50
1 uM	112.10	0.90	114.86	93.24	0.07	-26.34
10 uM	111.38	0.92	126.47	117.14	0.12	-0.80
