# Supplementary figures and images for: GSK3β regulates epithelial-mesenchymal transition and cancer stem cell properties in triple-negative breast cancer
Source: Breast Cancer Res. 2019 Mar 7;21:37. doi: 10.1186/s13058-019-1125-0 (PMC6407242; doi:10.1186/s13058-019-1125-0)

Supplementary Figure 1

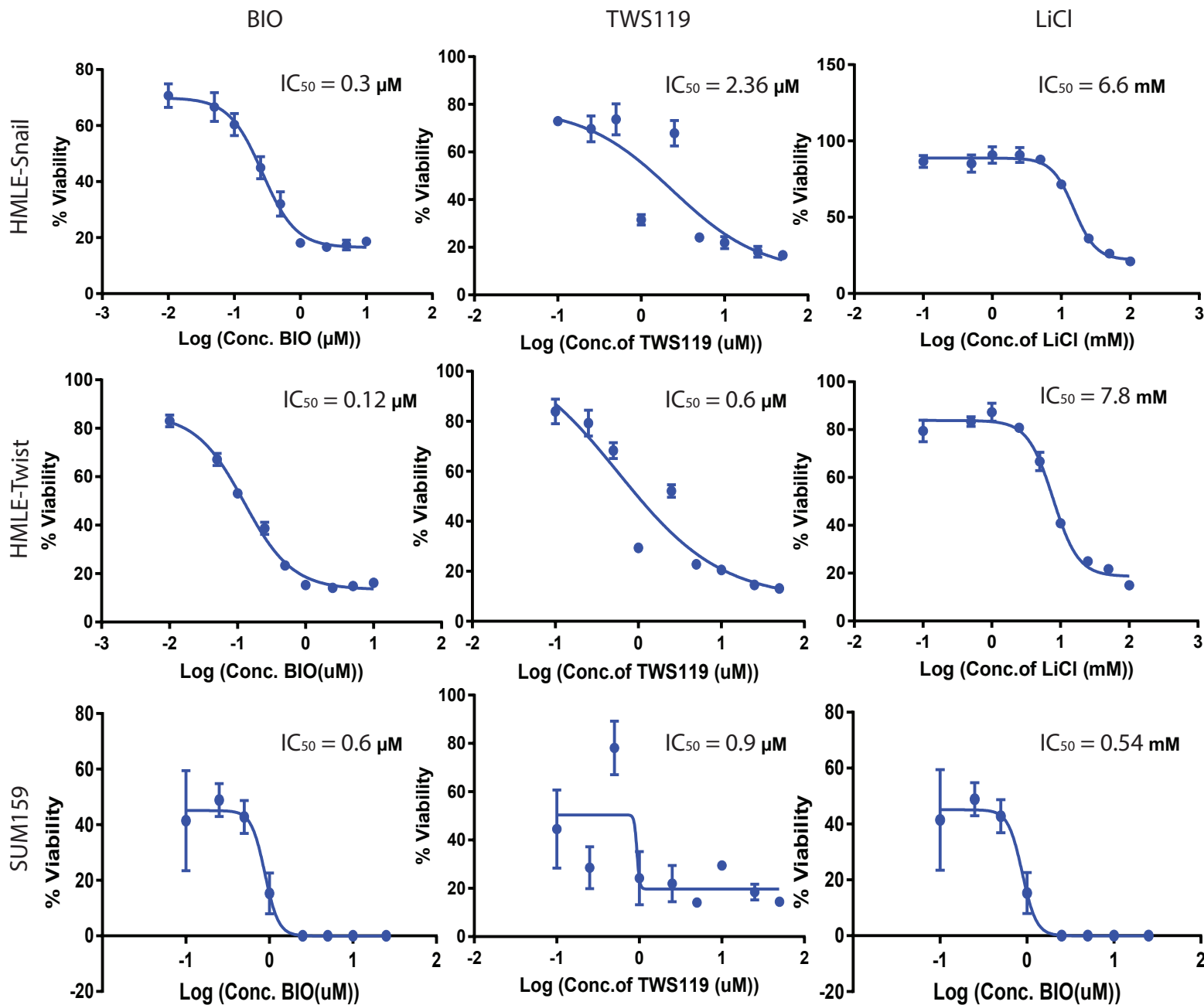

Supplement: Supplementary file 3 — Figure S1. The IC50 of the 3 drugs were calculated for the 3 mesenchymal-like cell lines used in this study. (PDF 122 kb) [file 13058_2019_1125_MOESM3_ESM.pdf]

Supplementary Figure 2

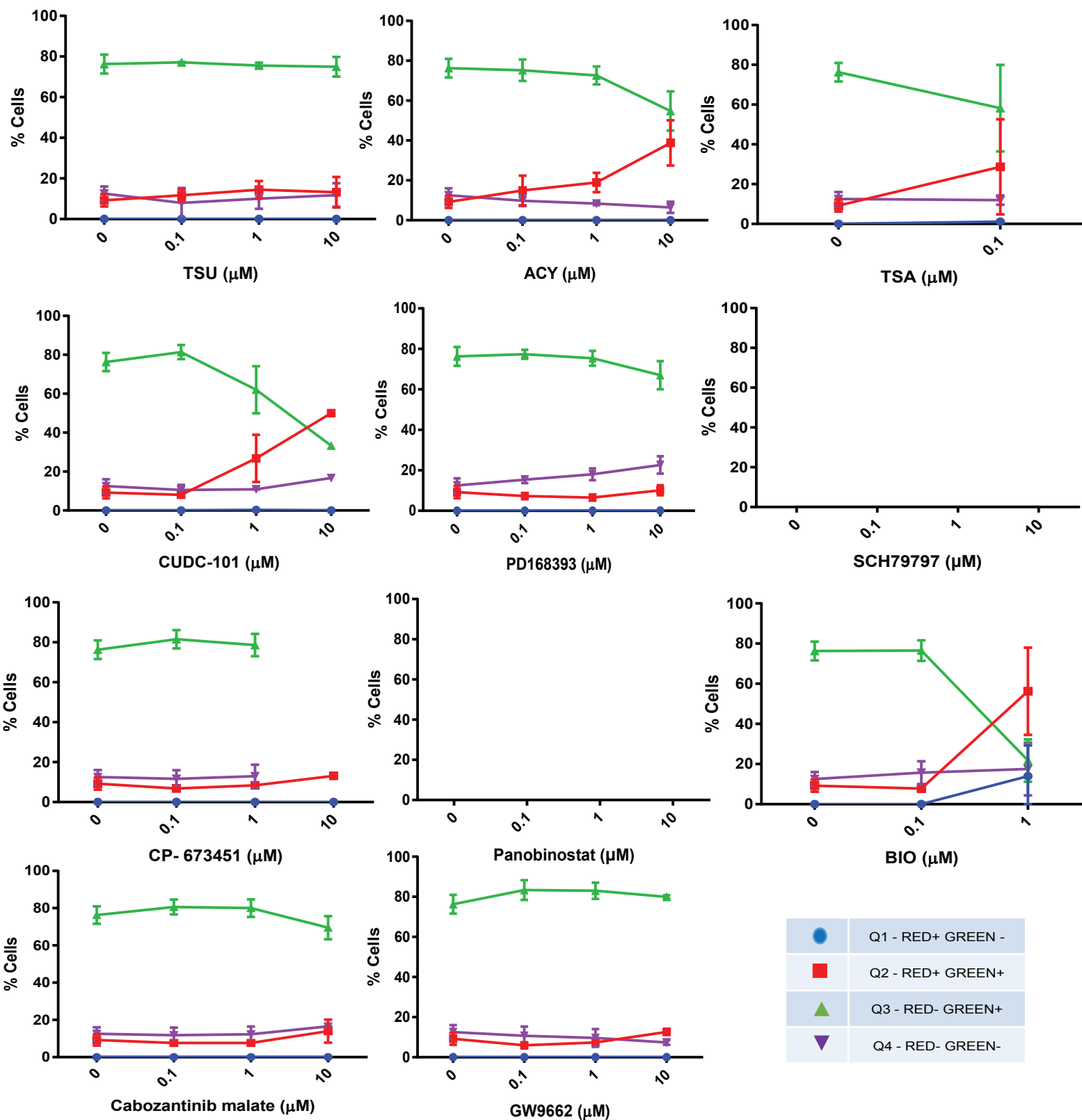

Supplement: Supplementary file 4 — Figure S2. The drugs that were selected from the screen were validated using FACS. The MDA MB 231 reporter cells were treated with all the 11 drugs selected from the screen. Following treatment, the percentage of cells fluorescing red and green were assessed using flow cytometry and plotted for each of the three concentrations to generate the graphs. (PDF 160 kb) [file 13058_2019_1125_MOESM4_ESM.pdf]

Supplementary Figure 3

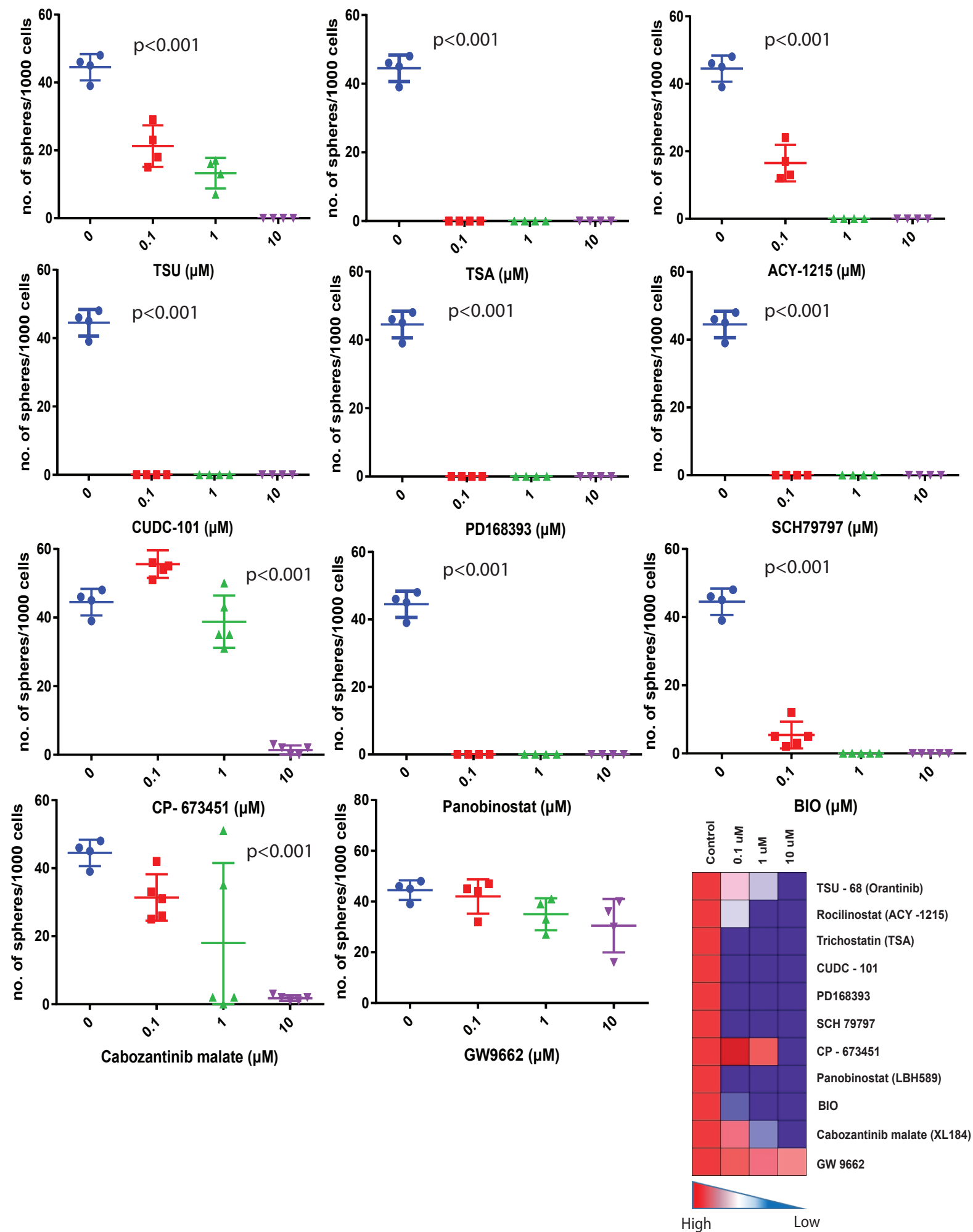

Supplement: Supplementary file 5 — Figure S3. GSK3β inhibitor, BIO, is one of the drugs that is capable of inhibiting the sphere forming ability of mesenchymal MDA-MB-231 cells. The drugs that were selected from the screen were used to treat the mammosphere assay. MDA MB 231 reporter cells were grown in ultra-low attachments plates in mammosphere media for 10 days. The number of mammospheres was counted and graphed, and BIO was one of the drugs that decreased the sphere forming ability of the reporter MDA MB 231 cells. The heatmap summarizes the mammosphere data showing that BIO is one of the drugs that decreases the sphere-forming ability of the MDA MB 231 reporter cells. (PDF 141 kb) [file 13058_2019_1125_MOESM5_ESM.pdf]

Supplementary Figure 4

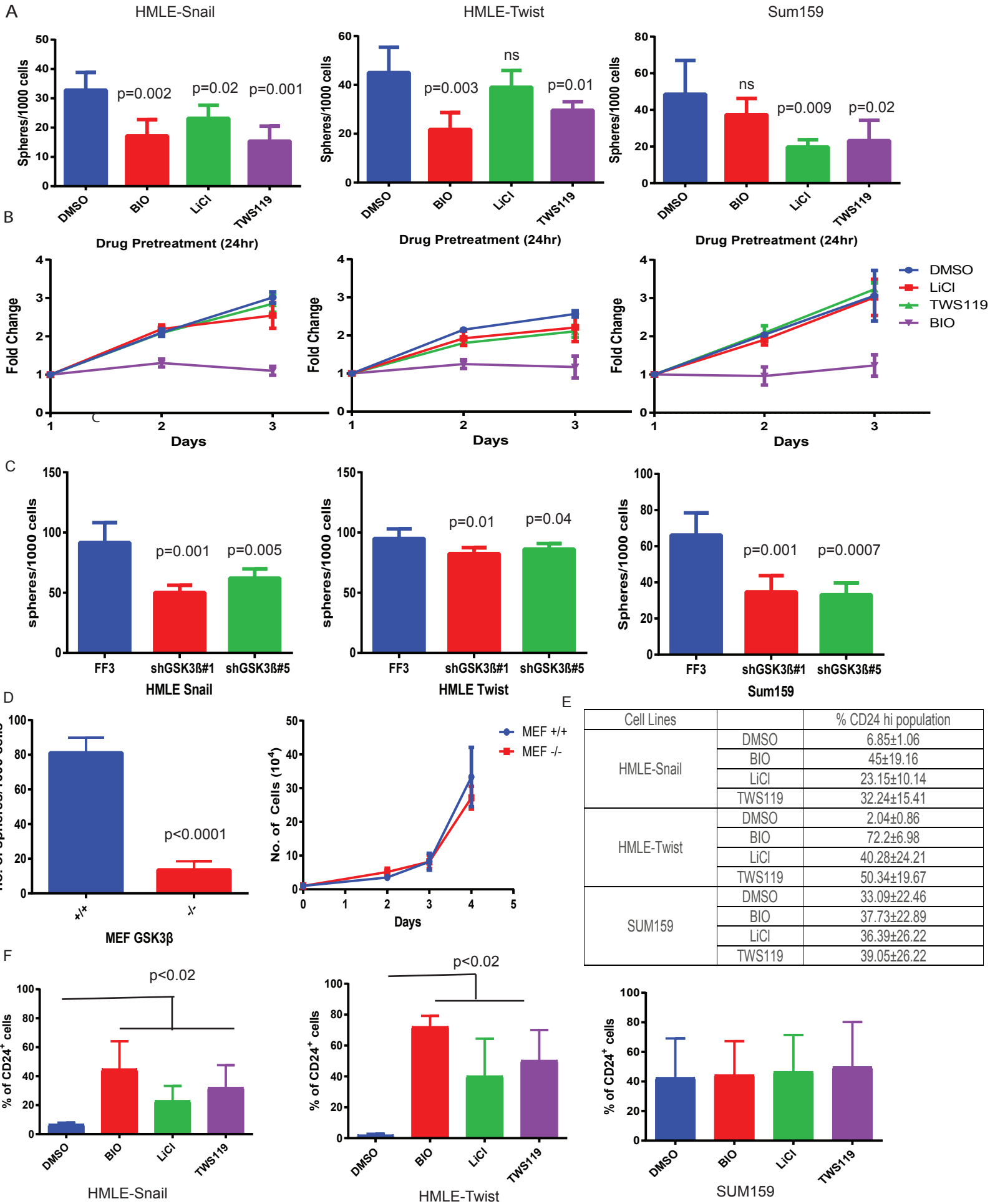

Supplement: Supplementary file 6 — Figure S4. Genetic suppression of GSK3β expression decreases the sphere-forming potential of mesenchymal-like cells. (A) Cells with mesenchymal properties were treated with the 3 GSK3β inhibitors for 24 h. Following the treatment, the cells were plated for mammosphere assays and (B) a growth curve was generated to ensure that decrease in proliferation is not the reason for the decreased sphere forming ability of these cells. (C) Knockdown of GSK3β decreases the mammosphere forming capability of the mesenchymal cells. HMLE Snail, HMLE Twist, and Sum159 cells were stably transfected with GSK3β shRNA and grown in ultra-low attachments plates in mammosphere media for 10 days. (D). Mouse embryonic fibroblasts (MEFs) in which GSK3β were knocked out were grown in ultra-low attachment plates in mammosphere media for 10 days. Mouse embryonic fibroblasts (MEFs) in which GSK3β was knocked out were grown for 4 days, and growth was assessed on days 2, 3, and 4. Knocking out of GSK3β in MEFs reduces the sphere forming potential of the MEFs. The cells with mesenchymal properties were treated with 3 GSK3β inhibitors and the change in the CD24/44 profile of these cells following treatment was quantified and represented as a (E) table and (F) bar graph. (PDF 153 kb) [file 13058_2019_1125_MOESM6_ESM.pdf]

Supplementary Figure 5

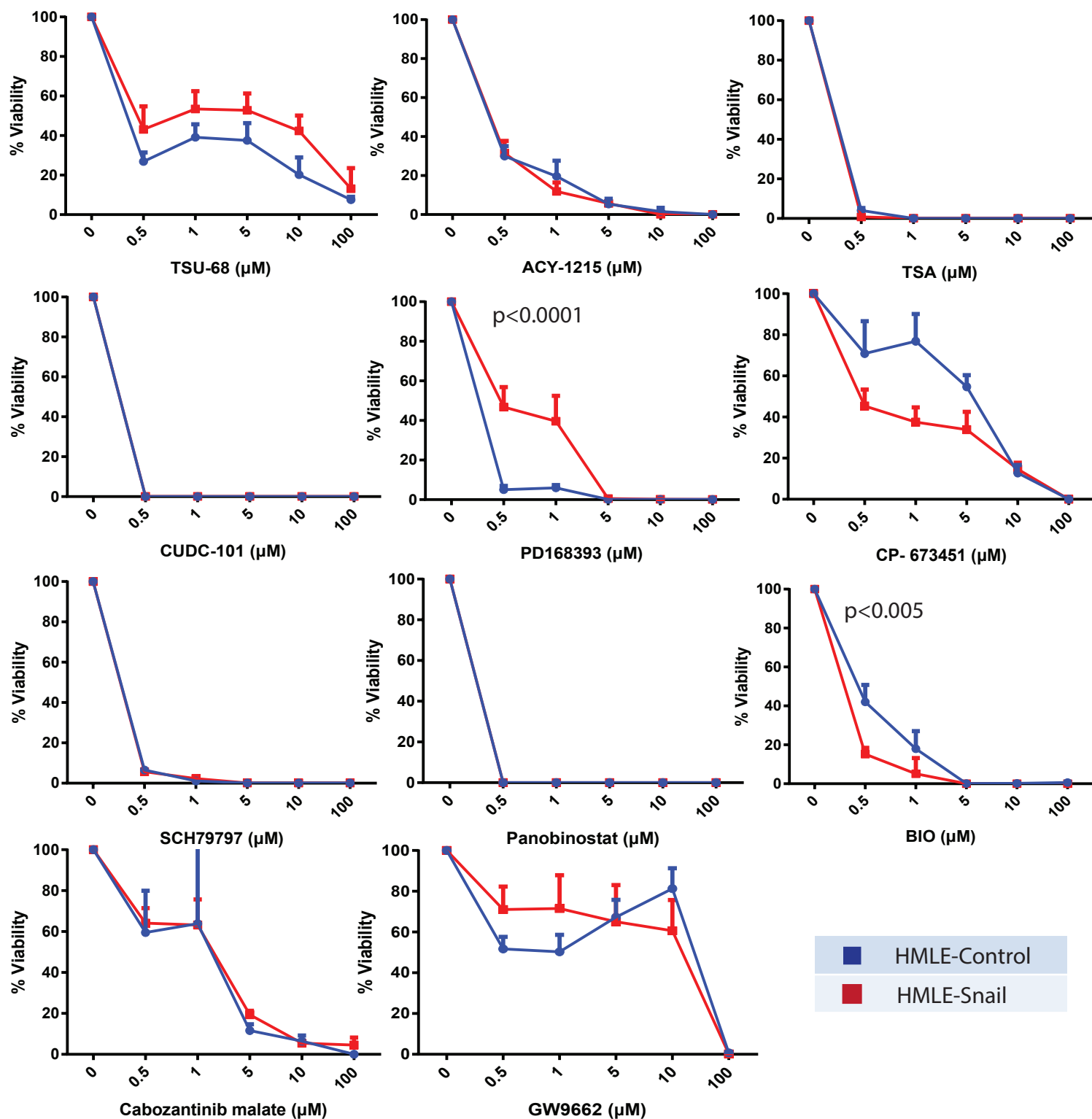

Supplement: Supplementary file 7 — Figure S5. HMLE-vector and HMLE-Snail cells were treated with a dose range of the tested inhibitors, and viability was assessed by MTT assay. Of the drugs that were shortlisted from the screen, BIO was one of the drugs that could selectively inhibit HMLE-Snail cells with mesenchymal phenotype more efficiently as compared to HMLE-vector cells with epithelial phenotype. (PDF 133 kb) [file 13058_2019_1125_MOESM7_ESM.pdf]

Supplementary Figure 6

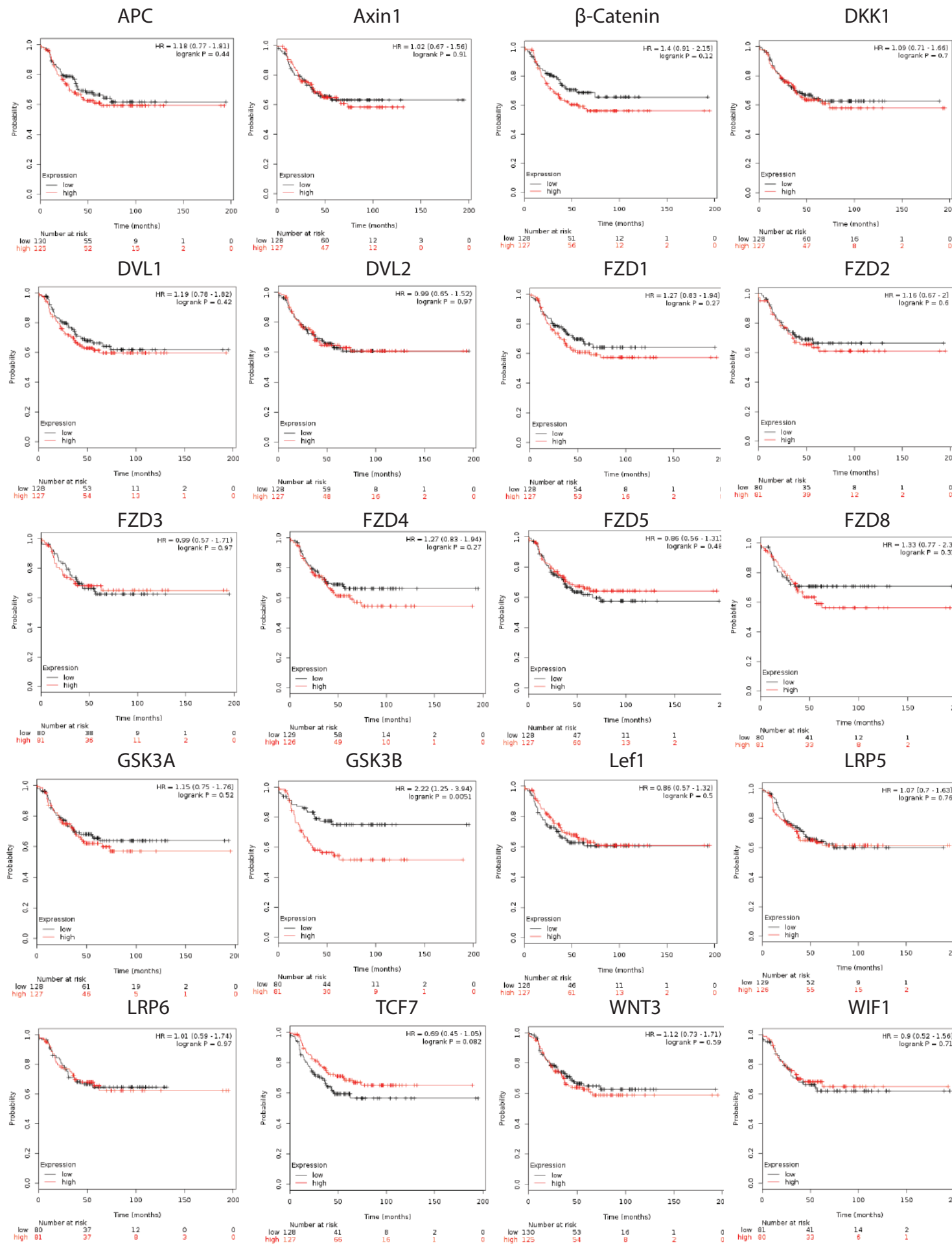

Supplement: Supplementary file 8 — Figure S6. KmPlots were generated for several major players of the Wnt signaling pathway using the KmPlotter. Of all the different players, GSK3β was the only gene, the upregulation of which significantly correlated with worse survival in TNBCs. (PDF 322 kb) [file 13058_2019_1125_MOESM8_ESM.pdf]

Supplementary figure 7

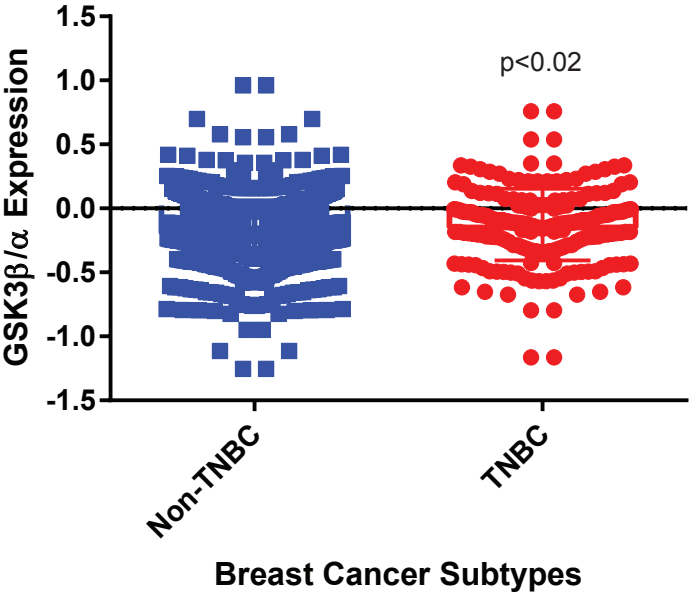

Supplement: Supplementary file 9 — Figure S7. TCGA RPPA data was mined to compare the expression of GSK3β in TNBCs and other types of breast cancer. The analysis of these data revealed a significant increase in the expression of GSK3 in TNBCs as compared to the other types of breast cancer. (PDF 139 kb) [file 13058_2019_1125_MOESM9_ESM.pdf]

Supplementary figure 8

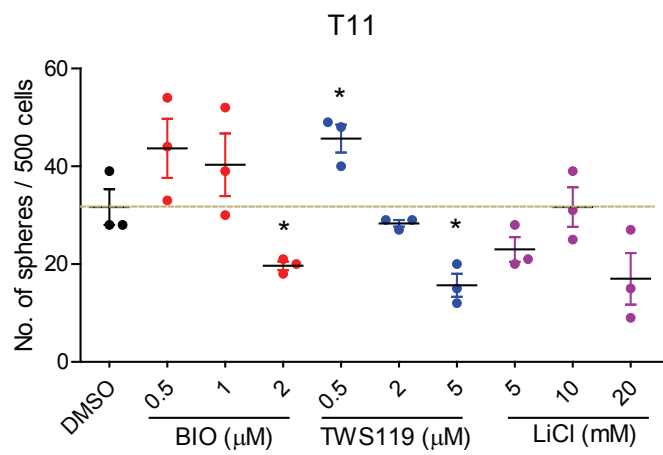

Supplement: Supplementary file 10 — Figure S8. Claudin-low T11 cells were grown in ultra-low attachment plates in mammosphere media for 10 days in the presence of 3 GSK3β inhibitors. The numbers of mammospheres were counted and graphed (n = 3, p values were calculated using Student’s unpaired two-tailed t test). (PDF 94 kb) [file 13058_2019_1125_MOESM10_ESM.pdf]
